# Synthesis of Hydrofluoroolefin‐Based Iodonium Reagent via Dyotropic Rearrangement and Its Utilization in Fluoroalkylation

**DOI:** 10.1002/anie.202208420

**Published:** 2022-08-09

**Authors:** János T. Csenki, Balázs L. Tóth, Ferenc Béke, Bálint Varga, Péter P. Fehér, András Stirling, Zsuzsanna Czégény, Attila Bényei, Zoltán Novák

**Affiliations:** ^1^ ELTE “Lendület” Catalysis and Organic Synthesis Research Group Department Institute of Chemistry Eötvös Loránd University Pázmány Péter stny. 1/A 1117 Budapest Hungary; ^2^ Research Centre for Natural Sciences Eötvös Loránd Research Network Magyar Tudósok körútja 2 1117 Budapest Hungary; ^3^ Department of Chemistry Eszterházy Károly Catholic University Leányka u. 6 3300 Eger Hungary; ^4^ Department of Physical Chemistry University of Debrecen Egyetem tér 1 4032 Debrecen Hungary

**Keywords:** Amines, Fluoroalkylation, Heterocycles, Iodonium Salts, Rearrangement

## Abstract

[1,2]‐shift of atoms in alkyl fragments belongs to the class of dyotropic rearrangements. Various atoms, including halogens can be involved in the migration, however participation of iodine is unprecedented. Herein, we report our experimental and DFT studies on the oxidation triggered dyotropic rearrangement of iodo and chloro functions via butterfly‐type transition state to demonstrate the migrating ability of λ^3^‐iodane centre. With the exploitation of dyotropic rearrangement we designed and synthesized a novel fluoroalkyl iodonium reagent from industrial feedstock gas HFO‐1234yf. We demonstrated that the hypervalent reagent serves as an excellent fluoroalkylation agent for various amines and nitrogen heterocycles.

## Graphical abstract

The synthesis of a novel hypervalent iodonium salt was developed with the exploitation of a dyotropic rearrangement of iodine and chlorine functions in fluoroalkyl chains from HFO‐1234yf gas. The electrophilic reagent enabled the direct and metal‐free introduction of a special fluoroalkyl group into aniline derivatives and N‐containing heteroaromatic compounds. Detailed experimental and computational data were collected and used to propose a mechanistic description of this unique rearrangement.

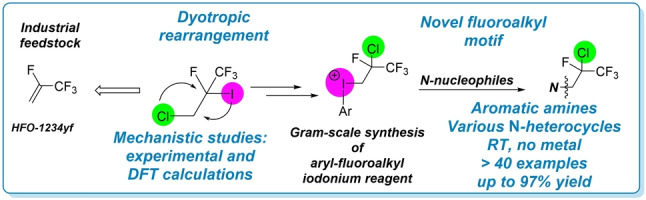

The design of novel fluorinated motifs is important,^[1]^ and the development of synthetic methods for the synthesis of fluorinated compounds are in the focus of organic chemistry,^[2]^ pharmaceutical and agrochemical research,^[3]^ due to the beneficial effect of the fluorine atom on the chemical, physical or biological properties of organic molecules.^[4]^ However, the extension of the existing palette of these fluorous functional groups often requires new industrial feedstocks and transformations. In this regard, hydrofluoroolefin HFO‐1234yf (**1**) gas^[5]^ is a perfect choice, because it is commonly used as 4^th^ generation refrigerants in air conditioners, due to its short atmospheric lifetime, decreased Ozone Depleting Potential (ODP) and Global Warming Potential (GWP) values compared to traditional CFCs. Moreover, it is cheap and available as bulk chemical, and the presence of electron‐deficient carbon–carbon double bond in their structure makes these industrial gases ideal fluorinated feedstocks for organic synthesis.^[6]^ In our current research program, we study the transformability of HFOs to valuable chemical reagents for organic syntheses and medicinal chemistry applications.

Dyotropic rearrangements, defined by Reetz,^[7]^ represent an interesting subclass of rearrangement reactions,^[8]^ where intramolecular migration of two vicinal atoms attached to *sp* ^*3*^ carbon centers through formal [1,2]‐shift is possible (Type I dyotropic rearrangements). The migration of neighboring atoms could occur simultaneously through four membered bicyclic transition state, however stepwise and radical mechanisms could be also reasonable (Scheme 1A).[^7d^, ^9^]

**Scheme 1:**
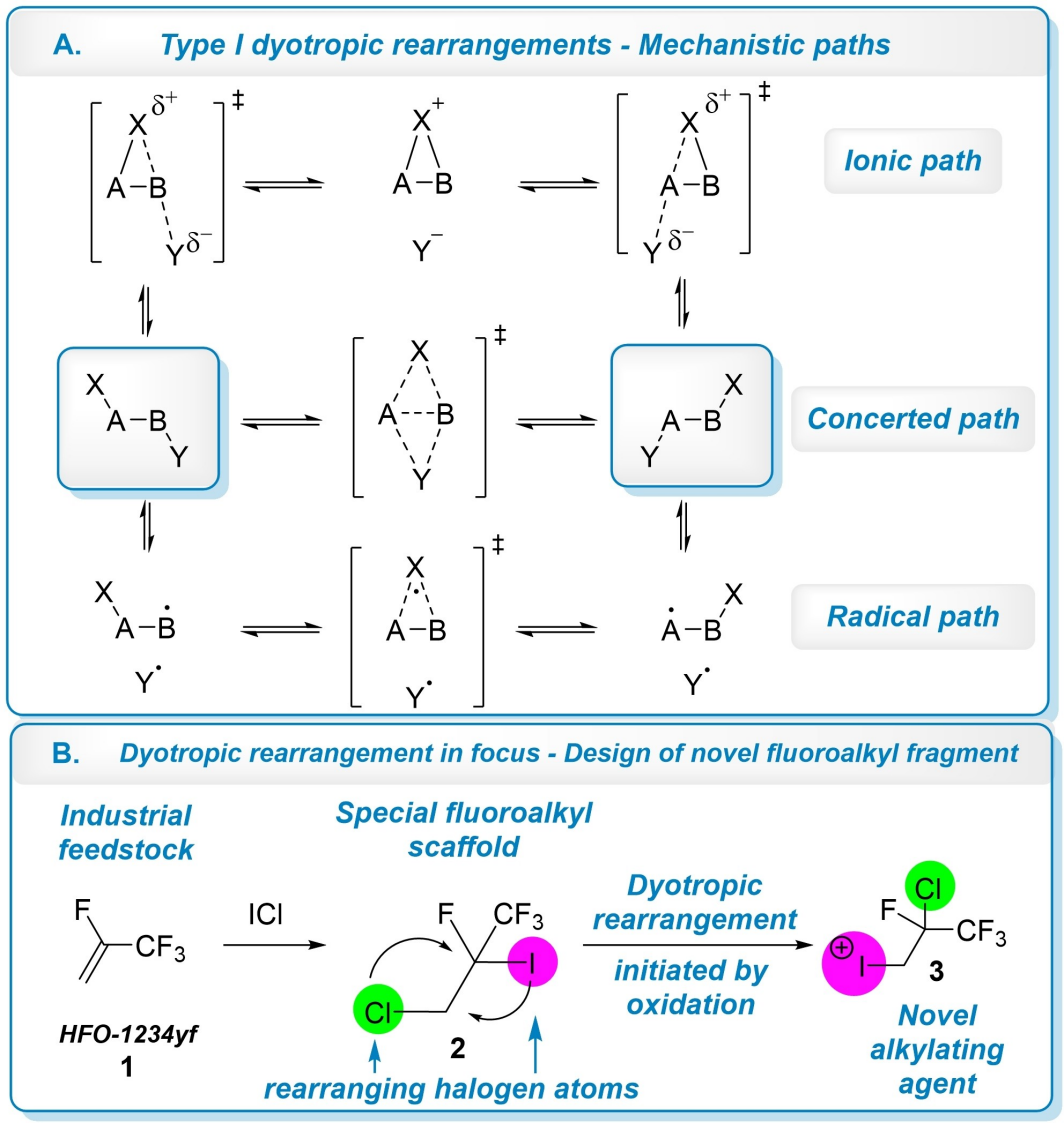
Type I dyotropic rearrangement paths and its synthetic exploitation.

Considering the atoms taking part in the rearrangement (X and Y on Scheme 1A), transition metals,^[10]^ carbon and oxygen could be involved in the migration,^[11]^ but [1,2]‐shift of halogens are also important examples of this reaction class, where fluoro, chloro and bromo atoms take part in the simultaneous migration.[^10b^, ^11e^, ^11f^, ^12^] However, to the best of our knowledge participation of iodine in dyotropic rearrangement is unprecedented, despite its expected highest migrating potential among the halogens.^[13]^ The closest example is a palladium catalyzed [1,2]‐iodine(III) shift in alkyne system described by Yoshikai.^[10b]^

In our work, we aimed to study the dyotropic rearrangement of polyhalogenated fluoroalkyl scaffold (**2**) to prepare novel iodonium reagent (**3**)^[14]^ for fluoroalkylation reactions (Scheme 1B). To achieve this, with the modification of our recently developed procedure,^[6i]^ we prepared the iodinated fluorous species (**2**) on multigram scale (218 g, 70 % yield, 95 % regioselectivity) from industrial feedstock HFO‐1234yf gas (**1**) as a potential substrate (**2**) for the study of a possible dyotropic rearrangement, due to the presence of various halogens in vicinal position.

As steric bulk is one of the driving forces of dyotropic rearrangement,^[12b]^ the oxidation of iodine centre to iodonium diacetate could induce the concerted [1,2]‐halogen shift. Provisional fluoroalkyliodonium diacetate enables the synthesis of novel aryl(fluoroalkyl)iodonium salts (**3**) in a subsequent transformation, which could be used as electrophilic reagent in various functionalizations. Thus, after the successful large‐scale synthesis of **2**, we studied the oxidation of the iodine centre under various reaction conditions (Scheme 2). Fluoroalkyl iodide **2** was treated with the oxidizing mixture of trifluoroacetic anhydride (TFAA), trifluoroacetic acid (HTFA) and H_2_O_2_ at −10 °C for 20 h. Then the oxidized intermediate **4** was reacted in situ with fluorobenzene in the presence of TfOH at 4 °C, but the formation of the desired aryl(fluoroalkyl)iodonium species **7**, was not observed. Instead, diaryliodonium salt **6** was isolated in 43 % yield. The product contained 5 % **10** as minor product (Entry 1), which could be originated from the minor regioisomer of the starting alkyl iodide **2**. Increasing the reaction temperature of the oxidation step (Step A) from −10 °C up to 55 °C (Entries 2–6) formation of **10** become dominant over **6**, which can be explained by the dyotropic rearrangement of **4** to **9** through the “butterfly‐type” transition state (**8**) (ratio of **10 : 6** shifted from 5 : 95 to 92 : 8, Entries 1–5). For our further studies we kept 35 °C as the optimal temperature, considering that we reached the highest yield of the rearranged iodonium product **10** (43 %, entry 4) in this case and further increase of the temperature caused lower yields (Entries 5 and 6). As the result of full reaction optimization,^[15]^ decreasing the amount of the hydrogen peroxide to 1.1 equivalent (Entry 7) enabled the selective formation of **10** and the pure reagent was isolated in 42 % yield on >10 g scale. The structure of **10** was confirmed by NMR and X‐ray crystallography (Scheme 2).^[16]^

**Scheme 2:**
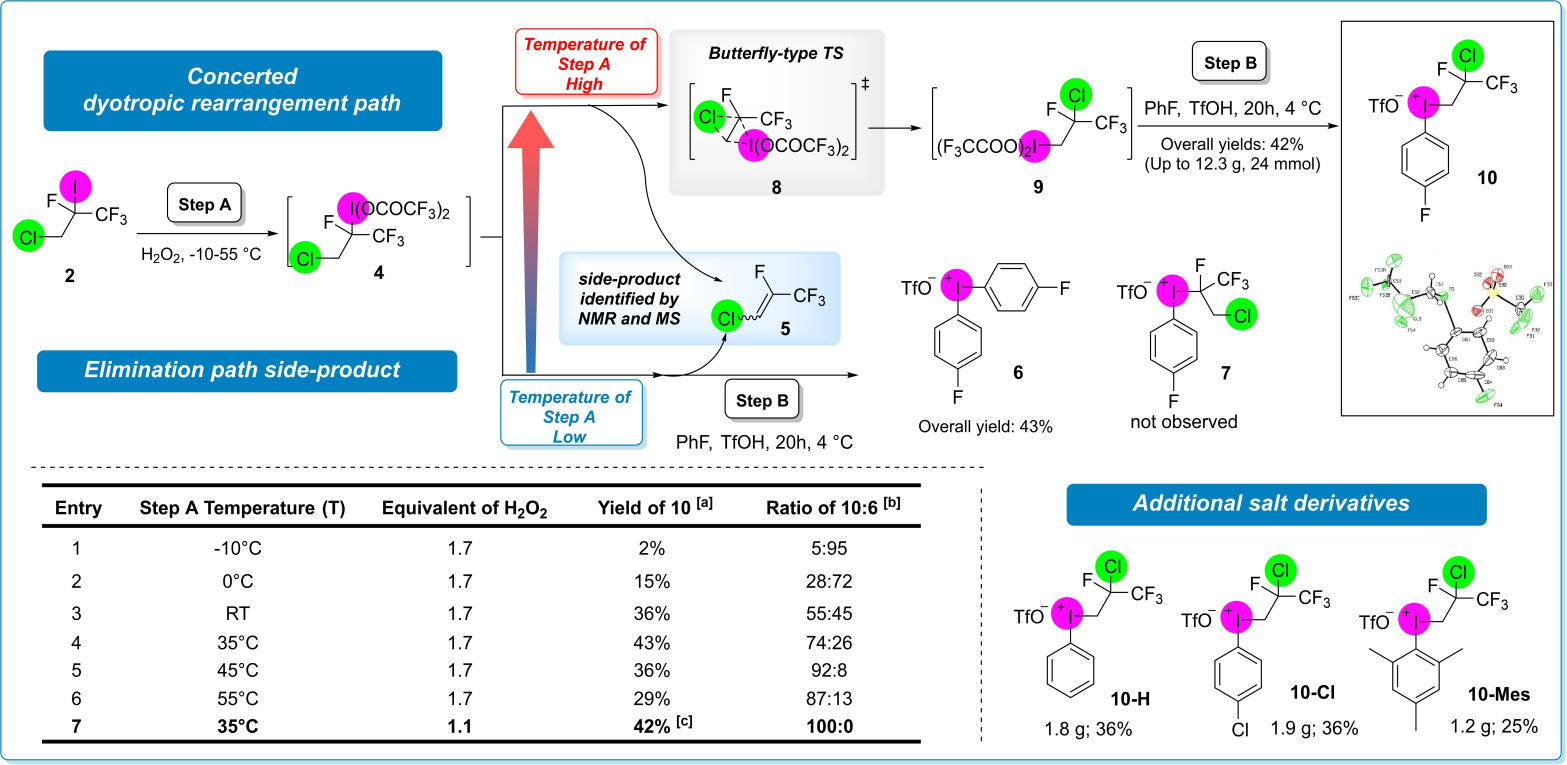
Optimization of reaction conditions for the synthesis of the iodonium salt. Reaction conditions: (Step A) trifluoroacetic anhydride (TFAA, 72 mmol, 7.2 equiv), trifluoroacetic acid (HTFA, 1 mmol, 0.1 equiv), 50w/w% H_2_O_2_, −10 °C, tetrafluoroalkyl iodide (10 mmol, 1.0 equiv), 20 h, *T*, (Step B) DCM (10 mL), PhF, TfOH, (10 mmol, 1 equiv), 20 h, 4 °C. [a] Mixture of **10** and **6** was isolated as product, yield of **10** was calculated on the basis of the NMR spectra of isolated product mixture. [b] Ratio of **10 : 6** was determined on the basis of NMR spectra of isolated product mixture. [c] Yield of pure isolated product **10**.

Based on the synthetic procedure additional derivatives of the iodonium reagent were prepared using different aromatic systems in Step B, and phenyl (**10‐H**), 4‐chlorophenyl (**10‐Cl**) and mesityl (**10‐Mes**) derivatives were prepared on gram‐scale in 36 %, 36 % and 25 % yield respectively. These yields were slightly lower compared to salt **10**, therefore we used the fluorophenyl derivative (**10**) for further study.

To reveal the mechanism of the reaction, we successfully isolated intermediate **4**, but we found that this intermediate is stable only below −10 °C. Redissolving compound **4** in TFA and stirring the mixture at 35 °C for 20 h, we were able to study its transformation to **9** with *in situ* NMR reaction monitoring at 35 °C, and isolated the more stable compound **9**, formed through the key dyotropic rearrangement reaction. The *in situ* monitoring studies revealed the formation of 1‐chloro‐2,3,3,3‐tetrafluoro‐prop‐1‐ene (**5**) as the product of a decomposition pathway, which is a competitive elimination reaction of the key dyotropic rearrangement, and could be responsible for the relatively low isolated yield of **10** (42 %). Coupled pyrolysis studies (Py‐GCMS) of isolated intermediates **4** and **9** also supported their relative stabilities, and we were able to detect the formation of gaseous fluoroolefin side product (**5**) during this thermoanalytical study.^[15]^

To elucidate the reaction mechanism of the dyotropic rearrangement, we have also performed Density Functional Theory (DFT) calculations.^[15]^ The first step of the reaction is the oxidation of **2** to the iodonium intermediate **4** with hydrogen peroxide. However, the additional TFAA, HTFA and the residual water produce an oxidation mixture where several possible reactions need to be considered (Figure SI6.1).^[15]^ Throughout these steps water is produced, but it can react immediately with the excess TFAA to produce HTFA in an exergonic reaction (Figure SI6.1)^[15]^ This implies that water does not participate in the rest of the mechanism. The next major step is the isomerization of **4** to the terminal iodonium **9** through a barrier of 15.3 kcal mol^−1^ (Figure 1). In the transition state (**TS_8_** ), the Cl and I atoms simultaneously migrate in an antiperiplanar fashion. This corresponds to the concerted mechanism of Scheme 1A. We have also explored the ionic and radical mechanisms, but we found that these routes were unfavorable.^[15]^ We note that the experiments^[15]^ and also calculations have revealed an undesired side reaction where the *C1* carbon of the propane chain is deprotonated by one of the coordinated TFA anions yielding **5**. This step is highly exergonic because the entropy increases as the number of molecules increases and the reduction of iodine from oxidation state +3 to +1 is also favourable. Still, this side reaction features a higher reaction barrier (by 2.7 kcal mol^−1^). As the higher barrier towards the gaseous side product **5** is still accessible at the reaction temperature, albeit at a reduced rate, this can explain the acceptable ≈40 % yield of **10**. To calculate the activation barriers of these competing steps we have taken into account the fact that the basicity of the deprotonating TFA is strongly affected by its solvation in HTFA medium.^[15]^

**Figure 1:**
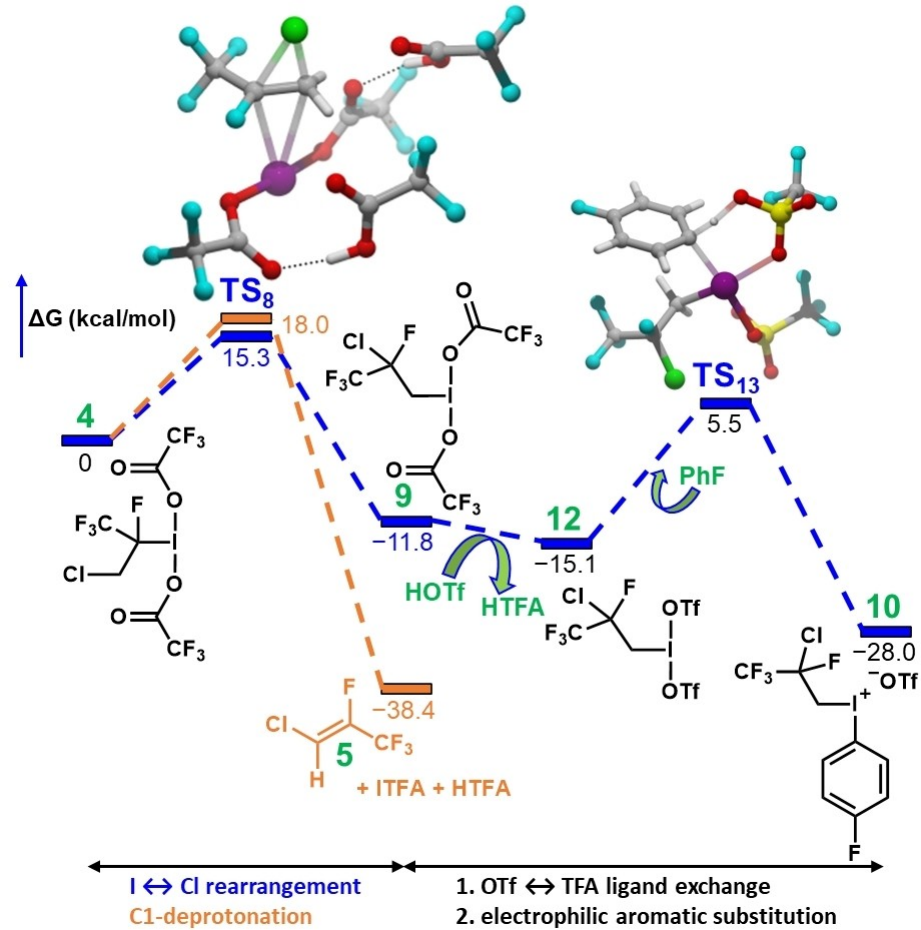
The energy profile of the proposed reaction mechanism.

The final part of the mechanism describes the formation of the final iodonium salt product **10**. When HOTf and fluorobenzene are added to **9**, the TFA anion ligands are replaced by triflate anions (**12**). In the encounter complex of **12** and PhF (Figure SI6.2)^[15]^ a facile aryl C−H functionalization occurs and leads to the final product **10**. The barrier of this step is 20.6 kcal mol^−1^ indicating rapid product formation at the reaction temperature. The experiments also indicate that the rearrangement can be suspended using low temperature where **4** converts to the diaryliodonium product **6**. This observation is in line with the calculated energy profiles of the side reactions leading to diaryliodonium formation (Figure SI6.3).^[15]^

After the study of dyotropic rearrangement, we tested the applicability of the new aryl(fluoroalkyl)iodonium salt (**10**) as an electrophilic reagent toward nitrogen nucleophiles to synthesize novel *N*‐fluoroalkylated molecules. For the optimization of the reaction conditions, we used 4′‐aminoacetophenone as the model substrate and studied the effect of solvent, base and stoichiometry on the transformation. We found that with the utilization of 1.1 equivalent iodonium salt and 1.2 equivalent of Li_2_CO_3_ in MeCN (0.3 M) ensures completion of the *N*‐fluoroalkylation at ambient temperature in case of our model substrate.^[15]^

Under the optimized conditions we studied the scope and limitation of the developed reaction with various aniline derivatives (**14**) (Scheme 3). First, the parent compound, aniline was fluoroalkylated and the product **15** was isolated in 88 % yield. The reactions proceeded smoothly with *ortho* (**16**–**22**), *meta* (**23**–**26**) and *para* (**27**–**35**) substituted aniline derivatives, in the presence of halogens, EWG or EDG groups and in most cases excellent yields up to 97 % were reached. A mild steric effect can be observed, when sterically more demanding *ortho* substituents are present in the aromatic system (**17**–**20**), and longer reaction time is required to the complete conversion of these substrates. It is worth noting that the presence of iodo substituent (**18**, **31**) did not seem to interfere with the reaction, and even the unprotected OH group (**22**, **26**, **35**) is completely orthogonal to the key transformation, and *N*‐selective alkylation took place. In addition, reaction of the silyl protected derivative (**25**) also took place with a similar efficiency. In the cases of the strongly electron‐donating groups (e.g. methoxy group, **36**–**40**), the starting materials were too reactive at ambient temperature and the products could be isolated only in low yields (about 30 %, not shown) due to decomposition. However, this problem was successfully solved by lowering the reaction temperature to 0 °C and significantly shortening the reaction time to 10–30 minutes. The lowest yield was observed in the case of *para*‐anisidine (**36**, 63 %), while the *meta*‐substituted compound (**38**, 92 %) gave the highest efficiency, demonstrating the influence of electronic effect caused by MeO group. This phenomenon was clearly observed in case of dimethoxy anilines. *Ortho*–*para* disubstituted substrate afforded product **39** in 28 % yield even at 0 °C, while 3,5‐dimethoxyaniline was *N*‐alkylated efficiently and **40** was obtained in excellent yield (92 %). The 2‐aminonaphthalene reacted smoothly and **41** was isolated in 92 % yield under the standard conditions. Secondary amine analogues were also successfully substituted (**42**–**44**), but only in 44–56 % yield range.

**Scheme 3:**
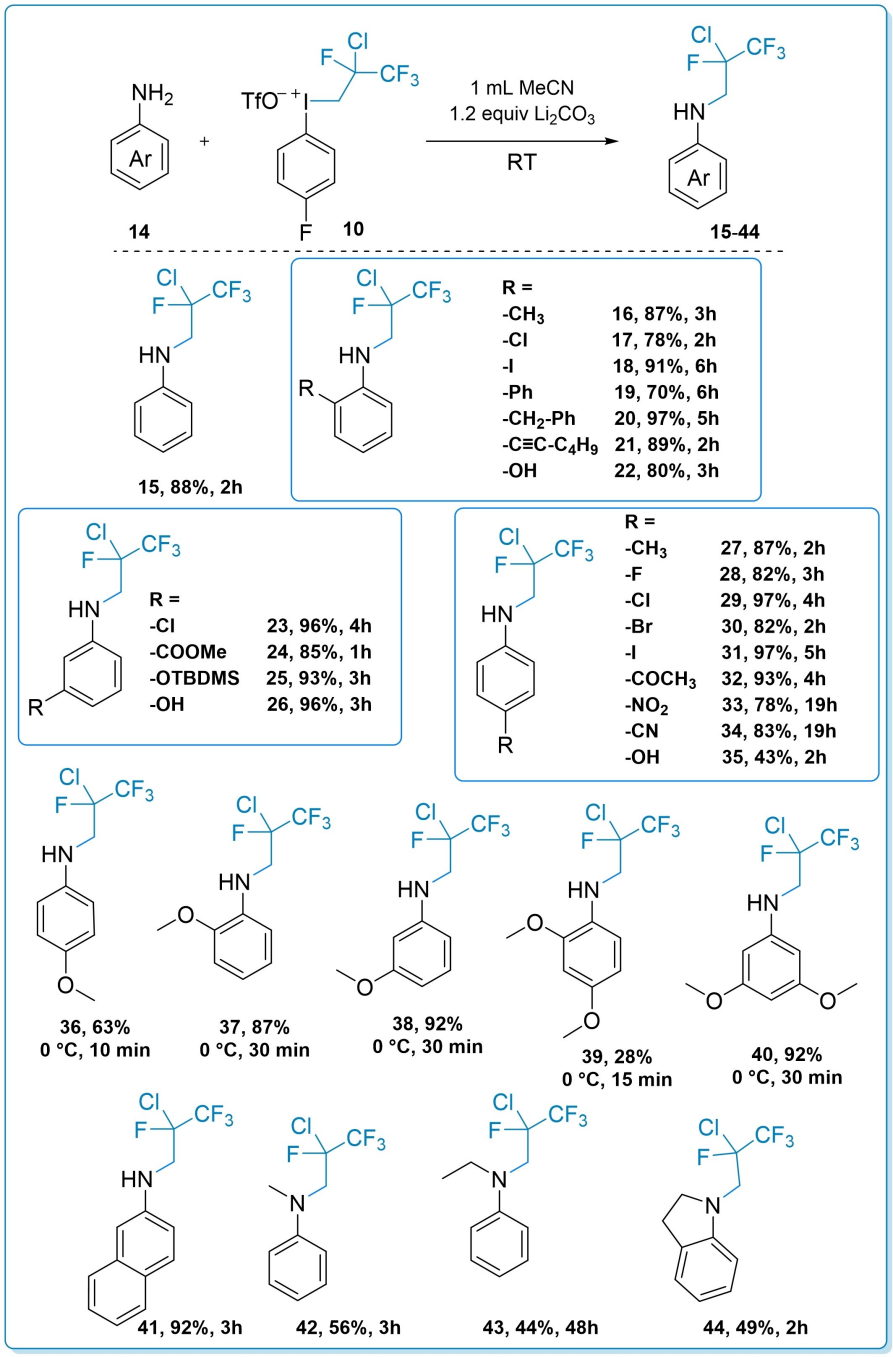
The scope of the substitution reaction with aniline derivatives. Reaction conditions: **14** (0.3 mmol, 1 equiv), **10** (0.33 mmol, 1.1 equiv), Li_2_CO_3_ (0.36 mmol, 1.2 equiv), MeCN (1 mL), RT or 0 °C.

We also examined the reactivity of *N*‐heterocycles (**45**), as shown in Scheme 4. In the first four examples (**46**–**49**), the substitution took place on the amino group and the expected products were obtained in good yields (67–94 %). Fluoroalkylation of 6‐aminoindazole under the established conditions led to the monosubstituted **49**, but utilizing 2.2 equivalent of iodonium salt afforded the corresponding disubstituted product (**50**) in 58 % yield, showing that the alkylation could also occur at the ring heteroatom. Accordingly, we functionalized various heterocycles such as tetrazole, benzotriazole, indazole and pyrazole at their NH position. In general, prolonged reaction time required to reach completion, and the yields of the products (**51**–**56**) are lower compared to the aniline derivatives. In the reaction of tetrazole **51**, benzotriazole **52** and pyrazole **54** we observed the presence of side products which formed in double substitution and elimination reactions. In the case of **52** we were able to isolate the minor components **52 x** and **52 z**, while **51 z** and **54 y** was not separated from its corresponding major product (**52** and **54**).^[15]^

**Scheme 4:**
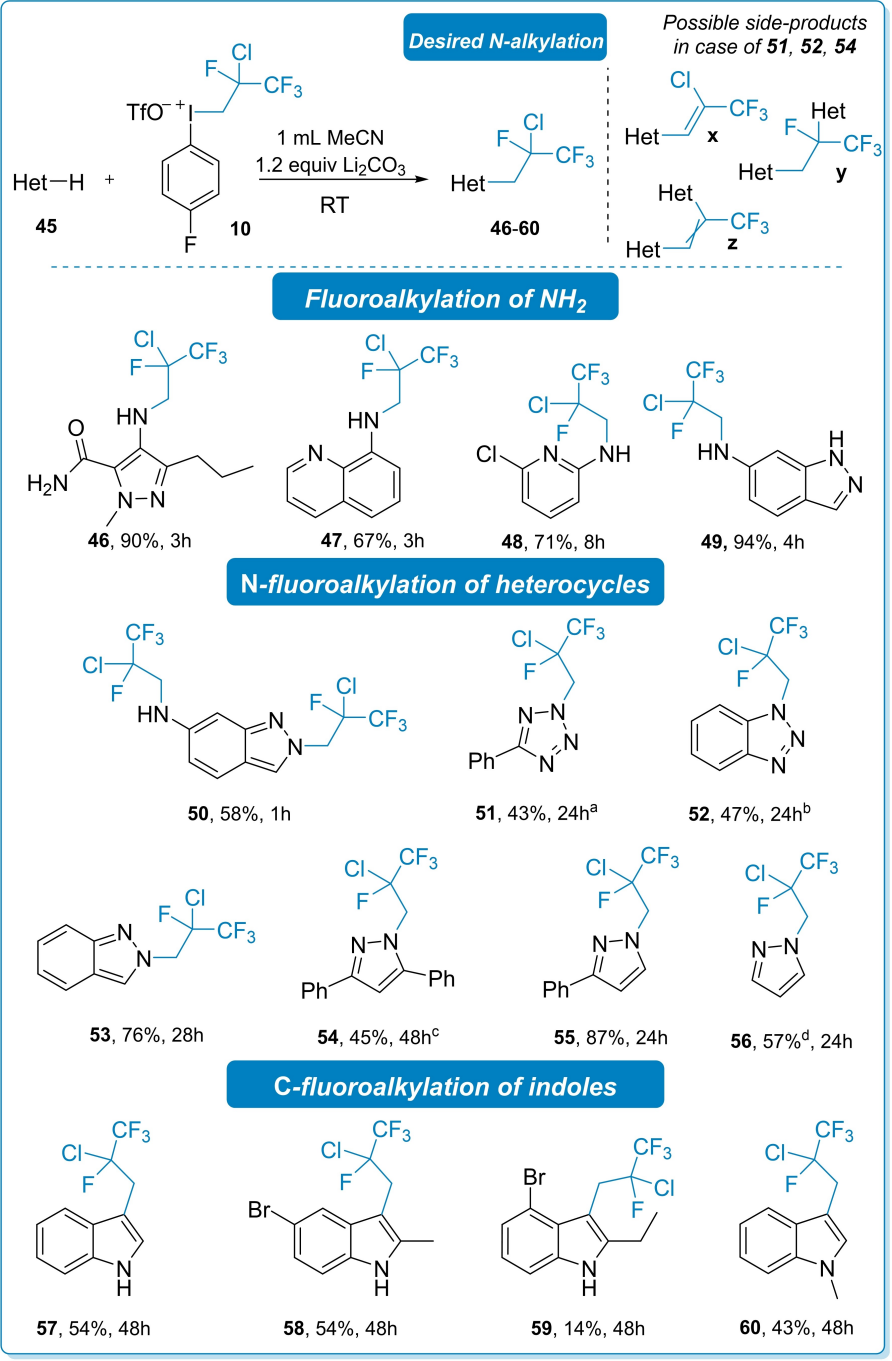
The scope of the substitution reaction with heteroaromatic compounds. Reaction conditions: **45** (0.3 mmol, 1 equiv), **10** (0.33 mmol, 1.1 equiv), Li_2_CO_3_ (0.36 mmol, 1.2 equiv), MeCN (1 mL) RT. [a] The product contains 30 % non‐separable side‐product **51 z**. [b] Side‐product **52 x** and **52 z** were also isolated as minor products. [c] The product contains 25 % non‐separable side‐product **54 y** as a result of double substitution. [d] The product was found to be volatile.

Finally, substitution of indoles took place at *C3* position with **10**, which is in line with the reactivities of indoles toward fluoroalkyliodonium salt,^[17]^ and the corresponding products (**57**–**60**) were isolated in 14–54 % yields.

In conclusion, we developed a novel organic transformation in which dyotropic rearrangement of iodine and chlorine function in alkyl chain enabled the construction of novel iodonium based fluoroalkylating reagent. The mechanism of the transformation was explored with DFT calculations, which justified the initial assumption of concerted [1,2]‐halogen shift. The reagent was synthesized on large scale using HFO‐1234yf fluorous feedstock. Moreover, we demonstrated that, the electrophilic reagent enables the direct metal‐free introduction of a special fluoroalkyl group to aniline derivatives and *N*‐containing heteroaromatic compounds in a substitution reaction under mild conditions, which provide unique fluorinated tags for various cyclic frameworks. The present synthetic concept can serve as a blueprint for exploring new strategies to develop iodonium based fluoroalkylating reagents.

## Conflict of interest

The authors declare no conflict of interest.

## Supporting information

As a service to our authors and readers, this journal provides supporting information supplied by the authors. Such materials are peer reviewed and may be re‐organized for online delivery, but are not copy‐edited or typeset. Technical support issues arising from supporting information (other than missing files) should be addressed to the authors.

Supporting Information

Supporting Information

## Data Availability

The data that support the findings of this study are available in the supplementary material of this article.
