# Supplementary material for: Synthesis of Hydrofluoroolefin‐Based Iodonium Reagent via Dyotropic Rearrangement and Its Utilization in Fluoroalkylation
Source: Angew Chem Int Ed Engl. 2022 Aug 9;61(37):e202208420. doi: 10.1002/anie.202208420 (PMC9540448; doi:10.1002/anie.202208420)

Angewandte Chemie
Eine Zeitschrift der Gesellschaft Deutscher Chemiker

Supporting Information

Synthesis of Hydrofluoroolefin-Based Iodonium Reagent via Dyotropic Rearrangement and Its Utilization in Fluoroalkylation

J. T. Csenki, B. L. Tóth, F. Béke, B. Varga, P. P. Fehér, A. Stirling, Z. Czégény, A. Bényei, Z. Novák**

Table of contents

1. General conditions 5

2. Optimization of the synthesis of iodonium salts 6

2.1 Optimization of the reaction temperature for the synthesis of iodonium salts 6

2.2 Optimization of the reaction time for the synthesis of iodonium salts 7

2.3 Other examination of the synthesis of iodonium salts 7

2.4 Examination of the aromatic compound for the synthesis of iodonium salts 8

2.5 Scalability of the synthesis of iodonium salts 8

3. Monitoring of iodonium salt synthesis by NMR 8

3.1 The first step of the two-step procedure at -10 °C, (-69)-(-83) ppm 10

3.2 The first step of the two-step procedure at -10 °C, (-100)-(-145) ppm 11

3.3 The second step of the two-step procedure using compound 4, (-69)-(-83) ppm 12

3.4 The second step of the two-step procedure using compound 4, (-69)-(-83) ppm 13

3.5 The first step of the two-step procedure at 35°C, (-69)-(-83) ppm 14

3.6 The first step of the two-step procedure at 35°C, (-100)-(-145) ppm 15

3.7 The first step of the two-step procedure at 35°C, (0)-(-20) ppm 16

3.8 The second step of the two-step procedure using compound 9, (-69)-(-83) ppm 17

3.9 The second step of the two-step procedure using compound 9, (-100)-(-145) ppm 18

3.10 Decomposition of the intermediate 4 at RT in acetonitrile, ^{19}F NMR: (-69)-(-83) ppm 19

3.11 Decomposition of the intermediate at RT in acetonitrile, ^{19}F NMR: (-100)-(-145) ppm 20

4. X-ray measurements of the iodonium salts 21

4.1. General information 21

4.2 Analysis of the structure 21

5. Pyrolysis-gas chromatography/mass spectrometry (Py-GC/MS) 27

6. Theoretical calculations for the rearrangement 28

6.1 General information 28

6.2 Calculations on the possible ionic and radical dyotropic pathways 30

6.3 Coordinates (.xyz format, in Å) and calculated Gibbs energies (including solvent and large basis electronic energy corrections) of the optimized structures 32

7. Optimization of the substitution reaction 37

7.1 Optimization of the reaction conditions: effect of bases 37

7.2 Optimization of the reaction conditions: effect of solvents 37

8. Synthesis of starting materials 38

2-(Hex-1-yn-1-yl)aniline 38

3-((Tert-butyldimethylsilyl)oxy)aniline 38

5-Phenyl-1*H*-tetrazole 38

3-Chloro-1,1,1,2-tetrafluoro-2-iodopropane (2) 39
9. Synthesis of iodonium salts 40
(2-Chloro-2,3,3,3-tetrafluoropropyl)(4-fluorophenyl)iodonium trifluoromethanesulfonate (10) ... 40
Bis(4-fluorophenyl)iodonium trifluoromethanesulfonate (6) 40
(2-Chloro-2,3,3,3-tetrafluoropropyl)(4-chlorophenyl)iodonium trifluoromethanesulfonate (10-Cl)40
(2-Chloro-2,3,3,3-tetrafluoropropyl)(phenyl)iodonium trifluoromethanesulfonate (10-H) 41
(2-Chloro-2,3,3,3-tetrafluoropropyl)(mesityl)iodonium trifluoromethanesulfonate (10-Mes) 41
10. Substitution on a larger scale in 1 mmol 41
11. Substitution reactions with iodonium salts 42
N-(2-Chloro-2,3,3,3-tetrafluoropropyl)aniline (15) 42
N-(2-Chloro-2,3,3,3-tetrafluoropropyl)-2-methylaniline (16) 43
2-Chloro-*N*-(2-chloro-2,3,3,3-tetrafluoropropyl)aniline (17) 43
N-(2-Chloro-2,3,3,3-tetrafluoropropyl)-2-iodoaniline (18) 43
N-(2-Chloro-2,3,3,3-tetrafluoropropyl)-[1,1'-biphenyl]-2-amine (19) 44
2-Benzyl-*N*-(2-chloro-2,3,3,3-tetrafluoropropyl)aniline (20) 44
N-(2-Chloro-2,3,3,3-tetrafluoropropyl)-2-(hex-1-yn-1-yl)aniline (21) 44
2-((2-Chloro-2,3,3,3-tetrafluoropropyl)amino)phenol (22) 45
3-Chloro-*N*-(2-chloro-2,3,3,3-tetrafluoropropyl)aniline (23) 45
Methyl 3-((2-Chloro-2,3,3,3-tetrafluoropropyl)amino)benzoate (24) 45
3-((Tert-butyldimethylsilyl)oxy)-*N*-(2-chloro-2,3,3,3-tetrafluoropropyl)aniline (25) 46
3-((2-Chloro-2,3,3,3-tetrafluoropropyl)amino)phenol (26) 46
N-(2-Chloro-2,3,3,3-tetrafluoropropyl)-4-methylaniline (27) 46
N-(2-Chloro-2,3,3,3-tetrafluoropropyl)-4-fluoroaniline (28) 47
4-Chloro-*N*-(2-chloro-2,3,3,3-tetrafluoropropyl)aniline (29) 47
4-Bromo-*N*-(2-chloro-2,3,3,3-tetrafluoropropyl)aniline (30) 47
N-(2-Chloro-2,3,3,3-tetrafluoropropyl)-4-iodoaniline (31) 48
1-(4-((2-Chloro-2,3,3,3-tetrafluoropropyl)amino)phenyl)ethan-1-one (32) 48
N-(2-Chloro-2,3,3,3-tetrafluoropropyl)-4-nitroaniline (33) 48
4-((2-Chloro-2,3,3,3-tetrafluoropropyl)amino)benzonitrile (34) 49
4-((2-Chloro-2,3,3,3-tetrafluoropropyl)amino)phenol (35) 49
N-(2-Chloro-2,3,3,3-tetrafluoropropyl)-4-methoxyaniline (36) 49
N-(2-Chloro-2,3,3,3-tetrafluoropropyl)-2-methoxyaniline (37) 49
N-(2-Chloro-2,3,3,3-tetrafluoropropyl)-3-methoxyaniline (38) 50
N-(2-Chloro-2,3,3,3-tetrafluoropropyl)-2,4-dimethoxyaniline (39) 50
N-(2-Chloro-2,3,3,3-tetrafluoropropyl)-3,5-dimethoxyaniline (40) 50
N-(2-Chloro-2,3,3,3-tetrafluoropropyl)naphthalen-2-amine (41) 51

N-(2-Chloro-2,3,3,3-tetrafluoropropyl)-*N*-methylaniline (42) 51
N-(2-Chloro-2,3,3,3-tetrafluoropropyl)-*N*-ethylaniline (43).. 51
1-(2-Chloro-2,3,3,3-tetrafluoropropyl)indoline (44) 52
4-((2-Chloro-2,3,3,3-tetrafluoropropyl)amino)-1-methyl-3-propyl-1*H*-pyrazole-5-carboxamide (46) 52
N-(2-Chloro-2,3,3,3-tetrafluoropropyl)quinolin-8-amine (47) 52
6-Chloro-*N*-(2-chloro-2,3,3,3-tetrafluoropropyl)pyridin-2-amine (48) 53
N-(2-Chloro-2,3,3,3-tetrafluoropropyl)-1*H*-indazol-6-amine (49).. 53
2-(2-Chloro-2,3,3,3-tetrafluoropropyl)-5-phenyl-2*H*-tetrazole (51 and 51z)................................... 54
1-(2-Chloro-2,3,3,3-tetrafluoropropyl)-1*H*-benzo[d][1,2,3]triazole (52) 54
1-(2-Chloro-3,3,3-trifluoroprop-1-en-1-yl)-1*H*-benzo[d][1,2,3]triazole (52x) 55
1-(2-(2*H*-benzo[d][1,2,3]triazol-2-yl)-3,3,3-trifluoroprop-1-en-1-yl)-1*H*-benzo[d][1,2,3]triazole (52z) 55
1-(2-Chloro-2,3,3,3-tetrafluoropropyl)-1*H*-indazole (53).. 55
1-(2-Chloro-2,3,3,3-tetrafluoropropyl)-3,5-diphenyl-1*H*-pyrazole (54 and 54y) 56
1-(2-Chloro-2,3,3,3-tetrafluoropropyl)-3-phenyl-1*H*-pyrazole (55) 56
1-(2-Chloro-2,3,3,3-tetrafluoropropyl)-1*H*-pyrazole (56) 56
3-(2-Chloro-2,3,3,3-tetrafluoropropyl)-1*H*-indole (57) 57
5-Bromo-3-(2-chloro-2,3,3,3-tetrafluoropropyl)-2-methyl-1*H*-indole (58) 57
4-Bromo-3-(2-chloro-2,3,3,3-tetrafluoropropyl)-2-ethyl-1*H*-indole (59)... 57
3-(2-Chloro-2,3,3,3-tetrafluoropropyl)-1-methyl-1*H*-indole (60) 58
12. Unsuccessful attempts.. 58
13. References 59
14. NMR spectra 59

1. General conditions

Unless otherwise indicated, starting materials were obtained from commercial suppliers, and were used without further purification. Analytical thin-layer chromatography (TLC) was performed on Merck DC pre-coated TLC plates with 0.25 mm Kieselgel 60 F_{254}. Visualization was performed with a 254 nm UV lamp and $KMnO_4$ stain.

All melting points were measured on Büchi 501 apparatus.

The ^{1}H, ^{13}C and ^{19}F NMR spectra were recorded on a Bruker Avance-250, Bruker Avance-400 and Bruker Avance-500 MHz spectrometer in $CDCl_3$, CD_3CN, DMSO-d6. Chemical shifts are expressed in parts per million (δ) using residual solvent protons as internal standards ($CDCl_3$: δ 7.26 for ^{1}H, δ 77.16 for ^{13}C, CD_3CN: δ 1.94 for ^{1}H, δ 1.32 for ^{13}C, DMSO-d6: δ 2.50 for ^{1}H, δ 39.52 for ^{13}C). Coupling constants (J) are reported in Hertz (Hz). Splitting patterns are designated as s (singlet), bs (broad singlet), d (doublet), t (triplet), q (quartet), dq (doublet quartet), qd (quartet doublet), m (multiplet).

Conversions determined by gas chromatography. This, and low-resolution mass spectrometry was obtained on an Agilent 6890N Gas Chromatograph (30 m x 0.25 mm column with 0.25 µm HP-5MS coating, He carrier gas) and Agilent 5973 Mass Spectrometer (Ion source: EI+, 70eV, 230°C interface 300°C). GC-MS conversion was calculated from the chromatogram using the integral of the peak belonging to the starting material (ISM) and the integral of the peak belonging to the product (IP):

$$conversion(\%) = \frac{IP}{IP + ISM} \cdot 100$$

IR spectra were obtained on a Mettler Toledo ReactIR™ 15, AgX DiComp probe, 6 mm x 1.5 m Fiber (Silver Halide), MCT detector.

High-resolution mass spectra were acquired on an Agilent 6230 time-of-flight mass spectrometer equipped with a Jet Stream electrospray ion source in positive ion mode. Injections of 0.1-0.3 µl were directed to the mass spectrometer at a flow rate 0.5 ml/min (70% acetonitrile-water mixture, 0.1 % formic acid), using an Agilent 1260 Infinity HPLC system. Jet Stream parameters: drying gas (N_2) flow and temperature: 10.0 l/min and 325 °C, respectively; nebulizer gas (N_2) pressure: 10 psi; capillary voltage: 4000V; sheath gas flow and temperature: 325°C and 7.5 l/min; TOFMS parameters: fragmentor voltage: 120 V; skimmer potential: 120 V; OCT 1 RF Vpp:750 V. Full-scan mass spectra were acquired over the m/z range 100-2500 at an acquisition rate of 250 ms/spectrum and processed by Agilent MassHunter B.03.01 software.

2. Optimization of the synthesis of iodonium salts

A screwed cap vials with a stirrer bar was evacuated and refilled with argon three times. Trifluoroacetic anhydride (15.122 g, 10.0 mL, 72 mmol) and catalytic amount of trifluoroacetic acid (0.144 g, 0.1 mL, 1 mmol) was added through syringe. The mixtures were cooled to -10 °C, then hydrogen-peroxide (50 w/w% in water) (1.156 g, 1.0 mL, 17 mmol) was added dropwise within two minutes. 3-Chloro-1,1,1,2-tetrafluoro-2-iodopropane (2.764 g, 1.3 mL, 10 mmol) was added dropwise through syringe. The resulting reaction mixtures were stirred at the given temperature (T_1) for the given time (t_1). The reaction mixture was cooled to the given temperature (T_2), then freshly distilled dichloromethane was added to the mixtures. Fluorobenzene was added dropwise to the reaction mixtures, followed by the addition of trifluoromethanesulfonic acid (1.501 g, 0.9 mL, 10 mmol). The reaction mixtures were kept between 0 °C and 4 °C. After that, all volatiles were removed under reduced pressure at 0 °C protected from light. The dark oil was shaken with cold (-20 °C) diethyl ether, getting white precipitate. The suspension was kept at -20 °C for 2 hours, then the white precipitate was filtered and washed with cold diethyl ether three times. (The diaryl-iodonium side product (**6**) can be removed by washing with DCM.)

2.1 Optimization of the reaction temperature for the synthesis of iodonium salts

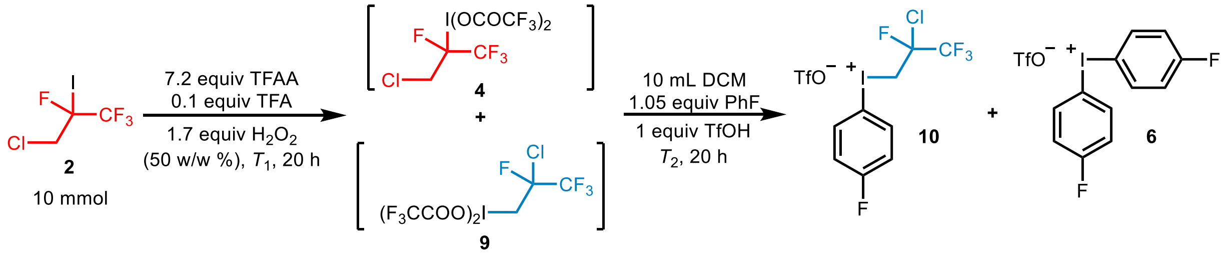

Entry	T_1 / °C	T_2 / °C	$m_{mixture}$ / g	Ratio (**10**:**6**) [a]	Yield [b]	m_{10} / g	$Yield_{10}$
1	-10	0	1.9897	05:95	2%	-	-
2	-10 -> 0	0	2.5548	28:72	15%	-	-
3	-10 -> RT	0	3.2068	55:45	36%	1.660	31%
**4**	**-10 -> 35**	**0**	**2.9403**	**75:25**	**43%**	**2.201**	**42%**
5	-10 -> 45	0	2.0100	92:08	36%	1.719	32%
6	-10 -> 55	0	1.7098	87:13	29%	1.396	26%
7	-10 -> RT	-20 -> 0	3.2138	70:30	45%	-	-
8	-10 -> RT	20	1.6875	78:22	26%	-	-

[a] Calculation based on NMR measurements. [b] Yields calculated on the basis of the NMR spectra of isolated mixture

An optimum temperature was found for the first step which is 35 °C. In the second step, the recommended temperature is -20 °C -> 0 °C.

2.2 Optimization of the reaction time for the synthesis of iodonium salts

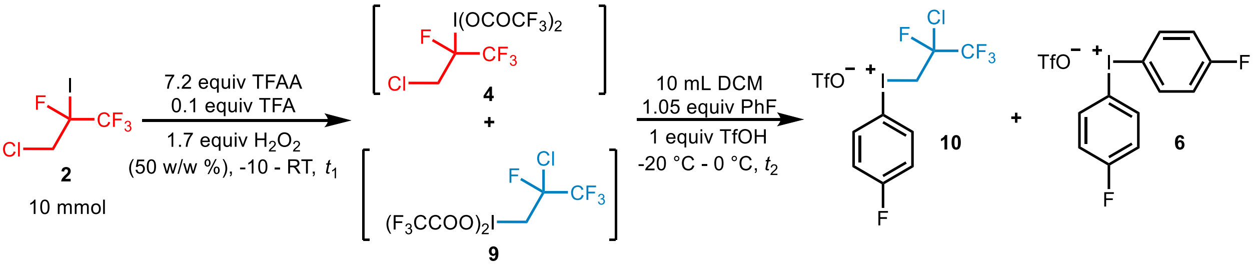

Entry	t_1 / h	t_2 / h	$m_{mixture}$ / g	Ratio (**10**:**6**) [a]	Yield [b]
1	20	20	3.2068	55:45	36%
2	20	6	3.1532	56:44	36%
3	2	20	2.2930	28:72	13%
4	48	20	3.0937	42:58	27%

[a] Calculation based on NMR measurements. [b] Yields calculated on the basis of the NMR spectra of isolated mixture

2.3 Other examination of the synthesis of iodonium salts

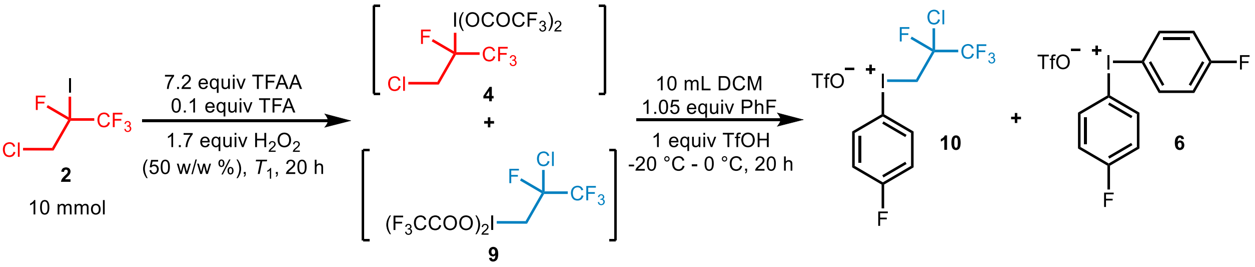

Entry	T_1 / °C	Comment	$m_{mixture}$ / g	Ratio (**10**:**6**) [a]	Yield
1	-10 -> RT	1.55 equiv PhF	3.0687	57:43	35%[b]
2	-10 -> RT	1.05 equiv PhF	3.2068	55:45	36%[b]
**3**	**-10 -> 35**	**1.1 equiv H_2O_2 instead of 1.7 equiv**	**2.1365**	**100:00**	**41%**

[a] Calculation based on NMR measurements. [b] Yields calculated on the basis of the NMR spectra of isolated mixture

Increasing the amount of fluorobenzene from 1.05 equiv to 1.55 equiv had no effect on the reaction. Decreasing the amount of hydrogen-peroxide resulted better ratio (100% pure product) but it had no effect on the final yield.

2.4 Examination of the aromatic compound for the synthesis of iodonium salts

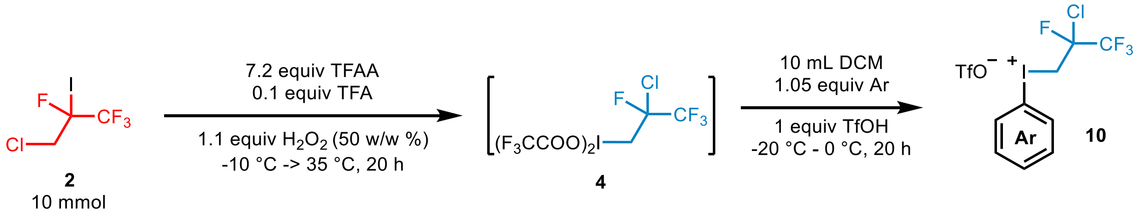

Entry	Ar	*m* / g	Yield
1	PhH	1.795	36%
**2**	**PhF**	**2.137**	**41%**
3	PhCl	1.935	36%
4	Mesitylene	1.368	25%

2.5 Scalability of the synthesis of iodonium salts

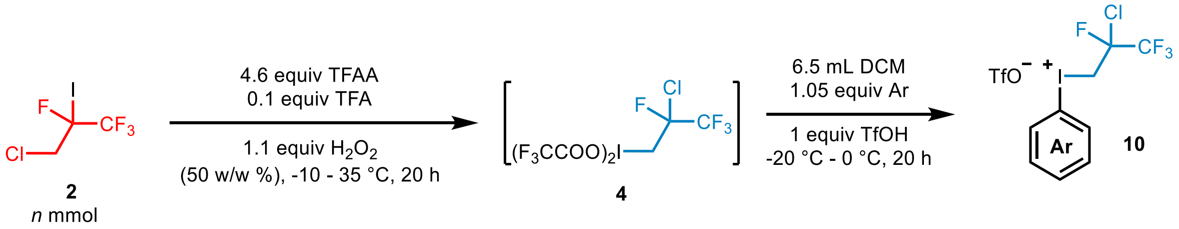

Entry	*n* / mmol	*m* / g	Yield
1	10	2.201	41%
2	20	3.806	37%
3	30	5.754	37%
4	50	12.317	47%

The amount of the TFAA was recalculated because of the amount of water in the less amount of H_2O_2.

3. Monitoring of iodonium salt synthesis by NMR

The iodonium salt synthesis was monitored by NMR (Nanalysis NMRReady 60PRO) in four separate experiments. Fresh sample was taken from the reaction mixture in all cases. The sample was dissolved in cold acetonitrile-d3 (-30°C) and measured as soon as possible (until it did not warm up, otherwise refrigerate the sample below -10 °C) because some intermediate could decompose at higher temperature (see **Chapter 3.10** and **3.11**).

The four separate experiments:

1, The first step of the two-step procedure at -10 °C. (**Chapter 3.1 and 3.2**)

2, The second step of the two-step procedure. (**Chapter 3.3 and 3.4**) (The intermediate was freshly prepared at -10 °C without sampling it.)

3, The first step of the two-step procedure at 35°C. (**Chapter 3.5, 3.6 and 3.7**)

4, The second step of the two-step procedure. (**Chapter 3.8 and 3.9**) (The intermediate was freshly prepared at 35 °C without sampling it.)

NMR spectra are discussed in two (one case three) magnified part for greater clarity.

General procedure of the two-step synthesis:

First step: Screwed cap vials with a stirrer bar was evacuated and refilled with argon three times. Trifluoroacetic anhydride (15.122 g, 10.0 mL, 72 mmol) and catalytic amount of trifluoroacetic acid (0.144 g, 0.1 mL, 1 mmol) was added through syringe. The mixtures were cooled to -10 °C, then hydrogen-peroxide (50 w/w% in water) (1.156 g, 1.0 mL, 17 mmol) was added dropwise within two minutes. 3-Chloro-1,1,1,2-tetrafluoro-2-iodopropane (2.764 g, 1.3 mL, 10 mmol) was added dropwise through syringe. The resulting reaction mixtures were stirred at *-10°C* or *35 °C* and evacuated the volatile compounds under reduced pressure at -10°C.

Second step: Using newly prepared intermediates, the reaction mixtures was cooled to -20 °C, then freshly distilled dichloromethane was added to the mixtures. 15 mmol fluorobenzene was added dropwise to the reaction mixtures, followed by the addition of trifluoromethanesulfonic acid (1.501 g, 0.9 mL, 10 mmol). The reaction mixtures were kept between 0 °C and 4 °C. After that, all volatiles were removed under reduced pressure at 0 °C protected from light. The dark oil was shaken with cold (-20 °C) diethyl ether, getting white precipitate. The suspension was kept at -20 °C for 2 hours, then the white precipitate was filtered and washed with cold diethyl ether three times.

Conclusions:

1, The expected iodonium-bis-trifluoroacetate compound (**4**) was prepared at -10 °C without rearrangement. This product contains the other isomer of the bis trifluoroacetate (**9**) in some amount because the starting material contains the other isomer about 5%. Air and temperature sensitive. This intermediate decomposes (mostly to 1-chloro-2,3,3,3-tetrafluoroprop-1-ene, **5**) in a few minutes when the sample or the reaction mixture warms up to room temperature in acetonitrile (**Chapter 3.10** and **3.11**).

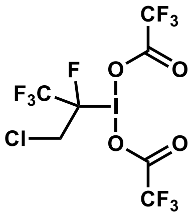

4

2, Using compound **4**. A decomosition reaction occurs momentary after addition of trifluoromethanesulfonic acid. 1-chloro-2,3,3,3-tetrafluoroprop-1-ene (**5**), 3-chloro-1,1,1,2-tetrafluoro-2-iodopropane (**2**) (the starting material) and bis(4-fluorophenyl) iodonium compounds (**6**) produced.

3, The compound **9** was prepared at 35°C while the starting material rearranged. The formation of **4** as an intermediate was observed while 1-chloro-2,3,3,3-tetrafluoroprop-1-ene gas produced (**5**) (most of the gas bubbled from the reaction mixture). CF_3I also formed (**Chapter 3.7**). Spike the sample with 50 µl 35% CF_3I in DMSO gave the same signal.

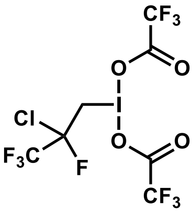

9

4, Using compound **9**. The reaction occurs momentary after addition of trifluoromethanesulfonic acid. The product is (2-Chloro-2,3,3,3-tetrafluoropropyl)(4-fluorophenyl)iodonium trifluoromethane-sulfonate (**10**) without any side product.

3.1 The first step of the two-step procedure at -10 °C, (-69)-(-83) ppm

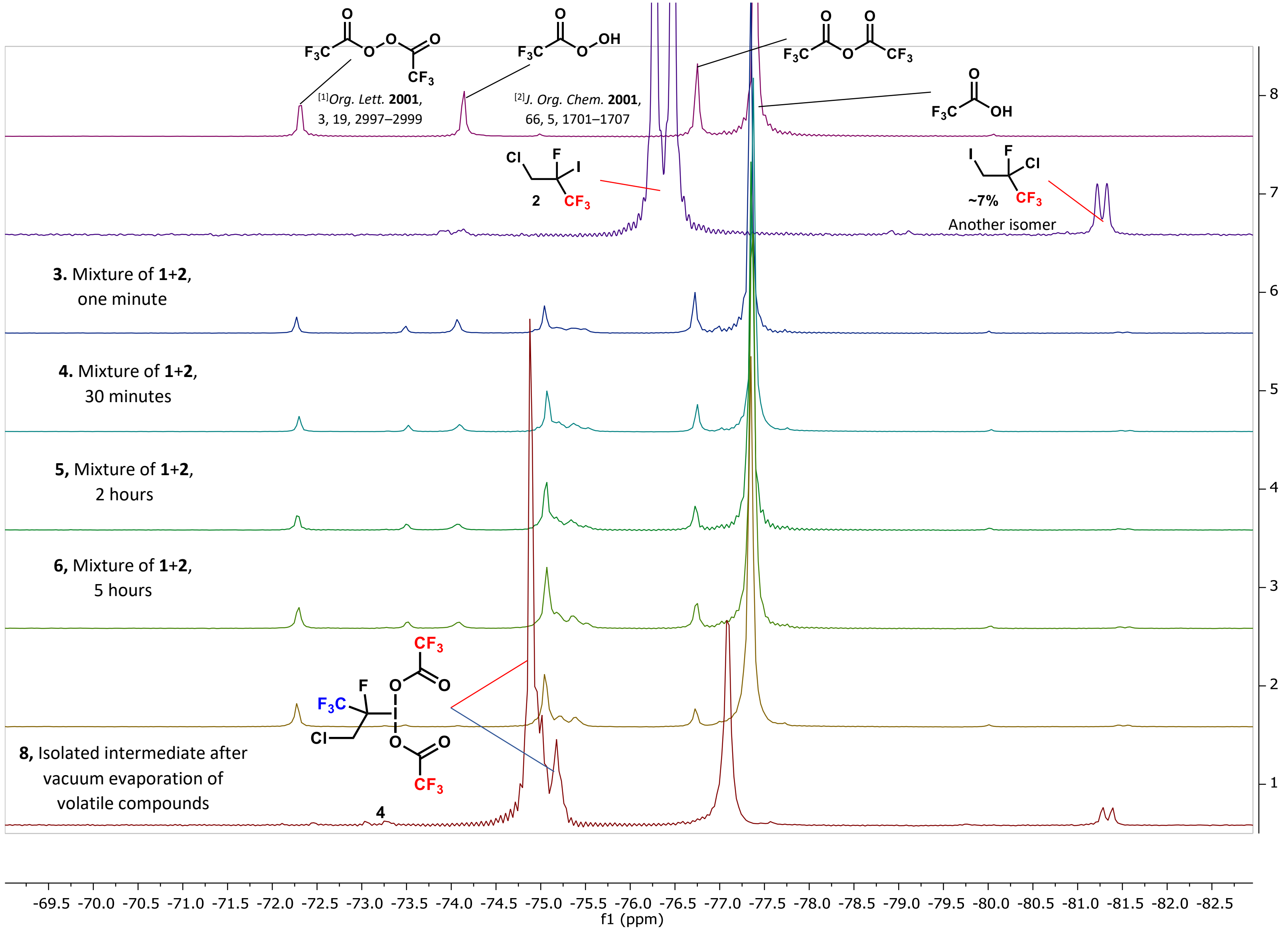

3.2 The first step of the two-step procedure at -10 °C, (-100)-(-145) ppm

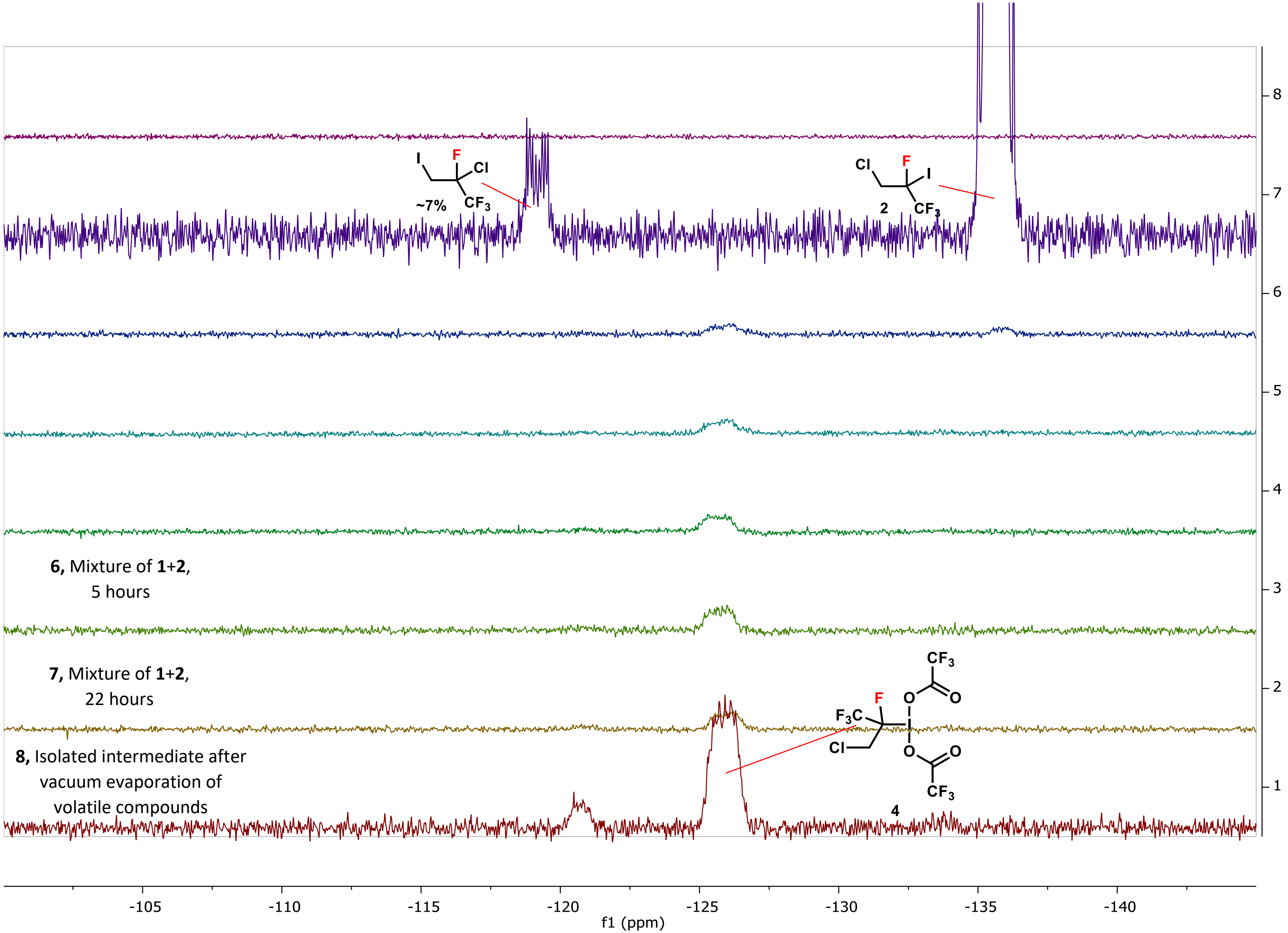

3.3 The second step of the two-step procedure using compound 4, (-69)-(-83) ppm

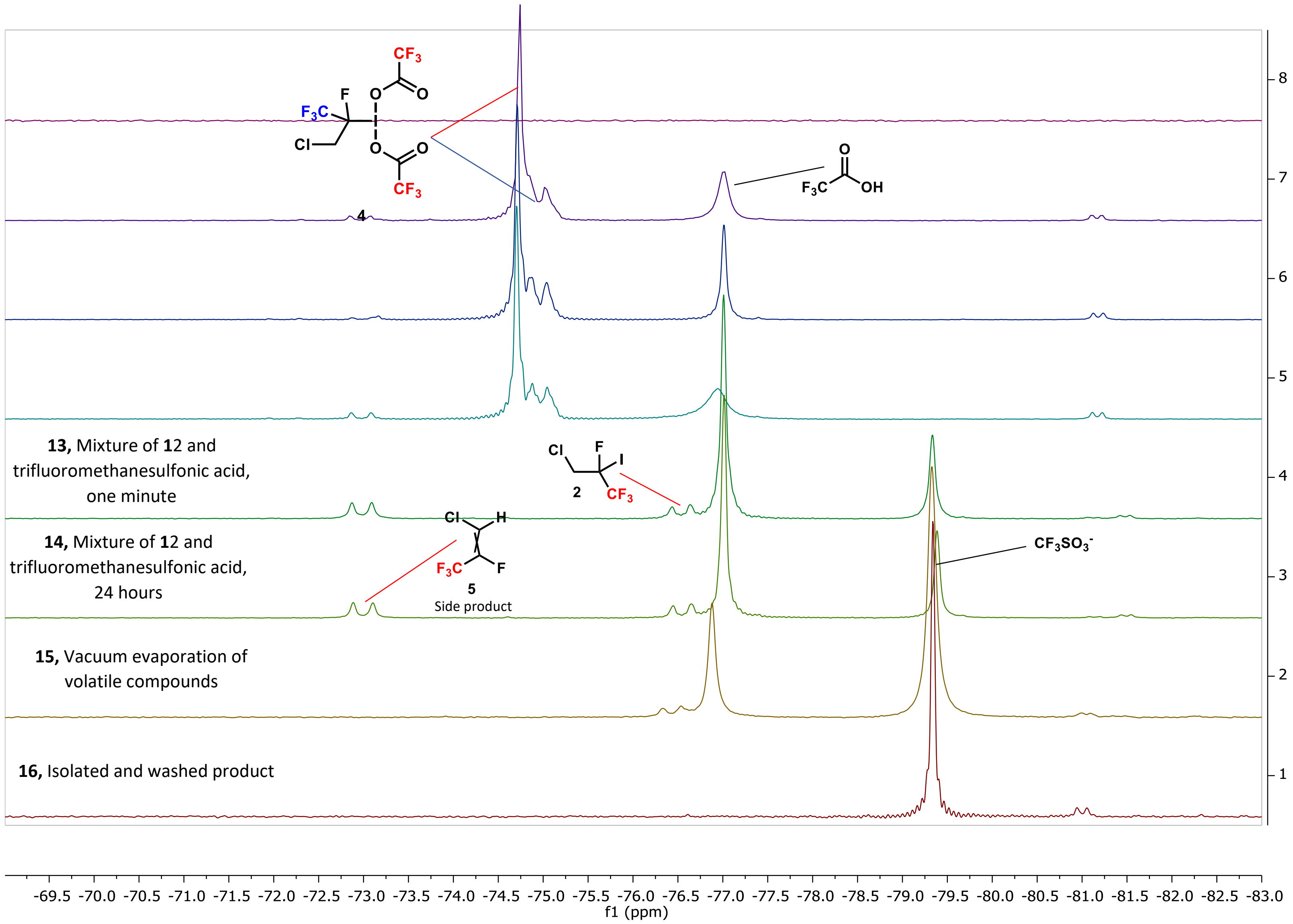

3.4 The second step of the two-step procedure using compound 4, (-69)-(-83) ppm

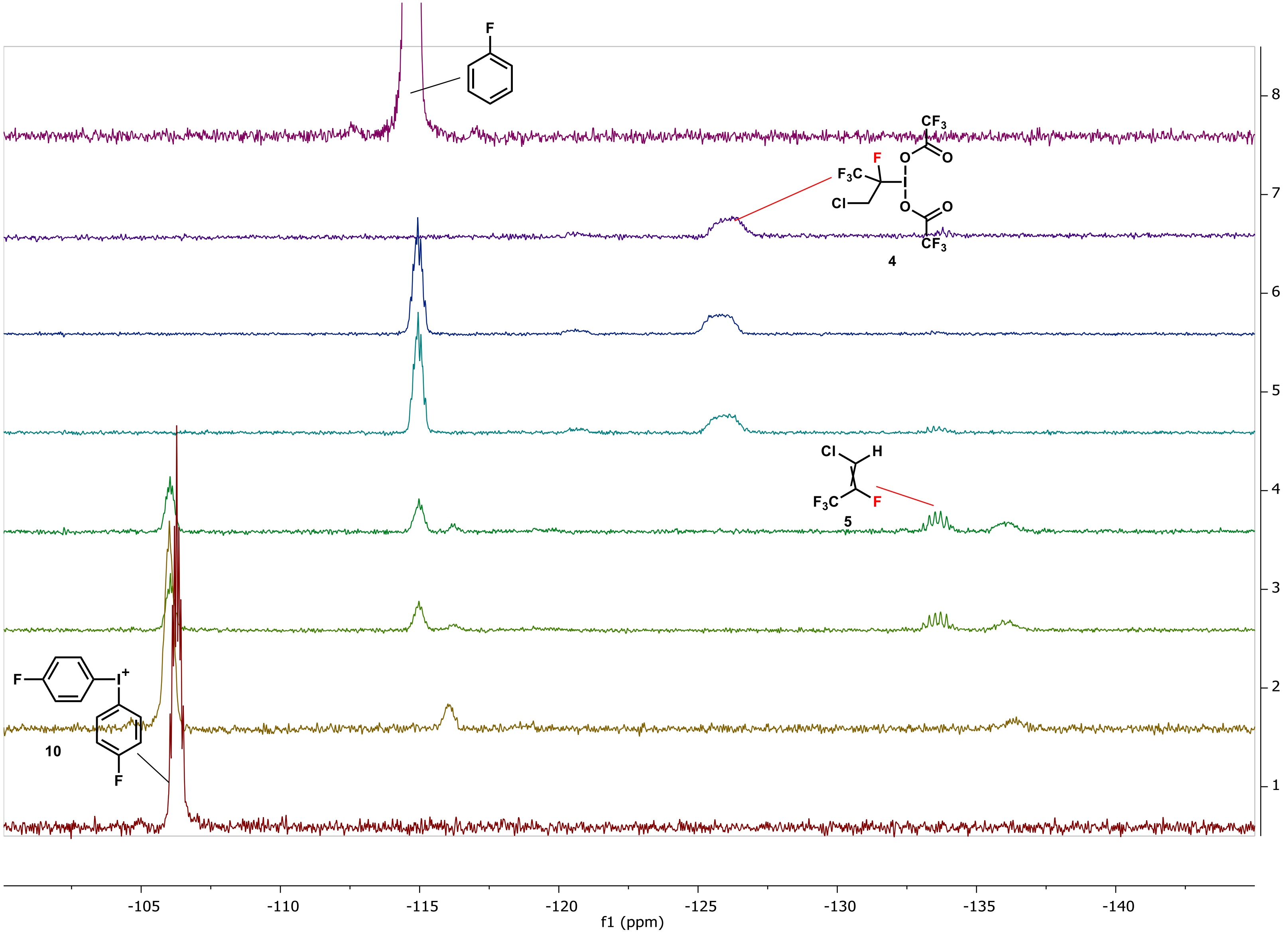

3.5 The first step of the two-step procedure at 35°C, (-69)-(-83) ppm

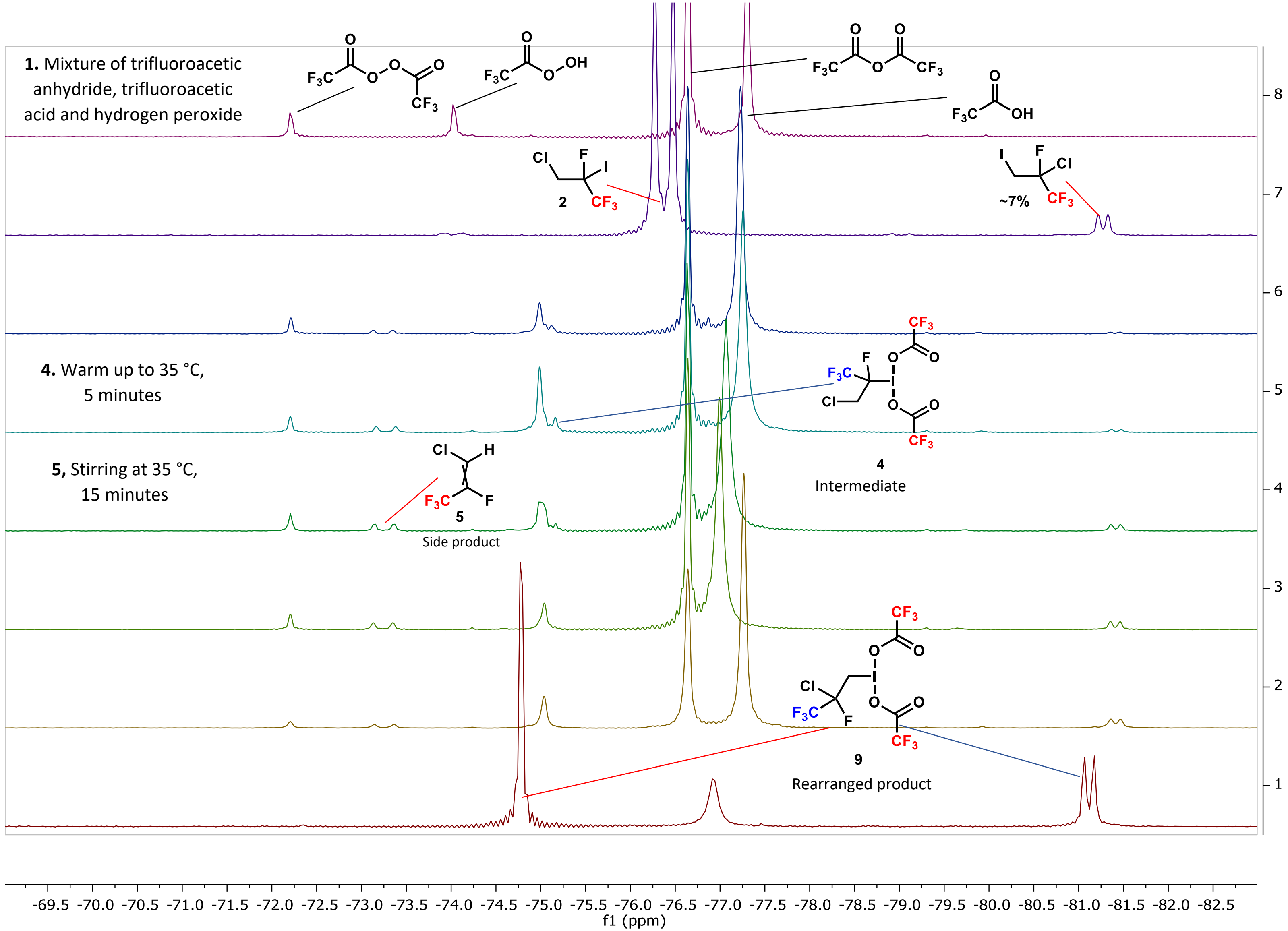

3.6 The first step of the two-step procedure at 35°C, (-100)-(-145) ppm

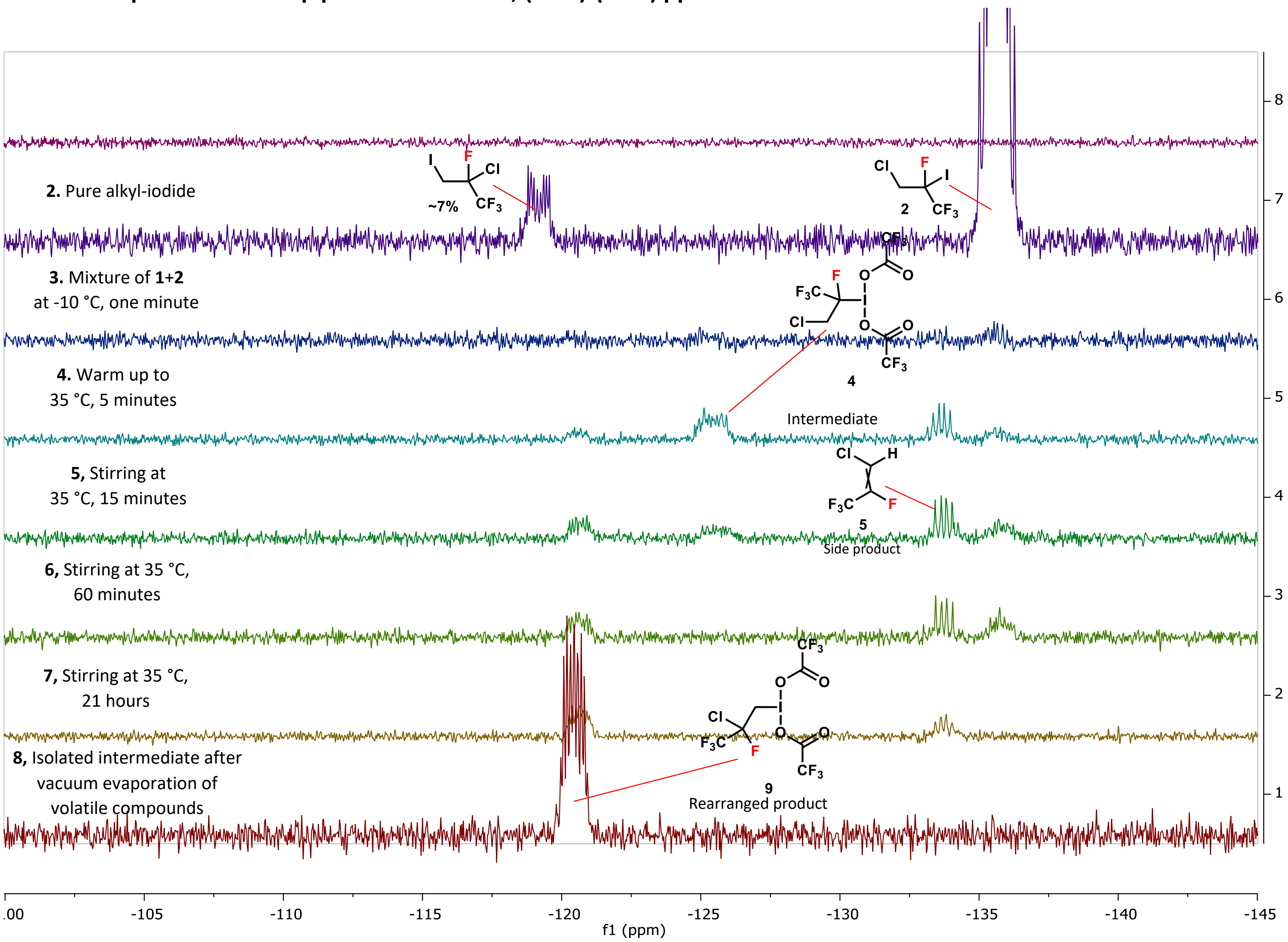

3.7 The first step of the two-step procedure at 35°C, (0)-(-20) ppm

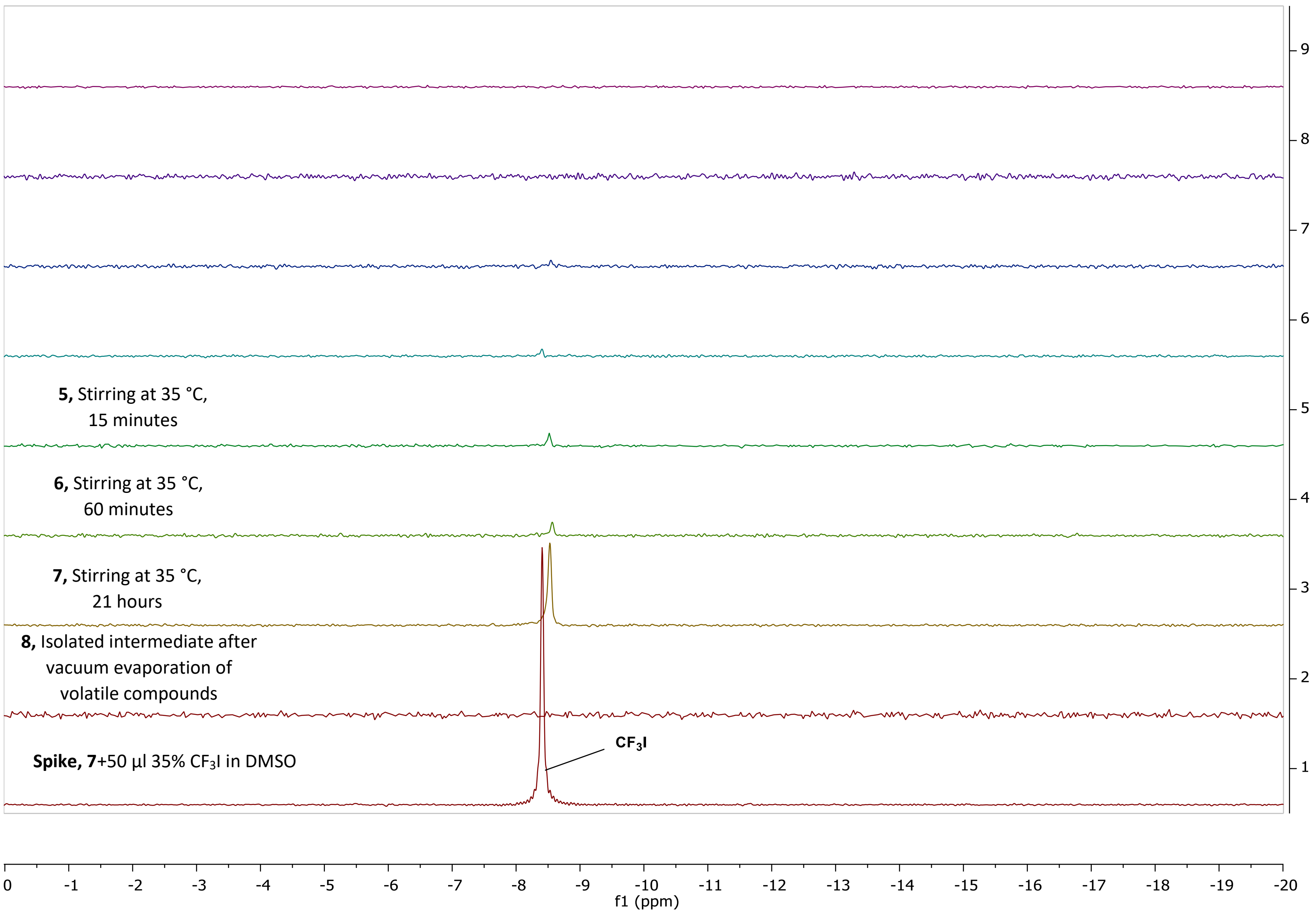

3.8 The second step of the two-step procedure using compound 9, (-69)-(-83) ppm

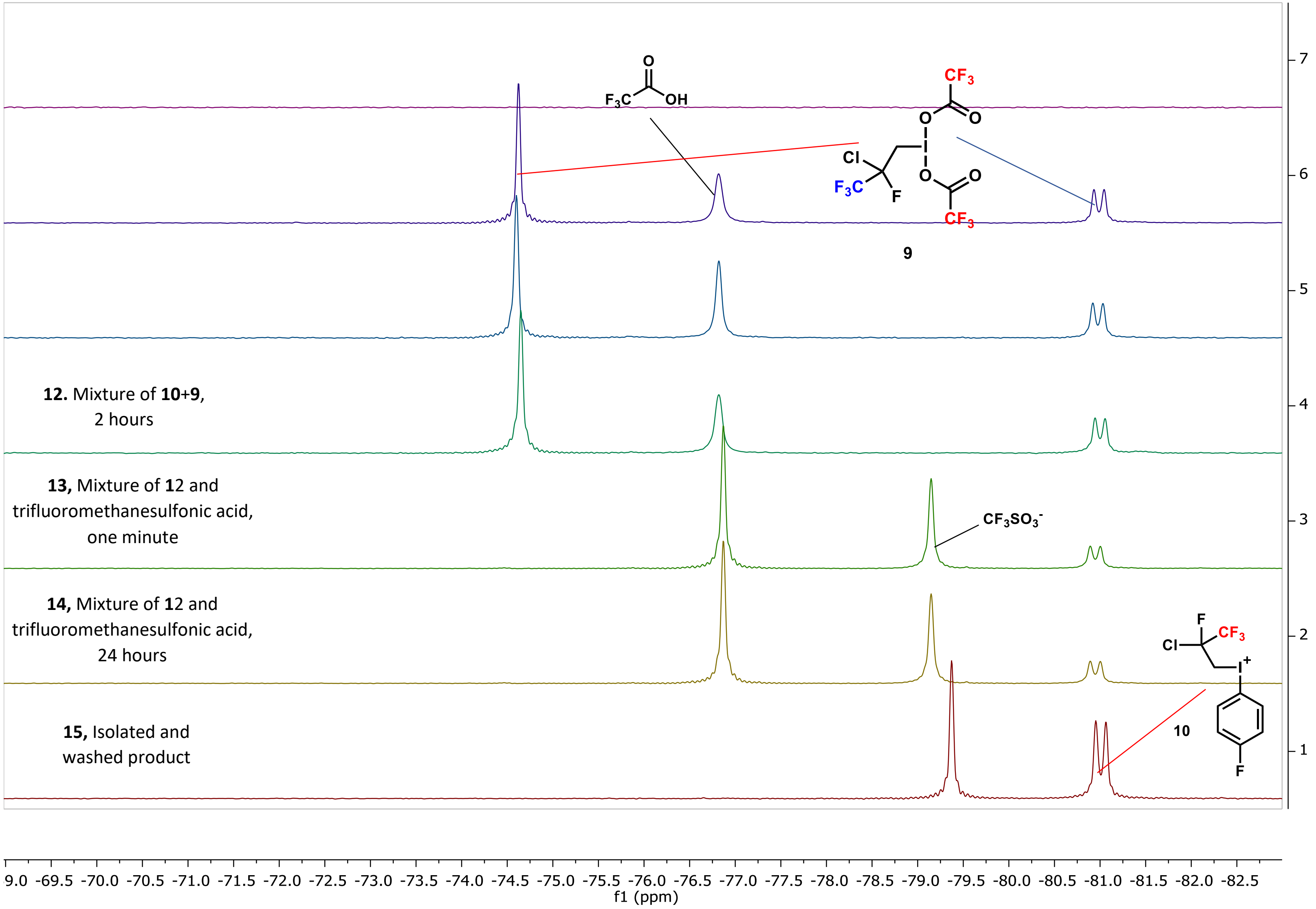

3.9 The second step of the two-step procedure using compound 9, (-100)-(-145) ppm

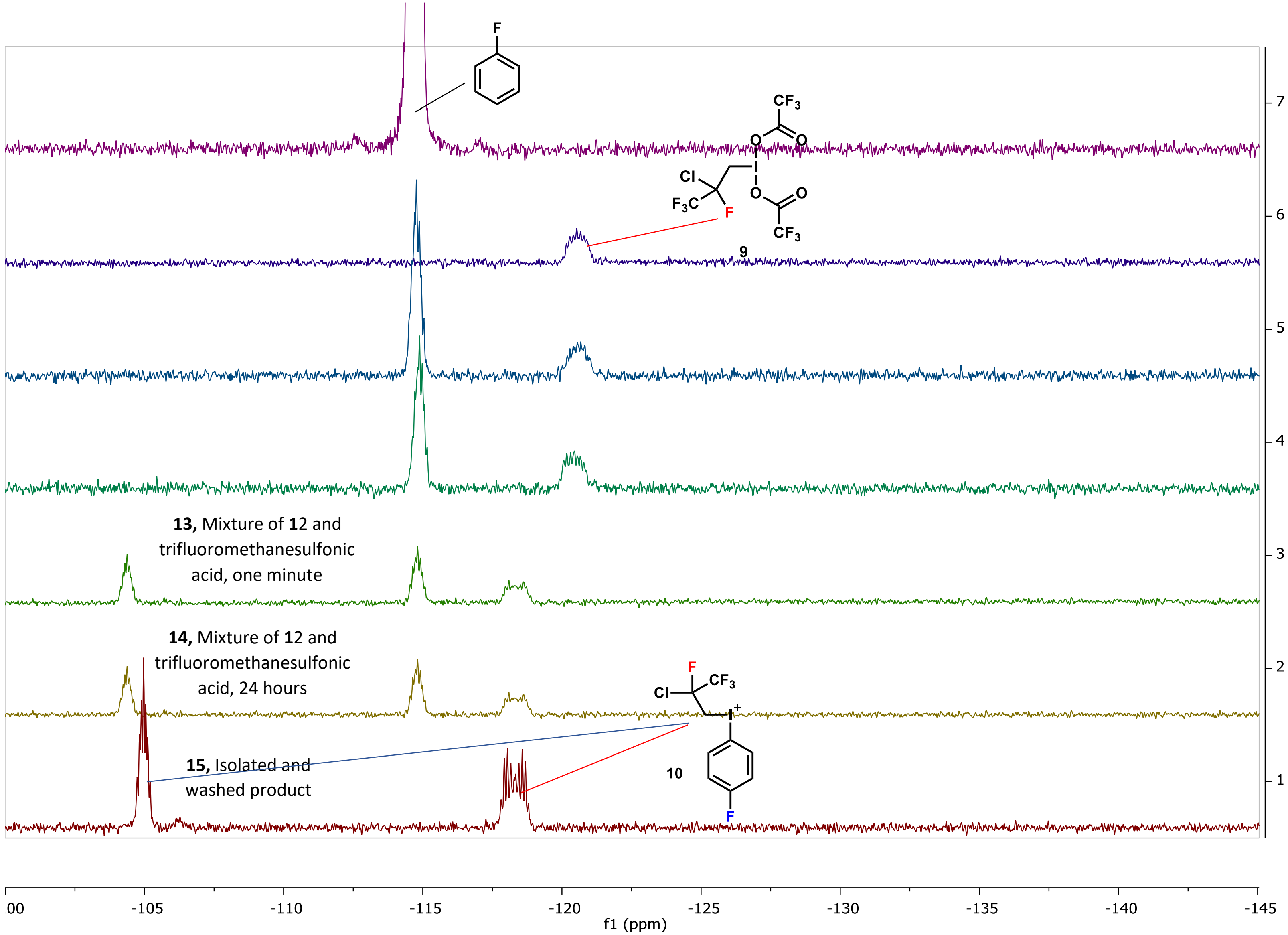

3.10 Decomposition of the intermediate 4 at RT in acetonitrile, ^{19}F NMR: (-69)-(-83) ppm

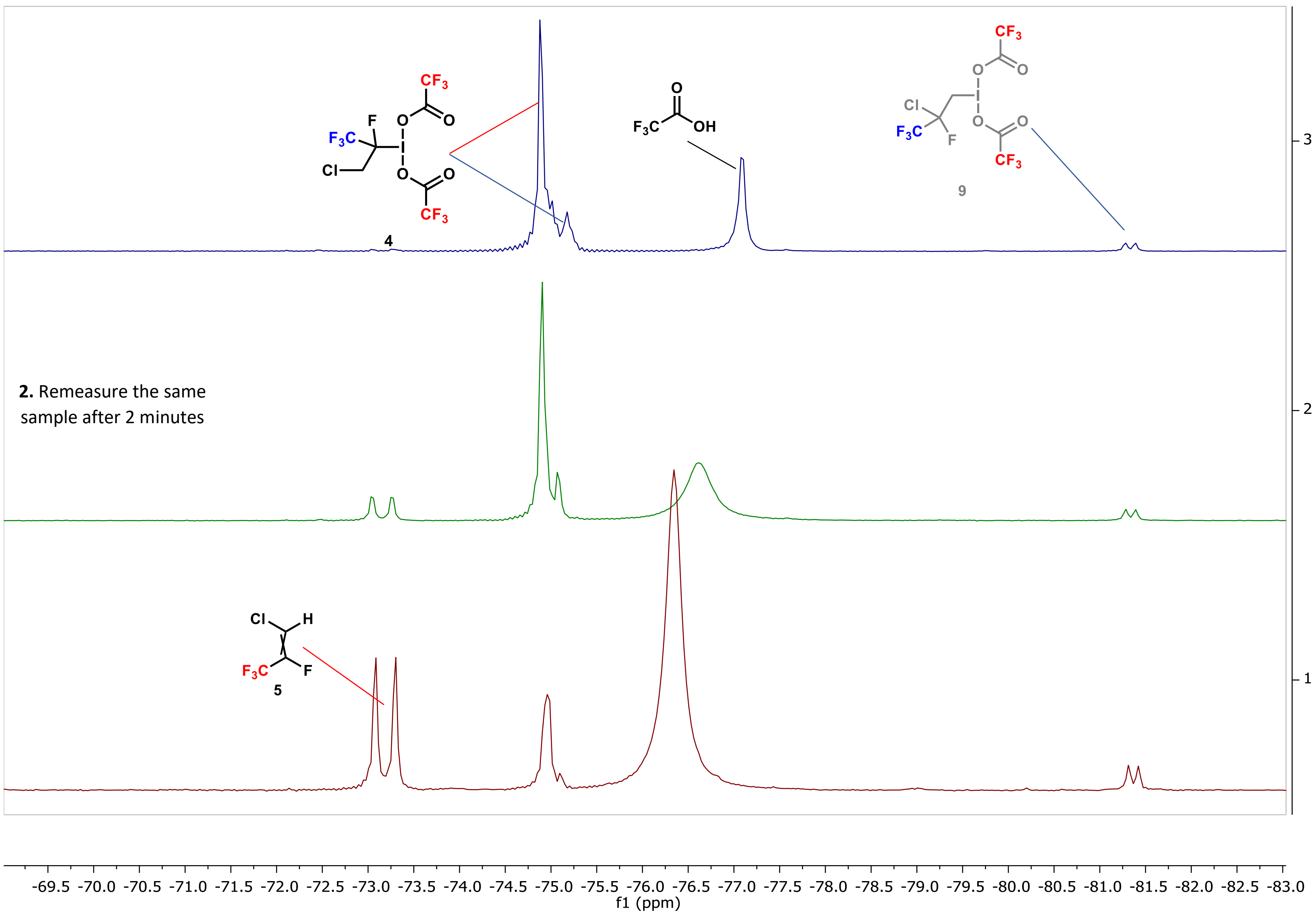

3.11 Decomposition of the intermediate at RT in acetonitrile, ^{19}F NMR: (-100)-(-145) ppm

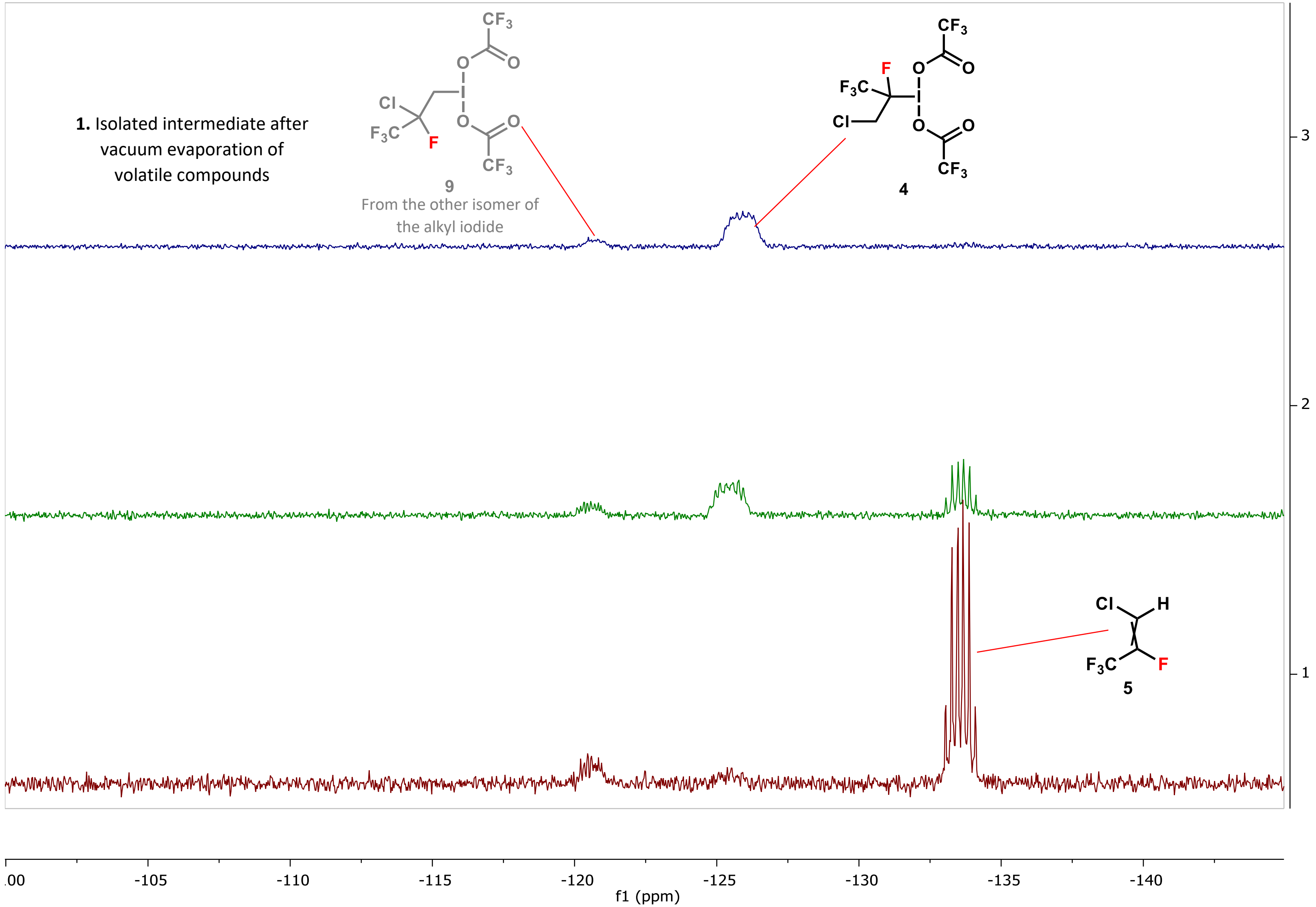

4. X-ray measurements of the iodonium salts

4.1. General information

X-ray-quality crystals of **10** were grown by slow evaporation of its acetonitrile solution at 0°C. A crystal well-looking in polarized light microscope was fixed under a microscope onto a Mitegen loop using high-density oil. Diffraction intensity data were collected at room temperature (291 K) as several full datasets of different crystals using a Bruker-D8 Venture diffractometer (Bruker AXS GmbH, Karlsruhe, Germany) equipped with INCOATEC IµS 3.0 (Incoatec GmbH, Geesthacht, Germany) dual (Cu and Mo) sealed tube micro sources and a Photon II Charge-Integrating Pixel Array detector (Bruker AXS GmbH, Karlsruhe, Germany) using Mo Kα (λ = 0.71073 Å) radiation. High multiplicity data collection and integration were performed using APEX4 (version 2021-4.0, Bruker AXS Inc., 2021, Madison, USA) software. Data reduction and multi-scan absorption correction were performed using SAINT (version 8.40B, Bruker AXS Inc., 2019, Madison, USA). The structure was solved using direct methods and refined on F^2 using the SHELXL program[3] incorporated into the APEX4 suite. Refinement was performed anisotropically for all non-hydrogen atoms. Hydrogen atoms were placed into geometric positions. Numerical absorption correction had to be applied because of the presence of heavy atoms and shape of the crystal. Further experimental details are shown at Table SI4.1. The CIF file was manually edited using Publcif software,[4] while graphics were prepared using the Mercury program.[5] The results for the X-ray diffraction structure determinations were very good according to the Checkcif functionality of PLATON software (Utrecht University, Utrecht, The Netherlands),[6] and structural parameters such as bond length and angle data were in the expected range and shown in Table SI4.2. Slight disorder of one of the trifluoro-methyl groups resulted in B level errors by a few irregular atomic displacement parameters but the overall correctness of the structure is not influenced. The supplementary crystallographic data for **10** can be obtained free of charge from the Cambridge Crystallographic Data Centre via http://www.ccdc.cam.ac.uk/data_request/cif, using reference deposition number 2176329.

4.2 Analysis of the structure

The single crystal x-ray diffraction study had unambiguously proved the chemical structure of **10** (Figure SI4.1). However, the crystal structure has interesting features. The compound crystallized in triclinic crystal system in chiral space group (P1, No. 1) with two molecules in the asymmetric unit. Moreover, the structure is well refined as racemic twin and the ratio of the two domain is approximately 55:45 as C2 and C52 are stereogenic centers. Also, one of the molecule has a disorder at the CF_3 group with occupancy ratio of 66:37. There is a slight difference in the conformation of the two cations in the asymmetric unit as it is shown at Figure SI4.2 with rotation of the phenyl ring around the C11-C14 axis, for the disordered molecule only the major component is shown. The packing diagram (Figure SI4.3) shows the layered structure stabilized with strong electrostatic interactions and C-H..O as well as C-H..F hydrogen bonds (Table SI4.3).

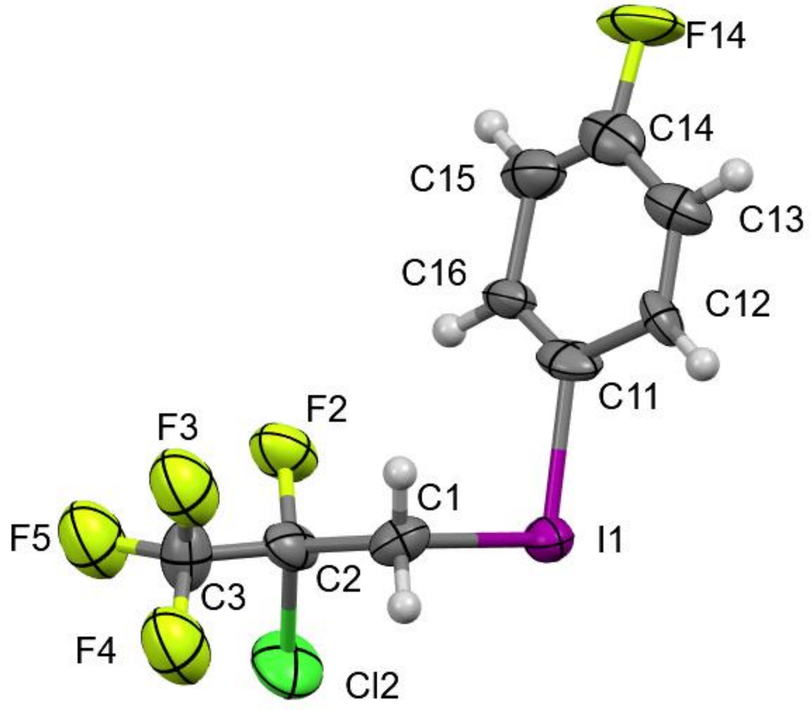

Figure SI4.1 *ORTEP view of **10** at 50% probability level with numbering scheme. Only one molecule from the asymmetric unit is shown, triflate counter ion is omitted for clarity. The numbering scheme for the other molecule is n+50, C1 corresponds C51.*

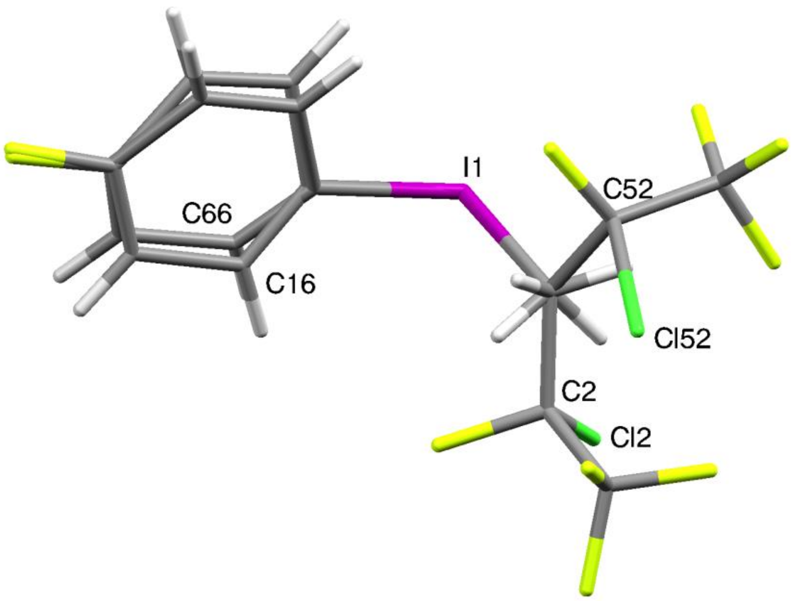

Figure SI4.2 *The two overlayed iodonium ions of the asymmetric unit. Stick model for better visibility. Major component of the second molecule (C52) is shown.*

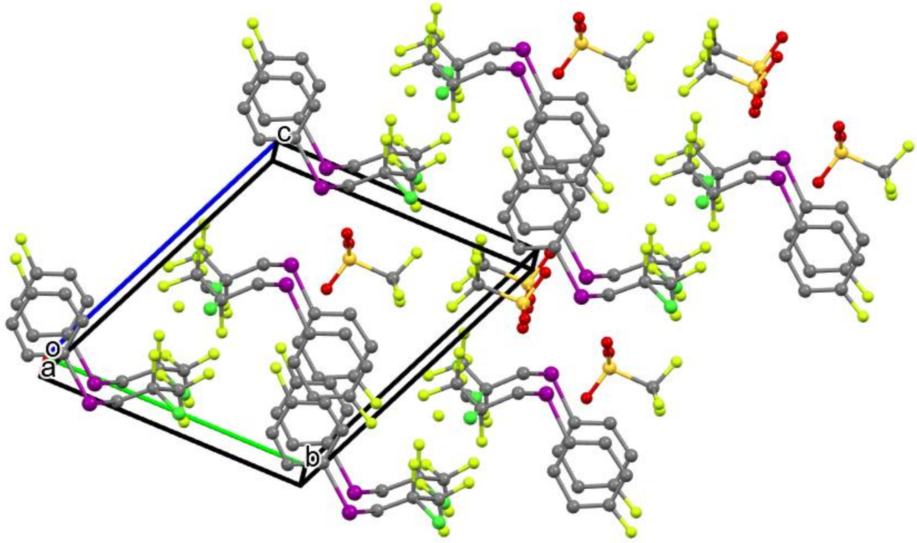

Figure SI4.3 Packing diagram of **10** showing the layered structure. Hydrogen atoms are omitted for clarity.

Table SI4.1 *Experimental details*

Crystal data	
Chemical formula	$C_9H_6ClF_5I{\cdot}CF_3O_3S$
M_r	520.56
Crystal system, space group	Triclinic, *P*1
Temperature (K)	291
a, *b*, *c* (Å)	6.759 (3), 10.882 (5), 12.089 (6)
α, β, γ (°)	64.62 (2), 87.05 (2), 77.10 (2)
V (Å^3)	782.1 (6)
Z	2
Radiation type	Mo *K*α
μ (mm^{-1})	2.45
Crystal size (mm)	0.27 × 0.10 × 0.06
Data collection	
Diffractometer	Bruker D8 VENTURE
Absorption correction	Numerical Krause, L., Herbst-Irmer, R., Sheldrick, G. M., Stalke, D. (2015). "Comparison of silver and molybdenum microfocus X-ray sources for single-crystal structure determination" J. Appl. Cryst. 48, 3-10. doi:10.1107/S1600576714022985
T_{min}, T_{max}	0.75, 0.87
No. of measured, independent and observed [$I > 2\sigma(I)$] reflections	16883, 6319, 5762
R_{int}	0.032
$(\sin \theta/\lambda)_{max}$ (Å^{-1})	0.626

Refinement	
$R[F^2 > 2\sigma(F^2)]$, $wR(F^2)$, S	0.036, 0.107, 1.86
No. of reflections	6319
No. of parameters	408
No. of restraints	382
H-atom treatment	H-atom parameters constrained
$\Delta\rangle_{max}$, $\Delta\rangle_{min}$ (e Å^{-3})	1.15, -0.90
Absolute structure	Refined as an inversion twin.
Absolute structure parameter	0.45 (3)

Table SI4.2 *Geometric parameters (Å, º) for* ***10***

Distances (Å)			
C1—C2	1.47 (3)	C52—F52	1.35 (2)
C1—I1	2.19 (2)	C52—C53	1.37 (3)
C2—F2	1.34 (2)	C52—C53A	1.39 (5)
C2—C3	1.50 (3)	C52—Cl2A	1.89 (3)
C2—Cl2	1.90 (3)	C52—Cl52	1.95 (3)
C3—F3	1.243 (12)	C61—C62	1.27 (3)
C3—F4	1.295 (16)	C61—C66	1.39 (3)
C3—F5	1.300 (17)	C61—I5	2.039 (17)
C11—C12	1.36 (3)	C62—C63	1.53 (3)
C11—C16	1.46 (2)	C63—C64	1.40 (3)
C11—I1	2.145 (18)	C64—F64	1.317 (17)
C12—C13	1.32 (3)	C64—C65	1.32 (3)
C13—C14	1.38 (3)	C65—C66	1.46 (2)
C14—C15	1.32 (3)	O21—S20	1.435 (16)
C14—F14	1.42 (2)	O22—S20	1.462 (16)
C15—C16	1.21 (3)	O23—S20	1.432 (15)
C20—F22	1.17 (2)	O31—S30	1.442 (13)
C20—F21	1.32 (3)	O32—S30	1.425 (16)
C20—F23	1.40 (3)	O33—S30	1.434 (15)
C20—S20	1.837 (18)	C53—F53	1.280 (17)
C30—F31	1.26 (3)	C53—F54	1.29 (2)
C30—F33	1.32 (3)	C53—F55	1.349 (17)
C30—F32	1.43 (3)	C53A—F54A	1.30 (3)
C30—S30	1.79 (2)	C53A—F55A	1.31 (3)
C51—C52	1.54 (3)	C53A—F53A	1.33 (3)
C51—I5	2.115 (19)		
Angles (°)			

C2—C1—I1	109.6 (15)	C53A—C52—Cl2A	113.7 (18)
F2—C2—C1	112.8 (19)	C51—C52—Cl2A	110.8 (15)
F2—C2—C3	109.2 (16)	F52—C52—Cl52	104.0 (14)
C1—C2—C3	118.4 (19)	C53—C52—Cl52	104.4 (15)
F2—C2—Cl2	100.3 (16)	C51—C52—Cl52	102.0 (15)
C1—C2—Cl2	113.3 (16)	C62—C61—C66	119.9 (18)
C3—C2—Cl2	100.8 (14)	C62—C61—I5	123.1 (16)
F3—C3—F4	105.8 (13)	C66—C61—I5	116.8 (13)
F3—C3—F5	109.2 (15)	C61—C62—C63	121 (2)
F4—C3—F5	109.8 (12)	C64—C63—C62	115.6 (16)
F3—C3—C2	100.4 (17)	F64—C64—C65	110.9 (19)
F4—C3—C2	113.2 (14)	F64—C64—C63	125.4 (17)
F5—C3—C2	117.4 (14)	C65—C64—C63	123.7 (15)
C12—C11—C16	124.4 (18)	C64—C65—C66	116.9 (19)
C12—C11—I1	116.3 (13)	C61—C66—C65	122.5 (18)
C16—C11—I1	119.0 (14)	C11—I1—C1	96.3 (7)
C13—C12—C11	113 (2)	C61—I5—C51	98.7 (7)
C12—C13—C14	122 (2)	O23—S20—O21	116.9 (11)
C15—C14—C13	122 (2)	O23—S20—O22	112.9 (11)
C15—C14—F14	114.0 (18)	O21—S20—O22	115.6 (10)
C13—C14—F14	124 (2)	O23—S20—C20	102.4 (10)
C16—C15—C14	123 (2)	O21—S20—C20	100.5 (10)
C15—C16—C11	116 (2)	O22—S20—C20	106.1 (10)
F22—C20—F21	100.7 (18)	O32—S30—O33	113.0 (9)
F22—C20—F23	113 (2)	O32—S30—O31	115.3 (10)
F21—C20—F23	109.9 (18)	O33—S30—O31	113.4 (9)
F22—C20—S20	113.3 (15)	O32—S30—C30	101.7 (10)
F21—C20—S20	115.8 (15)	O33—S30—C30	108.8 (10)
F23—C20—S20	104.6 (14)	O31—S30—C30	103.1 (9)
F31—C30—F33	105 (2)	F53—C53—F54	108.2 (17)
F31—C30—F32	105.4 (19)	F53—C53—F55	109.4 (13)
F33—C30—F32	109.4 (17)	F54—C53—F55	103.7 (18)
F31—C30—S30	117.8 (16)	F53—C53—C52	118.4 (18)
F33—C30—S30	108.9 (15)	F54—C53—C52	104.5 (18)
F32—C30—S30	109.6 (15)	F55—C53—C52	111.4 (16)
C52—C51—I5	114.1 (14)	F54A—C53A—F55A	101 (3)
F52—C52—C53	123 (2)	F54A—C53A—F53A	108 (3)
F52—C52—C53A	101.4 (19)	F55A—C53A—F53A	100 (3)

F52—C52—C51	110.7 (16)	F54A—C53A—C52	115 (3)
C53—C52—C51	110.1 (17)	F55A—C53A—C52	116 (3)
C53A—C52—C51	119 (2)	F53A—C53A—C52	115 (3)
F52—C52—Cl2A	98.1 (16)		
Torsion angles (°)			
I1—C1—C2—F2	-60 (2)	F22—C20—S20—O23	64 (2)
I1—C1—C2—C3	170.5 (14)	F21—C20—S20—O23	179.6 (17)
I1—C1—C2—Cl2	52.8 (17)	F23—C20—S20—O23	-59.3 (18)
F2—C2—C3—F3	-79 (2)	F22—C20—S20—O21	-57 (2)
C1—C2—C3—F3	52 (2)	F21—C20—S20—O21	59 (2)
Cl2—C2—C3—F3	176.5 (12)	F23—C20—S20—O21	180.0 (16)
F2—C2—C3—F4	169.2 (16)	F22—C20—S20—O22	-177.4 (18)
C1—C2—C3—F4	-60 (2)	F21—C20—S20—O22	-61.8 (19)
Cl2—C2—C3—F4	64.2 (15)	F23—C20—S20—O22	59.3 (17)
F2—C2—C3—F5	40 (2)	F31—C30—S30—O32	-60 (2)
C1—C2—C3—F5	170.5 (16)	F33—C30—S30—O32	59.5 (17)
Cl2—C2—C3—F5	-65.4 (16)	F32—C30—S30—O32	179.2 (14)
C16—C11—C12—C13	2 (3)	F31—C30—S30—O33	-179.9 (18)
I1—C11—C12—C13	176.2 (16)	F33—C30—S30—O33	-60.0 (18)
C11—C12—C13—C14	-2 (3)	F32—C30—S30—O33	59.6 (17)
C12—C13—C14—C15	0 (4)	F31—C30—S30—O31	59 (2)
C12—C13—C14—F14	-176 (2)	F33—C30—S30—O31	179.3 (15)
C13—C14—C15—C16	2 (4)	F32—C30—S30—O31	-61.0 (17)
F14—C14—C15—C16	178 (2)	F52—C52—C53—F53	-177.4 (17)
C14—C15—C16—C11	-1 (3)	C51—C52—C53—F53	-44 (3)
C12—C11—C16—C15	-1 (3)	Cl52—C52—C53—F53	65 (2)
I1—C11—C16—C15	-174.6 (16)	F52—C52—C53—F54	-57 (2)
I5—C51—C52—F52	52 (2)	C51—C52—C53—F54	77 (2)
I5—C51—C52—C53	-87 (2)	Cl52—C52—C53—F54	-174.6 (13)
I5—C51—C52—C53A	169.3 (18)	F52—C52—C53—F55	54 (3)
I5—C51—C52—Cl2A	-55.6 (17)	C51—C52—C53—F55	-172.1 (17)
I5—C51—C52—Cl52	162.2 (10)	Cl52—C52—C53—F55	-63.3 (18)
C66—C61—C62—C63	0 (3)	F52—C52—C53A—F54A	-172 (2)
I5—C61—C62—C63	174.9 (14)	C51—C52—C53A—F54A	66 (3)
C61—C62—C63—C64	-4 (3)	Cl2A—C52—C53A—F54A	-68 (3)
C62—C63—C64—F64	-177.2 (16)	F52—C52—C53A—F55A	70 (3)
C62—C63—C64—C65	4 (3)	C51—C52—C53A—F55A	-52 (3)
F64—C64—C65—C66	179.9 (18)	Cl2A—C52—C53A—F55A	174.2 (19)

C63—C64—C65—C66	-1 (3)	F52—C52—C53A—F53A	-45 (3)
C62—C61—C66—C65	3 (3)	C51—C52—C53A—F53A	-167 (2)
I5—C61—C66—C65	-172.1 (16)	Cl2A—C52—C53A—F53A	59 (3)
C64—C65—C66—C61	-2 (3)		

Table SI4.3 *Hydrogen-bond geometry (Å, º) for* ***10***

D—H···*A*	*D*—H	H···*A*	*D*···*A*	*D*—H···*A*
C1—H1*A*···O33^{i}	0.97	2.39	3.16 (2)	136
C1—H1*B*···O23^{ii}	0.97	2.54	3.40 (3)	147
C16—H16···F55	0.93	2.54	3.35 (2)	147
C51—H51*A*···O31	0.97	2.58	3.50 (3)	157
C51—H51*B*···O21^{iii}	0.97	2.48	3.16 (3)	127
C62—H62···F52	0.93	2.63	3.22 (3)	122

Symmetry codes: (i) *x*-1, *y*, *z*-1; (ii) *x*, *y*, *z*-1; (iii) *x*+1, *y*, *z*.

5. Pyrolysis-gas chromatography/mass spectrometry (Py-GC/MS)

Py-GC/MS measurements were carried out by using a Frontier Multi-Shot Pyrolyzer (EGA/Py-3030D) coupled to an Agilent 6890/5973 GC/MS instrument. Approximately 0.12 mg samples in a stainless-steel cup were dropped into the furnace heated to 100 °C. The helium carrier gas flushed the volatile products into the injector of the GC held at 120 °C. The helium flow of the chamber was set at 100 mL min^{-1}. The pyrolysis products were separated on an Agilent DB-1701 capillary column (30 m × 0.25 mm i.d., 0.25 μm film thickness). The GC oven was programmed to hold at 40 °C for 4 min and then increase to 200 °C at a rate of 6 °C min^{-1}. The range of m/z 14–500 was scanned by the mass spectrometer in electron impact mode at 70 eV electron energy. The identification of the pyrolysis products was based on the combined Wiley Registry 9th edition/NIST 2011 mass spectral library.

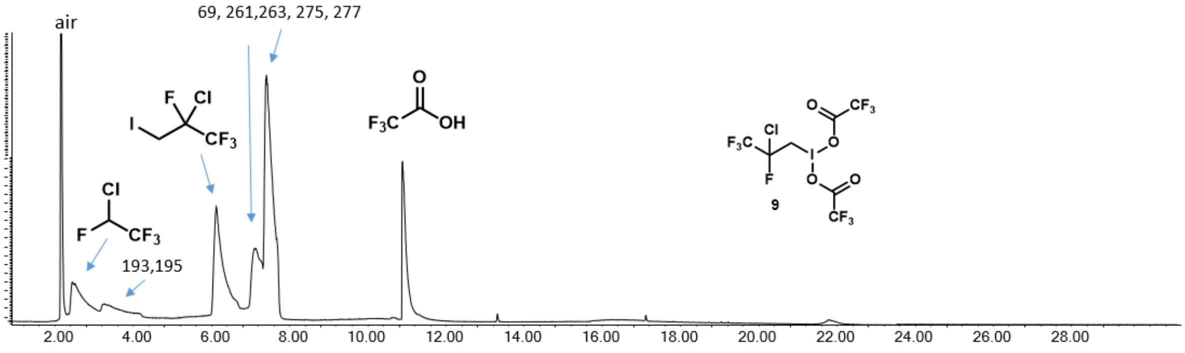

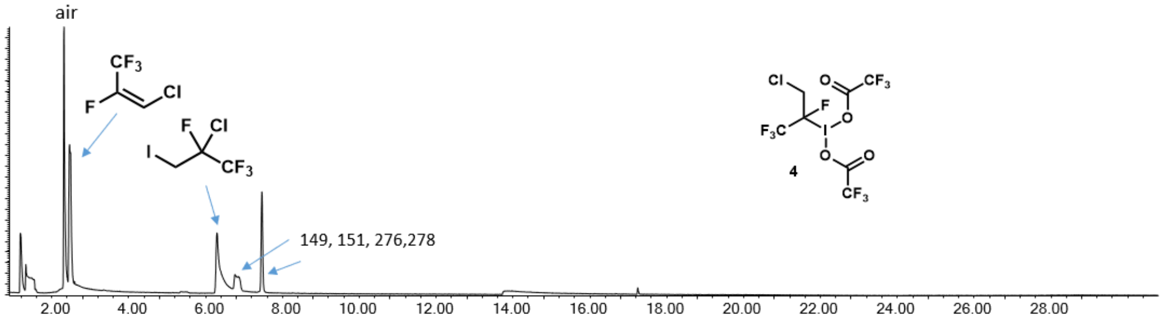

Figure SI5.1 *Py-GC/MS of intermediate* **9** *(above)* and **4** *(below)*

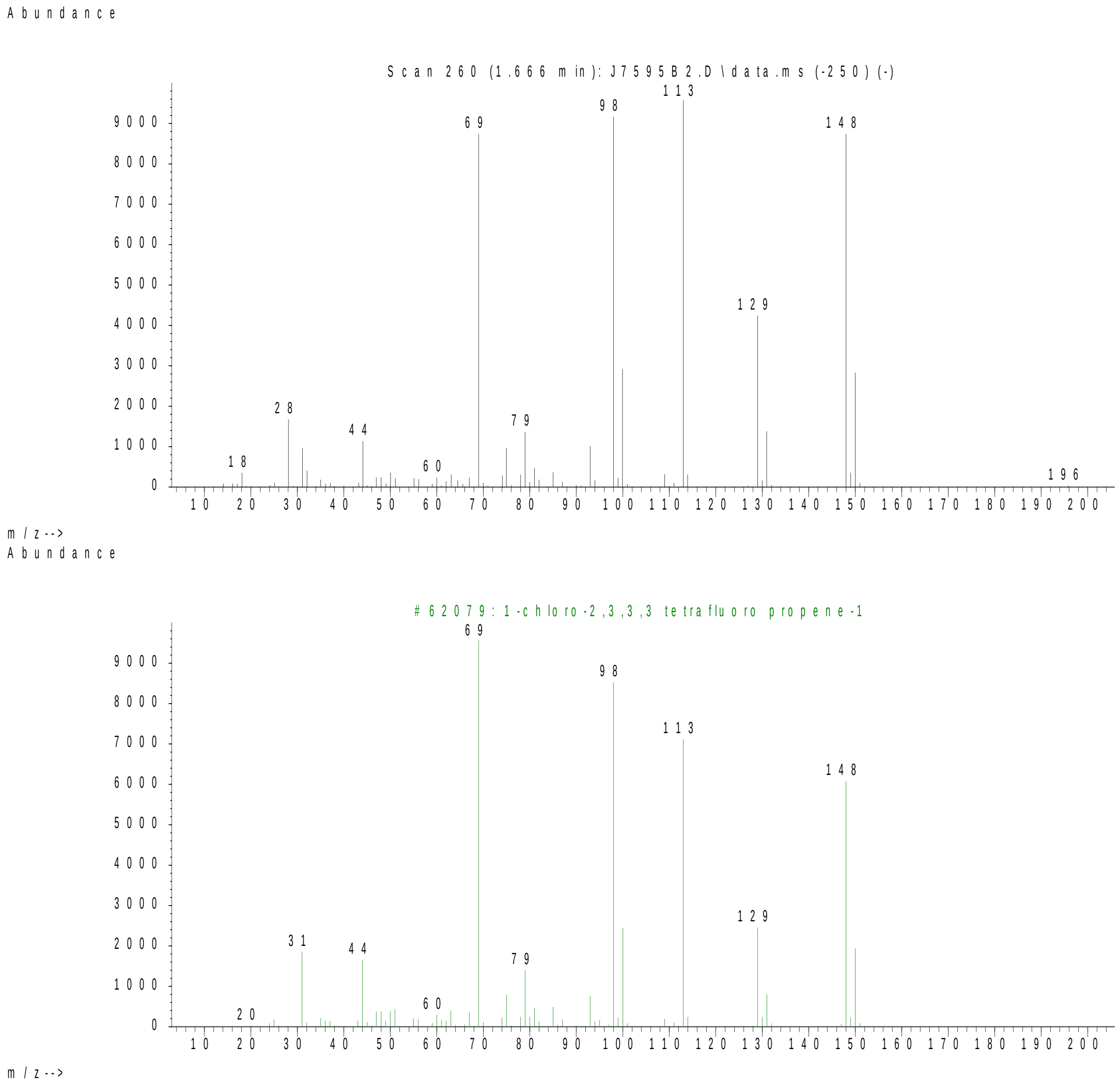

Figure SI5.2 *Mass spectrum of 1-chloro-2,3,3,3-tetrafluoroprop-1-ene (above: measured, below: database)*

6. Theoretical calculations for the rearrangement

6.1 General information

All the Density Functional Theory (DFT) calculations were performed using the Gaussian16 software.[7] For geometry optimizations and frequency calculations, the ω-B97XD functional was used together with the 6-31G* basis set for the atoms with the exception of iodine.[8],[9] For iodine, the LanL2DZ effective core potential (ECP) and the corresponding valence basis set has been selected with an additional set of polarization functions from the corresponding aug-cc-pVDZ-PP set.[10] To refine the obtained energies, single point calculations were performed employing PCM solvation and a larger basis set.[11] This basis set consists of the 6-311++G(3df,3pd) basis for the atoms except iodine, for which we employed the LanL08 ECP and basis set and completed it with the basis set of the d and f shell of the aug-cc-pVTZ-PP set.[12] The solvent used with PCM was acetic acid (ε = 6.25) as Gaussian does not include TFA in its solvent library. We also calculated the energies in ethyl formate which has a dielectric constant (ε = 8.33) closer to the dielectric constant of TFA (ε = 8.55). Between the two model solvents, we observed differences in the calculated relative energies at only the second decimal. The cluster-continuum approach[13] was employed when to calculate the transition barrier of the

dyotropic rearrangement of **4** to **9** and the possible competing elimination reaction of **4** leading to the formation of the gaseous olefinic side product. In this approach two explicite HTFA molecules, H-bonded to the TFA ligands were included to the cluster model. This was necessary as H-bonding situations with the solvent cannot be described by the implicit solvent models. This approach has also been employed for the rearrangement step to calculate the competing barriers on equal footing.

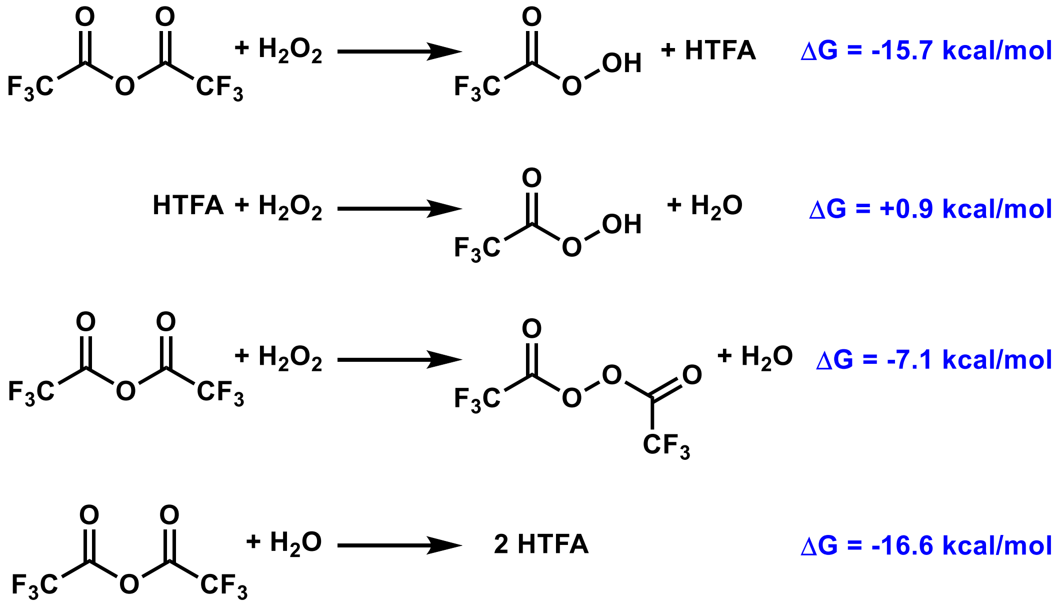

Figure SI6.1 *Possible reactions in the oxidizing mixture of TFAA, HTFA, H_2O and H_2O_2*

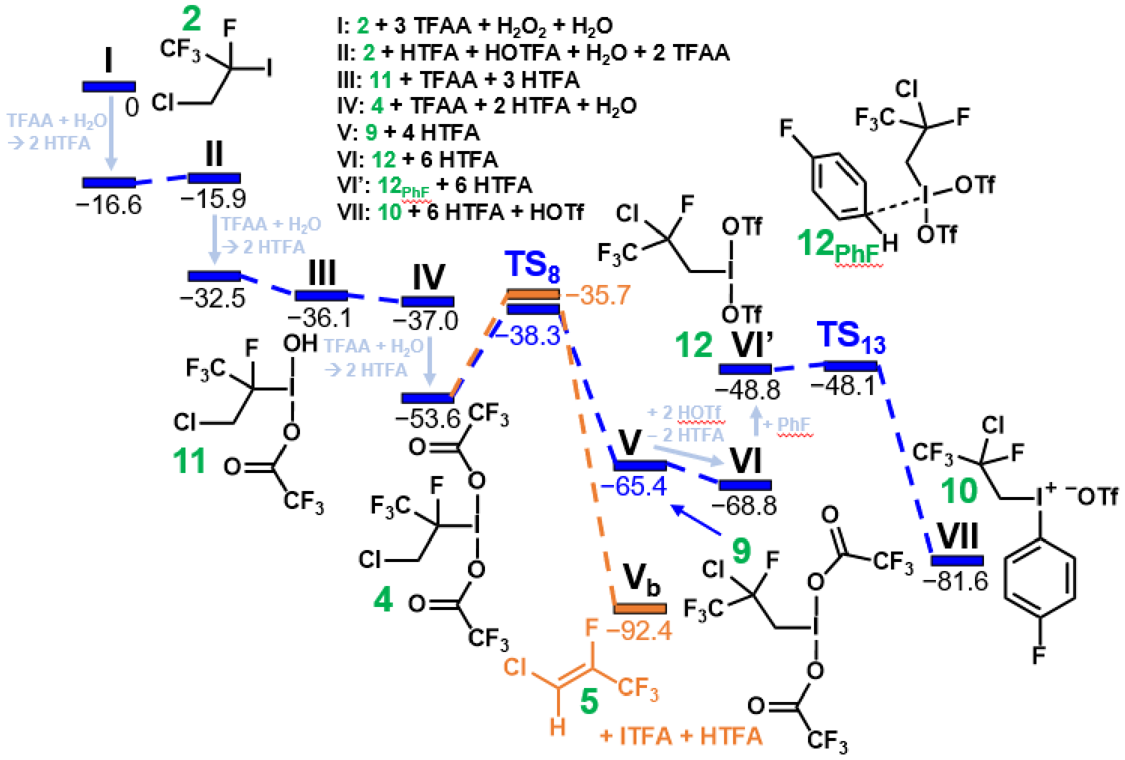

Figure SI6.2 *The detailed mechanism of the main reaction (roman numbering indicates reaction stages)*

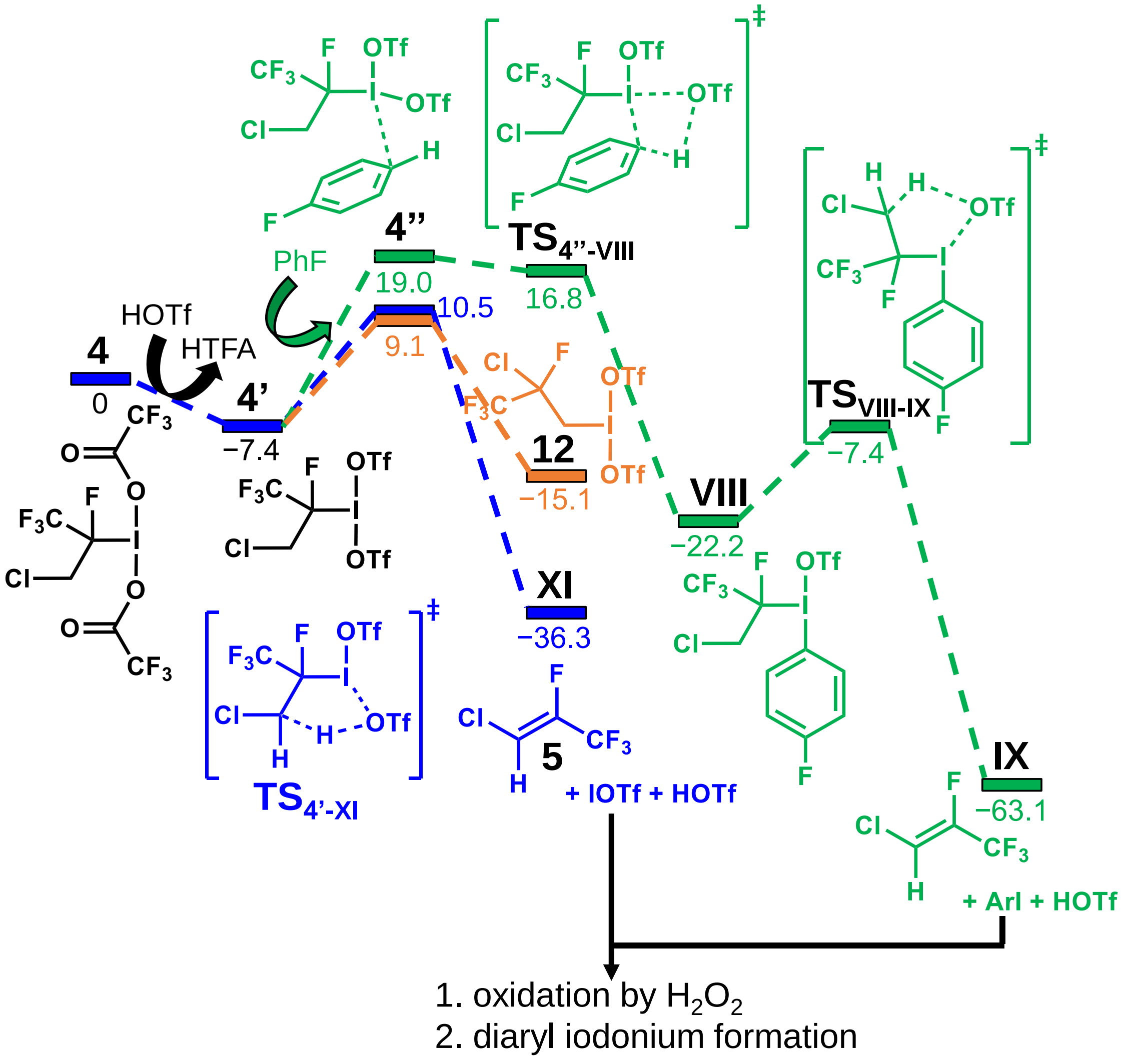

Figure SI6.3 *Energy profile of the side reactions (Note that diaryl-iodonium formation from IOTf and ArI is well-established in the literature and was not calculated in detail.)*

6.2 Calculations on the possible ionic and radical dyotropic pathways

As shown in Scheme I of the article dyotropic rearrangements can in principle follow an ionic pathway where one of the migrating groups dissociates heterolytically and ion pair is formed; and the rearrangement can also follow a radical route if the dissociation is homolytic. We have performed calculations for both the ionic and radical pathways and found that both routes are unfavorable. The calculated route for the ionic pathway is shown in Figure SI6.4. The calculations showed that this state is highly unstable, the ionpair formation requires 199 kcal/mol energy investment considering the Kohn-Sham energies. We note that the iodane cation is not stable in this form and it can decompose to $I(OCOCF_3)_2^+$ cation and the corresponding fluorinated propene, reaching a free energy level which is still 54.2 kcal/mol higher than the TS level of the concerted mechanism. These results clearly indicate that the ionic mechanism cannot play any role under the present experimental conditions.

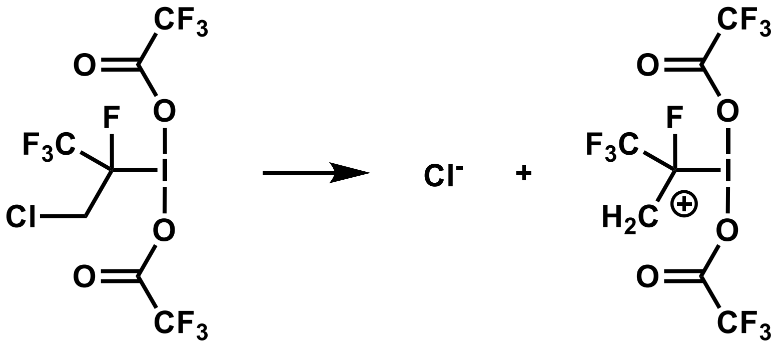

Figure SI6.4 *Calculated reaction route for the ionic mechanism. Note that the cationic iodane species undergoes further decomposition (see text).*

For the radical pathways we could not locate any open-shell singlet states. In order to obtain open-shell singlet states we have employed the option of the Gaussian software to define fragments with open-shell electronic configurations as initial guess for the SCF calculations. This option allows a complete symmetry break of the electronic orbitals and to locate open-shell singlet state if it is the most stable state for the given geometry. In our case we defined two fragments, where one of the fragments is the migrating chlorine radical or the migration iodane radical group and the rest of the molecule represents the other fragment. However, none of our attempts led to stable open shell singlet states; instead, the SCF calculations resulted in a full symmetrization of the alpha and beta orbitals and yielded strictly closed shell electronic configurations. Regarding the open-shell electronic configurations with triplet multiplicity, we have obtained that this state is more than 30 kcal/mol higher than the concerted, closed-shell, singlet TS level.

On the basis of the above results, we exclude the ionic and radical mechanism for this rearrangement.

6.3 Coordinates (.xyz format, in Å) and calculated Gibbs energies (including solvent and large basis electronic energy corrections) of the optimized structures

```
27
4’, G = -2909.493988 a.u.
C      -1.50949200    2.51522200   -0.69302000
H      -1.53821300    3.59534600   -0.52656900
H      -2.33937700    2.04323900   -0.16965300
C      -0.17211300    2.04347000   -0.15191100
C       0.01771100    2.39955100    1.34438000
F      -0.04547700    3.72748000    1.46374000
F      -0.95529700    1.85589900    2.07146800
F       1.19277200    1.98621800    1.79307400
F       0.85143000    2.55670200   -0.83201100
I       0.04122000   -0.19471800   -0.37044300
Cl     -1.67142500    2.22541800   -2.43508200
C      -3.77018800   -2.09431200    0.50462900
F      -4.57378000   -1.71753900   -0.47642200
F      -4.48125900   -2.60077900    1.49958300
F      -2.91516900   -3.00650700    0.05601000
S      -2.82035000   -0.63973400    1.13989000
O      -2.06772900   -0.27361800   -0.19942400
O      -3.76888500    0.40781700    1.44426100
O      -1.89027000   -1.13469100    2.13197100
C       3.90152900   -1.60017200   -0.64589000
F       2.93013200   -2.49495100   -0.82982200
F       4.86909800   -2.14269900    0.07498000
F       4.37414200   -1.21789400   -1.82003100
S       3.20889100   -0.15135900    0.27193500
O       2.64290800   -0.67218600    1.49803700
O       4.20317400    0.89081000    0.26434900
O       2.05707100    0.22031600   -0.75321200

39
4’’, G = -3240.875735 a.u.
I      -0.02163800    0.03987600   -0.89903200
C      -2.73575100   -3.12872500   -0.44828000
C      -3.43987500   -3.02005600    0.74056900
C      -2.80034700   -2.86886000    1.96656200
C      -1.40867100   -2.82789900    1.99528100
C      -0.67493400   -2.94140700    0.81335000
C      -1.34150200   -3.09279200   -0.40616400
H      -3.27494000   -3.24441500   -1.38146100
H      -3.39425200   -2.78592800    2.87017900
H      -0.89594900   -2.71450700    2.94525000
H       0.40877700   -2.92798000    0.83277700
H      -0.76444900   -3.21880200   -1.31795800
F      -4.77608500   -3.05967900    0.71066800
C       2.68729000    2.45664800    0.74555200
F       2.86713200    1.24020400    1.22446500
F       3.09492600    3.35107400    1.63257900
F       3.32498100    2.61761300   -0.39126500
S       0.86211000    2.75527000    0.50009000
O       0.16596500    2.10178400    1.58737400
O       0.69183600    4.14062000    0.16322900
O       0.57124400    1.94279500   -0.88717400
C      -2.54080100    0.52757100    0.78431600
H      -2.03381200    1.29731200    1.36607600
H      -2.24394900   -0.45684600    1.15072500
C      -2.15629800    0.65247300   -0.67553900
C      -2.44934500    2.00994000   -1.35185800
F      -3.74417200    2.10967500   -1.62653700
F      -2.10024300    3.01111100   -0.54048400
F      -1.77567600    2.12784700   -2.49931400
F      -2.75706000   -0.30576400   -1.43638600
Cl     -4.30221000    0.66936800    1.05005600
C       3.50026300   -1.85345700    0.19130100
F       4.53997000   -1.06398200    0.41763800
F       3.88664200   -3.12061300    0.22574000
F       2.59107200   -1.65526500    1.15611700
S       2.74511300   -1.47462400   -1.44963100
O       3.78796100   -1.65106200   -2.43042900
O       1.54395600   -2.32119000   -1.50679600
O       2.31335300   -0.02522900   -1.26135900

12
ArI, G = -342.2297852 a.u.
I      -0.50966600   -1.31544200   -1.95848300
C      -4.35097700    0.49893400   -0.99558200
C      -4.66699600   -0.07734300    0.22558100
C      -3.83207300   -0.99832700    0.84048800
C      -2.64219600   -1.35371700    0.21270400
C      -2.31183300   -0.78213200   -1.01467300
C      -3.16046600    0.14184800   -1.62140700
H      -5.02944600    1.21546600   -1.44498000
H      -4.11377500   -1.42689700    1.79584300
H      -1.98040300   -2.07231800    0.68336200
H      -2.90159000    0.58560000   -2.57638700
F      -5.81232200    0.26616900    0.82936900

3
H2O, G = -76.44030766 a.u.
O      -0.05413400   -0.59471200    0.00000000
H       0.90795900   -0.55155200    0.00000000
H      -0.33460100    0.32660100    0.00000000

4
H2O2, G = -151.5617671 a.u.
O      -1.11126700    0.39543500   -0.03421900
H      -1.31504600    1.33727000    0.06130700
O       0.31896800    0.44159000   -0.05712500
H       0.52274600   -0.05195400    0.75071100

9
HOTf, G = -962.1333744 a.u.
C       0.22317200    0.82840600   -0.01563600
F      -1.10115700    0.84362500    0.08687200
F       0.58911900    1.38977700   -1.15655300
F       0.75620500    1.48328900    1.00100200
S       0.80559300   -0.92752400   -0.01201700
O       0.10281500   -1.61452300   -1.07027500
O       2.23536800   -0.90917600    0.11905700
O       0.19085000   -1.39243000    1.40168800
H      -0.64920100   -1.85367000    1.23782000

9
HOTFA, G = -601.963103 a.u.
C      -0.00951500   -0.23362800   -0.02275400
O       1.16737200   -0.37926000   -0.13570000
O      -0.69119400    0.91906100    0.07576000
C      -1.04027100   -1.38728000    0.00459500
F      -1.86815600   -1.26378000    1.04586900
F      -0.40380400   -2.54535300    0.09453500
F      -1.76701500   -1.37621500   -1.11683600
O       0.21835100    2.01318900    0.00392500
H      -0.39065100    2.71477200    0.28444300

8
HTFA, G = -526.8423863 a.u.
C      -0.05387800   -0.20450200   -0.00098600
O       1.13886000   -0.32457400   -0.00092800
O      -0.72691900    0.94537900    0.00071600
H      -0.07257700    1.66340900    0.00117000
C      -1.03617300   -1.39294000   -0.00014100
F      -1.81465200   -1.34622700    1.08505300
F      -0.36806600   -2.53820800   -0.00150600
F      -1.81768100   -1.34542800   -1.08308700

9
IOTf, G = -972.8962548 a.u.
I       0.51560800   -1.29476300   -0.19371900
```

```
C      4.38474100   -1.06917900    0.69696200
F      3.67529800   -1.98855000    1.34003000
F      5.28093400   -0.54341700    1.51636100
F      4.98842700   -1.61769200   -0.34205800
S      3.25596100    0.28681600    0.12458800
O      2.53919800    0.77017900    1.27904800
O      4.01560200    1.13688400   -0.75273800
O      2.28795200   -0.58286800   -0.83672700

8
ITFA, G = -537.6089455 a.u.
C      0.83099300   -1.54654200    0.00169700
O      2.01325400   -1.72776400    0.00158600
O      0.18102600   -0.38254400    0.00181400
C     -0.21222700   -2.69206900    0.00181200
F     -0.98467100   -2.60990000    1.08655500
F      0.40769500   -3.86347200    0.00162700
F     -0.98508200   -2.60977700   -1.08262500
I      1.31553400    1.28259300    0.00178600

12
PhF, G = -331.4239907 a.u.
C      0.59539300   -0.36264200    0.00010900
C      1.98234500   -0.33633400    0.00061800
C      2.69872400    0.85159100   -0.00000500
C      1.99479900    2.05291800   -0.00104800
C      0.60156000    2.05562700   -0.00147500
C     -0.09282900    0.84771700   -0.00094900
H      0.07730900   -1.31538900    0.00052600
H      3.78285800    0.82374300    0.00032600
H      2.54168300    2.99097400   -0.00151400
H      0.05887400    2.99550300   -0.00227500
H     -1.17864700    0.84332600   -0.00133300
F      2.65366500   -1.49923500    0.00140700

9
propene_sideproduct (5), G = -974.5110119 a.u.
C     -0.09303900   -0.63283300    0.62172800
C      0.82550700   -1.32380000   -0.03912400
H     -0.05333700   -0.53003100    1.69692000
C      1.98869900   -2.01936200    0.61715100
F      3.14773600   -1.54576800    0.14727900
F      1.96735500   -1.84272500    1.94317900
F      1.95585400   -3.33249500    0.36631900
F      0.81974100   -1.47869400   -1.35753900
Cl    -1.40863100    0.14856300   -0.16153400

14
TFA_peroxo_anhydride, G = -1052.352927 a.u.
C     -0.26895800    0.04296200    0.10007000
O      0.74047000    0.29366500    0.67333300
O     -1.02820900    0.90466100   -0.63384300
C     -0.94996200   -1.34493400    0.03473100
F     -2.23461900   -1.25391600    0.37200800
F     -0.33410100   -2.16993000    0.86545600
F     -0.86261300   -1.82401900   -1.20778900
O     -0.40375300    2.16832700   -0.71103700
C     -0.70538500    2.91674500    0.38760400
O     -1.33842100    2.57346800    1.33193300
C     -0.08525500    4.31850700    0.17575600
F      1.21319800    4.21768800   -0.10120700
F     -0.69758900    4.92824400   -0.84084800
F     -0.24619400    5.03490500    1.27617200

13
TFAA, G = -977.218494 a.u.
C     -0.09352800   -1.80594900   -0.03971000
O      1.04877900   -2.05458000    0.16130400
O     -0.63839300   -0.56800000   -0.26580300
C      0.02112600    0.57588300    0.10444400
O      0.89385500    0.66731500    0.90261400
C     -0.60221000    1.75751000   -0.66714100
C     -1.22443200   -2.85067400   -0.13824900
F     -0.73131800   -4.06460300    0.04821900
F     -2.14715700   -2.60134300    0.79312400
F     -1.79977800   -2.78803300   -1.34006600
F     -0.45191800    1.56559500   -1.97924600
F     -1.90463400    1.84486200   -0.39248500
F     -0.00470300    2.88581900   -0.31865200

41
TS_4-5 (TS_4-Vb), G = -3092.539623 a.u.
C     -0.89447100   -1.14285600    1.23209300
H     -1.75544900   -1.37081900    2.04152700
H     -0.31076700   -2.06367200    1.32052400
C     -0.20275400   -0.01180100    1.72122800
C     -0.39969600    1.45329000    1.26826800
F      0.20918400    1.55008100    0.08471100
F     -1.67903700    1.74859500    1.14167100
F      0.18237400    2.27434600    2.12207800
F      0.83453100   -0.16878000    2.45372300
I     -1.73111500    0.59268000    4.48246900
Cl    -1.71535600   -0.96715000   -0.31478900
O      0.20744400    1.30694000    4.97680500
C      1.02244300    0.51966800    5.60591200
O      0.83637900   -0.60612000    6.02491000
O     -3.75865300   -0.22265800    3.87730500
C     -3.82369200   -1.37425300    3.38494500
O     -2.94290700   -1.99422400    2.74415000
C      2.40883000    1.18885500    5.76378600
C     -5.14028000   -2.12222600    3.71029100
F      2.95834600    1.33921300    4.54972800
F      2.29562100    2.39131800    6.32819100
F      3.21512800    0.44004200    6.50416100
F     -5.17601100   -2.33939200    5.02855600
F     -6.19495600   -1.39723300    3.36541500
F     -5.19855500   -3.30366000    3.09364700
O     -0.73443600   -2.77922800    5.58152100
C     -0.58160300   -3.12685100    4.32978700
O      0.11994400   -2.58758100    3.50452200
C     -1.40623800   -4.38515700    3.99011800
F     -0.79441900   -5.46729700    4.47790300
F     -2.63289000   -4.32132700    4.50707400
F     -1.50568800   -4.51761200    2.66537800
O     -3.71895100   -3.59477000    0.60880300
C     -4.58161200   -2.75206000    0.06989500
O     -5.06417700   -1.77649600    0.58462700
C     -4.88432400   -3.15024100   -1.38768700
F     -3.80819700   -2.90021200   -2.14989000
F     -5.17218000   -4.44929700   -1.48405900
F     -5.90644000   -2.44690900   -1.85840800
H     -3.50506300   -3.27360400    1.51249700
H     -0.19060800   -1.96869800    5.76574100

39
TS_4''-VIII, G = -3240.879332 a.u.
I     -0.10163300   -0.00267800   -0.58642100
C     -2.79263300   -3.31395900   -1.04524800
C     -3.12048000   -3.50623400    0.30071800
C     -2.32252100   -3.07363400    1.36891600
C     -1.15560600   -2.41794100    1.07123900
C     -0.76700500   -2.16074300   -0.28934800
C     -1.63714500   -2.62998100   -1.33181700
H     -3.45230200   -3.69509800   -1.81566500
H     -2.63062500   -3.28847600    2.38544300
H     -0.47384100   -2.12100700    1.86348100
H      0.31340200   -2.59559100   -0.45000400
H     -1.34454600   -2.46471300   -2.36464000
F     -4.23471000   -4.14507500    0.58254300
C      2.62134600    2.85828000    0.13689500
F      2.89598600    1.56155900    0.27360000
F      3.23744700    3.53807200    1.09413100
F      3.04586100    3.27206000   -1.04680400
S      0.79855400    3.11176900    0.28467000
O      0.43537700    2.55304500    1.58440800
O      0.53864000    4.50216500   -0.00566900
O      0.28408100    2.20371600   -0.85615600
C     -2.20397300    0.93552100    1.43763900
```

H	-1.55368200	1.78158300	1.66595300
H	-1.84619800	0.05795300	1.97732600
C	-2.19110600	0.64132500	-0.05017400
C	-2.67414300	1.80093900	-0.97594800
F	-4.00121800	1.73123500	-1.07452600
F	-2.34755000	2.98184700	-0.47035900
F	-2.17169200	1.68400000	-2.20613200
F	-2.98301700	-0.42255600	-0.33970900
Cl	-3.85562000	1.27412100	2.03334400
C	3.23020700	-1.96785000	0.99514100
F	4.14952600	-1.01591800	1.01216400
F	3.68552200	-3.03330900	1.64016700
F	2.13787900	-1.51665700	1.64890000
S	2.75374100	-2.40084700	-0.73238100
O	3.94699000	-2.89573800	-1.37858700
O	1.66127400	-3.39570400	-0.51080900
O	2.18804400	-1.12738200	-1.24805300

27
TS_4'-12, G = -2909.467751 a.u.

C	-0.19431000	1.56303300	1.42733900
H	0.80134200	1.36240000	1.82317200
H	-1.04369700	0.95625800	1.75521600
C	-0.24329900	1.93889200	0.02986500
C	0.92339000	2.51447600	-0.81682600
F	0.43676800	3.50693000	-1.55436800
F	1.89176800	2.96193700	-0.04631100
F	1.35445100	1.54895600	-1.60699400
F	-1.30123900	1.75745100	-0.65670300
I	0.01745600	-1.52190100	-0.09676300
Cl	-0.62454400	3.35157000	1.60967100
C	-4.52696800	-0.33618500	-0.57681400
F	-4.84371200	-1.24591200	-1.48403700
F	-4.17258400	0.79274300	-1.18759400
F	-5.57583500	-0.09857500	0.19993500
S	-3.13534000	-0.93809200	0.47733800
O	-2.08273900	-1.10800500	-0.63371300
O	-2.80081900	0.19175200	1.34983700
O	-3.54755700	-2.18264000	1.08494900
C	4.15031800	-0.65865300	-0.65350600
F	4.81769100	-1.77796500	-0.88264900
F	5.00591200	0.34171600	-0.47709000
F	3.37571300	-0.38656200	-1.70282000
S	3.10617700	-0.83829100	0.85832200
O	3.96763200	-1.31907700	1.91142600
O	2.45269100	0.46424400	1.01440500
O	2.11652600	-1.91481700	0.39090200

27
TS_4'-XI, G = -2909.465571 a.u.

C	-1.97850900	2.24560200	0.01195600
H	-2.28762600	2.88944400	0.84791300
H	-2.16016300	1.19440200	0.57777900
C	-0.59942100	2.41028200	-0.24252500
C	0.39189100	2.70161600	0.90420800
F	0.13696700	3.96435600	1.27589400
F	0.16832100	1.89581700	1.92210900
F	1.63314000	2.61935000	0.47979800
F	-0.14561000	2.65379700	-1.41310700
I	0.40334400	-0.56283300	-0.70567900
Cl	-3.04850300	2.35445900	-1.36597200
C	-4.25102400	-1.63839100	0.14002100
F	-4.71315400	-0.62852500	-0.58630700
F	-5.01107100	-1.79487500	1.21388100
F	-4.26017900	-2.74743100	-0.58096200
S	-2.52415800	-1.27232900	0.68605700
O	-1.84038500	-1.08496300	-0.64966000
O	-2.62338800	0.04946500	1.37872600
O	-2.06764100	-2.37841400	1.49107500
C	4.20904000	-1.61219400	-0.04569500
F	3.27016000	-2.55326200	0.01716100
F	5.11407900	-1.83088600	0.89718800
F	4.78745400	-1.64988700	-1.23634700
S	3.44052600	0.04620700	0.23409400
O	2.77531500	-0.01476300	1.51915000
O	4.45541200	1.03310600	-0.04677900
O	2.39823700	0.06317900	-0.94715800

39
TS_13, G = -3240.897292 a.u.

C	-1.85797400	0.39008400	0.40449200
H	-2.00712100	-0.44822500	1.08650100
H	-1.75264200	1.31496700	0.97601100
C	-3.00475100	0.44692600	-0.59454400
C	-4.34461300	0.21143400	0.15006300
F	-4.31495500	-1.01158300	0.70992900
F	-4.50350200	1.10947000	1.11823900
F	-5.37734300	0.26115500	-0.67673000
F	-2.91175800	-0.57379800	-1.50044000
Cl	-3.06939300	1.97490000	-1.47953900
I	0.08002100	0.06399100	-0.46143000
C	-2.22857200	-3.46612500	-0.27154000
C	-2.23101400	-3.62891900	1.11514900
C	-1.24496700	-3.10169600	1.96058300
C	-0.23925800	-2.36119100	1.38974800
C	-0.18605000	-2.13741100	-0.02746700
C	-1.21614700	-2.72111900	-0.83100300
H	-3.01807200	-3.91242100	-0.86424100
H	-1.29637200	-3.29378900	3.02602600
H	0.55345500	-1.94868500	2.00683800
H	0.92666800	-2.48773300	-0.41592700
H	-1.18438400	-2.57478800	-1.90576900
F	-3.20814200	-4.32277700	1.66063200
C	2.08696400	3.36155100	0.61736800
F	2.54487600	2.12507900	0.83834400
F	2.39546100	4.12565600	1.65684500
F	2.66066300	3.84411100	-0.47408200
S	0.25245500	3.31018500	0.40905600
O	-0.25591400	2.70982600	1.64208100
O	-0.16187300	4.64354700	0.03675700
O	0.11345600	2.32975300	-0.76665700
C	3.90295500	-1.48067300	0.53534300
F	2.90183700	-1.14389900	1.37431600
F	4.70914800	-0.43924000	0.40794600
F	4.56796000	-2.49555800	1.06881200
S	3.18016400	-1.94946200	-1.09468400
O	2.43526000	-0.73682100	-1.49144600
O	4.27595800	-2.37764600	-1.93081300
O	2.21780200	-3.03996300	-0.69928100

30
TS_VIII-IX, G = -2278.784905 a.u.

I	-0.46431400	-0.78785700	-0.73343800
C	-4.76252900	-0.25628400	-1.13722200
C	-5.26330000	-0.93722000	-0.03697300
C	-4.43544400	-1.58093600	0.87271900
C	-3.06026000	-1.53713000	0.67581200
C	-2.53946500	-0.85451400	-0.42414900
C	-3.38605400	-0.21798200	-1.33230600
H	-5.44419700	0.22917200	-1.82640300
H	-4.86848400	-2.10445700	1.71758000
H	-2.40095600	-2.03190700	1.38090800
H	-2.98049100	0.30569100	-2.19159800
F	-6.58570000	-0.97591000	0.15303600
C	3.53243700	-1.57274800	0.65532100
F	2.38286300	-1.72978000	1.32140700
F	4.48973000	-1.27389500	1.52500700
F	3.83158600	-2.71715600	0.05092000
S	3.35258000	-0.23167300	-0.59801100
O	2.96044800	0.93309300	0.28693400
O	4.64655900	-0.09368600	-1.22910200
O	2.21794100	-0.65591700	-1.43188900
C	0.86139900	2.41430300	-0.11162400
H	1.80088900	1.59475800	-0.03353800
H	1.29353300	3.09785200	0.63264100
C	-0.33883400	1.85022700	0.37704600
Cl	0.78077900	3.12670100	-1.70838900
C	-0.44547300	1.48764700	1.86784200

```
F      -1.53751800    0.78127600    2.12033800
F       0.62888400    0.82985300    2.26801000
F      -0.52507400    2.64264100    2.54335000
F      -1.49372600    2.10719700   -0.16461300

30
VIII, G = -2278.808447 a.u.
I      -0.42168400   -0.39009400   -0.65781800
C      -4.72912700   -0.10978800   -1.15938700
C      -5.20986900   -0.87065600   -0.10143800
C      -4.36810600   -1.48618800    0.81620700
C      -2.99532200   -1.33092100    0.67194000
C      -2.49733100   -0.56839500   -0.38561600
C      -3.35571300    0.03887100   -1.30410100
H      -5.42483500    0.35138900   -1.85088700
H      -4.78986700   -2.07105400    1.62550800
H      -2.32436900   -1.79831200    1.38471600
H      -2.96169300    0.63144200   -2.12339600
F      -6.52760200   -1.01697400    0.03766000
C       3.24090600   -1.58637400    0.55773600
F       2.17345900   -1.59142700    1.37537500
F       4.33412000   -1.72122800    1.29697000
F       3.13227800   -2.63492300   -0.25686700
S       3.29330100   -0.01809800   -0.41287900
O       3.32724200    1.04349400    0.59393900
O       4.42343700   -0.16406300   -1.30818500
O       1.96243500   -0.06254700   -1.12527100
C       0.63700500    2.53037000   -0.18256600
H       1.63921800    2.10481800   -0.09069800
H       0.57863600    3.45645200    0.39550800
C      -0.40840600    1.60072100    0.39272300
Cl      0.29796900    2.93040500   -1.88041500
C      -0.22886400    1.34842600    1.90939800
F      -1.20909600    0.56940200    2.37518700
F       0.93638900    0.78332600    2.16984700
F      -0.29965900    2.52382900    2.53950400
F      -1.65291300    2.08012500    0.22068400

27
12, G = -2909.506374 a.u.
C      -0.28226600    0.87264000    0.48427000
H       0.60992300    0.95665000    1.10946100
H      -1.16803000    0.70744000    1.09933700
C      -0.47384900    2.05608800   -0.45089700
C       0.79308100    2.47370900   -1.24052000
F       0.58751300    3.60522900   -1.90044500
F       1.82206800    2.62677800   -0.40991200
F       1.09566000    1.52188800   -2.12949700
F      -1.44197100    1.80413300   -1.36111000
I      -0.05326000   -1.02093200   -0.51203600
Cl     -0.97494100    3.41653000    0.58860100
C      -4.30824400   -0.02234400    0.24290000
F      -4.84267200   -0.11266800   -0.96209200
F      -3.66359800    1.13624500    0.35968400
F      -5.25575400   -0.09265400    1.16418800
S      -3.11514500   -1.40876300    0.52141400
O      -2.15944700   -1.11914800   -0.69884000
O      -2.41869600   -1.11137500    1.76244000
O      -3.82937600   -2.64611200    0.33715200
C       4.47938300   -0.26270400    0.18408900
F       4.87731400   -0.95164700   -0.87244800
F       5.37226300   -0.37316000    1.15601600
F       4.32655500    1.01144000   -0.14026500
S       2.87692500   -0.94875000    0.80996100
O       3.05570900   -2.36215500    1.04628100
O       2.40735800   -0.07774600    1.87413700
O       2.03668900   -0.68492800   -0.49967900

11
2, G = -986.5354068 a.u.
C      -1.22897100    0.56648400    0.03701300
H      -0.91034400    1.12495700   -0.84339400
H      -2.31497600    0.49109100    0.05232000
C      -0.73107100    1.29130200    1.27779400
C      -1.19987000    2.77007900    1.25525700
F      -0.70400200    3.36782000    0.16525400
F      -2.53265900    2.83745000    1.19919000
F      -0.77628600    3.43190400    2.32378300
F       0.62346100    1.33056800    1.29872800
Cl     -1.31222800    0.52983000    2.78178600
I      -0.44669700   -1.41018000   -0.18814300

25
4, G = -2038.901194 a.u.
C      -0.01273700    1.69855800   -1.13631000
H      -0.92392600    1.30054200   -1.58051400
H       0.85943400    1.30512600   -1.65839100
C       0.08690800    1.29796600    0.32415500
C      -1.06161400    1.77655600    1.26570300
F      -0.79098100    3.01477500    1.65918300
F      -2.22302400    1.77120300    0.61448000
F      -1.16602000    1.01202600    2.35115700
F       1.24472800    1.67445100    0.87456000
I       0.17101000   -0.96999100    0.37590000
Cl     -0.00770500    3.47744300   -1.30863400
O       2.25494100   -0.78225300    0.62680800
C       2.99164900   -0.40752500   -0.39393300
O       2.60965300   -0.00637200   -1.46644600
O      -1.94198900   -1.01843300    0.40416800
C      -2.60650800   -0.85385700   -0.71693200
O      -2.16347700   -0.52740100   -1.79136400
C       4.49470900   -0.54471000   -0.05737100
C      -4.11175700   -1.13155200   -0.49667300
F       4.79322000    0.19108400    1.01632900
F       4.78582600   -1.82227600    0.20704400
F       5.23942500   -0.13787500   -1.07683800
F      -4.28826000   -2.39549800   -0.09952500
F      -4.59442900   -0.31771800    0.44598100
F      -4.79072000   -0.93676000   -1.61967500

20
11, G = -1588.497587 a.u.
C      -1.00679400   -0.25596500    1.07184100
H      -0.75559000    0.22605100    0.12363800
H      -1.94953900   -0.79999900    0.99811000
C      -1.10118300    0.81928600    2.14086200
C      -2.05509800    1.96712900    1.70890600
F      -1.39496600    2.71375000    0.81695600
F      -3.14692000    1.49128700    1.11220200
F      -2.40596700    2.74223300    2.72635900
F       0.09548400    1.38192000    2.37828100
I      -1.67324100   -0.06059300    4.11288200
Cl      0.28618000   -1.43327100    1.43045800
O       0.24896400   -0.04567000    4.60979500
O      -3.75550900    0.18116600    3.46716100
C      -4.27210500   -0.68207700    2.64796500
O      -3.69554900   -1.50927000    1.96987500
C      -5.81176600   -0.54820500    2.59231700
F      -6.33899900   -0.74679700    3.80635400
F      -6.15211000    0.67954100    2.18186400
F      -6.33990500   -1.43400500    1.75319700
H       0.70462000   -0.76059800    4.13793500

25
9, G = -2038.919996 a.u.
C      -1.15865200    0.50165600   -0.15286800
H      -0.68348900    1.03207500   -0.97877200
H      -2.23812300    0.44464600   -0.28536700
C      -0.78665000    1.17101300    1.16496100
C      -1.15250000    2.67791800    1.09806900
F      -0.47224700    3.24264300    0.09566600
F      -2.45730100    2.82323000    0.85664800
F      -0.84196400    3.29955000    2.22496200
F       0.55126500    1.10796100    1.36319900
Cl     -1.60581000    0.42679800    2.55675400
I      -0.44641000   -1.49376500   -0.31374900
O      -2.44320900   -1.89288600    0.35446000
C      -2.58586800   -3.17527900    0.55191000
```

O	-1.73181800	-4.01494900	0.36580100
C	-3.99867400	-3.52509800	1.06651900
F	-4.23051600	-2.89497000	2.22256000
F	-4.91802500	-3.13148800	0.17874400
F	-4.11971100	-4.83151100	1.25809900
O	1.28598900	-0.43525900	-0.99816000
C	2.22592000	-1.29449500	-1.28397900
O	2.13336900	-2.49846200	-1.17584000
C	3.50995700	-0.60134500	-1.78814500
F	3.23580500	0.13587700	-2.86977300
F	3.99478600	0.20455400	-0.83888200
F	4.43448500	-1.49787600	-2.10401800

39
12_PhF, G = -3240.898418 a.u.

C	-1.89371400	0.15983300	0.18087100
H	-2.11585000	-0.76873000	0.71645900
H	-1.92790300	0.99316300	0.88358200
C	-2.87696100	0.32662100	-0.96403000
C	-4.33257800	0.31081300	-0.42698200
F	-4.55332800	-0.84508100	0.21186400
F	-4.51556400	1.30792100	0.43770300
F	-5.20975400	0.41655300	-1.41467700
F	-2.77380700	-0.72159400	-1.82538500
Cl	-2.61696900	1.83044100	-1.87533000
I	0.15808700	-0.07717400	-0.40819200
C	-2.76952300	-3.36339900	0.45990600
C	-2.28118800	-3.17482200	1.75194400
C	-0.93619500	-2.95639800	2.01278400
C	-0.04649700	-2.93895300	0.93847800
C	-0.51179200	-3.11343300	-0.37357200
C	-1.87670400	-3.32794400	-0.60173400
H	-3.82962300	-3.53097400	0.31050400
H	-0.59974600	-2.82414500	3.03486600
H	1.01662800	-2.80517000	1.11148100
H	0.20305000	-3.14797300	-1.18927900
H	-2.23955300	-3.47478100	-1.61297100
F	-3.14211800	-3.20914400	2.77212000
C	2.24301300	2.96958500	1.00582400
F	2.62763600	1.81974700	1.53273000
F	2.45207400	3.94879800	1.87372400
F	2.88379000	3.21069500	-0.11529700
S	0.40888800	2.91225200	0.69259600
O	-0.20395200	2.27672600	1.84125600
O	0.00604000	4.20402500	0.20666700
O	0.31586600	1.92603200	-0.58472400
C	4.04991300	-1.04575400	0.53568500
F	3.12334200	-1.06645500	1.50737900
F	4.85088700	-0.00827200	0.73698400
F	4.75473300	-2.16750200	0.60412300
S	3.21305400	-0.92130100	-1.10348300
O	2.47501300	0.39226100	-0.98222500
O	4.25990600	-0.93413800	-2.09815600
O	2.24168100	-2.02921500	-1.07801100

30
10, G = -2278.819026 a.u.

C	-0.79247300	0.97221200	0.30893700
H	0.19153300	1.13863800	-0.13394600
H	-1.56463100	1.24738100	-0.41050400
C	-0.93499500	1.79238100	1.57585500
C	-0.62935100	3.28795700	1.31232700
F	0.62932600	3.39375700	0.86934300
F	-1.44810200	3.76969500	0.37640600
F	-0.75039600	4.01181300	2.41560100
F	-0.02799900	1.38674200	2.51079500
Cl	-2.56789700	1.62905200	2.26904800
I	-0.97102700	-1.16488200	0.66110000
C	1.08595900	-1.44836300	0.28216900
C	2.02047300	-0.69015000	0.97898700
C	1.45118400	-2.40872200	-0.65559400
C	3.37135900	-0.88580300	0.71228600
H	1.71673900	0.04382000	1.71631100
C	2.80284100	-2.60387900	-0.91204200
H	0.70797400	-3.00967000	-1.16400300
C	3.73796400	-1.83952300	-0.22705100
H	4.13399000	-0.31803800	1.23260000
H	3.13339000	-3.34296800	-1.63254200
F	5.03408400	-2.02830400	-0.47930900
C	-1.90609900	-4.74184400	-1.11479200
F	-2.99395700	-5.34077700	-1.57808100
F	-0.85964400	-5.55005700	-1.27204500
F	-1.68230800	-3.63711000	-1.85340900
S	-2.10816300	-4.27223100	0.65860700
O	-2.24315300	-5.51862100	1.38107900
O	-0.78855300	-3.56679200	0.90169300
O	-3.21558100	-3.31503800	0.66245900

41
TS_8, G = -3092.543806 a.u.

C	-0.01053000	-0.40082700	1.70842500
H	0.67505300	0.41633200	1.47708900
H	0.03394500	-1.26415100	1.04272500
C	-1.34613500	-0.05158300	2.14708800
C	-1.77300300	1.27444200	2.83389800
F	-2.14676900	1.02010300	4.08212100
F	-0.75679200	2.11942200	2.82907800
F	-2.80033900	1.77455600	2.17232600
F	-2.31453500	-0.86149400	1.97963500
I	-1.39258000	1.38017400	-0.92228600
Cl	0.16882400	-0.93081900	3.45035400
O	-3.18909000	0.23571900	-0.45204400
C	-3.62880300	-0.69174300	-1.22637300
O	-3.18142700	-1.09889900	-2.28650800
O	0.38889800	2.61995500	-1.28868600
C	1.47540600	2.42883700	-0.65730900
O	1.69494200	1.66306900	0.28368300
C	-4.92139300	-1.32016500	-0.65088900
C	2.62305800	3.32166700	-1.19148500
F	-4.67466100	-1.82151900	0.57026900
F	-5.87914600	-0.39548500	-0.53394600
F	-5.36817400	-2.30149900	-1.42396600
F	2.83044400	3.08620800	-2.48690000
F	2.31128100	4.61004100	-1.03396400
F	3.75665400	3.07854500	-0.52968200
O	-0.65574500	-1.76420400	-2.78618600
C	-0.17502400	-2.06041300	-1.61166300
O	-0.78770500	-2.15449700	-0.56851800
C	1.35920800	-2.20767300	-1.63795300
F	1.79903600	-2.78915800	-2.74603700
F	1.89841300	-0.97873400	-1.55313300
F	1.76618400	-2.91195800	-0.58308600
O	3.98072500	0.23003900	0.34394900
C	3.65992200	-0.88061800	0.95984600
O	2.69496900	-1.07760500	1.65961700
C	4.70230600	-1.98097300	0.67887900
F	4.73707300	-2.25514300	-0.62943500
F	5.91894800	-1.57844300	1.05809200
F	4.39467900	-3.09124800	1.33958200
H	3.24066800	0.88441200	0.43732700
H	-1.61817800	-1.51393100	-2.68783300

7. Optimization of the substitution reaction

7.1 Optimization of the reaction conditions: effect of bases

1-(4-Aminophenyl)ethan-1-one (6.8 mg, 0.05 mmol, 1 equiv), base (0.06 mmol, 1.2 equiv), 3-chloro-1,1,1,2-tetrafluoropropan-2-yl)(4-fluorophenyl)iodonium trifluoromethanesulfonate (28.6 mg, 0.055 mmol, 1.1 equiv) dissolved in DCM (0.5 mL). The mixture was stirred at ambient temperature for one hour. A sample was taken and analyzed by GC-MS.

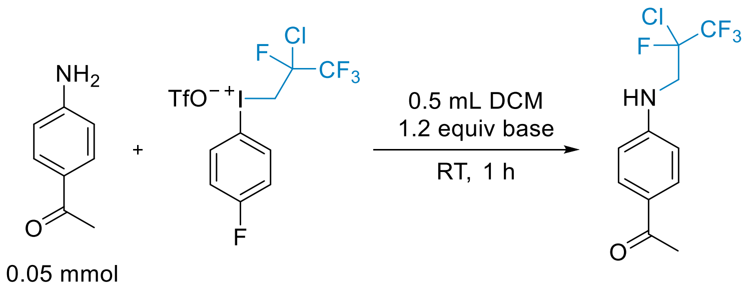

10 **33**

Entry	Base	Conversion
1	-	68%
2	NaO^{t}Bu	16%
3	K_3PO_4	62%
4	Cs_2CO_3	68%
5	Na_2CO_3	70%
6	K_2CO_3	75%
7	dtBuPy	78%
**8**	**Li_2CO_3**	**85%**

7.2 Optimization of the reaction conditions: effect of solvents

1-(4-Aminophenyl)ethan-1-one (6.8 mg, 0.05 mmol, 1 equiv), Li_2CO_3 (4.4 mg, 0.06 mmol, 1.2 equiv), 3-chloro-1,1,1,2-tetrafluoropropan-2-yl)(4-fluorophenyl)iodonium trifluoromethanesulfonate (28.6 mg, 0.055 mmol, 1.1 equiv) dissolved in the proper solvent (0.5 mL). The mixture was stirred at ambient temperature for one hour. A sample was taken and analyzed by GC-MS.

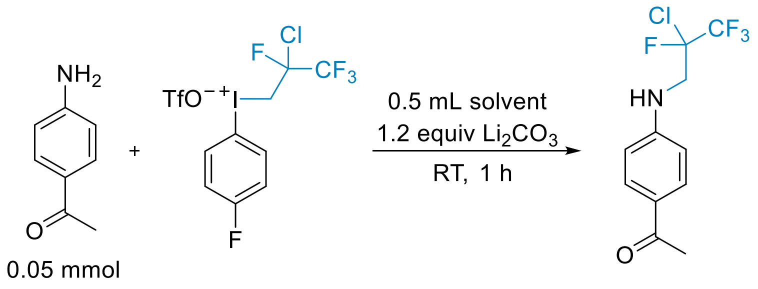

10 **33**

Entry	Solvent	Conversion
1	DMF	0%
2	Toluene	48%
3	EtOAc	78%
4	DCM	85%
5	THF	87%
**6**	**MeCN**	**100%**

8. Synthesis of starting materials

2-(Hex-1-yn-1-yl)aniline

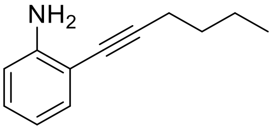

To a mixture of 2-iodoaniline (657.1 mg, 3.0 mmol), bis(triphenylphosphine)-palladium(II) chloride (210.6 mg, 0.3 mmol), CuI (57.1 mg, 0.3 mmol) in 1.2mL of triethylamine and THF (5.5 mL) was added hex-1-yne (1.1 g, 16.0 mmol). The mixture was stirred at room temperature under argon atmosphere for 20 h. Solvent was removed in vacuo, and the residue was taken up in 100 mL of diethyl ether. The mixture was filtered through Celite, and the filtrate was dried over Na_2SO_4. The crude product was purified by flash column chromatography using hexane and ethyl acetate as eluents.[14]

Yield: 370.4 mg (2.14 mmol, 71%) yellow oil. **R_f**= 0.39 in hexane : ethyl acetate 10:1. **^{1}H NMR** (250 MHz, Chloroform-*d*) δ 7.24 (dd, *J* = 7.7, 1.6 Hz, 1H), 7.06 (td, *J* = 7.7, 1.6 Hz, 1H), 6.77 – 6.50 (m, 2H), 4.09 (s, 2H), 2.46 (t, *J* = 6.8 Hz, 2H), 1.73 – 1.30 (m, 4H), 0.95 (t, *J* = 7.1 Hz, 3H). **^{13}C NMR** (63 MHz, Chloroform-*d*) δ 147.7, 132.0, 128.8, 117.9, 114.2, 109.0, 95.8, 77.1, 31.1, 22.1, 19.4, 13.7. **MS** (EI, 70 eV): m/z (%): 174 (11), 173 (73, [M^+]), 172 (8), 158 (17), 144 (49), 143 (19), 131 (15), 130 (100), 128 (10), 117 (12), 115 (10), 103 (22), 102 (9), 89 (7), 77 (25). Spectral data is in accordance with data given in literature.[14]

3-((Tert-butyldimethylsilyl)oxy)aniline

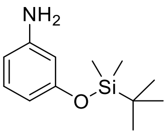

To a solution of 1.5 equiv imidazole (306.4 mg, 4.5 mmol) and 3-aminophenol (327.4 mg, 3.0 mmol) in dry THF (12 mL, freshly distilled) was added 1.3 equiv tertbutyldimethylsilyl chloride (587.8 mg, 3.9 mmol). The mixture was stirred at room temperature under argon atmosphere. Monitored by TLC. After completion (approximately 20 hours), the reaction mixture was poured into distilled water (20 mL), extracted with diethyl ether (3×30 mL). The combined organic layer was dried over Na_2SO_4, filtered. The solvent was evaporated under reduced pressure and the crude product was purified by flash column chromatography using hexane and ethyl acetate as eluents.[15]

Yield: 575.6 mg (2.78 mmol, 93%) yellow oil. **R_f**= 0.44 in hexane : ethyl acetate 4:1. **^{1}H NMR** (250 MHz, Chloroform-*d*) δ 7.02 (t, *J* = 8.0 Hz, 1H), 6.35 – 6.25 (m, 2H), 6.22 (t, *J* = 2.3 Hz, 1H), 3.59 (s, 2H), 1.02 (s, 9H), 0.22 (s, 6H). **^{13}C NMR** (63 MHz, Chloroform-*d*) δ 156.7, 147.7, 130.0, 110.5, 108.6, 107.2, 25.8, 18.2, -4.3. **MS** (EI, 70 eV): m/z (%): 224 (4), 223 (23, [M^+]), 168 (5), 167 (21), 166 (100), 150 (3), 149 (5), 136 (2), 106 (3), 92 (4), 83 (3), 75 (5), 74 (4). Spectral data is in accordance with data given in literature.[15]

5-Phenyl-1*H*-tetrazole

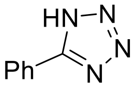

To a mixture of sodium azide (234.0 mg, 3.6 mmol), ammonium chloride (160.5 mg, 3.0 mmol) and DMF (6.0 mL) was added benzonitrile (309.4 mg, 306 µL, 3.0 mmol). The mixture was stirred for 24 h at 110 °C. After that, the reaction mixture was cooled to room temperature, and dissolved in 1.2 mL of NaOH aqueous solution (5 M) with 30 min stirring. Solvent was removed in vacuo, and the residue was taken up in 3 mL of water. After added 3 mL HCl aqueous solution (3M). The resulting white precipitate was filtered and washed with 3 mL HCl aqueous solution (3M) two times and dried.[16]

Yield: 388.2 mg (2.66 mmol, 89%) white solid. **R_f**= 0.14 in ethyl acetate. **^{1}H NMR** (250 MHz, DMSO-d_6) δ 8.11 – 8.00 (m, 2H), 7.65 – 7.51 (m, 3H). **^{13}C NMR** (63 MHz, DMSO-d_6) δ 155.3, 131.3, 129.4, 127.0,

124.2. **MS** (EI, 70 eV): m/z (%): 146 (18, [M^+]), 119 (10), 118 (100), 103 (32), 91 (41), 89 (15), 77 (32), 76 (14), 63 (24), 51 (14). Spectral data is in accordance with data given in literature.[16]

3-Chloro-1,1,1,2-tetrafluoro-2-iodopropane (2)

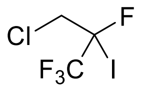

A 250 mL pressure flask was tared with the Teflon bottlecap and stirring bar. The flask was evacuated and refilled with HFO gas three times. 2,3,3,3-Tetrafluoroprop-1-ene (HFO-1234yf) gas was condensated and filled the flask about half (155.52 g, 1.364 mol) using liquid nitrogen bath then closed with the bottlecap. After the flask warmed up to room temperature the weight was measured and the required ICl amount was calculated. The flask was cooled down again using liquid nitrogen bath and the ICl (56.7 mL, 183.8 g, 1.132 mol) was added. The pressure flask was heated up to 50 °C using an oil bath for 3 hours. The reaction mixture cooled down until the next day to room temperature. The flask was cooled down again using liquid nitrogen bath then the bottlecap has been removed and the mixture was allowed to warm to room temperature. The dark oil was washed three times with 1:1 mixture of saturated $NaHCO_3$ and Na_2SO_3 (using no organic solvent). The organic phase was washed twice with 50 mL cc. NaCl and dried by pushing through a plug of $MgSO_4$ using positive pressure. The product was stored in a dark vial on copper shavings and refrigerated.

The addition reaction produces 5-7% regioisomer.

Yield: 217.5 g (0.787 mol, 70%) colorless oil. **^{1}H NMR** (400 MHz, DMSO-d_6) δ 4.55 (dd, *J* = 19.0, 13.5 Hz, 1H), 4.39 (dd, *J* = 19.5, 13.5 Hz, 1H). **^{19}F NMR** (376 MHz, DMSO-d_6) δ -74.7 (d, *J* = 11.8 Hz), -139.0 (q, *J* = 11.8 Hz). **^{13}C NMR** (101 MHz, DMSO-d_6) δ 121.5 (qd, *J* = 283.2, 29.2 Hz), 78.1 (dq, *J* = 255.6, 34.4 Hz), 48.4 (d, *J* = 22.1 Hz). **MS** (EI, 70 eV): m/z (%): 278 (13), 276 (41, [M^+]), 177 (10), 162 (12), 151 (32), 149 (100), 148 (12), 127 (33), 113 (9), 95 (15), 69 (41). Spectral data is in accordance with data given in literature.[17]

Regioisomer (minor component): **^{1}H NMR** (400 MHz, DMSO-d_6) δ 4.16 (dd, *J* = 12.5, 7.7 Hz, 1H), 4.04 (dd, *J* = 30.6, 12.5 Hz, 1H). **^{19}F NMR** (376 MHz, DMSO-d_6) δ -80.0 (d, *J* = 6.5 Hz), -117.5 (q, *J* = 6.5 Hz). **^{13}C NMR** (101 MHz, DMSO-d_6) δ 118.5 (qd, *J* = 285.4, 32.7 Hz), 105.5 (dq, *J* = 251.2, 36.1 Hz), 3.7 (d, *J* = 23.4 Hz). Spectral data is in accordance with data given in literature.[17]

9. Synthesis of iodonium salts

General procedures

A screwed cap vial with a stirrer bar was evacuated and refilled with argon three times. Trifluoroacetic anhydride (9.661 g, 6.5 mL, 46 mmol) and catalytic amount of trifluoroacetic acid (0.144 g, 0.1 mL, 1 mmol) was added through syringe. The mixture was cooled to -10 °C, then hydrogen-peroxide (50 w/w% in water) (0.748 g, 0.65 mL, 11 mmol) was added dropwise within two minutes. 3-Chloro-1,1,1,2-tetrafluoro-2-iodopropane (2.764 g, 1.3 mL, 10 mmol) was added dropwise through syringe. The resulting reaction mixture was stirred for 16 hours at 35 °C. After that, the mixture was cooled to -20 °C, then freshly distilled dichloromethane (6.5 mL) was added to the mixture. 10.5 mmol arene was added dropwise to the reaction mixture, followed by the addition of trifluoromethanesulfonic acid (1.501 g, 0.9 mL, 10 mmol). The reaction mixture was kept between 0 °C and 4 °C for 16 hours. After that, all volatiles were removed under reduced pressure at 0 °C protected from light. The dark oil was shaken with cold (-20 °C) diethyl ether, getting white precipitate. The suspension was kept at -20 °C for 2 hours, then the white precipitate was filtered and washed with cold diethyl ether three times.

(2-Chloro-2,3,3,3-tetrafluoropropyl)(4-fluorophenyl)iodonium trifluoromethanesulfonate (10)

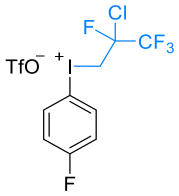

Using fluorobenzene (1.009 g, 1.0 mL, 10.5 mmol).

Yield: 2201 mg (4.23 mmol, 42%) white solid. Stable at room temperature for at least four months. **Mp.** 129-130 °C. **MS** (EI, 70 eV): m/z (%): Compound decomposes in injector. **^{1}H NMR** (400 MHz, Acetonitrile-*d*$_3$) δ 8.22 (dd, *J* = 8.9, 4.8 Hz, 2H), 7.33 (t, *J* = 8.8 Hz, 2H), 5.09 (dd, *J* = 13.1, 8.7 Hz, 1H), 4.93 (dd, *J* = 28.1, 13.2 Hz, 1H). **^{19}F NMR** (376 MHz, Acetonitrile-*d*$_3$) δ -79.3, -80.9 (d, *J* = 6.2 Hz), -104.9, -118.3 (q, *J* = 6.2 Hz). **^{13}C NMR** (101 MHz, Acetonitrile-*d*$_3$) δ 166.6 (d, *J* = 254.8 Hz), 141.4 (d, *J* = 9.6 Hz), 121.7 (q, *J* = 320.0 Hz), 120.8 (d, *J* = 23.6 Hz), 119.9 (qd, *J* = 285.5, 31.3 Hz), 104.7 (dq, *J* = 254.7, 38.4 Hz), 103.8 (d, *J* = 3.3 Hz), 41.7 (d, *J* = 21.9 Hz). **IR** (solid, ATR) 1581, 1484, 1275, 1231, 1182, 1163, 1126, 1022, 1003, 929, 832, 813 cm^{-1}. **HRMS** (ESI) [M - OTf]$^+$ calculated for $C_9H_6ClF_5I^+$: 370.9123, found: 370.9120.

Bis(4-fluorophenyl)iodonium trifluoromethanesulfonate (6)

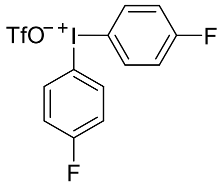

Side product, white solid. **Mp.** 158-160 °C. **MS** (EI, 70 eV): m/z (%): Compound decomposes in injector. **^{1}H NMR** (250 MHz, Acetonitrile-*d*$_3$) δ 8.26 – 7.93 (m, 4H), 7.37 – 7.11 (m, 4H). **^{19}F NMR** (235 MHz, Acetonitrile-*d*$_3$) δ -79.2, -106.3. **^{13}C NMR** (63 MHz, Acetonitrile-*d*$_3$) δ 166.1 (d, *J* = 253.7 Hz), 139.3 (d, *J* = 9.4 Hz), 121.8 (q, *J* = 320.3 Hz), 120.7 (d, *J* = 23.4 Hz), 108.7 (d, *J* = 3.2 Hz). **IR** (solid, ATR) 1577, 1480, 1402, 1268, 1246, 1219, 1163, 1026, 1007, 932, 839, 824, 757 cm^{-1}. **HRMS** (ESI) [M - OTf]$^+$calculated for $C_{12}H_8F_2I^+$: 316.9639, found: 316.9636. Spectral data is in accordance with data given in literature.[18]

(2-Chloro-2,3,3,3-tetrafluoropropyl)(4-chlorophenyl)iodonium trifluoromethanesulfonate (10-Cl)

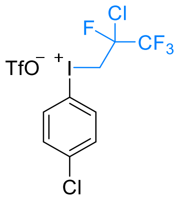

Using chlorobenzene (1.181 g, 1.1 mL, 10.5 mmol).

Yield: 1935 mg (3.60 mmol, 36%) white solid. **Mp.** 121-122 °C. **MS** (EI, 70 eV): m/z (%): Compound decomposes in injector. **^{1}H NMR** (400 MHz, Acetonitrile-*d*$_3$) δ 8.16 (d, *J* = 8.8 Hz, 2H), 7.58 (d, *J* = 8.8 Hz, 2H), 5.10 (dd, *J* = 13.2, 8.5 Hz, 1H), 4.94 (dd, *J* = 28.2,

13.2 Hz, 1H). **^{19}F NMR** (376 MHz, Acetonitrile-*d*$_3$) δ -79.3, -80.9 (d, *J* = 6.3 Hz), -118.2 (q, *J* = 6.1 Hz). **^{13}C NMR** (101 MHz, Acetonitrile-*d*$_3$) δ 140.9, 139.8, 133.2, 121.5 (q, *J* = 319.9 Hz), 119.9 (qd, *J* = 285.4, 31.1 Hz), 107.6, 104.5 (dq, *J* = 254.5, 38.3 Hz), 41.5 (d, *J* = 21.8 Hz). **IR** (solid, ATR) 1272, 1223, 1186 ,1171, 1126, 1093, 1018, 999, 966, 932, 820, 735 cm^{-1}. **HRMS** (ESI) [M - OTf]$^+$ calculated for $C_9H_6Cl_2F_4I^+$: 386.8827, found: 386.8824.

(2-Chloro-2,3,3,3-tetrafluoropropyl)(phenyl)iodonium trifluoromethanesulfonate (10-H)

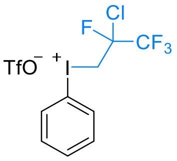

Using benzene (0.820 g, 0.94 mL, 10.5 mmol).

Yield: 1795 mg (3.60 mmol, 36%) white solid. **Mp.** 121-122 °C. **MS** (EI, 70 eV): m/z (%): Compound decomposes in injector. **^{1}H NMR** (400 MHz, Acetonitrile-*d*$_3$) δ 8.19 (d, *J* = 8.1 Hz, 2H), 7.79 (t, *J* = 7.5 Hz, 1H), 7.58 (t, *J* = 7.9 Hz, 2H), 5.10 (dd, *J* = 13.2, 8.5 Hz, 1H), 4.95 (dd, *J* = 28.3, 13.2 Hz, 1H). **^{19}F NMR** (376 MHz, Acetonitrile-*d*$_3$) δ -79.3, -80.9 (d, *J* = 6.0 Hz), -118.3 (q, *J* = 6.1 Hz). **^{13}C NMR** (101 MHz, Acetonitrile-*d*$_3$) δ 138.1, 134.5, 133.1, 121.6 (q, *J* = 320.0 Hz), 119.9 (qd, *J* = 285.5, 31.8 Hz), 110.4, 104.5 (dq, *J* = 254.5, 38.4 Hz), 41.0 (d, *J* = 21.9 Hz). **IR** (solid, ATR) 1275, 1219, 1186, 1167, 1141, 1126, 1022, 999, 929, 735, 679 cm^{-1}. **HRMS** (ESI) [M - OTf]$^+$ calculated for $C_9H_7ClF_4I^+$: 352.9212, found: 352.9214.

(2-Chloro-2,3,3,3-tetrafluoropropyl)(mesityl)iodonium trifluoromethanesulfonate (10-Mes)

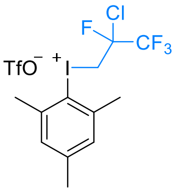

Using mesitylene (1.262 g, 1.5 mL, 10.5 mmol).

Yield: 1368 mg (2.51 mmol, 25%) white solid. **Mp.** 108-109 °C. **MS** (EI, 70 eV): m/z (%): Compound decomposes in injector. **^{1}H NMR** (400 MHz, Acetonitrile-*d*$_3$) δ 7.24 (s, 2H), 4.90 (dd, *J* = 12.9, 7.7 Hz, 1H), 4.80 (dd, *J* = 29.8, 12.9 Hz, 1H), 2.65 (s, 6H), 2.37 (s, 3H). **^{19}F NMR** (376 MHz, Acetonitrile-*d*$_3$) δ -79.3, -80.9 (d, *J* = 6.1 Hz), -118.1 (q, *J* = 6.2 Hz). **^{13}C NMR** (101 MHz, Acetonitrile-*d*$_3$) δ 146.2, 144.3, 131.1, 121.7 (q, *J* = 320.0 Hz), 119.8 (qd, *J* = 285.4, 31.3 Hz), 118.2, 104.5 (dq, *J* = 254.0, 38.5 Hz), 39.1 (d, *J* = 22.1 Hz), 27.4 (d, *J* = 1.2 Hz), 21.0. **IR** (solid, ATR) 1592, 1287, 1257, 1216, 1182, 1163, 1134, 1026, 1011, 929, 850, 735 cm^{-1}. **HRMS** (ESI) [M - OTf]$^+$ calculated for $C_{12}H_{13}ClF_4I^+$: 394.9681, found: 394.9683.

10. Substitution on a larger scale in 1 mmol

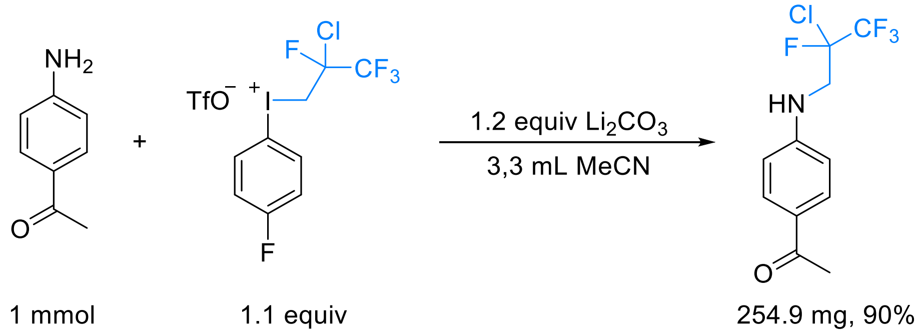

1-(4-Aminophenyl)ethan-1-one (135.2 mg, 1.0 mmol, 1 equiv), Li_2CO_3 (88.7 mg, 1.2 mmol, 1.2 equiv), 3-chloro-1,1,1,2-tetrafluoropropan-2-yl)(4-fluorophenyl)iodonium trifluoromethanesulfonate (572.6 mg, 1.1 mmol, 1.1 equiv) dissolved in MeCN (3.3 mL). The mixture was stirred at ambient temperature for four hours, then evaporated to Celite under reduced pressure and the crude product

was purified by column chromatography using hexanes-ethyl acetate as eluent. Yield: 254.9 mg (0.90 mmol, 90%), white solid. (Characterization: see in 0.3 mmol scale in the chapter of 1-(4-((2-chloro-2,3,3,3-tetrafluoropropyl)amino)phenyl)ethan-1-one.)

11. Substitution reactions with iodonium salts

Method A (for liquid state amines) for substitution

Li_2CO_3 (26.6 mg, 0.36 mmol), 3-chloro-1,1,1,2-tetrafluoropropan-2-yl)(4-fluorophenyl)iodonium trifluoromethanesulfonate (171.8 mg, 0.33 mmol) dissolved in MeCN (1.0 mL) and finally the amine (0.3 mmol) was measured by Hamilton glass syringe. The mixture was stirred at the indicated temperature for the indicated time, monitored by GC-MS and TLC, using hexanes-ethyl acetate as eluent. The solvent was evaporated under reduced pressure to Celite and the crude product was purified by column chromatography using hexanes-ethyl acetate as eluent.

Method B (for solid state amines) for substitution

Li_2CO_3 (26.6 mg, 0.36 mmol), amine (0.3 mmol), 3-chloro-1,1,1,2-tetrafluoropropan-2-yl)(4-fluorophenyl)iodonium trifluoromethanesulfonate (171.8 mg, 0.33 mmol) dissolved in MeCN (1.0 mL). The mixture was stirred at the indicated temperature for the indicated time, monitored by GC-MS and TLC, using hexanes-ethyl acetate as eluent. The solvent was evaporated to Celite under reduced pressure and the crude product was purified by column chromatography using hexanes-ethyl acetate as eluent.

N-(2-Chloro-2,3,3,3-tetrafluoropropyl)aniline (15)

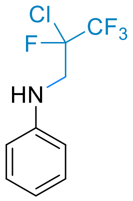

Method A. Using aniline (27.9 mg, 27.3 µL, 0.30 mmol), Li_2CO_3 (26.6 mg, 0.36 mmol) and iodonium salt (171.8 mg, 0.33 mmol) in MeCN (1.0 mL) (RT, 2 h).

Yield: 64.0 mg (0.26 mmol, 88%) yellow oil. **R_f**= 0.50 in hexane : ethyl acetate 4:1. **MS** (EI, 70 eV): m/z (%): 243 (4), 241 (14, [M^+]), 107 (8), 106 (100), 79 (7), 77 (19), 69 (3), 65 (8), 51 (9). **^{1}H NMR** (250 MHz, Chloroform-*d*) δ 7.13 (t, *J* = 7.9 Hz, 2H), 6.72 (t, *J* = 7.3 Hz, 1H), 6.62 (d, *J* = 8.0 Hz, 2H), 3.93 (s, 1H), 3.90 (dd, *J* = 15.3, 12.3 Hz, 1H), 3.75 (dd, *J* = 21.2, 15.3 Hz, 1H). **^{19}F NMR** (235 MHz, Chloroform-*d*) δ -80.6 (d, *J* = 6.1 Hz), -130.2 (q, *J* = 6.2 Hz). **^{13}C NMR** (63 MHz, Chloroform-*d*) δ 146.3, 129.6, 120.7 (qd, *J* = 284.9, 31.0 Hz), 119.4, 113.5, 107.0 (dq, *J* = 255.2, 34.5 Hz), 49.1 (d, *J* = 22.2 Hz). **IR** (film, ATR) 1605, 1516, 1499, 1307, 1258, 1214, 1193, 1150, 1115, 1105, 1077, 1057, 1027, 994, 971, 945, 751, 692 cm^{-1}. **HRMS** (EI) $[M]^+$ calculated for $C_9H_8ClF_4N^+$: 241.0281, found: 241.0276.

N-(2-Chloro-2,3,3,3-tetrafluoropropyl)-2-methylaniline (16)

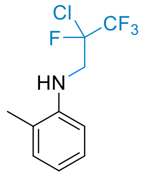

Method A. Using o-toluidine (32.2 mg, 31.9 µL, 0.30 mmol), Li_2CO_3 (26.6 mg, 0.36 mmol) and iodonium salt (171.8 mg, 0.33 mmol) in MeCN (1.0 mL) (RT, 3 h).

Yield: 66.5 mg (0.26 mmol, 87%) yellow oil. **R_f**= 0.61 in hexane : ethyl acetate 10:1. **MS** (EI, 70 eV): m/z (%): 257 (6), 255 (19, [M^+]), 220 (3), 121 (9), 120 (100), 119 (7), 91 (17), 77 (7), 69 (2), 65 (6). **^{1}H NMR** (250 MHz, Chloroform-*d*) δ 7.26 – 7.05 (m, 2H), 6.90 – 6.66 (m, 2H), 4.10 (dd, *J* = 15.1, 12.3 Hz, 1H), 3.94 (dd, *J* = 21.1, 15.0 Hz, 1H), 3.90 (s, 1H), 2.25 (s, 3H). **^{19}F NMR** (235 MHz, Chloroform-*d*) δ -80.6 (d, *J* = 6.2 Hz), -130.1 (q, *J* = 6.2 Hz). **^{13}C NMR** (63 MHz, Chloroform-*d*) δ 144.3, 130.7, 127.3, 122.8, 120.8 (qd, *J* = 284.6, 31.1 Hz), 119.0, 110.6 (d, *J* = 1.8 Hz), 107.0 (dq, *J* = 255.4, 34.8 Hz), 48.8 (d, *J* = 22.1 Hz), 17.5. **IR** (film, ATR) 1609, 1590, 1521, 1482, 1456, 1378, 1305, 1262, 1212, 1187, 1167, 1154, 1133, 1068, 1044, 971, 945, 926, 895, 844, 822, 746, 731, 714, 660 cm^{-1}. **HRMS** (EI) $[M]^+$ calculated for $C_{10}H_{10}ClF_4N^+$: 255.0438, found: 255.0435.

2-Chloro-N-(2-chloro-2,3,3,3-tetrafluoropropyl)aniline (17)

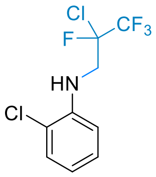

Method A. Using 2-chloroaniline (38.3 mg, 31.6 µL 0.30 mmol), Li_2CO_3 (26.6 mg, 0.36 mmol) and iodonium salt (171.8 mg, 0.33 mmol) in MeCN (1.0 mL) (RT, 2 h).

Yield: 65.0 mg (0.24 mmol, 78%) yellowish oil. **R_f**= 0.82 in hexane : ethyl acetate 4:1. **MS** (EI, 70 eV): m/z (%): 277 (9), 275 (15, [M^+]), 240 (2), 205 (2), 142 (31), 141 (9), 140 (100), 99 (7), 77 (22), 75 (8), 69 (5). **^{1}H NMR** (250 MHz, Chloroform-*d*) δ 7.31 (dd, *J* = 7.9, 1.6 Hz, 1H), 7.18 (t, *J* = 7.8 Hz, 1H), 6.92 – 6.57 (m, 2H), 4.78 (bs, 1H), 4.08 (t, *J* = 13.9 Hz, 1H), 3.92 (dd, *J* = 21.0, 15.4 Hz, 1H). **^{19}F NMR** (235 MHz, Chloroform-*d*) δ -80.6 (d, *J* = 6.1 Hz), -130.2 (q, *J* = 6.2 Hz). **^{13}C NMR** (63 MHz, Chloroform-*d*) δ 142.5, 129.7, 128.0, 120.7 (qd, *J* = 284.9, 31.1 Hz), 119.9, 119.2, 111.8 (d, *J* = 2.2 Hz), 106.8 (dq, *J* = 256.0, 34.6 Hz), 48.6 (d, *J* = 22.3 Hz). **IR** (film, ATR) 1599, 1519, 1465, 1445, 1378, 1327, 1307, 1292, 1271, 1243, 1215, 1191, 1169, 1152, 1133, 1107, 1066, 1035, 975, 947, 928, 897, 742, 686, 660 cm^{-1}. **HRMS** (EI) $[M]^+$ calculated for $C_9H_7Cl_2F_4N^+$: 274.9892, found: 274.9887.

N-(2-Chloro-2,3,3,3-tetrafluoropropyl)-2-iodoaniline (18)

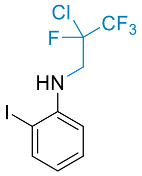

Method B. Using 2-iodoaniline (65.7 mg, 0.30 mmol), Li_2CO_3 (26.6 mg, 0.36 mmol) and iodonium salt (171.8 mg, 0.33 mmol) in MeCN (1.0 mL) (RT, 6 h).

Yield: 99.8 mg (0.27 mmol, 91%) colorless oil. **R_f**= 0.70 in hexane : ethyl acetate 4:1. **MS** (EI, 70 eV): m/z (%): 369 (12), 367 (37, [M^+]), 233 (7), 232 (100), 205 (5), 136 (10), 135 (12), 127 (10), 105 (10), 104 (26), 91 (13), 78 (11), 77 (12), 59 (9), 64 (10), 63 (10). **^{1}H NMR** (250 MHz, Chloroform-*d*) δ 7.63 (dd, *J* = 7.8, 1.5 Hz, 1H), 7.24 – 7.01 (m, 1H), 6.63 (d, *J* = 8.2 Hz, 1H), 6.47 (td, *J* = 7.6, 1.4 Hz, 1H), 4.54 (bs, 1H), 3.99 (dd, *J* = 15.3, 12.1 Hz, 1H), 3.83 (dd, *J* = 21.1, 15.3 Hz, 1H). **^{19}F NMR** (235 MHz, Chloroform-*d*) δ -80.5 (d, *J* = 6.1 Hz), -130.1 (q, *J* = 6.1 Hz). **^{13}C NMR** (63 MHz, Chloroform-*d*) δ 145.7, 139.6, 129.6, 120.7 (qd, *J* = 285.0, 30.9 Hz), 120.6, 111.2 (d, *J* = 2.3 Hz), 106.7 (dq, *J* = 255.8, 35.0 Hz), 86.0, 49.0 (d, *J* = 22.3 Hz). **IR** (film, ATR) 1592, 1516, 1456, 1443, 1435, 1307, 1290, 1266, 1242, 1214, 1191, 1171, 1154, 1135, 1098, 1064, 1042, 1008, 973, 947, 895, 744, 733, 703, 664 cm^{-1}. **HRMS** (EI) $[M]^+$ calculated for $C_9H_7ClF_4IN^+$: 366.9248, found: 366.9237.

N-(2-Chloro-2,3,3,3-tetrafluoropropyl)-[1,1'-biphenyl]-2-amine (19)

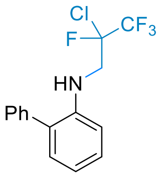

Method B. Using [1,1'-biphenyl]-2-amine (50.8 mg, 0.30 mmol), Li_2CO_3 (26.6 mg, 0.36 mmol) and iodonium salt (171.8 mg, 0.33 mmol) in MeCN (1.0 mL) (RT, 6 h).

Yield: 66.9 mg (0.21 mmol, 70%) yellow oil. $\mathbf{R_f}$= 0.78 in hexane : ethyl acetate 4:1. **MS** (EI, 70 eV): m/z (%): 319 (6), 317 (18, [M^+]), 183 (15), 182 (100), 181 (11), 180 (28), 167 (23), 166 (10), 165 (26), 152 (13), 90 (7), 84 (12), 69 (2). **^{1}H NMR** (250 MHz, Chloroform-*d*) δ 7.48 – 7.22 (m, 5H), 7.17 (td, *J* = 7.8, 1.7 Hz, 1H), 7.04 (dd, *J* = 7.5, 1.7 Hz, 1H), 6.83 – 6.61 (m, 2H), 3.89 (s, 1H), 3.87 (dd, *J* = 15.4, 12.4 Hz, 1H), 3.71 (dd, *J* = 21.0, 15.3 Hz, 1H). **^{19}F NMR** (235 MHz, Chloroform-*d*) δ -80.6 (d, *J* = 6.2 Hz), -129.9 (q, *J* = 6.1 Hz). **^{13}C NMR** (63 MHz, Chloroform-*d*) δ 143.3, 138.9, 130.7, 129.5, 129.2, 128.8, 128.6, 127.7, 120.7 (qd, *J* = 285.0, 31.0 Hz), 118.8, 110.8 (d, *J* = 2.1 Hz), 107.1 (dq, *J* = 255.7, 34.6 Hz), 48.8 (d, *J* = 22.3 Hz). **IR** (film, ATR) 1586, 1517, 1493, 1465, 1452, 1439, 1307, 1284, 1273, 1214, 1191, 1169, 1154, 1130, 1091, 1064, 1048, 1008, 971, 943, 895, 770, 748, 733, 705 cm^{-1}. **HRMS** (EI) $[M]^+$ calculated for $C_{15}H_{12}ClF_4N^+$: 317.0594, found: 317.0591.

2-Benzyl-*N*-(2-chloro-2,3,3,3-tetrafluoropropyl)aniline (20)

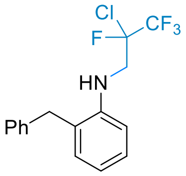

Method B. Using 2-benzylaniline (55.0 mg, 0.30 mmol), Li_2CO_3 (26.6 mg, 0.36 mmol) and iodonium salt (171.8 mg, 0.33 mmol) in MeCN (1.0 mL) (RT, 5 h).

Yield: 96.8 mg (0.29 mmol, 97%) white solid. **Mp.** 84-86 °C. $\mathbf{R_f}$= 0.58 in hexane : ethyl acetate 4:1. **MS** (EI, 70 eV): m/z (%): 333 (7), 331 (21, [M^+]), 197 (15), 196 (100), 182 (11), 180 (12), 165 (12), 118 (31), 117 (7), 91 (37). **^{1}H NMR** (250 MHz, Chloroform-*d*) δ 7.25 – 7.01 (m, 7H), 6.77 (t, *J* = 7.4 Hz, 1H), 6.64 (d, *J* = 8.1 Hz, 1H), 3.88 (s, 2H), 3.82 – 3.57 (m, 3H). **^{19}F NMR** (235 MHz, Chloroform-*d*) δ -80.6 (d, *J* = 6.2 Hz), -130.0 (q, *J* = 6.2 Hz). **^{13}C NMR** (63 MHz, Chloroform-*d*) δ 144.5, 139.0, 131.3, 129.0, 128.6, 128.0, 126.8, 125.8, 120.7 (qd, *J* = 285.0, 31.0 Hz), 119.1, 111.4 (d, *J* = 1.8 Hz), 106.8 (dq, *J* = 255.4, 34.9 Hz), 48.5 (d, *J* = 22.4 Hz), 38.5. **IR** (film, ATR) 1512, 1493, 1454, 1297, 1223, 1193, 1152, 1143, 1128, 1076, 1066, 1048, 969, 953, 755 cm^{-1}. **HRMS** (ESI) $[M+H]^+$ calculated for $C_{16}H_{15}ClF_4N^+$: 332.0824, found: 332.0824.

N-(2-Chloro-2,3,3,3-tetrafluoropropyl)-2-(hex-1-yn-1-yl)aniline (21)

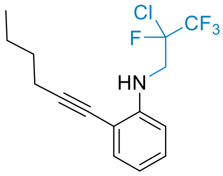

Method A. Using 2-(hex-1-yn-1-yl)aniline (52.0 mg, 54.6 µL, 0.30 mmol), Li_2CO_3 (26.6 mg, 0.36 mmol) and iodonium salt (171.8 mg, 0.33 mmol) in MeCN (1.0 mL) (RT, 2 h).

Yield: 86.0 mg (0.27 mmol, 89%) yellow oil. $\mathbf{R_f}$= 0.61 in hexane : ethyl acetate 10:1. **MS** (EI, 70 eV): m/z (%): 323 (14), 321 (41, [M^+]), 187 (8), 186 (54), 172 (7), 157 (15), 156 (19), 144 (43), 143 (47), 131 (12), 130 (100), 128 (13), 118 (31), 117 (15), 116 (12), 115 (29), 102 (7), 91 (1), 89 (7), 77 (10), 69 (11). **^{1}H NMR** (250 MHz, Chloroform-*d*) δ 7.32 (dd, *J* = 7.5, 1.6 Hz, 1H), 7.20 (td, *J* = 7.8, 1.6 Hz, 1H), 6.81 – 6.59 (m, 2H), 4.94 (bs, 1H), 4.08 (dd, *J* = 15.3, 12.5 Hz, 1H), 3.93 (dd, *J* = 20.6, 15.2 Hz, 1H), 2.52 (t, *J* = 6.8 Hz, 2H), 1.78 – 1.38 (m, 4H), 0.99 (t, *J* = 7.1 Hz, 3H). **^{19}F NMR** (235 MHz, Chloroform-*d*) δ -80.6 (d, *J* = 6.2 Hz), -130.3 (q, *J* = 6.1 Hz). **^{13}C NMR** (63 MHz, Chloroform-*d*) δ 147.1, 132.2, 129.1, 120.8 (qd, *J* = 284.9, 31.0 Hz), 118.2, 109.8, 109.8, 106.9 (dq, *J* = 255.6, 34.8 Hz), 97.0, 76.5, 48.6 (d, *J* = 22.5 Hz), 31.0, 22.1, 19.4, 13.7. **IR** (film, ATR) 2962, 2936, 1603, 1581, 1516, 1460, 1327, 1307, 1284, 1269, 1215, 1191, 1167, 1154, 1132, 1087, 1066, 1040, 973, 947, 895, 746, 731, 686, 662 cm^{-1}. **HRMS** (ESI) $[M+H]^+$ calculated for $C_{15}H_{17}ClF_4N^+$: 322.0980, found: 322.0982.

2-((2-Chloro-2,3,3,3-tetrafluoropropyl)amino)phenol (22)

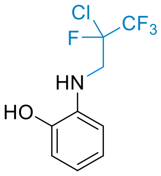

Method B. Using 2-aminophenol (32.7 mg, 0.30 mmol), Li_2CO_3 (26.6 mg, 0.36 mmol) and iodonium salt (171.8 mg, 0.33 mmol) in MeCN (1.0 mL) (RT, 3 h).

Yield: 62.0 mg (0.24 mmol, 80%) green oil. **R$_f$**= 0.34 in hexane : ethyl acetate 4:1. **MS** (EI, 70 eV): m/z (%): 259 (7), 257 (20, [M$^+$]), 222 (3), 123 (8), 122 (100), 120 (12), 95 (19), 94 (10), 80 (10), 77 (16), 69 (8). **^{1}H NMR** (500 MHz, DMSO-*d*$_6$) δ 9.46 (s, 1H), 6.73 – 6.62 (m, 3H), 6.50 (td, *J* = 7.1, 2.1 Hz, 1H), 5.27 (t, *J* = 7.2 Hz, 1H), 4.18 – 3.87 (m, 2H). **^{19}F NMR** (376 MHz, DMSO-*d*$_6$) δ -79.5 (d, *J* = 6.4 Hz), -128.4 – -128.8 (m). **^{13}C NMR** (126 MHz, DMSO-*d*$_6$) δ 144.2, 135.9, 120.6 (qd, *J* = 284.9, 31.5 Hz), 119.5, 117.3, 113.8, 110.3 (d, *J* = 2.7 Hz), 107.7 (dq, *J* = 254.0, 33.3 Hz), 47.6 (d, *J* = 21.2 Hz). **IR** (film, ATR) 1611, 1518, 1454, 1376, 1305, 1268, 1249, 1190, 1152, 1119, 1067, 1037, 973, 899, 835, 738, 660 cm^{-1}. **HRMS** (EI) [M]$^+$ calculated for $C_9H_8ClF_4NO^+$: 257.0231, found: 257.0228.

3-Chloro-*N*-(2-chloro-2,3,3,3-tetrafluoropropyl)aniline (23)

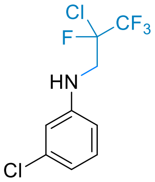

Method A. Using 3-chloroaniline (38.3 mg, 31.8 µL, 0.30 mmol), Li_2CO_3 (26.6 mg, 0.36 mmol) and iodonium salt (171.8 mg, 0.33 mmol) in MeCN (1.0 mL) (RT, 4 h).

Yield: 79.9 mg (0.29 mmol, 96%) yellow oil. **R$_f$**= 0.43 in hexane : ethyl acetate 4:1. **MS** (EI, 70 eV): m/z (%): 279 (1), 277 (8), 275 (13, [M$^+$]), 142 (32), 141 (8), 140 (100), 111 (8), 105 (7), 77(7), 75 (8), 69 (3). **^{1}H NMR** (250 MHz, Chloroform-*d*) δ 7.13 (t, *J* = 8.0 Hz, 1H), 6.79 (dd, *J* = 8.0, 1.9 Hz, 1H), 6.70 (t, *J* = 2.2 Hz, 1H), 6.59 (dd, *J* = 8.3, 2.4 Hz, 1H), 4.10 – 3.62 (m, 3H). **^{19}F NMR** (235 MHz, Chloroform-*d*) δ -80.6 (d, *J* = 6.2 Hz), -130.3 (q, *J* = 6.1 Hz). **^{13}C NMR** (63 MHz, Chloroform-*d*) δ 147.6, 135.3, 130.5, 120.7 (qd, *J* = 284.8, 31.0 Hz), 119.2, 113.3, 111.6, 106.8 (dq, *J* = 255.6, 35.0 Hz), 48.7 (d, *J* = 22.2 Hz). **IR** (film, ATR) 1601, 1514, 1486, 1441, 1428, 1376, 1325, 1307, 1284, 1269, 1242, 1214, 1191, 1152, 1135, 1113, 1094, 1063, 992, 975, 947, 906, 848, 766, 731, 680, 662 cm^{-1}. **HRMS** (EI) [M]$^+$ calculated for $C_9H_7Cl_2F_4N^+$: 274.9892, found: 274.9885.

Methyl 3-((2-Chloro-2,3,3,3-tetrafluoropropyl)amino)benzoate (24)

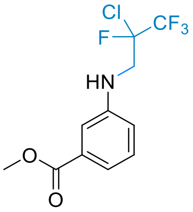

Method B. Using methyl 3-aminobenzoate (45.3 mg, 0.30 mmol), Li_2CO_3 (26.6 mg, 0.36 mmol) and iodonium salt (171.8 mg, 0.33 mmol) in MeCN (1.0 mL) (RT, 1 h).

Yield: 76.0 mg (0.25 mmol, 85%) white solid. **Mp.** 99-103 °C. **R$_f$**= 0.51 in hexane : ethyl acetate 4:1. **MS** (EI, 70 eV): m/z (%): 301 (2), 299 (8, [M$^+$]), 268 (5), 165 (10), 164 (100), 105 (7), 104 (7), 91 (4), 77 (5), 69 (2), 66 (5). **^{1}H NMR** (250 MHz, Chloroform-*d*) δ 7.47 (dd, *J* = 7.7, 1.3 Hz, 1H), 7.40 (s, 1H), 7.27 (t, *J* = 7.9 Hz, 1H), 6.90 (dd, *J* = 8.1, 2.6 Hz, 1H), 4.13 – 3.76 (m, 6H). **^{19}F NMR** (235 MHz, Chloroform-*d*) δ -80.6 (d, *J* = 6.1 Hz), -130.2 (q, *J* = 6.1 Hz). **^{13}C NMR** (63 MHz, Chloroform-*d*) δ 167.3, 146.5, 131.4, 129.5, 120.7 (qd, *J* = 284.9, 31.0 Hz), 120.3, 117.7, 114.1, 106.8 (dq, *J* = 255.4, 34.7 Hz), 52.2, 48.8 (d, *J* = 22.2 Hz). **IR** (film, ATR) 1704, 1612, 1594, 1549, 1495, 1445, 1348, 1320, 1309, 1290, 1273, 1251, 1214, 1193, 1159, 1135, 1117, 1089, 1057, 1046, 1022, 992, 975, 900, 753 cm^{-1}. **HRMS** (ESI) [M+H]$^+$ calculated for $C_{11}H_{11}ClF_4NO_2^+$: 300.0409, found: 300.0410.

3-((Tert-butyldimethylsilyl)oxy)-*N*-(2-chloro-2,3,3,3-tetrafluoropropyl)aniline (25)

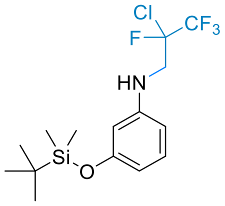

Method A. Using 3-((tert-butyldimethylsilyl)oxy)aniline (67.0 mg, 69.9 µL, 0.30 mmol), Li_2CO_3 (26.6 mg, 0.36 mmol) and iodonium salt (171.8 mg, 0.33 mmol) in MeCN (1.0 mL) (RT, 3 h).

Yield: 103,5 mg (0.28 mmol, 93%) yellow oil. $\mathbf{R_f}$= 0.73 in hexane : ethyl acetate 4:1. **MS** (EI, 70 eV): m/z (%): 373 (15), 372 (9), 371 (39, [M^+]), 356 (4), 336 (6), 314 (11), 278 (25), 258 (19), 236 (24), 218 (8), 210 (9), 209 (13), 199 (15), 198 (100), 184 (8), 182 (12), 180 (26), 178 (12), 135 (9), 93 (10), 91 (9), 90 (31), 77 (29), 75 (17), 73 (23), 59 (7), 57 (11). ^{1}**H NMR** (250 MHz, Chloroform-*d*) δ 7.07 (t, *J* = 8.1 Hz, 1H), 6.34 (dd, *J* = 8.0, 2.3 Hz, 2H), 6.24 (s, 1H), 4.19 – 3.48 (m, 3H), 1.02 (s, 9H), 0.23 (s, 6H). ^{19}**F NMR** (235 MHz, Chloroform-*d*) δ -80.6 (d, *J* = 6.1 Hz), -130.1 (q, *J* = 6.2 Hz). ^{13}**C NMR** (63 MHz, Chloroform-*d*) δ 156.9, 147.7, 130.0, 120.6 (qd, *J* = 285.0, 31.3 Hz), 111.0, 106.8 (dq, *J* = 255.1, 34.6 Hz), 106.7, 105.3, 49.0 (d, *J* = 22.3 Hz), 25.7, 18.2, -4.4. **IR** (film, ATR) 2962, 2955, 2934, 2861, 1603, 1517, 1495, 1473, 1465, 1447, 1376, 1363, 1329, 1305, 1275, 1256, 1197, 1159, 1146, 1109, 1077, 1061, 982, 964, 940, 895, 837, 781, 766, 731, 688, 665 cm^{-1}. **HRMS** (ESI) $[M+H]^+$ calculated for $C_{15}H_{23}ClF_4NOSi^+$: 372.1168, found: 372.1169.

3-((2-Chloro-2,3,3,3-tetrafluoropropyl)amino)phenol (26)

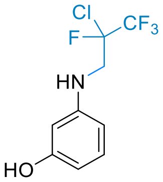

Method B. Using 3-aminophenol (32.7 mg, 0.30 mmol), Li_2CO_3 (26.6 mg, 0.36 mmol) and iodonium salt (171.8 mg, 0.33 mmol) in MeCN (1.0 mL) (RT, 3 h).

Yield: 74.0 mg (0.29 mmol, 96%) brown solid. **Mp.** 55-56 °C. $\mathbf{R_f}$= 0.47 in hexane : ethyl acetate 4:1. **MS** (EI, 70 eV): m/z (%): 259 (4), 257 (14, [M^+]), 123 (8), 122 (100), 94 (10), 93 (7), 69 (3), 65 (8). ^{1}**H NMR** (250 MHz, Chloroform-*d*) δ 7.07 (t, *J* = 8.0 Hz, 1H), 6.30 (d, *J* = 8.0 Hz, 2H), 6.21 (s, 1H), 4.68 (bs, 2H), 3.95 (dd, *J* = 15.3, 12.5 Hz, 1H), 3.79 (dd, *J* = 21.3, 15.3 Hz, 1H). ^{19}**F NMR** (235 MHz, Chloroform-*d*) δ -80.6 (d, *J* = 6.1 Hz), -130.2 (q, *J* = 6.1 Hz). ^{13}**C NMR** (63 MHz, Chloroform-*d*) δ 156.7, 148.1, 130.6, 120.7 (qd, *J* = 284.8, 31.1 Hz), 106.8 (dq, *J* = 255.6, 34.9 Hz), 106.3, 100.6, 48.9 (d, *J* = 22.2 Hz). **IR** (film, ATR) 1601, 1523, 1499, 1441, 1329, 1305, 1186, 1165, 1146, 1111, 1061, 995, 977, 945, 887, 831, 762, 731, 686, 664 cm^{-1}. **HRMS** (EI) $[M]^+$ calculated for $C_9H_8ClF_4NO^+$: 257.0231, found: 257.0223.

N-(2-Chloro-2,3,3,3-tetrafluoropropyl)-4-methylaniline (27)

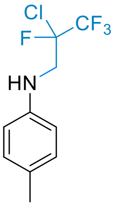

Method B. Using p-toluidine (32.2 mg, 0.30 mmol), Li_2CO_3 (26.6 mg, 0.36 mmol) and iodonium salt (171.8 mg, 0.33 mmol) in MeCN (1.0 mL) (RT, 2 h).

Yield: 67.0 mg (0.26 mmol, 87%) yellow solid. **Mp.** 43-46 °C. $\mathbf{R_f}$= 0.57 in hexane : ethyl acetate 10:1. **MS** (EI, 70 eV): m/z (%): 369 (10), 367 (30, [M^+]), 240 (6), 233 (8), 232 (100), 136 (7), 116 (8), 106 (11), 105 (52), 104 (14), 91 (8), 77 (8), 76 (15), 69 (4), 63 (11). ^{1}**H NMR** (250 MHz, Chloroform-*d*) δ 7.07 (d, *J* = 8.1 Hz, 2H), 6.67 (d, *J* = 8.3 Hz, 2H), 4.11 – 3.67 (m, 3H), 2.30 (s, 3H). ^{19}**F NMR** (235 MHz, Chloroform-*d*) δ -80.6 (d, *J* = 6.2 Hz), -130.1 (q, *J* = 6.1 Hz). ^{13}**C NMR** (63 MHz, Chloroform-*d*) δ 144.0, 130.0, 128.7, 120.8 (qd, *J* = 284.8, 31.0 Hz), 113.7, 107.0 (dq, *J* = 255.4, 34.8 Hz), 49.5 (d, *J* = 22.1 Hz), 20.5. **IR** (film, ATR) 1618, 1525, 1437, 1374, 1312, 1303, 1271, 1255, 1212, 1189, 1156, 1128, 1117, 1105, 1046, 971, 951, 899, 803, 740, 720, 660 cm^{-1}. **HRMS** (EI) $[M]^+$ calculated for $C_{10}H_{10}ClF_4N^+$: 255.0438, found: 255.0434.

N-(2-Chloro-2,3,3,3-tetrafluoropropyl)-4-fluoroaniline (28)

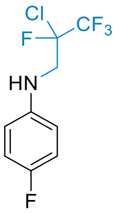

Method A. Using 4-fluoroaniline (33.3 mg, 28.4 µL, 0.30 mmol), Li_2CO_3 (26.6 mg, 0.36 mmol) and iodonium salt (171.8 mg, 0.33 mmol) in MeCN (1.0 mL) (RT, 3 h).

Yield: 64.1 mg (0.25 mmol, 82%) yellowish oil. $\mathbf{R_f}$= 0.69 in hexane : ethyl acetate 4:1. **MS** (EI, 70 eV): m/z (%): 261 (4), 259 (12, [M^+]), 125 (8), 124 (100), 97 (6), 96 (7), 95 (11), 83 (8), 75 (6), 69 (3). **^{1}H NMR** (250 MHz, Chloroform-*d*) δ 7.02 – 6.79 (m, 2H), 6.78 – 6.49 (m, 2H), 3.97 (dd, *J* = 15.3, 12.3 Hz, 1H), 3.85 (s, 1H), 3.81 (dd, *J* = 21.2, 15.2 Hz, 1H). **^{19}F NMR** (235 MHz, Chloroform-*d*) δ -80.6 (d, *J* = 6.1 Hz), -125.9, -130.2 (q, *J* = 6.1 Hz). **^{13}C NMR** (63 MHz, Chloroform-*d*) δ 156.8 (d, *J* = 237.1 Hz), 142.7 (d, *J* = 2.1 Hz), 120.7 (qd, *J* = 284.8, 31.0 Hz), 116.0 (d, *J* = 22.6 Hz), 114.6 (d, *J* = 7.6 Hz), 107.0 (dq, *J* = 255.0, 34.9 Hz), 49.8 (d, *J* = 22.1 Hz). **IR** (film, ATR) 1616, 1516, 1439, 1378, 1307, 1268, 1255, 1217, 1193, 1148, 1115, 1096, 1064, 1044, 973, 947, 899, 822, 777, 761, 744, 733, 699, 660 cm^{-1}. **HRMS** (EI) $[M]^+$ calculated for $C_9H_7ClF_5N^+$: 259.0187, found: 259.0179.

4-Chloro-*N*-(2-chloro-2,3,3,3-tetrafluoropropyl)aniline (29)

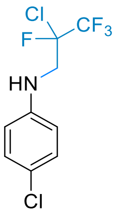

Method B. Using 4-chloroaniline (38.3 mg, 0.30 mmol), Li_2CO_3 (26.6 mg, 0.36 mmol) and iodonium salt (171.8 mg, 0.33 mmol) in MeCN (1.0 mL) (RT, 4 h).

Yield: 80.7 mg (0.29 mmol, 97%) yellow oil. $\mathbf{R_f}$= 0.76 in hexane : ethyl acetate 4:1. **MS** (EI, 70 eV): m/z (%): 277 (6), 275 (10, [M^+]), 142 (32), 141 (9), 140 (100), 111 (7), 105 (7), 99 (6), 77 (6), 75 (8), 69 (3), 63 (5). **^{1}H NMR** (250 MHz, Chloroform-*d*) δ 7.17 (d, *J* = 8.8 Hz, 2H), 6.64 (d, *J* = 8.8 Hz, 2H), 3.98 (dd, *J* = 15.4, 12.4 Hz, 1H), 3.88 (s, 1H), 3.83 (dd, *J* = 21.1, 15.3 Hz, 1H). **^{19}F NMR** (235 MHz, Chloroform-*d*) δ -80.6 (d, *J* = 6.2 Hz), -130.2 (q, *J* = 6.1 Hz). **^{13}C NMR** (63 MHz, Chloroform-*d*) δ 145.0, 129.4, 123.9, 120.7 (qd, *J* = 285.0, 31.0 Hz), 114.5, 106.9 (dq, *J* = 255.4, 34.7 Hz), 49.1 (d, *J* = 22.1 Hz). **IR** (film, ATR) 1603, 1508, 1491, 1439, 1378, 1307, 1296, 1268, 1249, 1214, 1191, 1150, 1124, 1094, 1063, 1046, 1005, 973, 949, 897, 815, 761, 731, 710, 667 cm^{-1}. **HRMS** (EI) $[M]^+$ calculated for $C_9H_7Cl_2F_4N^+$: 274.9892, found: 274.9882.

4-Bromo-*N*-(2-chloro-2,3,3,3-tetrafluoropropyl)aniline (30)

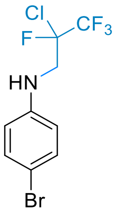

Method B. Using 4-bromoaniline (51.6 mg, 0.30 mmol), Li_2CO_3 (26.6 mg, 0.36 mmol) and iodonium salt (171.8 mg, 0.33 mmol) in MeCN (1.0 mL) (RT, 2 h).

Yield: 79.0 mg (0.25 mmol, 82%) brown oil. $\mathbf{R_f}$= 0.63 in hexane : ethyl acetate 4:1. **MS** (EI, 70 eV): m/z (%): 321 (14), 319 (11, [M^+]), 205 (7), 187 (7), 186 (93), 185 (11), 184 (100), 105 (36), 104 (20), 91 (13), 76 (15), 75 (12), 69 (9), 64 (12), 63 (16). **^{1}H NMR** (250 MHz, Chloroform-*d*) δ 7.20 (d, *J* = 8.7 Hz, 2H), 6.49 (d, *J* = 8.7 Hz, 2H), 4.05 – 3.56 (m, 3H). **^{19}F NMR** (235 MHz, Chloroform-*d*) δ -80.6 (d, *J* = 6.2 Hz), -130.2 (q, *J* = 6.2 Hz). **^{13}C NMR** (63 MHz, Chloroform-*d*) δ 145.4, 132.3, 120.7 (qd, *J* = 284.9, 31.1 Hz), 115.0 (d, *J* = 1.1 Hz), 111.0, 106.8 (dq, *J* = 255.4, 34.8 Hz), 48.9 (d, *J* = 22.2 Hz). **IR** (film, ATR) 1598, 1508, 1488, 1309, 1296, 1269, 1249, 1214, 1191, 1150, 1124, 1107, 1076, 1061, 1044, 1003, 971, 947, 891, 813, 759, 746, 731, 679, 664 cm^{-1}. **HRMS** (EI) $[M]^+$ calculated for $C_9H_7BrClF_4N^+$: 318.9387, found: 318.9382.

N-(2-Chloro-2,3,3,3-tetrafluoropropyl)-4-iodoaniline (31)

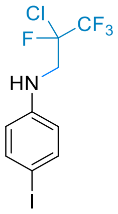

Method B. Using 4-iodoaniline (65.7 mg, 0.30 mmol), Li_2CO_3 (26.6 mg, 0.36 mmol) and iodonium salt (171.8 mg, 0.33 mmol) in MeCN (1.0 mL) (RT, 5 h).

Yield: 106.8 mg (0.29 mmol, 97%) yellow solid. **Mp.** 32-35 °C. **R_f**= 0.70 in hexane : ethyl acetate 4:1. **MS** (EI, 70 eV): m/z (%): 369 (10), 367 (30, [M^+]), 240 (6), 233 (8), 232 (100), 136 (7), 116 (8), 106 (11), 105 (52), 104 (14), 91 (8), 77 (8), 76 (15), 69 (4), 63 (11). **^{1}H NMR** (250 MHz, Chloroform-*d*) δ 7.48 (d, *J* = 8.6 Hz, 2H), 6.50 (d, *J* = 8.7 Hz, 2H), 4.25 – 3.46 (m, 3H). **^{19}F NMR** (235 MHz, Chloroform-*d*) δ -80.5 (d, *J* = 6.1 Hz), -130.2 (q, *J* = 6.1 Hz). **^{13}C NMR** (63 MHz, Chloroform-*d*) δ 146.1, 138.1, 120.6 (qd, *J* = 285.1, 31.2 Hz), 115.5 (d, *J* = 1.1 Hz), 106.8 (dq, *J* = 255.4, 34.7 Hz), 80.2, 48.7 (d, *J* = 22.2 Hz). **IR** (film, ATR) 1594, 1506, 1484, 1309, 1294, 1269, 1249, 1214, 1189, 1150, 1124, 1107, 1066, 1044, 997, 973, 947, 895, 811, 731, 693, 664 cm^{-1}. **HRMS** (EI) $[M]^+$ calculated for $C_9H_7ClF_4IN^+$: 366.9248, found: 366.9247.

1-(4-((2-Chloro-2,3,3,3-tetrafluoropropyl)amino)phenyl)ethan-1-one (32)

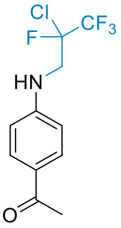

Method B. Using 1-(4-aminophenyl)ethan-1-one (40.5 mg, 0.30 mmol), Li_2CO_3 (26.6 mg, 0.36 mmol) and iodonium salt (171.8 mg, 0.33 mmol) in MeCN (1.0 mL) (RT, 4 h).

Yield: 79.4 mg (0.28 mmol, 93%) white solid. **Mp.** 159-163 °C. **R_f**= 0.26 in hexane : ethyl acetate 4:1. **MS** (EI, 70 eV): m/z (%): 285 (12), 283 (36, [M^+]), 270 (14), 268 (45), 149 (11), 148 (100), 135 (9), 119 (7), 106 (10), 105 (27), 104 (9), 91 (11), 77 (8), 69 (7), 83 (8). **^{1}H NMR** (250 MHz, Chloroform-*d*) δ 7.84 (d, *J* = 8.8 Hz, 2H), 6.71 (d, *J* = 8.8 Hz, 2H), 4.68 (bs, 1H), 4.07 (dd, *J* = 15.6, 12.6 Hz, 1H), 3.92 (dd, *J* = 20.9, 15.6 Hz, 1H), 2.50 (s, 3H). **^{19}F NMR** (235 MHz, Chloroform-*d*) δ -80.6 (d, *J* = 6.3 Hz), -130.2 (q, *J* = 6.3 Hz). **^{13}C NMR** (63 MHz, Chloroform-*d*) δ 196.7, 150.8, 130.8, 128.4, 120.6 (qd, *J* = 284.9, 30.9 Hz), 112.2 (d, *J* = 1.1 Hz), 106.6 (dq, *J* = 255.6, 34.9 Hz), 48.0 (d, *J* = 22.4 Hz), 26.1. **IR** (film, ATR) 1657, 1598, 1583, 1536, 1437, 1366, 1342, 1318, 1277, 1249, 1215, 1191, 1184, 1132, 1115, 1068, 1046, 960, 938, 828 cm^{-1}. **HRMS** (ESI) $[M+H]^+$ calculated for $C_{11}H_{11}ClF_4NO^+$: 284.0460, found: 284.0463.

N-(2-Chloro-2,3,3,3-tetrafluoropropyl)-4-nitroaniline (33)

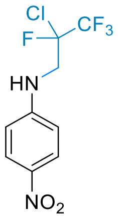

Method B. Using 4-nitroaniline (41.4 mg, 0.30 mmol), Li_2CO_3 (26.6 mg, 0.36 mmol) and iodonium salt (171.8 mg, 0.33 mmol) in MeCN (1.0 mL) (RT, 19 h).

Yield: 71.0 mg (0.25 mmol, 78%) yellow solid. **Mp.** 161-164 °C. **R_f**= 0.37 in hexane : ethyl acetate 4:1. **MS** (EI, 70 eV): m/z (%): 288 (3), 286 (9, [M^+]), 205 (4), 152 (9), 151 (100), 135 (9), 105 (70), 104 (13), 76 (12), 69 (7), 64 (12), 63 (12). **^{1}H NMR** (250 MHz, Acetonitrile-d_3) δ 8.05 (d, *J* = 9.2 Hz, 2H), 6.81 (d, *J* = 9.2 Hz, 2H), 5.96 (bs, 1H), 4.33 – 3.78 (m, 2H). **^{19}F NMR** (235 MHz, Acetonitrile-d_3) δ -81.3 (d, *J* = 6.2 Hz), -130.4 (q, *J* = 6.0 Hz). **^{13}C NMR** (63 MHz, Acetonitrile-d_3) δ 154.1, 139.9, 126.9, 121.6 (qd, *J* = 284.2, 31.2 Hz), 112.8 (d, *J* = 1.4 Hz), 107.9 (dq, *J* = 253.8, 34.6 Hz), 48.2 (d, *J* = 22.0 Hz). **IR** (film, ATR) 1601, 1555, 1506, 1489, 1344, 1324, 1307, 1284, 1251, 1199, 1111, 1070, 936, 859, 839, 815, 753, 697 cm^{-1}. **HRMS** (EI) $[M]^+$ calculated for $C_9H_7ClF_4N_2O_2^+$: 286.0132, found: 286.0127.

4-((2-Chloro-2,3,3,3-tetrafluoropropyl)amino)benzonitrile (34)

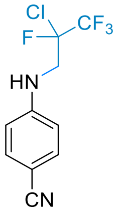

Method B. Using 4-aminobenzonitrile (35.4 mg, 0.30 mmol), Li_2CO_3 (26.6 mg, 0.36 mmol) and iodonium salt (171.8 mg, 0.33 mmol) in MeCN (1.0 mL) (RT, 19 h).

Yield: 66.0 mg (0.25 mmol, 83%) yellow solid. **Mp.** 159-161 °C. **R_f**= 0.30 in hexane : ethyl acetate 4:1. **MS** (EI, 70 eV): m/z (%): 268 (4), 266 (13, [M^+]), 231 (4), 132 (9), 131 (100), 129 (6), 104 (7), 102 (12), 90 (8), 75 (7), 69 (8). **^{1}H NMR** (250 MHz, Chloroform-*d*) δ 7.47 (d, *J* = 8.8 Hz, 2H), 6.72 (d, *J* = 8.8 Hz, 2H), 4.65 (bs, 1H), 4.06 (dd, *J* = 15.6, 12.4 Hz, 1H), 3.91 (dd, *J* = 20.8, 15.5 Hz, 1H). **^{19}F NMR** (235 MHz, Chloroform-*d*) δ -80.6 (d, *J* = 6.1 Hz), -130.3 (q, *J* = 6.2 Hz). **^{13}C NMR** (63 MHz, Chloroform-*d*) δ 149.9, 133.9, 120.5 (qd, *J* = 285.2, 30.6 Hz), 119.9, 113.0 (d, *J* = 1.2 Hz), 106.5 (dq, *J* = 255.5, 35.1 Hz), 101.2, 48.0 (d, *J* = 22.3 Hz). **IR** (film, ATR) 2215, 1605, 1581, 1534, 1441, 1348, 1309, 1284, 1268, 1232, 1215, 1189, 1176, 1159, 1139, 1122, 1107, 1061, 1046, 977, 824, 733, 667 cm^{-1}. **HRMS** (EI) $[M]^+$ calculated for $C_{10}H_7ClF_4N_2^+$: 266.0234, found: 266.0225.

4-((2-Chloro-2,3,3,3-tetrafluoropropyl)amino)phenol (35)

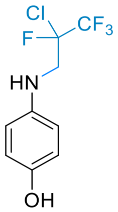

Method B. Using 4-aminophenol (32.7 mg, 0.30 mmol), Li_2CO_3 (26.6 mg, 0.36 mmol) and iodonium salt (171.8 mg, 0.33 mmol) in MeCN (1.0 mL) (RT, 3 h).

Yield: 33.0 mg (0.13 mmol, 43%) brown solid. **Mp.** 60-61 °C. **R_f**= 0.36 in hexane : ethyl acetate 4:1. **MS** (EI, 70 eV): m/z (%): 259 (4), 257 (13, [M^+]), 222 (3), 123 (8), 122 (100), 121 (6), 108 (8), 94 (14), 81 (8), 69 (5), 65 (10). **^{1}H NMR** (500 MHz, DMSO-d_6) δ 8.54 (bs, 1H), 6.68 – 6.35 (m, 4H), 5.68 (bs, 1H), 3.95 (t, *J* = 15.3 Hz, 1H), 3.84 (dd, *J* = 22.3, 15.8 Hz, 1H). **^{19}F NMR** (376 MHz, DMSO-d_6) δ -79.4 (d, *J* = 6.4 Hz), -128.6 – -128.9 (m). **^{13}C NMR** (126 MHz, DMSO-d_6) δ 149.2, 140.2, 120.6 (qd, *J* = 285.2, 31.8 Hz), 115.6, 113.9, 107.7 (dq, *J* = 253.2, 33.3 Hz), 49.0 (d, *J* = 21.3 Hz). **IR** (film, ATR) 1518, 1439, 1376, 1305, 1190, 1149, 1119, 1100, 1063, 973, 899, 824, 750, 731, 664 cm^{-1}. **HRMS** (ESI) $[M+H]^+$ calculated for $C_9H_9ClF_4NO^+$: 258.0303, found: 258.0300.

***N*-(2-Chloro-2,3,3,3-tetrafluoropropyl)-4-methoxyaniline (36)**

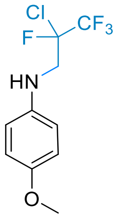

Method B. Using 4-methoxyaniline (36.9 mg, 0.30 mmol), Li_2CO_3 (26.6 mg, 0.36 mmol) and iodonium salt (171.8 mg, 0.33 mmol) in MeCN (1.0 mL) (0 °C, 10 min.).

Yield: 51.7 mg (0.19 mmol, 63%) brown oil. **R_f**= 0.63 in hexane : ethyl acetate 4:1. **MS** (EI, 70 eV): m/z (%): 273 (5), 271 (17, [M^+]), 137 (9), 136 (100), 121 (13), 108 (6), 93 (4), 92 (3), 77 (3), 69 (2). **^{1}H NMR** (500 MHz, DMSO-d_6) δ 6.76 – 6.71 (m, 2H), 6.70 – 6.66 (m, 2H), 5.90 (bs, 1H), 4.00 (t, *J* = 15.4 Hz, 1H), 3.89 (dd, *J* = 22.7, 16.4 Hz, 1H), 3.64 (s, 3H). **^{19}F NMR** (376 MHz, DMSO-d_6) δ -79.4 (d, *J* = 6.4 Hz), -128.5 – -128.9 (m). **^{13}C NMR** (126 MHz, DMSO-d_6) δ 151.5, 141.5, 120.6 (qd, *J* = 285.2, 31.7 Hz), 114.5, 113.6, 107.7 (dq, *J* = 254.0, 33.4 Hz), 55.3, 48.6 (d, *J* = 21.4 Hz). **IR** (film, ATR) 1514, 1465, 1443, 1378, 1307, 1251, 1236, 1214, 1189, 1148, 1104, 1064, 1033, 971, 943, 897, 818, 761, 733, 658 cm^{-1}. **HRMS** (EI) $[M]^+$ calculated for $C_{10}H_{10}ClF_4NO^+$: 271.0387, found: 271.0379.

***N*-(2-Chloro-2,3,3,3-tetrafluoropropyl)-2-methoxyaniline (37)**

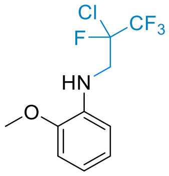

Method A. Using 2-methoxyaniline (36.9 mg, 33.8 μL, 0.30 mmol), Li_2CO_3 (26.6 mg, 0.36 mmol) and iodonium salt (171.8 mg, 0.33 mmol) in MeCN (1.0 mL) (0 °C, 30 min.).

Yield: 70.7 mg (0.26 mmol, 87%) yellowish oil. **R_f**= 0.73 in hexane : ethyl acetate 4:1. **MS** (EI, 70 eV): m/z (%): 273 (6), 271 (19, [M^+]), 137 (9), 136 (100), 122 (5), 121 (36),

120 (34), 93 (8), 92 (6), 77 (6), 69 (3), 65 (9). **^{1}H NMR** (250 MHz, Chloroform-*d*) δ 7.08 – 6.55 (m, 4H), 4.52 (bs, 1H), 4.14 – 3.67 (m, 5H). **^{19}F NMR** (235 MHz, Chloroform-*d*) δ -80.6 (d, *J* = 6.1 Hz), -130.1 (q, *J* = 6.2 Hz). **^{13}C NMR** (63 MHz, Chloroform-*d*) δ 147.1, 136.5, 121.3, 120.8 (qd, *J* = 285.2, 31.0 Hz), 118.4, 110.4 (d, *J* = 2.0 Hz), 110.2, 107.1 (dq, *J* = 255.4, 34.5 Hz), 55.7, 48.9 (d, *J* = 22.1 Hz). **IR** (film, ATR) 1605, 1525, 1461, 1435, 1378, 1350, 1325, 1307, 1275, 1253, 1223, 1191, 1152, 1126, 1098, 1070, 1046, 1029, 973, 943, 902, 738, 662 cm^{-1}. **HRMS** (EI) $[M]^+$ calculated for $C_{10}H_{10}ClF_4NO^+$: 271.0387, found: 271.0379.

N-(2-Chloro-2,3,3,3-tetrafluoropropyl)-3-methoxyaniline (38)

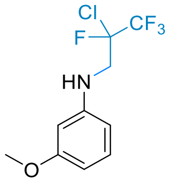

Method A. Using 3-methoxyaniline (36.9 mg, 33.5 µL, 0.30 mmol), Li_2CO_3 (26.6 mg, 0.36 mmol) and iodonium salt (171.8 mg, 0.33 mmol) in MeCN (1.0 mL) (0 °C, 30 min.).

Yield: 74.6 mg (0.27 mmol, 92%) yellow oil. **R$_f$**= 0.59 in hexane : ethyl acetate 4:1. **MS** (EI, 70 eV): m/z (%): 273 (5), 271 (15, [M$^+$]), 236 (3), 137 (9), 136 (100), 121 (6), 108 (8), 93 (5), 92 (5), 77 (5), 69 (2). **^{1}H NMR** (250 MHz, Chloroform-*d*) δ 7.15 (t, *J* = 8.1 Hz, 1H), 6.45 – 6.18 (m, 3H), 4.11 – 3.68 (m, 6H). **^{19}F NMR** (235 MHz, Chloroform-*d*) δ -80.6 (d, *J* = 6.3 Hz), -130.2 (q, *J* = 6.2 Hz). **^{13}C NMR** (63 MHz, Chloroform-*d*) δ 161.0, 147.9, 130.3, 120.7 (qd, *J* = 284.8, 31.2 Hz), 106.9 (dq, *J* = 255.2, 35.0 Hz), 106.3, 104.2, 99.8 (d, *J* = 1.2 Hz), 55.2, 49.0 (d, *J* = 22.2 Hz). **IR** (film, ATR) 1616, 1601, 1519, 1499, 1463, 1437, 1378, 1342, 1327, 1305, 1262, 1212, 1200, 1189, 1167, 1145, 1111, 1044, 992, 975, 938, 923, 880, 828, 759, 733, 686, 662 cm^{-1}. **HRMS** (EI) $[M]^+$ calculated for $C_{10}H_{10}ClF_4NO^+$: 271.0387, found: 271.0384.

N-(2-Chloro-2,3,3,3-tetrafluoropropyl)-2,4-dimethoxyaniline (39)

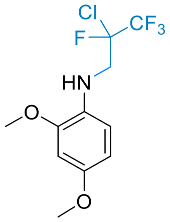

Method B. Using 2,4-dimethoxyaniline (46.0 mg, 0.30 mmol), Li_2CO_3 (26.6 mg, 0.36 mmol) and iodonium salt (171.8 mg, 0.33 mmol) in MeCN (1.5 mL) (0 °C, 15 min.).

Yield: 25.6 mg (0.08 mmol, 28%) brown oil. **R$_f$**= 0.58 in hexane : ethyl acetate 4:1. **MS** (EI, 70 eV): m/z (%): 303 (9), 301 (27, [M$^+$]), 286 (5), 167 (10), 166 (100), 152 (12), 151 (54), 150 (18), 136 (14), 123 (6), 108 (8), 79 (6), 69 (6). **^{1}H NMR** (500 MHz, DMSO-*d*$_6$) δ 6.64 (dd, *J* = 8.6, 1.2 Hz, 1H), 6.52 (d, *J* = 2.7 Hz, 1H), 6.38 (dd, *J* = 8.7, 2.7 Hz, 1H), 5.09 (bs, 1H), 4.10 – 3.89 (m, 2H), 3.79 (s, 3H), 3.67 (s, 3H). **^{19}F NMR** (376 MHz, DMSO-*d*$_6$) δ -79.5 (d, *J* = 6.4 Hz), -128.4 – -128.6 (m). **^{13}C NMR** (126 MHz, DMSO-*d*$_6$) δ 151.9, 147.4, 130.6, 120.6 (qd, *J* = 285.2, 31.8 Hz), 110.4 (d, *J* = 2.7 Hz), 107.8 (dq, *J* = 254.2, 33.4 Hz), 104.0, 99.2, 55.6, 55.3, 48.2 (d, *J* = 21.2 Hz). **IR** (film, ATR) 1600, 1521, 1462, 1439, 1421, 1290, 1260, 1205, 1186, 1156, 1134, 1104, 1033, 973, 932, 917, 899, 835, 787, 731, 712, 660 cm^{-1}. **HRMS** (EI) $[M]^+$ calculated for $C_{11}H_{12}ClF_4NO_2^+$: 301.0493, found: 301.0483.

N-(2-Chloro-2,3,3,3-tetrafluoropropyl)-3,5-dimethoxyaniline (40)

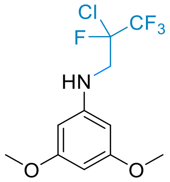

Method B. Using 3,5-dimethoxyaniline (46.0 mg, 0.30 mmol), Li_2CO_3 (26.6 mg, 0.36 mmol) and iodonium salt (171.8 mg, 0.33 mmol) in MeCN (1.0 mL) (0 °C, 30 min.).

Yield: 83.4 mg (0.28 mmol, 92%) yellow oil. **R$_f$**= 0.47 in hexane : ethyl acetate 4:1. **MS** (EI, 70 eV): m/z (%): 303 (6), 301 (17, [M$^+$]), 266 (3), 167 (10), 166 (100), 151 (4), 138 (8), 122 (4), 108 (4), 83 (4), 69 (3). **^{1}H NMR** (250 MHz, Chloroform-*d*) δ 5.98 (s, 1H), 5.89 (s, 2H), 4.17 – 3.57 (m, 9H). **^{19}F NMR** (235 MHz, Chloroform-*d*) δ -80.6 (d, *J* = 6.2 Hz), -130.1 (q, *J* = 6.1 Hz). **^{13}C NMR** (63 MHz, Chloroform-*d*) δ 161.9, 148.5, 120.7 (qd, *J* = 284.9, 31.1 Hz), 106.8 (dq, *J* = 255.4,

34.8 Hz), 92.4, 91.2, 55.3, 48.9 (d, J = 22.3 Hz). **IR** (film, ATR) 1620, 1601, 1527, 1486, 1460, 1439, 1420, 1379, 1309, 1266, 1206, 1154, 1113, 1061, 994, 977, 943, 880, 813, 731, 682 cm^{-1}. **HRMS** (ESI) $[M+H]^+$ calculated for $C_{11}H_{13}ClF_4NO_2^+$: 302.0560, found: 302.0567.

N-(2-Chloro-2,3,3,3-tetrafluoropropyl)naphthalen-2-amine (41)

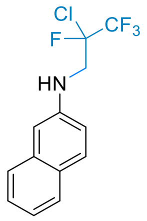

Method B. Using naphthalen-2-amine (43.0 mg, 0.30 mmol), Li_2CO_3 (26.6 mg, 0.36 mmol) and iodonium salt (171.8 mg, 0.33 mmol) in MeCN (1.0 mL) (RT, 3 h).

Yield: 80.6 mg (0.28 mmol, 92%) pink solid. **Mp.** 105-110 °C. $\mathbf{R_f}$= 0.62 in hexane : ethyl acetate 4:1. **MS** (EI, 70 eV): m/z (%): 293 (8), 291 (23, $[M^+]$), 256 (3), 157 (13), 156 (100), 155 (7), 129 (11), 128 (18), 127 (21), 115 (17), 78 (11), 77 (6), 69 (2). ^{1}**H NMR** (500 MHz, DMSO-d_6) δ 7.67 (t, J = 7.9 Hz, 2H), 7.60 (d, J = 8.0 Hz, 1H), 7.33 (td, J = 8.2, 1.2 Hz, 1H), 7.16 (td, J = 6.9, 1.2 Hz, 1H), 7.12 (dd, J = 8.8, 2.4 Hz, 1H), 7.00 (d, J = 2.3 Hz, 1H), 6.60 (t, J = 7.0 Hz, 1H), 4.21 (td, J = 15.6, 6.8 Hz, 1H), 4.12 (ddd, J = 22.8, 15.9, 7.2 Hz, 1H). ^{19}**F NMR** (376 MHz, DMSO-d_6) δ -79.4 (d, J = 6.4 Hz), -128.3 – -128.6 (m). ^{13}**C NMR** (126 MHz, DMSO-d_6) δ 145.4, 134.7, 128.5, 127.4, 127.0, 126.2, 125.7, 121.8, 120.6 (qd, J = 285.2, 31.7 Hz), 117.8, 107.5 (dq, J = 253.7, 33.6 Hz), 103.8 (d, J = 1.8 Hz), 47.5 (d, J = 21.6 Hz). **IR** (film, ATR) 1633, 1600, 1503, 1432, 1369, 1301, 1275, 1249, 1205, 1186, 1145, 1126, 1100, 1063, 973, 943, 891, 843, 809, 757, 735, 660 cm^{-1}. **HRMS** (EI) $[M]^+$ calculated for $C_{13}H_{10}ClF_4N^+$: 291.0438, found: 291.0435.

N-(2-Chloro-2,3,3,3-tetrafluoropropyl)-*N*-methylaniline (42)

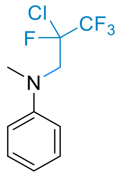

Method A. Using *N*-methylaniline (32.2 mg, 32.6 µL, 0.30 mmol), Li_2CO_3 (26.6 mg, 0.36 mmol) and iodonium salt (171.8 mg, 0.33 mmol) in MeCN (1.0 mL) (RT, 3 h).

Yield: 44.9 mg (0.18 mmol, 56%) yellowish oil. $\mathbf{R_f}$= 0.70 in hexane : ethyl acetate 10:1. **MS** (EI, 70 eV): m/z (%): 257 (4), 255 (13, $[M^+]$), 220 (4), 121 (9), 120 (100), 105 (9), 104 (9), 91 (4), 77 (18), 69 (2). ^{1}**H NMR** (250 MHz, Chloroform-*d*) δ 7.39 – 7.27 (m, 2H), 6.92 – 6.78 (m, 3H), 4.14 (t, J = 15.7 Hz, 1H), 4.04 (dd, J = 22.3, 16.3 Hz, 1H), 3.12 (s, 3H). ^{19}**F NMR** (235 MHz, Chloroform-*d*) δ -80.8 (d, J = 6.0 Hz), -127.1 (q, J = 6.1 Hz). ^{13}**C NMR** (63 MHz, Chloroform-*d*) δ 149.0, 129.3, 120.8 (qd, J = 285.1, 31.5 Hz), 118.4, 112.9 (d, J = 1.5 Hz), 107.9 (dq, J = 256.6, 34.2 Hz), 57.4 (d, J = 20.3 Hz), 40.1 (d, J = 2.0 Hz). **IR** (film, ATR) 1601, 1579, 1506, 1486, 1447, 1426, 1368, 1348, 1305, 1260, 1214, 1189, 1143, 1118, 1059, 1035, 994, 975, 941, 872, 749, 723, 692, 658 cm^{-1}. **HRMS** (EI) $[M]^+$ calculated for $C_{10}H_{10}ClF_4N^+$: 255.0438, found: 255.0435.

N-(2-Chloro-2,3,3,3-tetrafluoropropyl)-*N*-ethylaniline (43)

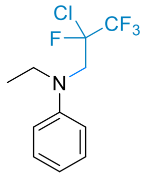

Method A. Using *N*-ethylaniline (36.4 mg, 37.8 µL, 0.30 mmol), Li_2CO_3 (26.6 mg, 0.36 mmol) and iodonium salt (171.8 mg, 0.33 mmol) in MeCN (1.0 mL) (RT, 48 h).

Yield: 35.8 mg (0.13 mmol, 44%) yellowish oil. $\mathbf{R_f}$= 0.53 in hexane : ethyl acetate 10:1. **MS** (EI, 70 eV): m/z (%): 271 (3), 269 (9, $[M^+]$), 254 (3), 234 (3), 135 (11), 134 (100), 106 (27), 105 (9), 104 (13), 91 (5), 77 (20), 69 (2). ^{1}**H NMR** (250 MHz, Chloroform-*d*) δ 7.40 – 7.16 (m, 2H), 6.97 – 6.76 (m, 3H), 4.10 (t, J = 15.5 Hz, 1H), 4.00 (dd, J = 23.5, 16.3 Hz, 1H), 3.57 (q, J = 7.0 Hz, 2H), 1.22 (t, J = 7.0 Hz, 3H). ^{19}**F NMR** (235 MHz, Chloroform-*d*) δ -80.8 (d, J = 6.1 Hz), -126.8 (q, J = 6.1 Hz). ^{13}**C NMR** (63 MHz, Chloroform-*d*) δ 147.5, 129.4, 120.9 (qd, J = 284.6, 31.8 Hz), 118.3, 113.6 (d, J = 1.6 Hz), 108.0 (dq, J = 256.1, 34.0 Hz), 55.5 (d, J = 20.3 Hz), 46.4 (d, J = 1.7 Hz), 11.4. **IR** (film, ATR)

1601, 1506, 1376, 1353, 1305, 1253, 1215, 1191, 1143, 1124, 1064, 1051, 1036, 1014, 999, 962, 919, 748, 720, 693 cm^{-1}. **HRMS** (EI) $[M]^+$ calculated for $C_{11}H_{12}ClF_4N^+$: 269.0594, found: 269.0583.

1-(2-Chloro-2,3,3,3-tetrafluoropropyl)indoline (44)

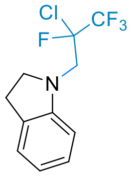

Method A. Using indoline (35.8 mg, 33.7 µL, 0.30 mmol), Li_2CO_3 (26.6 mg, 0.36 mmol) and iodonium salt (171.8 mg, 0.33 mmol) in MeCN (1.0 mL) (RT, 2 h).

Yield: 39.0 mg (0.15 mmol, 49%) gray oil. $\mathbf{R_f}$= 0.81 in hexane : ethyl acetate 4:1. **MS** (EI, 70 eV): m/z (%): 269 (4), 267 (14, $[M^+]$), 232 (4), 133 (11), 132 (100), 130 (11), 117 (19), 91 (9), 77 (7), 69 (3). ^{1}**H NMR** (250 MHz, Chloroform-*d*) δ 7.12 (t, *J* = 7.5 Hz, 2H), 6.77 (t, *J* = 7.3 Hz, 1H), 6.56 (d, *J* = 7.9 Hz, 1H), 3.94 – 3.70 (m, 2H), 3.64 (t, *J* = 9.0 Hz, 2H), 3.10 (t, *J* = 8.5 Hz, 2H). ^{19}**F NMR** (235 MHz, Chloroform-*d*) δ -80.4 (d, *J* = 6.1 Hz), -128.5 (q, *J* = 6.0 Hz). ^{13}**C NMR** (63 MHz, Chloroform-*d*) δ 151.4, 129.1, 127.6, 124.8, 120.8 (qd, *J* = 285.0, 31.2 Hz), 119.0, 107.4 (dq, *J* = 254.3, 34.1 Hz), 106.8 (d, *J* = 1.5 Hz), 56.4 (d, *J* = 22.1 Hz), 55.6, 28.9. **IR** (film, ATR) 1609, 1491, 1476, 1461, 1432, 1374, 1327, 1307, 1296, 1275, 1256, 1212, 1189, 1163, 1137, 1091, 1072, 1053, 1023, 1007, 995, 979, 960, 938, 882, 843, 744, 727, 714, 667 cm^{-1}. **HRMS** (EI) $[M]^+$ calculated for $C_{11}H_{10}ClF_4N^+$: 267.0438, found: 267.0434.

4-((2-Chloro-2,3,3,3-tetrafluoropropyl)amino)-1-methyl-3-propyl-1*H*-pyrazole-5-carboxamide (46)

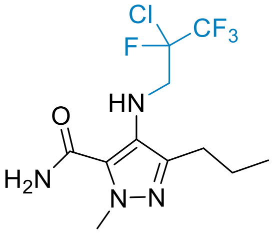

Method B. Using 4-amino-1-methyl-3-propyl-1*H*-pyrazole-5-carboxamide (54.7 mg, 0.30 mmol), Li_2CO_3 (26.6 mg, 0.36 mmol) and iodonium salt (171.8 mg, 0.33 mmol) in MeCN (1.0 mL) (RT, 3 h).

Yield: 89.6 mg (0.27 mmol, 90%) pink solid. **Mp.** 115-117 °C. $\mathbf{R_f}$= 0.44 in hexane : ethyl acetate 1:1. **MS** (EI, 70 eV): m/z (%): 332 (7), 330 (23, $[M^+]$), 294 (8), 278 (6), 258 (4), 249 (6), 208 (13), 195 (37), 179 (12), 178 (100), 177 (8), 172 (7), 95 (6), 69 (11), 68 (8), 67 (13). ^{1}**H NMR** (500 MHz, DMSO-d_6) δ 7.97 (bs, 1H), 7.71 (bs, 1H), 4.82 (t, *J* = 7.4 Hz, 1H), 3.90 (s, 3H), 3.76 (ddd, *J* = 14.8, 12.3, 7.0 Hz, 1H), 3.67 (ddd, *J* = 22.7, 14.8, 7.4 Hz, 1H), 2.49 (t, *J* = 2.3 Hz, 2H), 1.68 – 1.54 (m, 2H), 0.92 (t, *J* = 7.4 Hz, 3H). ^{19}**F NMR** (376 MHz, DMSO-d_6) δ -79.8 (d, *J* = 6.5 Hz), -130.0 – -130.3 (m). ^{13}**C NMR** (126 MHz, DMSO-d_6) δ 161.2, 145.6, 129.4, 127.5, 120.4 (qd, *J* = 284.9, 31.2 Hz), 106.9 (dq, *J* = 253.1, 34.1 Hz), 52.8 (d, *J* = 20.5 Hz), 39.2, 27.3, 21.5, 14.0. **IR** (film, ATR) 1674, 1600, 1462, 1421, 1376, 1335, 1301, 1260, 1190, 1149, 1096, 1070, 1040, 973, 951, 895, 876, 727, 686 cm^{-1}. **HRMS** (ESI) $[M+H]^+$ calculated for $C_{11}H_{16}ClF_4N_4O^+$: 331.0943, found: 331.0944.

N-(2-Chloro-2,3,3,3-tetrafluoropropyl)quinolin-8-amine (47)

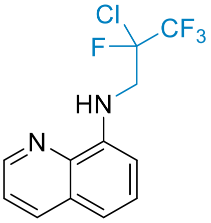

Method B. Using quinolin-8-amine (43.3 mg, 0.30 mmol), Li_2CO_3 (26.6 mg, 0.36 mmol) and iodonium salt (171.8 mg, 0.33 mmol) in MeCN (1.0 mL) (RT, 3 h).

Yield: 60.0 mg (0.21 mmol, 67%) yellow oil. $\mathbf{R_f}$= 0.68 in hexane : ethyl acetate 4:1. **MS** (EI, 70 eV): m/z (%): 294 (4), 292 (12, $[M^+]$), 257 (3), 237 (7), 217 (4), 187 (26), 158 (12), 157 (100), 156 (18), 155 (9), 129 (19), 128 (10), 116 (6), 102 (7), 78 (12), 69 (3). ^{1}**H NMR** (250 MHz, Chloroform-*d*) δ 8.77 (dd, *J* = 4.2, 1.7 Hz, 1H), 8.08 (dd, *J* = 8.3, 1.7 Hz, 1H), 7.54 – 7.33 (m, 2H), 7.17 (dd, *J* = 8.2, 1.2 Hz, 1H), 6.87 (d, *J* = 7.7 Hz, 1H), 6.79 (s, 1H), 4.23 (ddd, *J* = 13.9, 12.6, 6.6 Hz, 1H), 4.09 (ddd, *J* = 22.0, 15.3, 7.0 Hz, 1H). ^{19}**F NMR** (235 MHz, Chloroform-*d*) δ -80.5 (d, *J* = 6.1 Hz), -129.7 (q, *J* = 6.1 Hz). ^{13}**C NMR** (63 MHz, Chloroform-*d*) δ 147.5,

143.3, 138.2, 136.2, 128.7, 127.5, 121.8, 120.9 (qd, *J* = 284.9, 31.0 Hz), 116.0, 107.2 (dq, *J* = 255.8, 34.6 Hz), 105.5 (d, *J* = 2.2 Hz), 48.7 (d, *J* = 22.4 Hz). **IR** (film, ATR) 1618, 1579, 1527, 1484, 1441, 1426, 1385, 1376, 1340, 1324, 1307, 1273, 1214, 1187, 1154, 1135, 1092, 1057, 1025, 969, 943, 891, 869, 839, 818, 805, 790, 746, 733, 720, 680, 662 cm^{-1}. **HRMS** (ESI) $[M+H]^+$ calculated for $C_{12}H_{10}ClF_4N_2^+$: 293.0463, found: 293.0463.

6-Chloro-*N*-(2-chloro-2,3,3,3-tetrafluoropropyl)pyridin-2-amine (48)

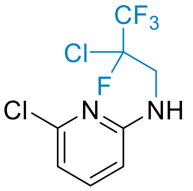

Method B. Using 6-chloropyridin-2-amine (38.6 mg, 0.30 mmol), Li_2CO_3 (26.6 mg, 0.36 mmol) and iodonium salt (171.8 mg, 0.33 mmol) in MeCN (1.0 mL) (RT, 8 h).

Yield: 58.9 mg (0.21 mmol, 71%) yellowish solid. **Mp.** 62-65 °C. **R_f**= 0.58 in hexane : ethyl acetate 4:1. **MS** (EI, 70 eV): m/z (%): 280 (1), 278 (6), 276 (10, $[M^+]$), 243 (4), 241 (14), 221 (5), 171 (6), 143 (32), 141 (100), 114 (7), 112 (19), 105 (15), 92 (9), 69 (5). **^{1}H NMR** (250 MHz, Chloroform-*d*) δ 7.39 (t, *J* = 7.9 Hz, 1H), 6.68 (d, *J* = 7.6 Hz, 1H), 6.41 (d, *J* = 8.2 Hz, 1H), 4.97 (bs, 1H), 4.41 – 3.99 (m, 2H). **^{19}F NMR** (235 MHz, Chloroform-*d*) δ -80.6 (d, *J* = 6.4 Hz), -130.4 (q, *J* = 6.4 Hz). **^{13}C NMR** (63 MHz, Chloroform-*d*) δ 157.2, 149.6, 140.1, 120.6 (qd, *J* = 284.7, 30.9 Hz), 114.0, 106.4 (dq, *J* = 254.6, 35.3 Hz), 106.0, 45.9 (d, *J* = 22.4 Hz). **IR** (film, ATR) 1601, 1571, 1506, 1463, 1450, 1437, 1400, 1376, 1307, 1215, 1193, 1163, 1143, 1117, 1063, 982, 949, 928, 906, 861, 777, 762, 731, 686, 662 cm^{-1}. **HRMS** (EI) $[M]^+$ calculated for $C_8H_6Cl_2F_4N_2^+$: 275.9844, found: 275.9837.

***N*-(2-Chloro-2,3,3,3-tetrafluoropropyl)-1*H*-indazol-6-amine (49)**

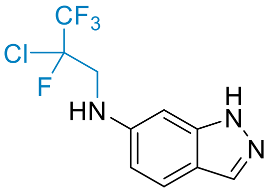

Method B. Using 1*H*-indazol-6-amine (39.9 mg, 0.30 mmol), Li_2CO_3 (26.6 mg, 0.36 mmol) and iodonium salt (171.8 mg, 0.33 mmol) in MeCN (1.0 mL) (RT, 4 h).

Yield: 79.4 mg (0.28 mmol, 94%) blue solid. **Mp.** 105-110 °C. **R_f**= 0.70 in ethyl acetate. **MS** (EI, 70 eV): m/z (%): 283 (5), 281 (17, $[M^+]$), 147 (10), 146 (100), 119 (6), 118 (12), 117 (7), 105 (5), 90 (9), 73 (7), 69 (2). **^{1}H NMR** (250 MHz, Chloroform-*d*) δ 10.21 (bs, 1H), 7.95 (s, 1H), 7.59 – 7.42 (m, 1H), 6.87 – 6.37 (m, 2H), 4.59 (bs, 1H), 4.00 (t, *J* = 14.0 Hz, 1H), 3.85 (dd, *J* = 21.0, 15.2 Hz, 1H). **^{19}F NMR** (235 MHz, Chloroform-*d*) δ -80.5 (d, *J* = 6.1 Hz), -129.9 (q, *J* = 6.2 Hz). **^{13}C NMR** (63 MHz, Chloroform-*d*) δ 146.4, 141.9, 134.3, 121.9, 120.7 (qd, *J* = 284.9, 31.2 Hz), 117.3, 112.6, 106.9 (dq, *J* = 255.4, 34.8 Hz), 90.0 (d, *J* = 1.8 Hz), 49.0 (d, *J* = 22.2 Hz). **IR** (film, ATR) 1635, 1590, 1516, 1473, 1363, 1324, 1307, 1273, 1247, 1215, 1191, 1137, 1100, 1068, 975, 945, 891, 850, 803, 759, 731, 664 cm^{-1}. **HRMS** (ESI) $[M+H]^+$ calculated for $C_{10}H_9ClF_4N_3^+$: 282.0416, found: 282.0418.

***N*,1-Bis(2-chloro-2,3,3,3-tetrafluoropropyl)-1*H*-indazol-6-amine (50)**

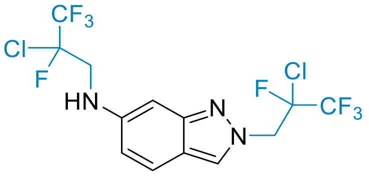

Method B. Using 1*H*-indazol-6-amine (39.9 mg, 0.30 mmol), Li_2CO_3 (53.2 mg, 0.72 mmol) and iodonium salt (343.6 mg, 0.66 mmol) in MeCN (1.0 mL) (RT, 4 h).

Yield: 74.6 mg (0.17 mmol, 58%) purple oil. **R_f**= 0.83 in hexane : ethyl acetate 2:1. **MS** (EI, 70 eV): m/z (%): 433 (2), 432 (2), 431 (12), 430 (3), 429 (19, $[M^+]$), 394 (6), 296 (33), 295 (15), 294 (100), 258 (10), 158 (11), 145 (7), 130 (7), 117 (8), 88 (9), 80 (12), 69 (13). **^{1}H NMR** (400 MHz, Chloroform-*d*) δ 7.90 (s, 1H), 7.49 (d, *J* = 9.0 Hz, 1H), 6.76 (d, *J* = 2.2 Hz, 1H), 6.63 (dd, *J* = 9.0, 2.0 Hz, 1H), 5.10 (dd, *J* = 15.1, 12.7 Hz, 1H), 4.95 (dd, *J* = 22.5, 15.1

Hz, 1H), 4.21 (s, 1H), 4.04 (dd, *J* = 15.1, 12.2 Hz, 1H), 3.90 (dd, *J* = 21.5, 15.1 Hz, 1H). **^{19}F NMR** (376 MHz, Chloroform-*d*) δ -80.6 (d, *J* = 6.1 Hz), -80.8 (d, *J* = 6.2 Hz), -128.2 (q, *J* = 6.3 Hz), -129.8 – -130.1 (m). **^{13}C NMR** (101 MHz, Chloroform-*d*) δ 150.7, 145.6, 125.2 (d, *J* = 1.8 Hz), 121.4, 120.7 (qd, *J* = 284.8, 31.0 Hz), 120.3 (qd, *J* = 285.4, 31.0 Hz), 117.7, 117.3, 106.8 (dq, *J* = 255.3, 34.9 Hz), 104.4 (dq, *J* = 256.8, 36.3 Hz), 93.5 (d, *J* = 1.8 Hz), 56.6 (d, *J* = 22.1 Hz), 48.8 (d, *J* = 22.2 Hz). **IR** (film, ATR) 1645, 1566, 1536, 1495, 1369, 1339, 1301, 1275, 1190, 1163, 1134, 1067, 1048, 999, 973, 947, 891, 854, 805, 761, 731, 705, 668 cm^{-1}. **HRMS** (ESI) [M+H]$^+$ calculated for $C_{13}H_{10}Cl2F_8N_3^+$: 430.0119, found: 430.0121.

2-(2-Chloro-2,3,3,3-tetrafluoropropyl)-5-phenyl-2*H*-tetrazole (51 and 51z)

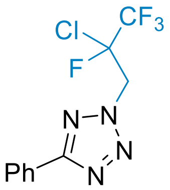

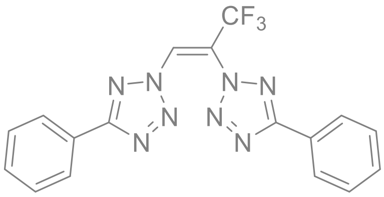

Inseparable side products in 30%

Method B. Using 5-phenyl-2*H*-tetrazole (43.8 mg, 0.30 mmol), Li_2CO_3 (26.6 mg, 0.36 mmol) and iodonium salt (171.8 mg, 0.33 mmol) in MeCN (1.0 mL) (RT, 24 h).

Yield: 37.7 mg, white solid, mixture of two compound. **R_f**= 0.51 in hexane : ethyl acetate 4:1.

Major product (**51**): **MS** (EI, 70 eV): m/z (%): (-N_2) 268 (5), 266 (13), 230 (2), 132 (9), 131 (100), 105 (8), 104 (100), 103 (24), 89 (11), 77 (39), 76 (16), 69 (11), 63 (20). **^{1}H NMR** (500 MHz, DMSO-*d*$_6$) δ 8.13 – 8.07 (m, 2H), 7.62 – 7.57 (m, 3H), 6.19 – 6.05 (m, 2H). **^{19}F NMR** (376 MHz, DMSO-*d*$_6$) δ -79.9 (d, *J* = 6.5 Hz), -127.2 – -127.4 (m). **^{13}C NMR** (126 MHz, DMSO-*d*$_6$) δ 164.8, 131.1, 129.5, 126.6, 126.1, 119.8 (qd, *J* = 285.3, 31.0 Hz), 103.4 (dq, *J* = 256.6, 36.8 Hz), 54.9 (d, *J* = 22.4 Hz). **HRMS** (ESI) [M+H]$^+$ calculated for $C_{10}H_8ClF_4N_4^+$: 295.0368, found: 295.0368.

Minor compound (**51z**): **^{1}H NMR** (500 MHz, DMSO-d6) δ 9.83 (s, 1H), 8.21 – 8.17 (m, 2H), 7.74 – 7.68 (m, 2H), 7.67 – 7.61 (m, 3H), 7.52 (t, *J* = 7.5 Hz, 1H), 7.42 (t, *J* = 7.7 Hz, 2H).**^{19}F NMR** (376 MHz, DMSO-*d*$_6$) δ -65.7 (d, *J* = 1.1 Hz). **^{13}C NMR** (126 MHz, DMSO-*d*$_6$) δ 165.8, 164.9, 132.0, 131.8 (q, *J* = 4.6 Hz), 131.6, 129.6, 129.4, 126.9, 126.6, 125.6, 124.7, 120.1 (q, *J* = 273.5 Hz), 114.4 (q, *J* = 39.1 Hz).

1-(2-Chloro-2,3,3,3-tetrafluoropropyl)-1*H*-benzo[d][1,2,3]triazole (52)

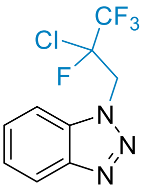

Method B. Using 1*H*-benzo[d][1,2,3]triazole (35.7 mg, 0.30 mmol), Li_2CO_3 (26.6 mg, 0.36 mmol) and iodonium salt (171.8 mg, 0.33 mmol) in MeCN (1.0 mL) (RT, 24 h).

Yield: 37.6 mg (0.14 mmol, 47%) white solid. **Mp.** 124-128 °C. **R_f**= 0.50 in hexane : ethyl acetate 4:1. **MS** (EI, 70 eV): m/z (%): 269 (6), 267 (20, [M$^+$]), 239 (8), 135 (8), 132 (11), 104 (11), 91 (100), 90 (22), 85 (8), 77 (44), 76 (11), 69 (23), 64 (25), 63 (25), 51 (26). **^{1}H NMR** (250 MHz, Chloroform-*d*) δ 8.10 (d, *J* = 8.3 Hz, 1H), 7.67 – 7.49 (m, 2H), 7.50 – 7.33 (m, 1H), 5.49 – 5.18 (m, 2H). **^{19}F NMR** (235 MHz, Chloroform-*d*) δ -80.7 (d, *J* = 6.1 Hz), -126.9 (q, *J* = 6.0 Hz). **^{13}C NMR** (63 MHz, Chloroform-*d*) δ 146.1, 133.7, 128.7, 124.7, 120.5, 120.2 (qd, *J* = 285.2, 30.6 Hz), 109.6 (d, *J* = 3.5 Hz), 104.8 (dq, *J* = 257.2, 36.5 Hz), 52.0 (d, *J* = 22.9 Hz). **IR** (film, ATR) 2988, 1456, 1435, 1305, 1273, 1236, 1214, 1200, 1167, 1158, 1132, 1120, 1107, 1023, 1005, 973, 925, 887, 783, 768, 742, 721 cm^{-1}. **HRMS** (ESI) [M+H]$^+$ calculated for $C_9H_7ClF_4N_3^+$: 268.0259, found: 268.0261.

1-(2-Chloro-3,3,3-trifluoroprop-1-en-1-yl)-1*H*-benzo[d][1,2,3]triazole (52x)

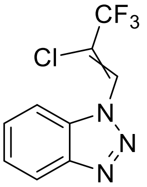

8.4 mg side product, yellow oil. **MS** (EI, 70 eV): m/z (%): 249 (6), 247 (19, [M^+]), 200 (8), 199 (19), 185 (10), 184 (100), 169 (22), 164 (36), 150 (14), 134 (28), 121 (12), 114 (9), 91 (9), 90 (24), 89 (38), 76 (14), 75 (28), 74 (15), 69 (44), 64 (31), 63 (50), 62 (16), 50 (25). **^{1}H NMR** (500 MHz, DMSO-d_6) δ 8.65 (q, *J* = 1.2 Hz, 1H), 8.25 (dt, *J* = 8.4, 1.0 Hz, 1H), 7.78 (dt, *J* = 8.4, 1.1 Hz, 1H), 7.76 – 7.71 (m, 1H), 7.61 – 7.53 (m, 1H). **^{19}F NMR** (376 MHz, DMSO-d_6) δ -66.2. **^{13}C NMR** (126 MHz, DMSO-d_6) δ 144.5, 135.6 (q, *J* = 4.8 Hz), 132.9, 129.9, 125.4, 125.0 (q, *J* = 36.8 Hz), 120.2 (q, *J* = 274.5 Hz), 120.1, 110.1.**HRMS** (ESI) [M+H]$^+$ calculated for $C_9H_5ClF_3N_3^+$: 248.0202, found: 248.0198.

1-(2-(2*H*-benzo[d][1,2,3]triazol-2-yl)-3,3,3-trifluoroprop-1-en-1-yl)-1*H*-benzo[d][1,2,3]triazole (52z)

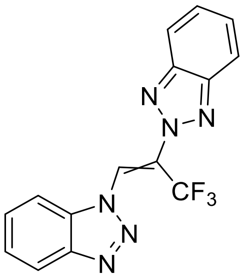

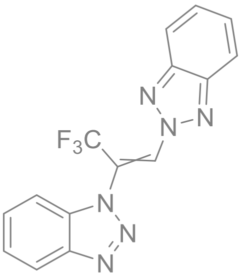

This isomer can not be exluded.

5.6 mg side product, white solid. **MS** (EI, 70 eV): m/z (%): 331 (8), 330 (44, [M^+]), 302 (25), 301 (18), 274 (21), 273 (100), 253 (18), 233 (23), 207 (13), 206 (24), 205 (74), 204 (13), 203 (16), 184 (58), 183 (26), 180 (15), 179 (44), 178 (26), 177 (16), 164 (12), 154 (31), 153 (15), 152 (18), 151 (22), 140 (17), 134 (34), 129 (36), 121 (13), 108 (14), 103 (10), 102 (24), 91 (12), 90 (27), 77 (47), 76 (64), 75 (28), 64 (36), 63 (37), 62 (16), 52 (15) 51 (23). **^{1}H NMR** (500 MHz, DMSO-d_6) δ 9.34 (d, *J* = 1.2 Hz, 1H), 8.26 (dt, *J* = 8.3, 1.0 Hz, 1H), 7.68 – 7.61 (m, 3H), 7.59 – 7.56 (m, 1H), 7.54 – 7.49 (m, 1H), 7.45 – 7.41 (m, 2H). **^{19}F NMR** (376 MHz, DMSO-d_6) δ -66.0. **^{13}C NMR** (126 MHz, DMSO-d_6) δ 145.6, 145.5, 134.7 (q, *J* = 4.7 Hz), 134.5, 130.5, 129.9, 125.4, 121.7 (q, *J* = 274.5 Hz), 120.4, 118.8, 114.6 (q, *J* = 36.9 Hz), 110.6. **HRMS** (ESI) [M+H]$^+$ calculated for $C_{15}H_9F_3N_6^+$: 331.0919, found: 331.0915.

1-(2-Chloro-2,3,3,3-tetrafluoropropyl)-1*H*-indazole (53)

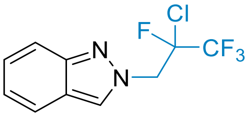

Method B. Using 1*H*-indazole (35.4 mg, 0.30 mmol), Li_2CO_3 (26.6 mg, 0.36 mmol) and iodonium salt (171.8 mg, 0.33 mmol) in MeCN (1.0 mL) (RT, 28 h).

Yield: 60.4 mg (0.23 mmol, 76%) white solid. **Mp.** 91-94 °C. **R_f**= 0.43 in hexane : ethyl acetate 4:1. **MS** (EI, 70 eV): m/z (%): 268 (29), 267 (10), 266 (87, [M^+]), 231 (7), 132 (9), 131 (100), 104 (27), 103 (41), 102 (9), 89 (17), 77 (52), 69 (15), 63 (28), 51 (14). **^{1}H NMR** (250 MHz, Chloroform-*d*) δ 8.05 (s, 1H), 7.74 (d, *J* = 8.8 Hz, 1H), 7.67 (d, *J* = 8.5 Hz, 1H), 7.37 – 7.28 (m, 1H), 7.17 – 7.07 (m, 1H), 5.19 (dd, *J* = 15.0, 12.8 Hz, 1H), 5.03 (dd, *J* = 22.4, 15.0 Hz, 1H). **^{19}F NMR** (235 MHz, Chloroform-*d*) δ -80.8 (d, *J* = 6.1 Hz), -128.1 (q, *J* = 6.1 Hz). **^{13}C NMR** (63 MHz, Chloroform-*d*) δ 149.3, 127.1, 125.0 (d, *J* = 1.6 Hz), 122.8, 122.5, 120.4, 119.4 (qd, *J* = 285.4, 30.6 Hz), 117.9, 104.3 (dq, *J* = 257.2, 36.6 Hz), 57.0 (d, *J* = 21.9 Hz). **IR** (film, ATR) 2990, 2973, 2947, 1633, 1519, 1370, 1307, 1214, 1187, 1163, 1148, 1128, 1050, 1008, 984, 971, 917, 891, 789, 755, 744, 729, 703, 669 cm^{-1}. **HRMS** (ESI) [M+H]$^+$ calculated for $C_{10}H_8ClF_4N_2^+$: 267.0307, found: 267.0308.

1-(2-Chloro-2,3,3,3-tetrafluoropropyl)-3,5-diphenyl-1*H*-pyrazole (54 and 54y)

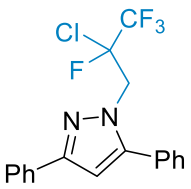

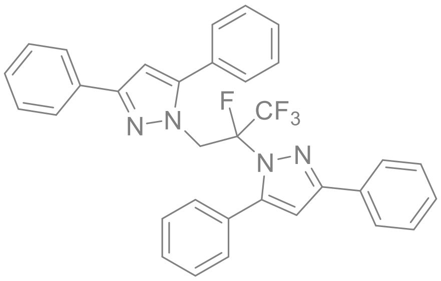

Inseparable side products in 25%

Method B. Using 3,5-diphenyl-1*H*-pyrazole (66.1 mg, 0.30 mmol), Li_2CO_3 (26.6 mg, 0.36 mmol) and iodonium salt (171.8 mg, 0.33 mmol) in MeCN (1.0 mL) (RT, 48 h).

Yield: 49.8 mg, yellow oil. **R$_f$**= 0.55 in hexane : ethyl acetate 4:1.

Major product (**54**): **MS** (EI, 70 eV): m/z (%): 370 (12), 369 (7), 368 (35, [M$^+$]), 333 (3), 234 (18), 233 (100), 204 (4), 191 (7), 189 (13), 165 (4), 130 (22), 116 (6), 104 (12), 103 (9), 77 (31), 69 (4). **^{1}H NMR** (500 MHz, DMSO-d_6) δ 7.90 – 7.87 (m, 2H), 7.59 – 7.29 (m), 7.01 (s, 1H), 5.24 (dd, *J* = 16.0, 14.2 Hz, 1H), 5.08 (dd, *J* = 22.5, 16.0 Hz, 1H). **^{19}F NMR** (376 MHz, DMSO-d_6) δ -80.4 (d, *J* = 6.4 Hz), -125.7 – -126.5 (m). **HRMS** (ESI) [M+H]$^+$ calculated for $C_{18}H_{14}ClF_4N_2^+$: 369.0776, found: 369.0777.

Minor compound (**54y**): **^{1}H NMR** (500 MHz, DMSO-d_6) δ 7.82 – 7.79 (m, 2H), 7.70 – 7.67 (m, 2H), 7.59 – 7.29 (m), 7.15 – 7.10 (m, 2H), 6.91 (s, 1H), 6.89 (s, 1H), 5.60 (dd, *J* = 26.1, 15.5 Hz, 1H), 5.32 (dd, *J* = 15.5, 12.2 Hz, 1H). **^{19}F NMR** (376 MHz, DMSO-d_6) δ -79.4 (d, *J* = 5.3 Hz), -150.5 – -151.0 (m).

1-(2-Chloro-2,3,3,3-tetrafluoropropyl)-3-phenyl-1*H*-pyrazole (55)

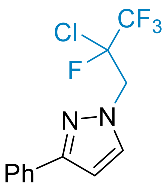

Method B. Using 3-phenyl-1*H*-pyrazole (43.3 mg, 0.30 mmol), Li_2CO_3 (26.6 mg, 0.36 mmol) and iodonium salt (171.8 mg, 0.33 mmol) in MeCN (1.0 mL) (RT, 24 h).

Yield: 76.4 mg (0.26 mmol, 87%) colorless solid. **Mp.** 33-36 °C. **R$_f$**= 0.57 in hexane : ethyl acetate 4:1. **MS** (EI, 70 eV): m/z (%): 294 (10), 292 (31, [M$^+$]), 257 (4), 158 (11), 157 (100), 130 (19), 118 (11), 115 (8), 103 (8), 77 (34), 69 (5), 51 (12). **^{1}H NMR** (250 MHz, Chloroform-*d*) δ 7.84 (d, *J* = 7.1 Hz, 2H), 7.55 (s, 1H), 7.51 – 7.28 (m, 3H), 6.67 (d, *J* = 2.5 Hz, 1H), 5.07 – 4.63 (m, 2H). **^{19}F NMR** (235 MHz, Chloroform-*d*) δ -80.7 (d, *J* = 6.2 Hz), -128.8 (q, *J* = 6.3 Hz). **^{13}C NMR** (63 MHz, Chloroform-*d*) δ 152.9, 132.9, 132.5 (d, *J* = 1.7 Hz), 128.8, 128.2, 126.0, 120.3 (qd, *J* = 285.0, 30.6 Hz), 104.8, 104.6 (dq, *J* = 256.4, 36.2 Hz), 55.8 (d, *J* = 22.3 Hz). **IR** (film, ATR) 1532, 1506, 1460, 1411, 1359, 1303, 1238, 1215, 1193, 1161, 1135, 1102, 1077, 1059, 1029, 999, 971, 947, 917, 889, 833, 811, 751, 727, 718, 693, 680, 662 cm^{-1}. **HRMS** (ESI) [M+H]$^+$ calculated for $C_{12}H_{10}ClF_4N_2^+$: 293.0463, found: 293.0462.

1-(2-Chloro-2,3,3,3-tetrafluoropropyl)-1*H*-pyrazole (56)

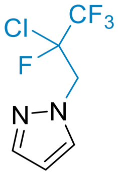

Method B. Using 1*H*-pyrazole (34.0 mg, 0.50 mmol), Li_2CO_3 (44.3 mg, 0.60 mmol) and iodonium salt (286.3 mg, 0.55 mmol) in MeCN (1.0 mL) (RT, 24 h).

Yield: 61.8 mg (0.29 mmol, 57%) yellow, volatile oil. **R$_f$**= 0.46 in hexane : ethyl acetate 4:1. **MS** (EI, 70 eV): m/z (%): 218 (6), 216 (18, [M$^+$]), 181 (4), 161 (4), 81 (100), 69 (15), 54 (17), 53 (16). **^{1}H NMR** (500 MHz, DMSO-d_6) δ 7.87 (d, *J* = 2.3 Hz, 1H), 7.58 (dd, *J* = 1.9, 0.6 Hz, 1H), 6.37 – 6.36 (m, 1H), 5.31 – 5.13 (m, 2H). **^{19}F NMR** (376 MHz, DMSO-d_6) δ -79.9 (d, *J* = 6.6 Hz), -

127.7 – -128.0 (m). **^{13}C NMR** (126 MHz, DMSO-*d*$_6$) δ 140.7, 132.9 (d, *J* = 1.3 Hz), 120.5 (qd, *J* = 285.1, 31.3 Hz), 106.8, 105.2 (dq, *J* = 255.1, 35.8 Hz), 54.5 (d, *J* = 21.6 Hz).). **IR** (film, ATR) 1395, 1301, 1219, 1193, 1134, 1093, 1055, 1033, 981, 951, 888, 753, 712, 668 cm^{-1}. **HRMS** (ESI) [M+H]$^+$ calculated for $C_6H_6ClF_4N_2^+$: 217.0150, found: 217.015.

3-(2-Chloro-2,3,3,3-tetrafluoropropyl)-1*H*-indole (57)

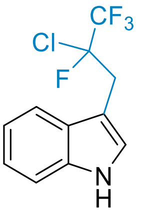

Method B. Using 1*H*-indole (35.1 mg, 0.30 mmol), Li_2CO_3 (26.6 mg, 0.36 mmol) and iodonium salt (171.8 mg, 0.33 mmol) in MeCN (1.0 mL) (RT, 48 h).

Yield: 42.8 mg (0.16 mmol, 54%) green solid. **Mp.** 93-96 °C. **R$_f$**= 0.59 in DCM : hexane 1:1. **MS** (EI, 70 eV): m/z (%): 267 (5), 265 (15, [M$^+$]), 230 (3), 161 (5), 131 (11), 130 (100), 103 (5), 77 (5), 69 (1). **^{1}H NMR** (500 MHz, DMSO-*d*$_6$) δ 11.21 (s, 1H), 7.56 (d, *J* = 8.0 Hz, 1H), 7.42 – 7.37 (m, 2H), 7.11 (td, *J* = 7.0, 1.1 Hz, 1H), 7.03 (td, *J* = 7.3, 1.0 Hz, 1H), 3.85 (dd, *J* = 15.5, 11.5 Hz, 1H), 3.69 (dd, *J* = 32.0, 15.5 Hz, 1H). **^{19}F NMR** (376 MHz, DMSO-*d*$_6$) δ -80.5 (d, *J* = 6.4 Hz), -122.5 – -122.8 (m). **^{13}C NMR** (126 MHz, DMSO-*d*$_6$) δ 136.0, 127.6, 126.6, 121.2, 121.0 (qd, *J* = 284.7, 32.1 Hz), 119.0, 118.6 (d, *J* = 2.2 Hz), 111.6, 107.9 (dq, *J* = 250.5, 34.4 Hz), 103.1 (d, *J* = 2.0 Hz), 31.9 (d, *J* = 21.7 Hz). **IR** (film, ATR) 1458, 1425, 1339, 1309, 1290, 1260, 1190, 1130, 1100, 1074, 1003, 970, 940, 869, 746, 712, 675 cm^{-1}. **HRMS** (EI) [M]$^+$ calculated for $C_{11}H_8ClF_4N^+$: 265.0281, found: 265.0277.

5-Bromo-3-(2-chloro-2,3,3,3-tetrafluoropropyl)-2-methyl-1*H*-indole (58)

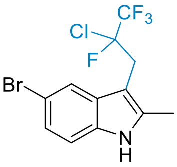

Method B. Using 5-bromo-2-methyl-1*H*-indole (63.0 mg, 0.30 mmol), Li_2CO_3 (26.6 mg, 0.36 mmol) and iodonium salt (171.8 mg, 0.33 mmol) in MeCN (1.0 mL) (RT, 48 h).

Yield: 57.7 mg (0.16 mmol, 54%) yellow solid. **Mp.** 163-167 °C. **R$_f$**= 0.70 in hexane : ethyl acetate 1:1. **MS** (EI, 70 eV): m/z (%): 359 (23), 357 (18, [M$^+$]), 225 (11), 224 (98), 223 (16), 222 (100), 221 (6), 172 (6), 144 (8), 143 (36), 142 (9), 115 (10), 69 (5). **^{1}H NMR** (250 MHz, Chloroform-*d*) δ 8.00 (bs, 1H), 7.65 (d, *J* = 1.9 Hz, 1H), 7.24 (dd, *J* = 8.5, 1.9 Hz, 1H), 7.14 (d, *J* = 8.4 Hz, 1H), 3.63 (dd, *J* = 15.5, 10.6 Hz, 1H), 3.42 (dd, *J* = 31.3, 15.4 Hz, 1H), 2.41 (s, 3H). **^{19}F NMR** (235 MHz, Chloroform-*d*) δ -81.8 (d, *J* = 6.1 Hz), -123.4 (q, *J* = 6.1 Hz). **^{13}C NMR** (63 MHz, Chloroform-*d*) δ 136.4, 133.8, 131.0, 124.5, 121.2 (qd, *J* = 284.6, 31.7 Hz), 121.1 (d, *J* = 2.9 Hz), 113.4, 111.9, 108.2 (dq, *J* = 252.9, 34.9 Hz), 101.5, 31.8 (d, *J* = 22.3 Hz), 12.2 (d, *J* = 1.8 Hz). **IR** (film, ATR) 1581, 1469, 1428, 1294, 1253, 1212, 1190, 1134, 1052, 992, 970, 921, 865, 798, 723 cm^{-1}. **HRMS** (EI) [M]$^+$ calculated for $C_{12}H_9BrClF_4N^+$: 356.9543, found: 356.9544.

4-Bromo-3-(2-chloro-2,3,3,3-tetrafluoropropyl)-2-ethyl-1*H*-indole (59)

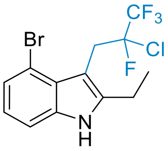

Method A. Using 4-bromo-2-ethyl-1*H*-indole (67.2 mg, 47.8 µL, 0.30 mmol), Li_2CO_3 (26.6 mg, 0.36 mmol) and iodonium salt (171.8 mg, 0.33 mmol) in MeCN (1.0 mL) (RT, 48 h).

Yield: 15.5 mg (0.04 mmol, 14%) yellow oil. **R$_f$**= 0.81 in hexane : ethyl acetate 1:1. **MS** (EI, 70 eV): m/z (%): 373 (20), 371(15, [M$^+$]), 239 (11), 238 (95), 237 (14), 236 (100), 223 (11), 221 (10), 172 (6), 158 (6), 157 (8), 156 (12), 155 (6), 154 (8), 115 (6), 69 (4). **^{1}H NMR** (500 MHz, DMSO-*d*$_6$) δ 11.55 (s, 1H), 7.36 (dd, *J* = 8.1, 0.9 Hz, 1H), 7.18 (dd, *J* = 7.6, 0.9 Hz, 1H), 6.95 (t, *J* = 7.8 Hz, 1H), 4.12 – 3.96 (m, 2H), 2.76 (q, *J* = 7.6 Hz, 2H), 1.27 (t, *J* = 7.6 Hz, 3H). **^{19}F NMR** (376 MHz, DMSO-*d*$_6$) δ -80.9 (d, *J* = 6.7 Hz), -121.7 – -122.1 (m). **^{13}C NMR** (126 MHz, DMSO-*d*$_6$) δ 143.4, 137.0, 125.6, 123.8, 121.6, 121.0 (qd,

J = 284.9, 32.1 Hz), 112.0, 110.8, 108.1 (dq, J = 251.6, 34.1 Hz), 98.7, 29.5 (d, J = 20.4 Hz), 19.2 (d, J = 2.6 Hz), 13.5. **HRMS** (EI) $[M]^+$ calculated for $C_{13}H_{11}BrClF_4N^+$: 370.9700, found: 370.9691.

3-(2-Chloro-2,3,3,3-tetrafluoropropyl)-1-methyl-1*H*-indole (60)

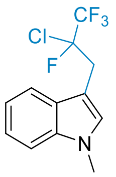

Method A. Using 1-methyl-1*H*-indole (39.4 mg, 37.5 µL, 0.30 mmol), Li_2CO_3 (26.6 mg, 0.36 mmol) and iodonium salt (171.8 mg, 0.33 mmol) in MeCN (1.0 mL) (RT, 48 h).

Yield: 36.2 mg (0.13 mmol, 43%) green oil. $\mathbf{R_f}$= 0.54 in hexane : ethyl acetate 4:1. **MS** (EI, 70 eV): m/z (%): 281 (5), 279 (15, $[M^+]$), 244 (4), 175 (5), 145 (11), 144 (100), 143 (8), 133 (3), 128 (4), 115 (4), 102 (4), 77 (5), 69 (2). ^{1}**H NMR** (500 MHz, DMSO-d_6) δ 7.57 (dt, J = 7.9, 1.1 Hz, 1H), 7.44 (dt, J = 8.2, 0.9 Hz, 1H), 7.39 (s, 1H), 7.18 (td, J = 7.1, 1.1 Hz, 1H), 7.07 (td, J = 7.1, 1.1 Hz, 1H), 3.84 (dd, J = 15.5, 11.4 Hz, 1H), 3.80 (s, 3H), 3.69 (dd, J = 32.0, 15.5 Hz, 1H). ^{19}**F NMR** (376 MHz, DMSO-d_6) δ -80.5 (d, J = 6.5 Hz), -122.6 – -122.9 (m). ^{13}**C NMR** (126 MHz, DMSO-d_6) δ 136.4, 130.6, 127.9, 121.3, 120.9 (qd, J = 284.7, 32.2 Hz), 119.1, 118.8 (d, J = 2.2 Hz), 109.8, 107.7 (dq, J = 250.8, 34.7 Hz), 102.3 (d, J = 2.1 Hz), 32.5, 31.7 (d, J = 21.7 Hz). **IR** (film, ATR) 1473, 1428, 1380, 1331, 1294, 1260, 1186, 1160, 1134, 1067, 1014, 999, 958, 936, 873, 738, 686 cm^{-1}. **HRMS** (EI) $[M]^+$ calculated for $C_{12}H_{10}ClF_4N^+$: 279.0438, found: 279.0431.

12. Unsuccessful attempts

Using the following substrates, the products could not be isolated or detected at all. The reactions monitored by GC-MS and TLC.

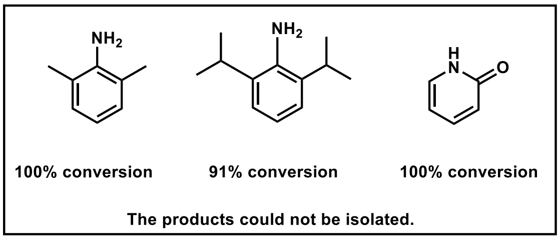

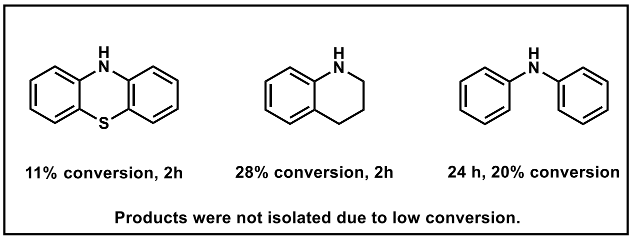

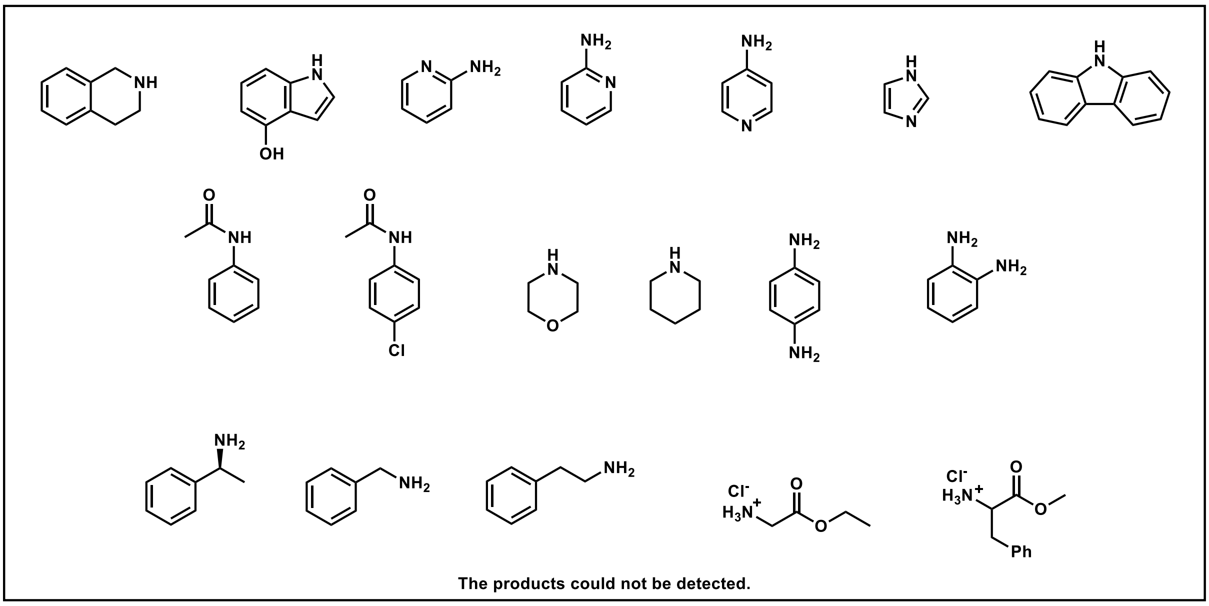

13. References

[1] P. A. Krasutsky, I. V. Kolomitsyn, R. M. Carlson, *Org. Lett.* **2001**, *3*, 2997-2999.
[2] P. A. Krasutsky, I. V. Kolomitsyn, P. Kiprof, R. M. Carlson, N. A. Sydorenko, A. A. Fokin, *J. Org. Chem.* **2001**, *66*, 1701-1707.
[3] G. M. Sheldrick, *Acta Crystallogr., Sect. A: Found. Crystallogr.* **2008**, *64*, 112-122.
[4] S. P. Westrip, *J. Appl. Cryst.* **2010**, *43*, 920-925.
[5] C. F. Macrae, P. R. Edgington, P. McCabe, E. Pidcock, G. P. Shields, R. Taylor, M. Towler, J. van der Streek, *J. Appl. Cryst.* **2006**, *39*, 453-457.
[6] A. L. Spek, *J. Appl. Cryst.* **2003**, *36*, 7-13.
[7] M. J. Frisch, G. W. Trucks, H. B. Schlegel, G. E. Scuseria, M. A. Robb, J. R. Cheeseman, G. Scalmani, V. Barone, G. A. Petersson, H. Nakatsuji, X. Li, M. Caricato, A. V. Marenich, J. Bloino, B. G. Janesko, R. Gomperts, B. Mennucci, H. P. Hratchian, J. V. Ortiz, A. F. Izmaylov, J. L. Sonnenberg, D. Williams-Young, F. Ding, F. Lipparini, F. Egidi, J. Goings, B. Peng, A. Petrone, T. Henderson, D. Ranasinghe, V. G. Zakrzewski, J. Gao, N. Rega, G. Zheng, W. Liang, M. Hada, M. Ehara, K. Toyota, R. Fukuda, J. Hasegawa, M. Ishida, T. Nakajima, Y. Honda, O. Kitao, H. Nakai, T. Vreven, K. Throssell, J. A. Montgomery Jr., J. E. Peralta, F. Ogliaro, M. J. Bearpark, J. J. Heyd, E. N. Brothers, K. N. Kudin, V. N. Staroverov, T. A. Keith, R. Kobayashi, J. Normand, K. Raghavachari, A. P. Rendell, J. C. Burant, S. S. Iyengar, J. Tomasi, M. Cossi, J. M. Millam, M. Klene, C. Adamo, R. Cammi, J. W. Ochterski, R. L. Martin, K. Morokuma, O. Farkas, J. B. Foresman, D. J. Fox, Wallingford, CT, **2016**.
[8] J.-D. Chai, M. Head-Gordon, *Phys. Chem. Chem. Phys.* **2008**, *10*, 6615-6620.
[9] W. J. Hehre, R. Ditchfield, J. A. Pople, *J. Chem. Phys.* **1972**, *56*, 2257-2261.
[10] W. R. Wadt, P. J. Hay, *J. Chem. Phys.* **1985**, *82*, 284-298.
[11] J. Tomasi, B. Mennucci, R. Cammi, *Chem. Rev.* **2005**, *105*, 2999-3094.
[12] L. E. Roy, P. J. Hay, R. L. Martin, *J. Chem. Theory Comput.* **2008**, *4*, 1029-1031.
[13] J. R. Pliego Jr, J. M. Riveros, *WIREs Comput. Mol. Sci.* **2020**, *10*, e1440.
[14] C. Xu, Y. Feng, F. Li, J. Han, Y.-M. He, Q.-H. Fan, *Organometallics* **2019**, *38*, 3979-3990.
[15] Á. Mészáros, A. Székely, A. Stirling, Z. Novák, *Angew. Chem. Int. Ed.* **2018**, *57*, 6643-6647.
[16] Y. Zhou, C. Yao, R. Ni, G. Yang, *Synth. Commun.* **2010**, *40*, 2624-2632.
[17] B. Varga, B. L. Tóth, F. Béke, J. T. Csenki, A. Kotschy, Z. Novák, *Org. Lett.* **2021**, *23*, 4925-4929.
[18] Z. Gonda, Z. Novák, *Chem. Eur. J.* **2015**, *21*, 16801-16806.

14. NMR spectra

^{1}H NMR (250 MHz, Chloroform-*d*) δ 7.29 (dd, *J* = 7.7, 1.6 Hz, 1H), 7.11 (td, *J* = 7.7, 1.6 Hz, 1H), 6.82 – 6.55 (m, 2H), 4.14 (s, 2H), 2.51 (t, *J* = 6.8 Hz, 2H), 1.78 – 1.35 (m, 4H), 1.00 (t, *J* = 7.1 Hz, 3H).

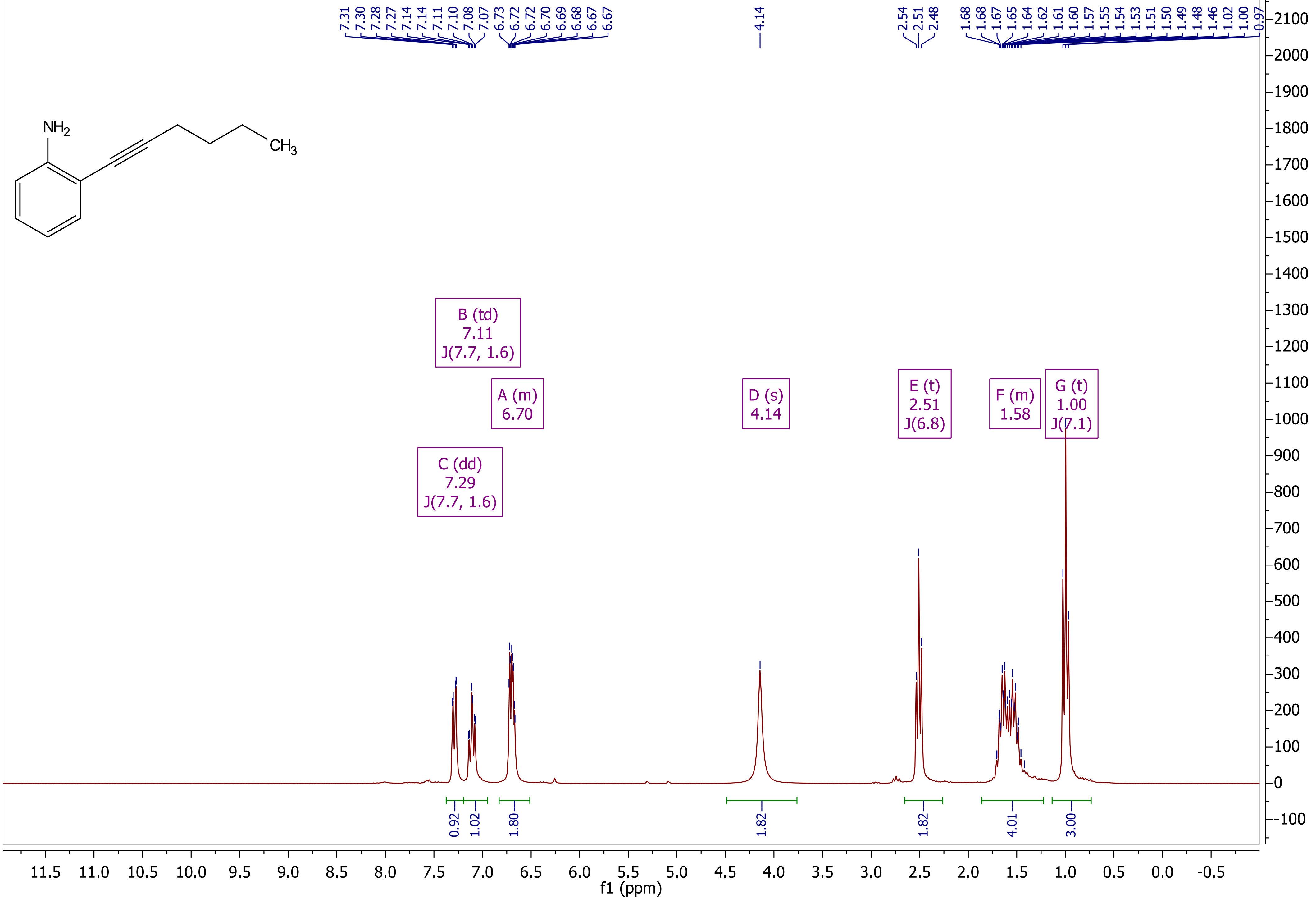

^{13}C NMR (63 MHz, Chloroform-*d*) δ 147.7, 132.0, 128.8, 117.9, 114.2, 109.0, 95.8, 77.1, 31.1, 22.1, 19.4, 13.7.

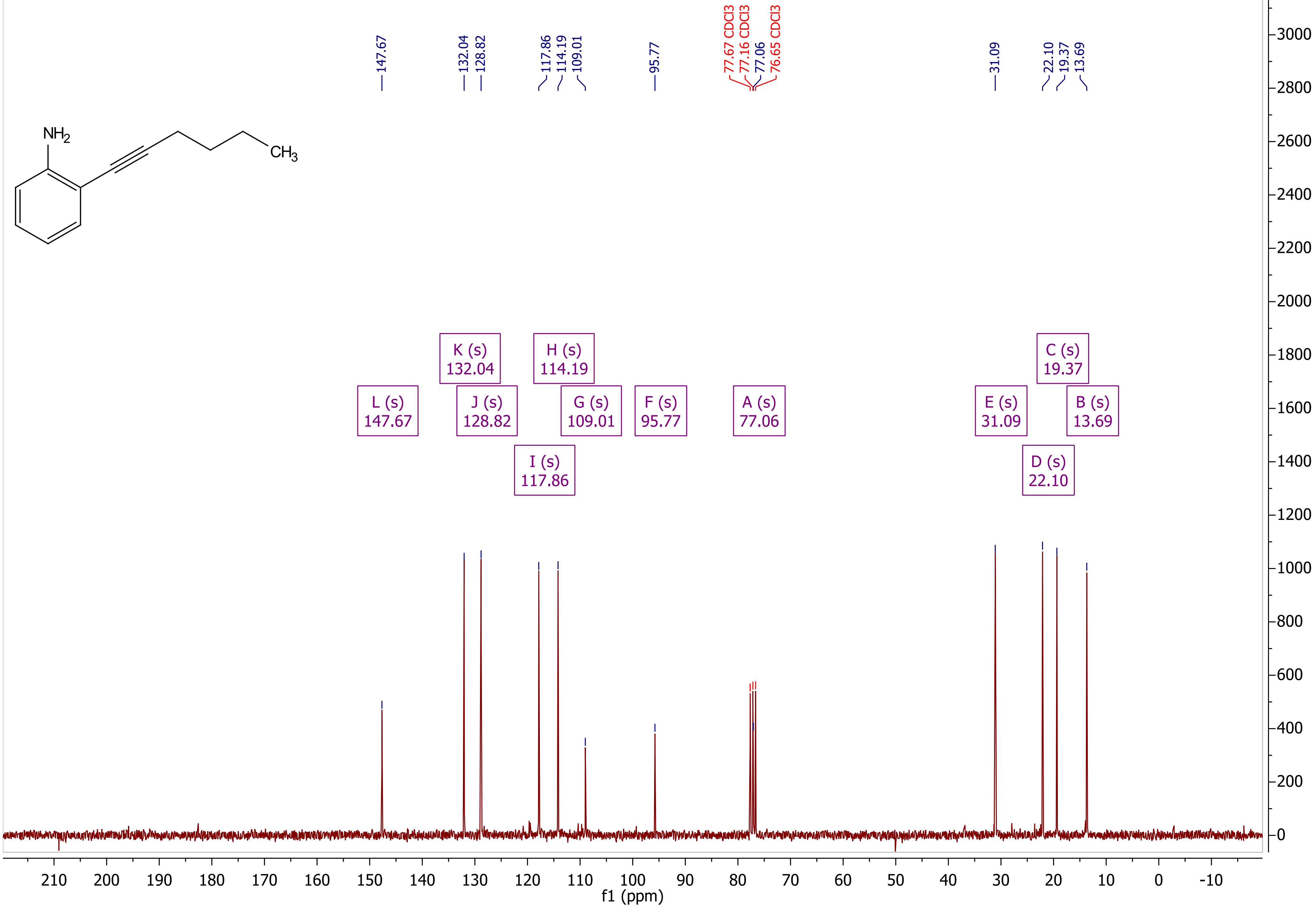

^{1}H NMR (250 MHz, Chloroform-*d*) δ 7.02 (t, *J* = 8.0 Hz, 1H), 6.35 – 6.25 (m, 2H), 6.22 (t, *J* = 2.3 Hz, 1H), 3.59 (s, 2H), 1.02 (s, 9H), 0.22 (s, 6H).

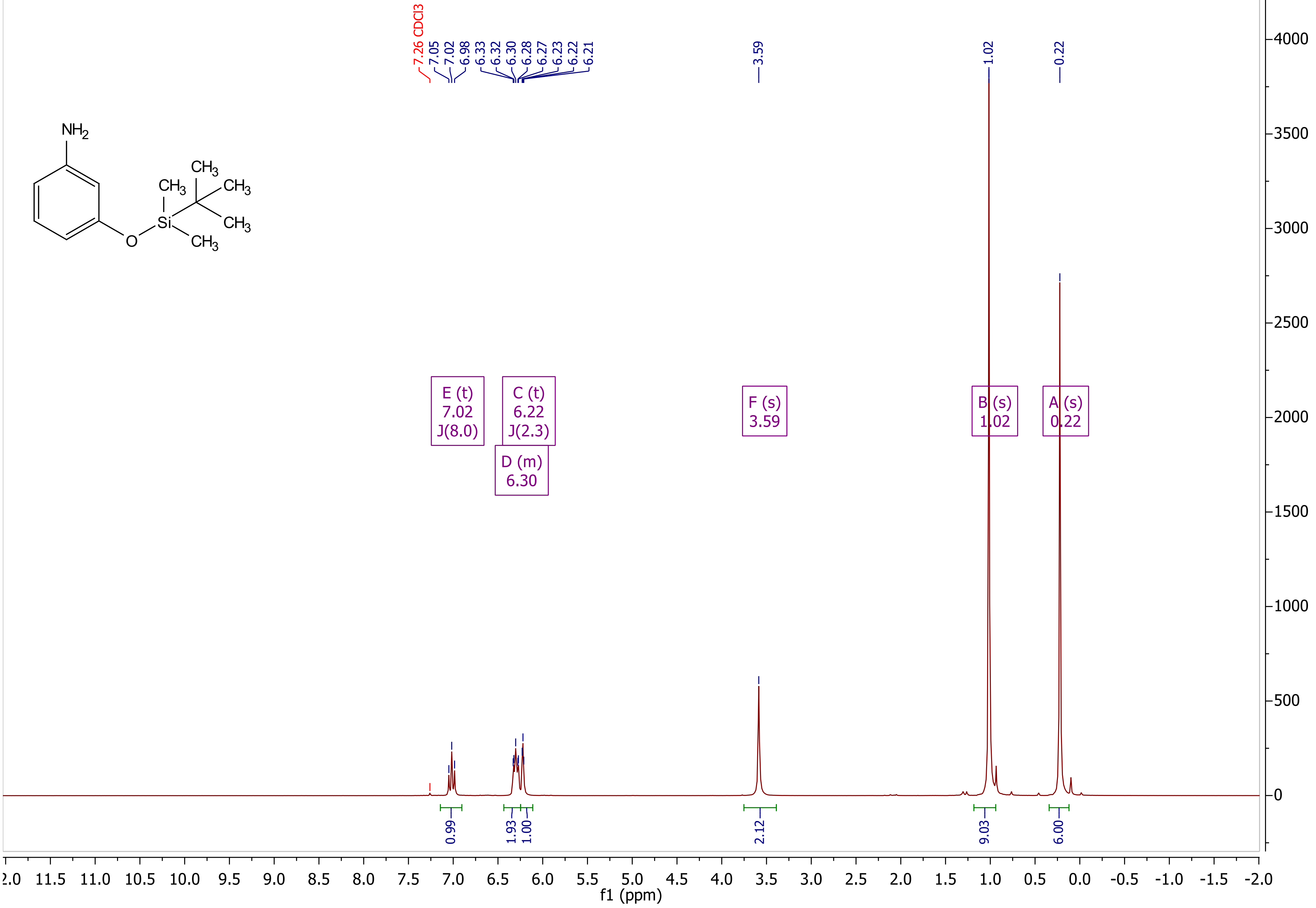

^{13}C NMR (63 MHz, Chloroform-*d*) δ 156.7, 147.7, 130.0, 110.5, 108.6, 107.2, 25.8, 18.2, -4.3.

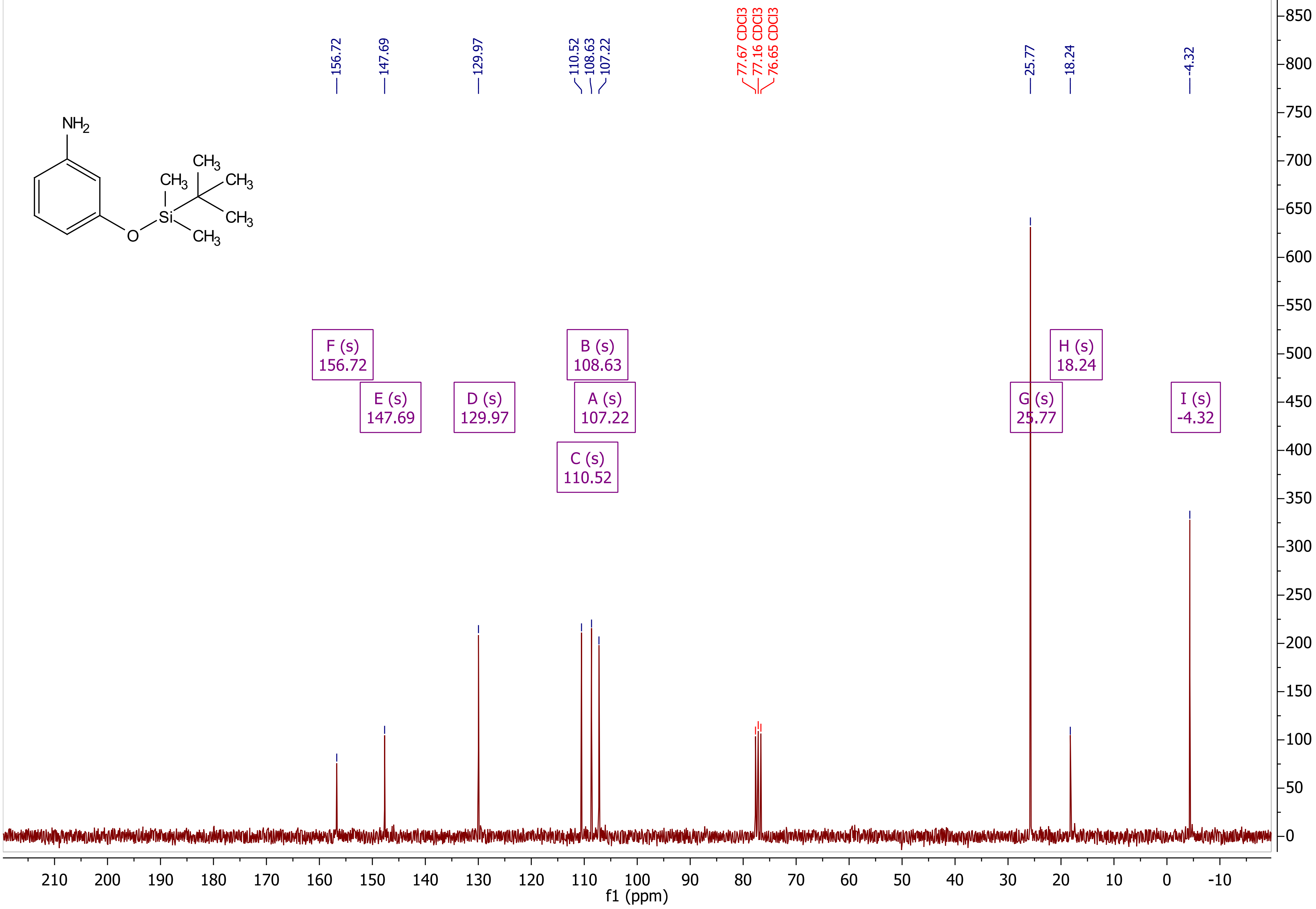

^{1}H NMR (250 MHz, DMSO-d_6) δ 8.11 – 8.00 (m, 2H), 7.65 – 7.51 (m, 3H).

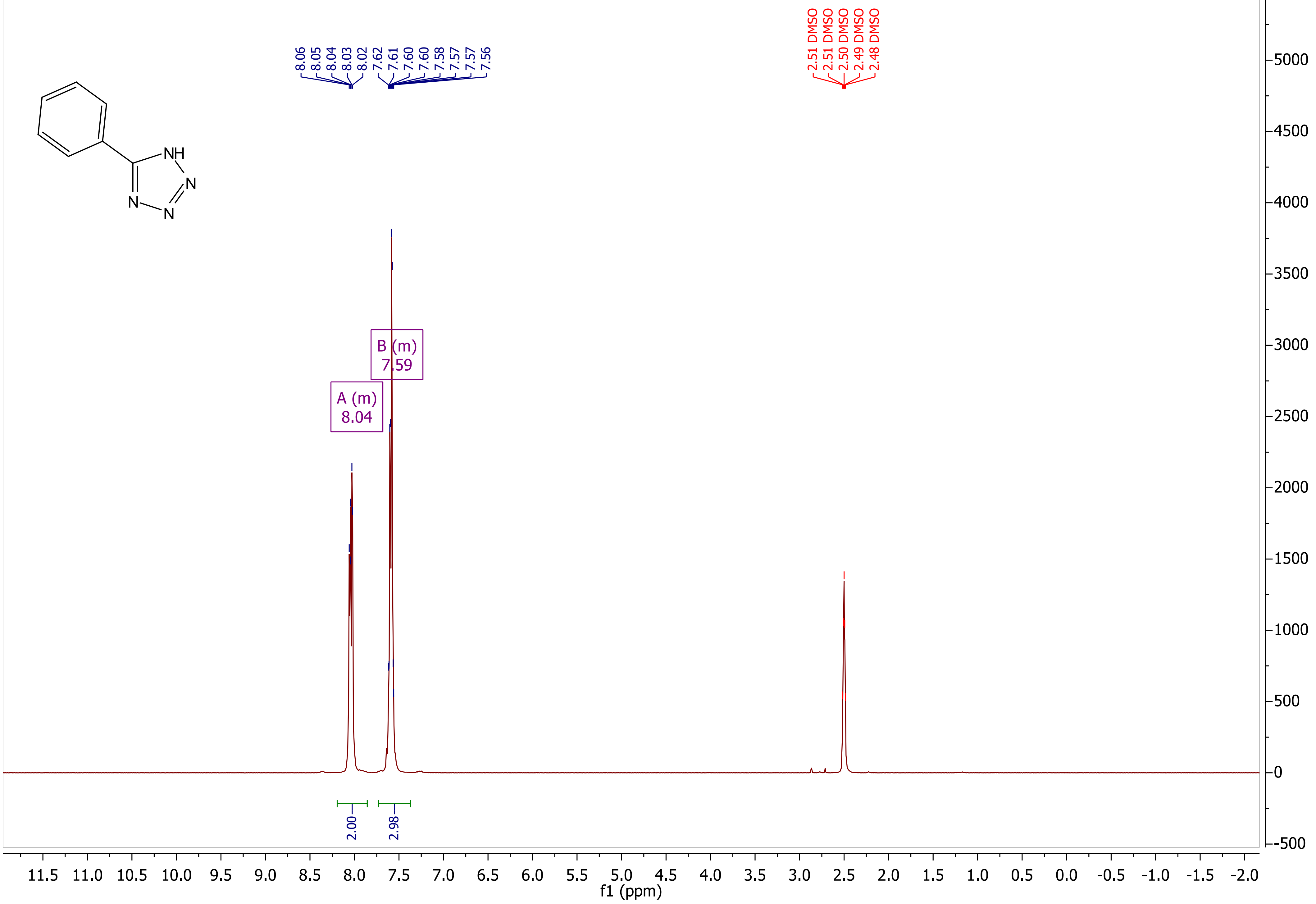

^{13}C NMR (63 MHz, DMSO-d_6) δ 155.3, 131.3, 129.4, 127.0, 124.2.

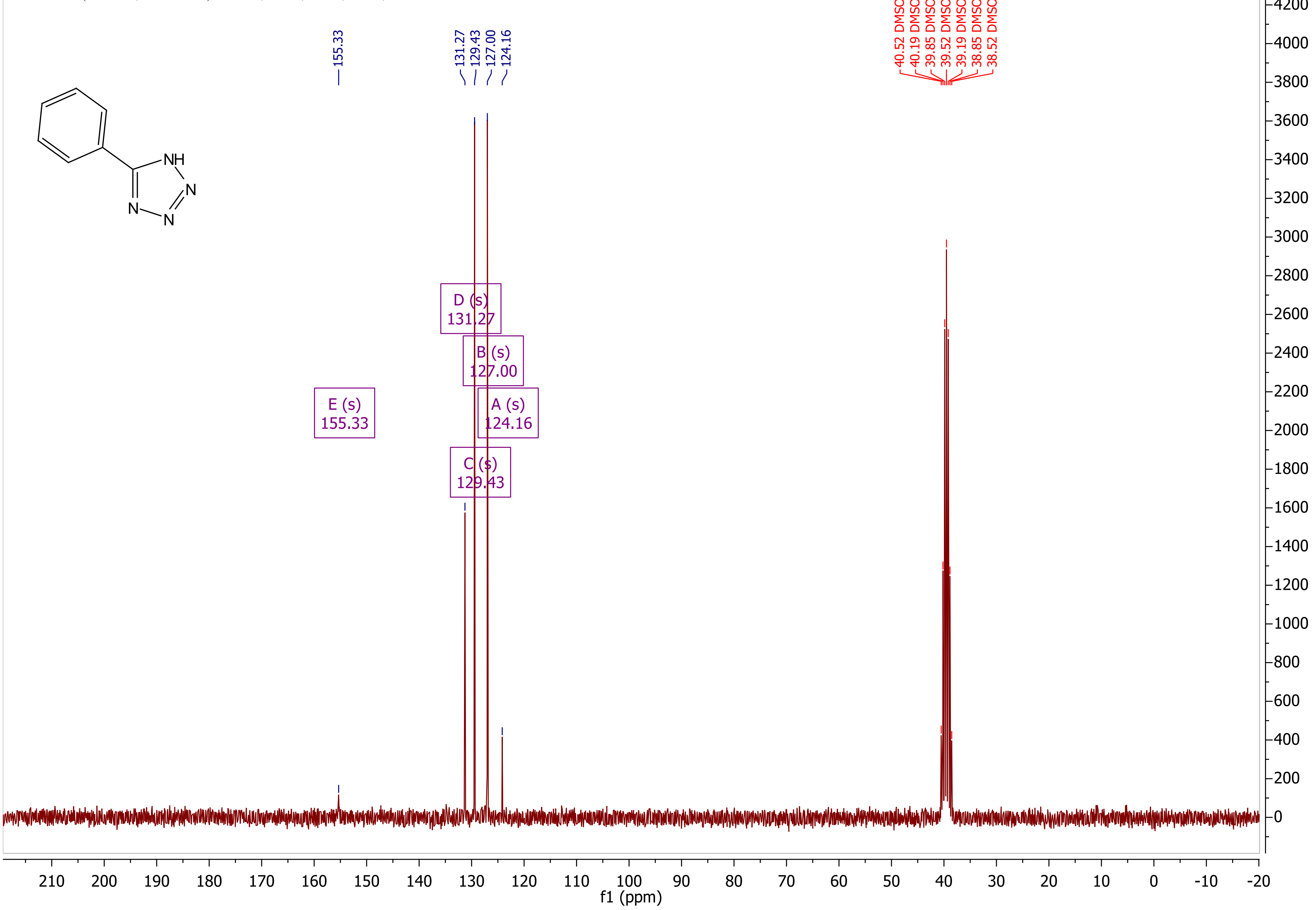

^{1}H NMR (400 MHz, DMSO-d_6) δ 4.55 (dd, J = 19.0, 13.5 Hz, 1H), 4.39 (dd, J = 19.5, 13.5 Hz, 1H).

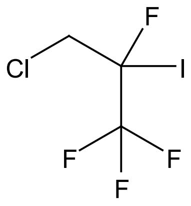

(2)

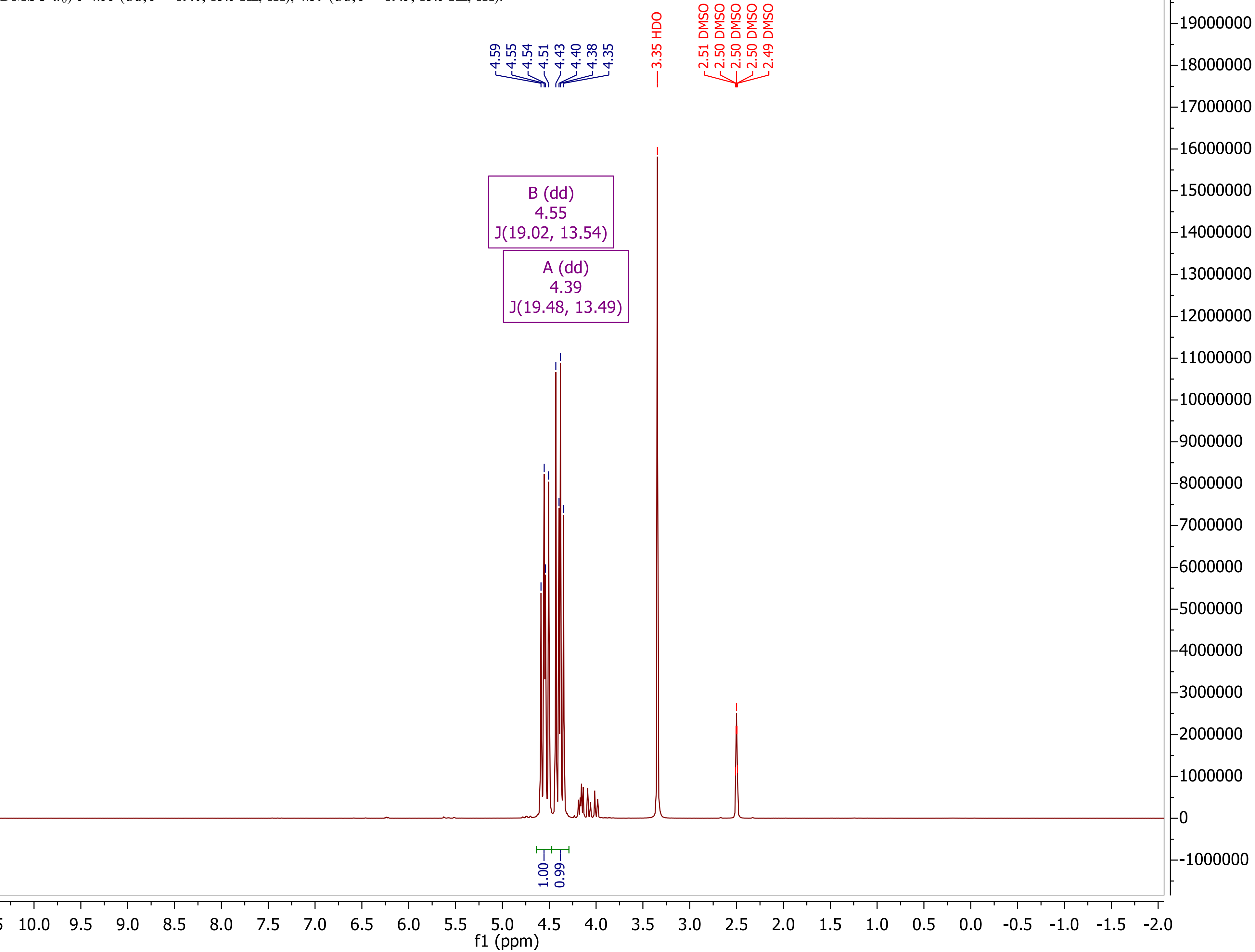

^{19}F NMR (376 MHz, DMSO-d_6) δ -74.7 (d, J = 11.8 Hz), -139.0 (q, J = 11.8 Hz).

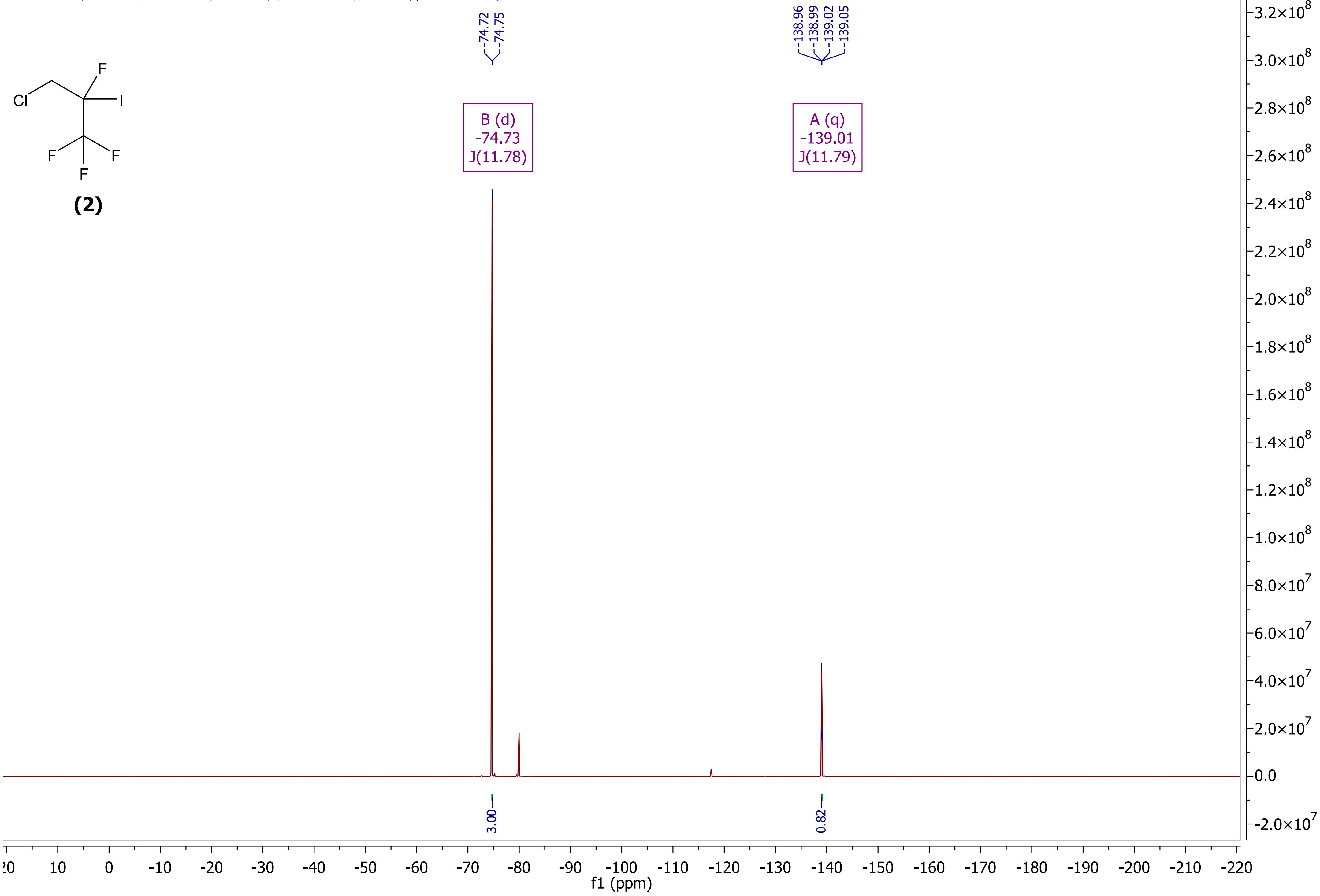

(2)

^{13}C NMR (101 MHz, DMSO-d_6) δ 121.5 (qd, J = 283.2, 29.2 Hz), 78.1 (dq, J = 255.6, 34.4 Hz), 48.4 (d, J = 22.1 Hz).

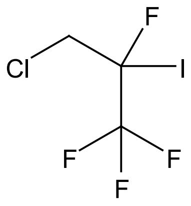

(2)

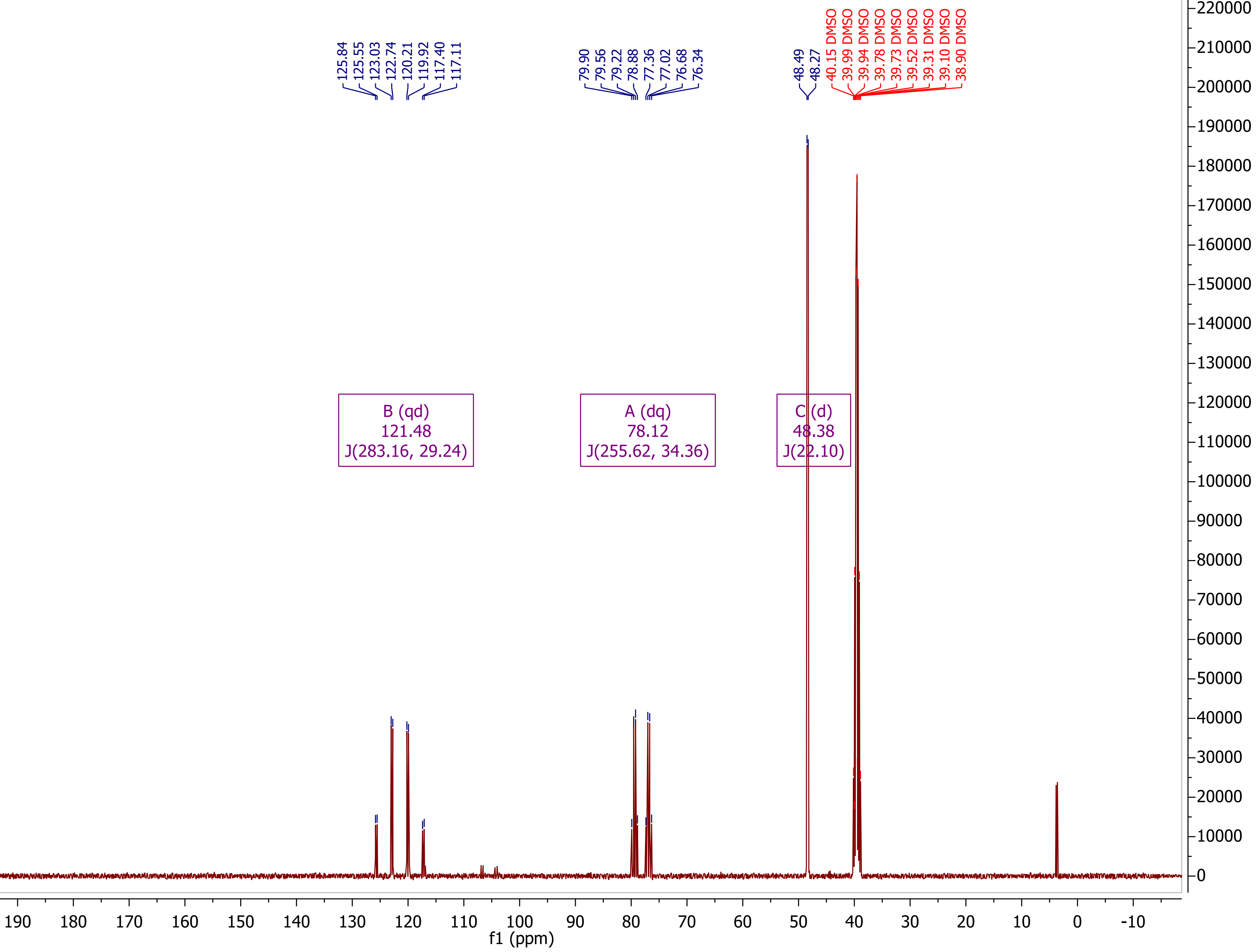

^{1}H NMR (400 MHz, DMSO-d_6) δ 4.16 (dd, *J* = 12.5, 7.7 Hz, 1H), 4.04 (dd, *J* = 30.6, 12.5 Hz, 1H).

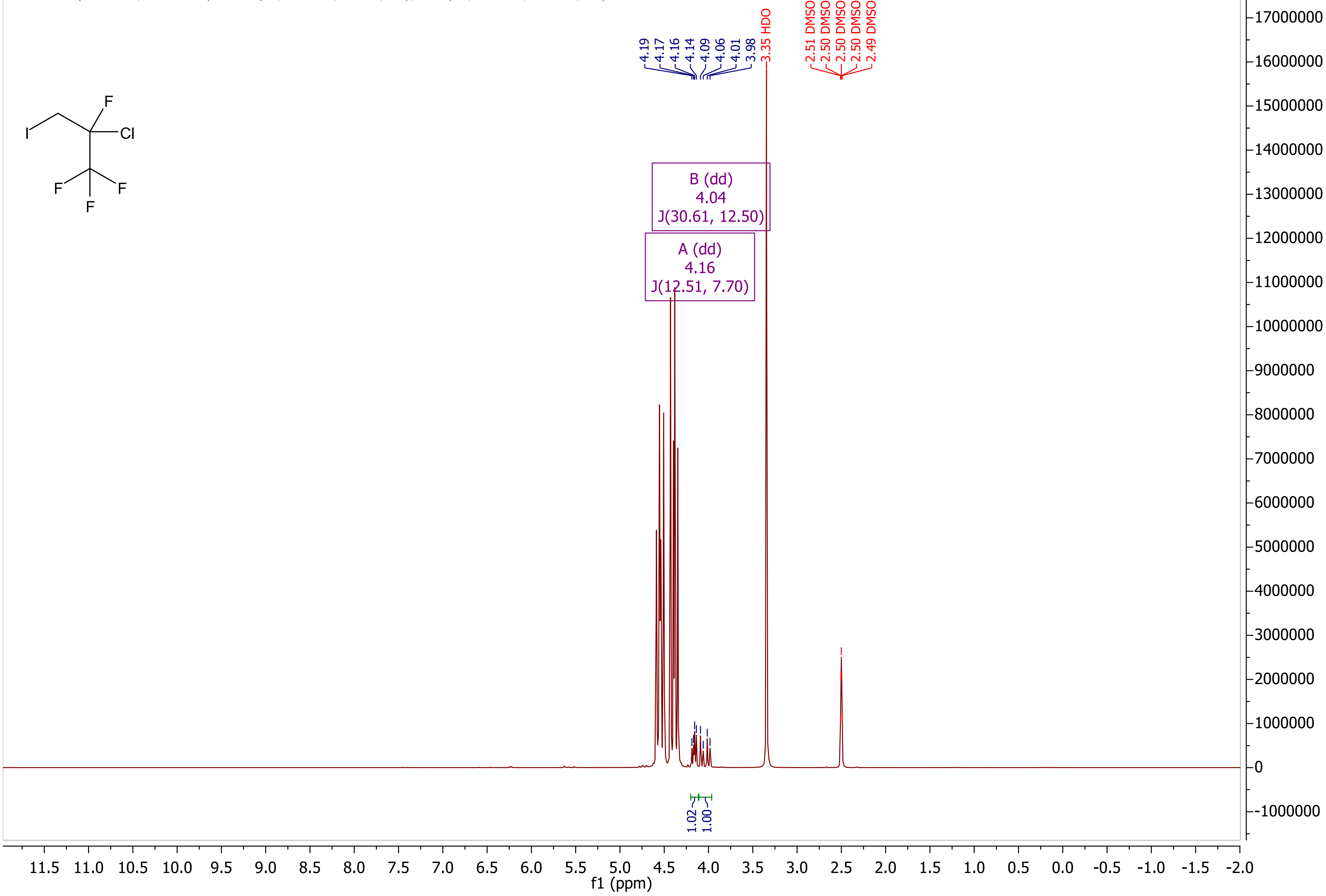

^{19}F NMR (376 MHz, DMSO-d_6) δ -80.0 (d, J = 6.5 Hz), -117.5 (q, J = 6.5 Hz).

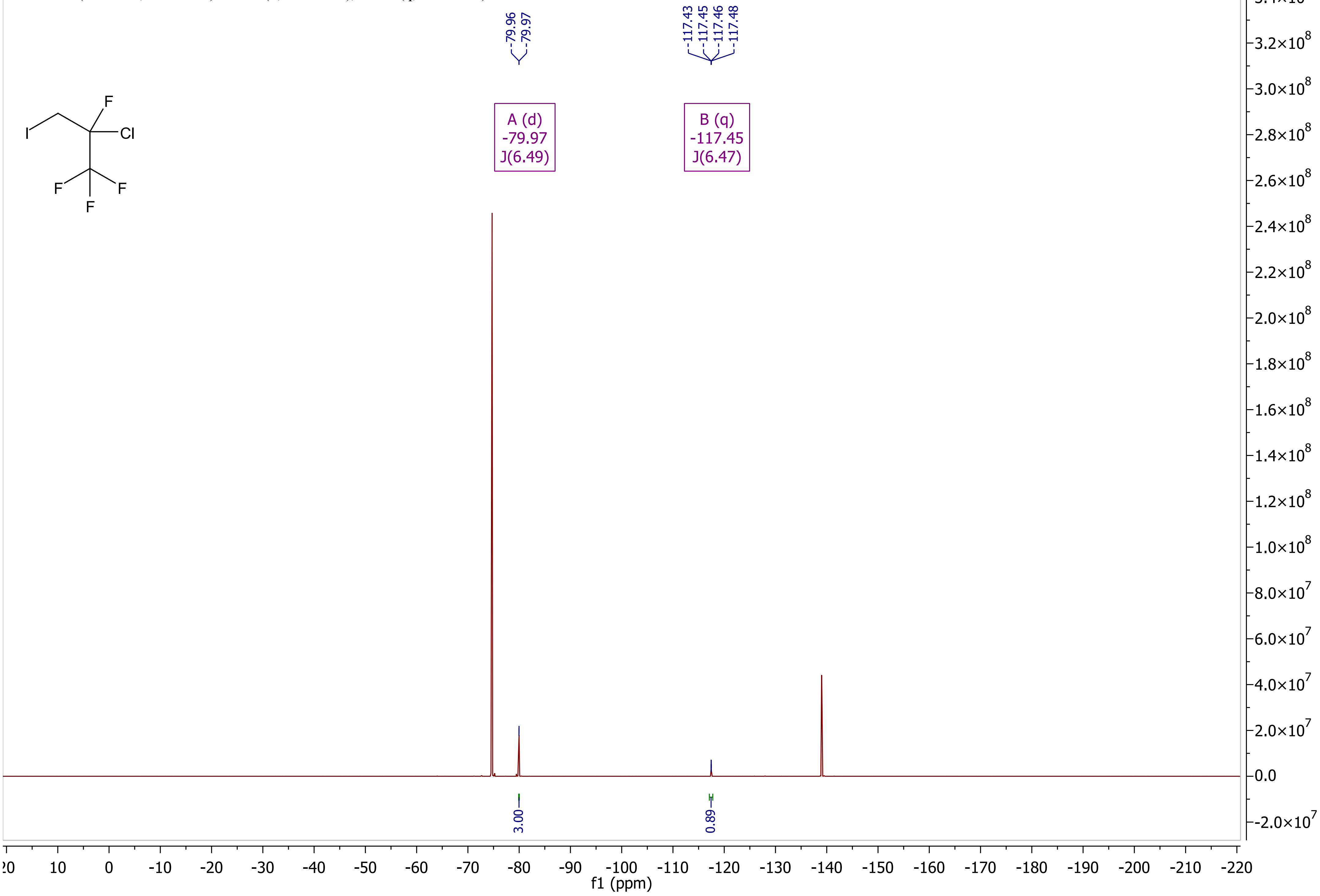

^{13}C NMR (101 MHz, DMSO-d_6) δ 118.5 (qd, J = 285.4, 32.7 Hz), 105.5 (dq, J = 251.2, 36.1 Hz), 3.7 (d, J = 23.4 Hz).

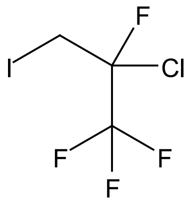

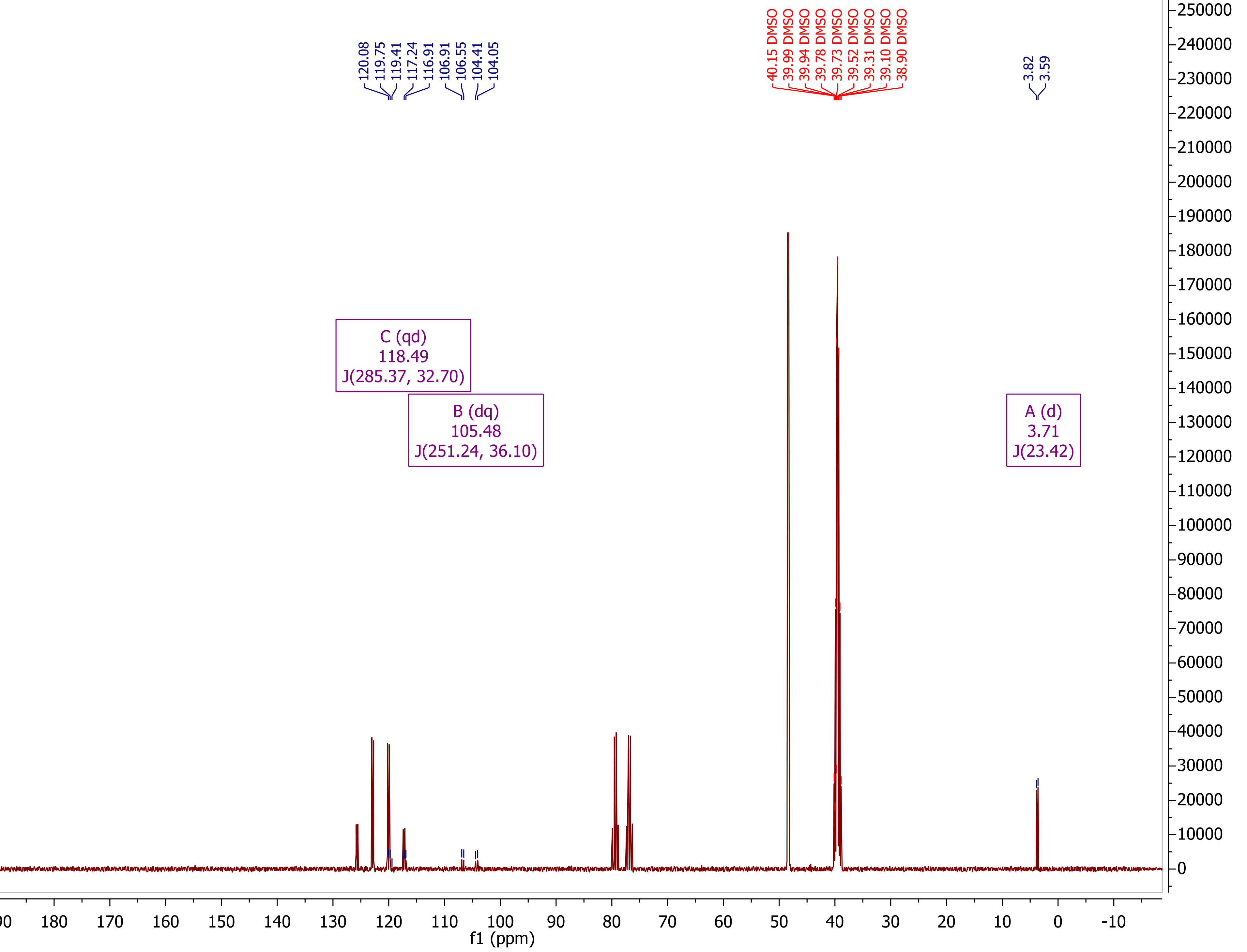

^{1}H NMR (400 MHz, Acetonitrile-d_3) δ 8.22 (dd, J = 8.9, 4.8 Hz, 2H), 7.33 (t, J = 8.8 Hz, 2H), 5.09 (dd, J = 13.1, 8.7 Hz, 1H), 4.93 (dd, J = 28.1, 13.2 Hz, 1H).

(10)

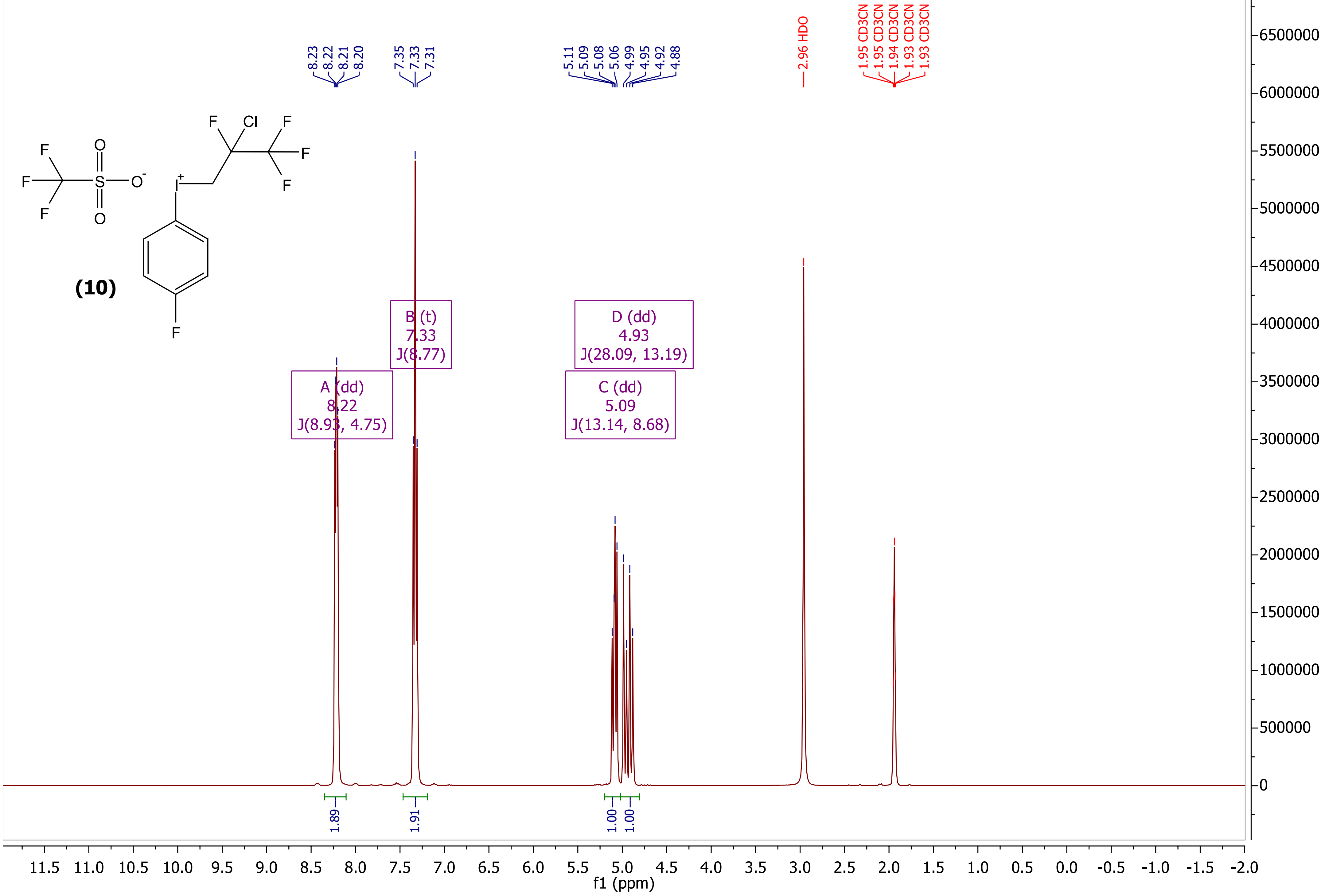

^{19}F NMR (376 MHz, Acetonitrile-d_3) δ -79.3, -80.9 (d, J = 6.2 Hz), -104.9, -118.3 (q, J = 6.2 Hz).

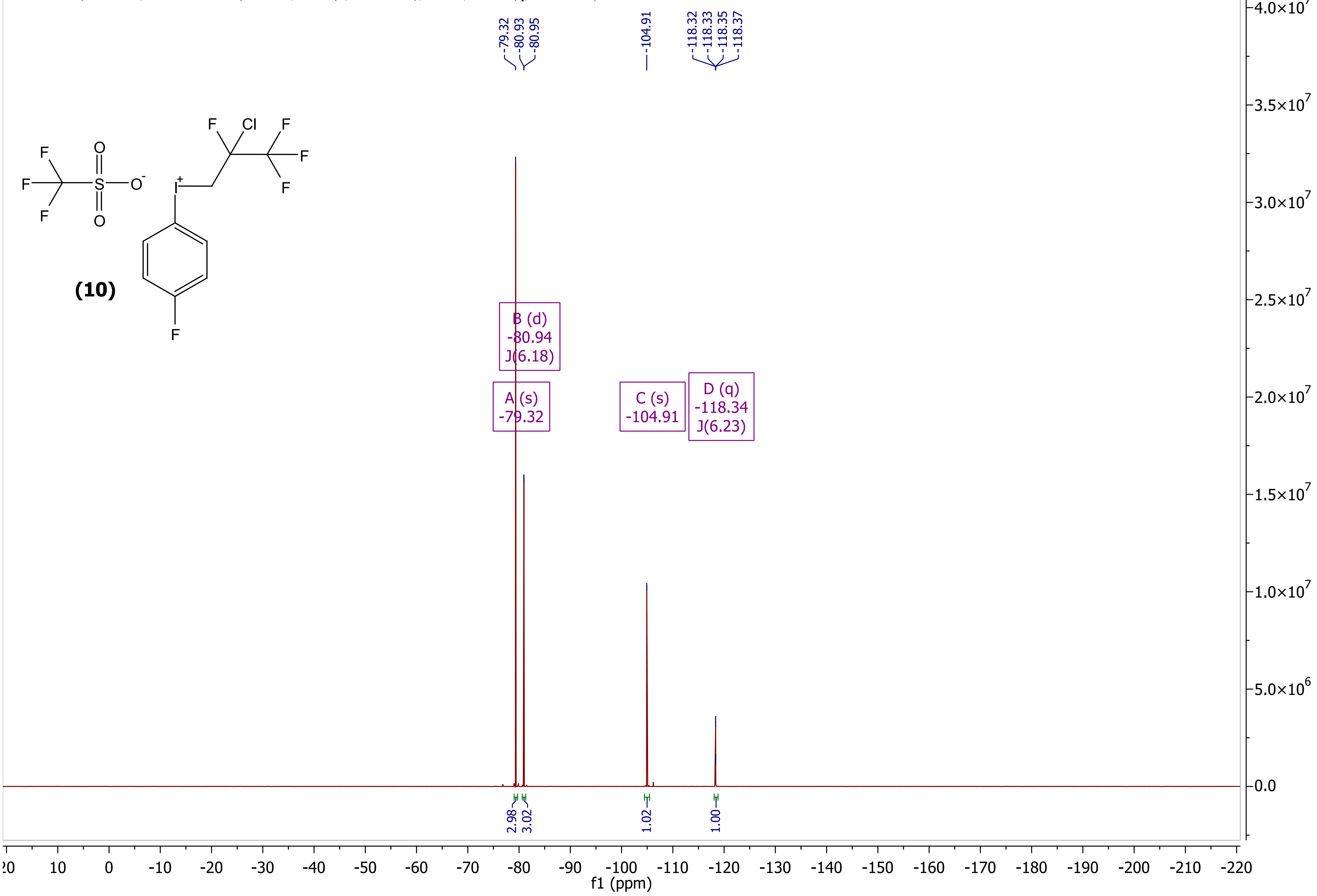

^{13}C NMR (101 MHz, Acetonitrile-d_3) δ 166.6 (d, J = 254.8 Hz), 141.4 (d, J = 9.6 Hz), 121.7 (q, J = 320.0 Hz), 120.8 (d, J = 23.6 Hz), 119.9 (qd, J = 285.5, 31.3 Hz), 104.7 (dq, J = 254.7, 38.4 Hz), 103.8 (d, J = 3.3 Hz), 41.7 (d, J = 21.9 Hz).

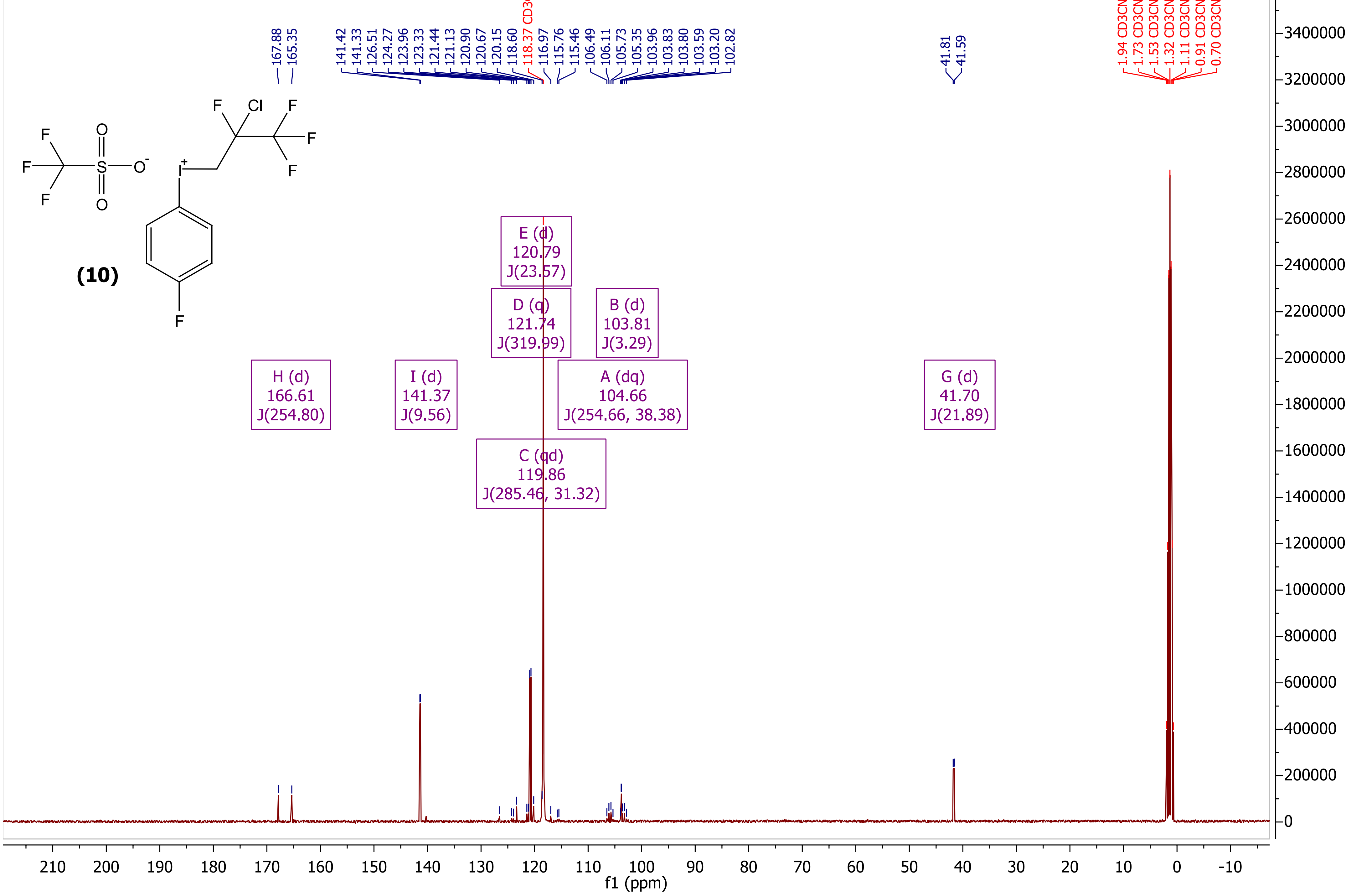

(10)

^{1}H NMR (250 MHz, Acetonitrile-d_3) δ 8.26 – 7.93 (m, 4H), 7.37 – 7.11 (m, 4H).

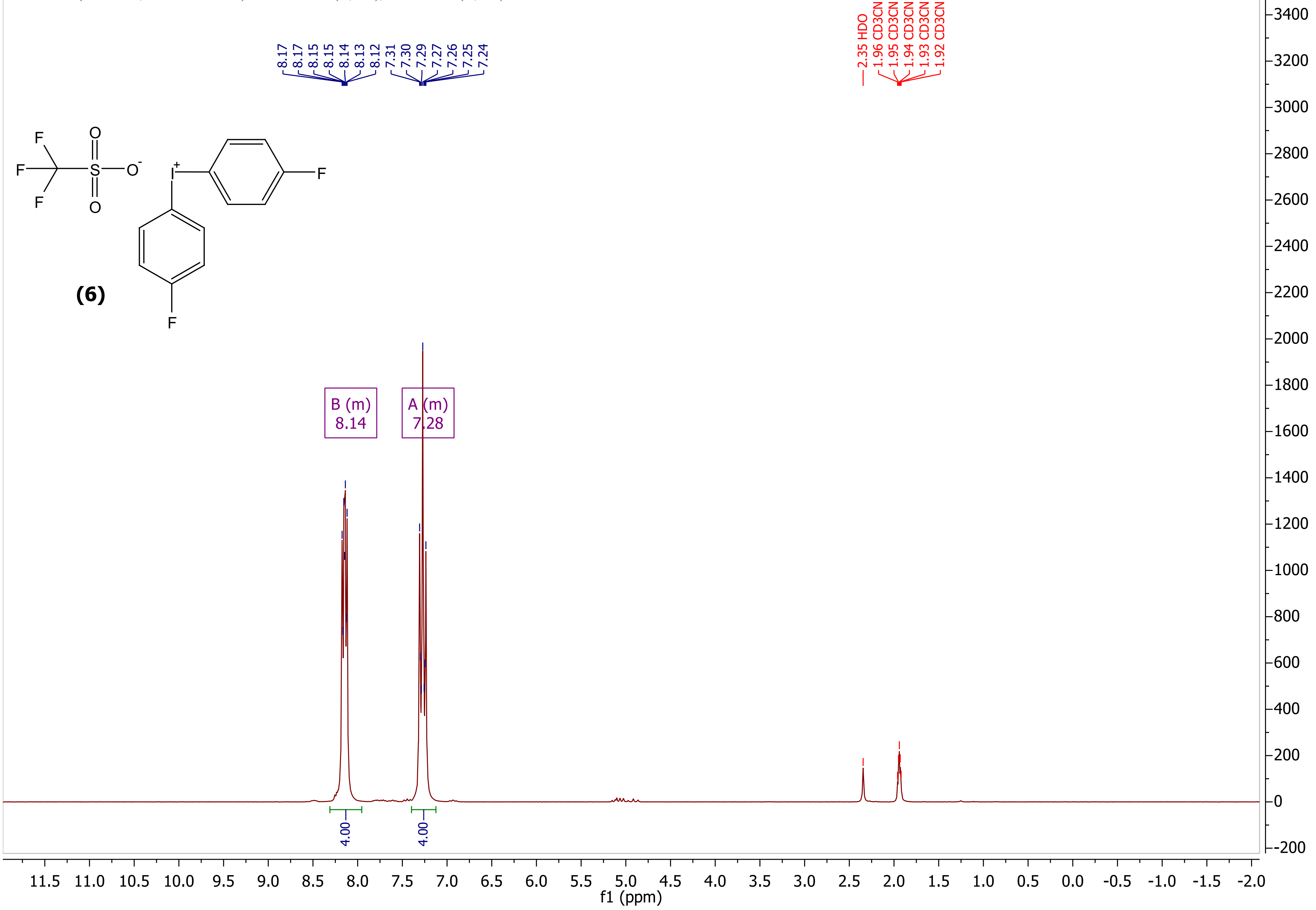

^{19}F NMR (235 MHz, Acetonitrile-d_3) δ -79.2, -106.3.

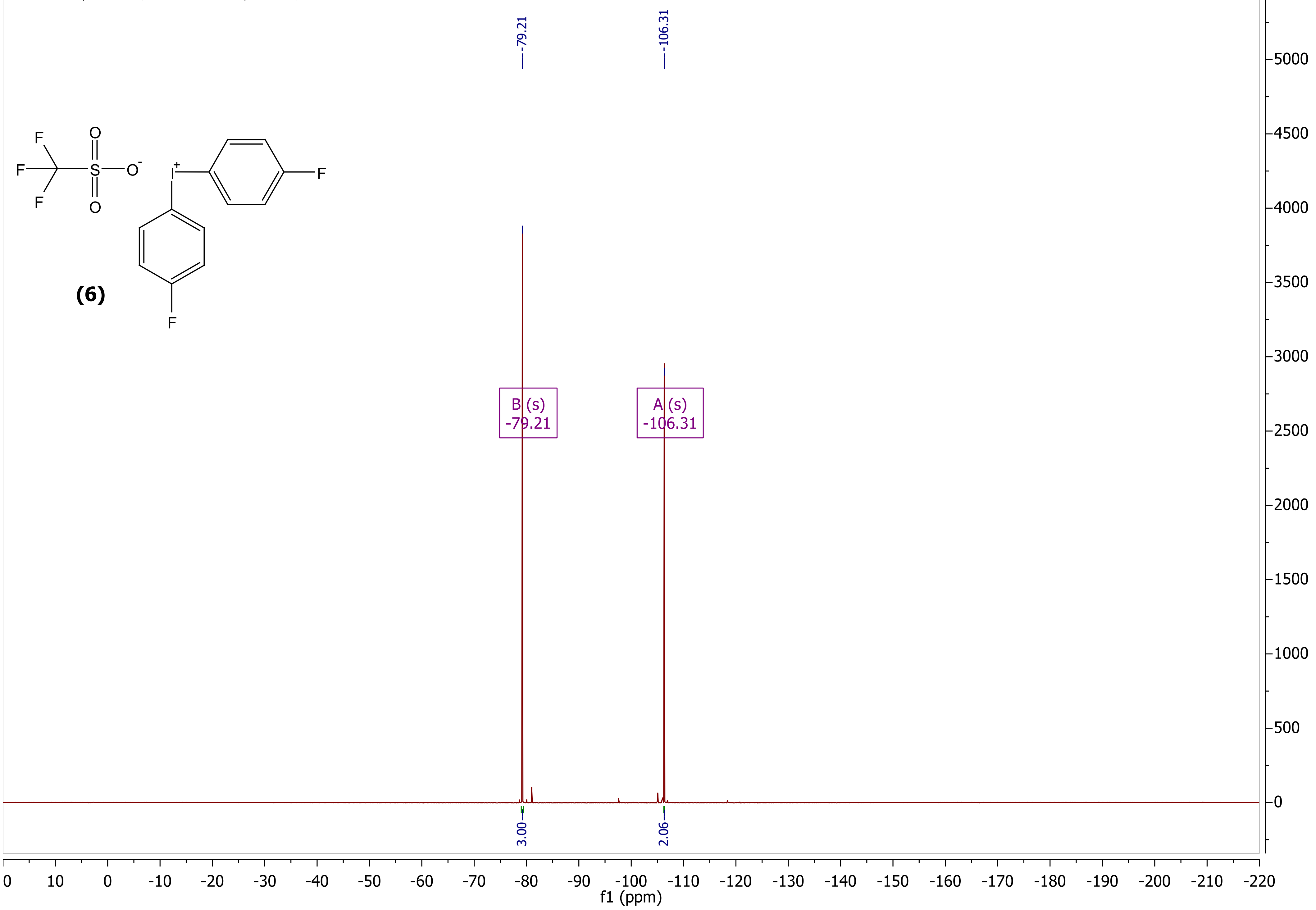

^{13}C NMR (63 MHz, Acetonitrile-d_3) δ 166.1 (d, J = 253.7 Hz), 139.3 (d, J = 9.4 Hz), 121.8 (q, J = 320.3 Hz), 120.7 (d, J = 23.4 Hz), 108.7 (d, J = 3.2 Hz).

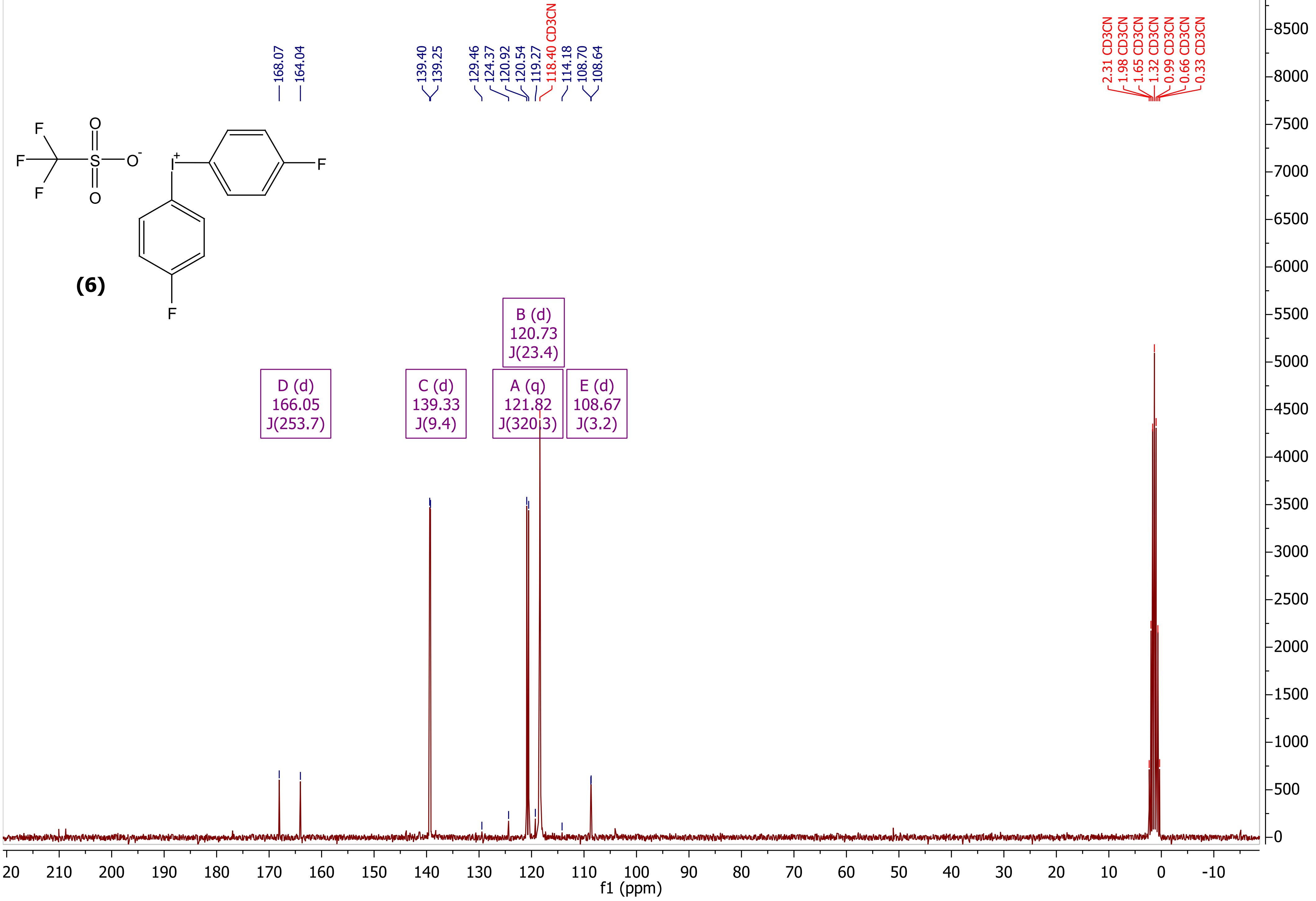

^{1}H NMR (400 MHz, Acetonitrile-d_3) δ 8.16 (d, J = 8.8 Hz, 2H), 7.58 (d, J = 8.8 Hz, 2H), 5.10 (dd, J = 13.2, 8.5 Hz, 1H), 4.94 (dd, J = 28.2, 13.2 Hz, 1H).

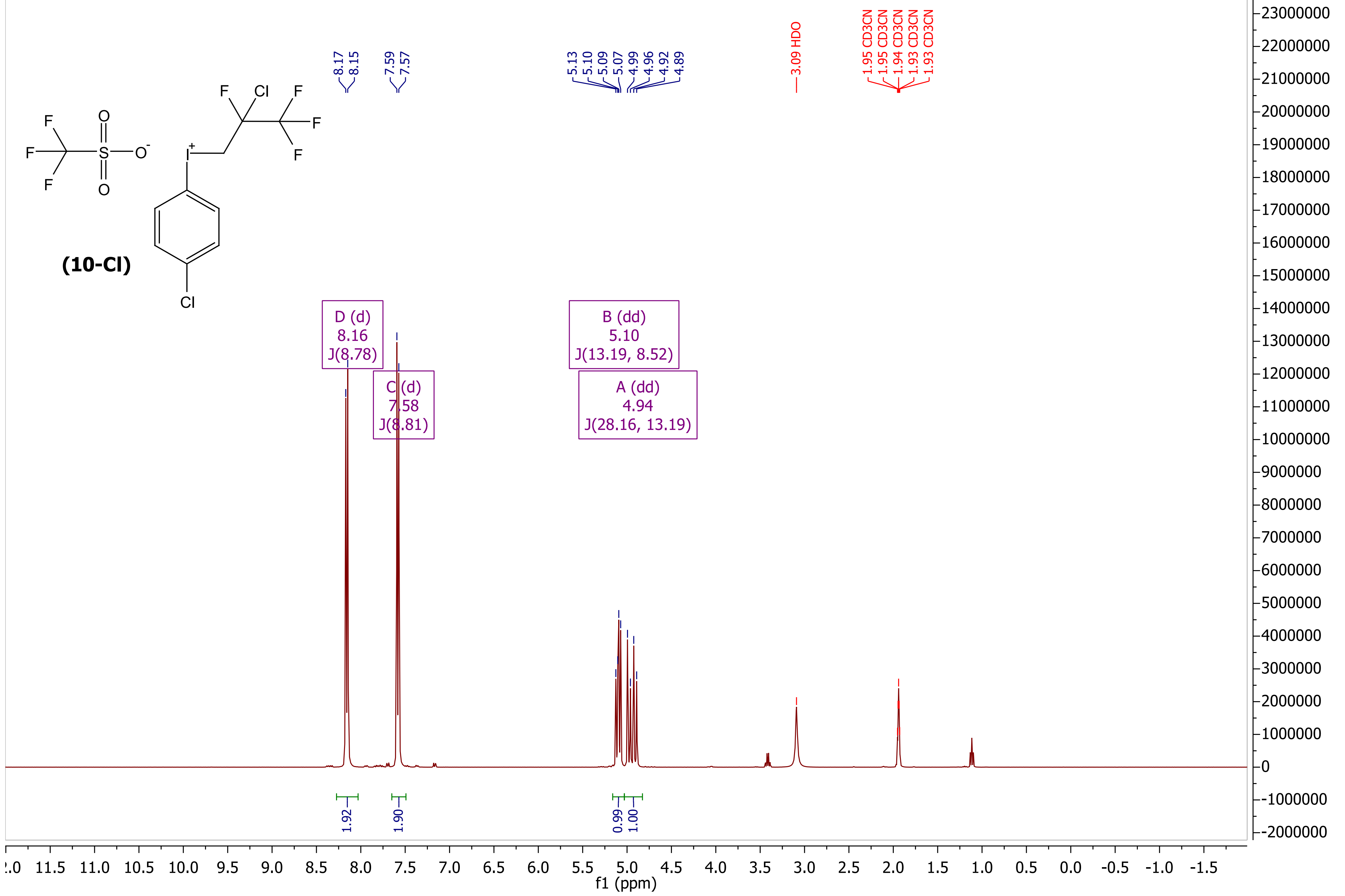

^{19}F NMR (376 MHz, Acetonitrile-d_3) δ -79.3, -80.9 (d, J = 6.3 Hz), -118.2 (q, J = 6.1 Hz).

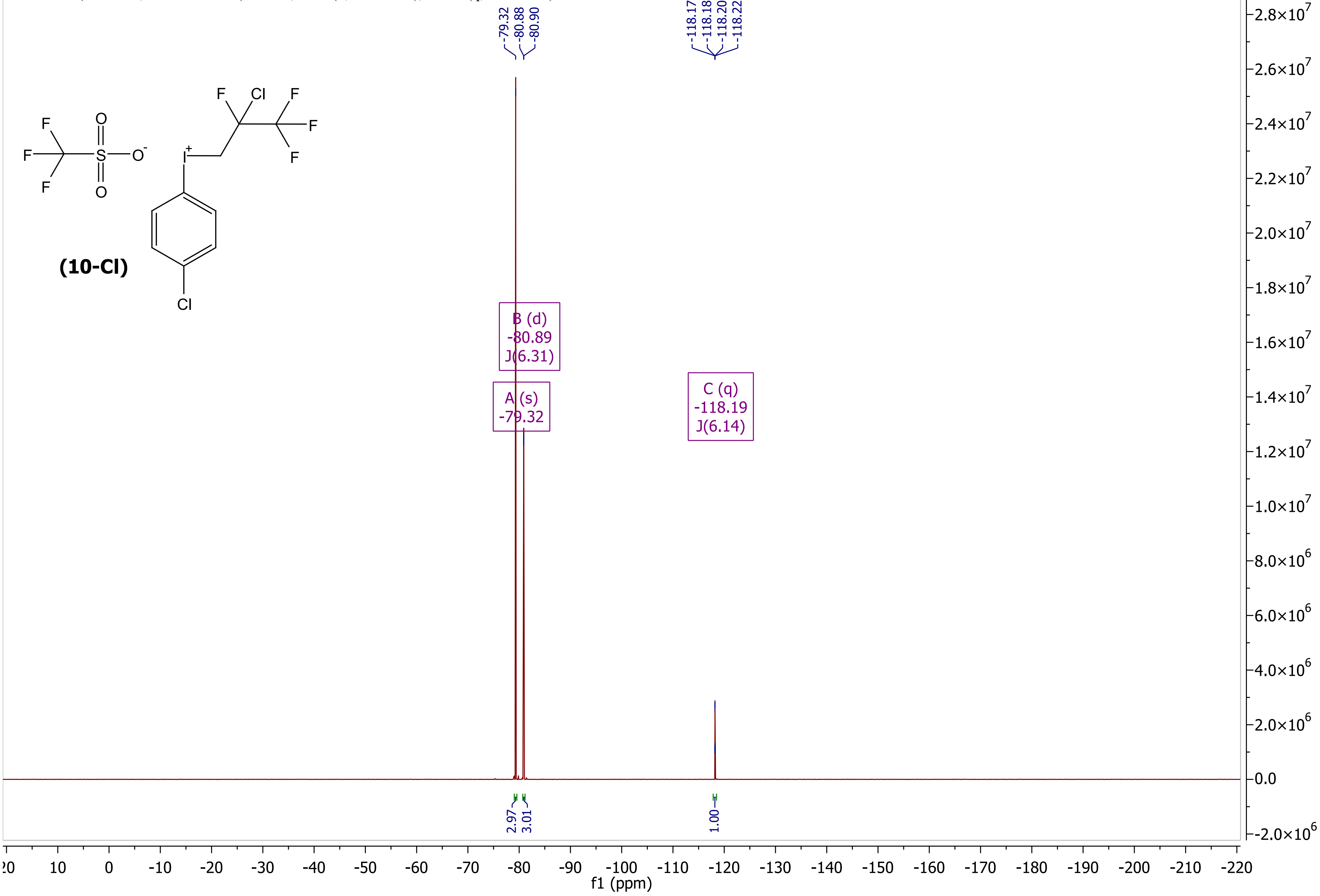

^{13}C NMR (101 MHz, Acetonitrile-d_3) δ 140.9, 139.8, 133.2, 121.5 (q, J = 319.9 Hz), 119.9 (qd, J = 285.4, 31.1 Hz), 107.6, 104.5 (dq, J = 254.5, 38.3 Hz), 41.5 (d, J = 21.8 Hz).

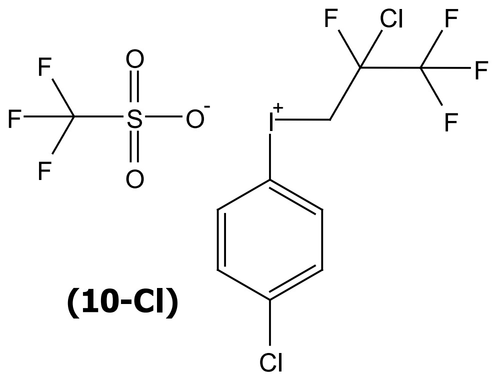

(10-Cl)

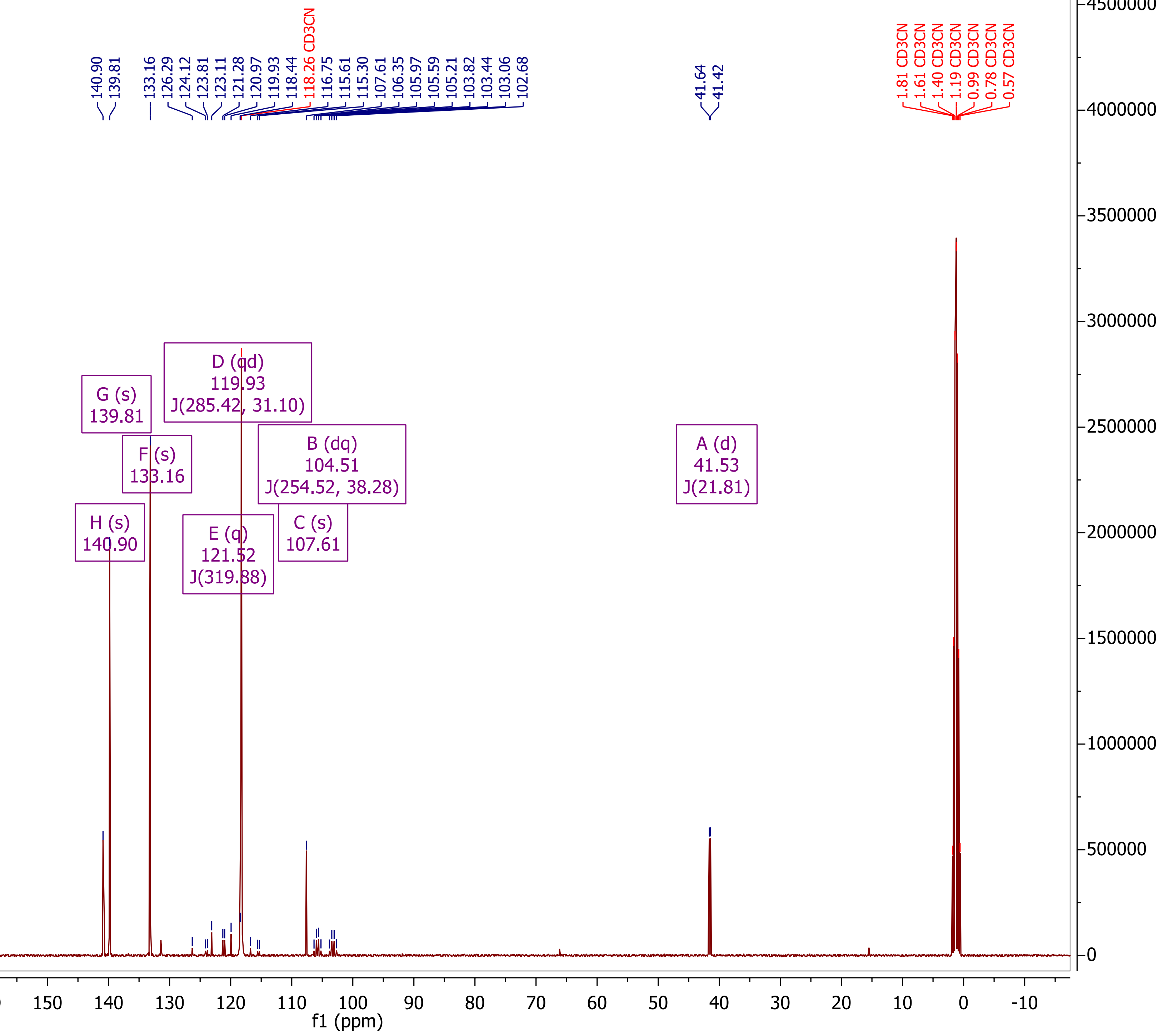

^{1}H NMR (400 MHz, Acetonitrile-d_3) δ 8.19 (d, J = 8.1 Hz, 2H), 7.79 (t, J = 7.5 Hz, 1H), 7.58 (t, J = 7.9 Hz, 2H), 5.10 (dd, J = 13.2, 8.5 Hz, 1H), 4.95 (dd, J = 28.3, 13.2 Hz, 1H).

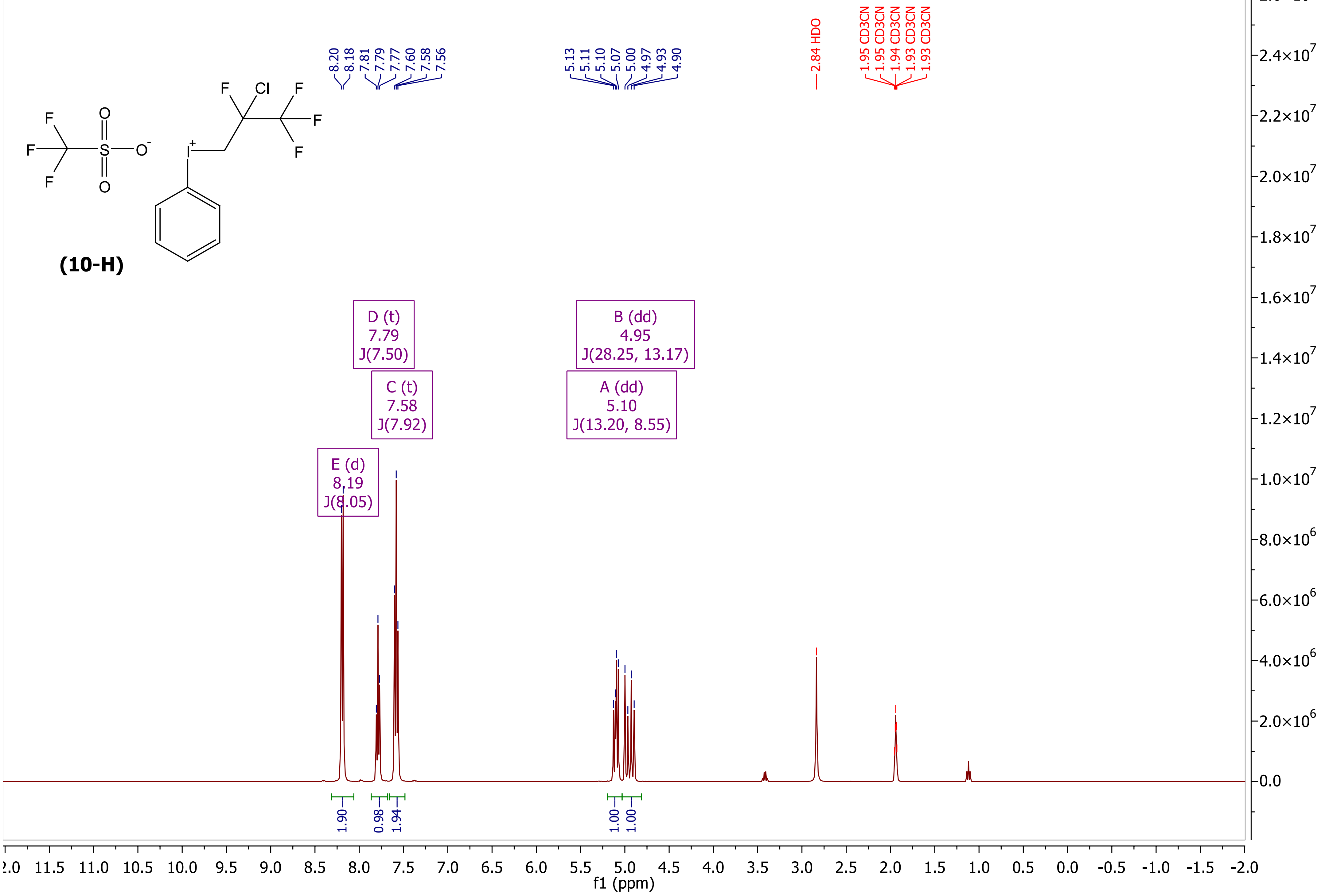

^{19}F NMR (376 MHz, Acetonitrile-d_3) δ -79.3, -80.9 (d, J = 6.0 Hz), -118.3 (q, J = 6.1 Hz).

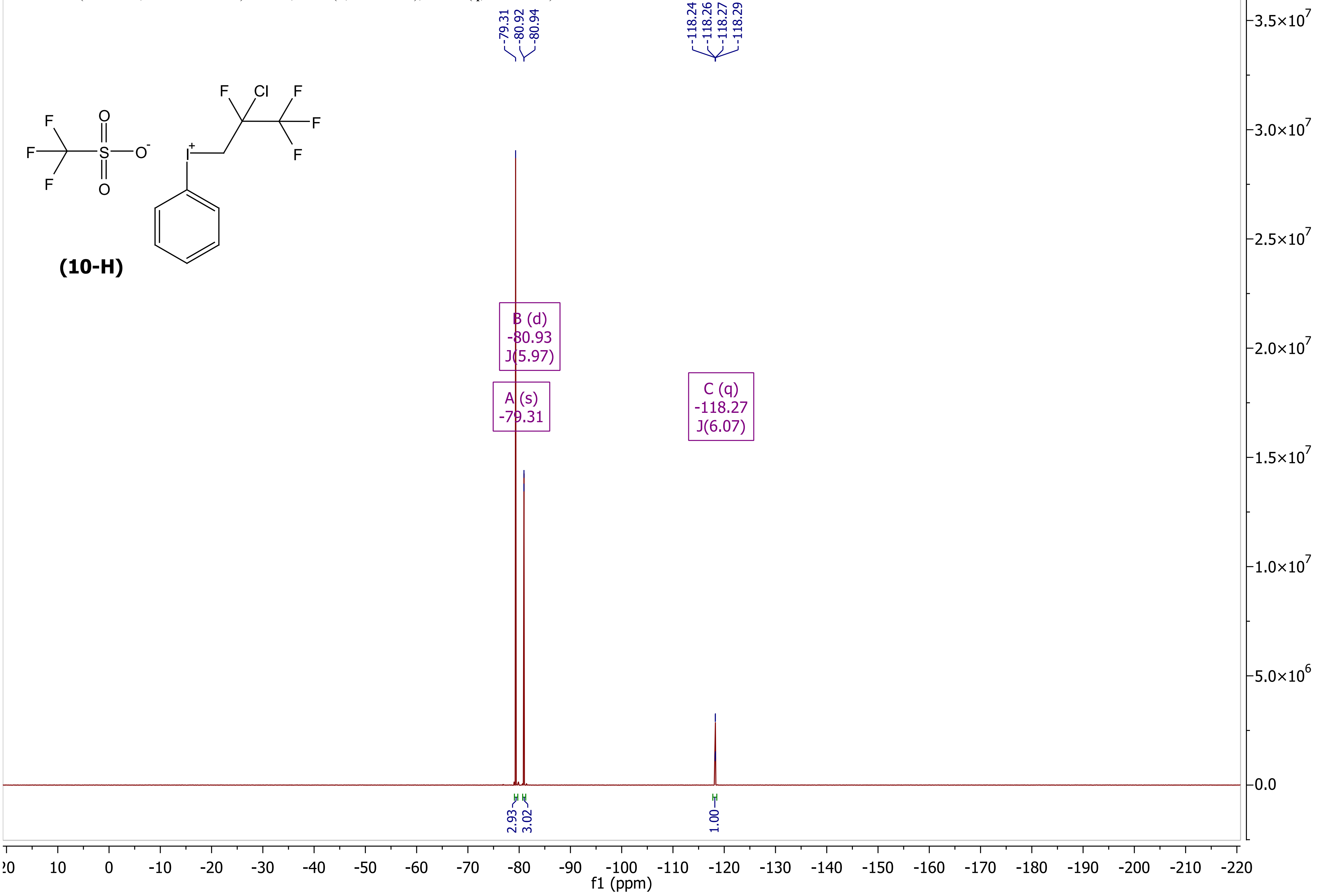

^{13}C NMR (101 MHz, Acetonitrile-d_3) δ 138.1, 134.5, 133.1, 121.6 (q, J = 320.0 Hz), 119.9 (qd, J = 285.5, 31.8 Hz), 110.4, 104.5 (dq, J = 254.5, 38.4 Hz), 41.0 (d, J = 21.9 Hz).

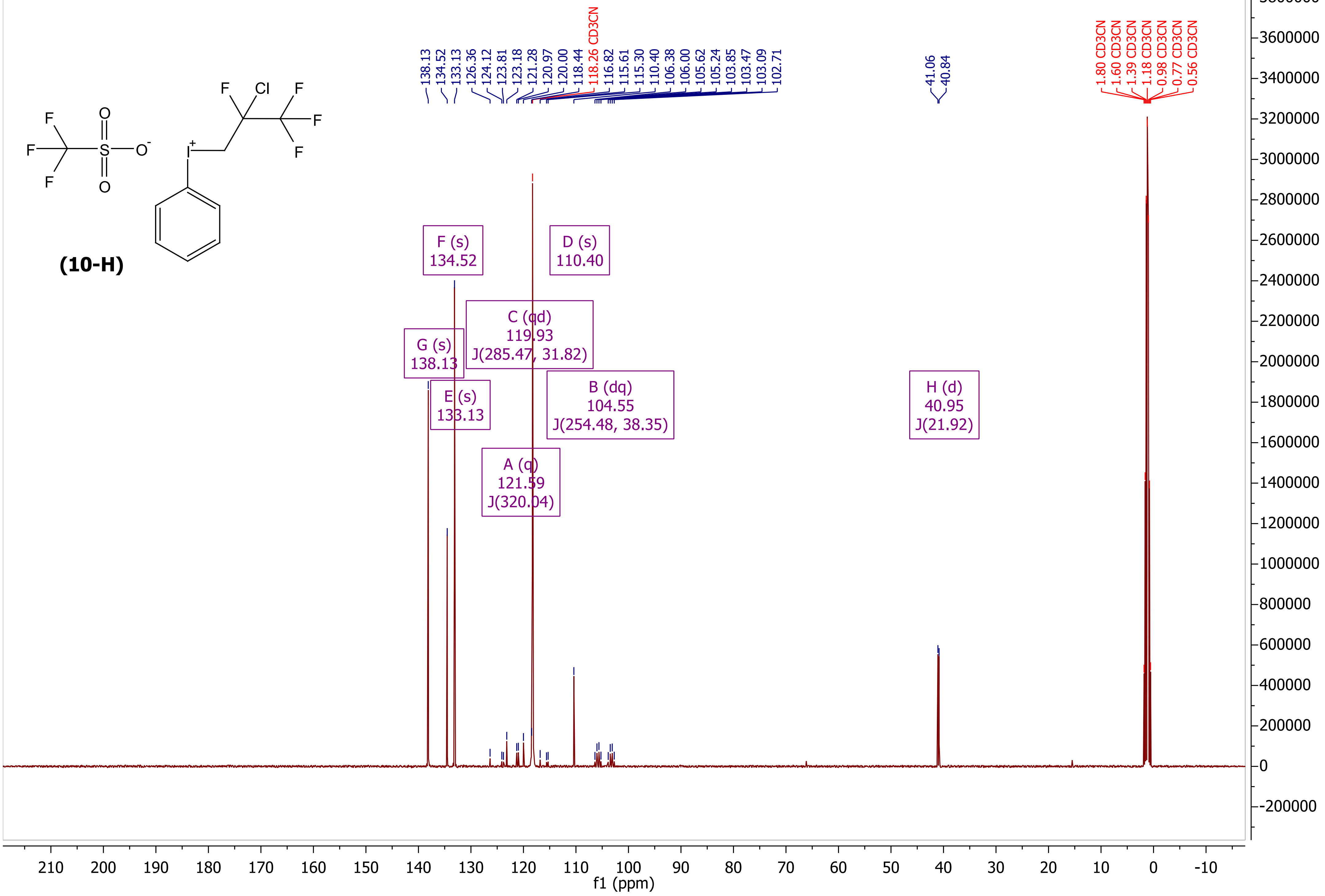

^{1}H NMR (400 MHz, Acetonitrile-d_3) δ 7.24 (s, 2H), 4.90 (dd, J = 12.9, 7.7 Hz, 1H), 4.80 (dd, J = 29.8, 12.9 Hz, 1H), 2.65 (s, 6H), 2.37 (s, 3H).

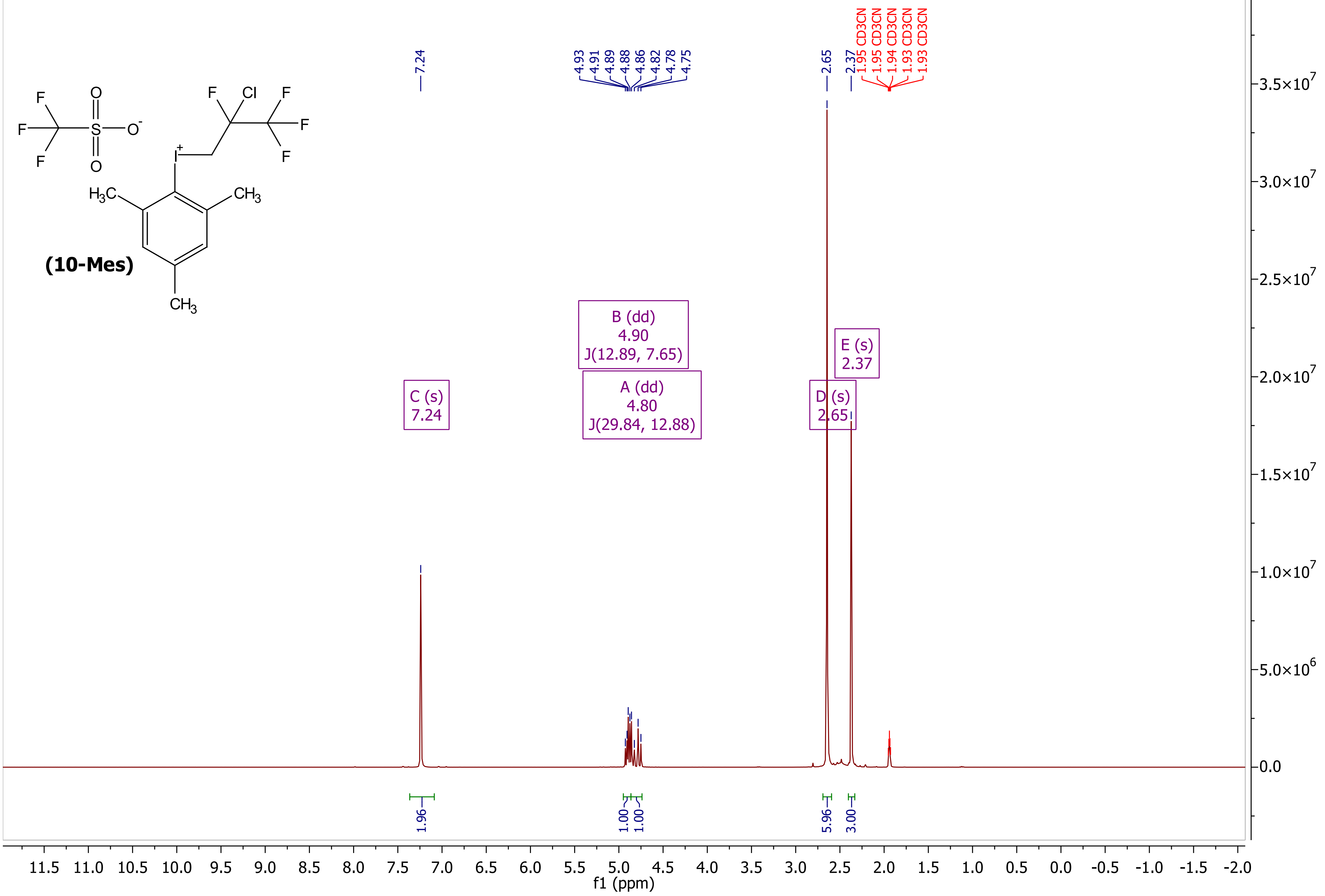

^{19}F NMR (376 MHz, Acetonitrile-d_3) δ -79.3, -80.9 (d, J = 6.1 Hz), -118.1 (q, J = 6.2 Hz).

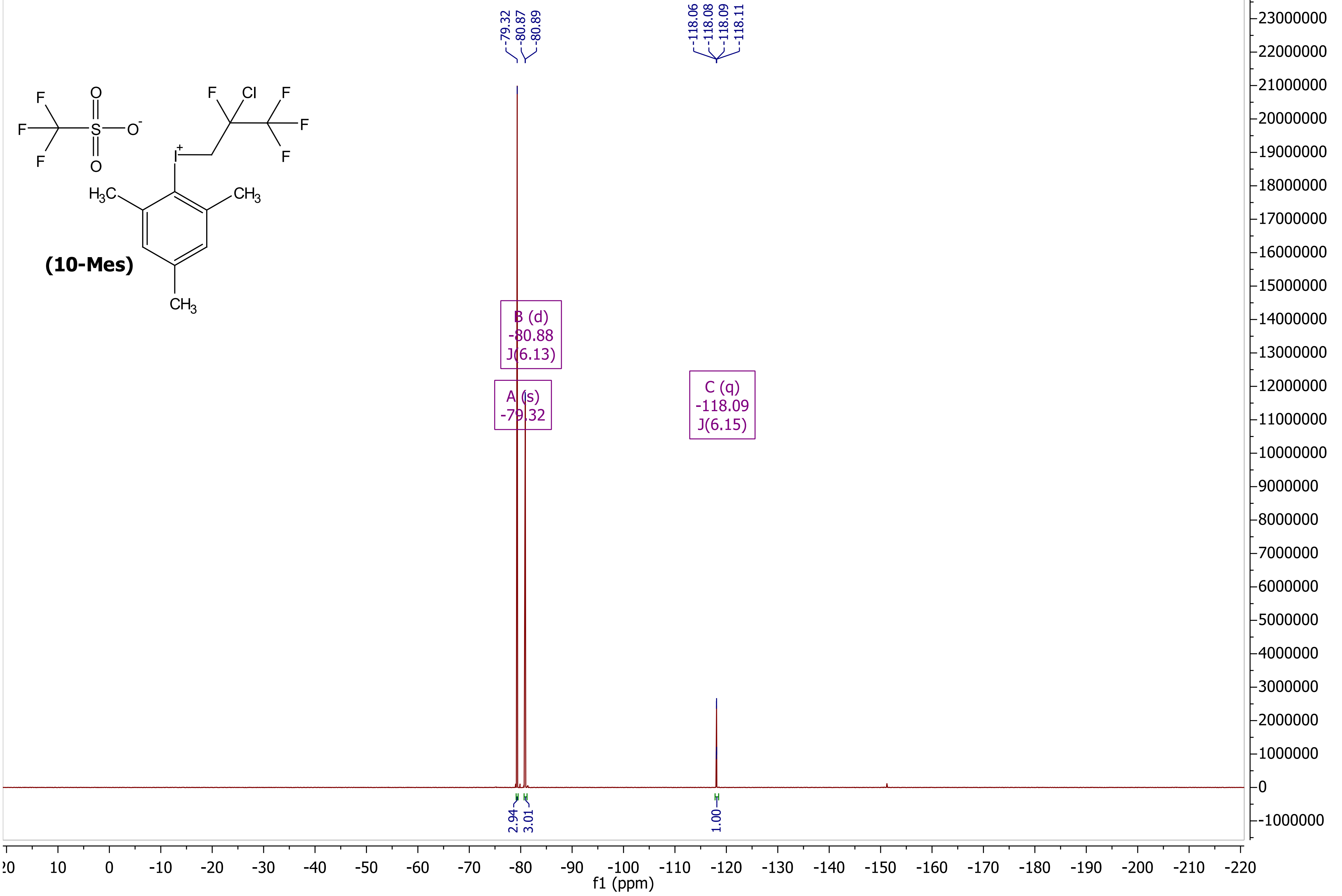

^{13}C NMR (101 MHz, Acetonitrile-d_3) δ 146.2, 144.3, 131.1, 121.7 (q, J = 320.0 Hz), 119.8 (qd, J = 285.4, 31.3 Hz), 118.2, 104.5 (dq, J = 254.0, 38.5 Hz), 39.1 (d, J = 22.1 Hz), 27.4 (d, J = 1.2 Hz), 21.0.

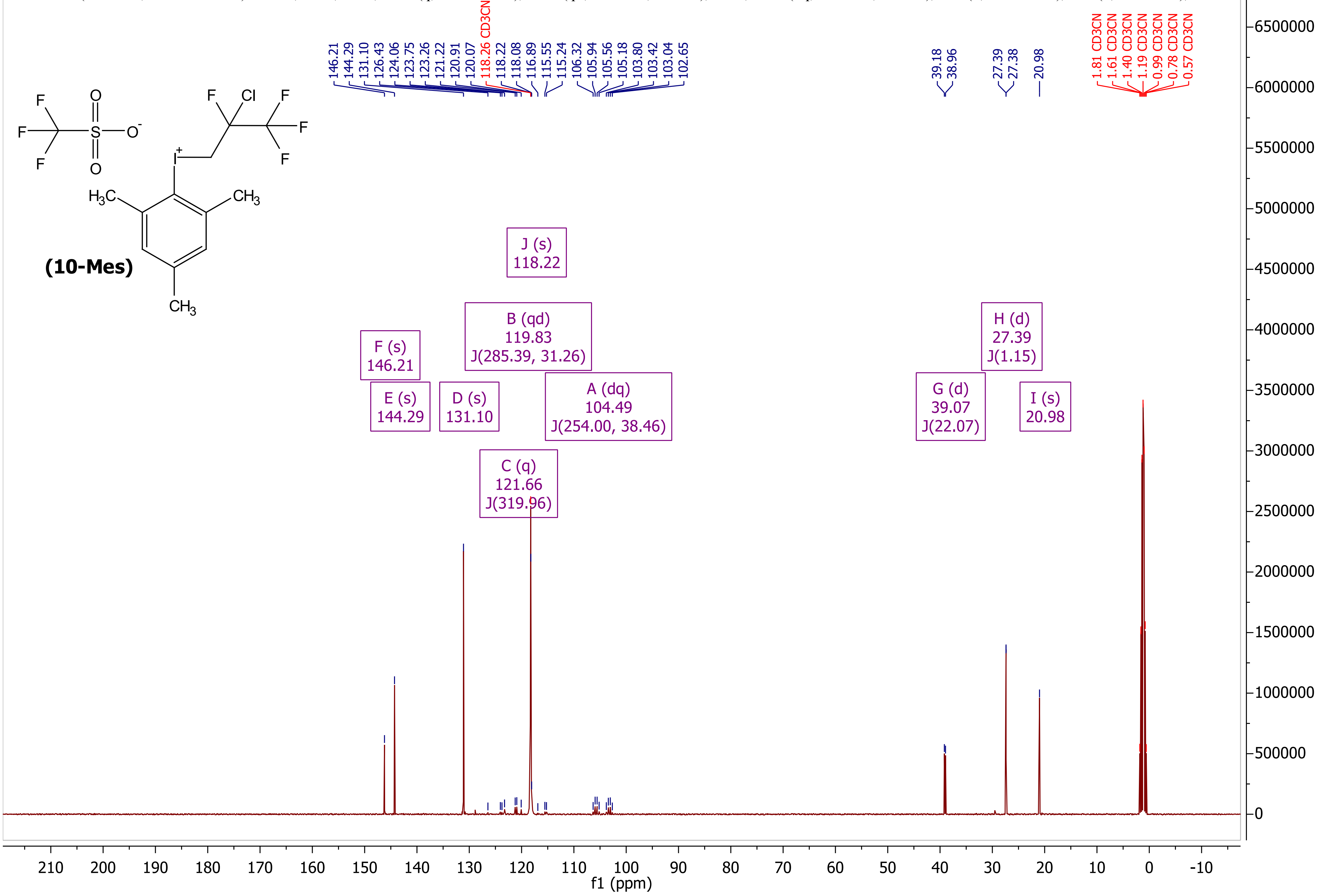

^{1}H NMR (250 MHz, Chloroform-*d*) δ 7.13 (t, *J* = 7.9 Hz, 2H), 6.72 (t, *J* = 7.3 Hz, 1H), 6.62 (d, *J* = 8.0 Hz, 2H), 3.93 (s, 1H), 3.90 (dd, *J* = 15.3, 12.3 Hz, 1H), 3.75 (dd, *J* = 21.2, 15.3 Hz, 1H).

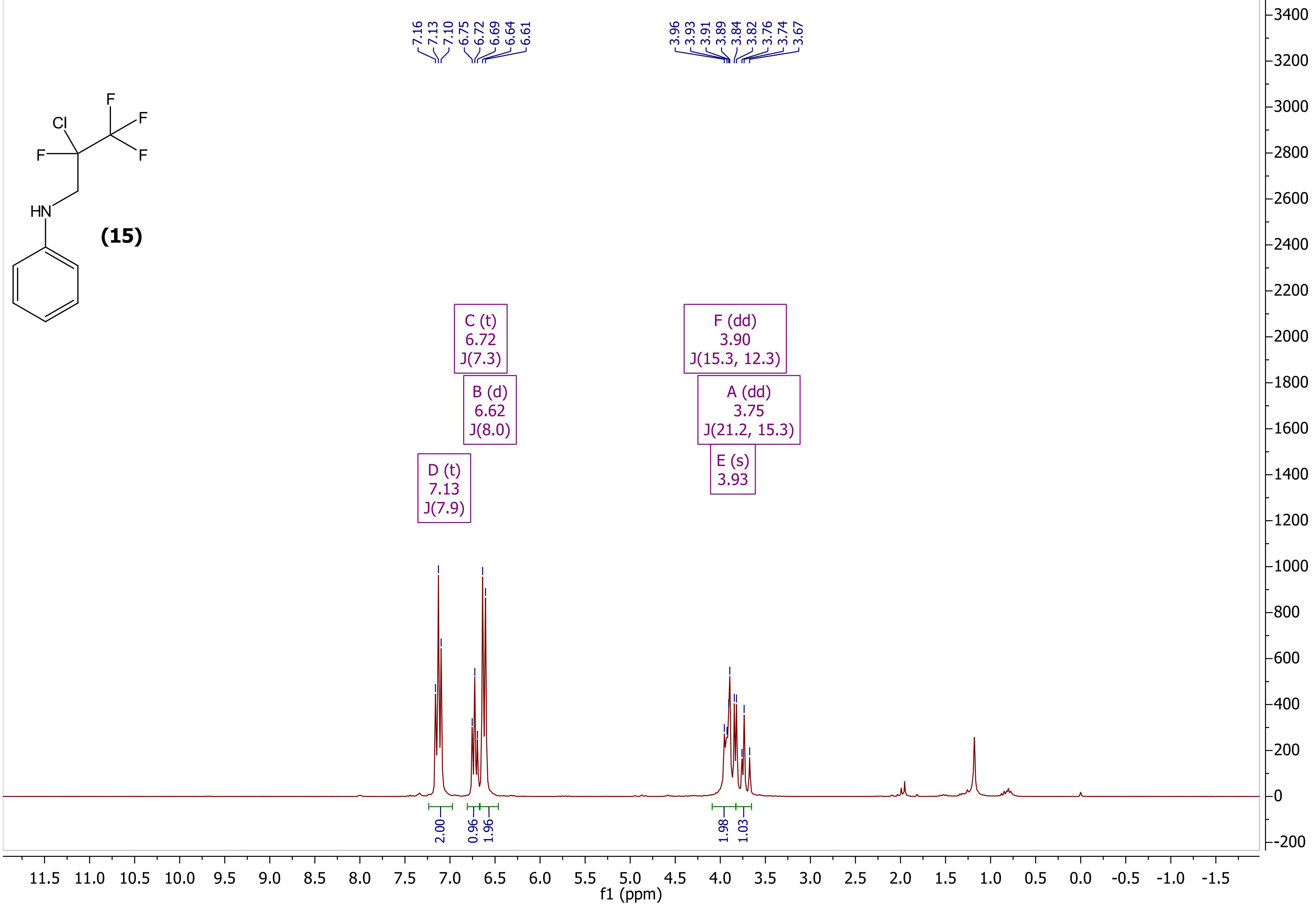

^{19}F NMR (235 MHz, Chloroform-*d*) δ -80.6 (d, *J* = 6.1 Hz), -130.2 (q, *J* = 6.2 Hz).

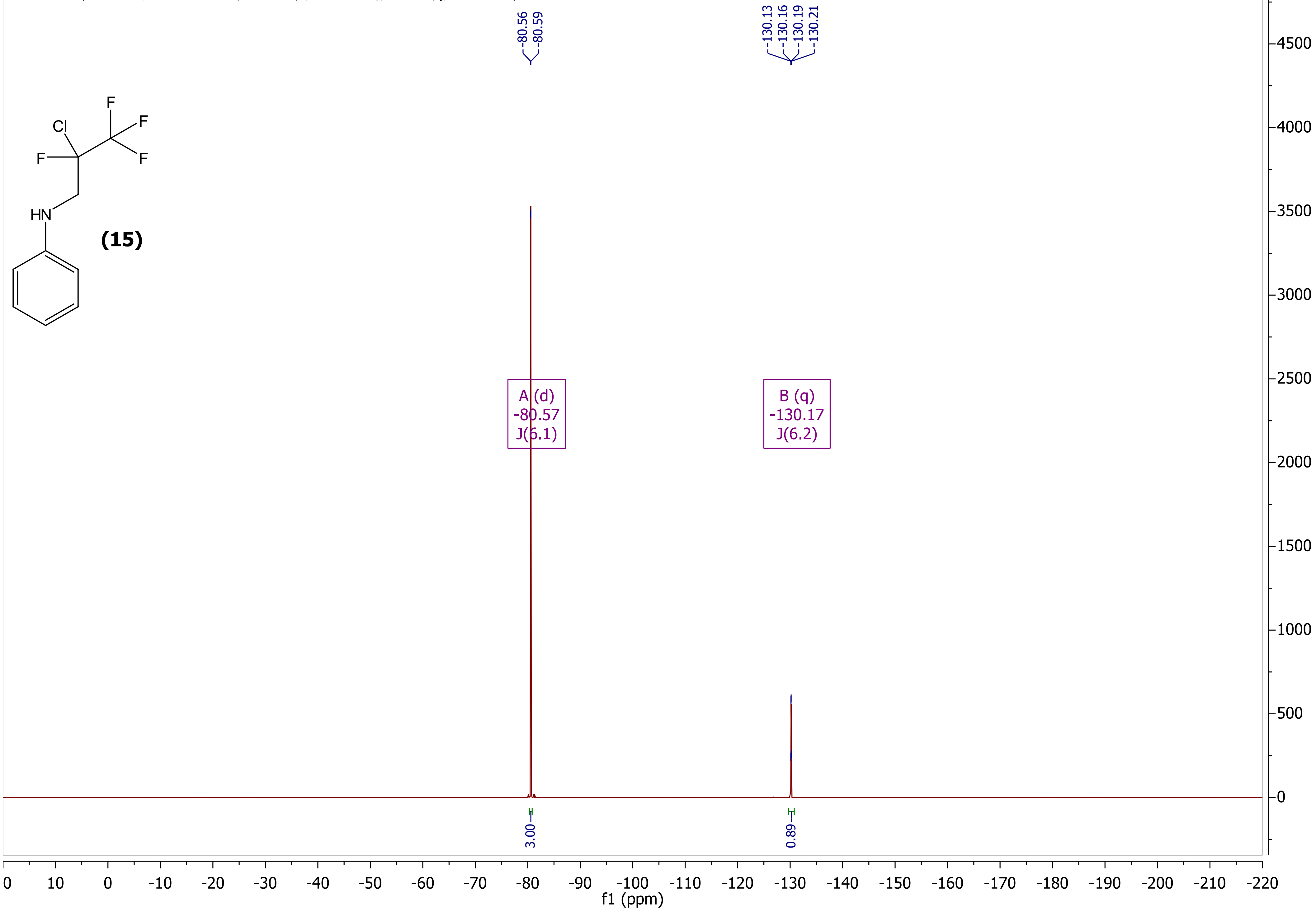

^{13}C NMR (63 MHz, Chloroform-*d*) δ 146.3, 129.6, 120.7 (qd, *J* = 284.9, 31.0 Hz), 119.4, 113.5, 107.0 (dq, *J* = 255.2, 34.5 Hz), 49.1 (d, *J* = 22.2 Hz).

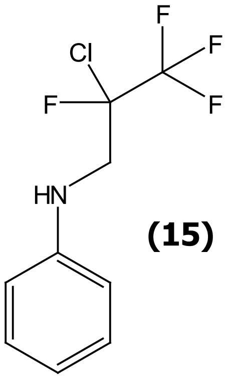

(15)

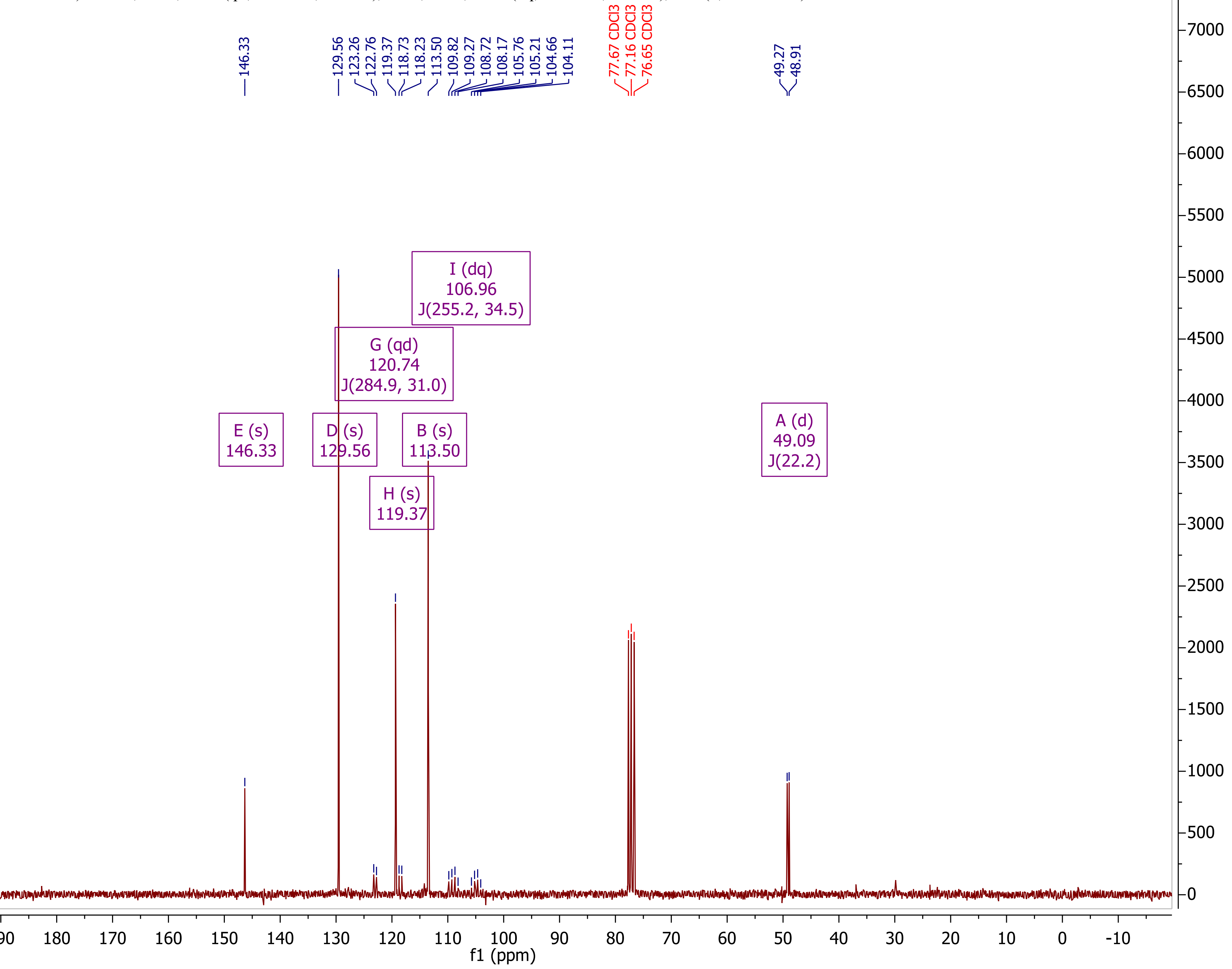

^{1}H NMR (250 MHz, Chloroform-*d*) δ 7.26 – 7.05 (m, 2H), 6.90 – 6.66 (m, 2H), 4.10 (dd, *J* = 15.1, 12.3 Hz, 1H), 3.94 (dd, *J* = 21.1, 15.0 Hz, 1H), 3.90 (s, 1H), 2.25 (s, 3H).

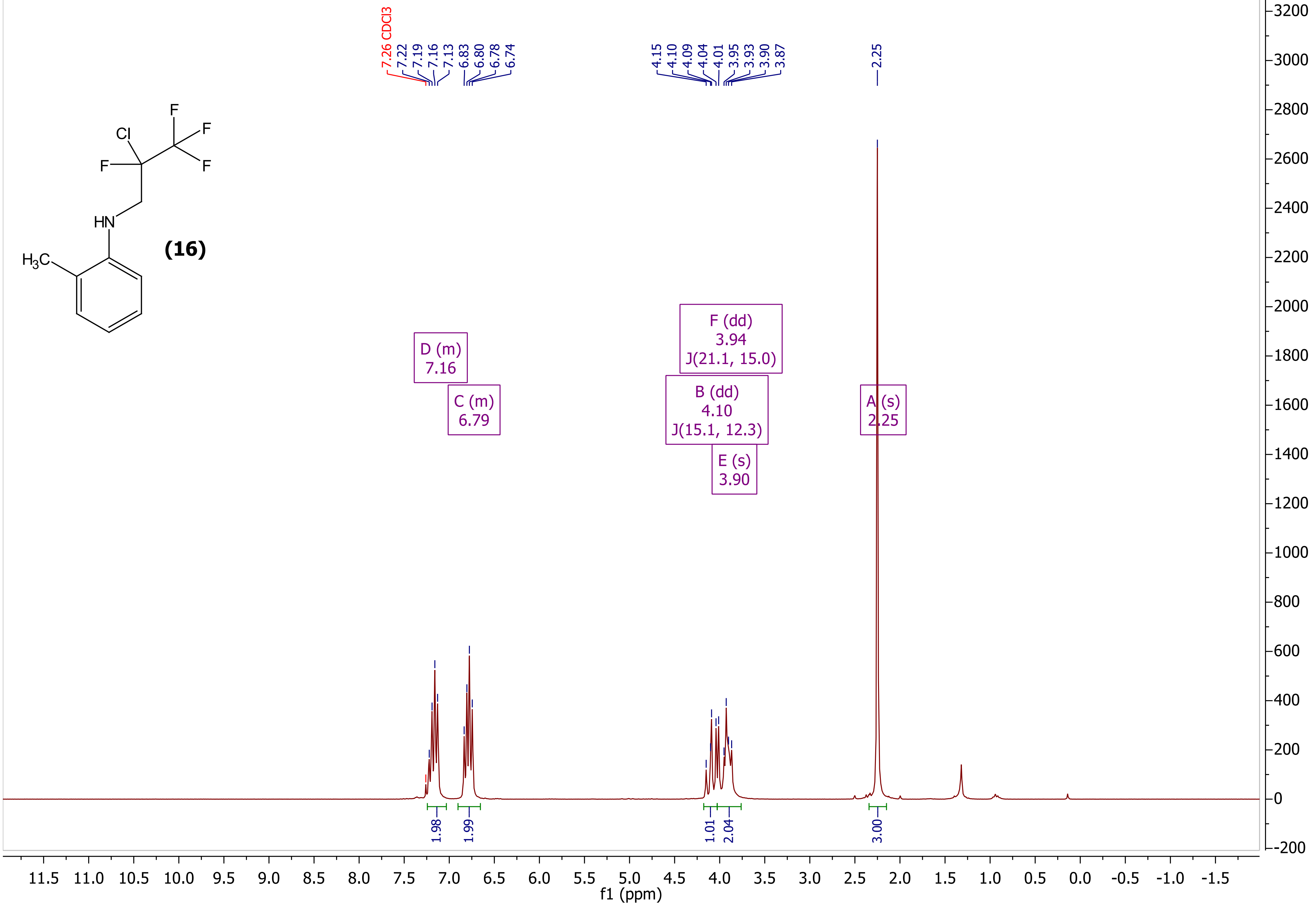

^{19}F NMR (235 MHz, Chloroform-*d*) δ -80.6 (d, *J* = 6.2 Hz), -130.1 (q, *J* = 6.2 Hz).

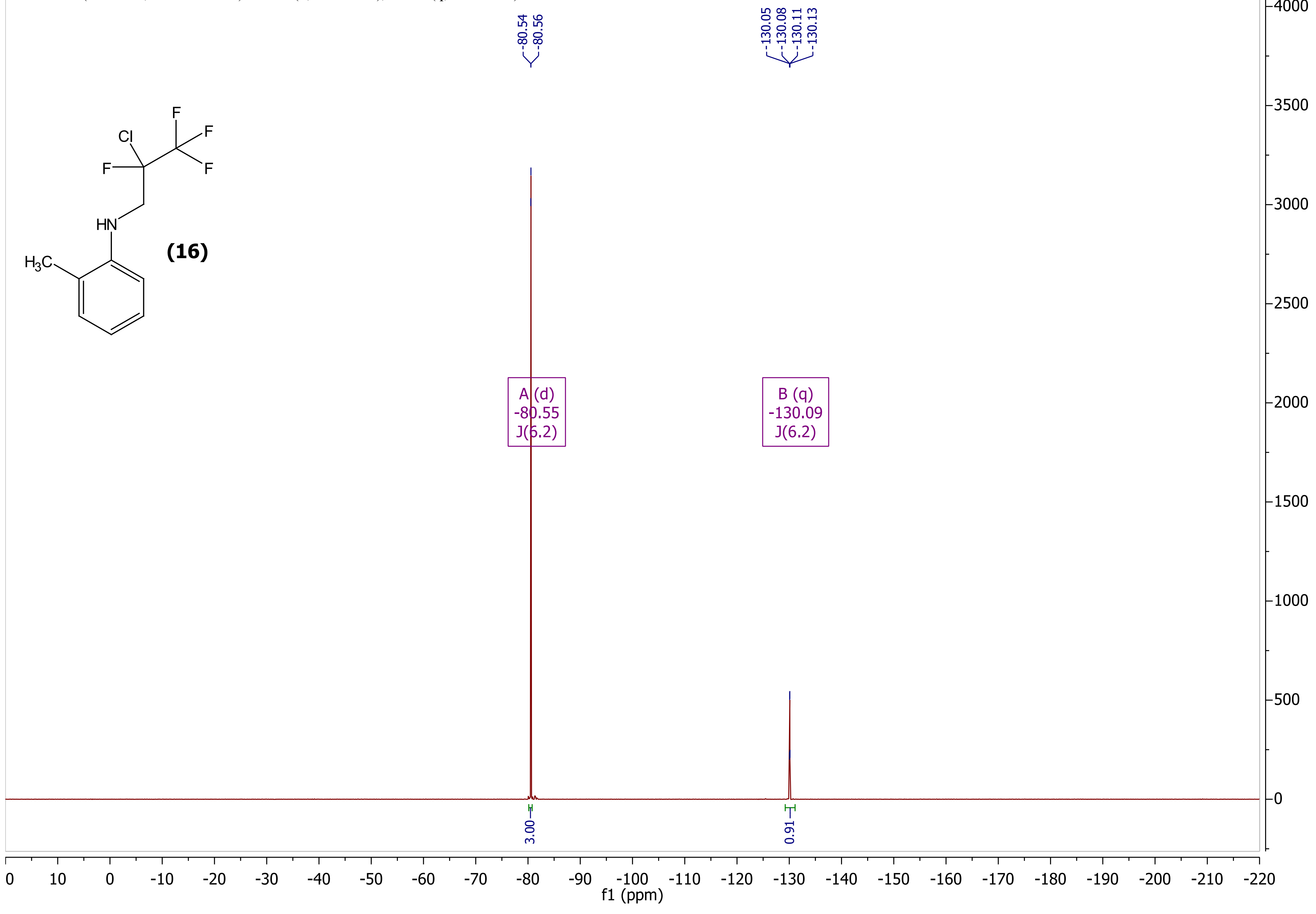

^{13}C NMR (63 MHz, Chloroform-*d*) δ 144.3, 130.7, 127.3, 122.8, 120.8 (qd, *J* = 284.6, 31.1 Hz), 119.0, 110.6 (d, *J* = 1.8 Hz), 107.0 (dq, *J* = 255.4, 34.8 Hz), 48.8 (d, *J* = 22.1 Hz), 17.5.

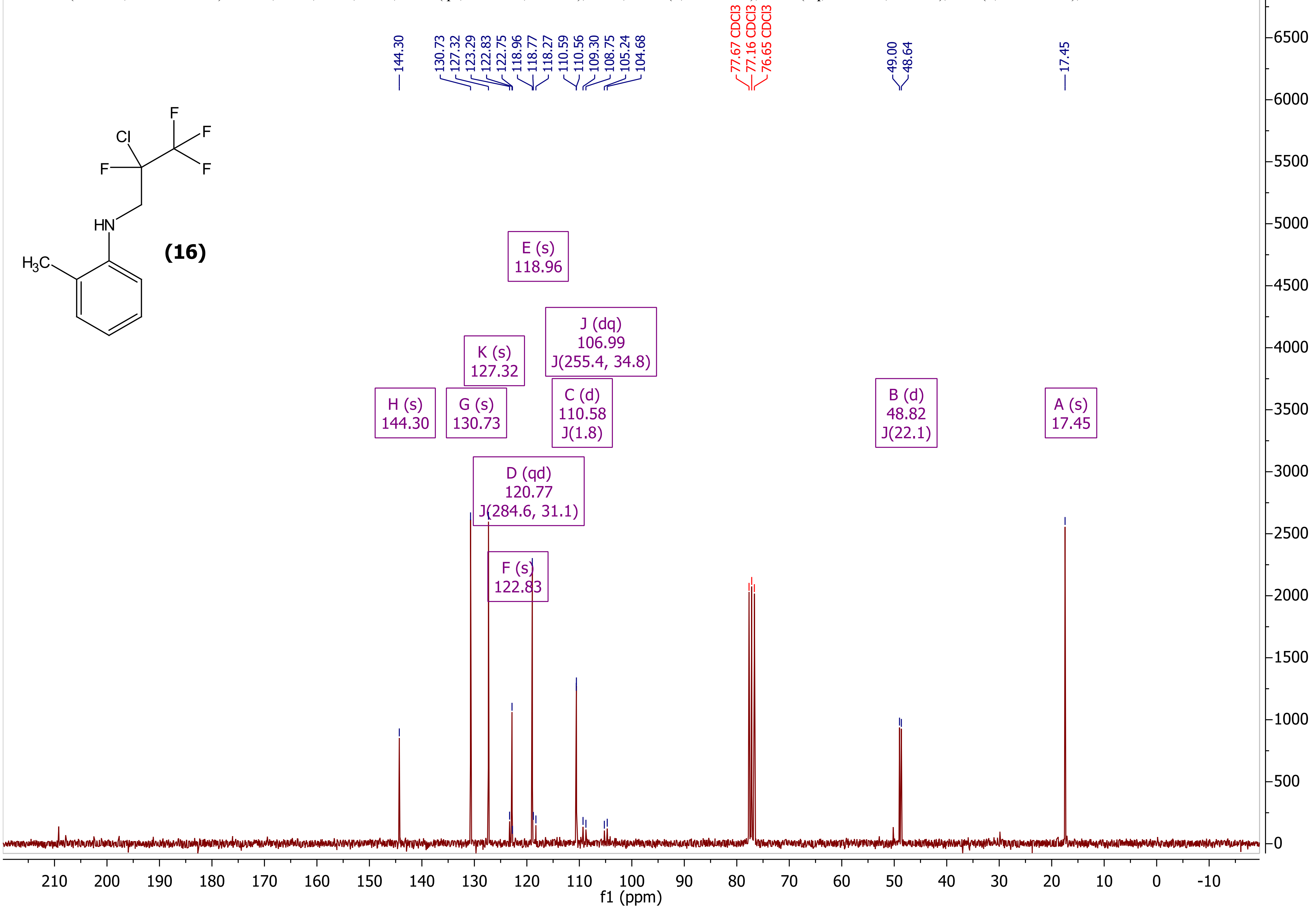

^{1}H NMR (250 MHz, Chloroform-*d*) δ 7.31 (dd, *J* = 7.9, 1.6 Hz, 1H), 7.18 (t, *J* = 7.8 Hz, 1H), 6.92 – 6.57 (m, 2H), 4.78 (bs, 1H), 4.08 (t, *J* = 13.9 Hz, 1H), 3.92 (dd, *J* = 21.0, 15.4 Hz, 1H).

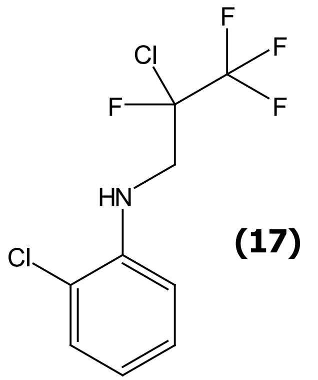

(17)

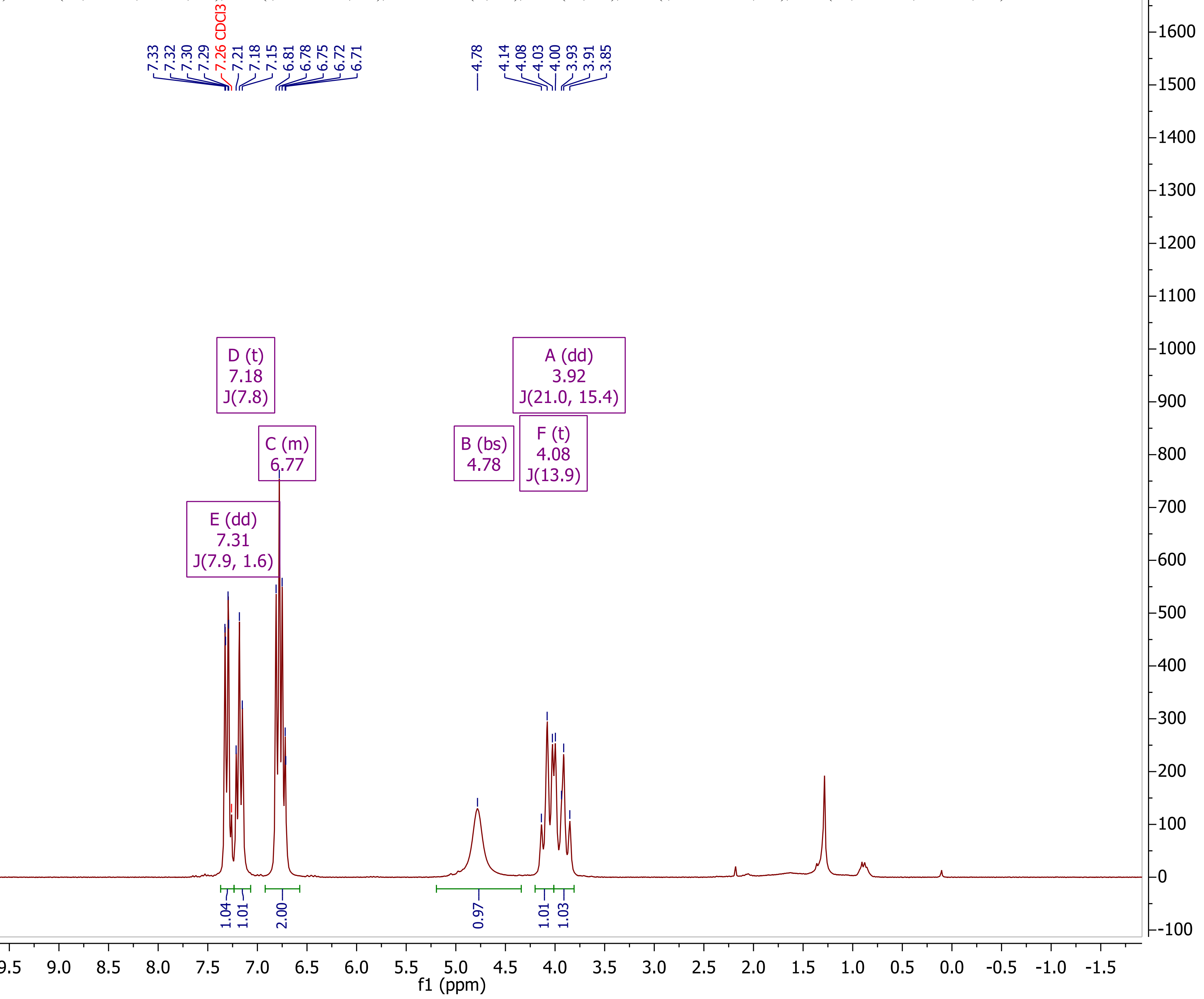

^{13}C NMR (63 MHz, Chloroform-*d*) δ 142.5, 129.7, 128.0, 120.7 (qd, *J* = 284.9, 31.1 Hz), 119.9, 119.2, 111.8 (d, *J* = 2.2 Hz), 106.8 (dq, *J* = 256.0, 34.6 Hz), 48.6 (d, *J* = 22.3 Hz).

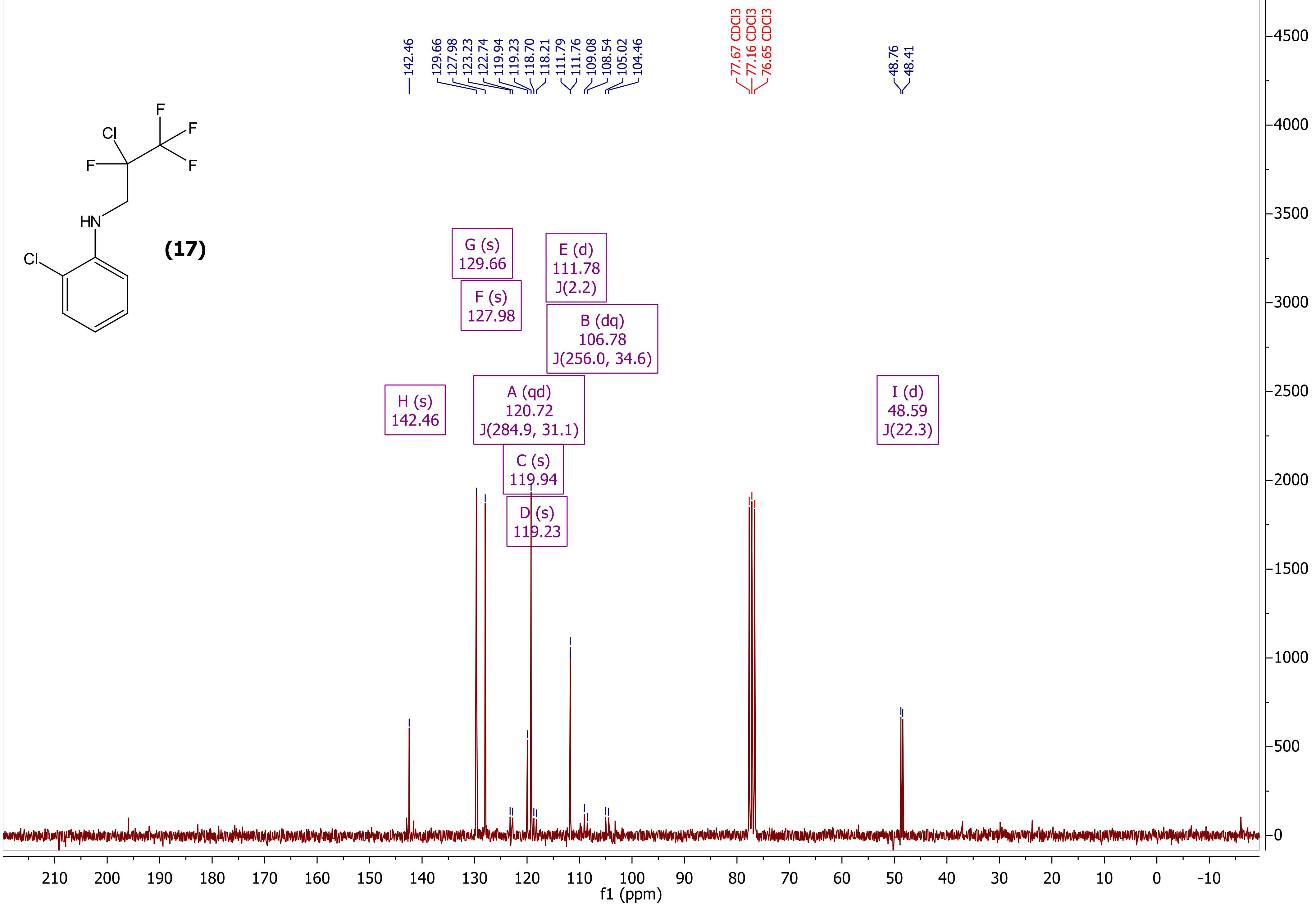

^{19}F NMR (235 MHz, Chloroform-*d*) δ -80.6 (d, *J* = 6.1 Hz), -130.2 (q, *J* = 6.2 Hz).

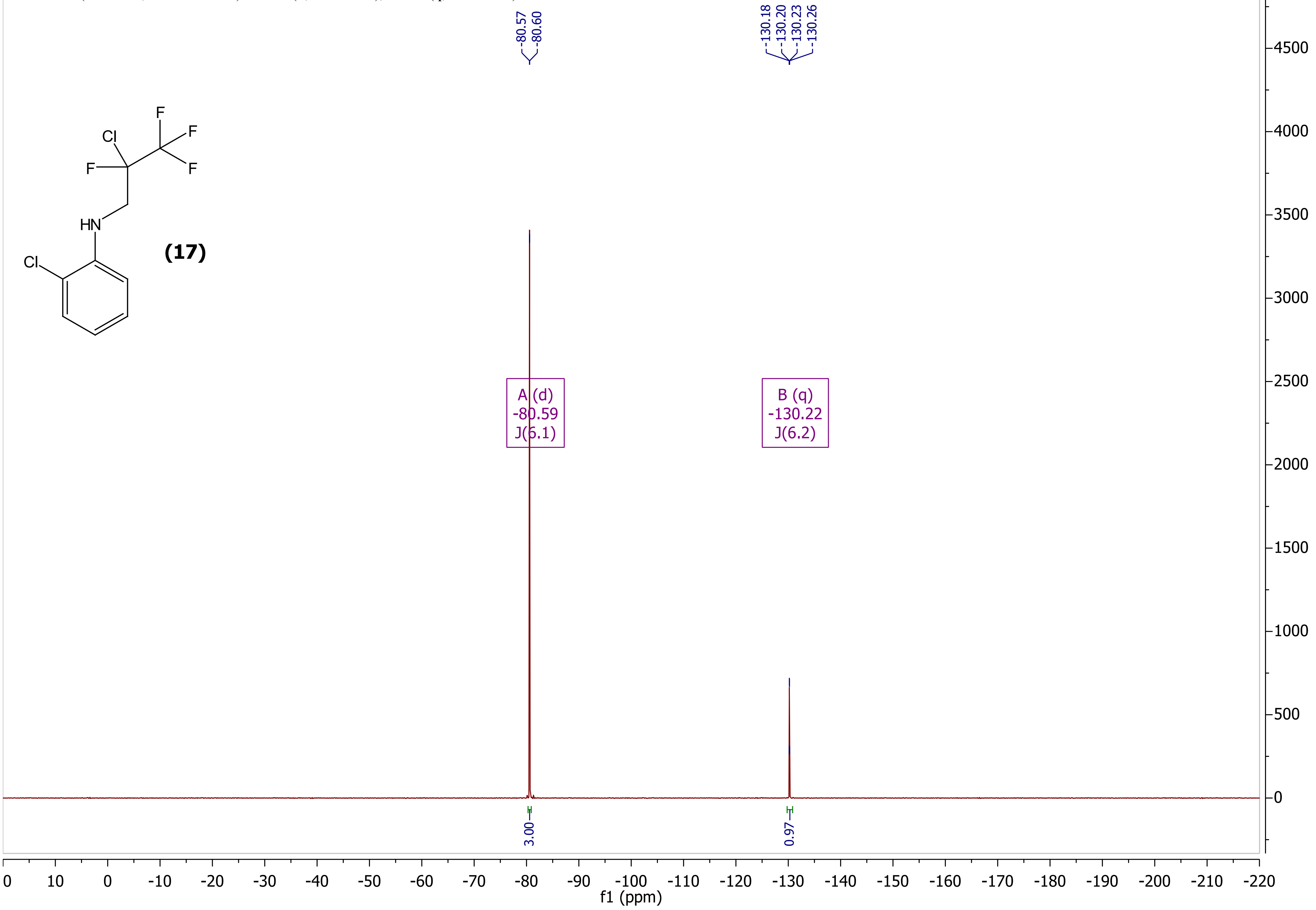

^{1}H NMR (250 MHz, Chloroform-d) δ 7.63 (dd, J = 7.8, 1.5 Hz, 1H), 7.24 – 7.01 (m, 1H), 6.63 (d, J = 8.2 Hz, 1H), 6.47 (td, J = 7.6, 1.4 Hz, 1H), 4.54 (bs, 1H), 3.99 (dd, J = 15.3, 12.1 Hz, 1H), 3.83 (dd, J = 21.1, 15.3 Hz, 1H).

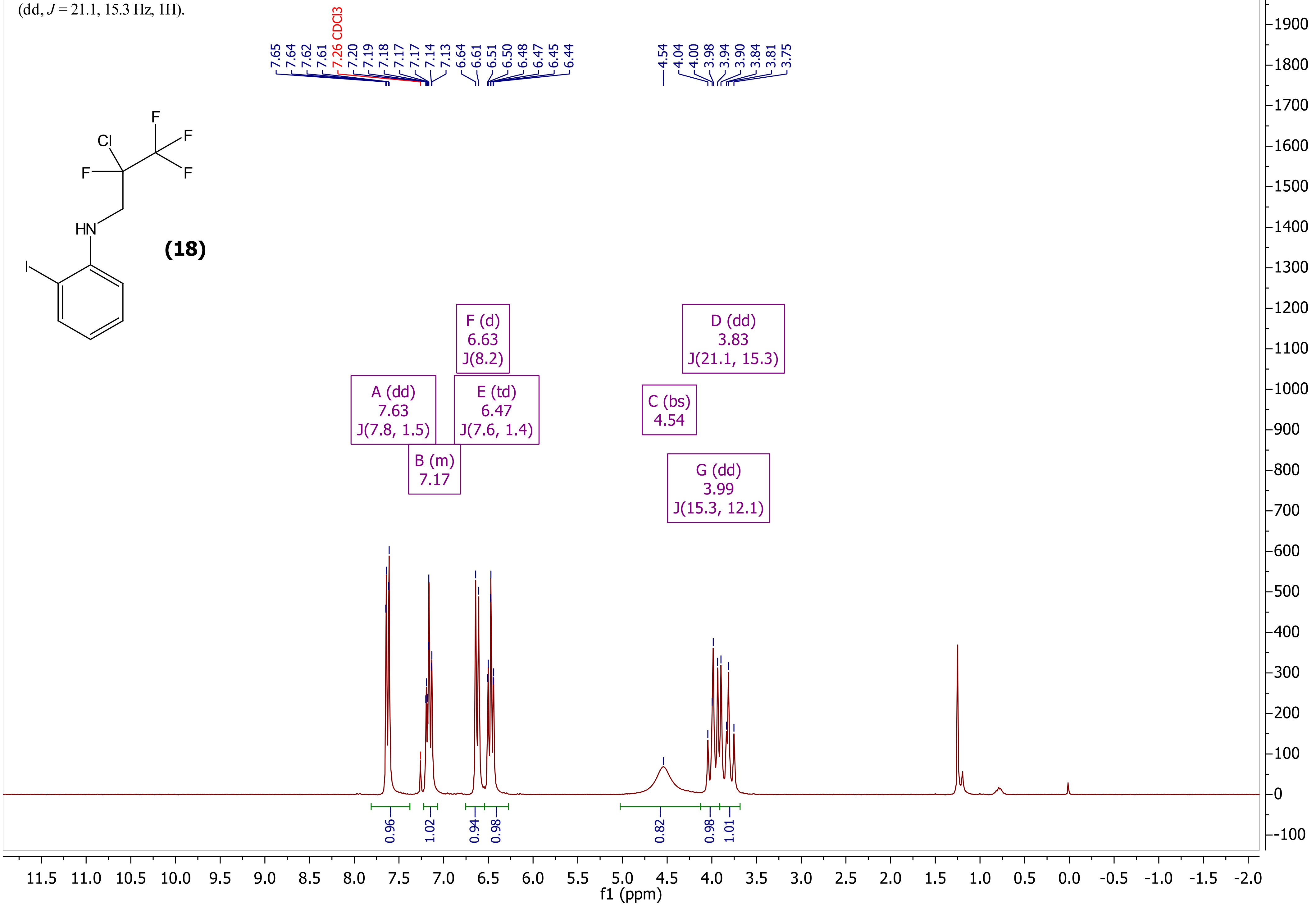

^{19}F NMR (235 MHz, Chloroform-*d*) δ -80.5 (d, *J* = 6.1 Hz), -130.1 (q, *J* = 6.1 Hz).

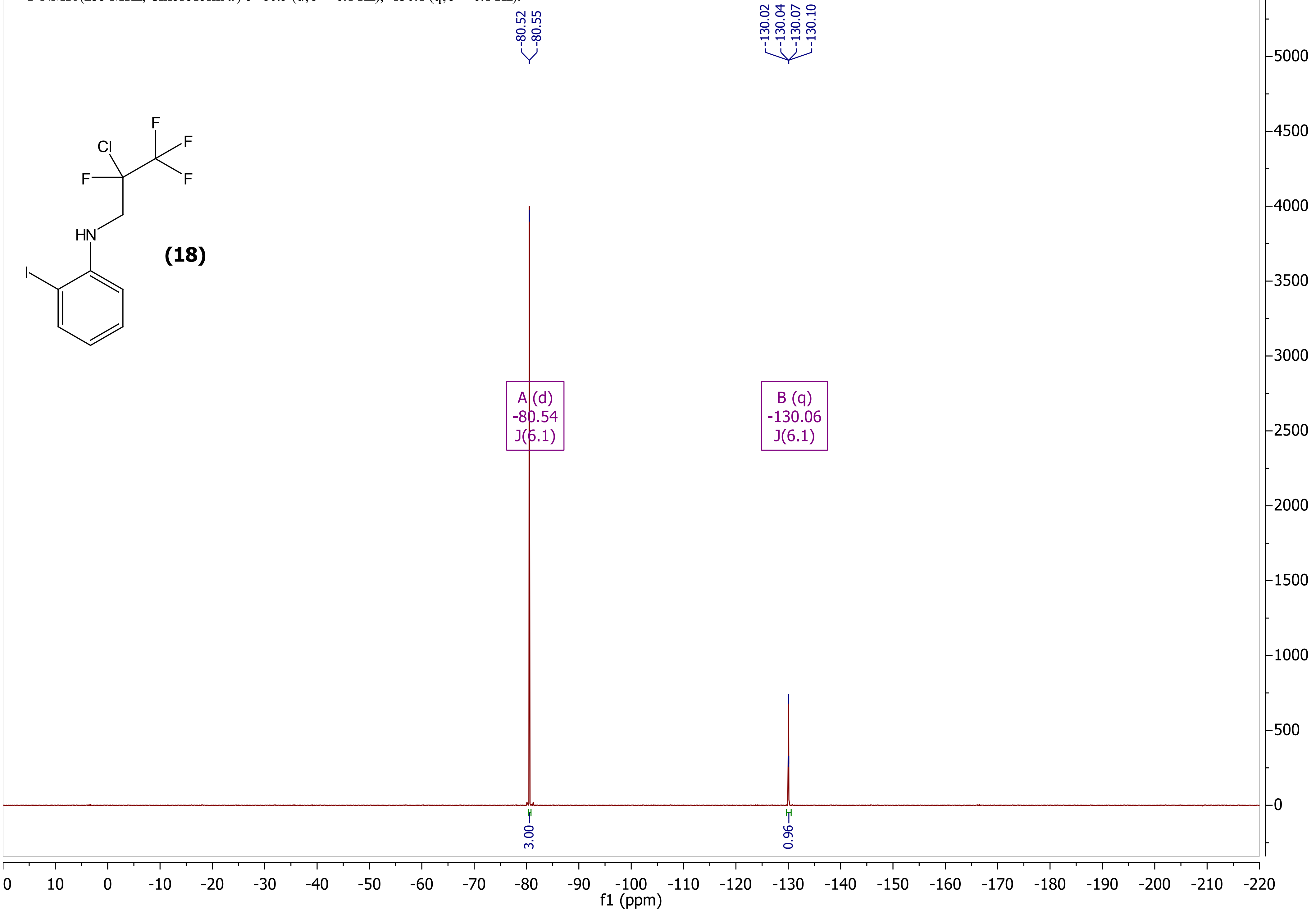

^{13}C NMR (63 MHz, Chloroform-*d*) δ 145.7, 139.6, 129.6, 120.7 (qd, *J* = 285.0, 30.9 Hz), 120.6, 111.2 (d, *J* = 2.3 Hz), 106.7 (dq, *J* = 255.8, 35.0 Hz), 86.0, 49.0 (d, *J* = 22.3 Hz).

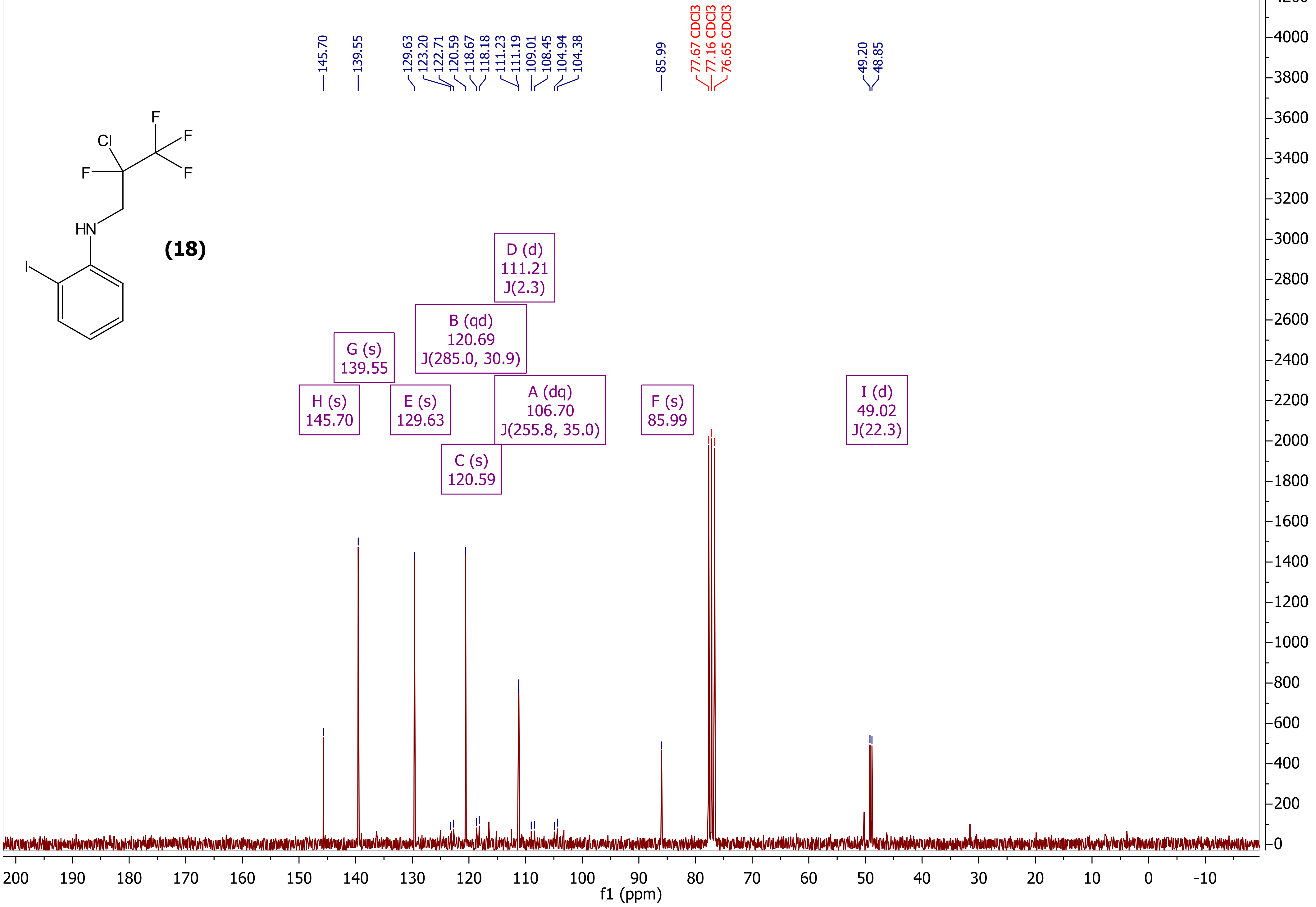

^{1}H NMR (250 MHz, Chloroform-*d*) δ 7.48 – 7.22 (m, 5H), 7.17 (td, *J* = 7.8, 1.7 Hz, 1H), 7.04 (dd, *J* = 7.5, 1.7 Hz, 1H), 6.83 – 6.61 (m, 2H), 3.89 (s, 1H), 3.87 (dd, *J* = 15.4, 12.4 Hz, 1H), 3.71 (dd, *J* = 21.0, 15.3 Hz, 1H).

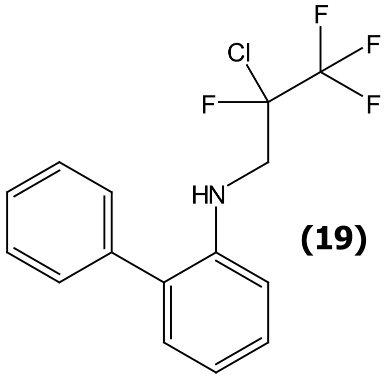

(19)

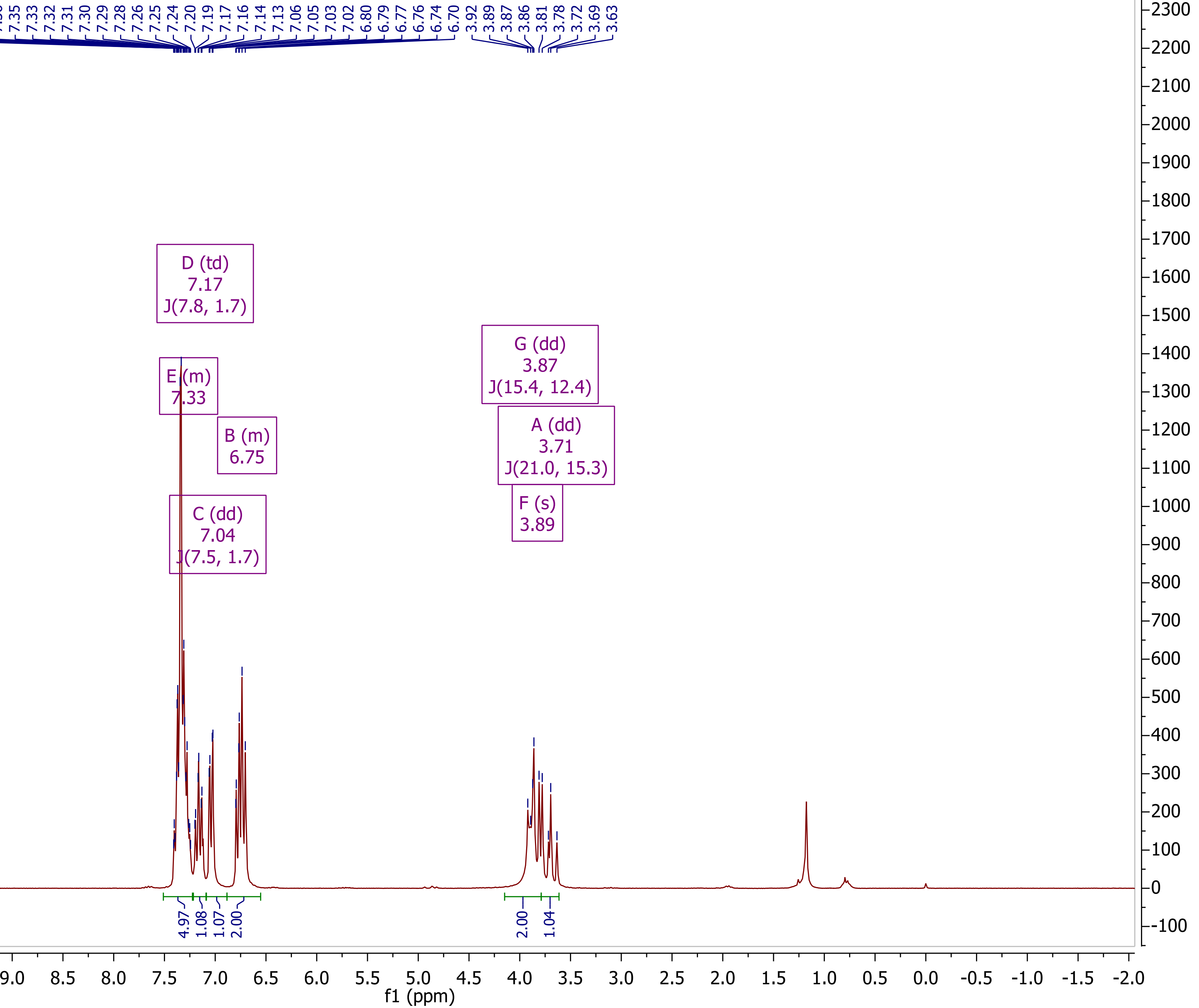

^{19}F NMR (235 MHz, Chloroform-*d*) δ -80.6 (d, *J* = 6.2 Hz), -129.9 (q, *J* = 6.1 Hz).

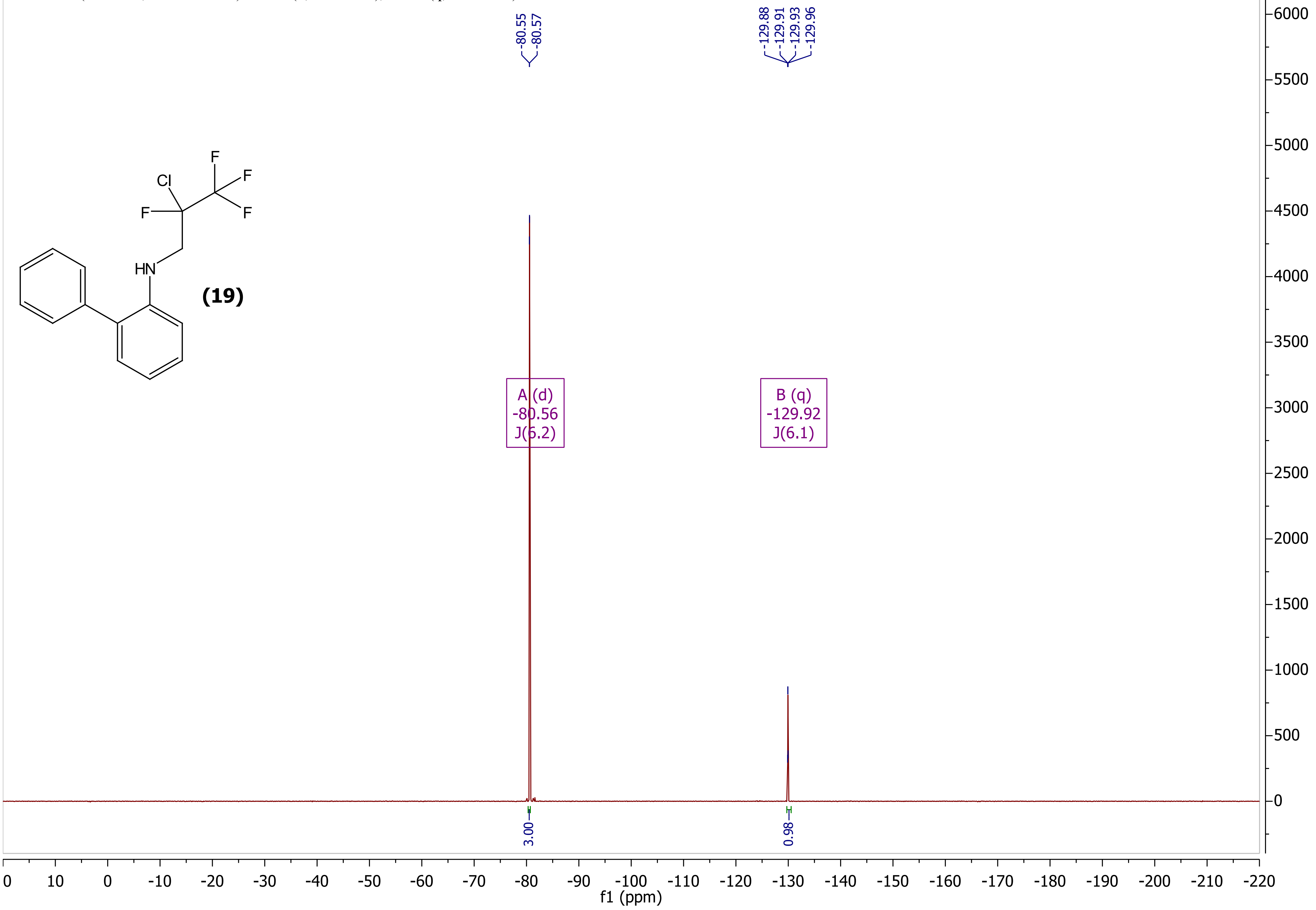

^{13}C NMR (63 MHz, Chloroform-*d*) δ 143.3, 138.9, 130.7, 129.5, 129.2, 128.8, 128.6, 127.7, 120.7 (qd, *J* = 285.0, 31.0 Hz), 118.8, 110.8 (d, *J* = 2.1 Hz), 107.1 (dq, *J* = 255.7, 34.6 Hz), 48.8 (d, *J* = 22.3 Hz).

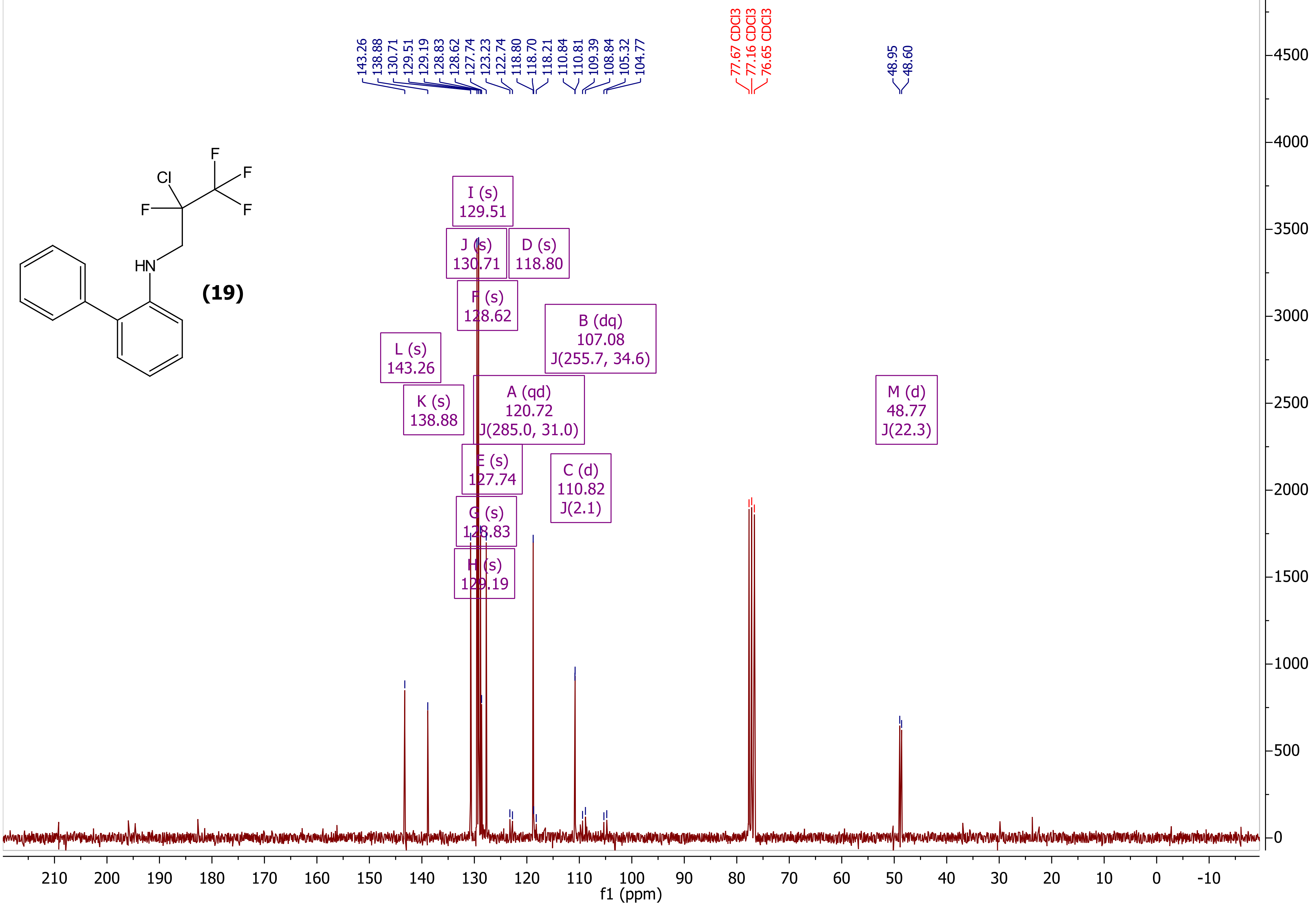

(19)

^{1}H NMR (250 MHz, Chloroform-*d*) δ 7.25 – 7.01 (m, 7H), 6.77 (t, *J* = 7.4 Hz, 1H), 6.64 (d, *J* = 8.1 Hz, 1H), 3.88 (s, 2H), 3.82 – 3.57 (m, 3H).

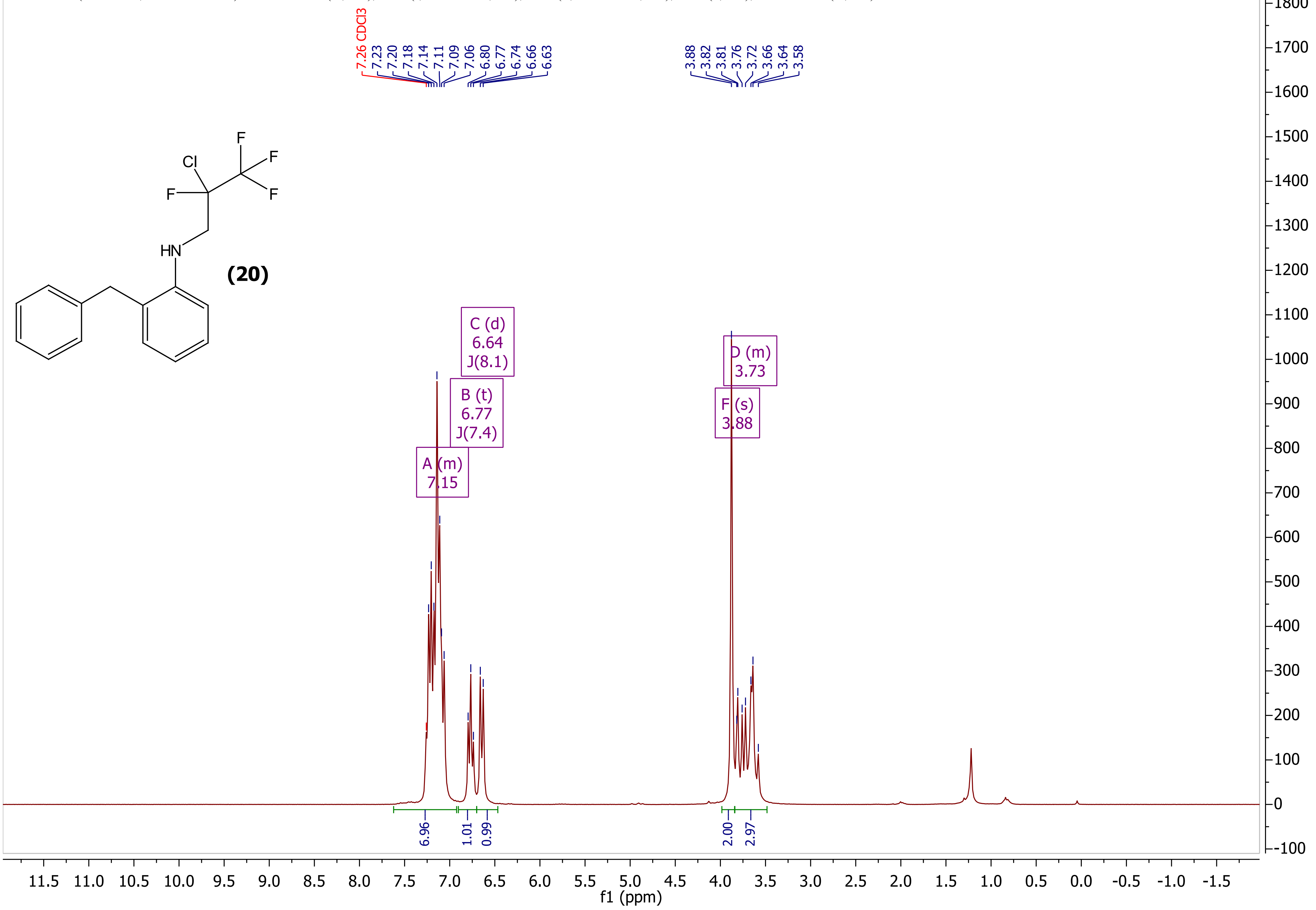

^{19}F NMR (235 MHz, Chloroform-*d*) δ -80.6 (d, *J* = 6.2 Hz), -130.0 (q, *J* = 6.2 Hz).

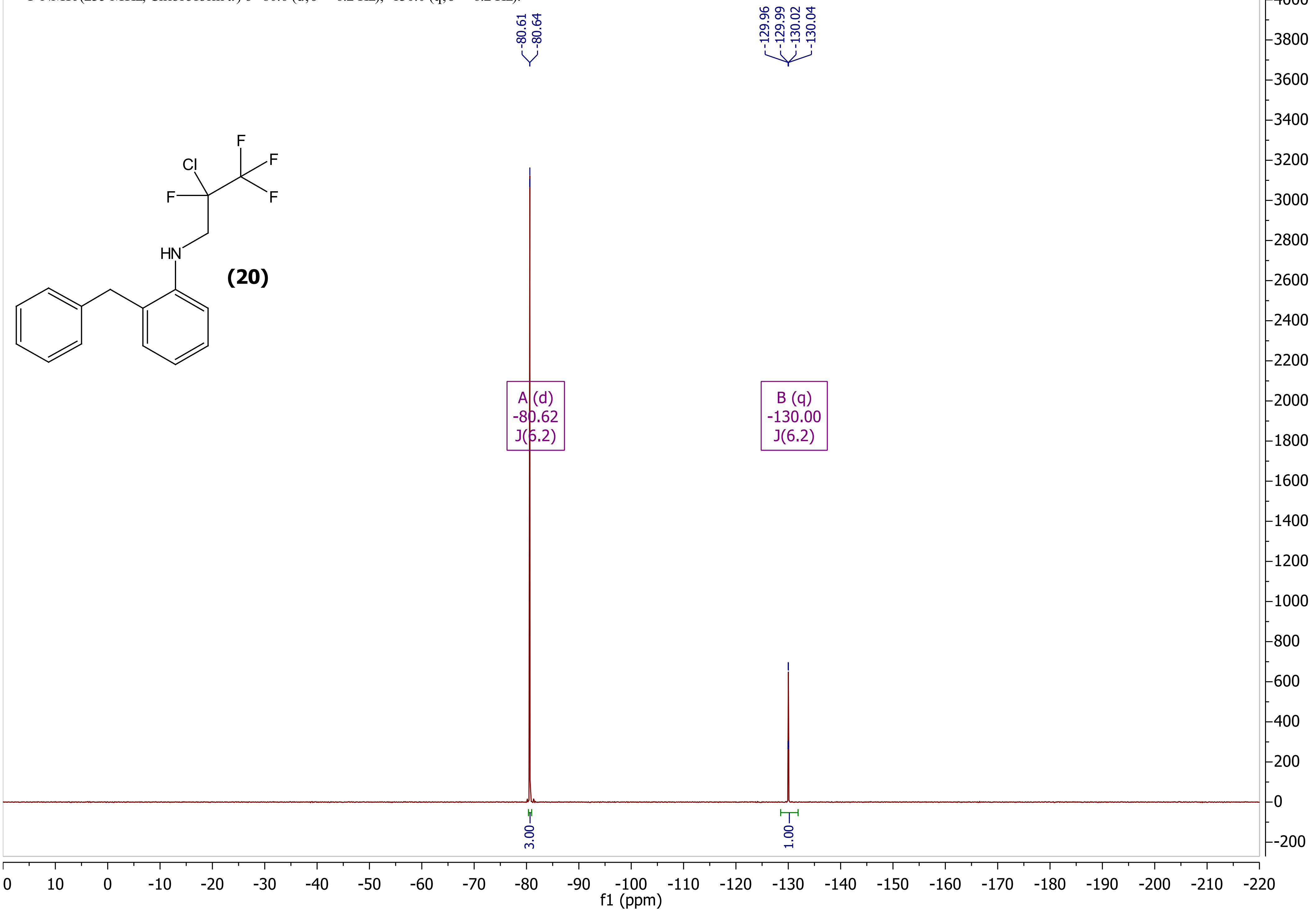

^{13}C NMR (63 MHz, Chloroform-*d*) δ 144.5, 139.0, 131.3, 129.0, 128.6, 128.0, 126.8, 125.8, 120.7 (qd, *J* = 285.0, 31.0 Hz), 119.1, 111.4 (d, *J* = 1.8 Hz), 106.8 (dq, *J* = 255.4, 34.9 Hz), 48.5 (d, *J* = 22.4 Hz), 38.5.

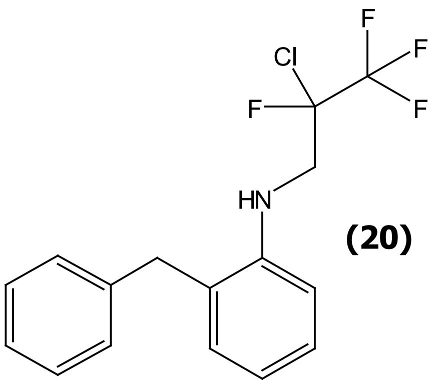

(20)

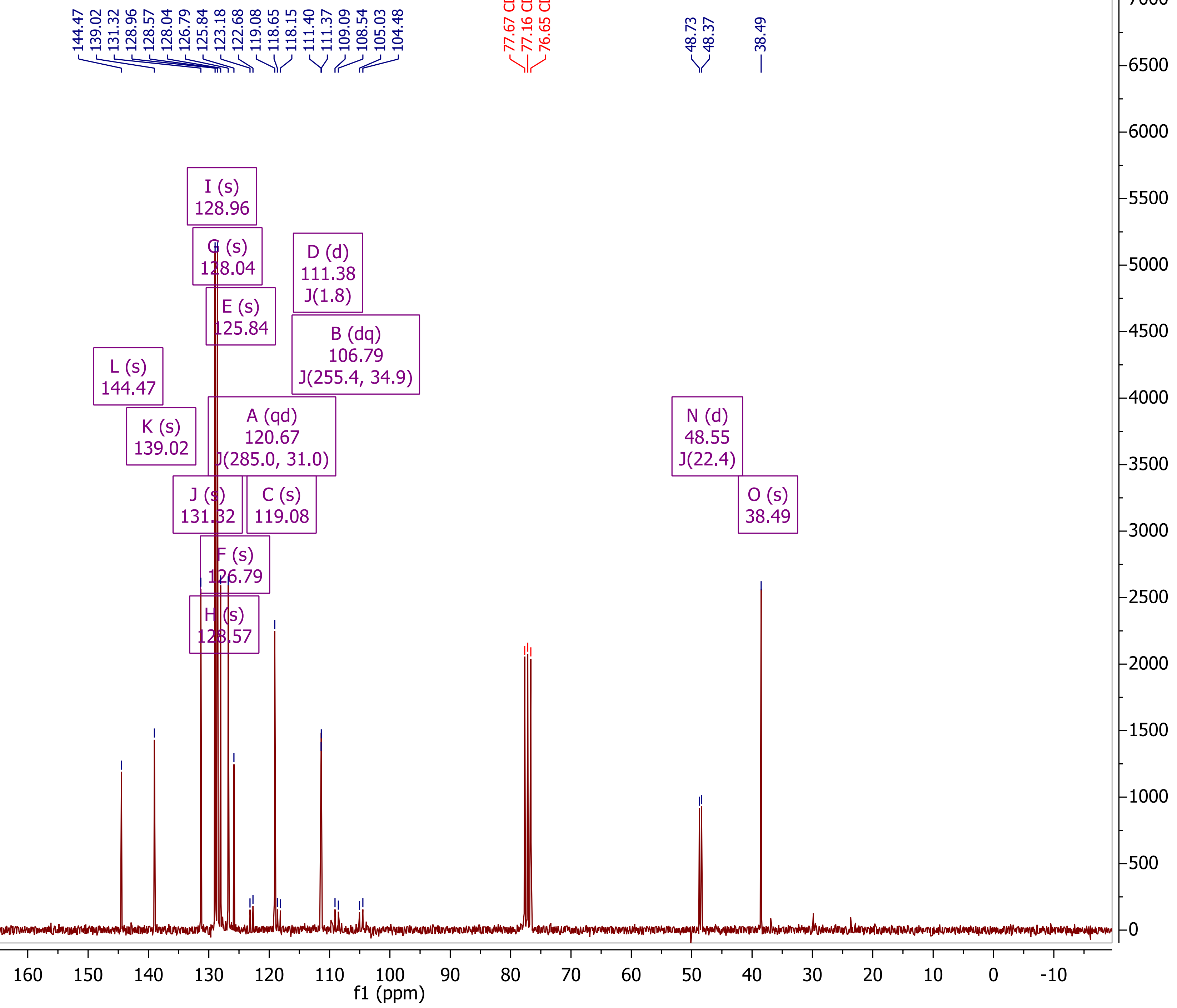

^{1}H NMR (250 MHz, Chloroform-d) δ 7.32 (dd, J = 7.5, 1.6 Hz, 1H), 7.20 (td, J = 7.8, 1.6 Hz, 1H), 6.81 – 6.59 (m, 2H), 4.94 (bs, 1H), 4.08 (dd, J = 15.3, 12.5 Hz, 1H), 3.93 (dd, J = 20.6, 15.2 Hz, 1H), 2.52 (t, J = 6.8 Hz, 2H), 1.78 – 1.38 (m, 4H), 0.99 (t, J = 7.1 Hz, 3H).

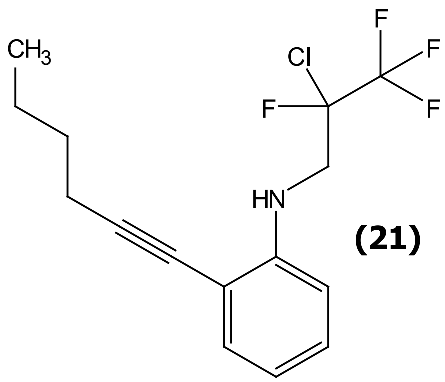

(21)

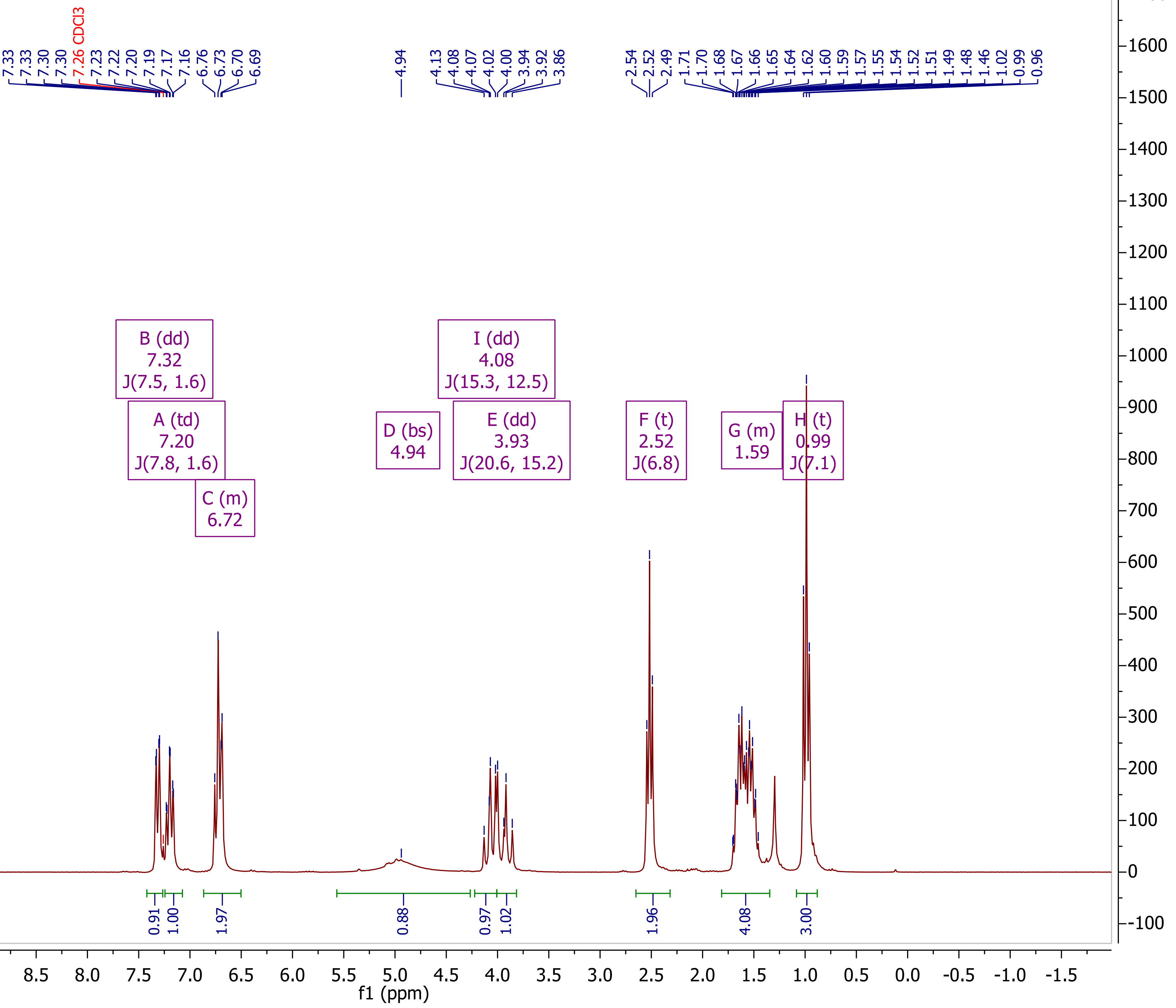

^{19}F NMR (235 MHz, Chloroform-*d*) δ -80.6 (d, *J* = 6.2 Hz), -130.3 (q, *J* = 6.1 Hz).

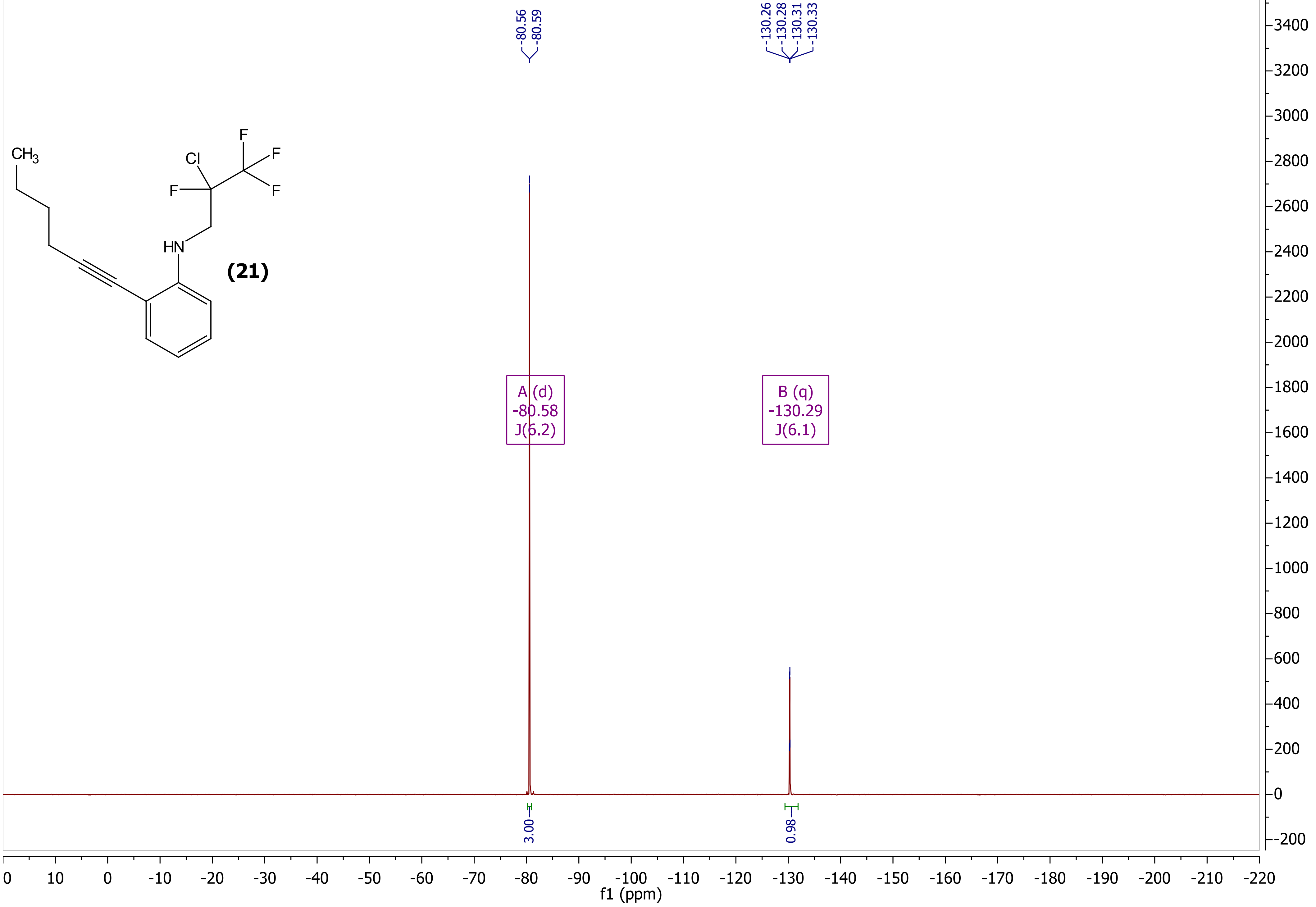

^{13}C NMR (63 MHz, Chloroform-*d*) δ 147.1, 132.2, 129.1, 120.8 (qd, *J* = 284.9, 31.0 Hz), 118.2, 109.8, 109.8, 106.9 (dq, *J* = 255.6, 34.8 Hz), 97.0, 76.5, 48.6 (d, *J* = 22.5 Hz), 31.0, 22.1, 19.4, 13.7.

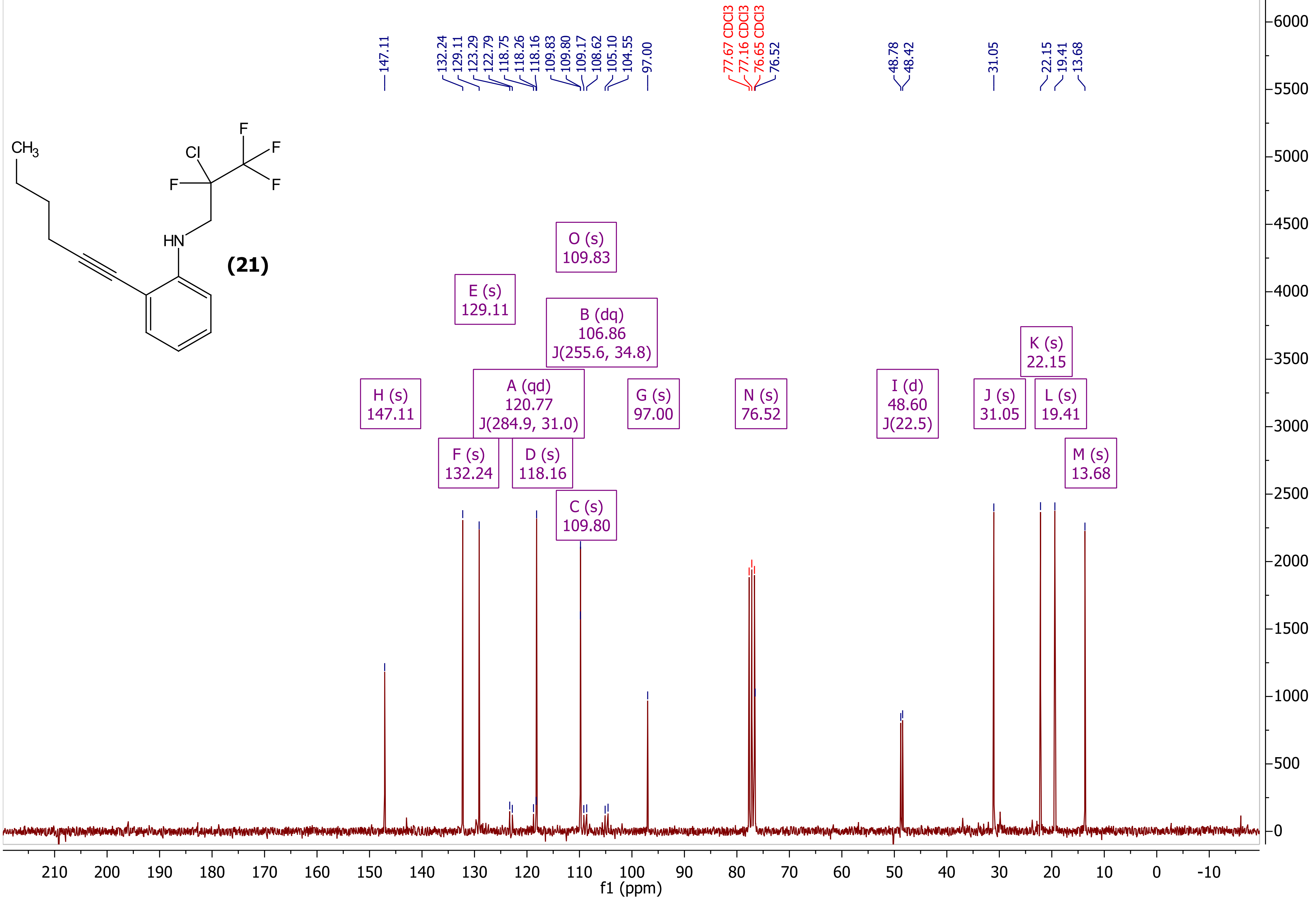

^{1}H NMR (500 MHz, DMSO-d_6) δ 9.46 (s, 1H), 6.73 – 6.62 (m, 3H), 6.50 (td, J = 7.1, 2.1 Hz, 1H), 5.27 (t, J = 7.2 Hz, 1H), 4.18 – 3.87 (m, 2H).

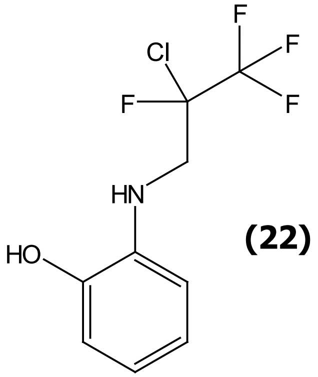

(22)

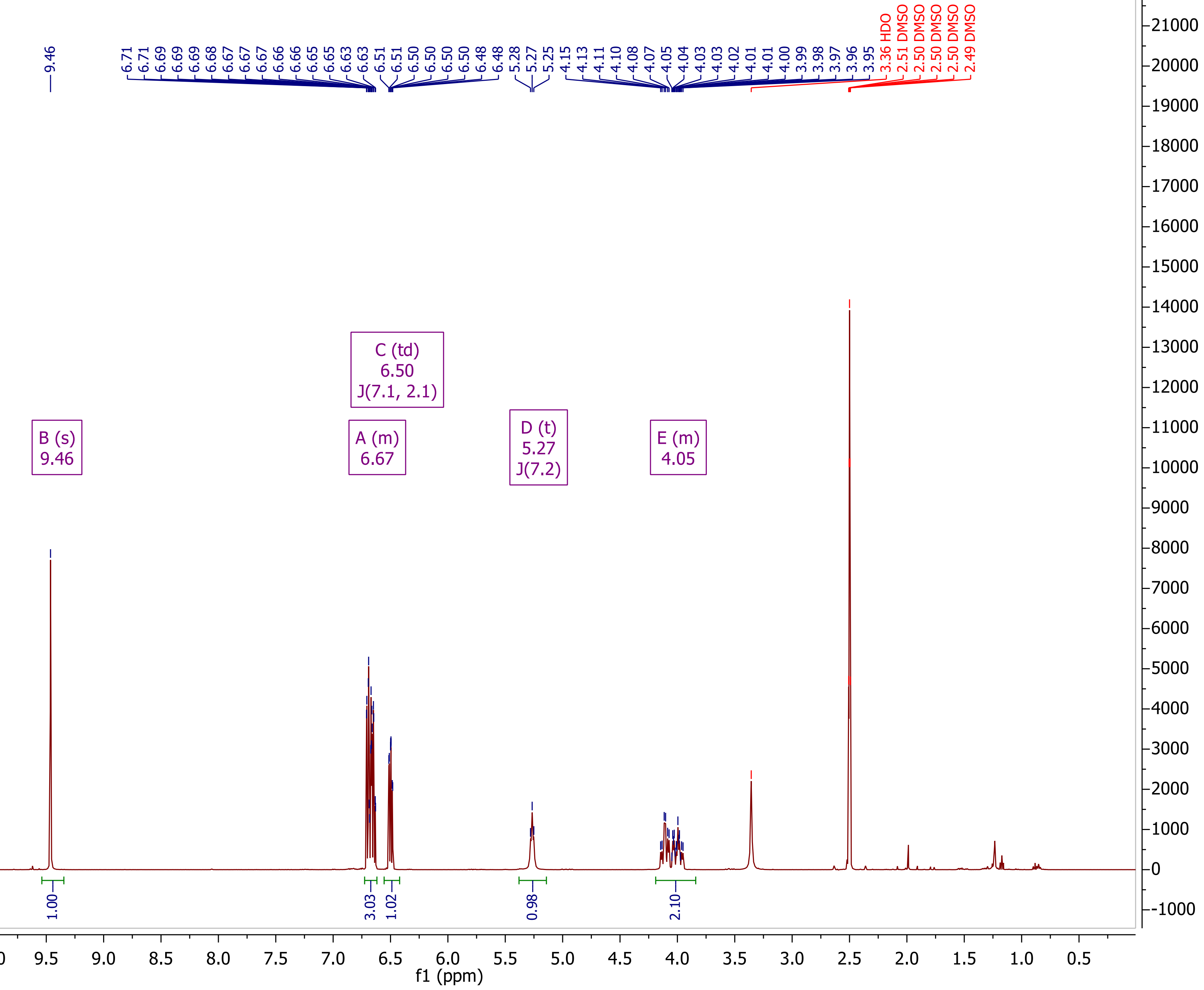

^{19}F NMR (376 MHz, DMSO-d_6) δ -79.5 (d, J = 6.4 Hz), -128.4 – -128.8 (m).

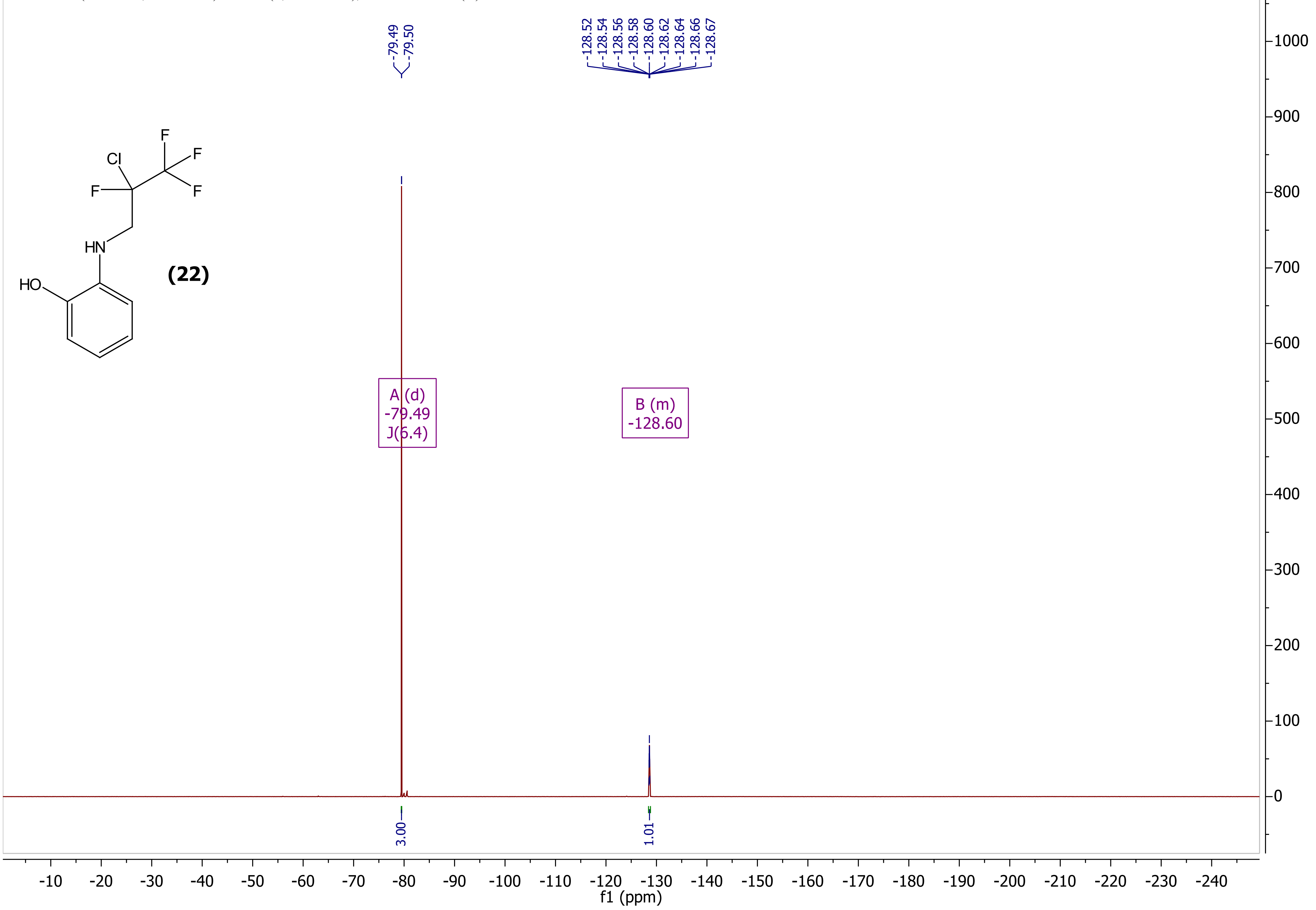

^{13}C NMR (126 MHz, DMSO-d_6) δ 144.2, 135.9, 120.6 (qd, J = 284.9, 31.5 Hz), 119.5, 117.3, 113.8, 110.3 (d, J = 2.7 Hz), 107.7 (dq, J = 254.0, 33.3 Hz), 47.6 (d, J = 21.2 Hz).

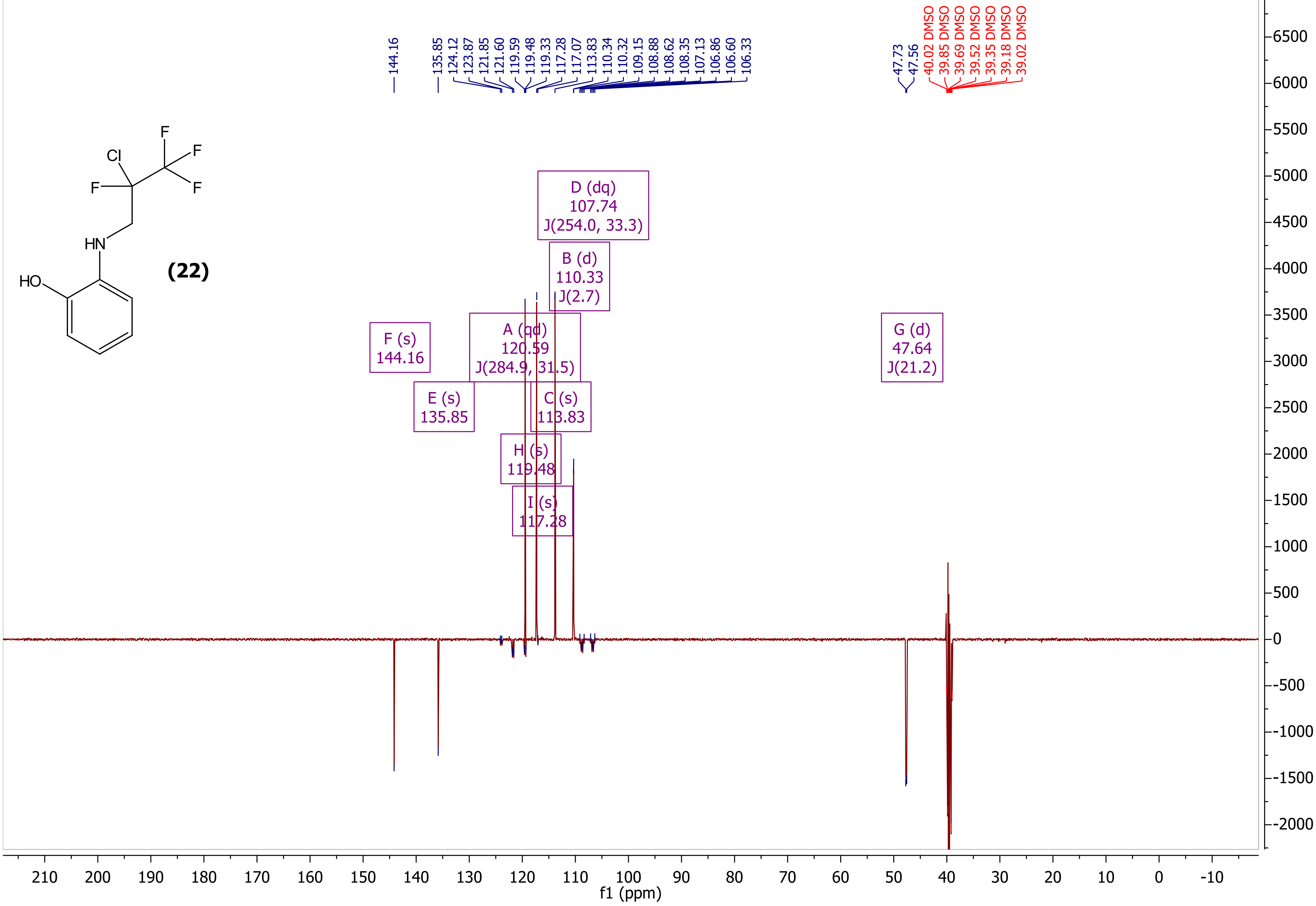

^{1}H NMR (250 MHz, Chloroform-*d*) δ 7.13 (t, *J* = 8.0 Hz, 1H), 6.79 (dd, *J* = 8.0, 1.9 Hz, 1H), 6.70 (t, *J* = 2.2 Hz, 1H), 6.59 (dd, *J* = 8.3, 2.4 Hz, 1H), 4.10 – 3.62 (m, 3H).

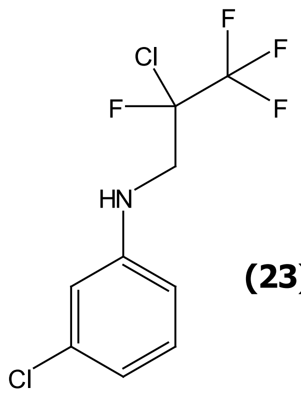

(23)

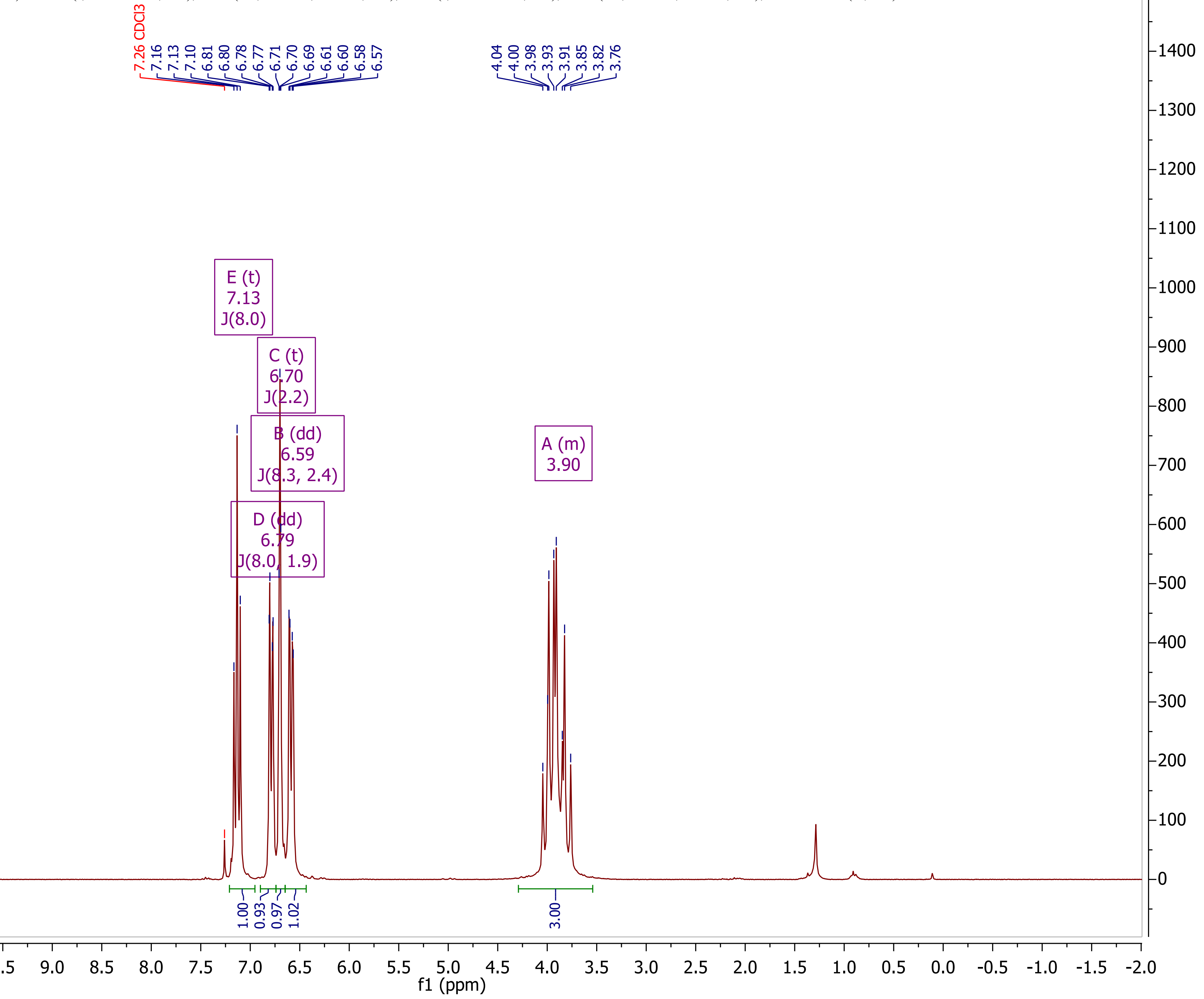

^{19}F NMR (235 MHz, Chloroform-*d*) δ -80.6 (d, *J* = 6.2 Hz), -130.3 (q, *J* = 6.1 Hz).

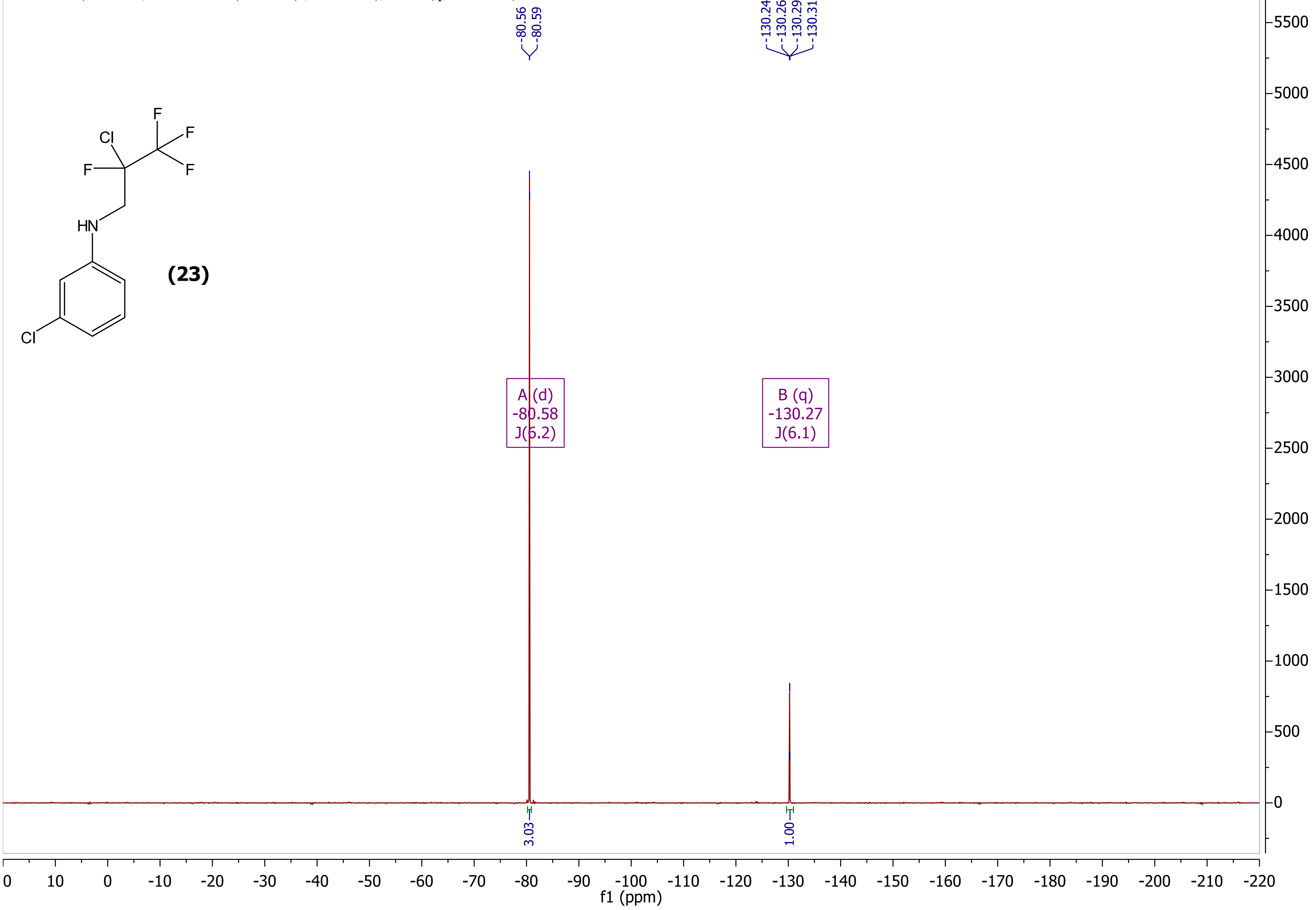

^{13}C NMR (63 MHz, Chloroform-*d*) δ 147.6, 135.3, 130.5, 120.7 (qd, *J* = 284.8, 31.0 Hz), 119.2, 113.3, 111.6, 106.8 (dq, *J* = 255.6, 35.0 Hz), 48.7 (d, *J* = 22.2 Hz).

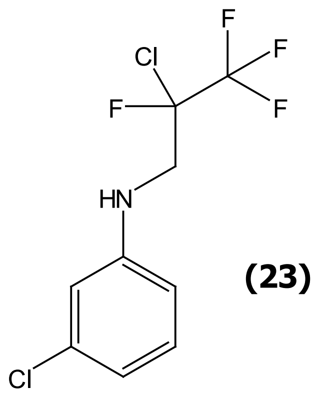

(23)

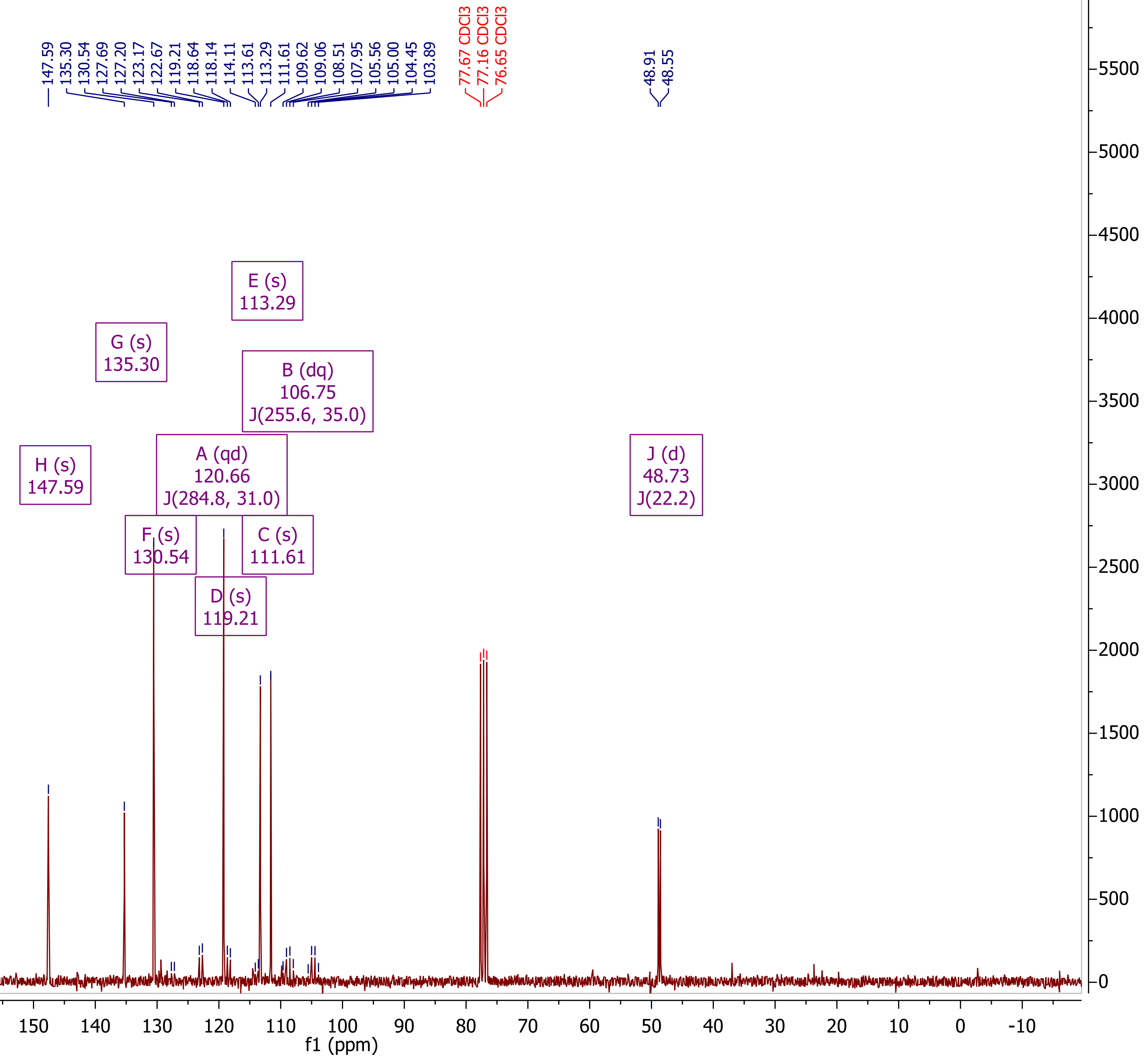

^{1}H NMR (250 MHz, Chloroform-*d*) δ 7.47 (dd, *J* = 7.7, 1.3 Hz, 1H), 7.40 (s, 1H), 7.27 (t, *J* = 7.9 Hz, 1H), 6.90 (dd, *J* = 8.1, 2.6 Hz, 1H), 4.13 – 3.76 (m, 6H).

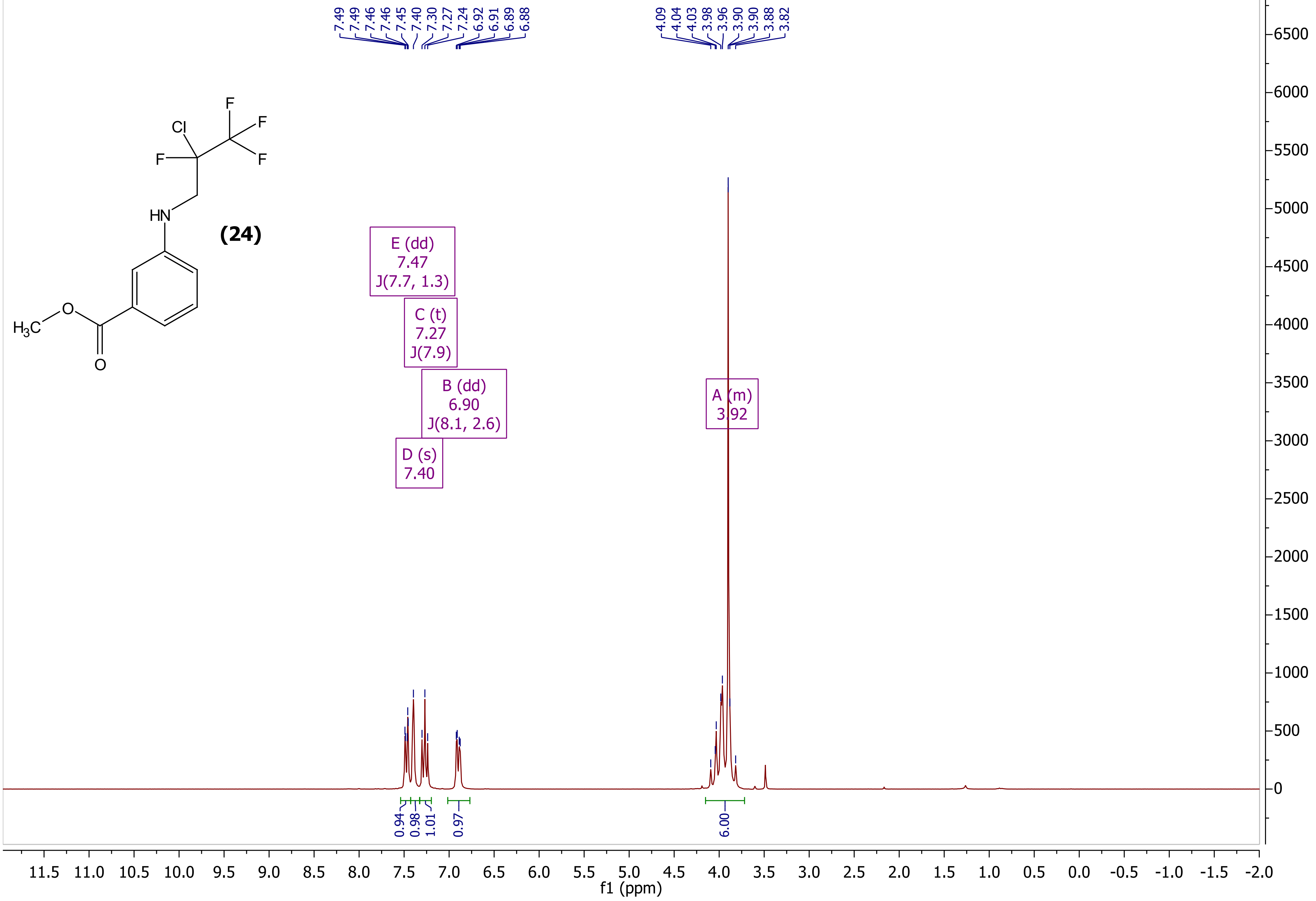

^{19}F NMR (235 MHz, Chloroform-*d*) δ -80.6 (d, *J* = 6.1 Hz), -130.2 (q, *J* = 6.1 Hz).

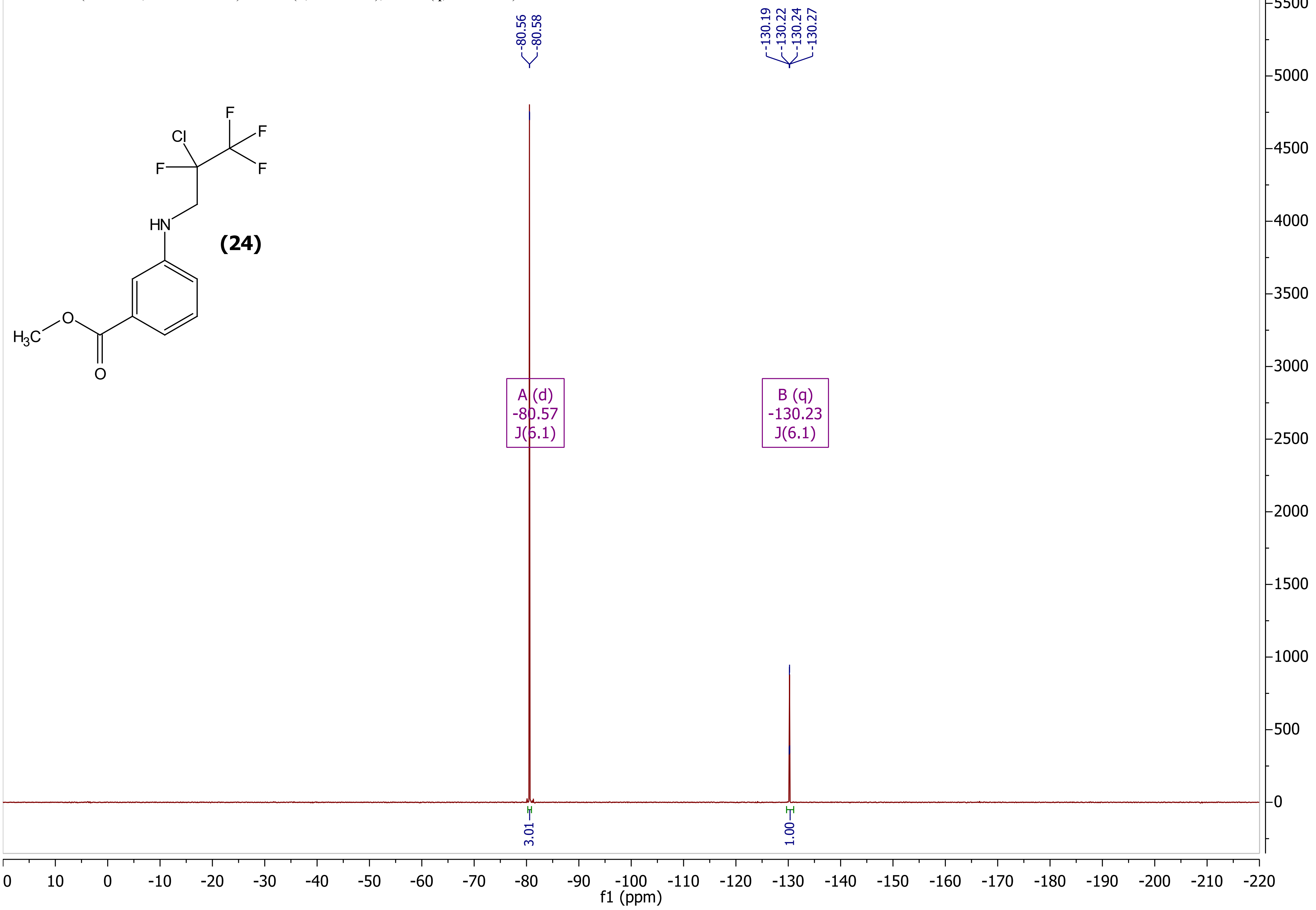

^{13}C NMR (63 MHz, Chloroform-*d*) δ 167.3, 146.5, 131.4, 129.5, 120.7 (qd, *J* = 284.9, 31.0 Hz), 120.3, 117.7, 114.1, 106.8 (dq, *J* = 255.4, 34.7 Hz), 52.2, 48.8 (d, *J* = 22.2 Hz).

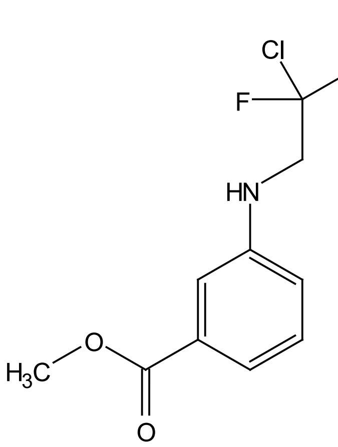

(24)

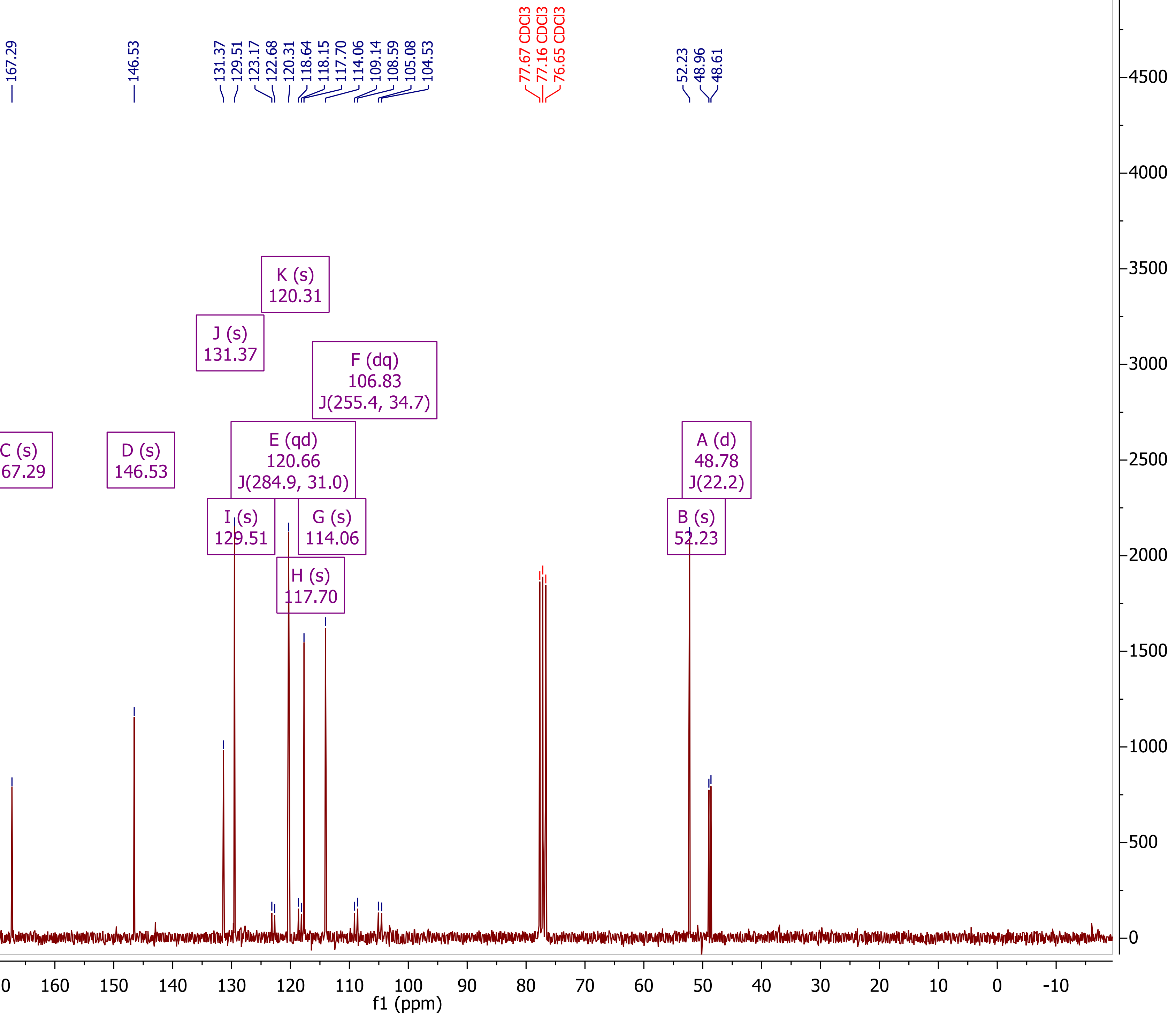

^{1}H NMR (250 MHz, Chloroform-*d*) δ 7.07 (t, *J* = 8.1 Hz, 1H), 6.34 (dd, *J* = 8.0, 2.3 Hz, 2H), 6.24 (s, 1H), 4.19 – 3.48 (m, 3H), 1.02 (s, 9H), 0.23 (s, 6H).

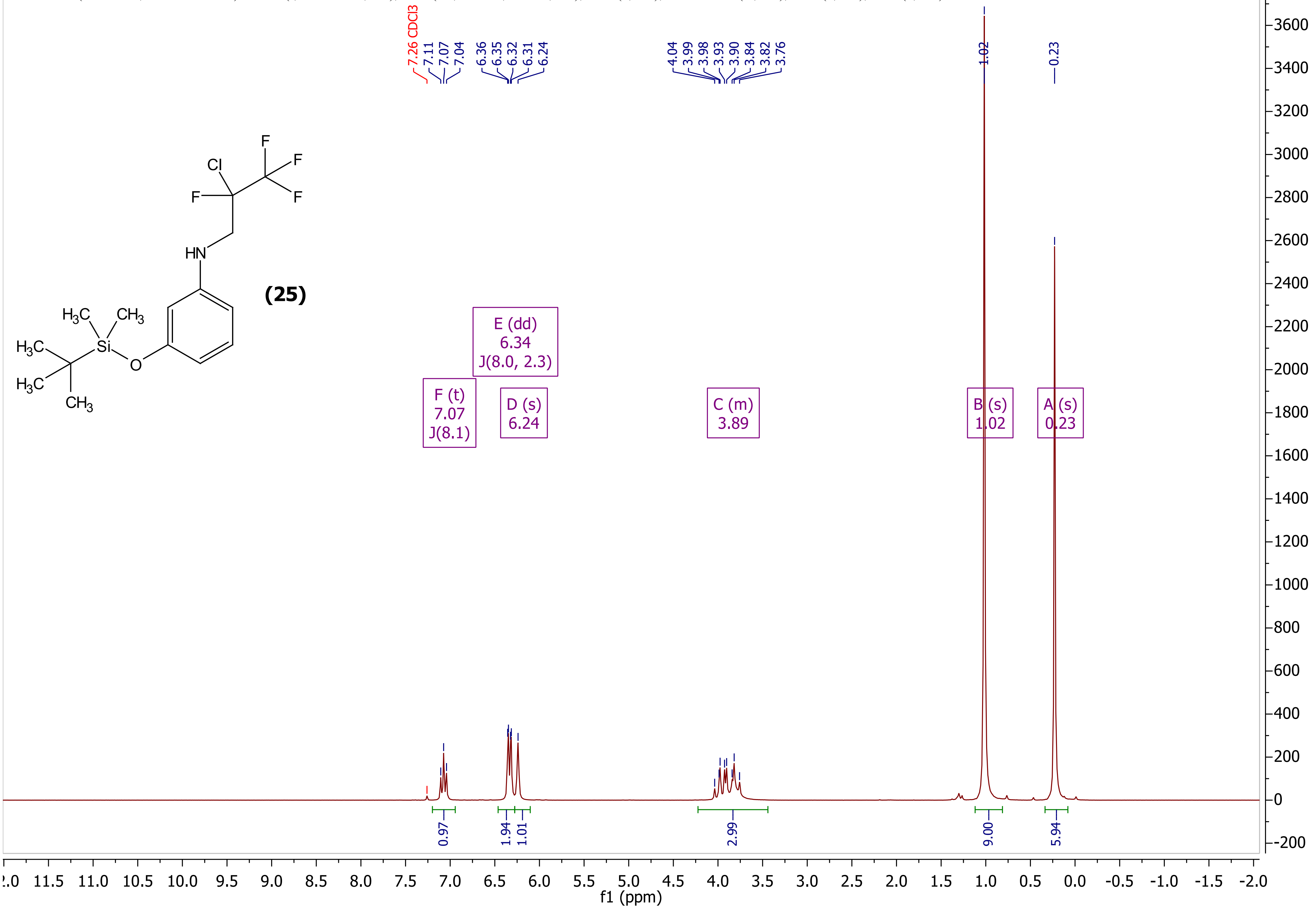

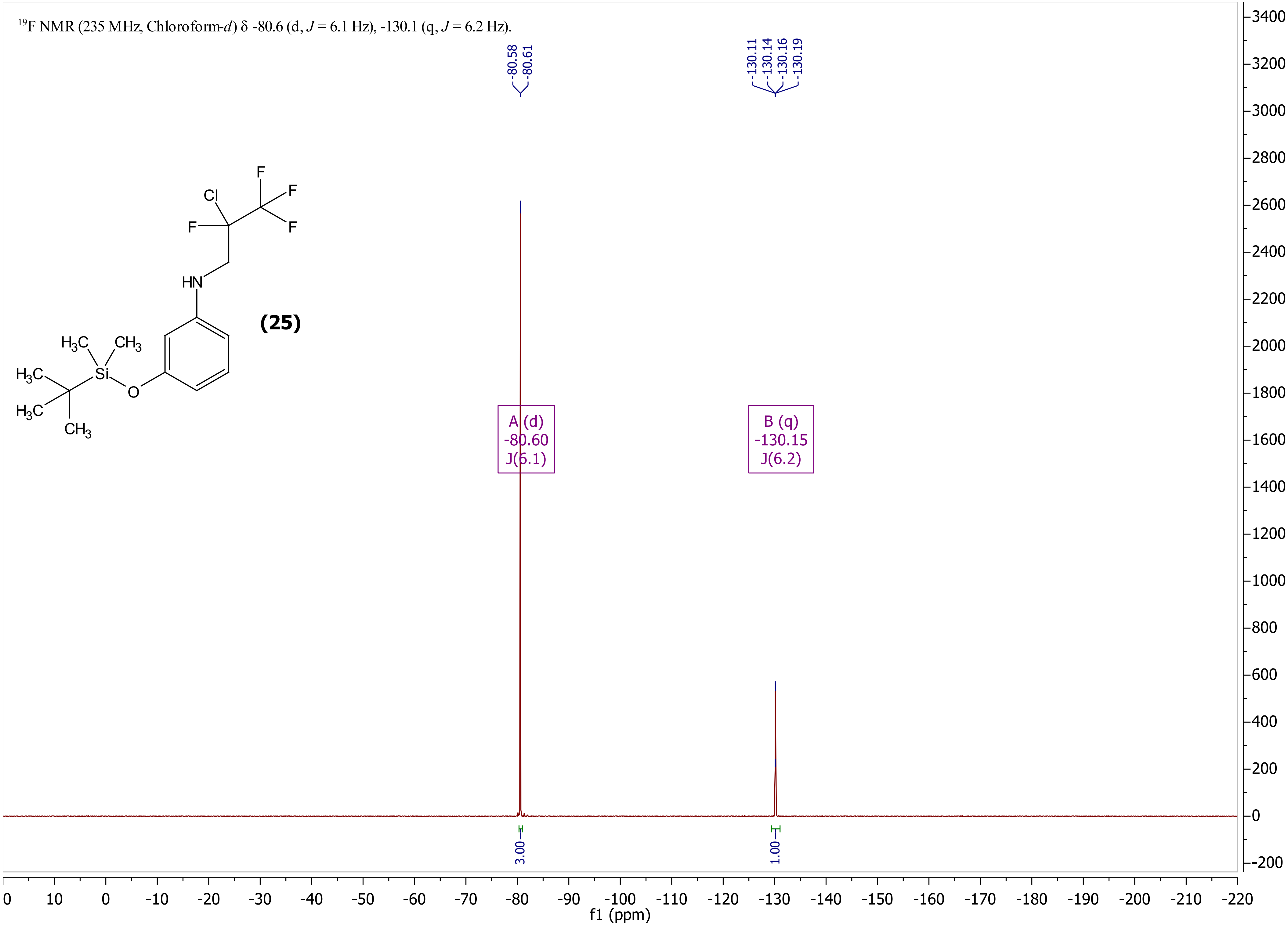

^{19}F NMR (235 MHz, Chloroform-d) δ -80.6 (d, J = 6.1 Hz), -130.1 (q, J = 6.2 Hz).
-80.58
-80.61
-130.11
-130.14
-130.16
-130.19
(25)
A (d)
-80.60
J(6.1)
B (q)
-130.15
J(6.2)
3.00
1.00
f1 (ppm)

^{13}C NMR (63 MHz, Chloroform-*d*) δ 156.9, 147.7, 130.0, 120.6 (qd, *J* = 285.0, 31.3 Hz), 111.0, 106.8 (dq, *J* = 255.1, 34.6 Hz), 106.7, 105.3, 49.0 (d, *J* = 22.3 Hz), 25.7, 18.2, -4.4.

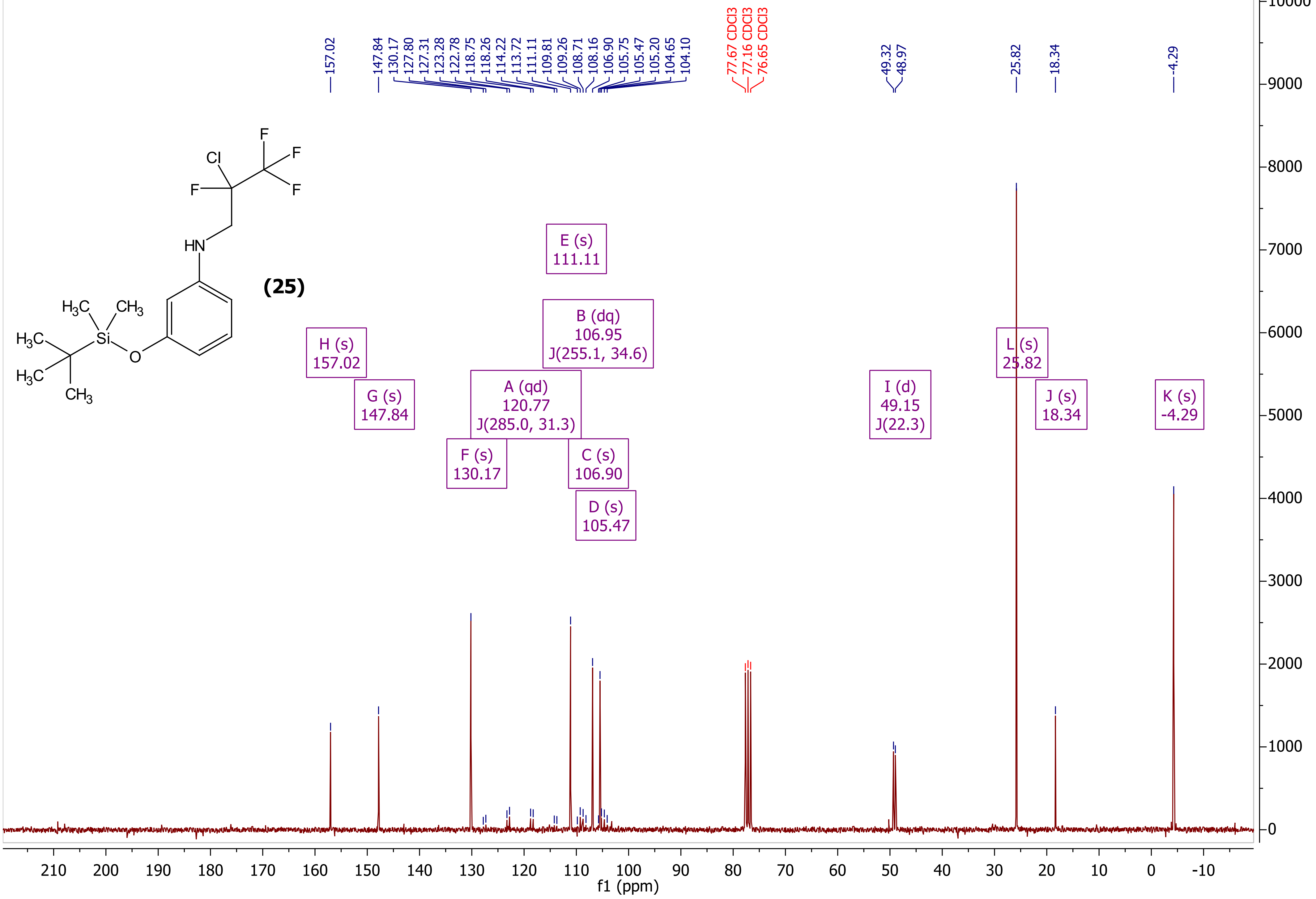

^{1}H NMR (250 MHz, Chloroform-*d*) δ 7.07 (t, *J* = 8.0 Hz, 1H), 6.30 (d, *J* = 8.0 Hz, 2H), 6.21 (s, 1H), 4.68 (bs, 2H), 3.95 (dd, *J* = 15.3, 12.5 Hz, 1H), 3.79 (dd, *J* = 21.3, 15.3 Hz, 1H).

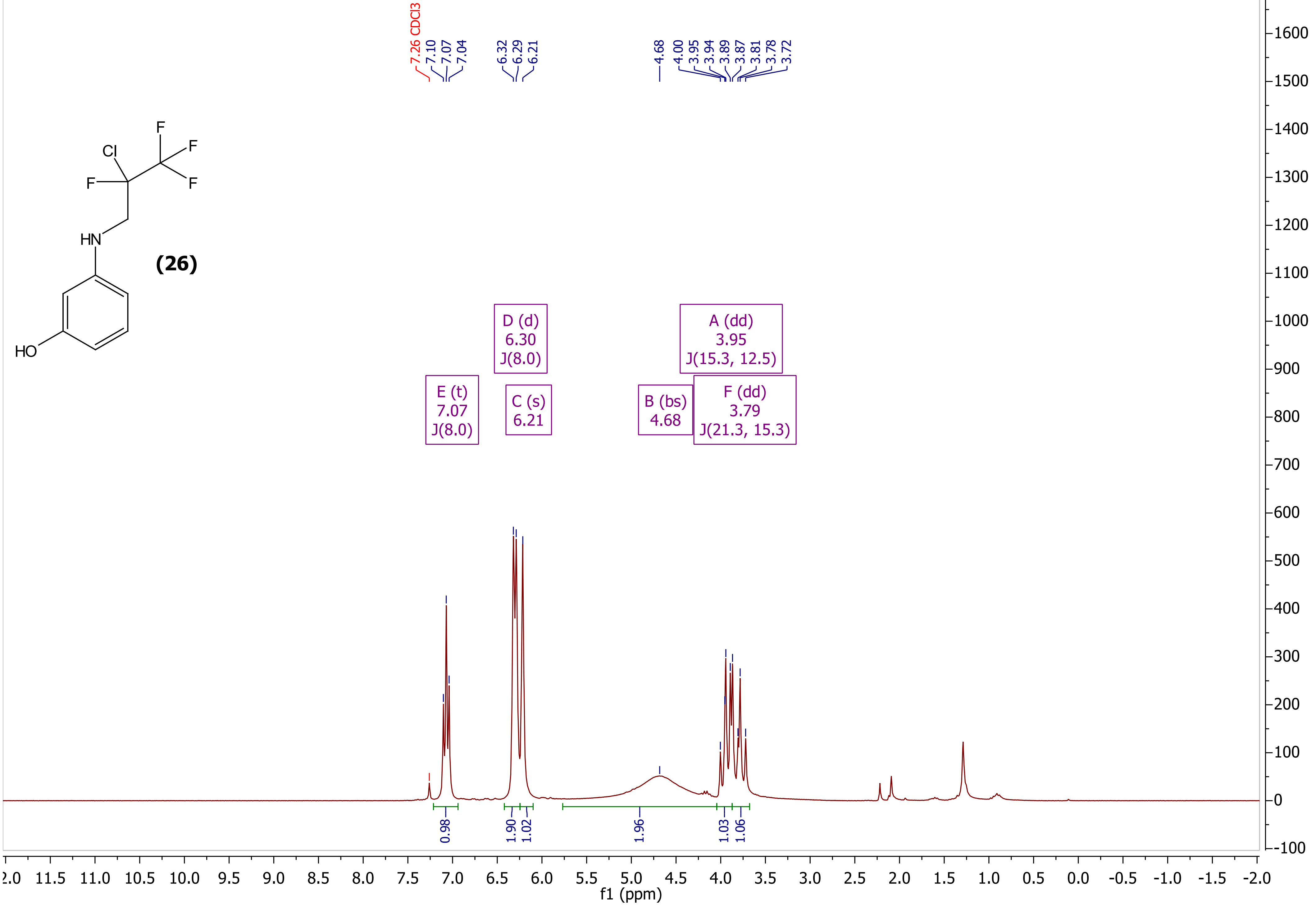

^{19}F NMR (235 MHz, Chloroform-*d*) δ -80.6 (d, *J* = 6.1 Hz), -130.2 (q, *J* = 6.1 Hz).

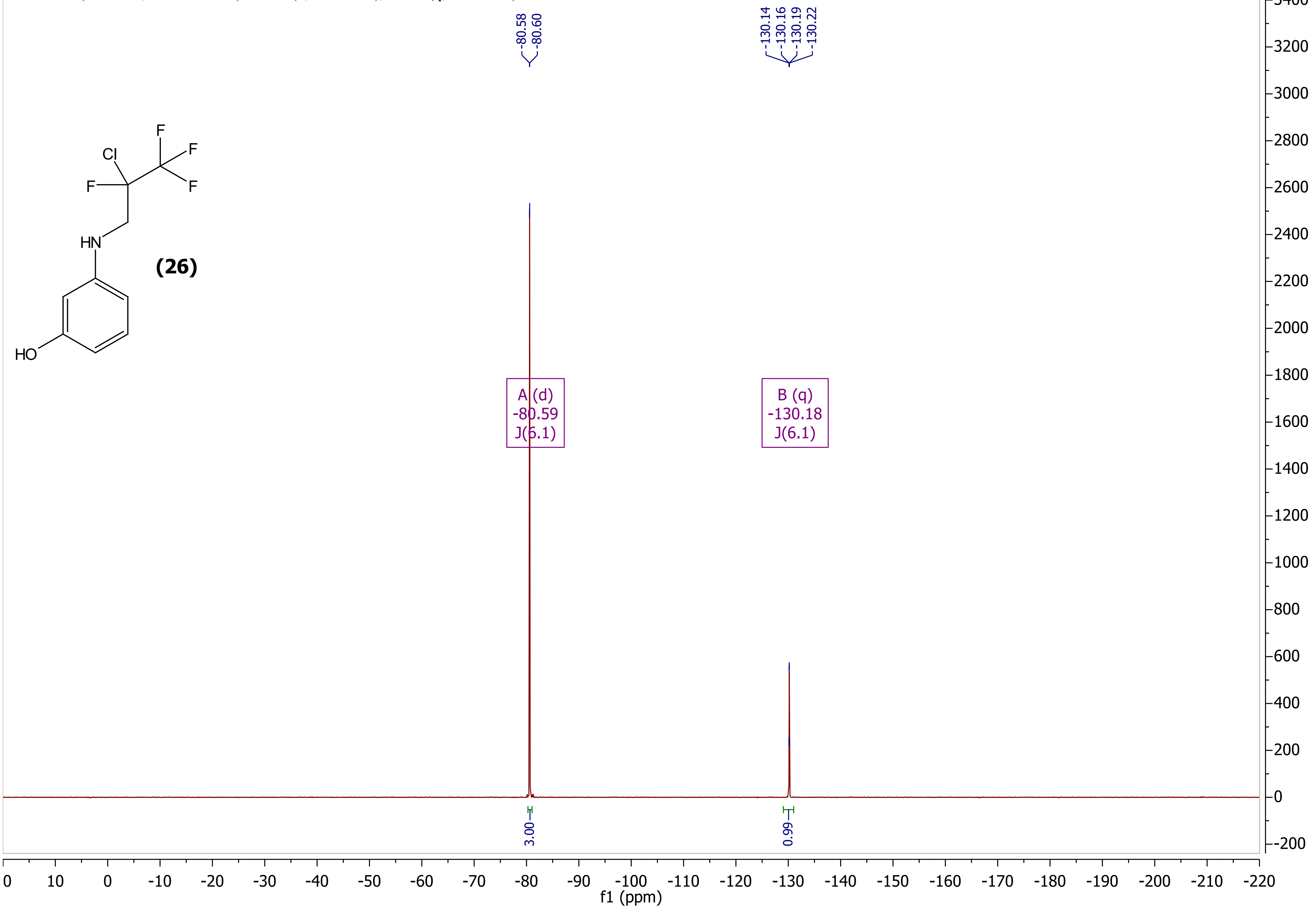

^{13}C NMR (63 MHz, Chloroform-*d*) δ 156.7, 148.1, 130.6, 120.7 (qd, *J* = 284.8, 31.1 Hz), 106.8 (dq, *J* = 255.6, 34.9 Hz), 106.3, 100.6, 48.9 (d, *J* = 22.2 Hz).

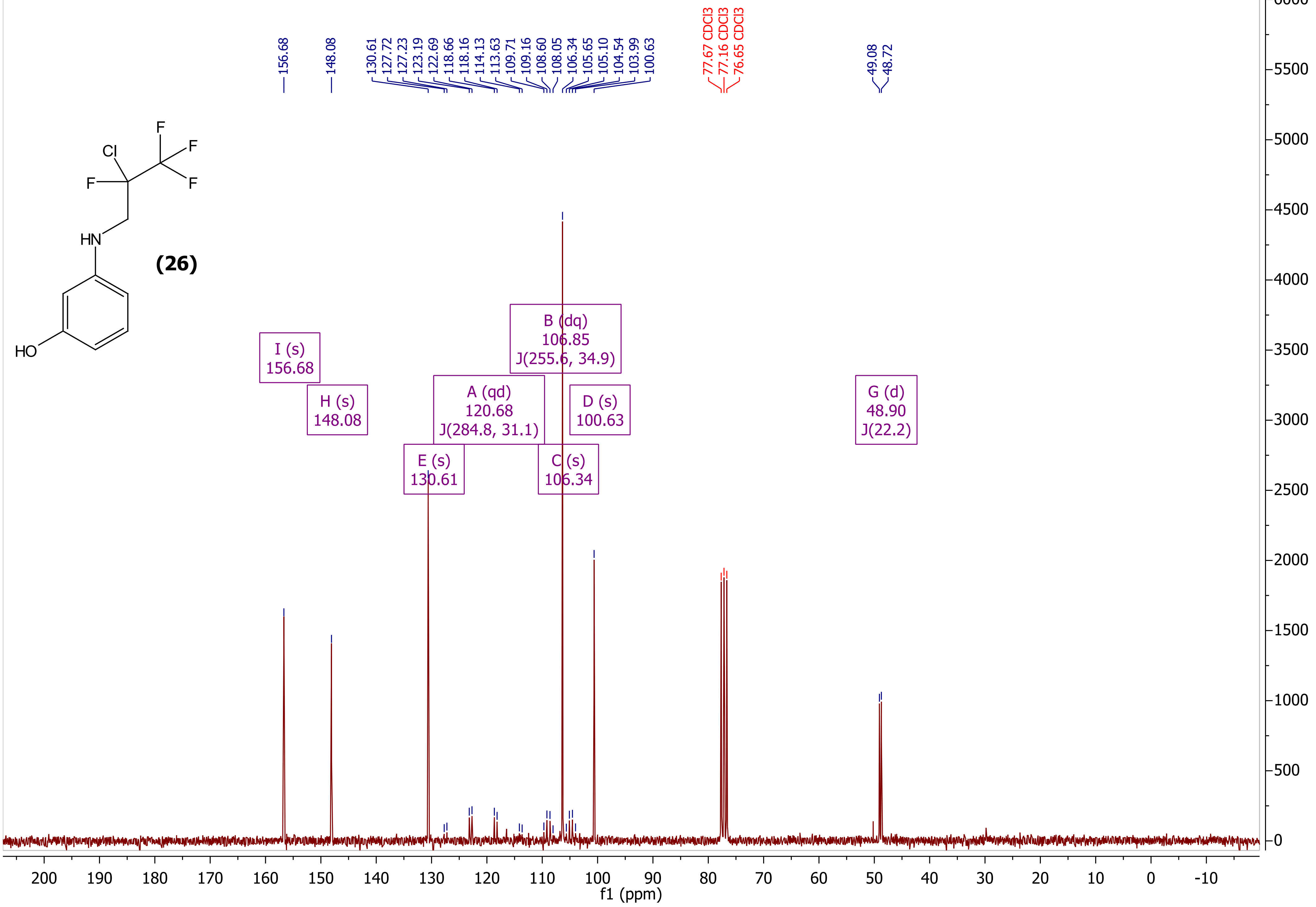

^{1}H NMR (250 MHz, Chloroform-*d*) δ 7.07 (d, *J* = 8.1 Hz, 2H), 6.67 (d, *J* = 8.3 Hz, 2H), 4.11 – 3.67 (m, 3H), 2.30 (s, 3H).

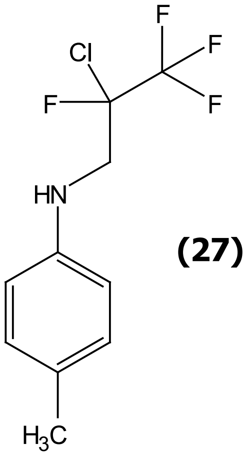

(27)

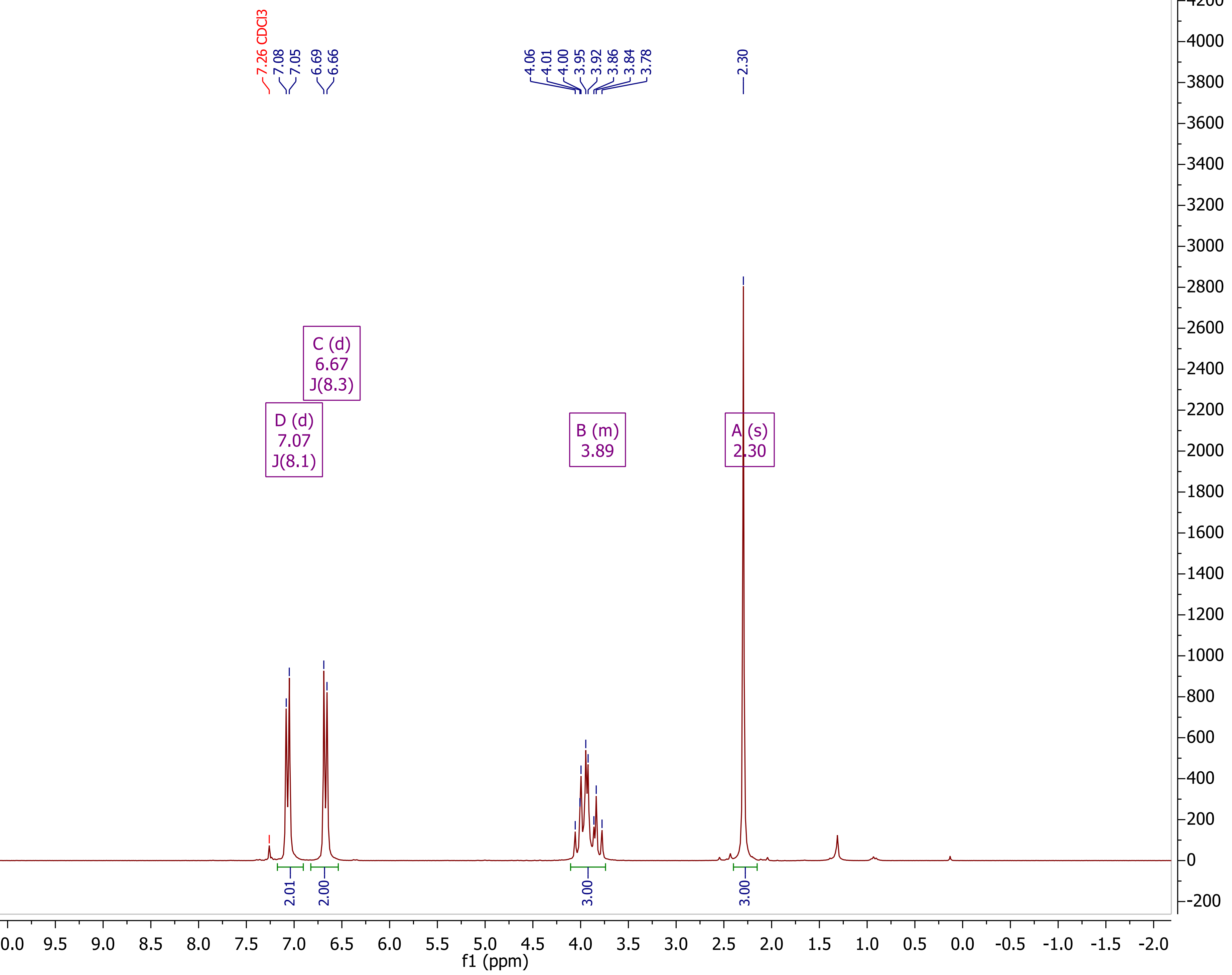

^{19}F NMR (235 MHz, Chloroform-*d*) δ -80.6 (d, *J* = 6.2 Hz), -130.1 (q, *J* = 6.1 Hz).

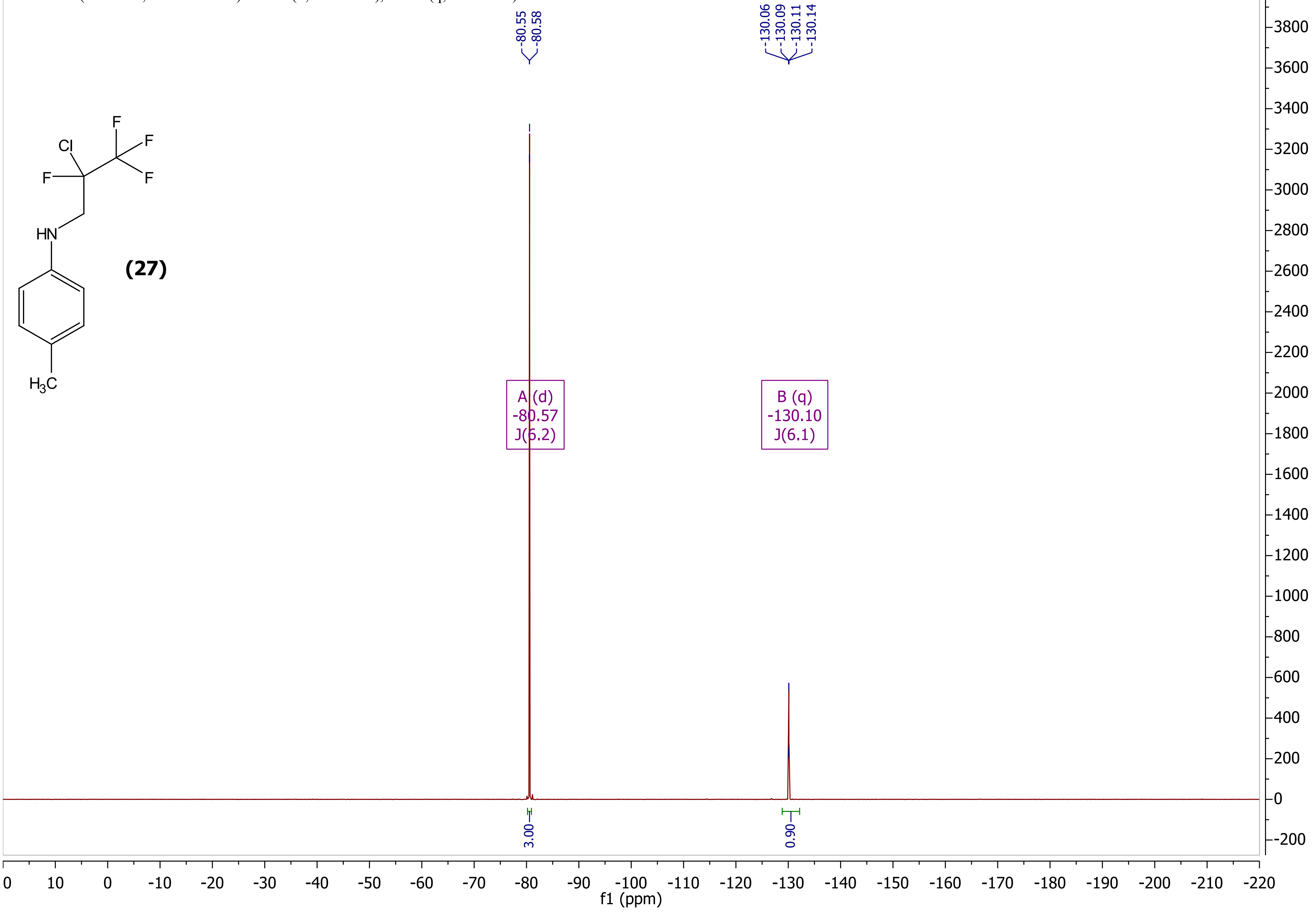

^{13}C NMR (63 MHz, Chloroform-*d*) δ 144.0, 130.0, 128.7, 120.8 (qd, *J* = 284.8, 31.0 Hz), 113.7, 107.0 (dq, *J* = 255.4, 34.8 Hz), 49.5 (d, *J* = 22.1 Hz), 20.5.

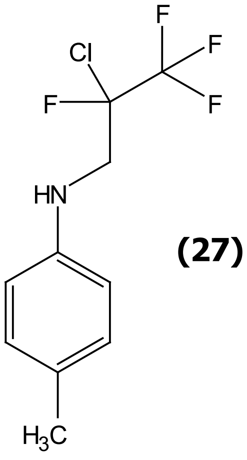

(27)

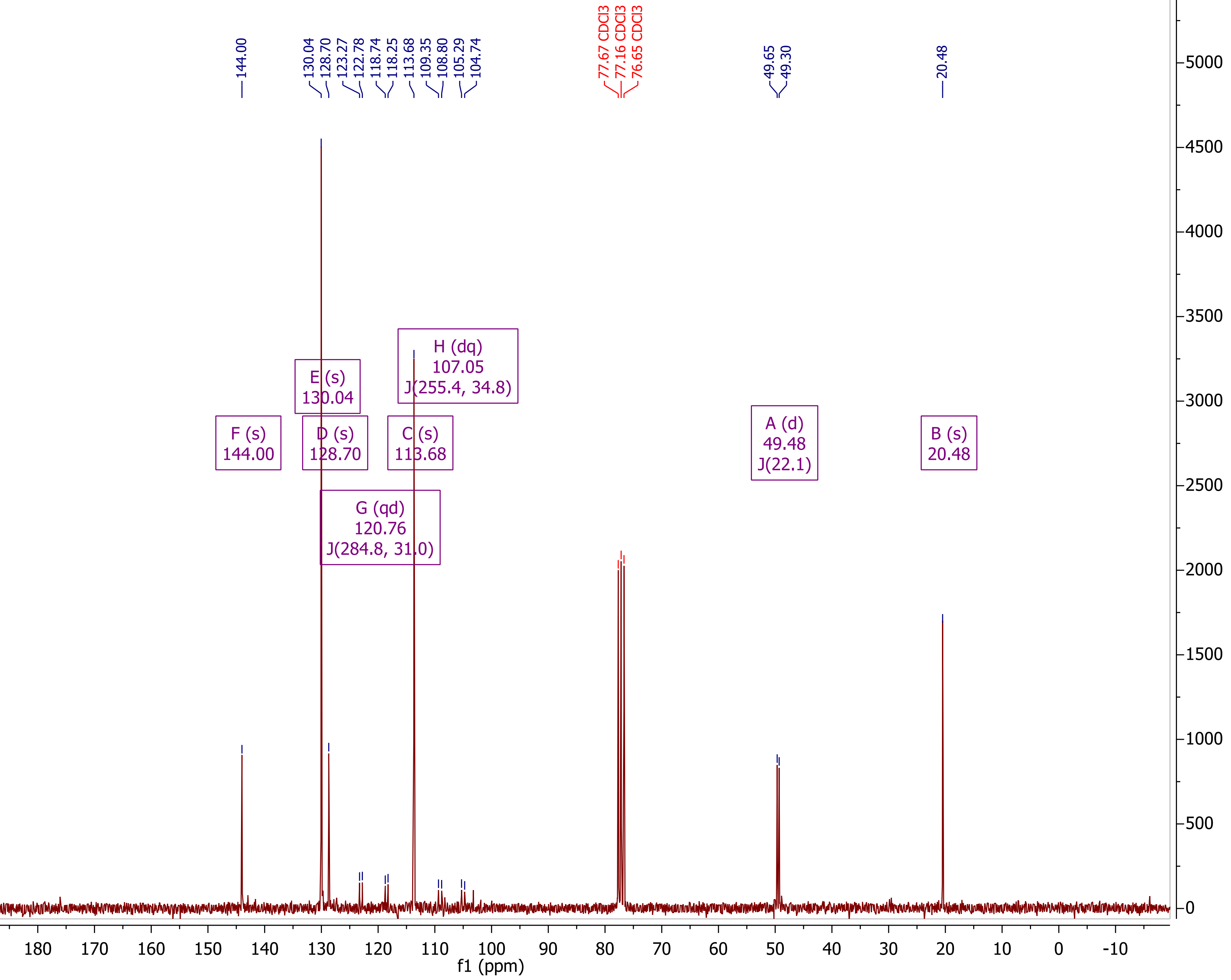

^{1}H NMR (250 MHz, Chloroform-*d*) δ 7.02 – 6.79 (m, 2H), 6.78 – 6.49 (m, 2H), 3.97 (dd, *J* = 15.3, 12.3 Hz, 1H), 3.85 (s, 1H), 3.81 (dd, *J* = 21.2, 15.2 Hz, 1H).

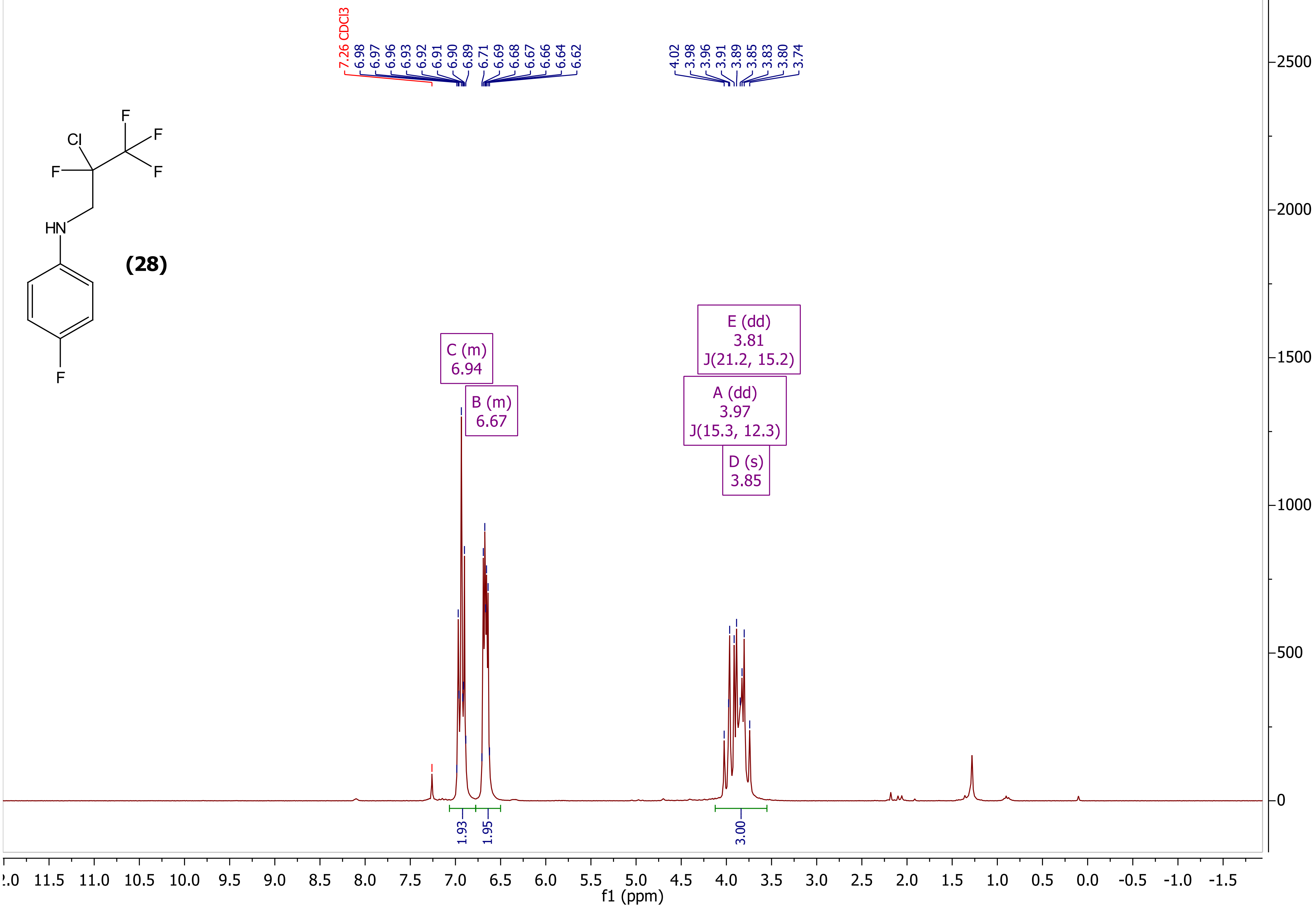

^{19}F NMR (235 MHz, Chloroform-*d*) δ -80.6 (d, *J* = 6.1 Hz), -125.9, -130.2 (q, *J* = 6.1 Hz).

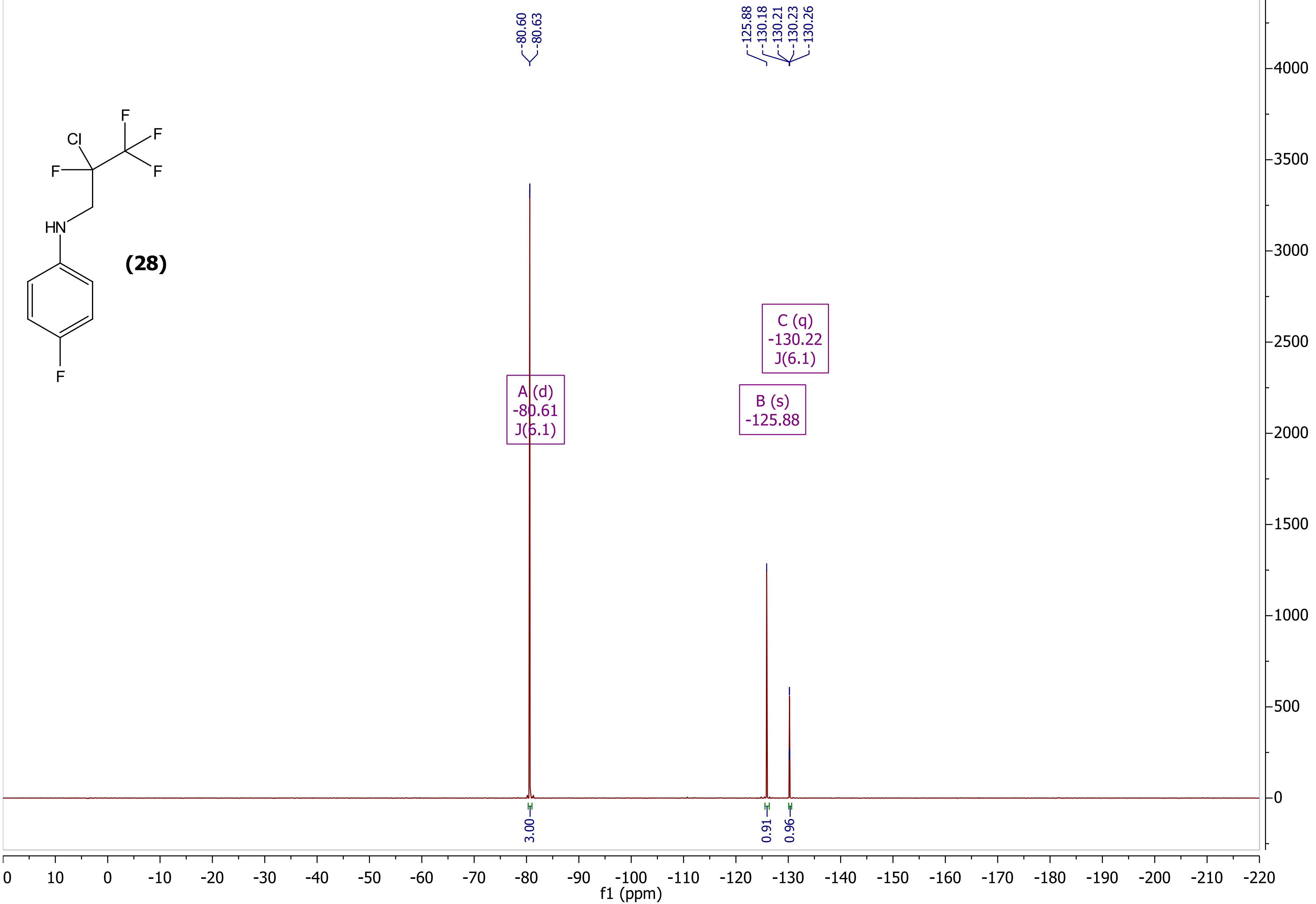

^{13}C NMR (63 MHz, Chloroform-*d*) δ 156.8 (d, *J* = 237.1 Hz), 142.7 (d, *J* = 2.1 Hz), 120.7 (qd, *J* = 284.8, 31.0 Hz), 116.0 (d, *J* = 22.6 Hz), 114.6 (d, *J* = 7.6 Hz), 107.0 (dq, *J* = 255.0, 34.9 Hz), 49.8 (d, *J* = 22.1 Hz).

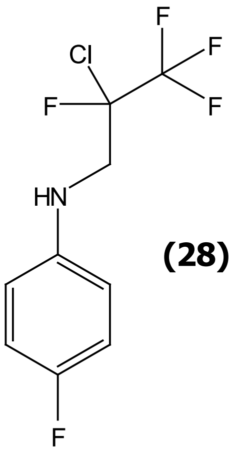

(28)

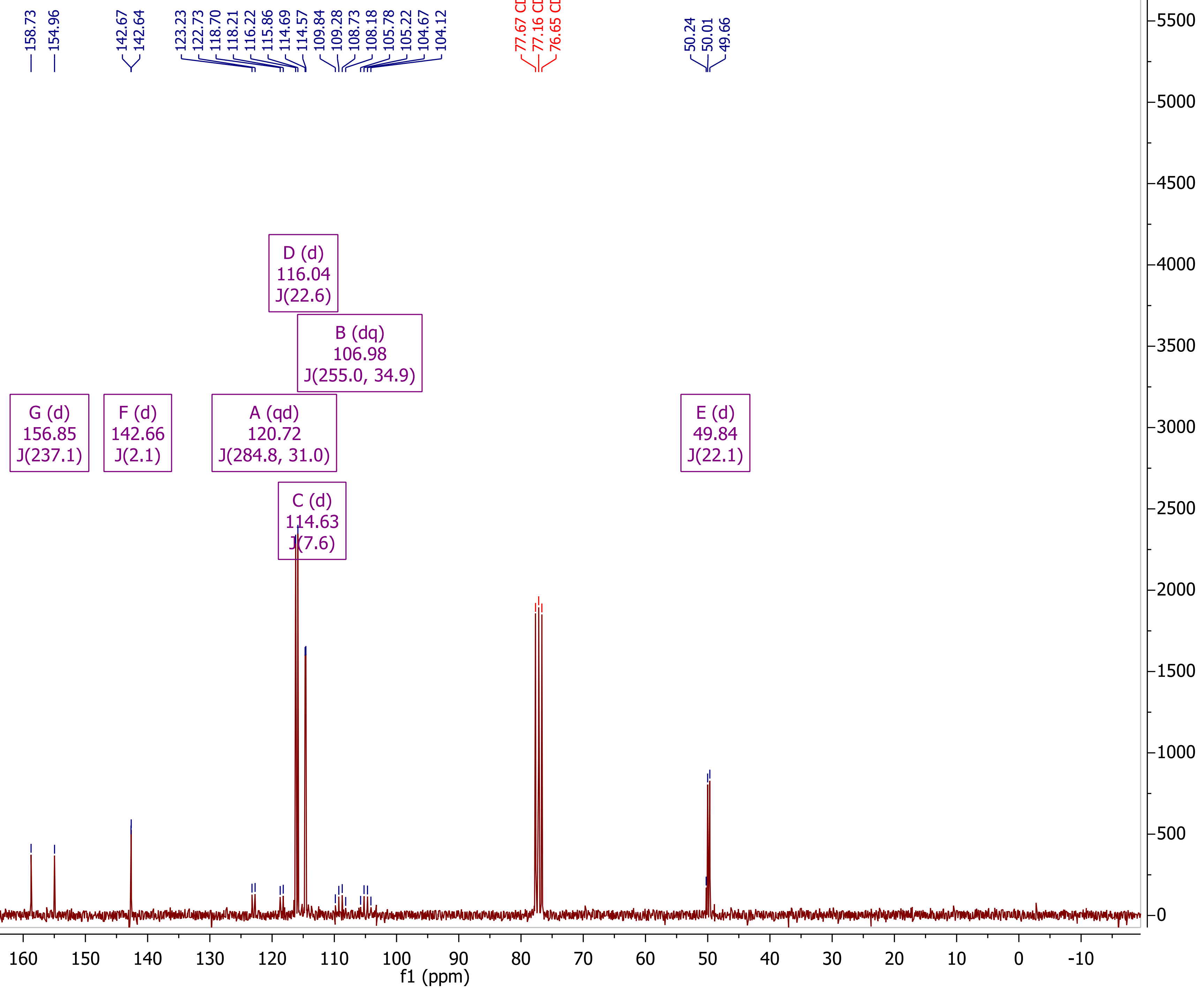

^{1}H NMR (250 MHz, Chloroform-*d*) δ 7.17 (d, *J* = 8.8 Hz, 2H), 6.64 (d, *J* = 8.8 Hz, 2H), 3.98 (dd, *J* = 15.4, 12.4 Hz, 1H), 3.88 (s, 1H), 3.83 (dd, *J* = 21.1, 15.3 Hz, 1H).

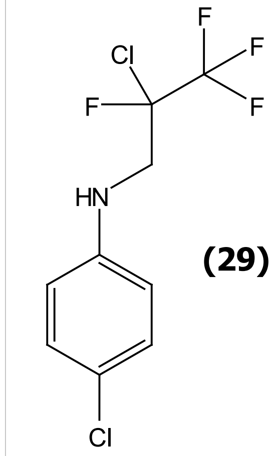

(29)

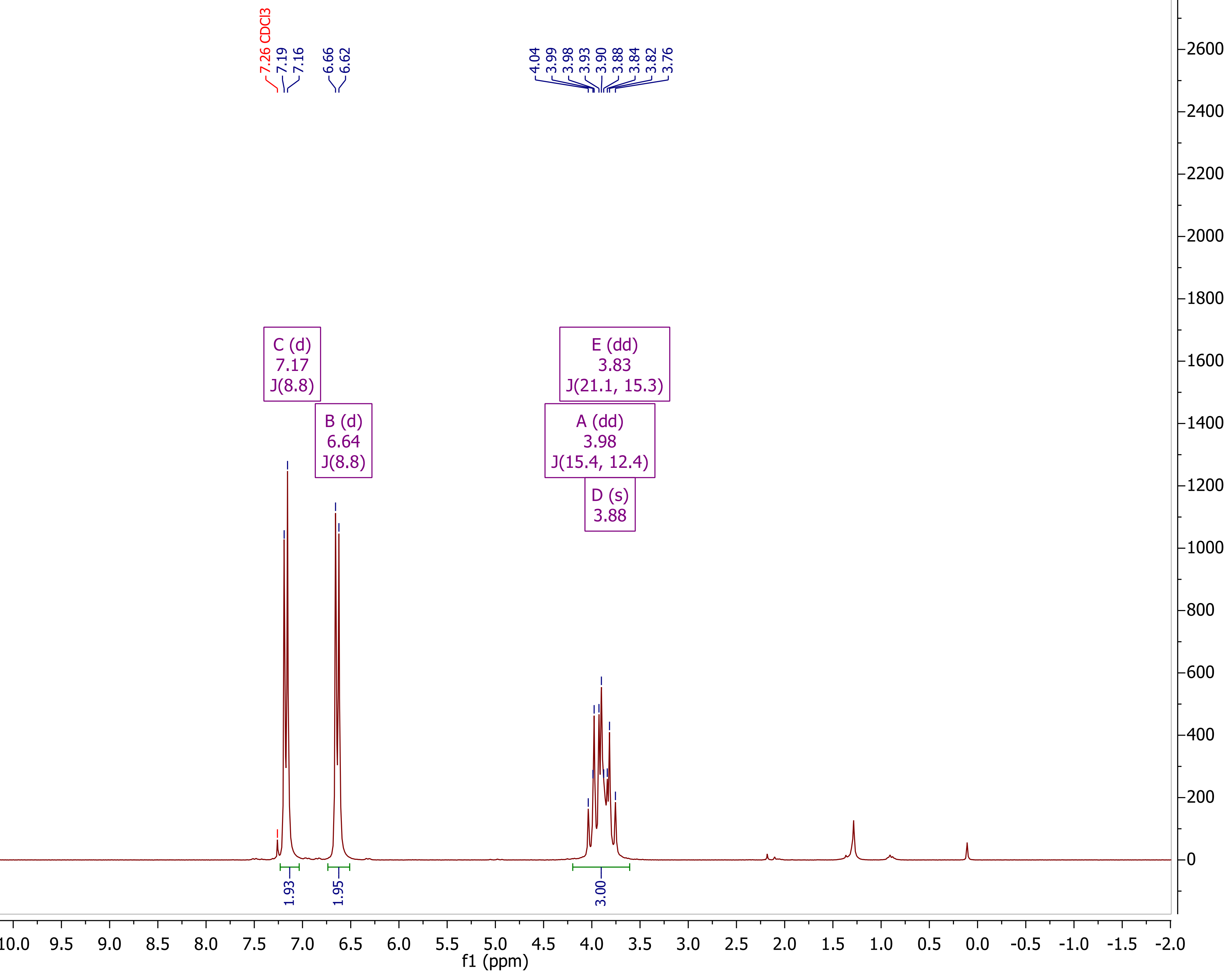

^{19}F NMR (235 MHz, Chloroform-*d*) δ -80.6 (d, *J* = 6.2 Hz), -130.2 (q, *J* = 6.1 Hz).

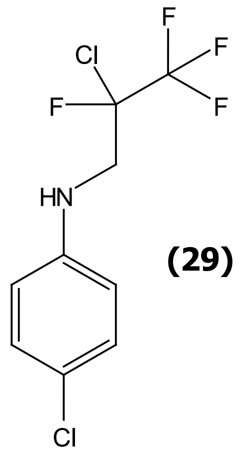

(29)

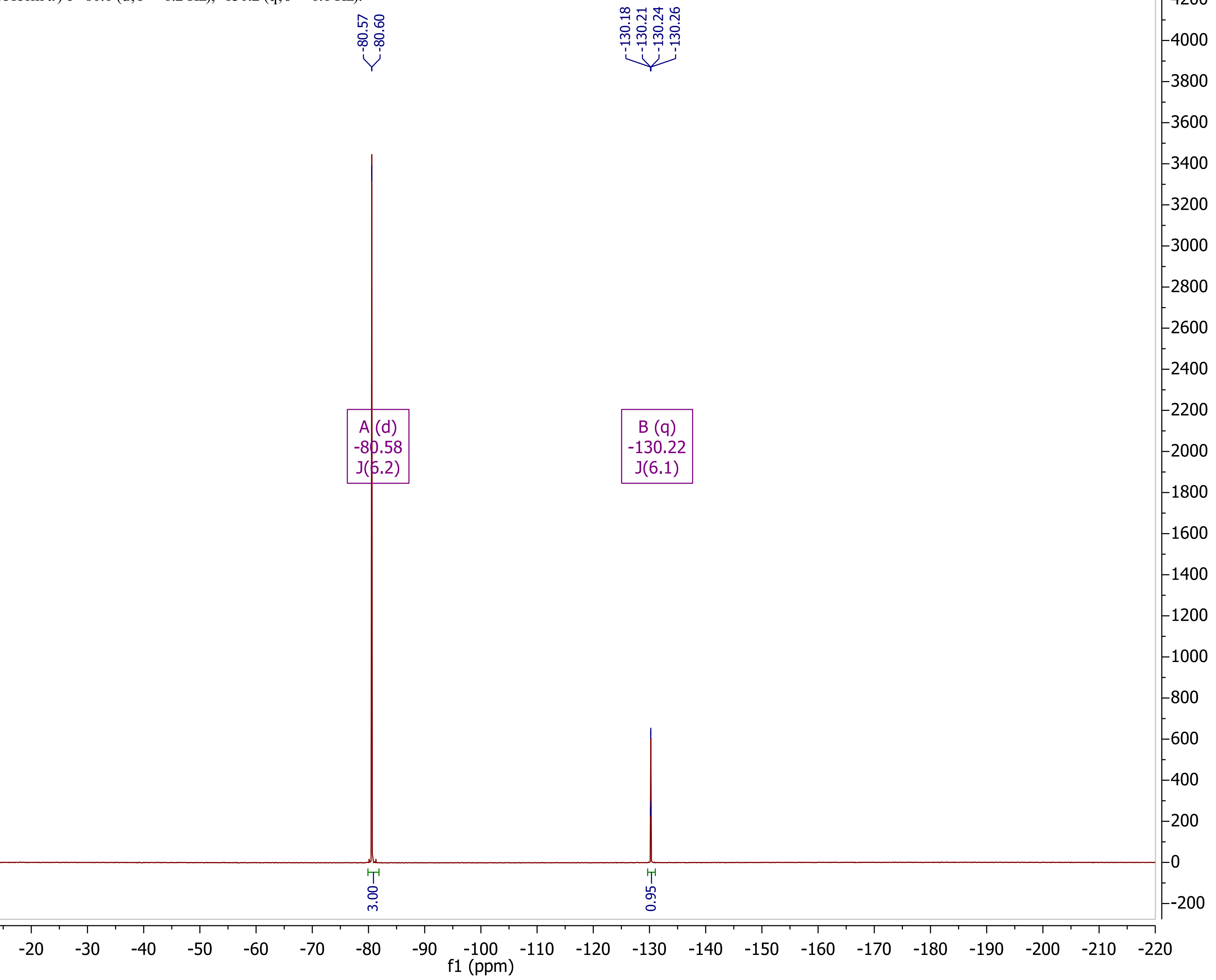

^{13}C NMR (63 MHz, Chloroform-*d*) δ 145.0, 129.4, 123.9, 120.7 (qd, *J* = 285.0, 31.0 Hz), 114.5, 106.9 (dq, *J* = 255.4, 34.7 Hz), 49.1 (d, *J* = 22.1 Hz).

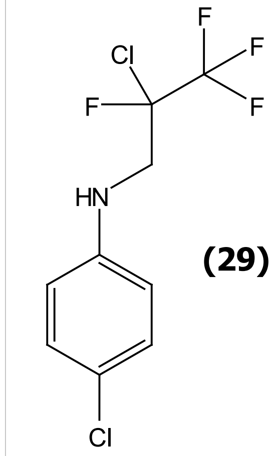

(29)

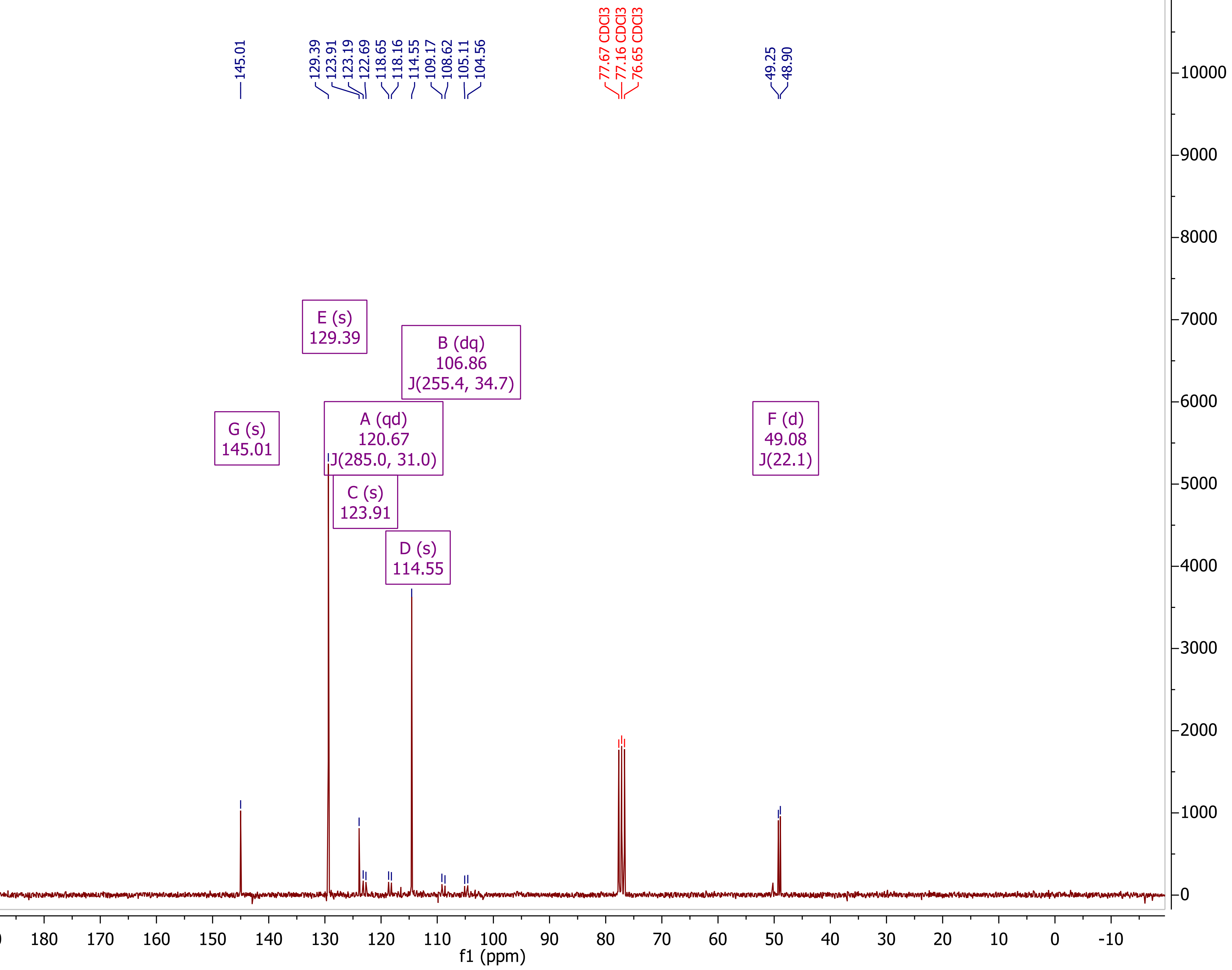

^{1}H NMR (250 MHz, Chloroform-*d*) δ 7.20 (d, *J* = 8.7 Hz, 2H), 6.49 (d, *J* = 8.7 Hz, 2H), 4.05 – 3.56 (m, 3H).

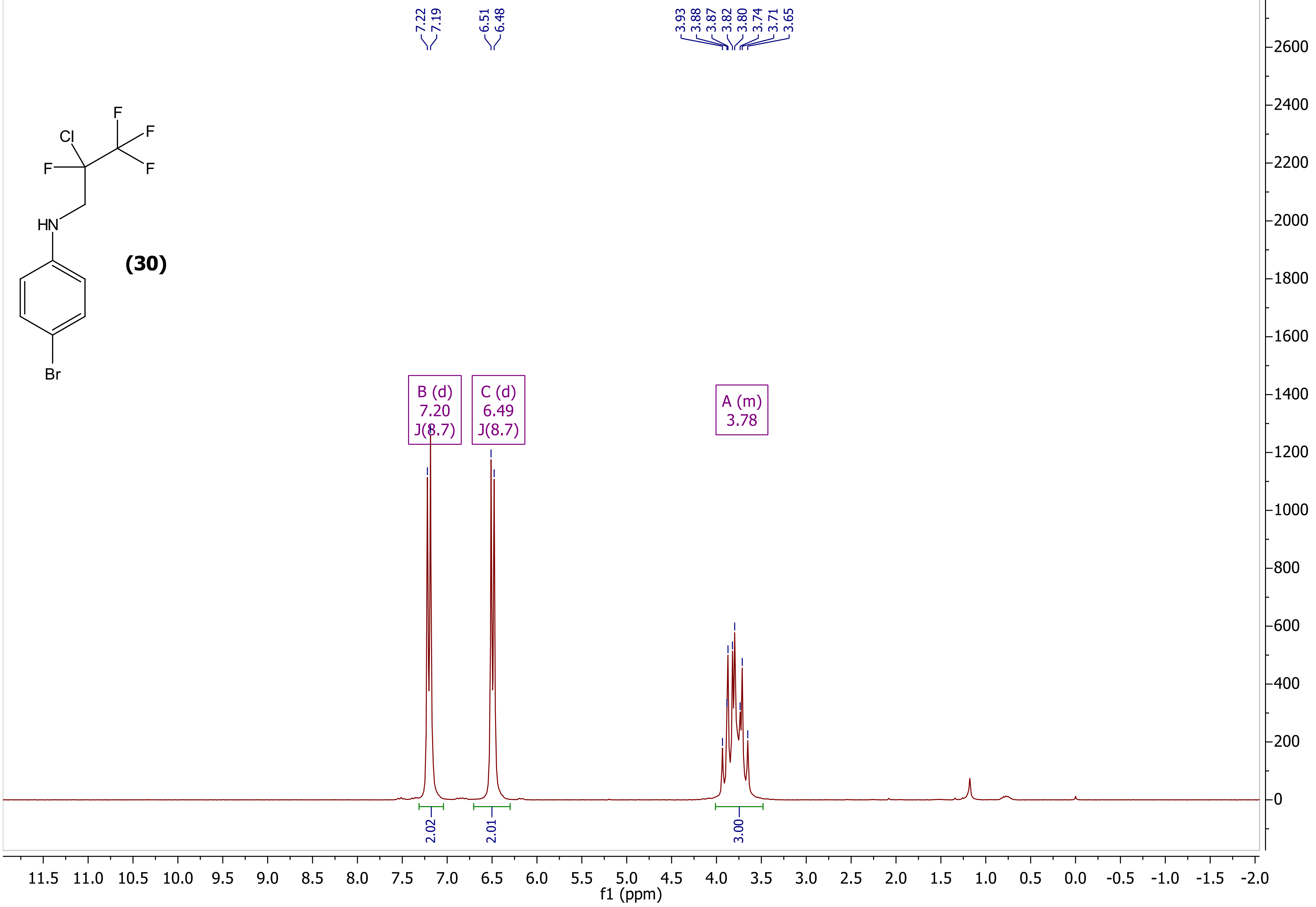

(30)

^{19}F NMR (235 MHz, Chloroform-*d*) δ -80.6 (d, *J* = 6.2 Hz), -130.2 (q, *J* = 6.2 Hz).

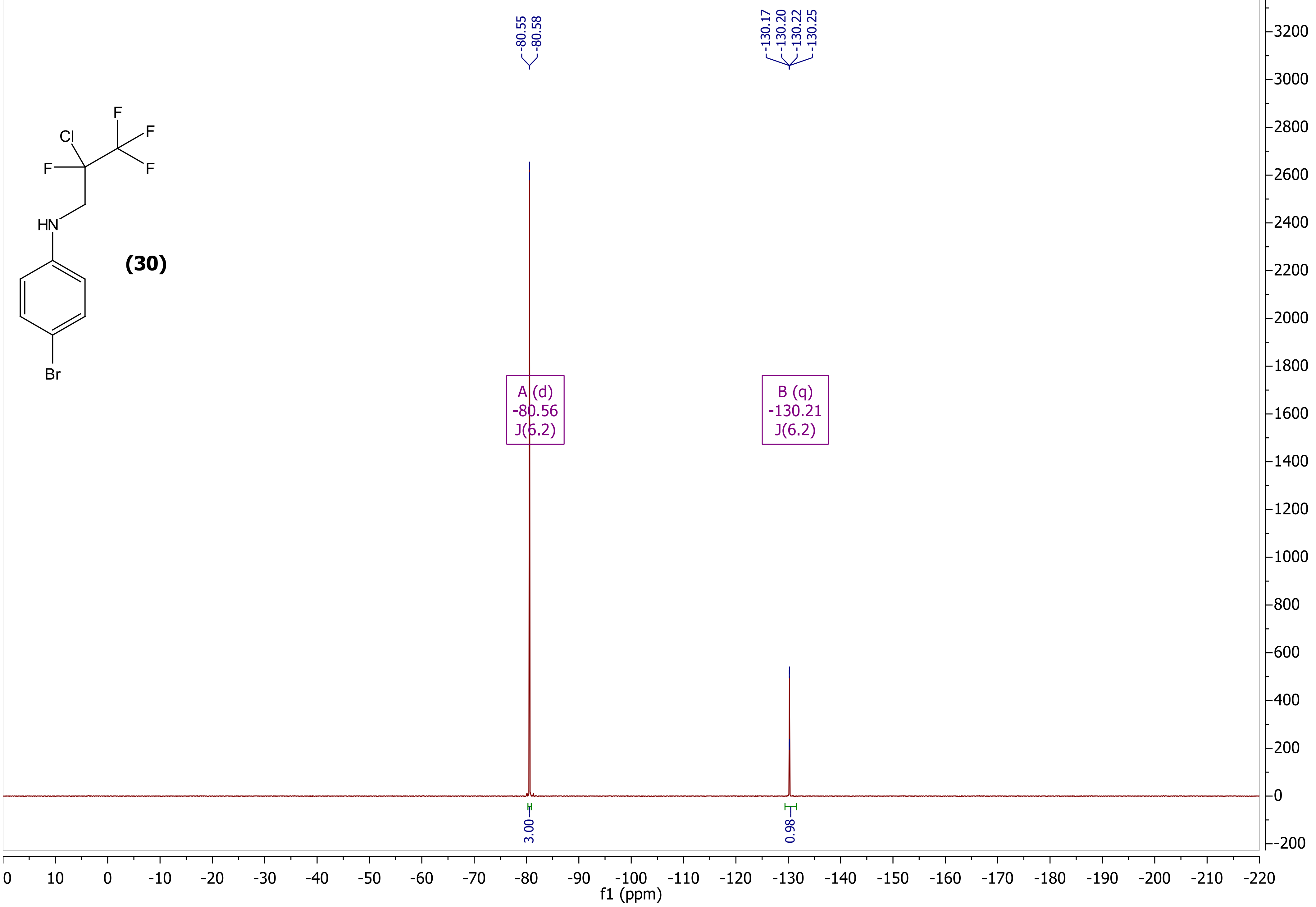

^{13}C NMR (63 MHz, Chloroform-*d*) δ 145.4, 132.3, 120.7 (qd, *J* = 284.9, 31.1 Hz), 115.0 (d, *J* = 1.1 Hz), 111.0, 106.8 (dq, *J* = 255.4, 34.8 Hz), 48.9 (d, *J* = 22.2 Hz).

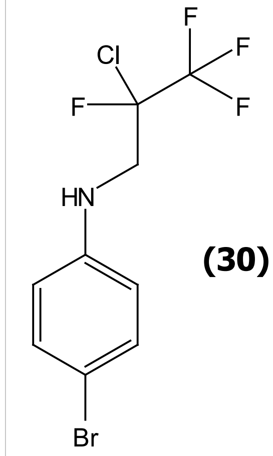

(30)

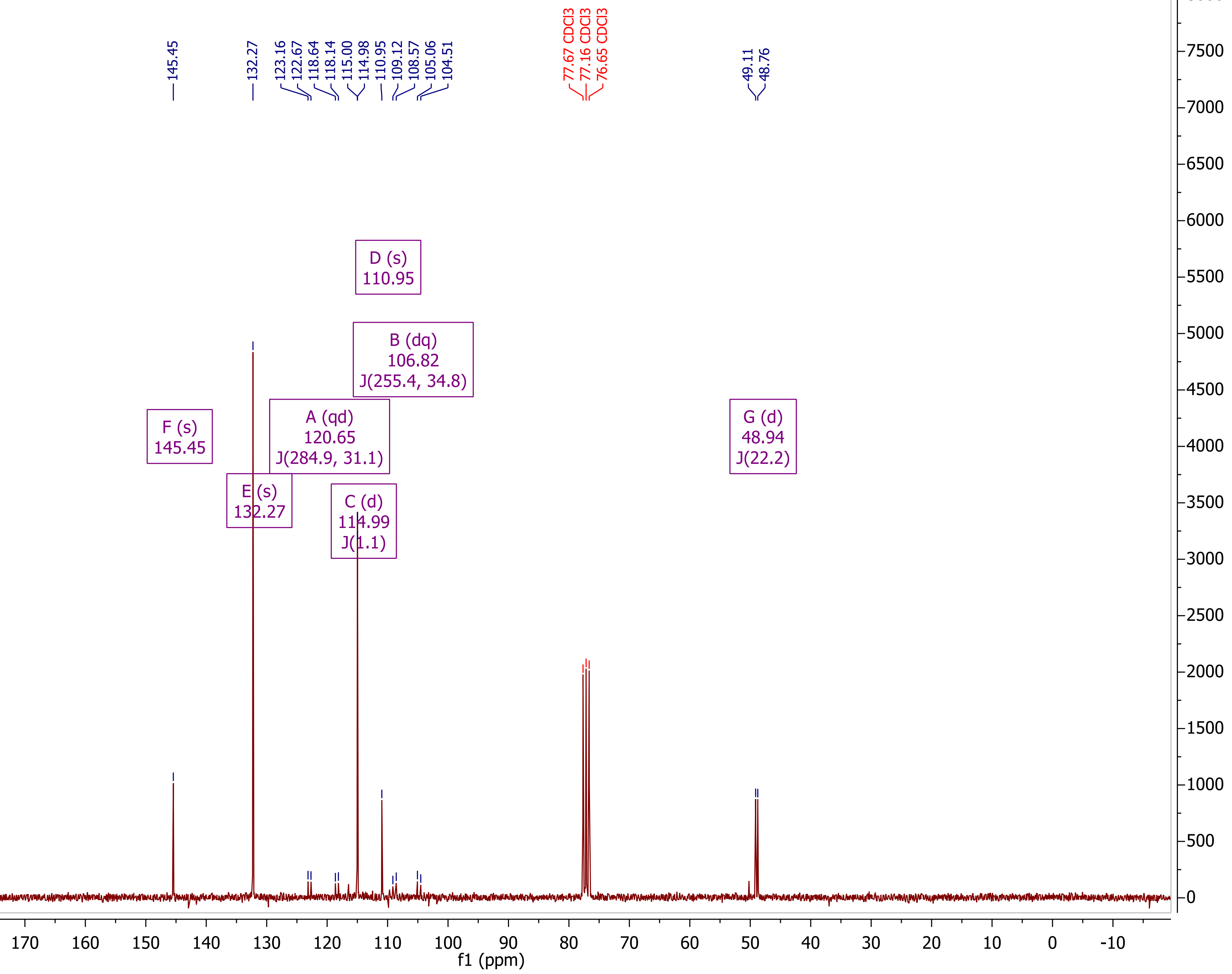

^{1}H NMR (250 MHz, Chloroform-*d*) δ 7.48 (d, *J* = 8.6 Hz, 2H), 6.50 (d, *J* = 8.7 Hz, 2H), 4.25 – 3.46 (m, 3H).

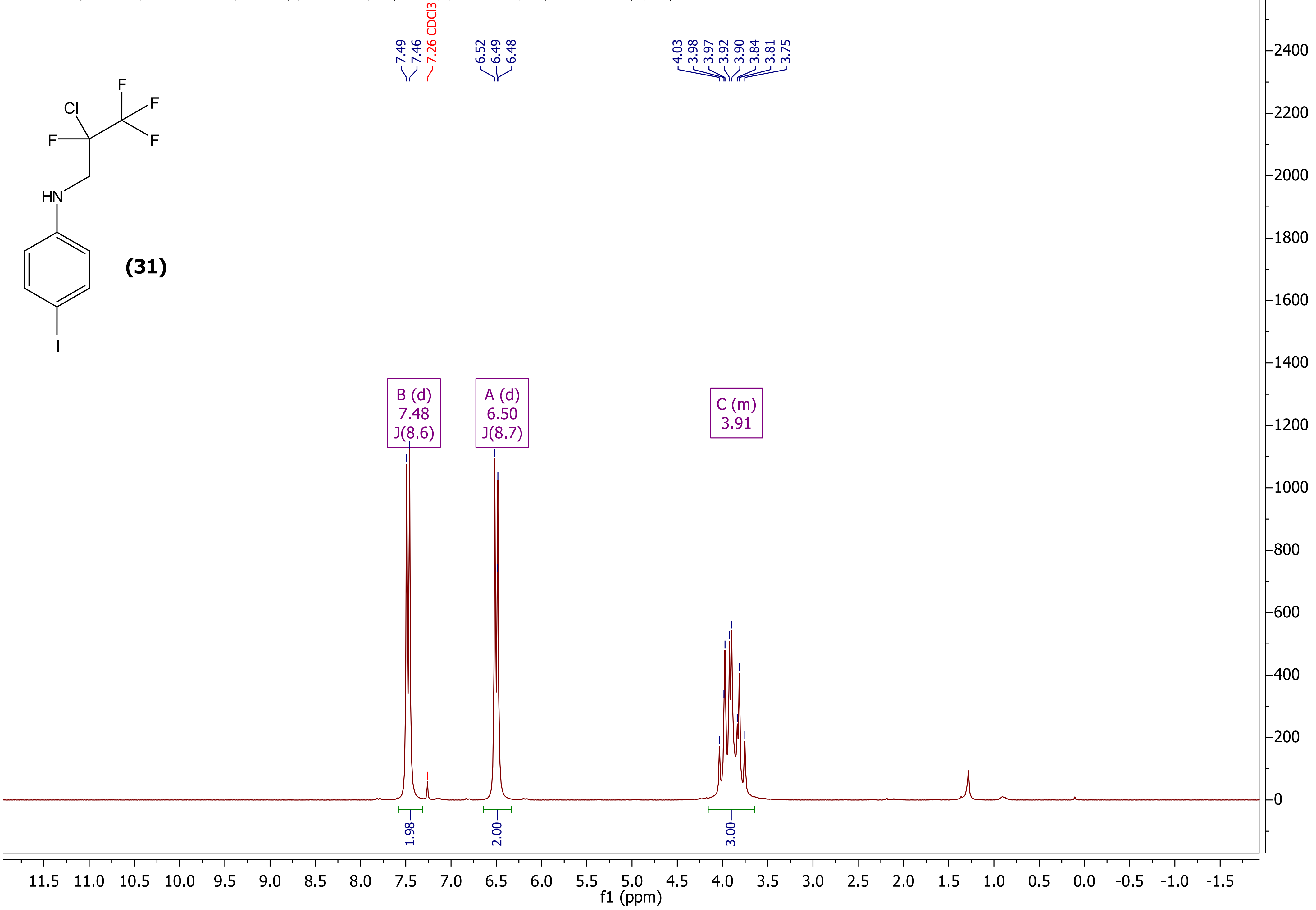

^{19}F NMR (235 MHz, Chloroform-*d*) δ -80.5 (d, *J* = 6.1 Hz), -130.2 (q, *J* = 6.1 Hz).

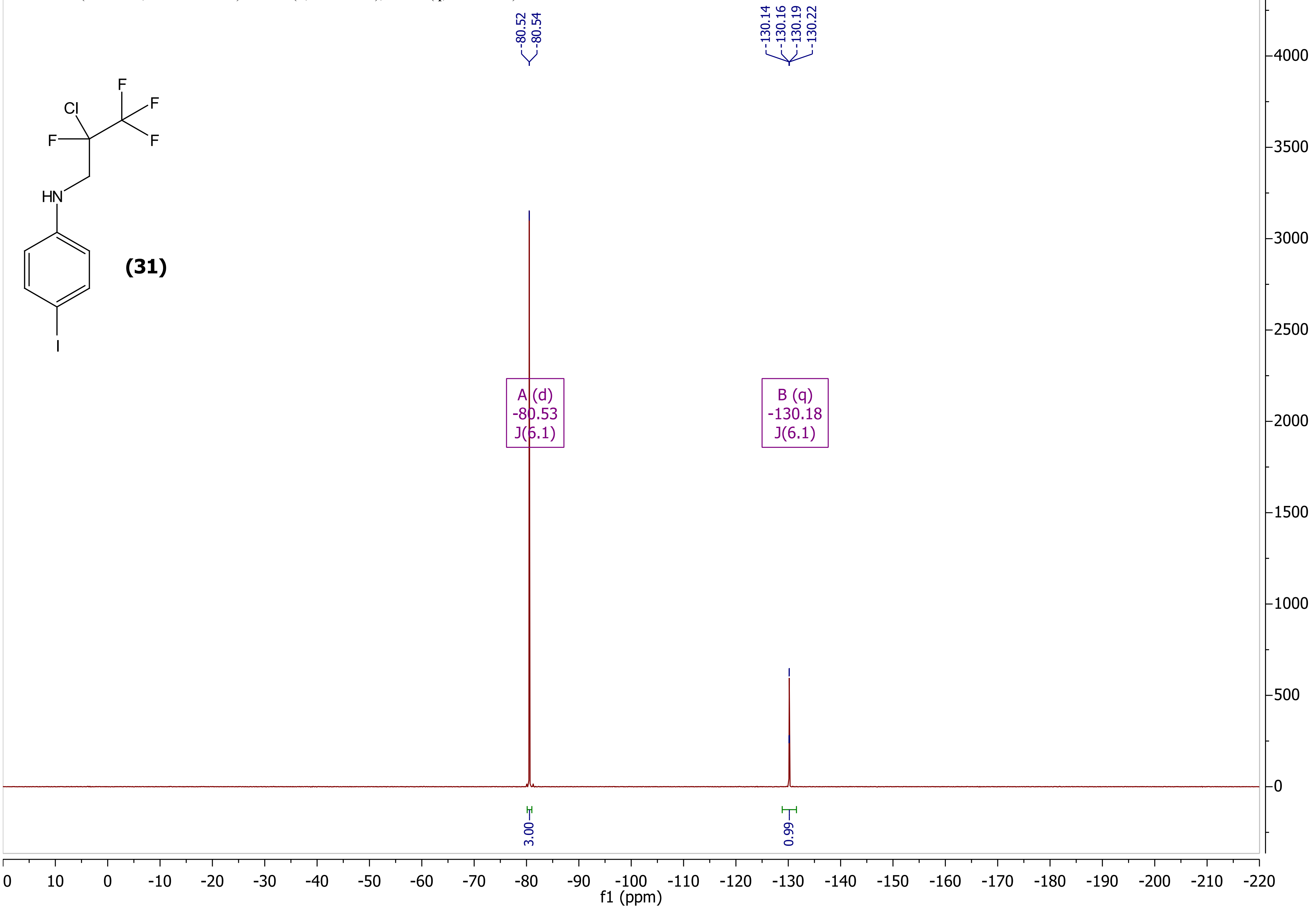

^{13}C NMR (63 MHz, Chloroform-*d*) δ 146.1, 138.1, 120.6 (qd, *J* = 285.1, 31.2 Hz), 115.5 (d, *J* = 1.1 Hz), 106.8 (dq, *J* = 255.4, 34.7 Hz), 80.2, 48.7 (d, *J* = 22.2 Hz).

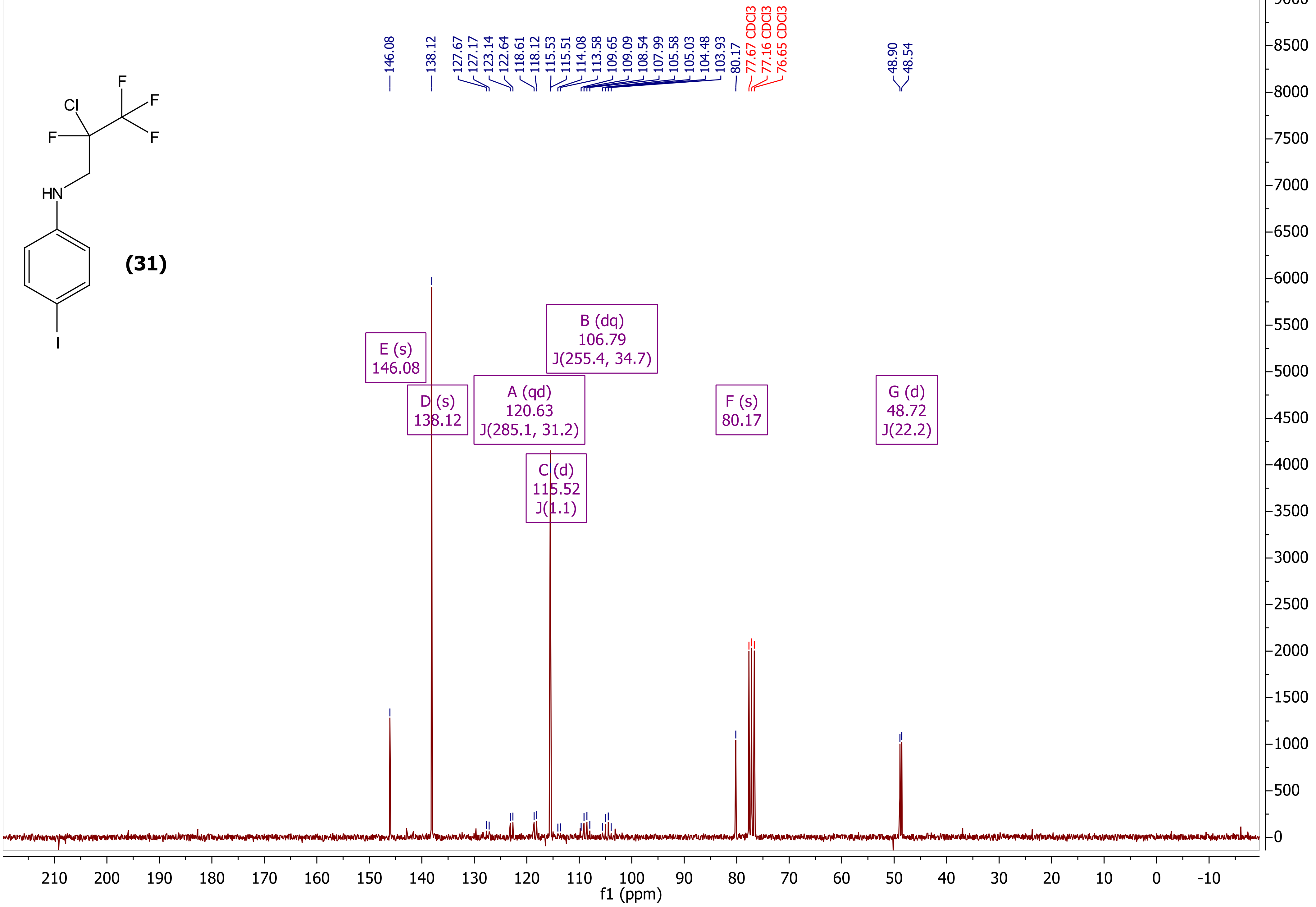

(31)

^{1}H NMR (250 MHz, Chloroform-*d*) δ 7.84 (d, *J* = 8.8 Hz, 2H), 6.71 (d, *J* = 8.8 Hz, 2H), 4.68 (bs, 1H), 4.07 (dd, *J* = 15.6, 12.6 Hz, 1H), 3.92 (dd, *J* = 20.9, 15.6 Hz, 1H), 2.50 (s, 3H).

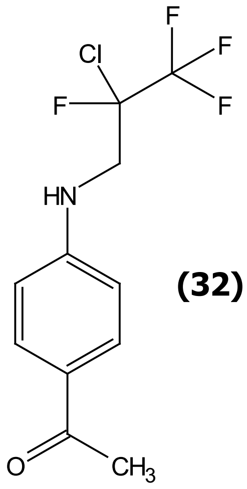

(32)

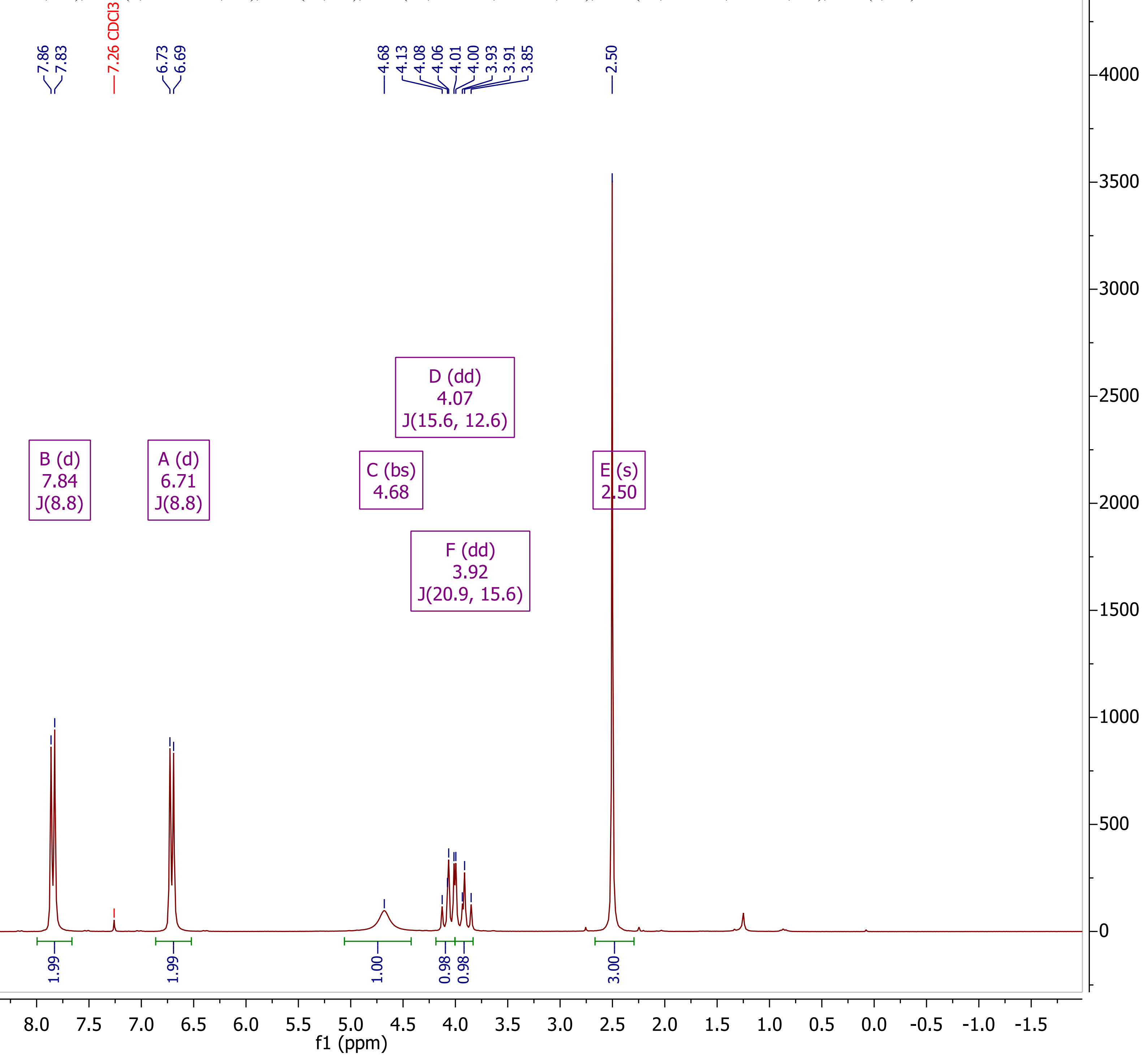

^{19}F NMR (235 MHz, Chloroform-*d*) δ -80.6 (d, *J* = 6.3 Hz), -130.2 (q, *J* = 6.3 Hz).

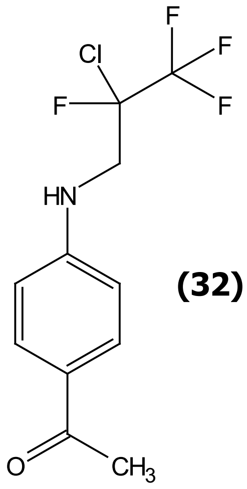

(32)

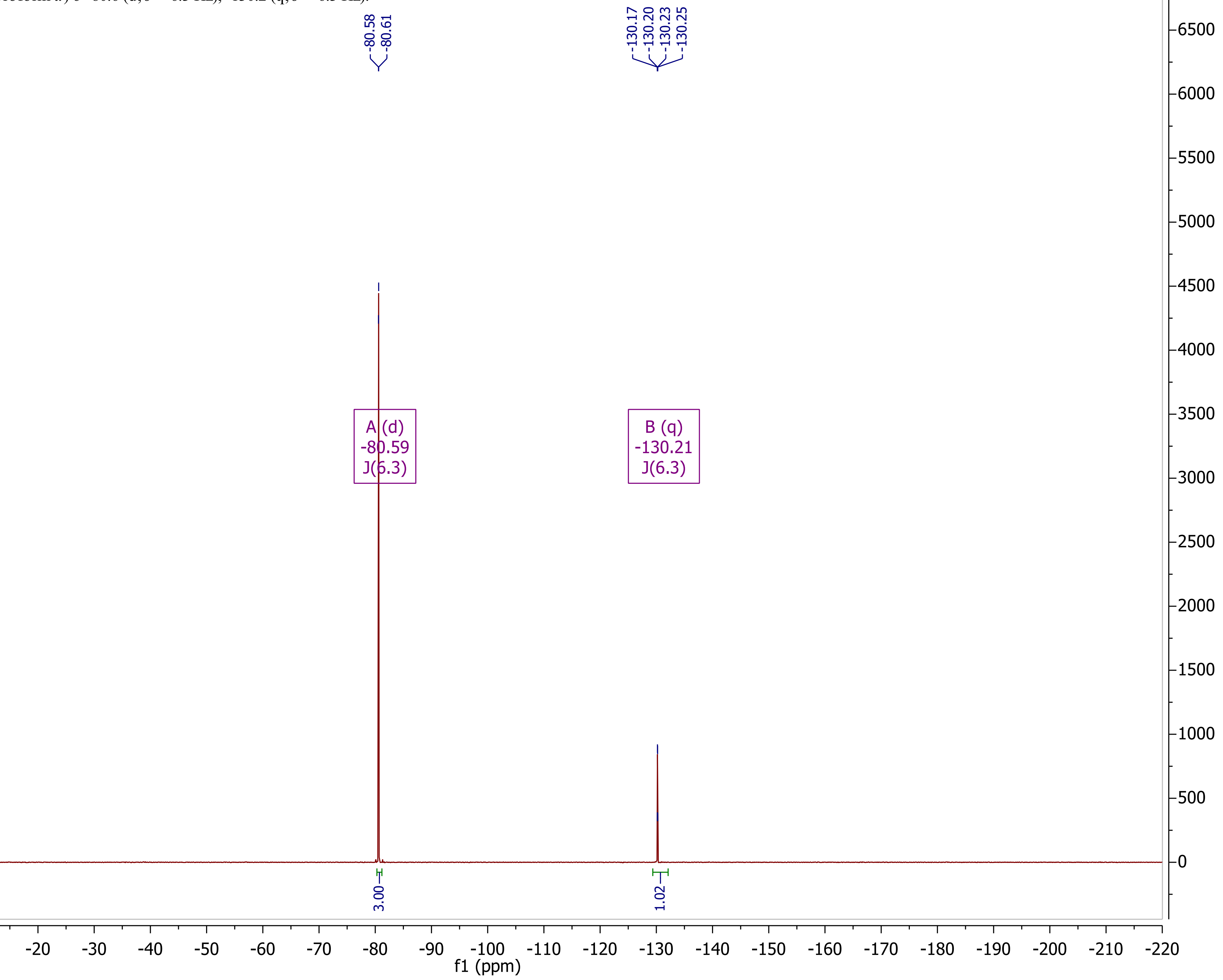

^{13}C NMR (63 MHz, Chloroform-*d*) δ 196.7, 150.8, 130.8, 128.4, 120.6 (qd, *J* = 284.9, 30.9 Hz), 112.2 (d, *J* = 1.1 Hz), 106.6 (dq, *J* = 255.6, 34.9 Hz), 48.0 (d, *J* = 22.4 Hz), 26.1.

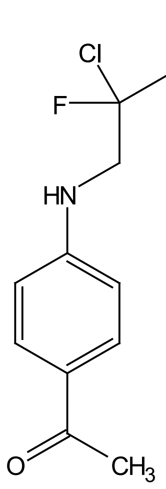

(32)

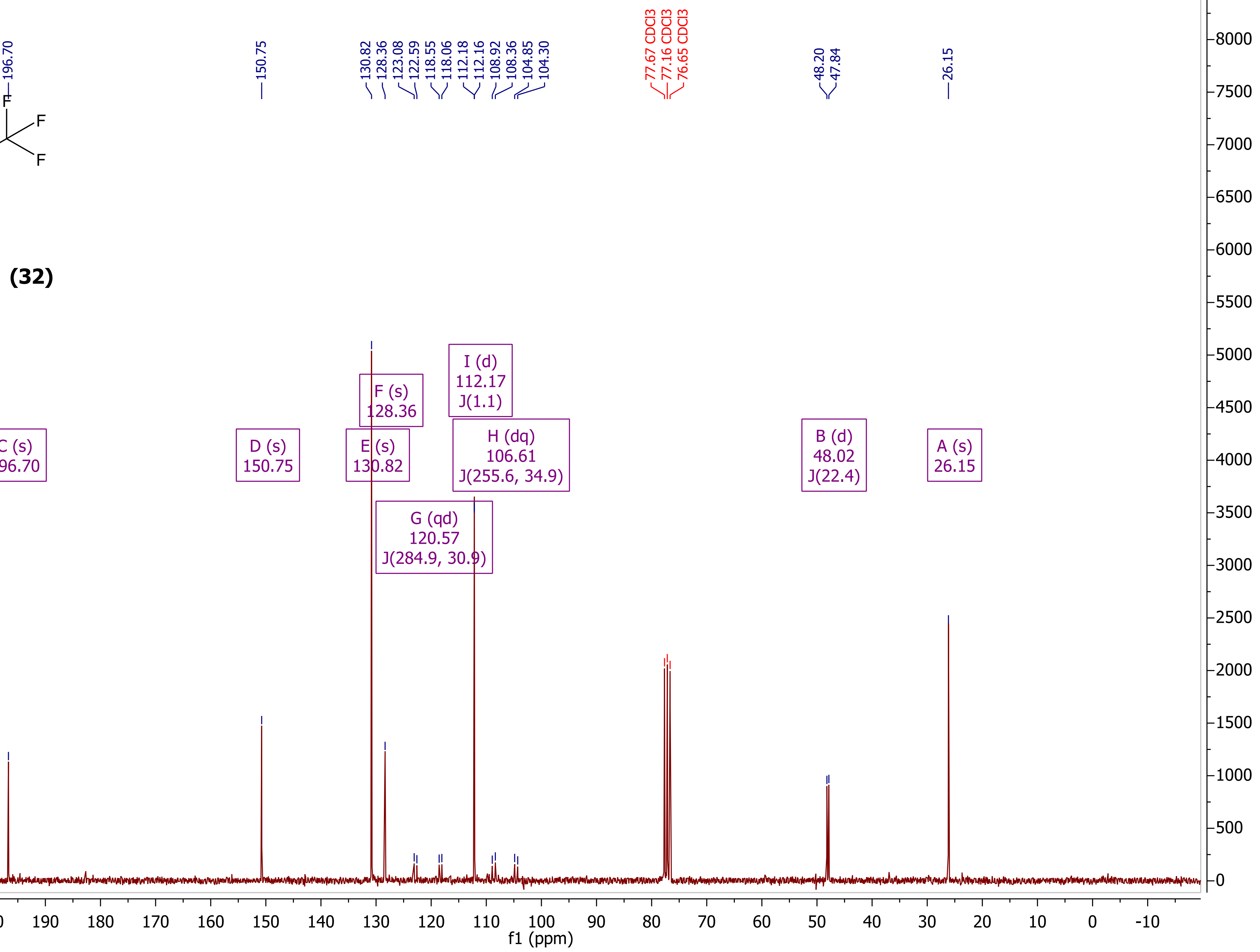

^{1}H NMR (250 MHz, Acetonitrile-d_3) δ 8.05 (d, J = 9.2 Hz, 2H), 6.81 (d, J = 9.2 Hz, 2H), 5.96 (bs, 1H), 4.33 – 3.78 (m, 2H).

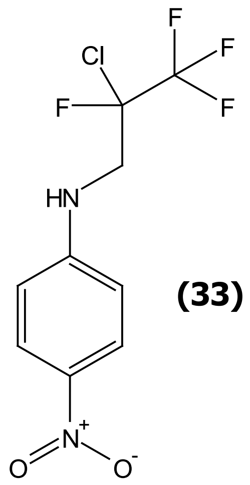

(33)

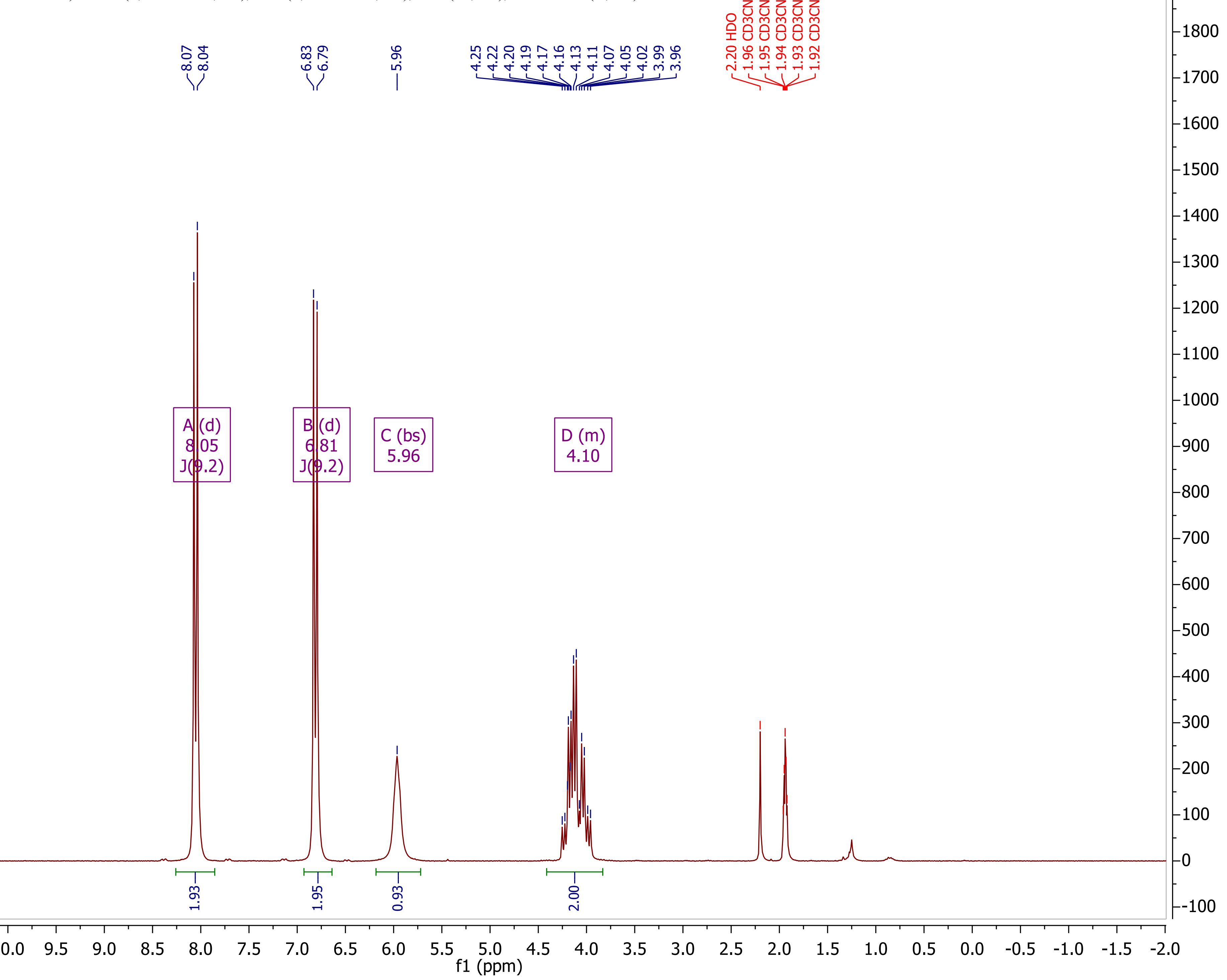

^{19}F NMR (235 MHz, Acetonitrile-d_3) δ -81.3 (d, J = 6.2 Hz), -130.4 (q, J = 6.0 Hz).

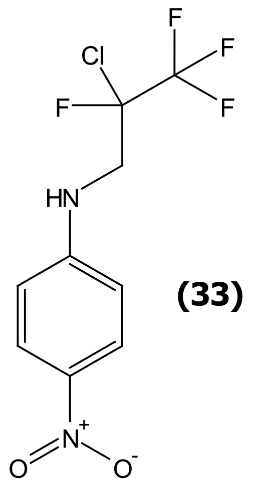

(33)

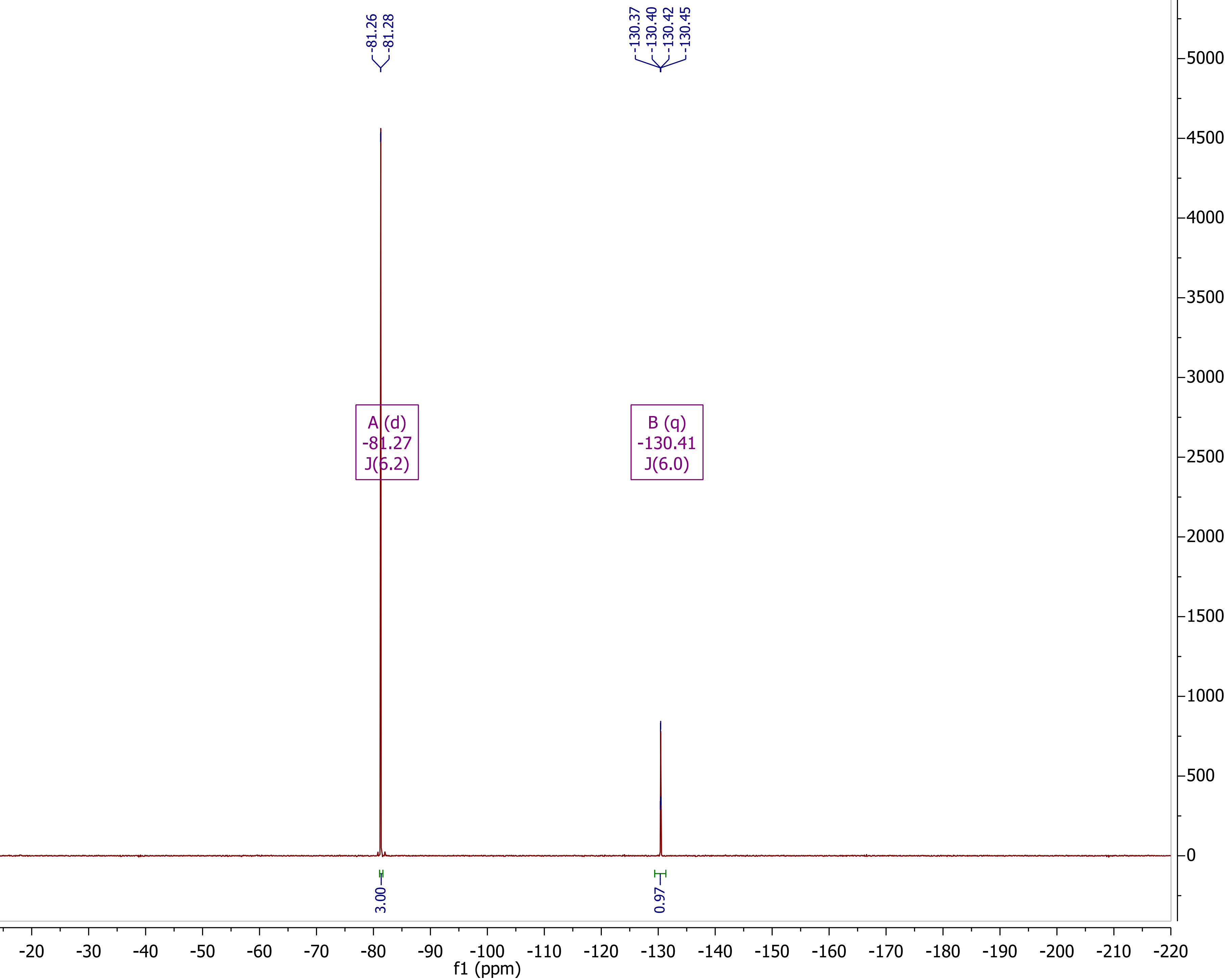

^{13}C NMR (63 MHz, Acetonitrile-d_3) δ 154.1, 139.9, 126.9, 121.6 (qd, J = 284.2, 31.2 Hz), 112.8 (d, J = 1.4 Hz), 107.9 (dq, J = 253.8, 34.6 Hz), 48.2 (d, J = 22.0 Hz).

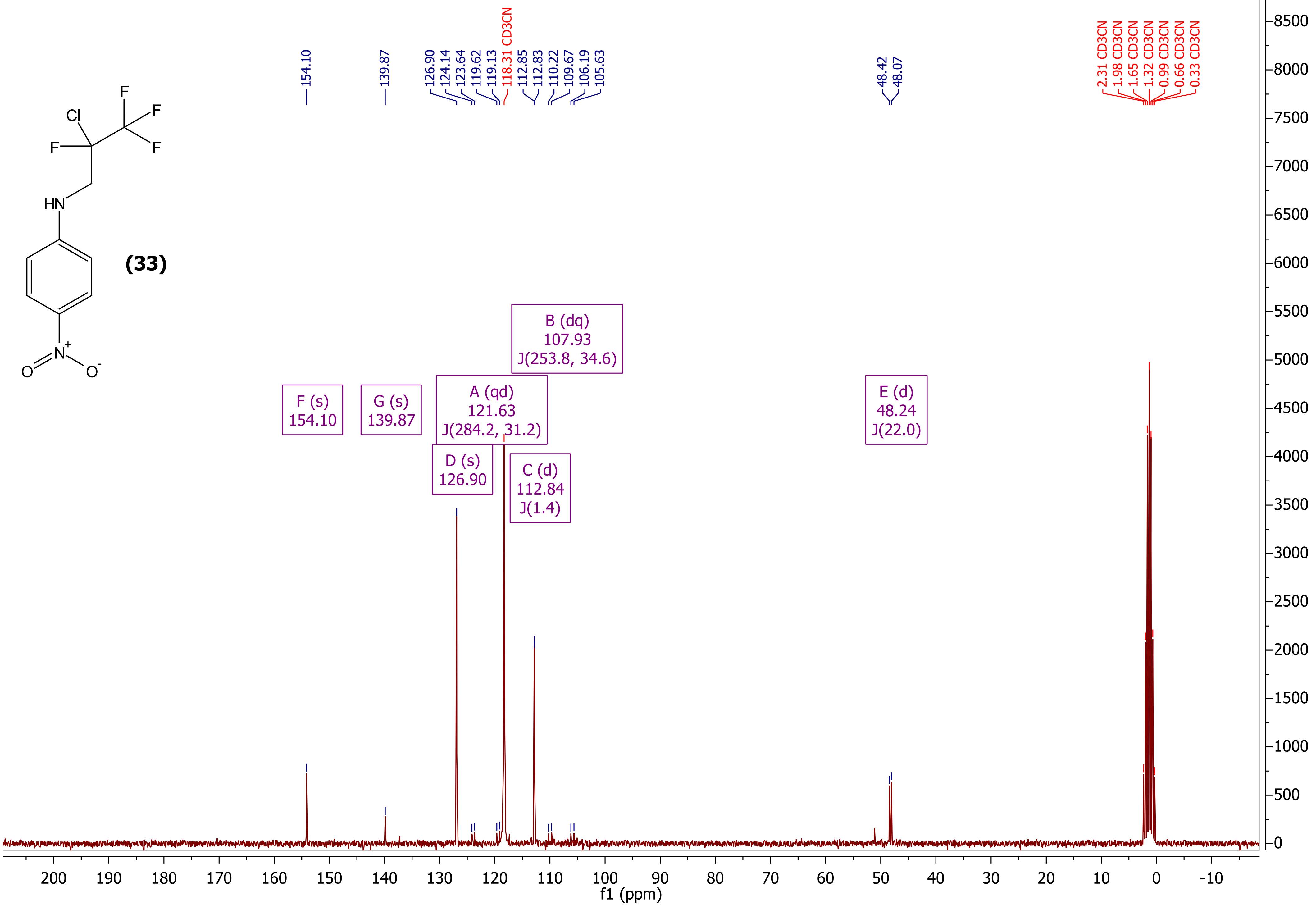

^{1}H NMR (250 MHz, Chloroform-*d*) δ 7.47 (d, *J* = 8.8 Hz, 2H), 6.72 (d, *J* = 8.8 Hz, 2H), 4.65 (bs, 1H), 4.06 (dd, *J* = 15.6, 12.4 Hz, 1H), 3.91 (dd, *J* = 20.8, 15.5 Hz, 1H).

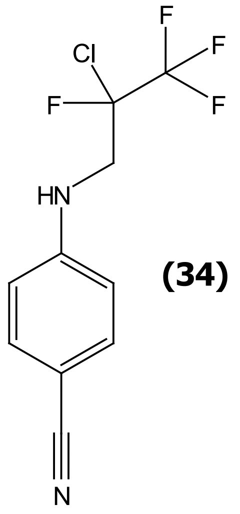

(34)

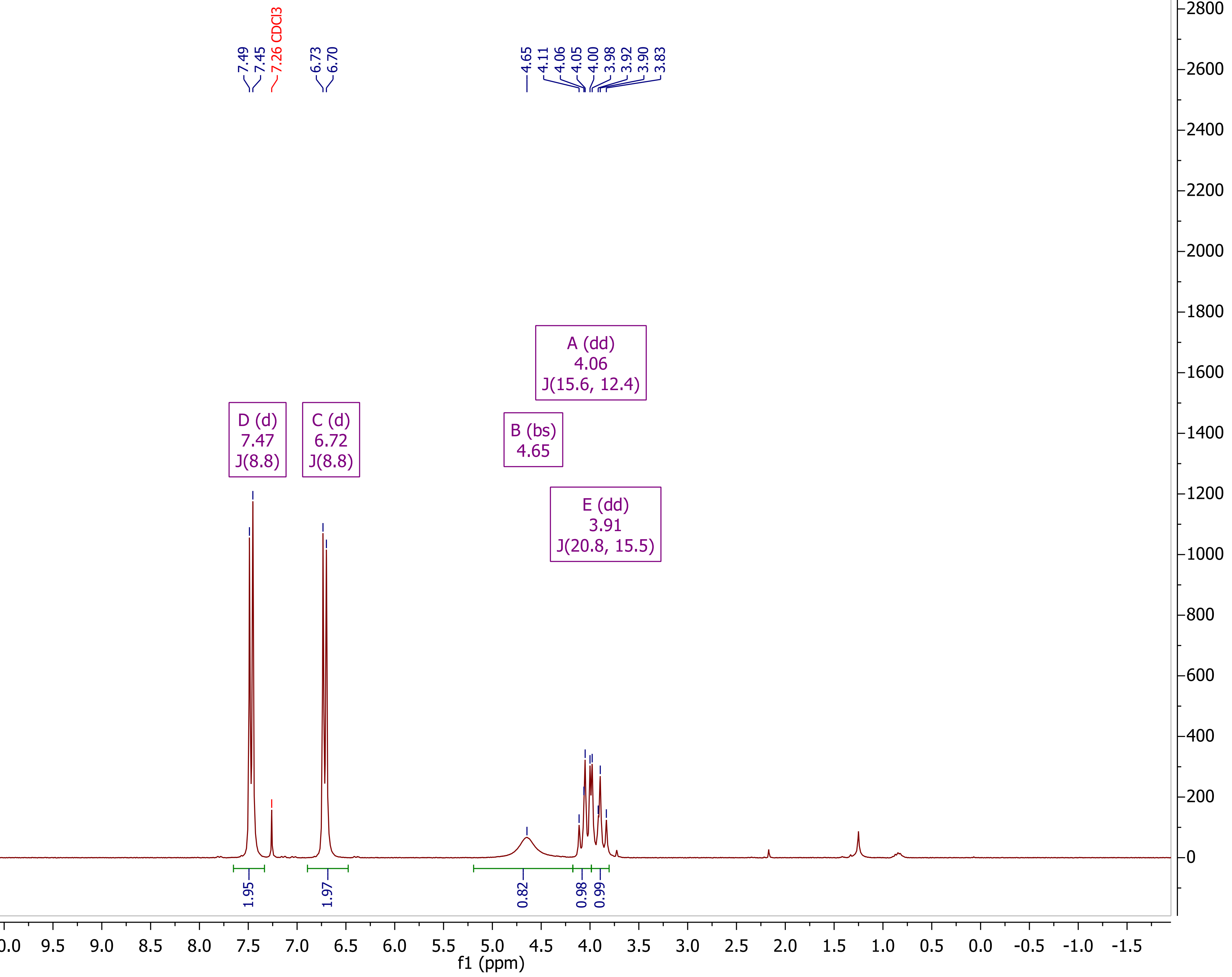

^{19}F NMR (235 MHz, Chloroform-*d*) δ -80.6 (d, *J* = 6.1 Hz), -130.3 (q, *J* = 6.2 Hz).

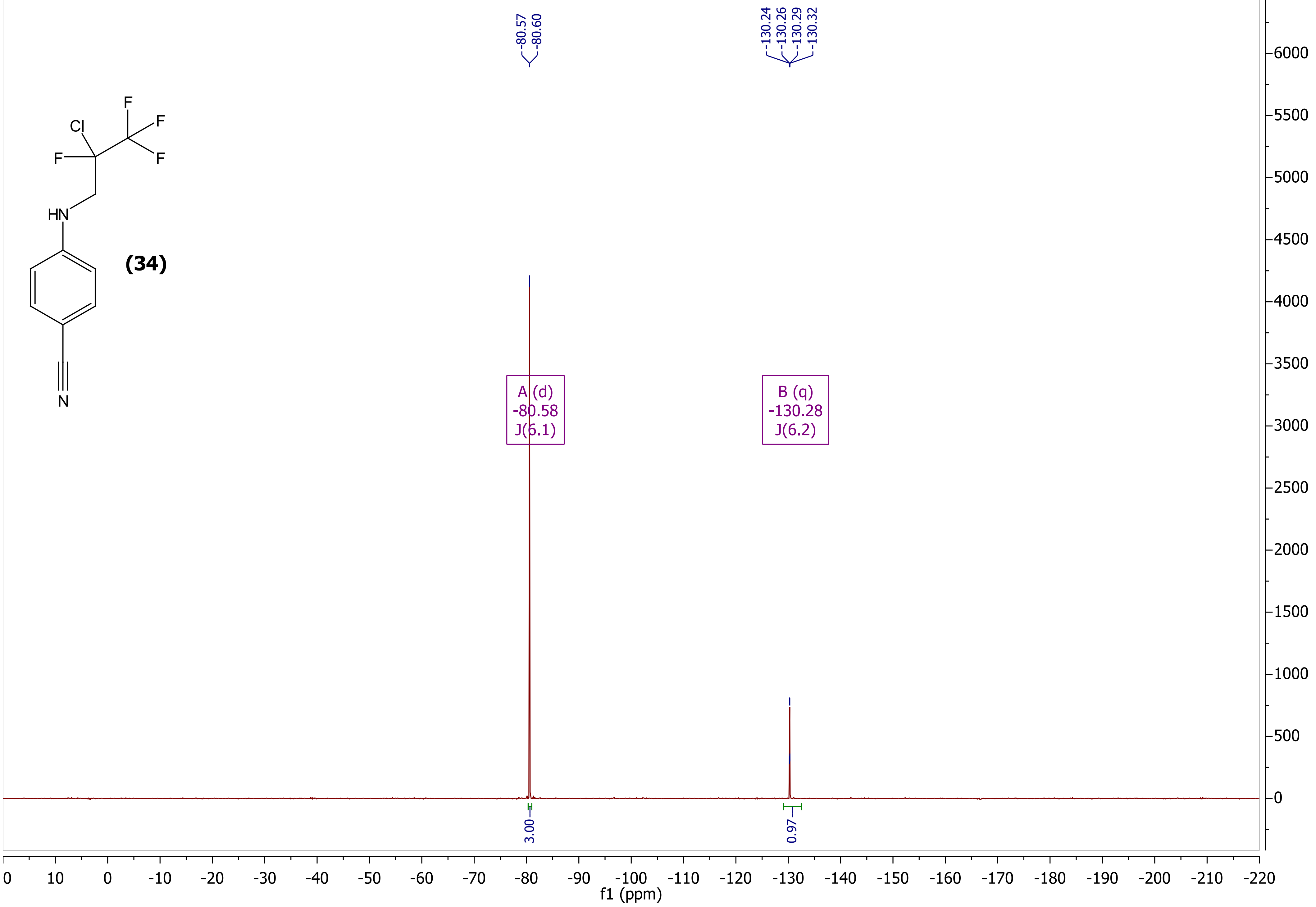

^{13}C NMR (63 MHz, Chloroform-*d*) δ 149.9, 133.9, 120.5 (qd, *J* = 285.2, 30.6 Hz), 119.9, 113.0 (d, *J* = 1.2 Hz), 106.5 (dq, *J* = 255.5, 35.1 Hz), 101.2, 48.0 (d, *J* = 22.3 Hz).

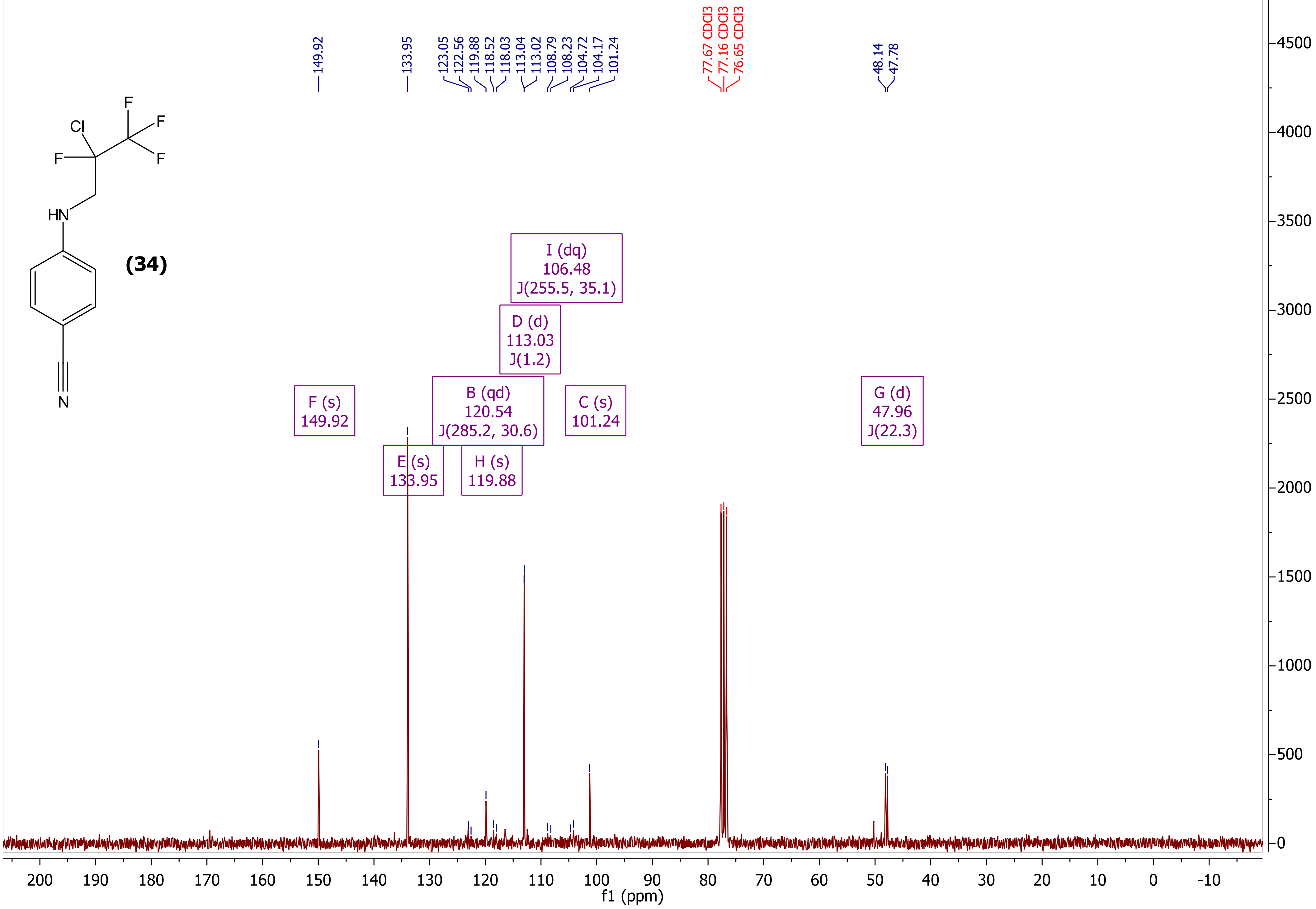

^{1}H NMR (500 MHz, DMSO-d_6) δ 8.54 (bs, 1H), 6.68 – 6.35 (m, 4H), 5.68 (bs, 1H), 3.95 (t, J = 15.3 Hz, 1H), 3.84 (dd, J = 22.3, 15.8 Hz, 1H).

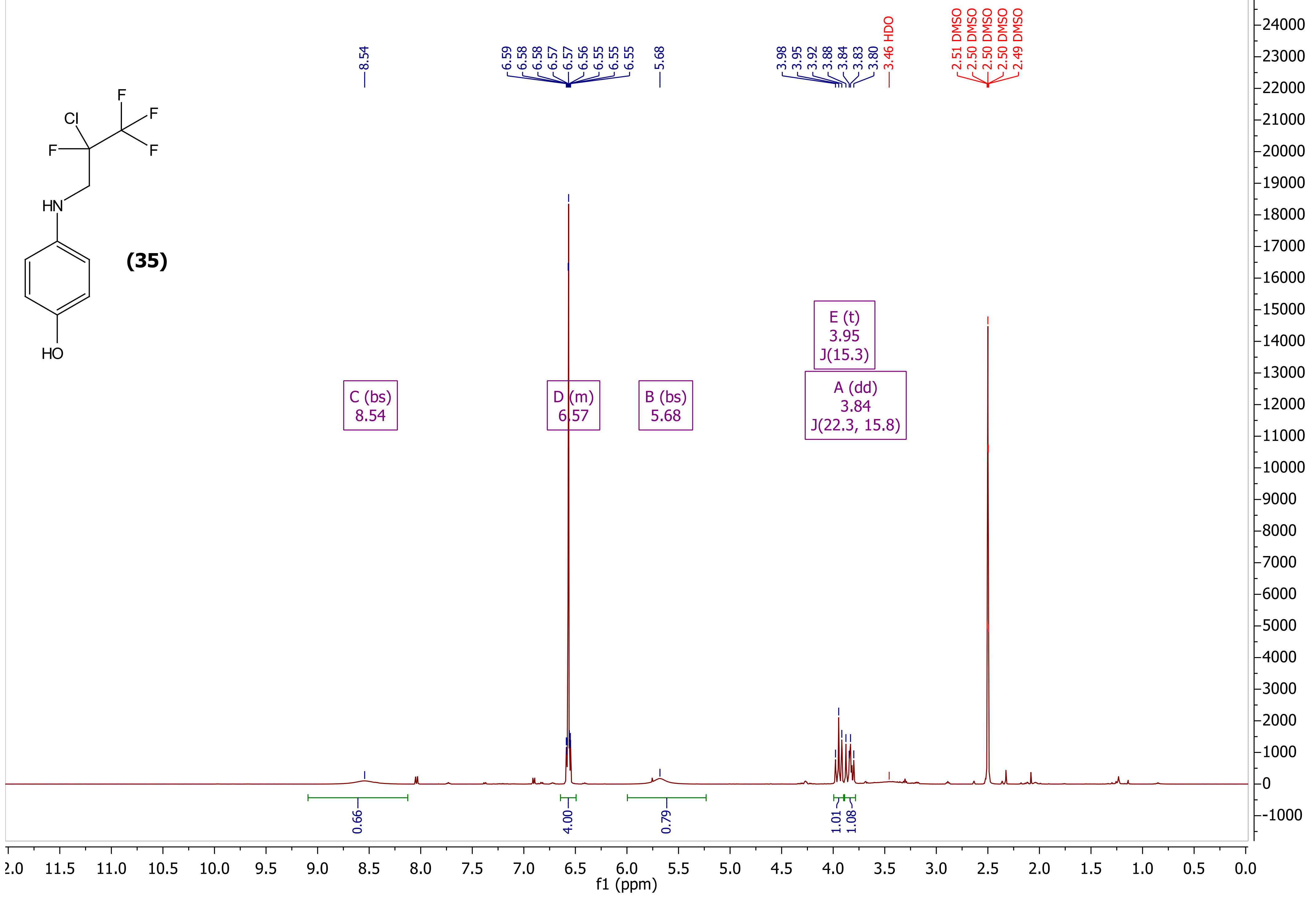

^{19}F NMR (376 MHz, DMSO-d_6) δ -79.4 (d, J = 6.4 Hz), -128.6 – -128.9 (m).

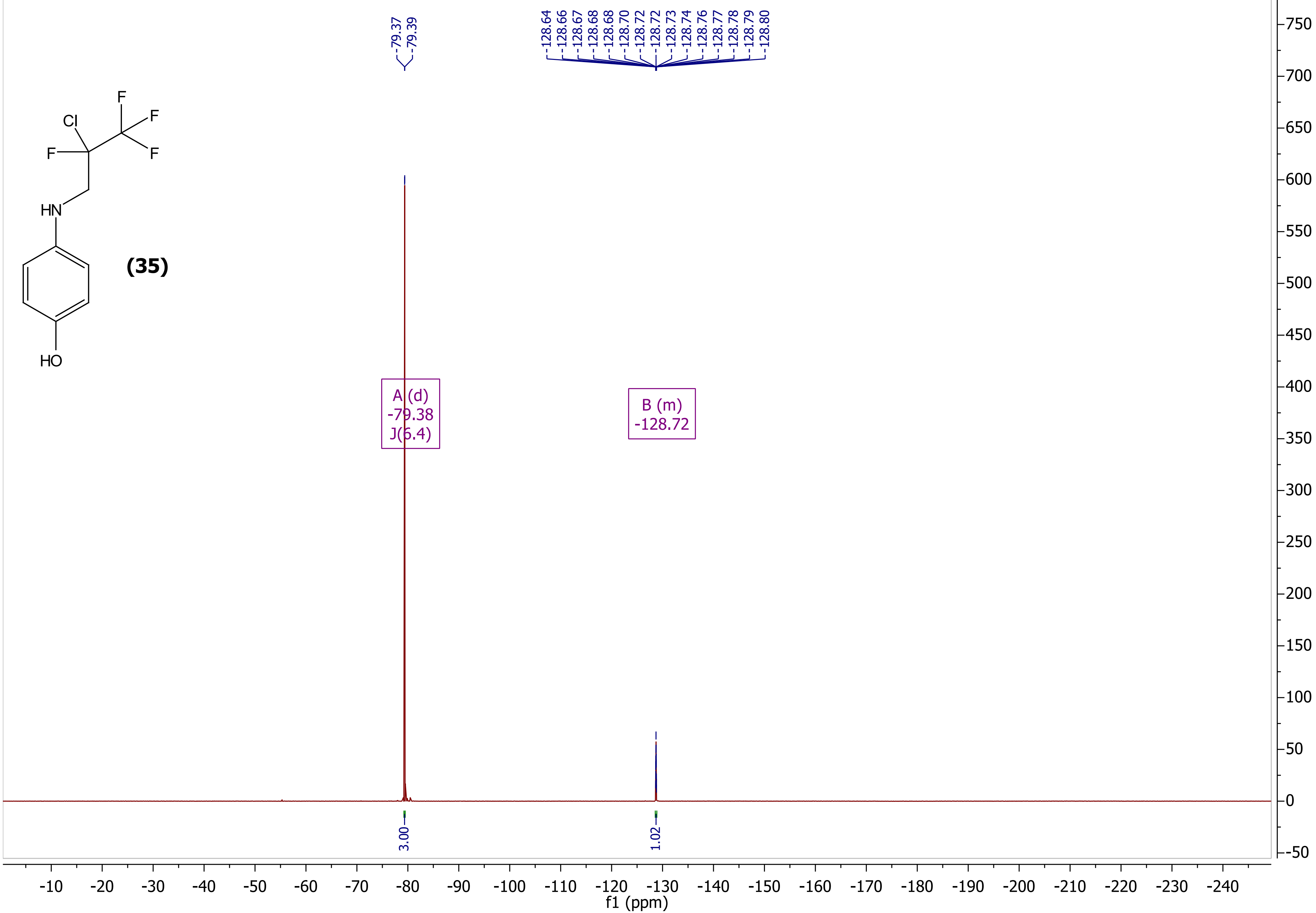

^{13}C NMR (126 MHz, DMSO-d_6) δ 149.2, 140.2, 120.6 (qd, J = 285.2, 31.8 Hz), 115.6, 113.9, 107.7 (dq, J = 253.2, 33.3 Hz), 49.0 (d, J = 21.3 Hz).

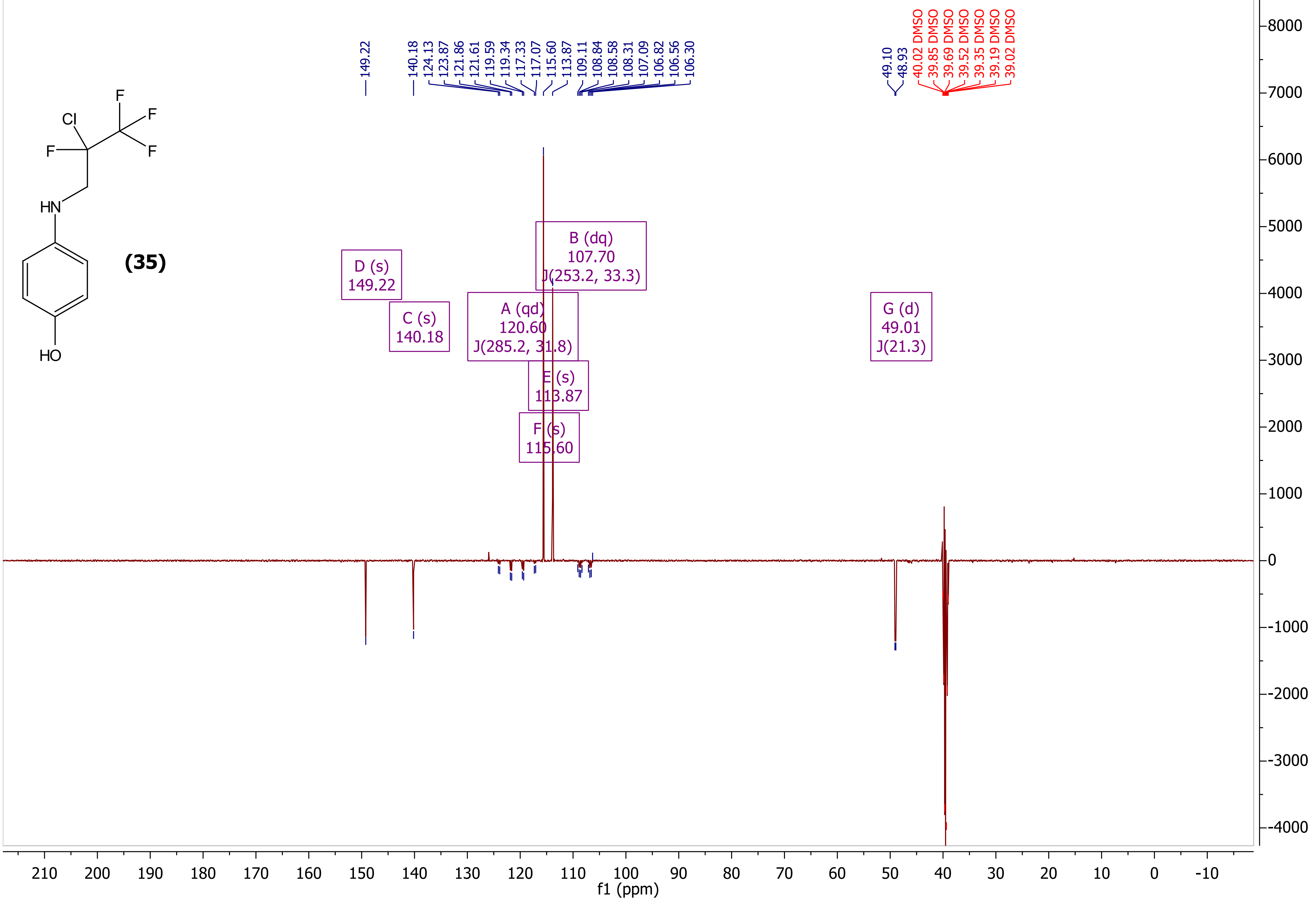

^{1}H NMR (500 MHz, DMSO-d_6) δ 6.76 – 6.71 (m, 2H), 6.70 – 6.66 (m, 2H), 5.90 (bs, 1H), 4.00 (t, J = 15.4 Hz, 1H), 3.89 (dd, J = 22.7, 16.4 Hz, 1H), 3.64 (s, 3H).

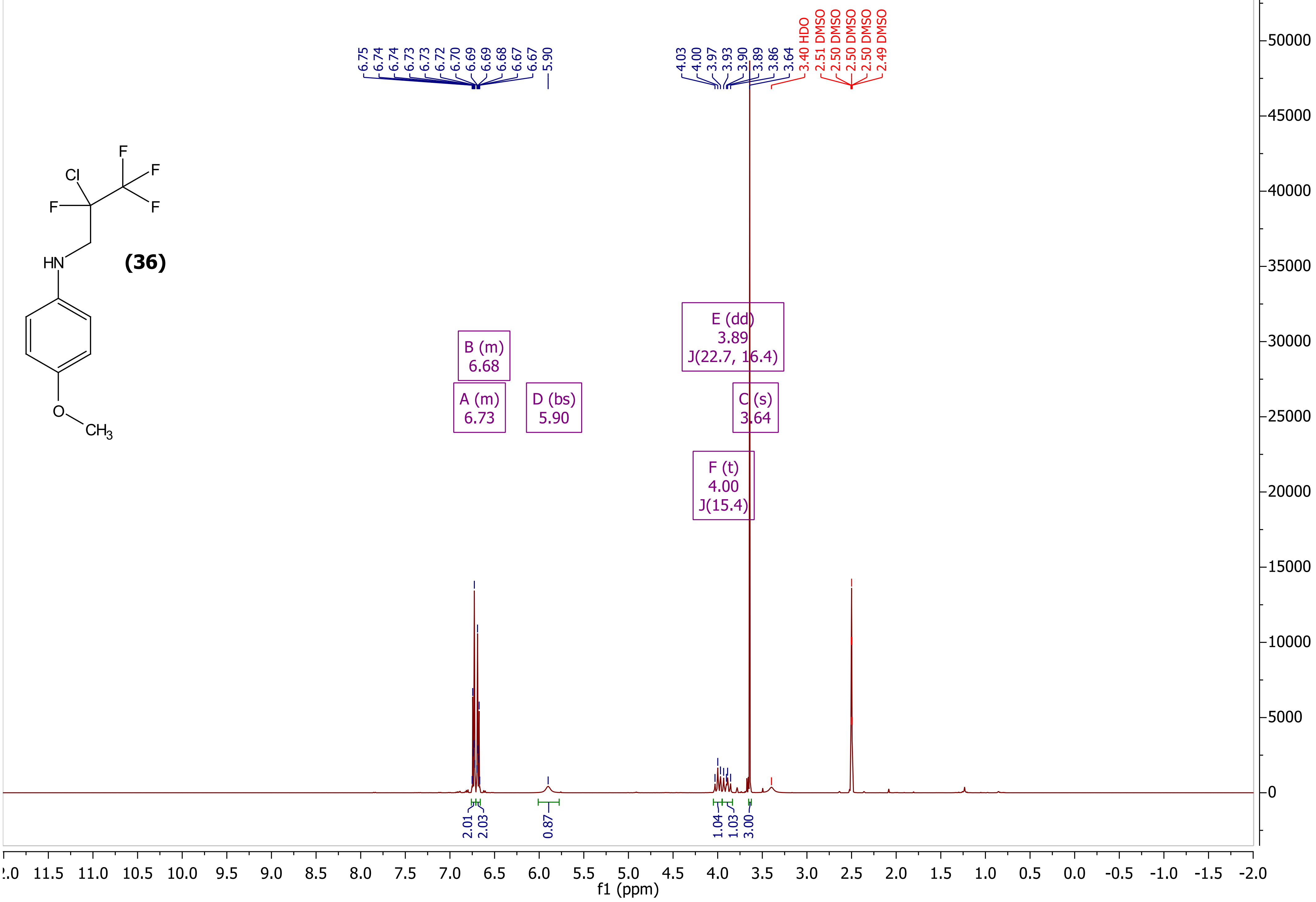

^{19}F NMR (376 MHz, DMSO-d_6) δ -79.4 (d, J = 6.4 Hz), -128.5 – -128.9 (m).

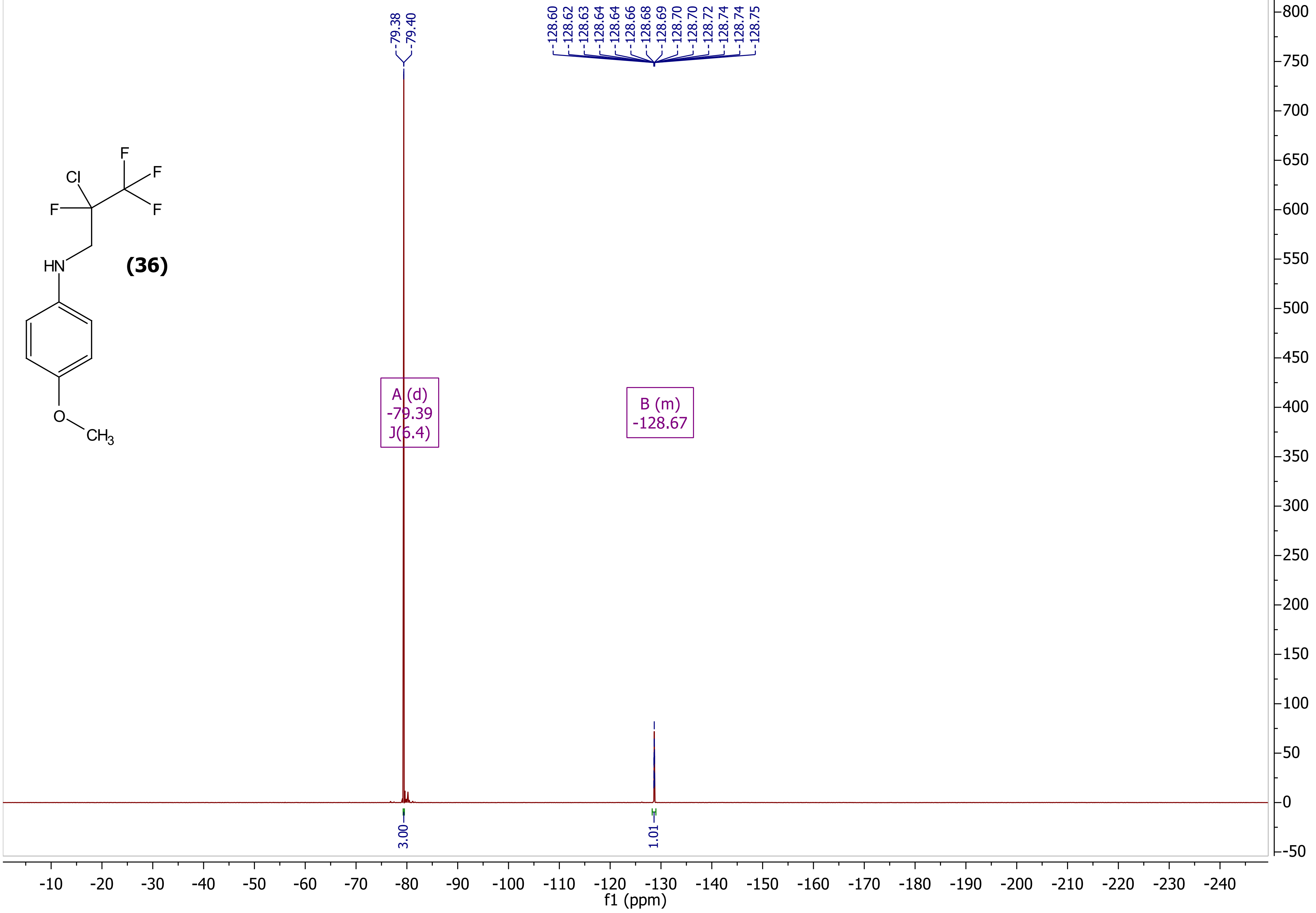

(36)

^{13}C NMR (126 MHz, DMSO-d_6) δ 151.5, 141.5, 120.6 (qd, J = 285.2, 31.7 Hz), 114.5, 113.6, 107.7 (dq, J = 254.0, 33.4 Hz), 55.3, 48.6 (d, J = 21.4 Hz).

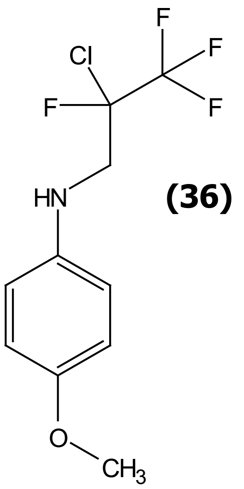

(36)

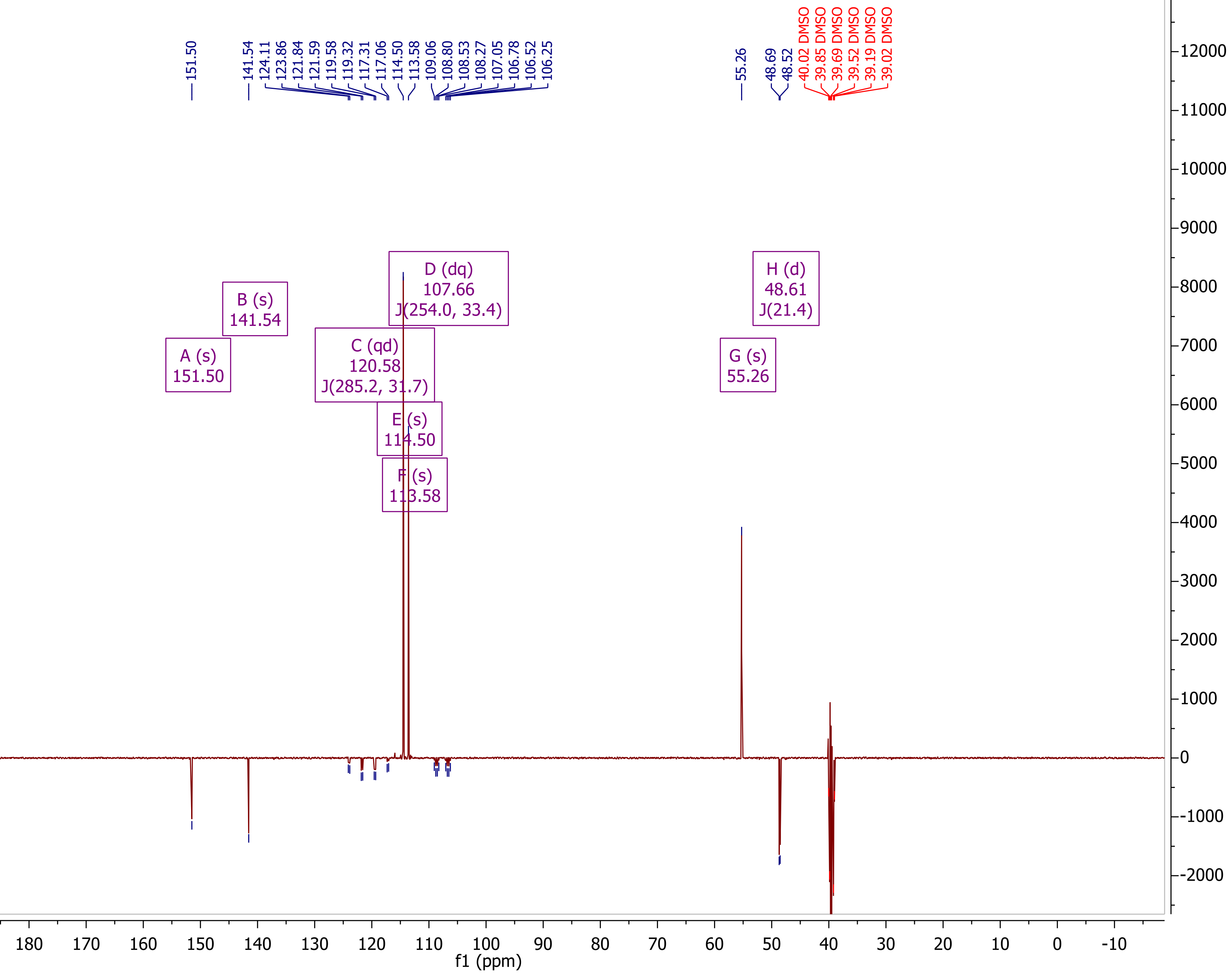

^{1}H NMR (250 MHz, Chloroform-*d*) δ 7.08 – 6.55 (m, 4H), 4.52 (bs, 1H), 4.14 – 3.67 (m, 5H).

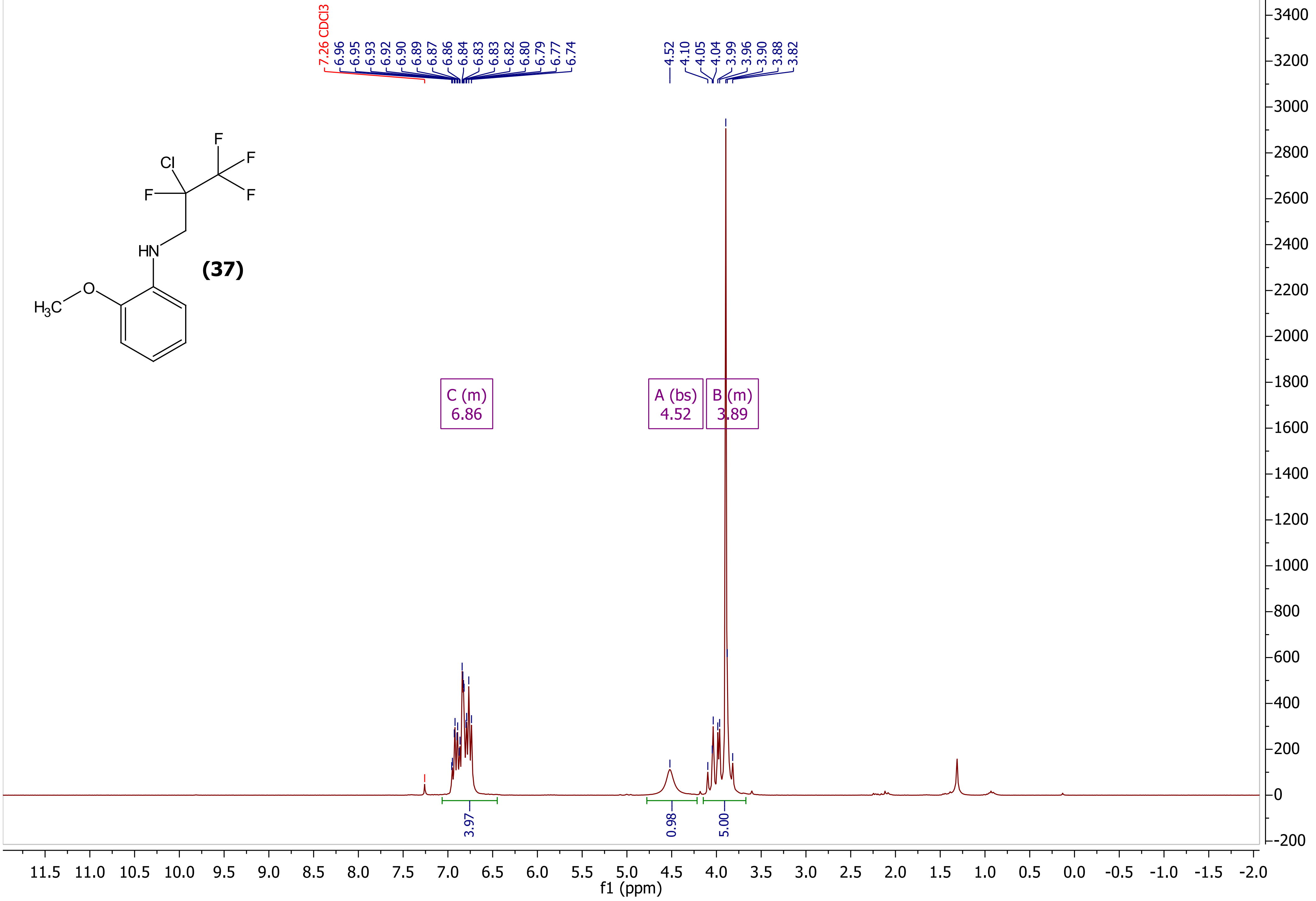

^{19}F NMR (235 MHz, Chloroform-*d*) δ -80.6 (d, *J* = 6.1 Hz), -130.1 (q, *J* = 6.2 Hz).

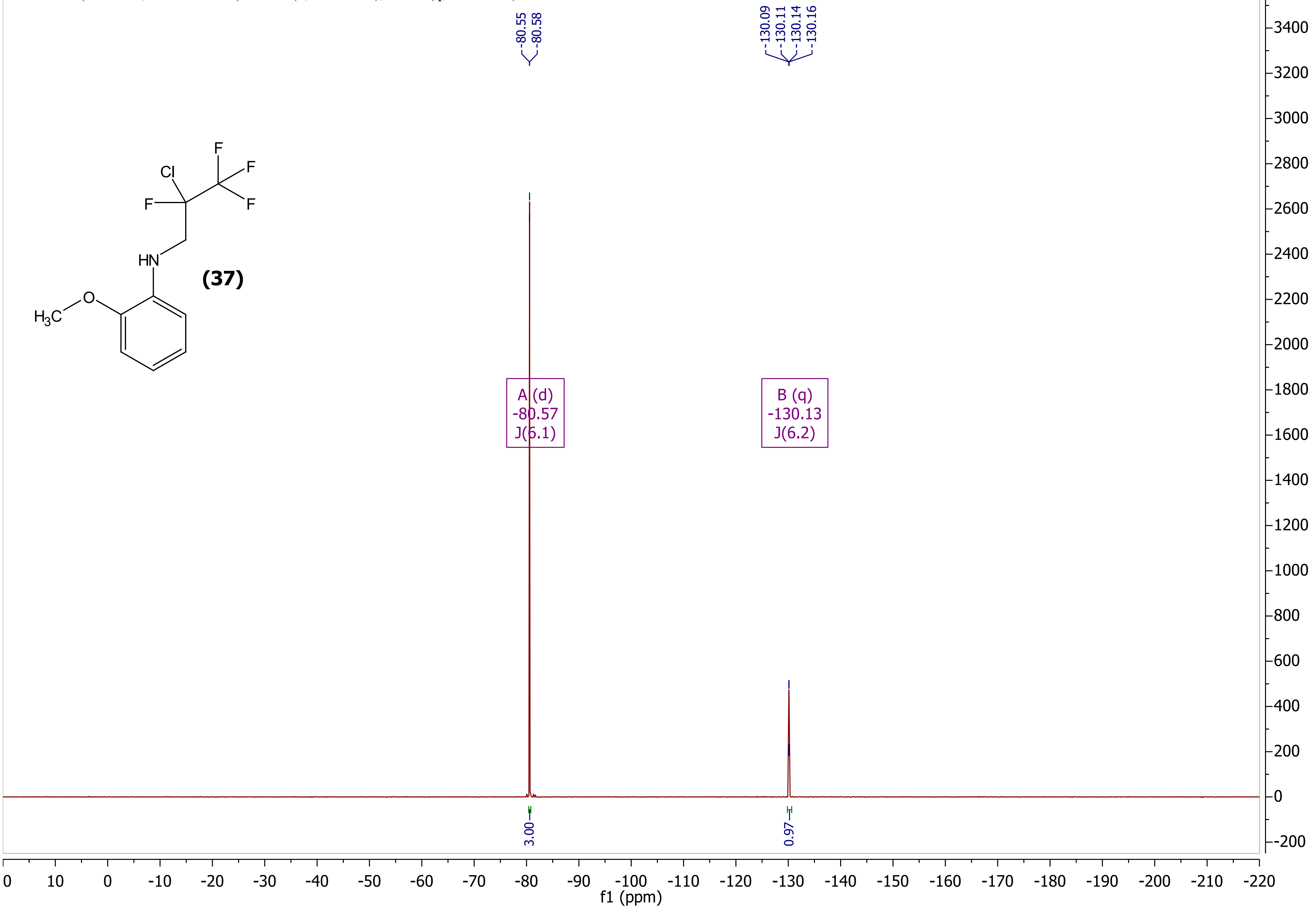

^{13}C NMR (63 MHz, Chloroform-*d*) δ 147.1, 136.5, 121.3, 120.8 (qd, *J* = 285.2, 31.0 Hz), 118.4, 110.4 (d, *J* = 2.0 Hz), 110.2, 107.1 (dq, *J* = 255.4, 34.5 Hz), 55.7, 48.9 (d, *J* = 22.1 Hz).

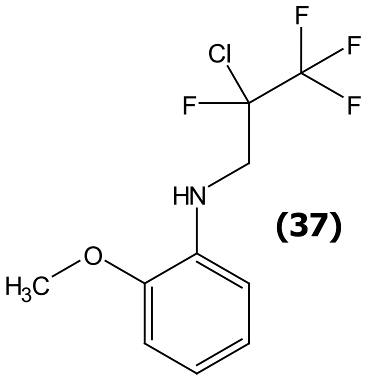

(37)

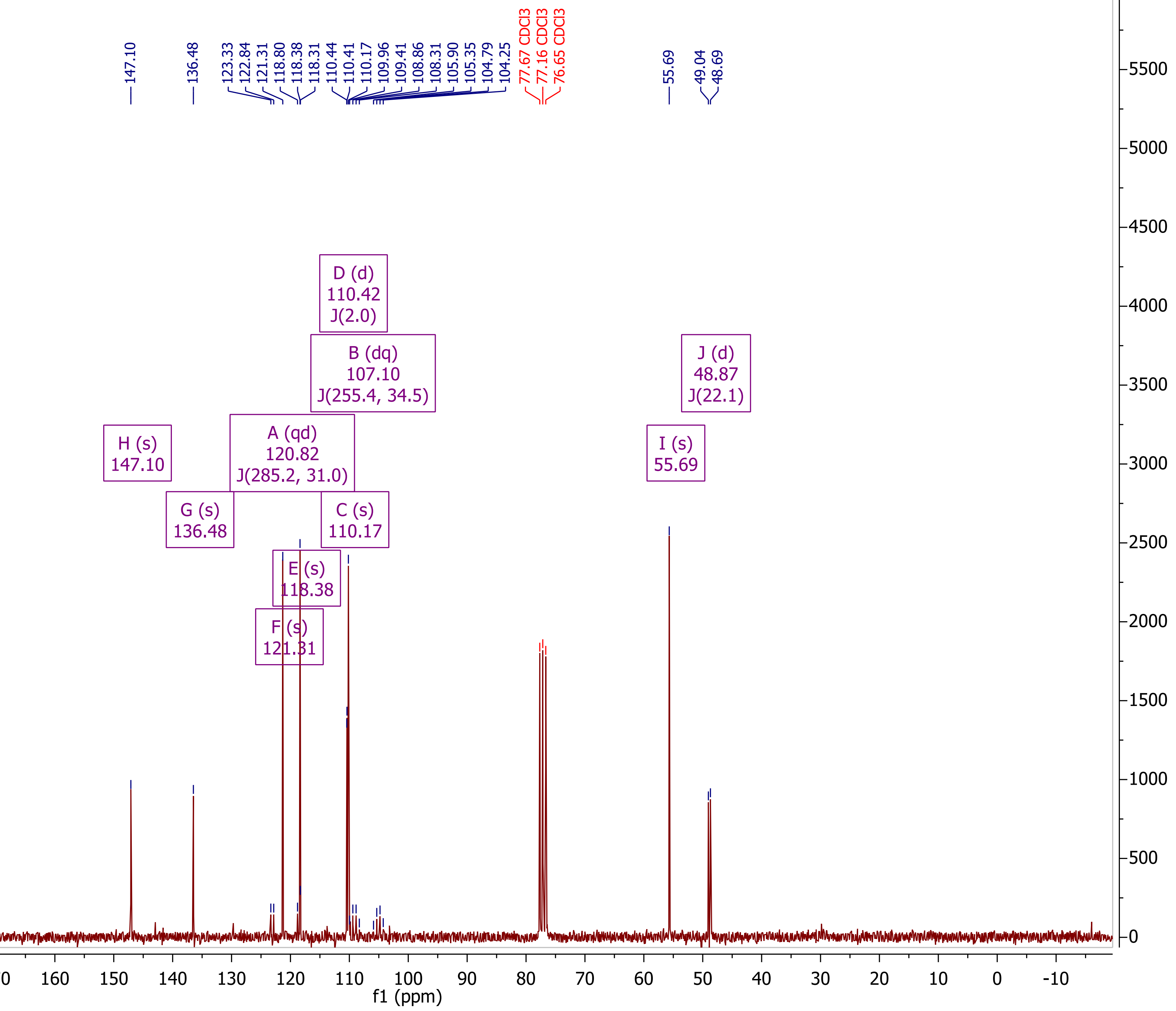

^{1}H NMR (250 MHz, Chloroform-*d*) δ 7.15 (t, *J* = 8.1 Hz, 1H), 6.45 – 6.18 (m, 3H), 4.11 – 3.68 (m, 6H).

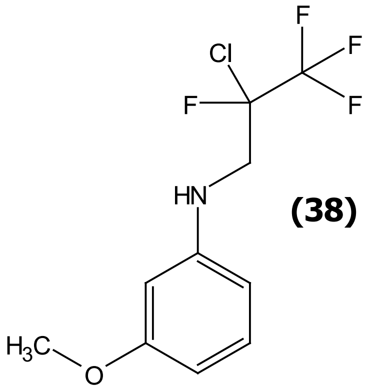

(38)

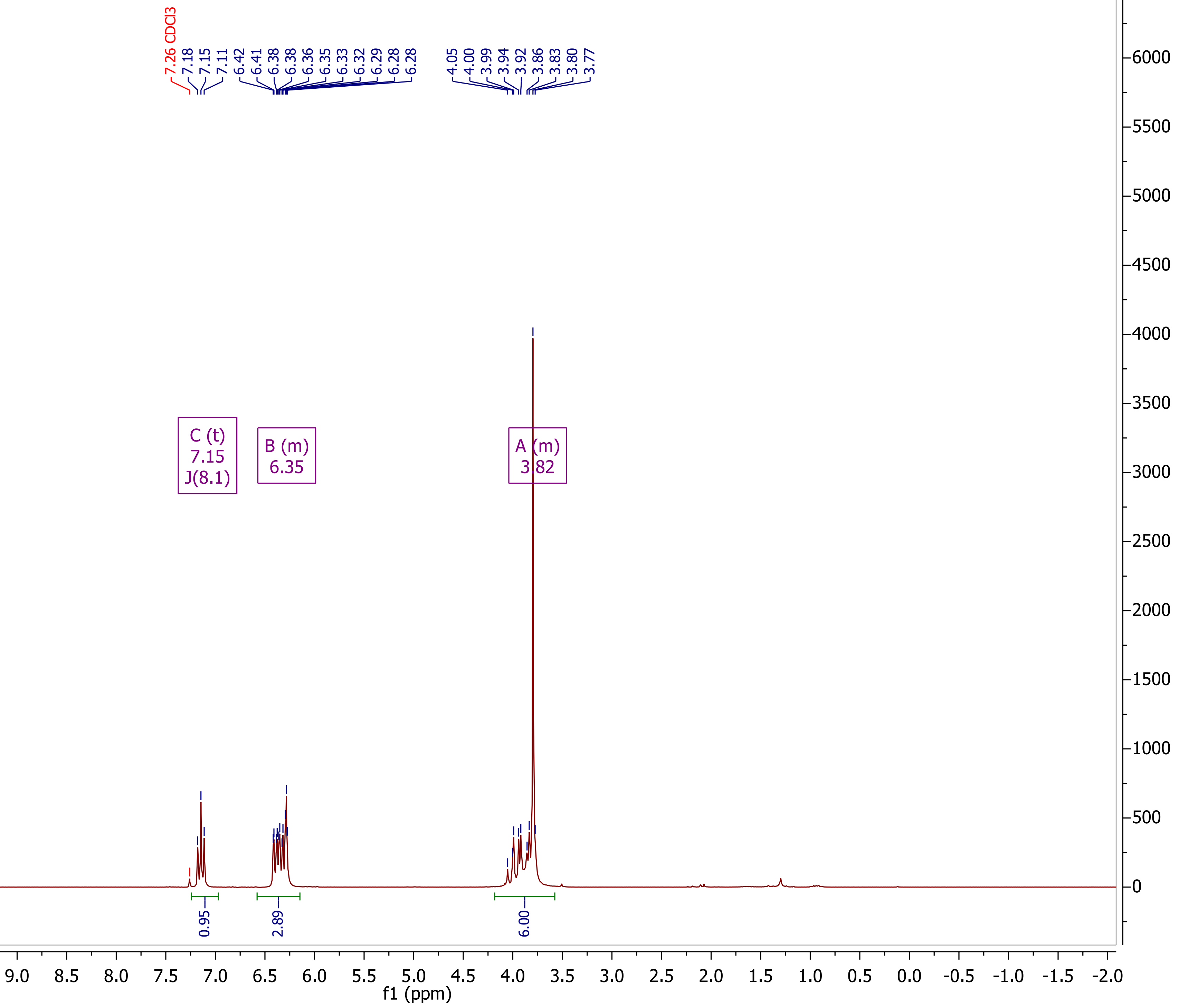

^{19}F NMR (235 MHz, Chloroform-*d*) δ -80.6 (d, *J* = 6.3 Hz), -130.2 (q, *J* = 6.2 Hz).

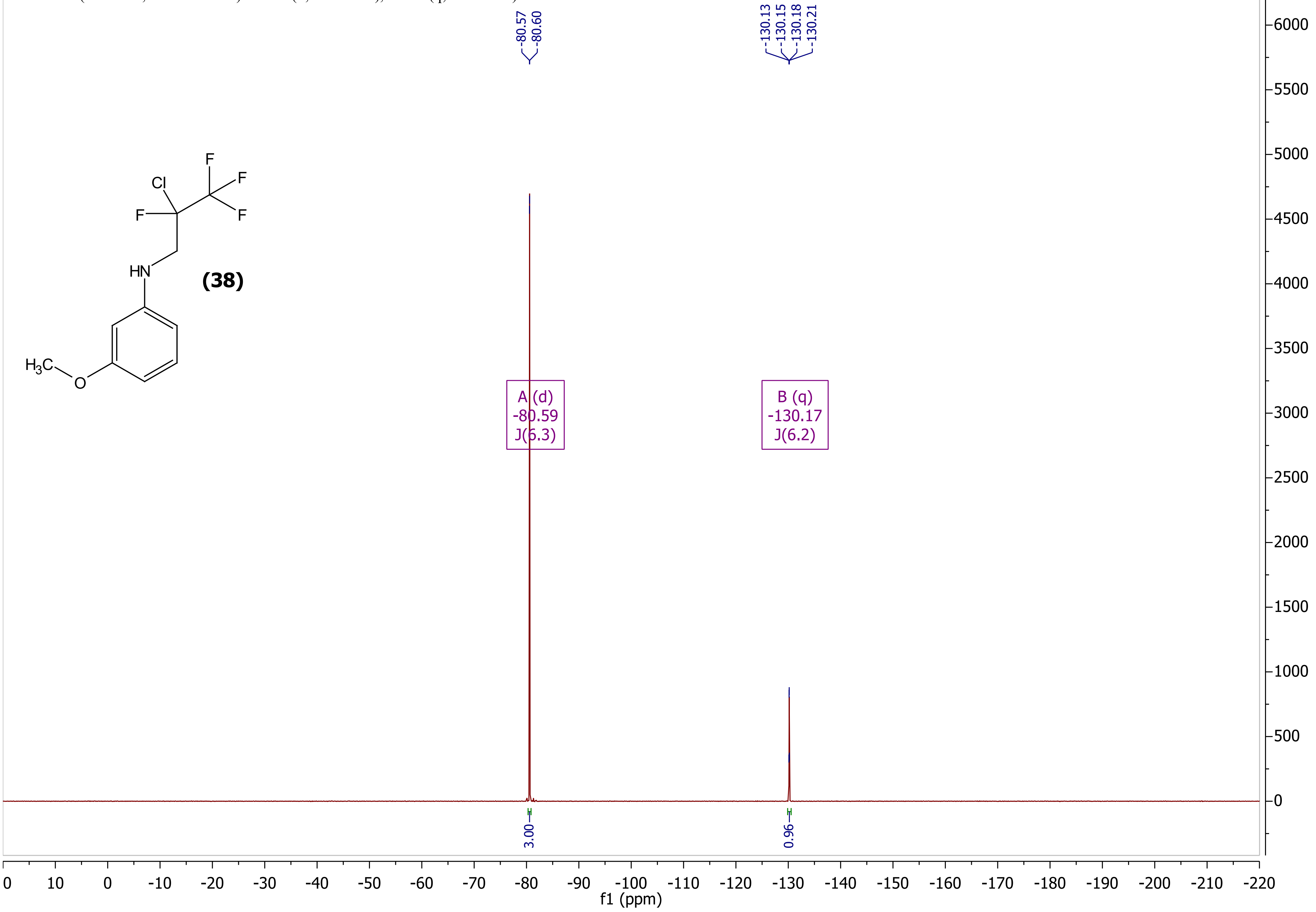

^{13}C NMR (63 MHz, Chloroform-*d*) δ 161.0, 147.9, 130.3, 120.7 (qd, *J* = 284.8, 31.2 Hz), 106.9 (dq, *J* = 255.2, 35.0 Hz), 106.3, 104.2, 99.8 (d, *J* = 1.2 Hz), 55.2, 49.0 (d, *J* = 22.2 Hz).

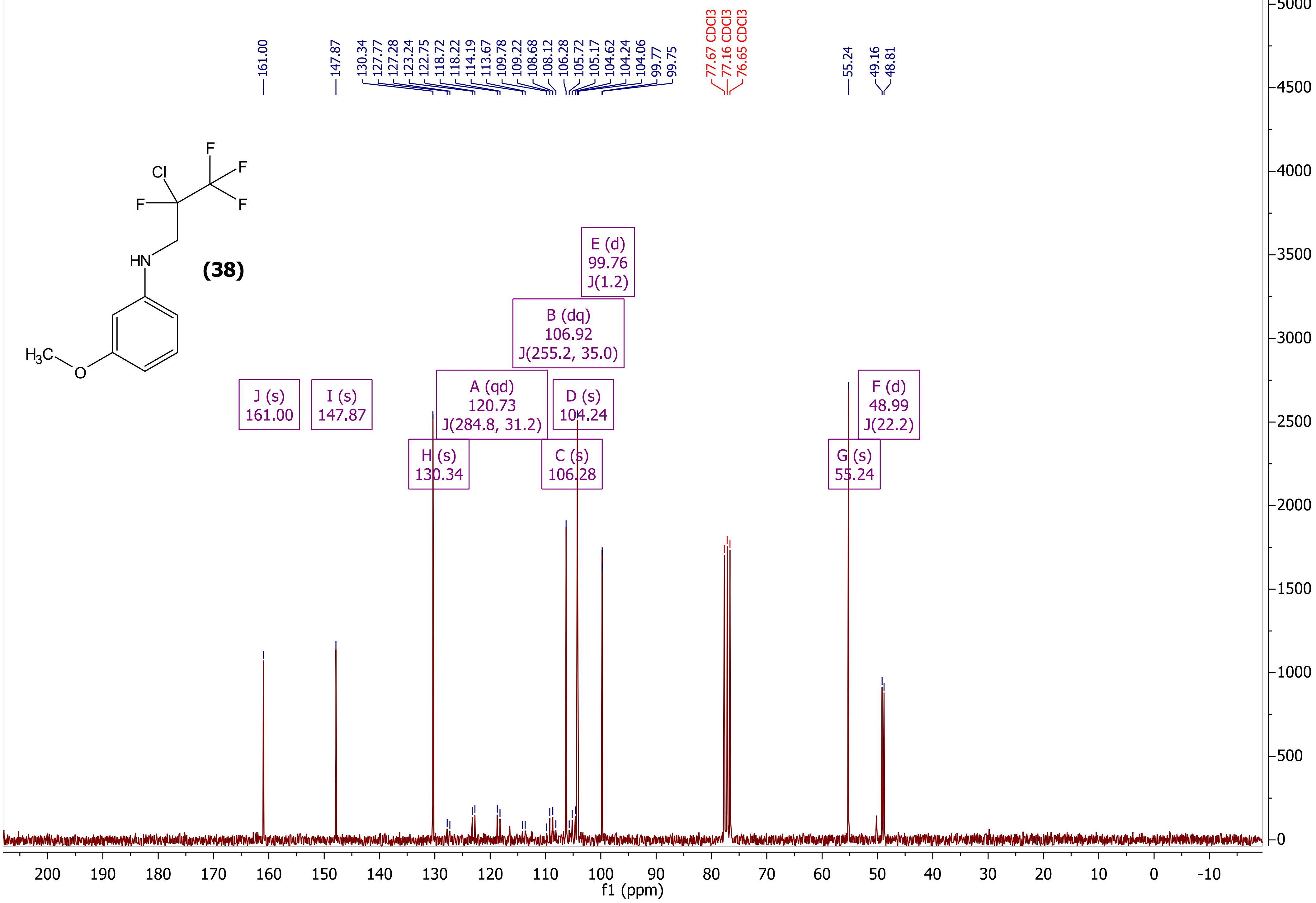

^{1}H NMR (500 MHz, DMSO-d_6) δ 6.64 (dd, *J* = 8.6, 1.2 Hz, 1H), 6.52 (d, *J* = 2.7 Hz, 1H), 6.38 (dd, *J* = 8.7, 2.7 Hz, 1H), 5.09 (bs, 1H), 4.10 – 3.89 (m, 2H), 3.79 (s, 3H), 3.67 (s, 3H).

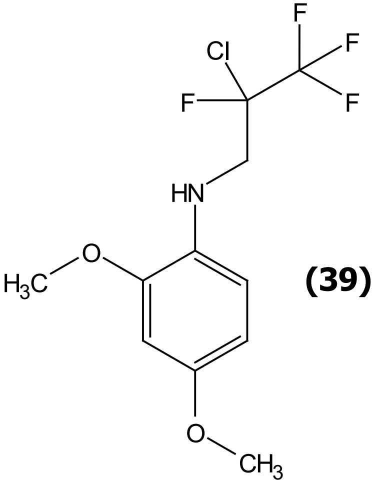

(39)

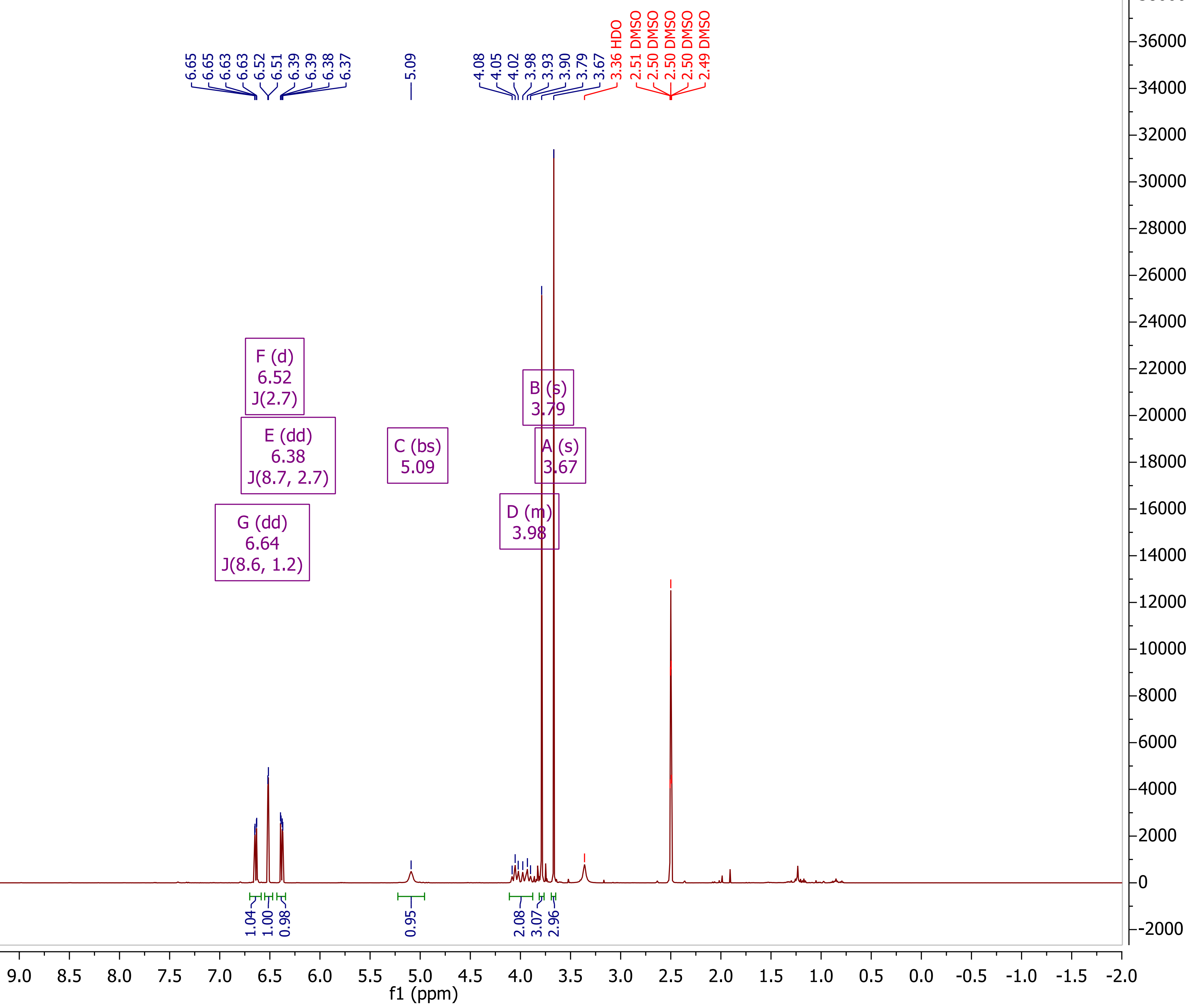

^{19}F NMR (376 MHz, DMSO-d_6) δ -79.5 (d, J = 6.4 Hz), -128.4 – -128.6 (m).

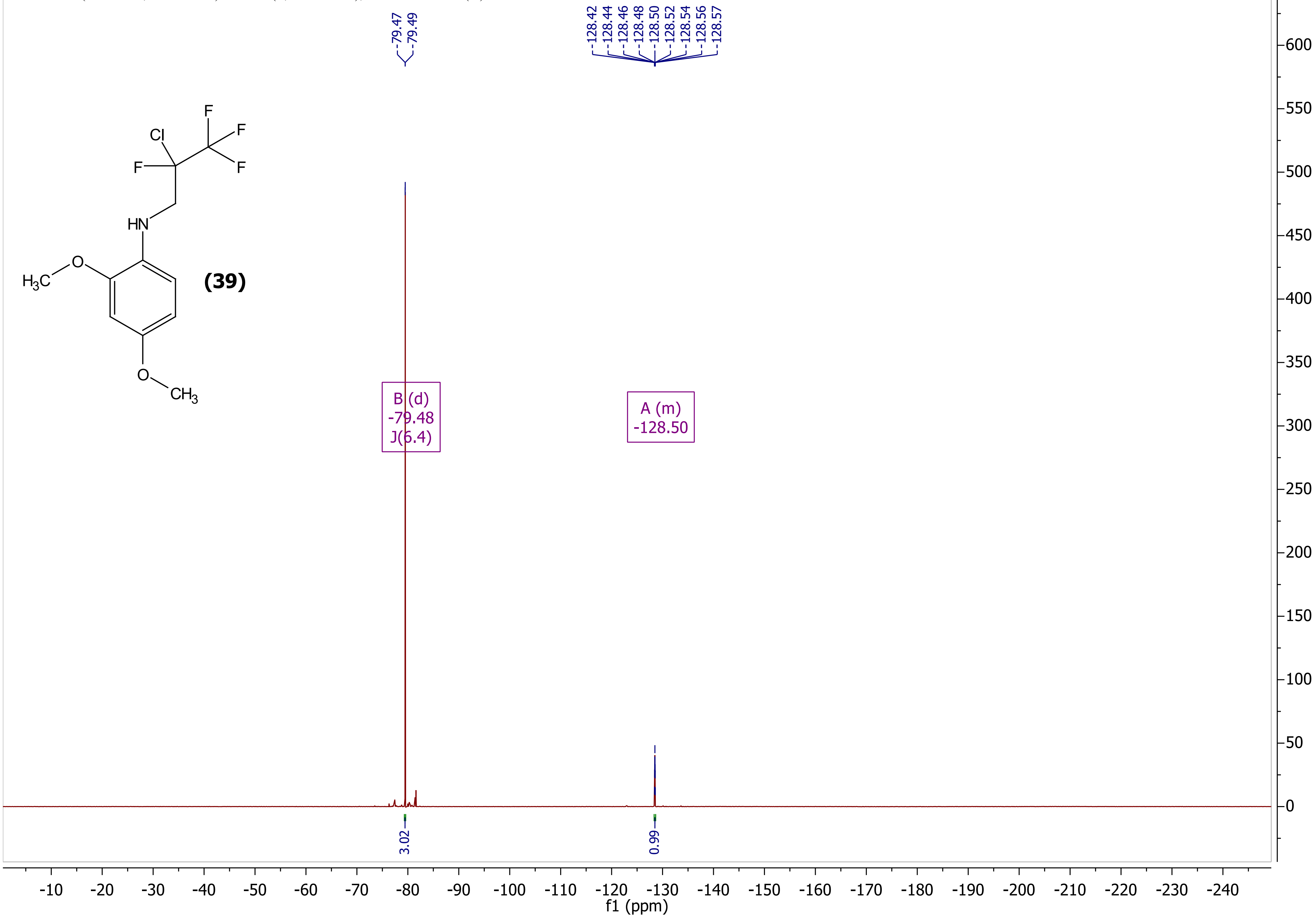

^{13}C NMR (126 MHz, DMSO-d_6) δ 151.9, 147.4, 130.6, 120.6 (qd, J = 285.2, 31.8 Hz), 110.4 (d, J = 2.7 Hz), 107.8 (dq, J = 254.2, 33.4 Hz), 104.0, 99.2, 55.6, 55.3, 48.2 (d, J = 21.2 Hz).

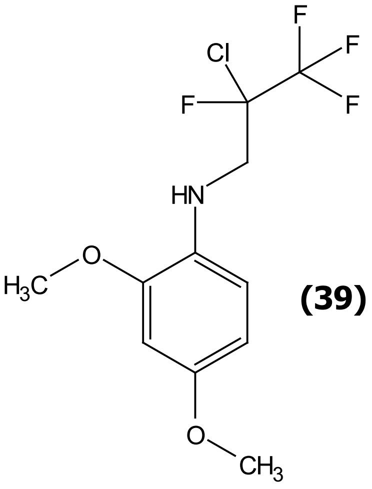

(39)

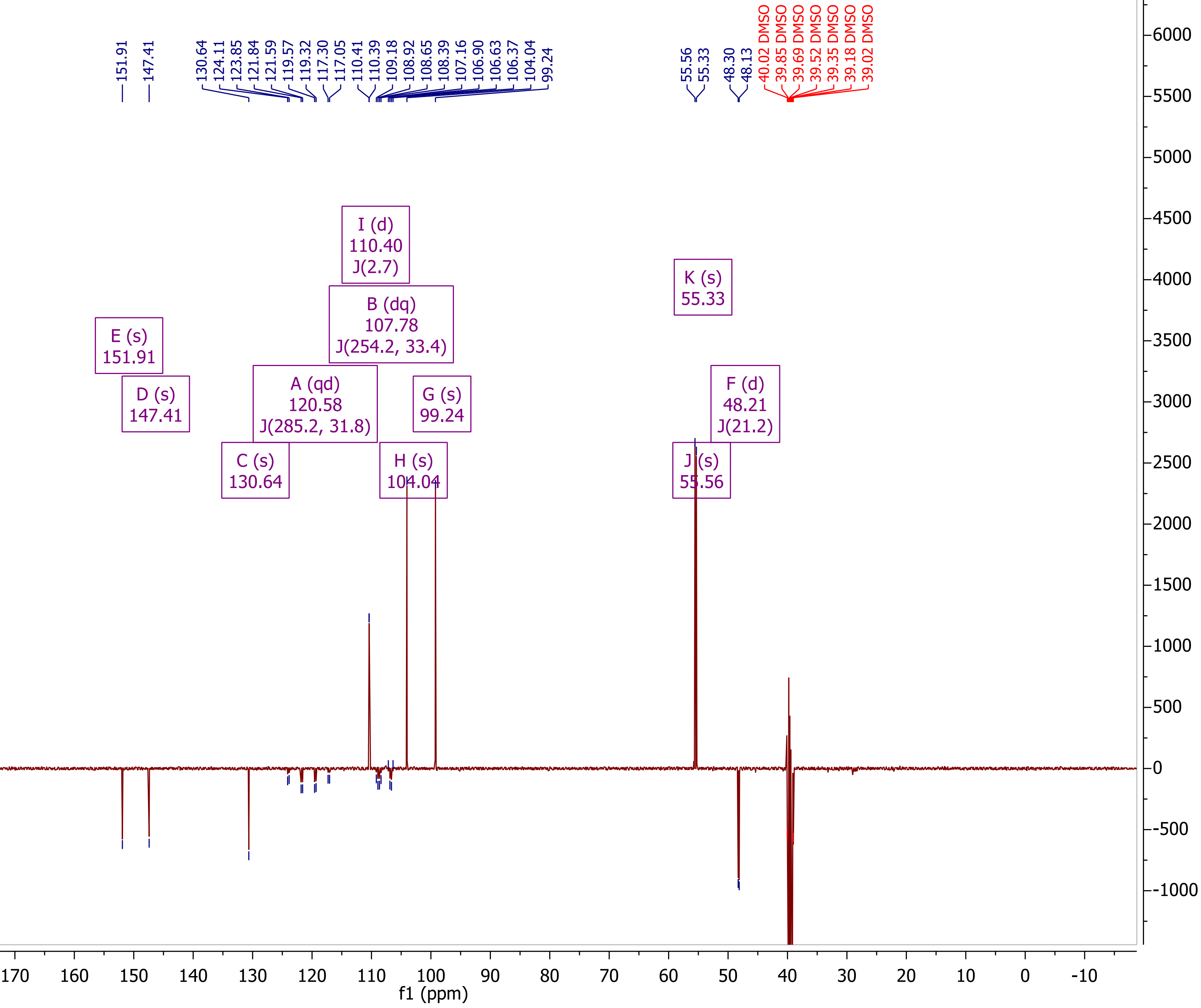

^{1}H NMR (250 MHz, Chloroform-*d*) δ 5.98 (s, 1H), 5.89 (s, 2H), 4.17 – 3.57 (m, 9H).

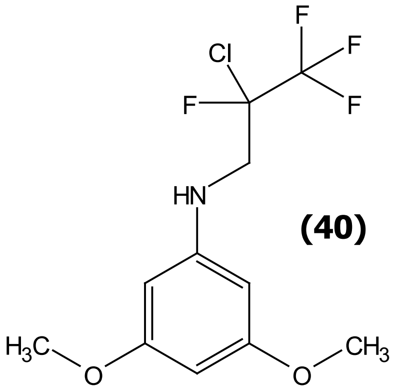

(40)

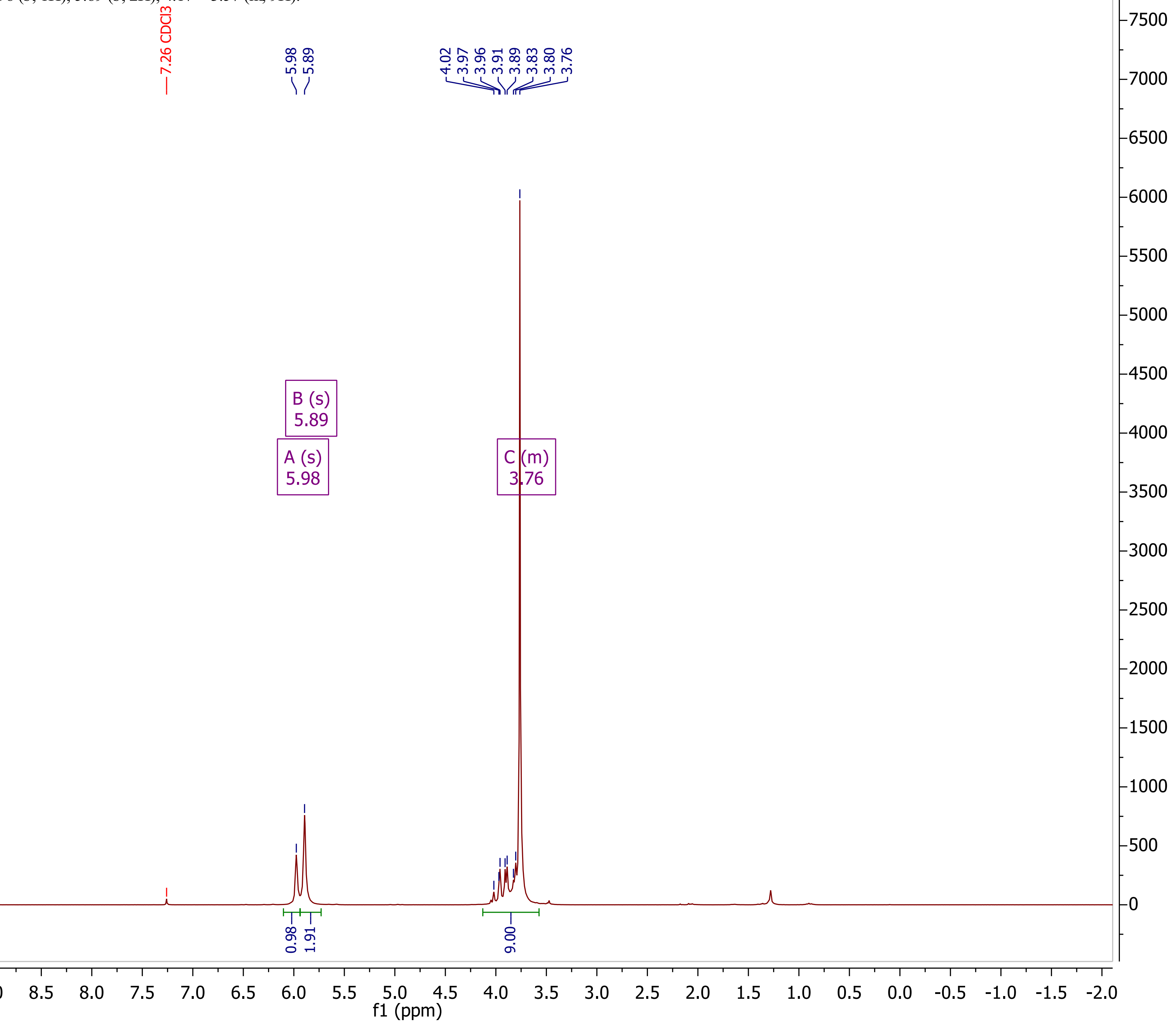

^{19}F NMR (235 MHz, Chloroform-*d*) δ -80.6 (d, *J* = 6.2 Hz), -130.1 (q, *J* = 6.1 Hz).

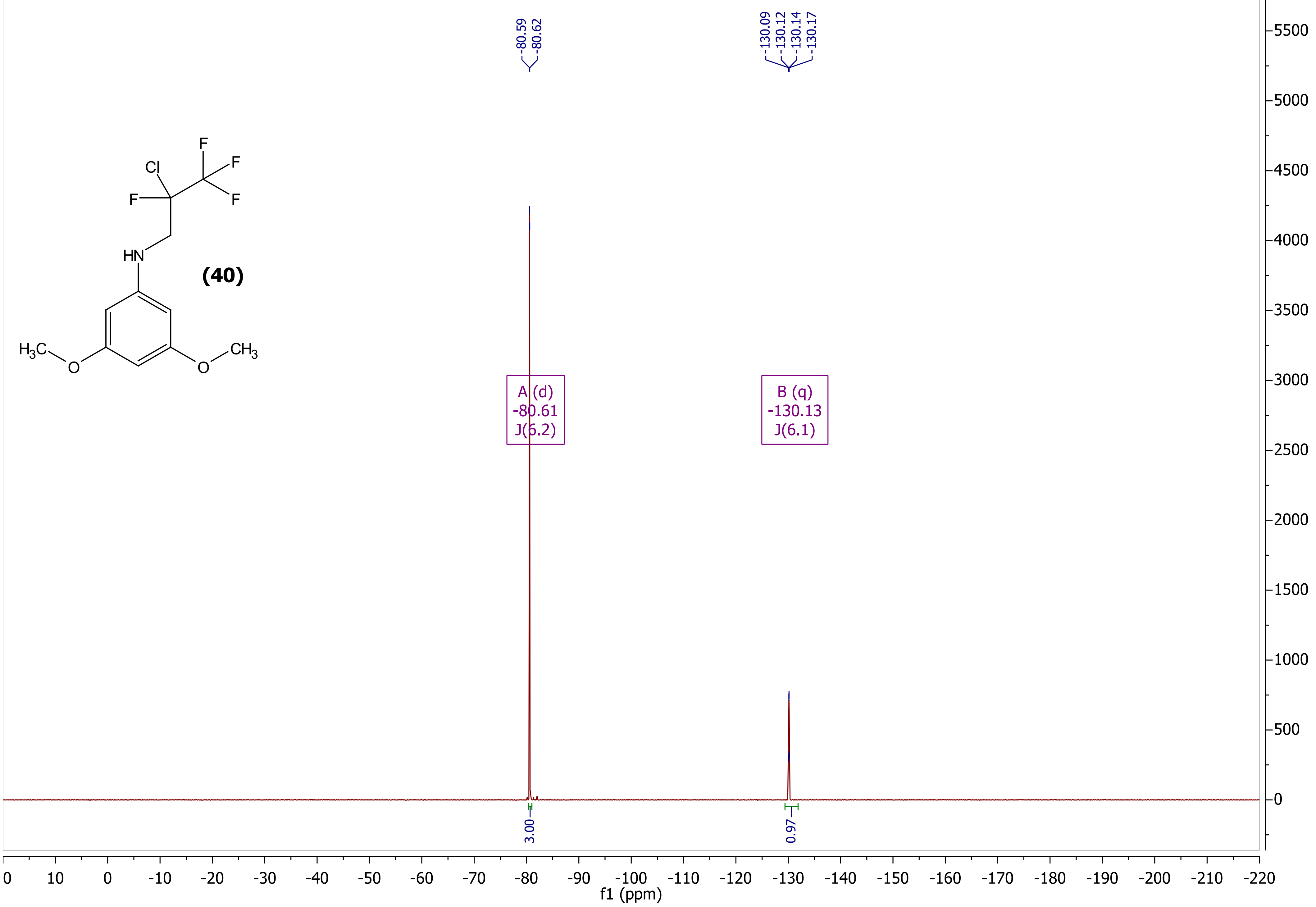

^{13}C NMR (63 MHz, Chloroform-*d*) δ 161.9, 148.5, 120.7 (qd, *J* = 284.9, 31.1 Hz), 106.8 (dq, *J* = 255.4, 34.8 Hz), 92.4, 91.2, 55.3, 48.9 (d, *J* = 22.3 Hz).

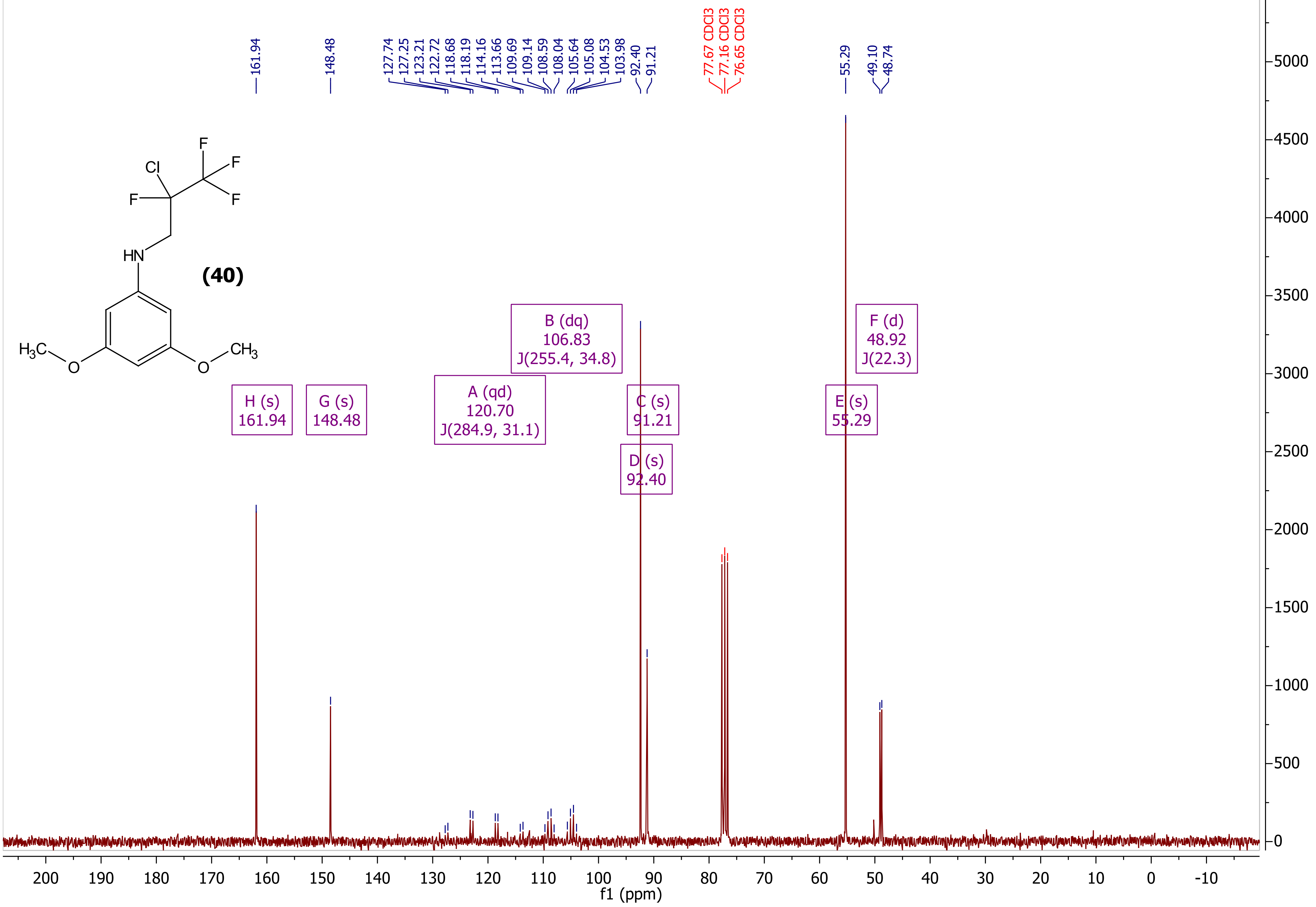

^{1}H NMR (500 MHz, DMSO-d_6) δ 7.67 (t, J = 7.9 Hz, 2H), 7.60 (d, J = 8.0 Hz, 1H), 7.33 (td, J = 8.2, 1.2 Hz, 1H), 7.16 (td, J = 6.9, 1.2 Hz, 1H), 7.12 (dd, J = 8.8, 2.4 Hz, 1H), 7.00 (d, J = 2.3 Hz, 1H), 6.60 (t, J = 7.0 Hz, 1H), 4.21 (td, J = 15.6, 6.8 Hz, 1H), 4.12 (ddd, J = 22.8, 15.9, 7.2 Hz, 1H).

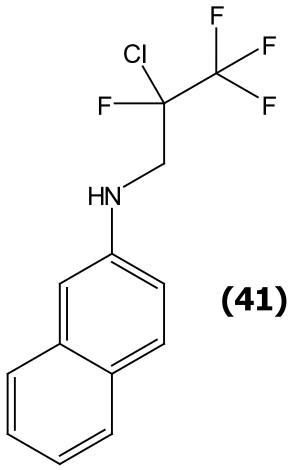

(41)

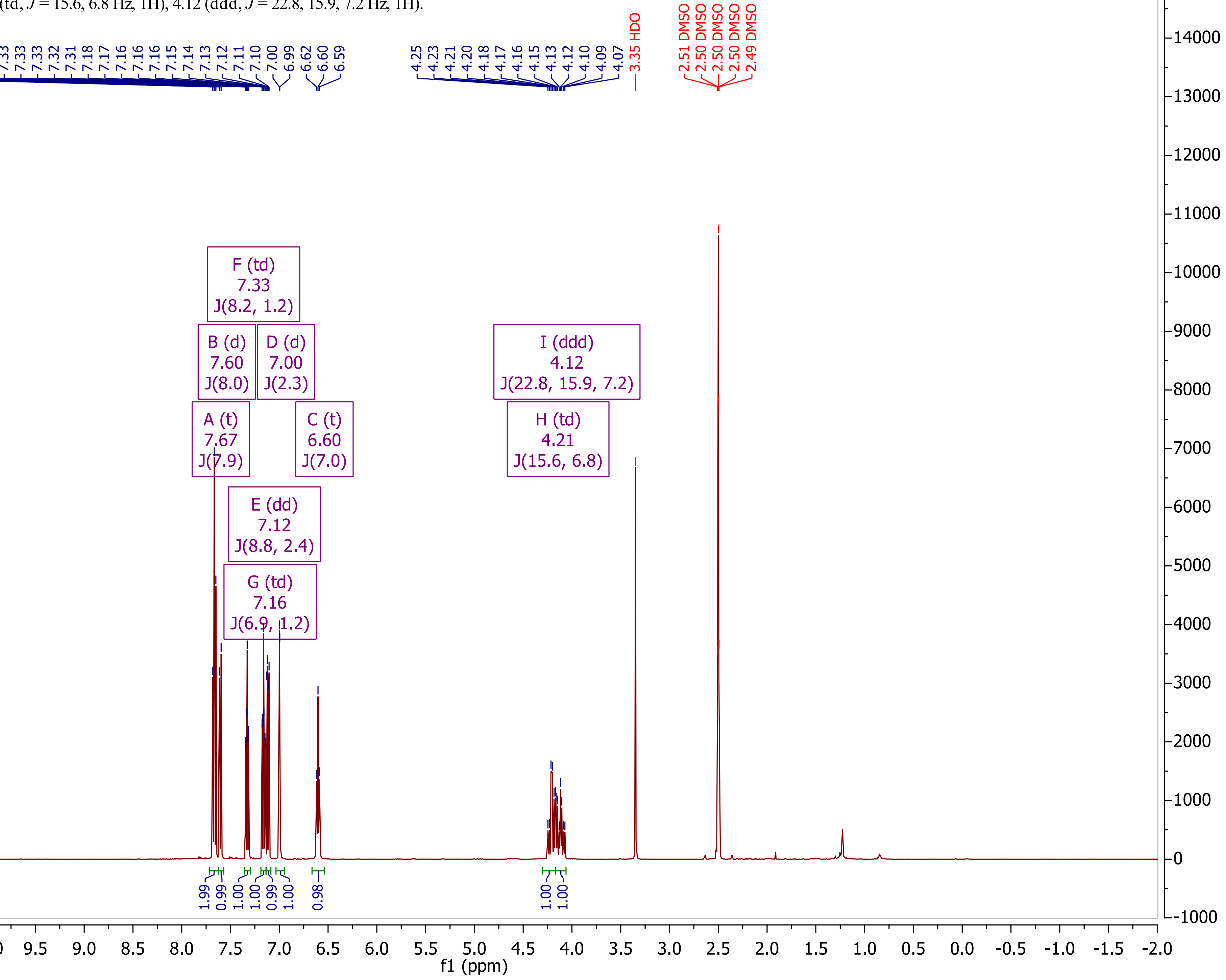

^{19}F NMR (376 MHz, DMSO-d_6) δ -79.4 (d, J = 6.4 Hz), -128.3 – -128.6 (m).

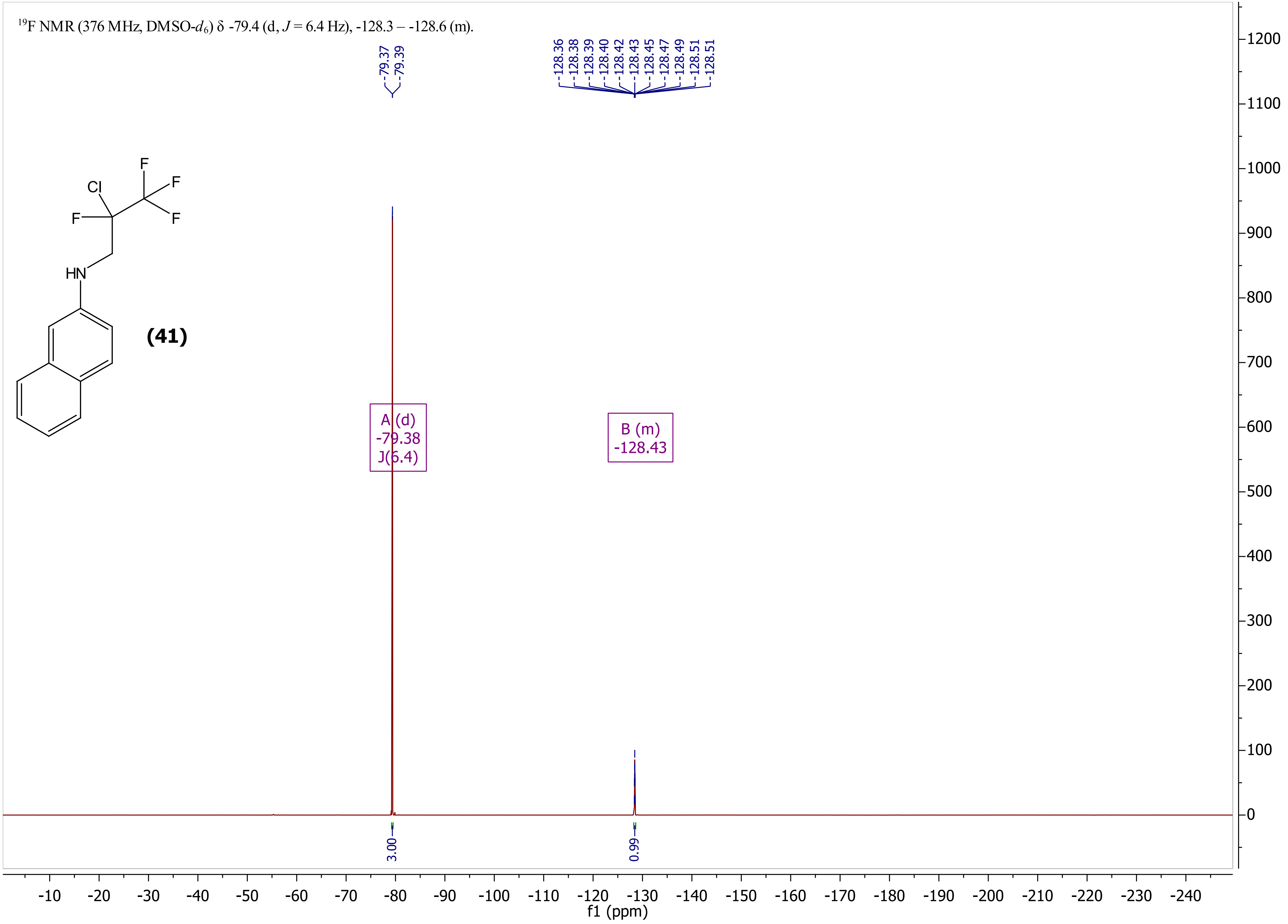

^{13}C NMR (126 MHz, DMSO-d_6) δ 145.4, 134.7, 128.5, 127.4, 127.0, 126.2, 125.7, 121.8, 120.6 (qd, J = 285.2, 31.7 Hz), 117.8, 107.5 (dq, J = 253.7, 33.6 Hz), 103.8 (d, J = 1.8 Hz), 47.5 (d, J = 21.6 Hz).

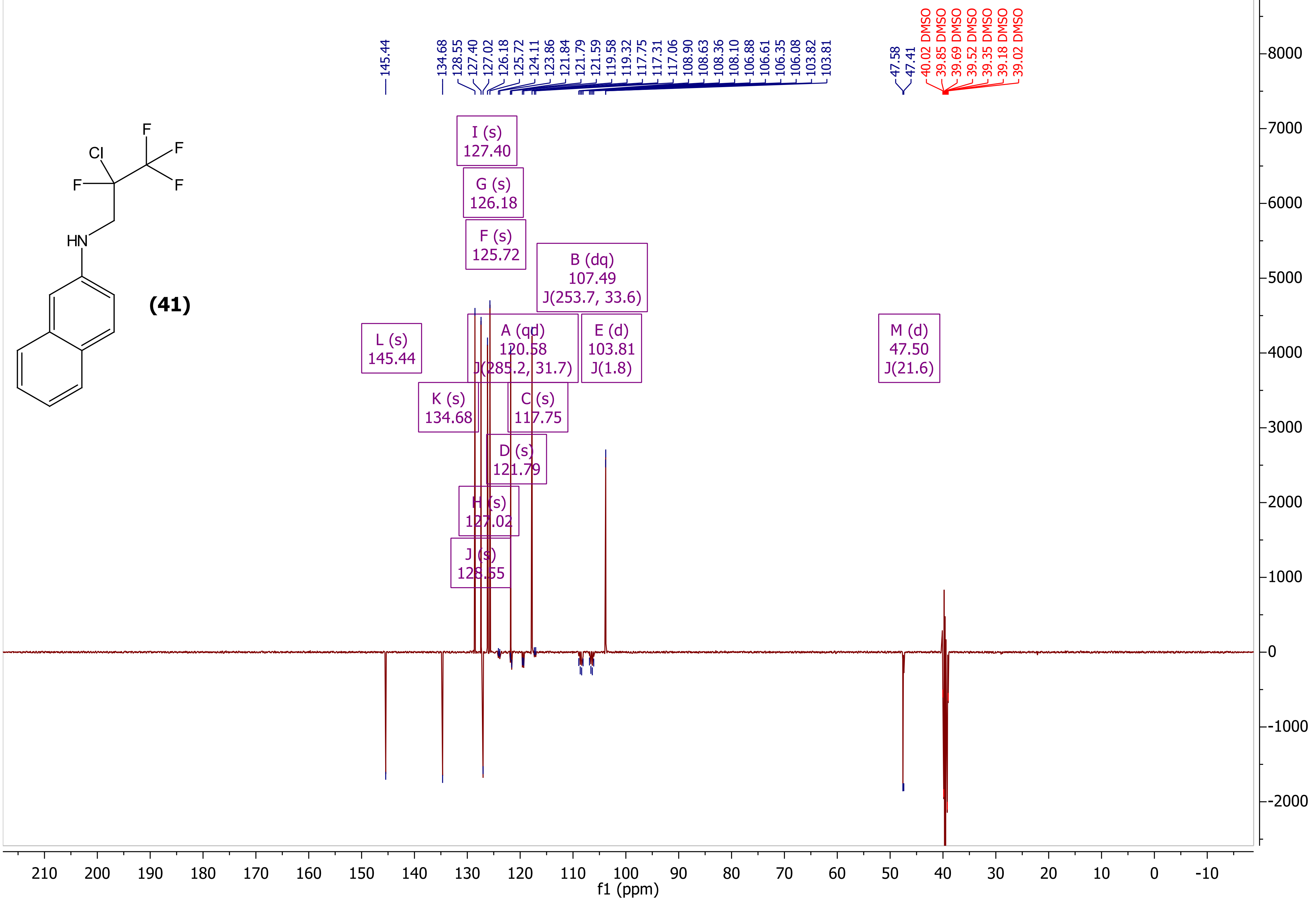

^{1}H NMR (250 MHz, Chloroform-*d*) δ 7.39 – 7.27 (m, 2H), 6.92 – 6.78 (m, 3H), 4.14 (t, *J* = 15.7 Hz, 1H), 4.04 (dd, *J* = 22.3, 16.3 Hz, 1H), 3.12 (s, 3H).

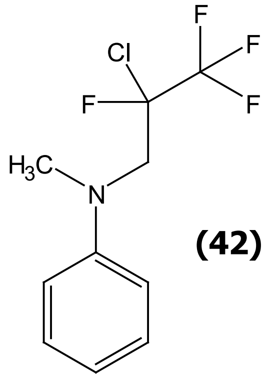

(42)

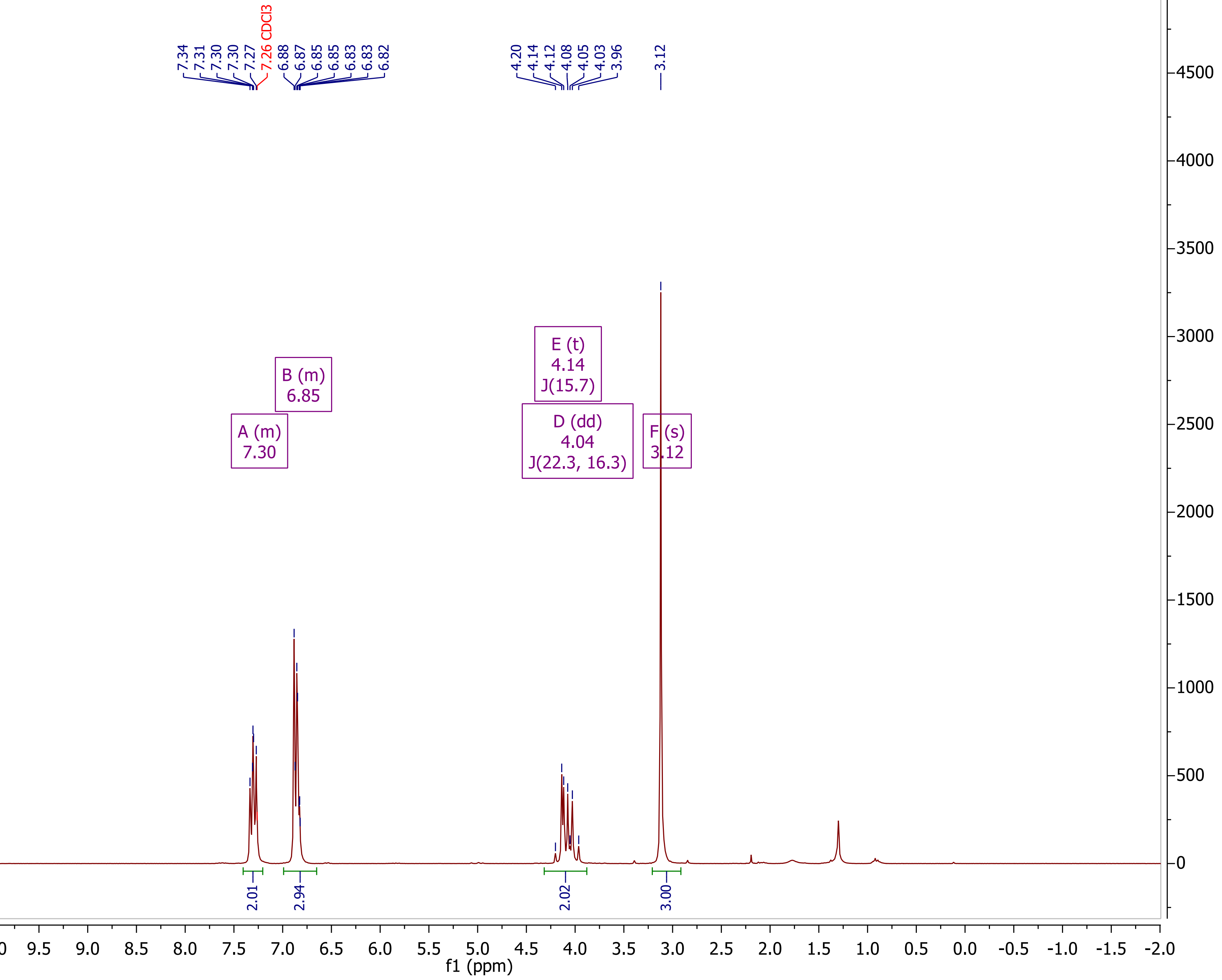

^{19}F NMR (235 MHz, Chloroform-*d*) δ -80.8 (d, *J* = 6.0 Hz), -127.1 (q, *J* = 6.1 Hz).

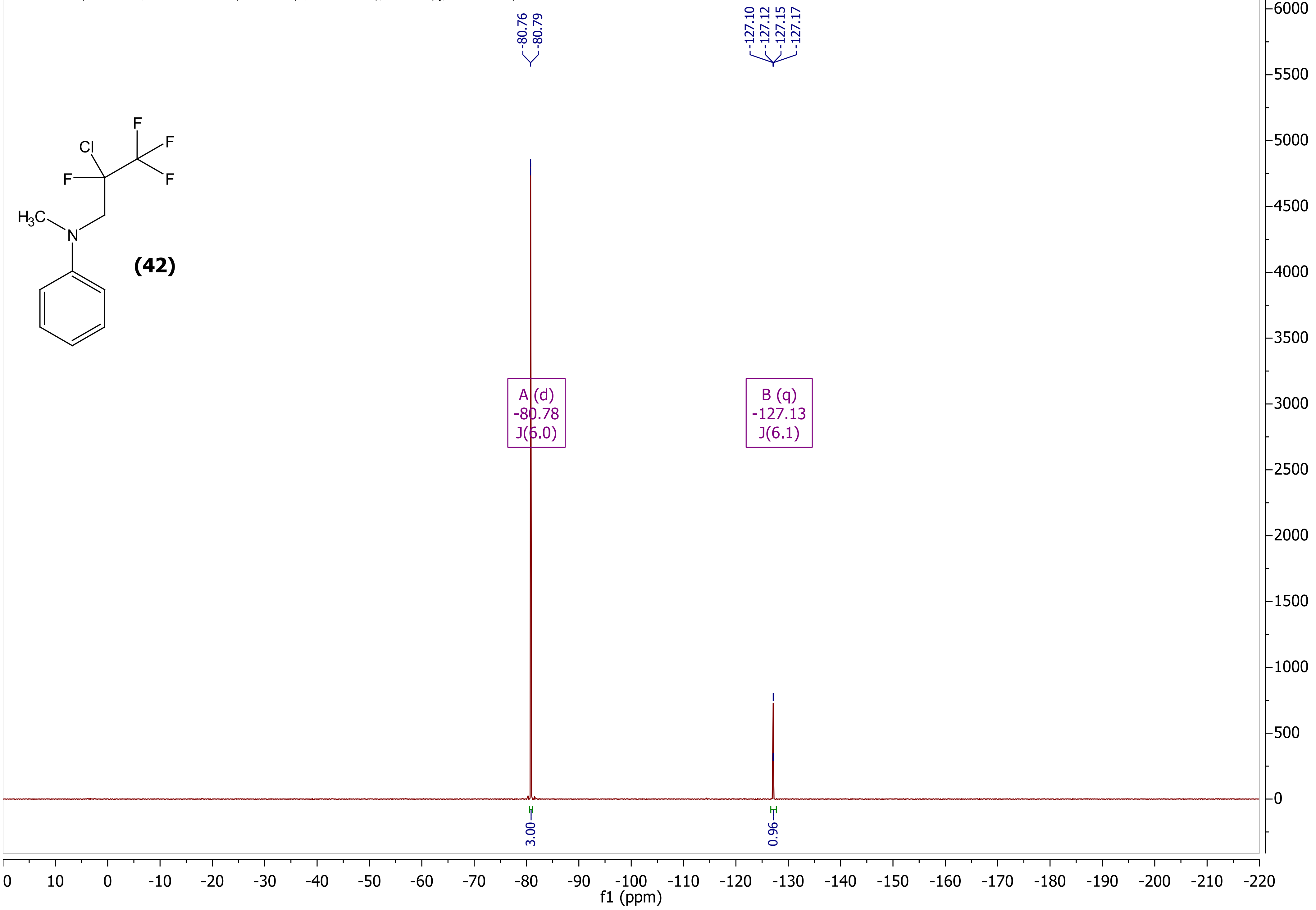

^{13}C NMR (63 MHz, Chloroform-*d*) δ 149.0, 129.3, 120.8 (qd, *J* = 285.1, 31.5 Hz), 118.4, 112.9 (d, *J* = 1.5 Hz), 107.9 (dq, *J* = 256.6, 34.2 Hz), 57.4 (d, *J* = 20.3 Hz), 40.1 (d, *J* = 2.0 Hz).

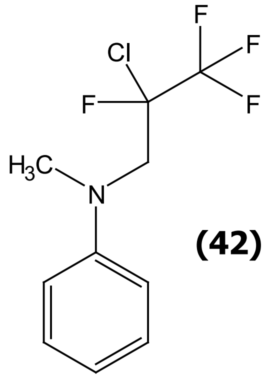

(42)

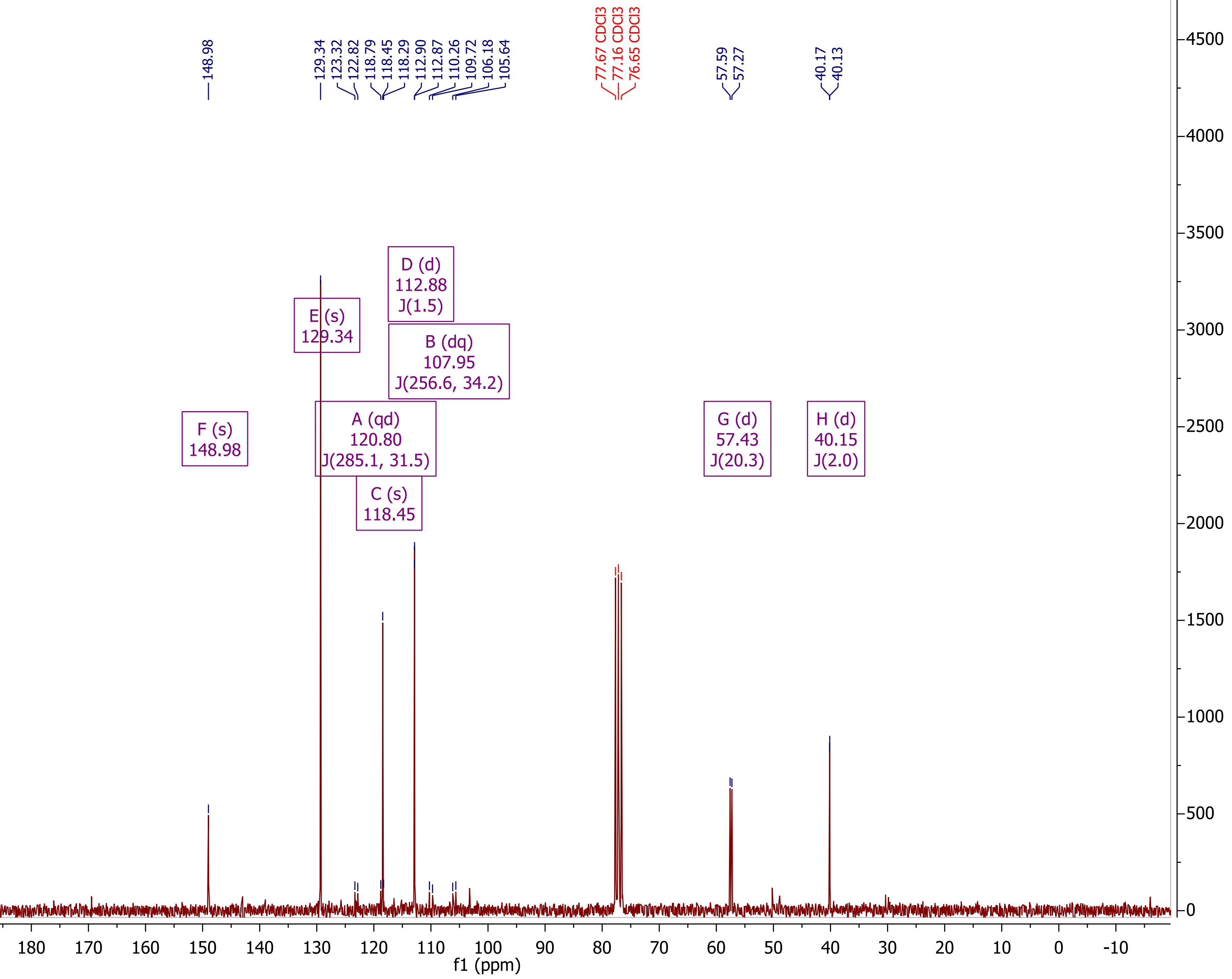

^{1}H NMR (250 MHz, Chloroform-*d*) δ 7.40 – 7.16 (m, 2H), 6.97 – 6.76 (m, 3H), 4.10 (t, *J* = 15.5 Hz, 1H), 4.00 (dd, *J* = 23.5, 16.3 Hz, 1H), 3.57 (q, *J* = 7.0 Hz, 2H), 1.22 (t, *J* = 7.0 Hz, 3H).

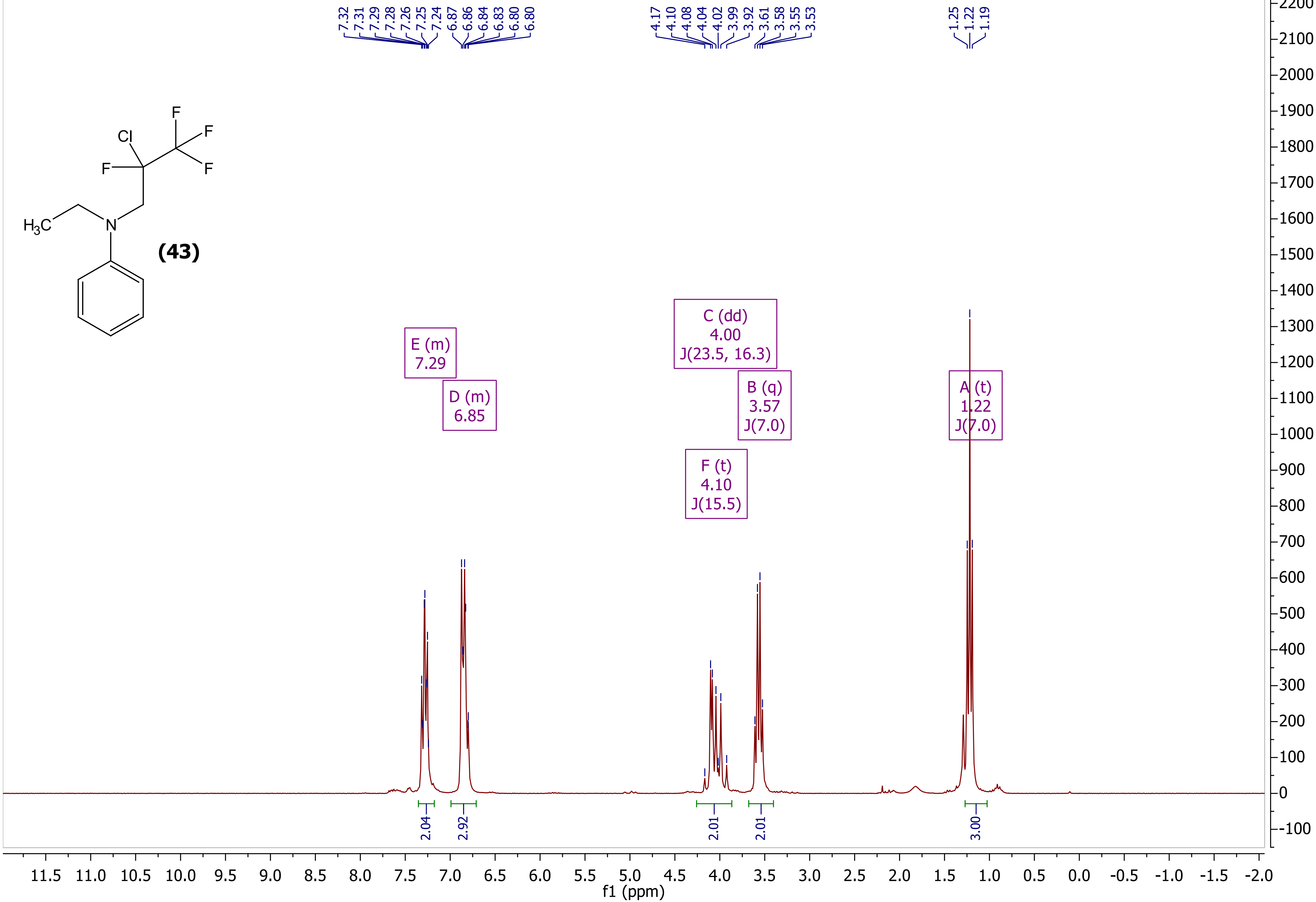

^{19}F NMR (235 MHz, Chloroform-*d*) δ -80.8 (d, *J* = 6.1 Hz), -126.8 (q, *J* = 6.1 Hz).

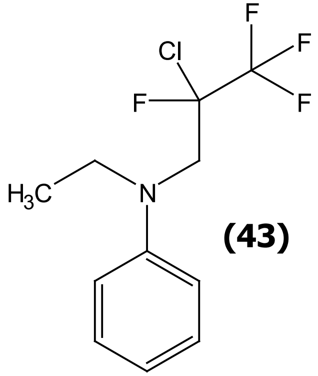

(43)

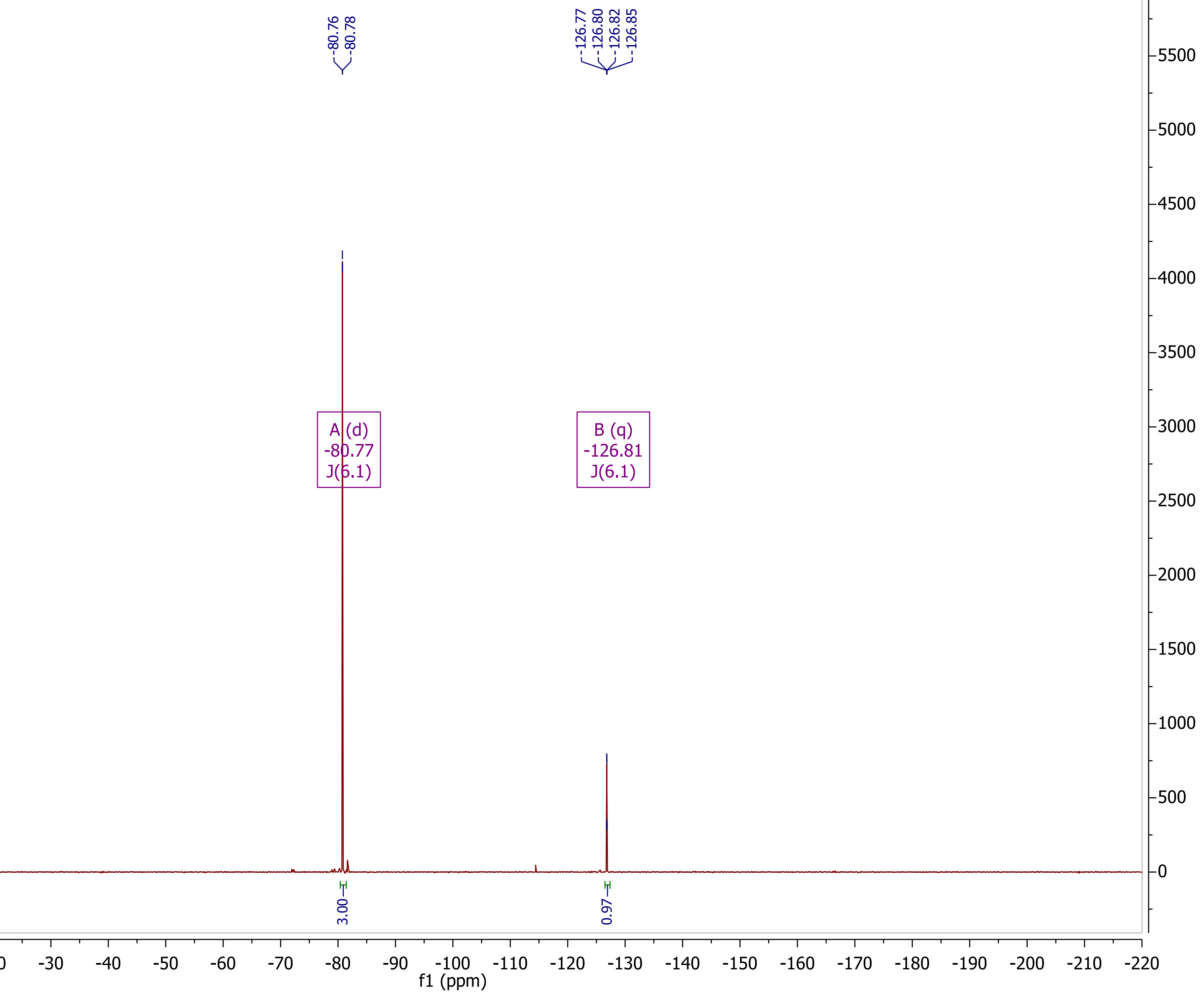

^{13}C NMR (63 MHz, Chloroform-*d*) δ 147.5, 129.4, 120.9 (qd, *J* = 284.6, 31.8 Hz), 118.3, 113.6 (d, *J* = 1.6 Hz), 108.0 (dq, *J* = 256.1, 34.0 Hz), 55.5 (d, *J* = 20.3 Hz), 46.4 (d, *J* = 1.7 Hz), 11.4.

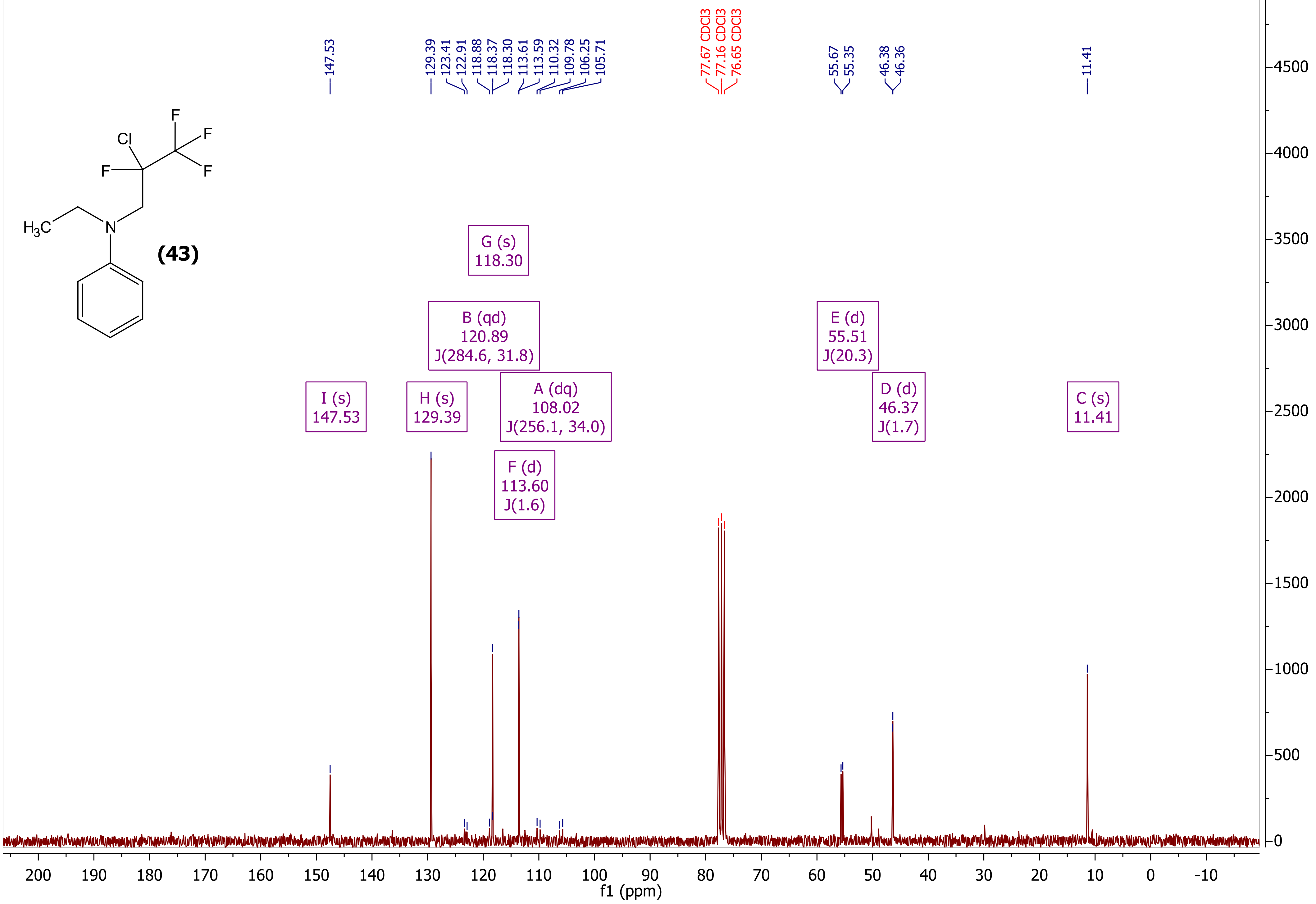

^{1}H NMR (250 MHz, Chloroform-*d*) δ 7.12 (t, *J* = 7.5 Hz, 2H), 6.77 (t, *J* = 7.3 Hz, 1H), 6.56 (d, *J* = 7.9 Hz, 1H), 3.94 – 3.70 (m, 2H), 3.64 (t, *J* = 9.0 Hz, 2H), 3.10 (t, *J* = 8.5 Hz, 2H).

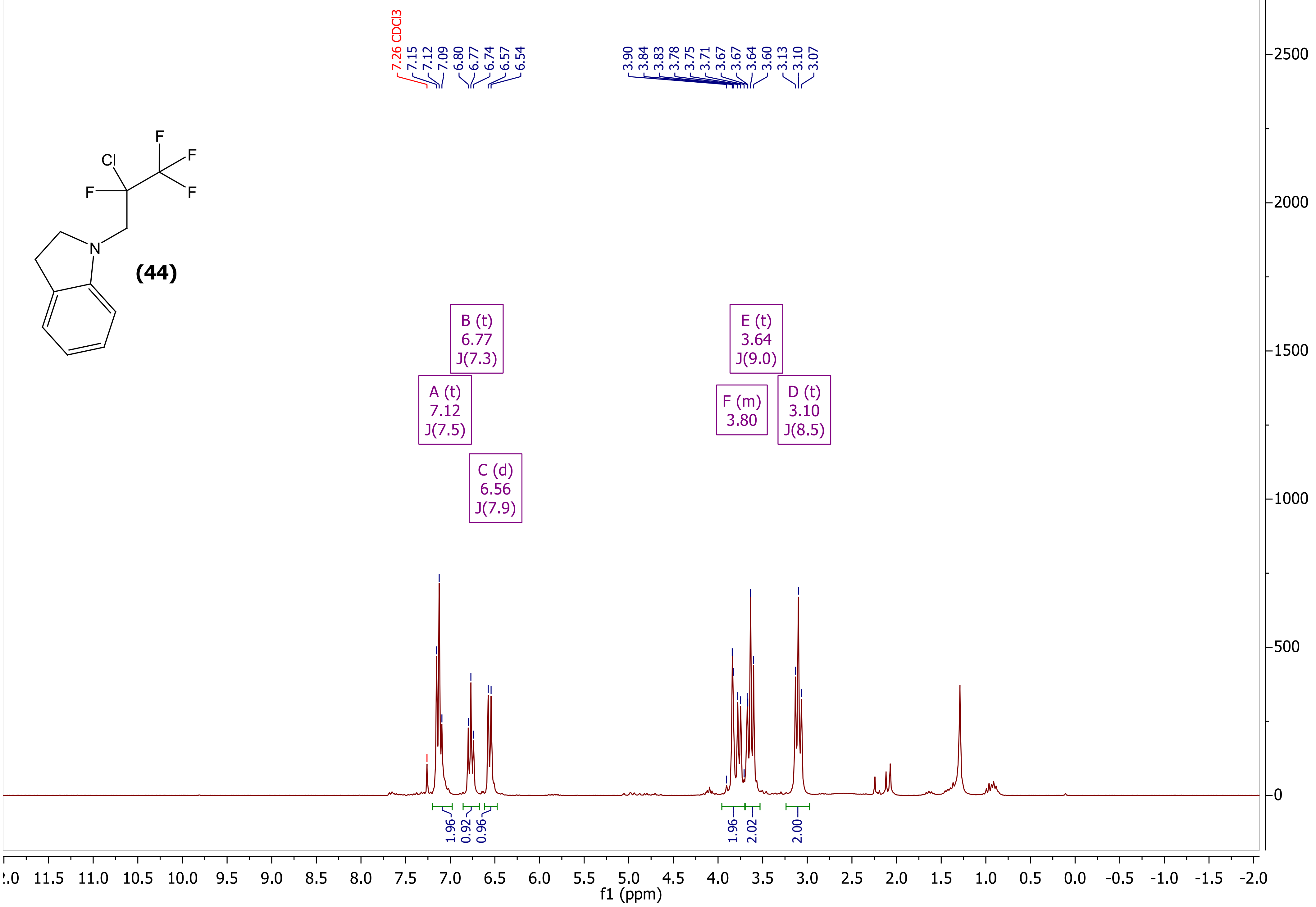

^{19}F NMR (235 MHz, Chloroform-*d*) δ -80.4 (d, *J* = 6.1 Hz), -128.5 (q, *J* = 6.0 Hz).

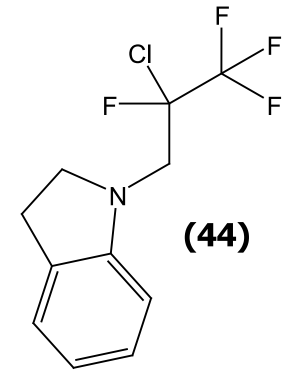

(44)

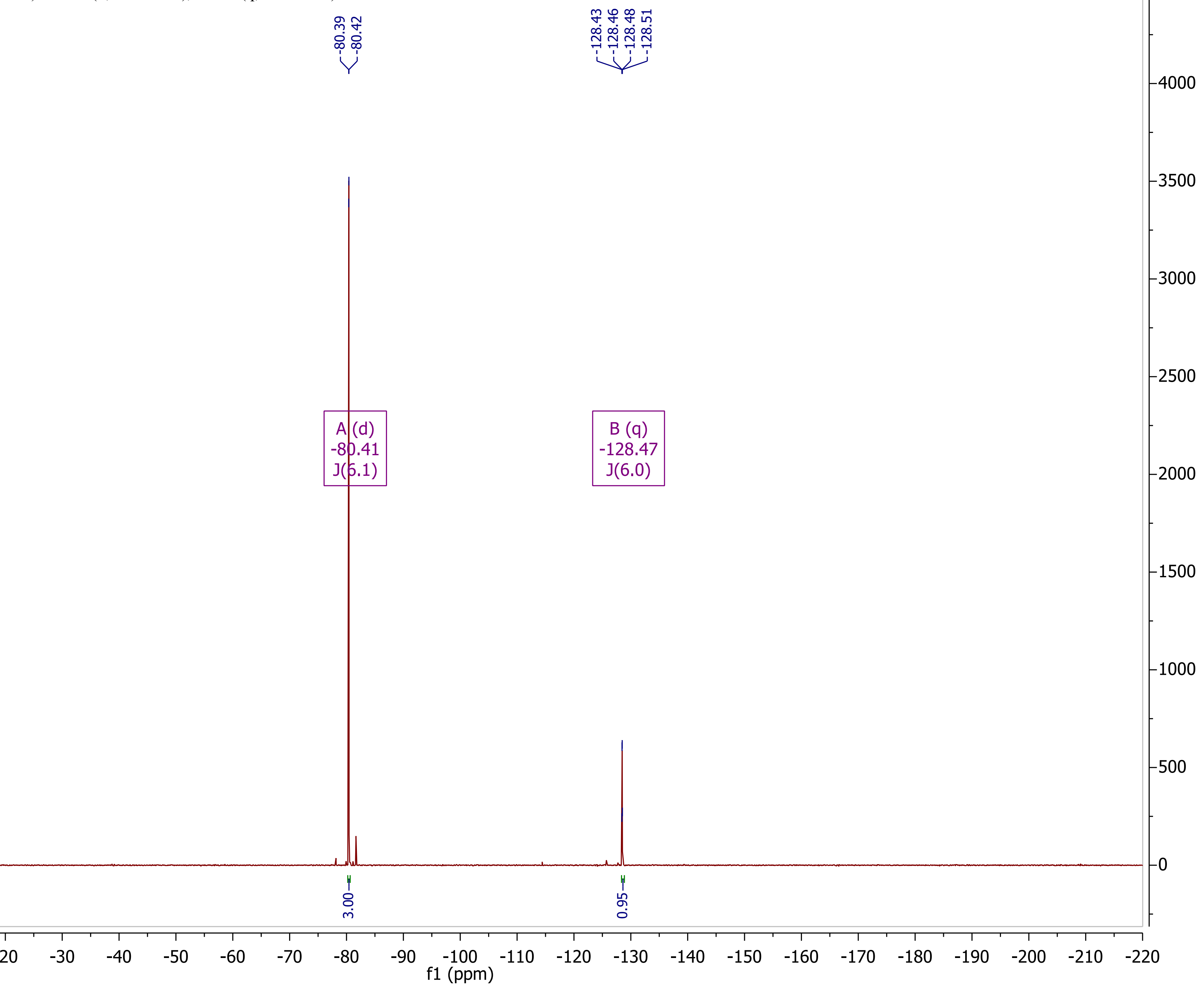

^{13}C NMR (63 MHz, Chloroform-*d*) δ 151.4, 129.1, 127.6, 124.8, 120.8 (qd, *J* = 285.0, 31.2 Hz), 119.0, 107.4 (dq, *J* = 254.3, 34.1 Hz), 106.8 (d, *J* = 1.5 Hz), 56.4 (d, *J* = 22.1 Hz), 55.6, 28.9.

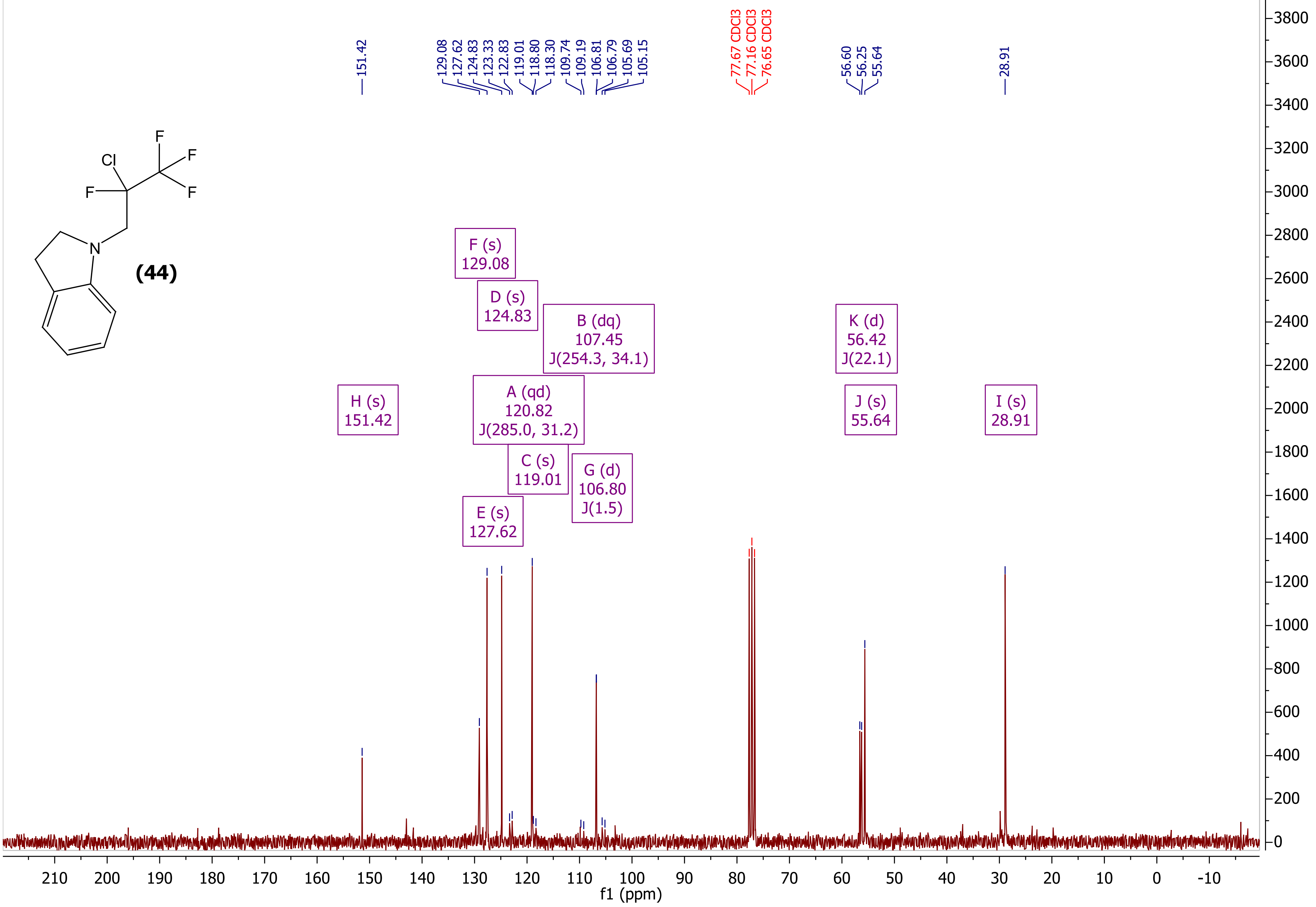

^{1}H NMR (500 MHz, DMSO-d_6) δ 7.97 (bs, 1H), 7.71 (bs, 1H), 4.82 (t, J = 7.4 Hz, 1H), 3.90 (s, 3H), 3.76 (ddd, J = 14.8, 12.3, 7.0 Hz, 1H), 3.67 (ddd, J = 22.7, 14.8, 7.4 Hz, 1H), 2.49 (t, J = 2.3 Hz, 2H), 1.68 – 1.54 (m, 2H), 0.92 (t, J = 7.4 Hz, 3H).

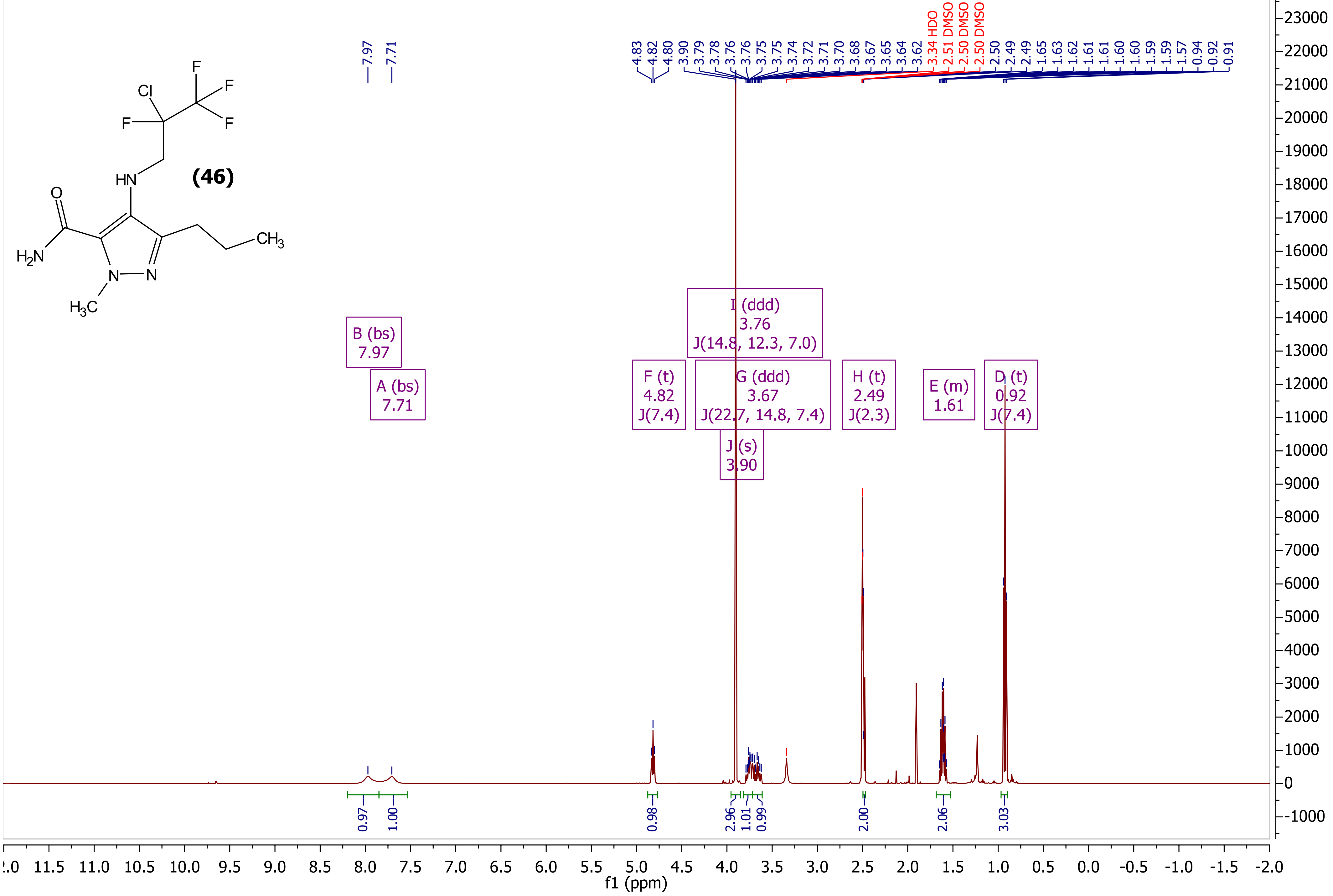

^{19}F NMR (376 MHz, DMSO-d_6) δ -79.8 (d, J = 6.5 Hz), -130.0 – -130.3 (m).

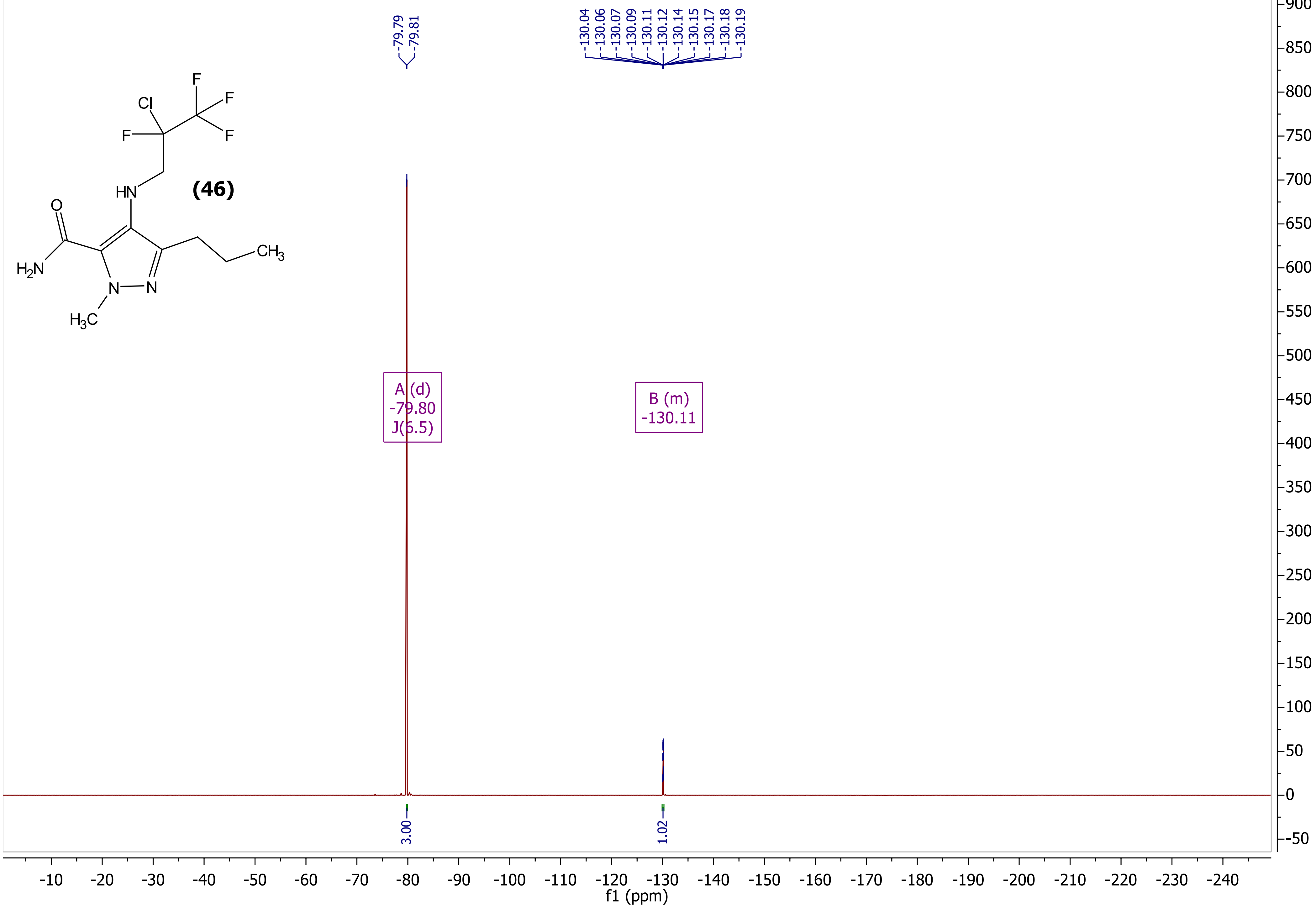

^{13}C NMR (126 MHz, DMSO-d_6) δ 161.2, 145.6, 129.4, 127.5, 120.4 (qd, J = 284.9, 31.2 Hz), 106.9 (dq, J = 253.1, 34.1 Hz), 52.8 (d, J = 20.5 Hz), 39.2, 27.3, 21.5, 14.0.

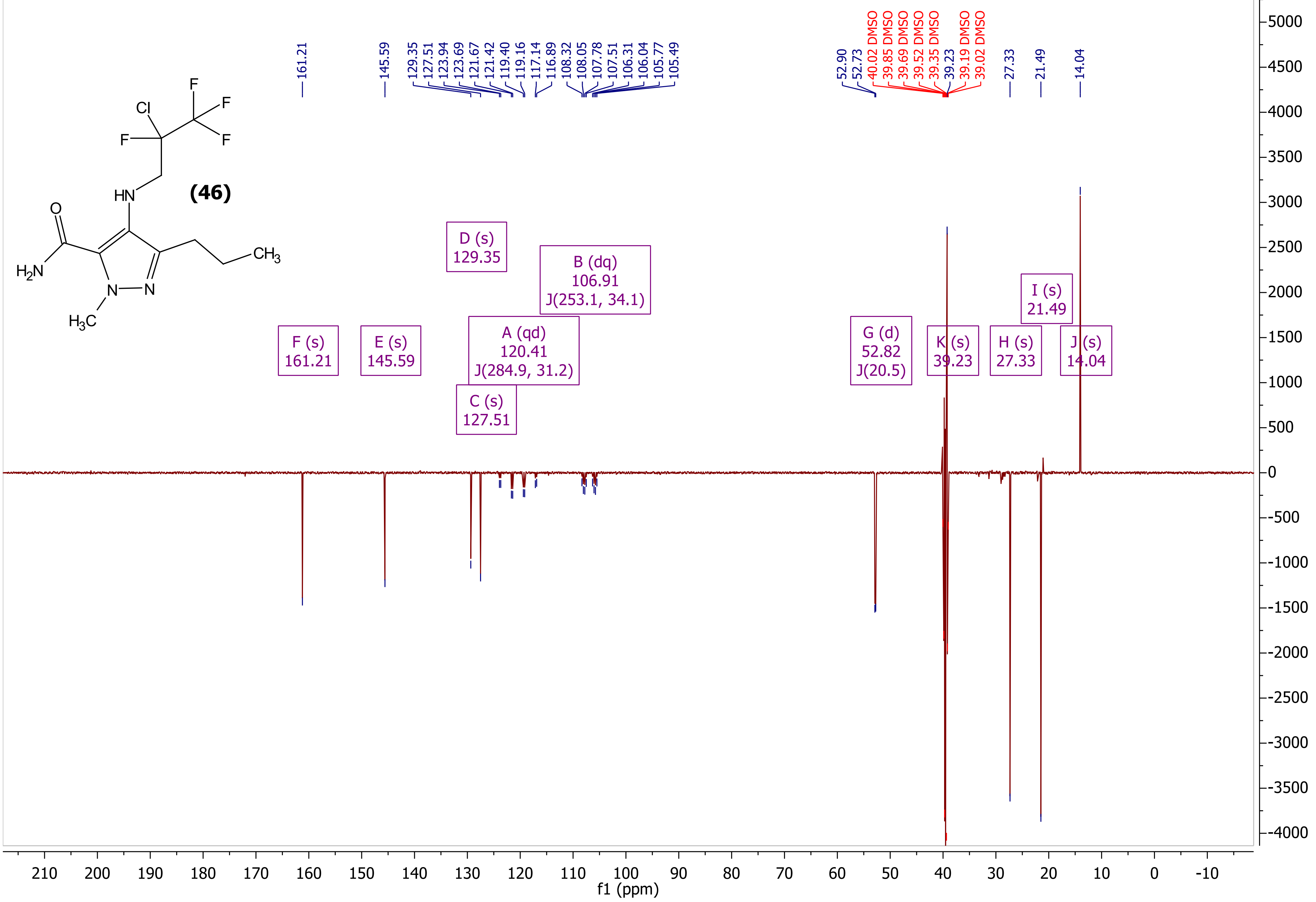

^{1}H NMR (250 MHz, Chloroform-*d*) δ 8.77 (dd, *J* = 4.2, 1.7 Hz, 1H), 8.08 (dd, *J* = 8.3, 1.7 Hz, 1H), 7.50 – 7.31 (m, 2H), 7.17 (dd, *J* = 8.2, 1.2 Hz, 1H), 6.87 (d, *J* = 7.7 Hz, 1H), 6.79 (s, 1H), 4.23 (ddd, *J* = 13.9, 12.6, 6.6 Hz, 1H), 4.09 (ddd, *J* = 22.0, 15.3, 7.0 Hz, 1H).

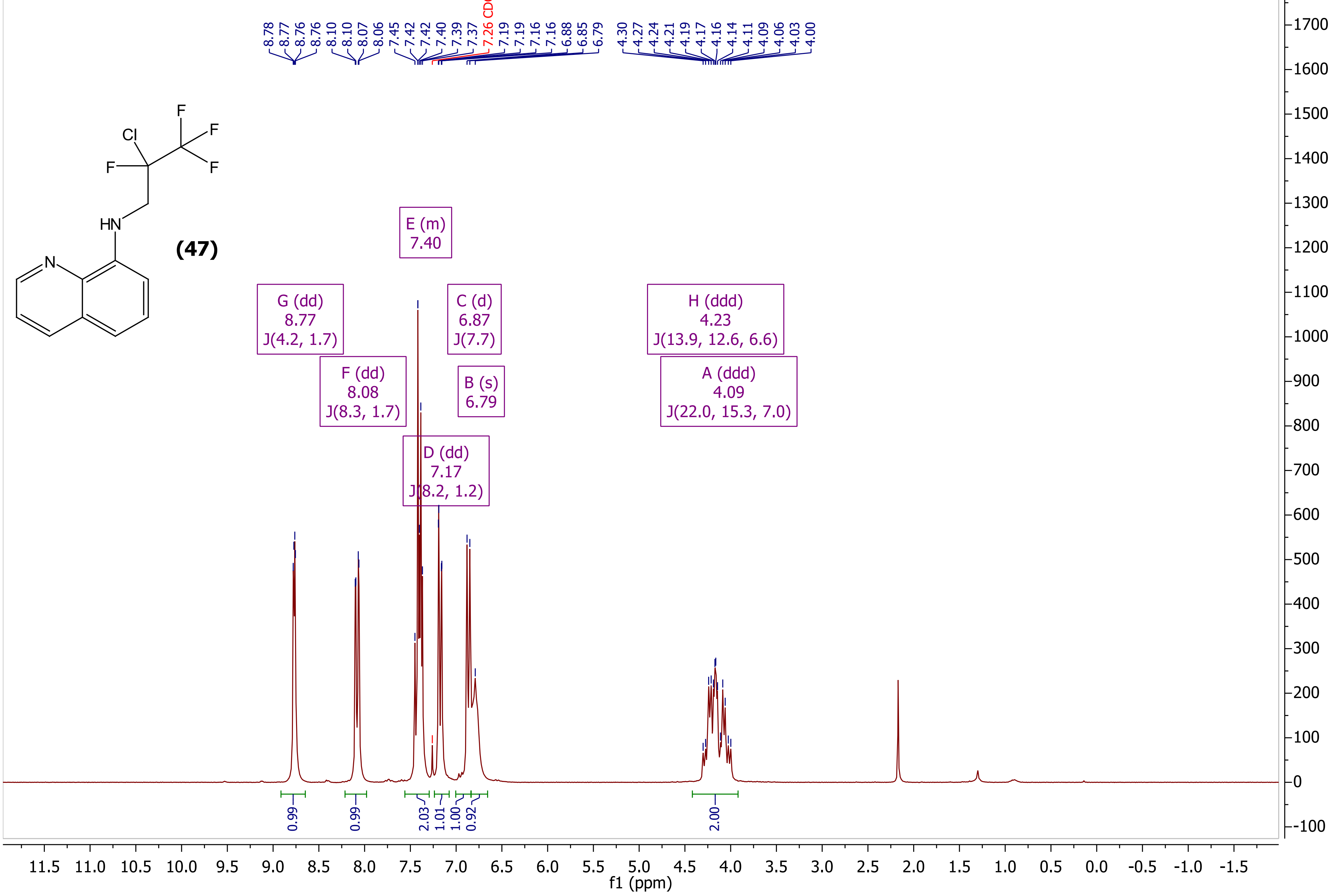

^{19}F NMR (235 MHz, Chloroform-*d*) δ -80.5 (d, *J* = 6.1 Hz), -129.7 (q, *J* = 6.1 Hz).

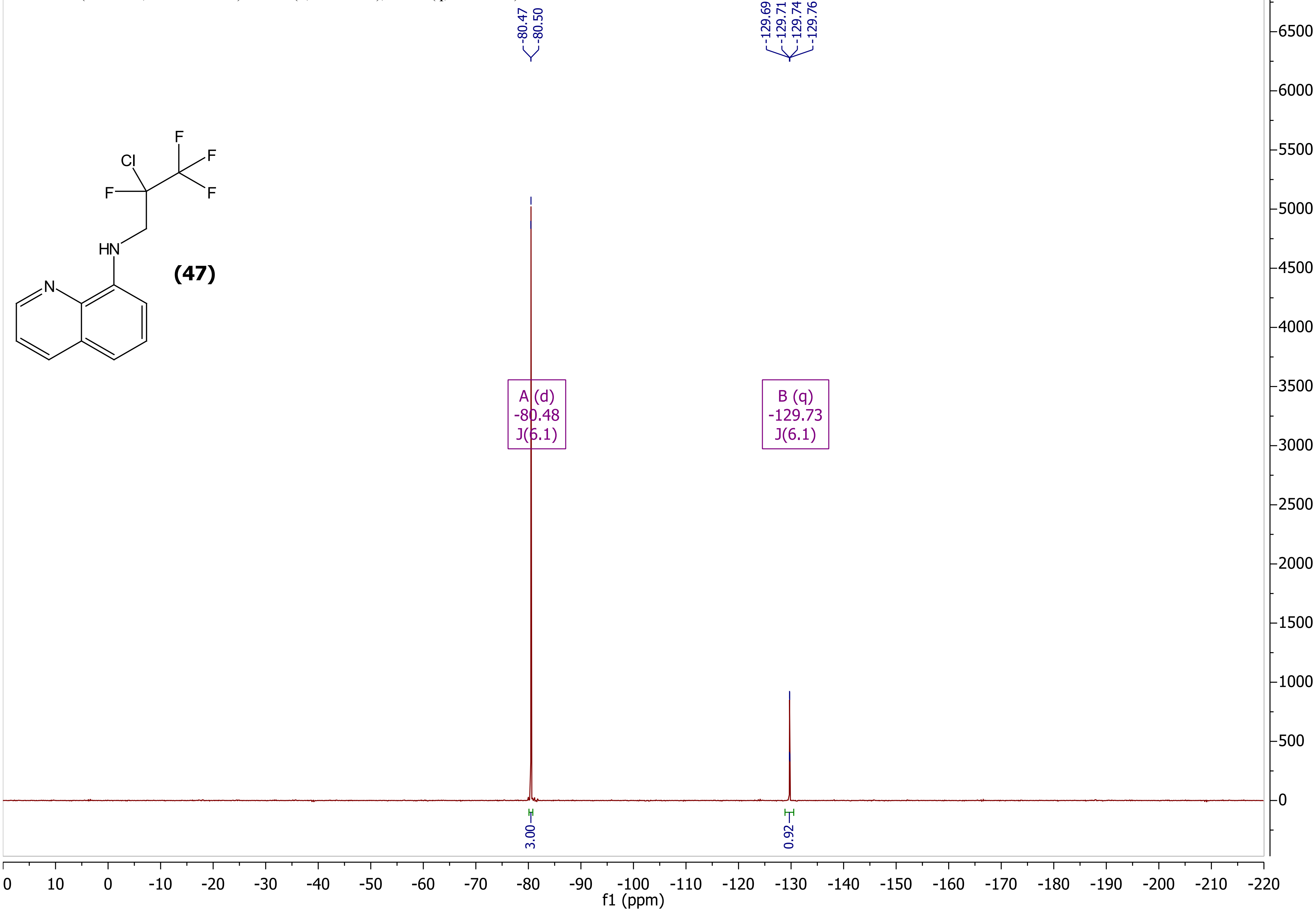

^{13}C NMR (63 MHz, Chloroform-*d*) δ 147.5, 143.3, 138.2, 136.2, 128.7, 127.5, 121.8, 120.9 (qd, *J* = 284.9, 31.0 Hz), 116.0, 107.2 (dq, *J* = 255.8, 34.6 Hz), 105.5 (d, *J* = 2.2 Hz), 48.7 (d, *J* = 22.4 Hz).

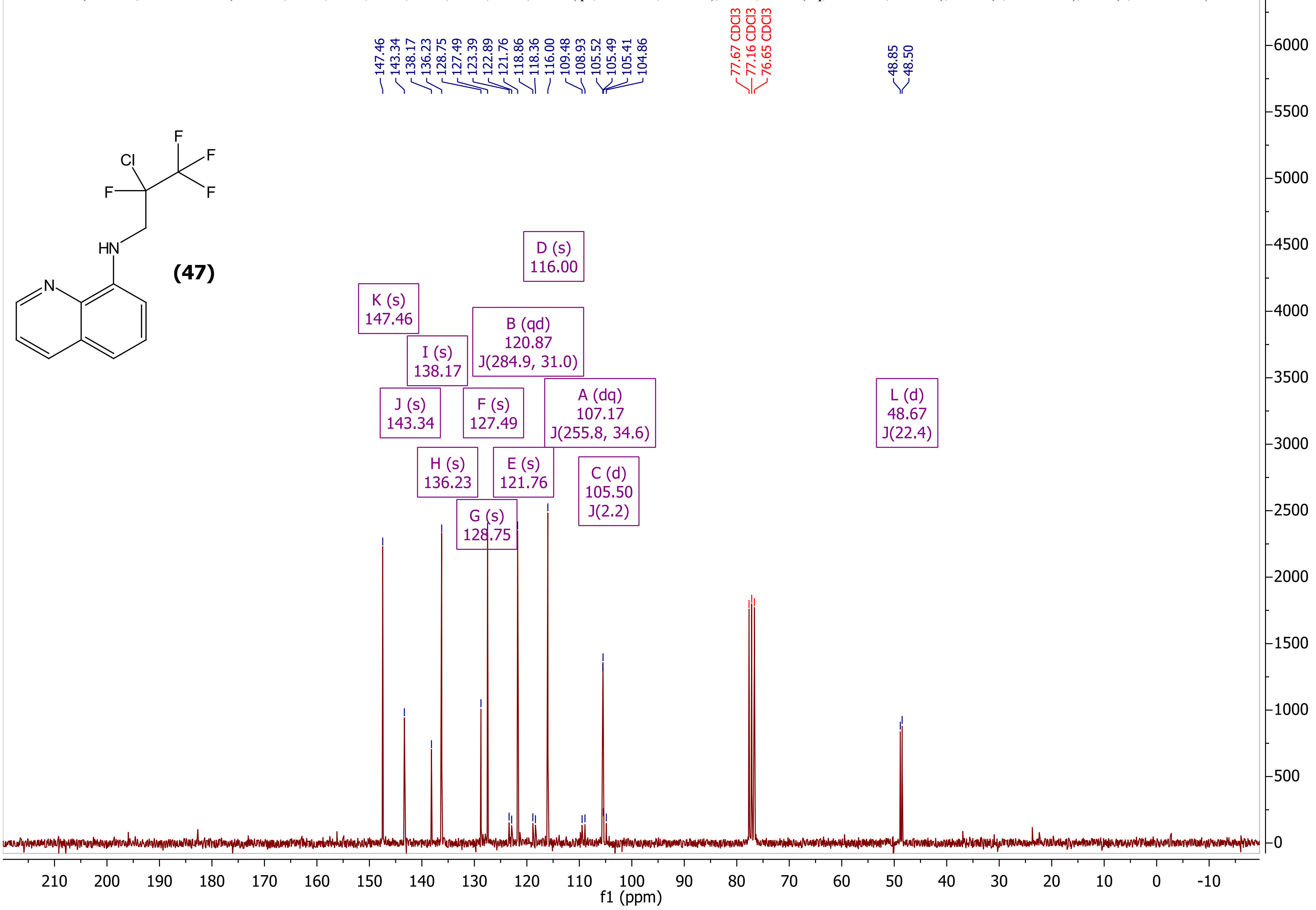

^{1}H NMR (250 MHz, Chloroform-*d*) δ 7.39 (t, *J* = 7.9 Hz, 1H), 6.68 (d, *J* = 7.6 Hz, 1H), 6.41 (d, *J* = 8.2 Hz, 1H), 4.97 (bs, 1H), 4.41 – 3.99 (m, 2H).

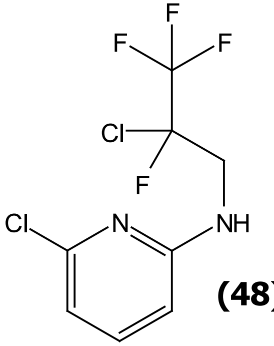

(48)

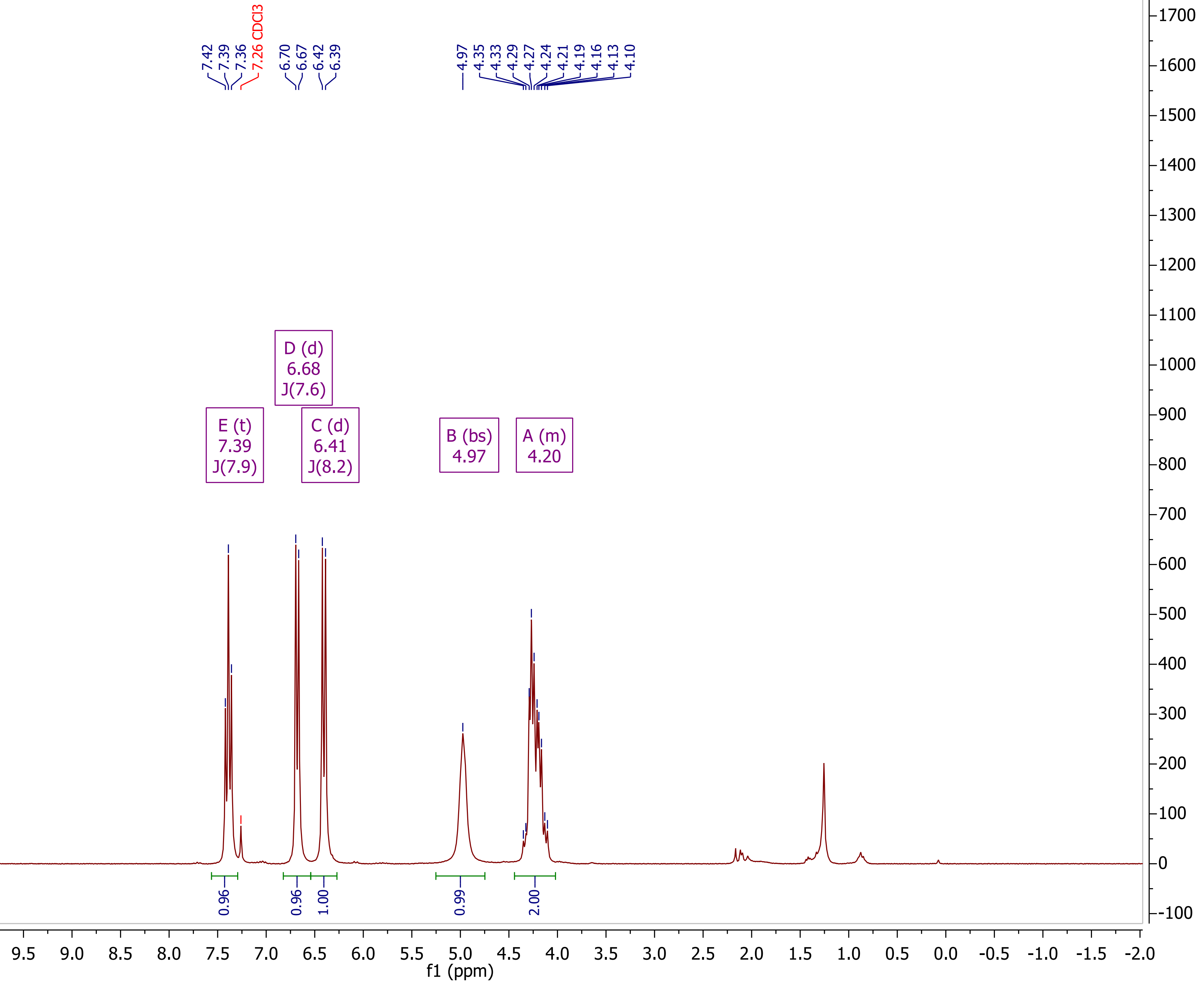

^{19}F NMR (235 MHz, Chloroform-*d*) δ -80.6 (d, *J* = 6.4 Hz), -130.4 (q, *J* = 6.4 Hz).

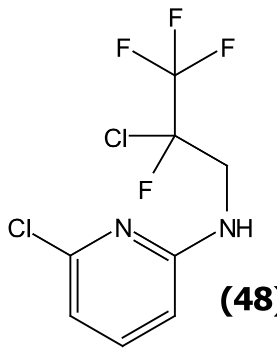

(48)

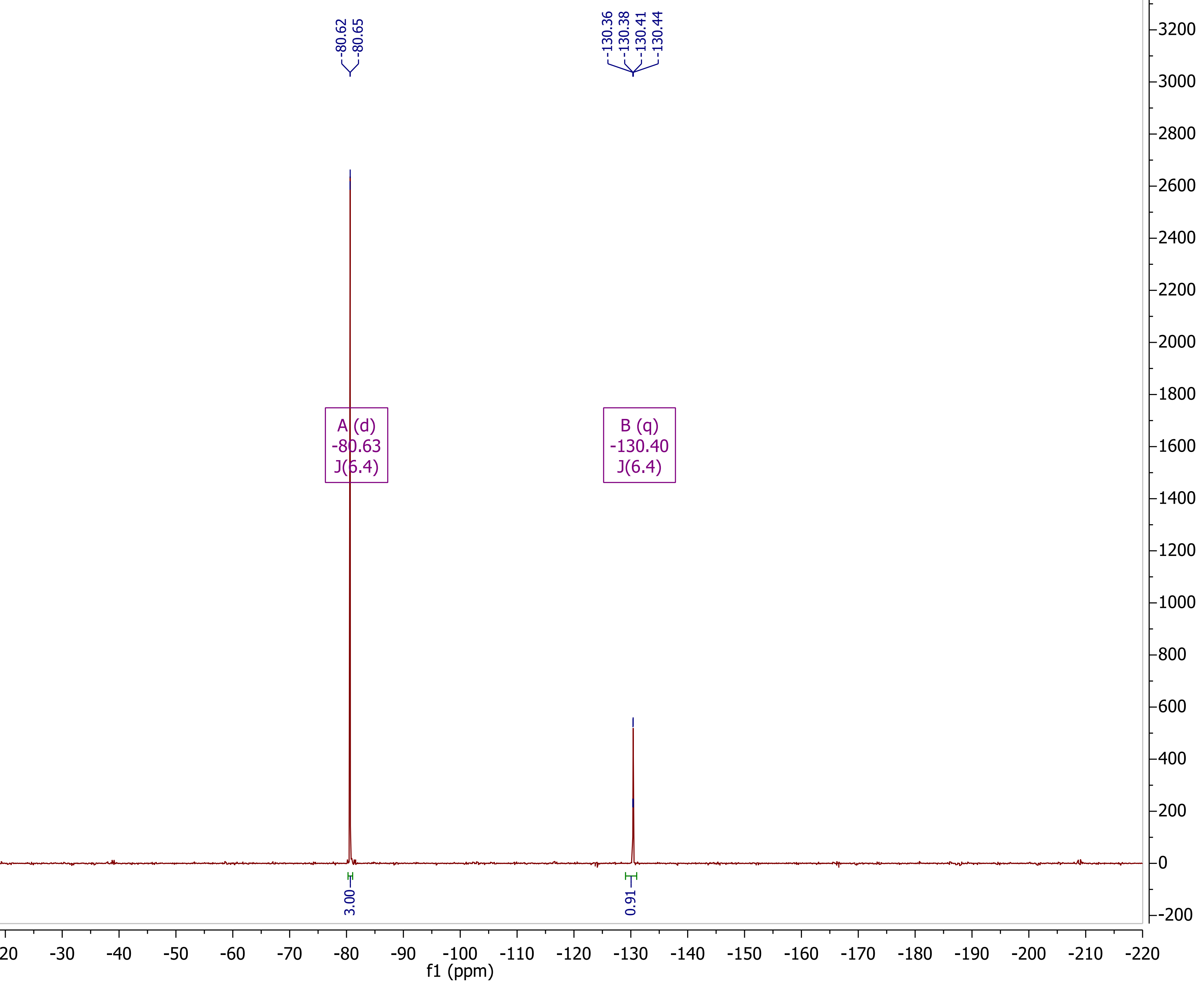

^{13}C NMR (63 MHz, Chloroform-*d*) δ 157.2, 149.6, 140.1, 120.6 (qd, *J* = 284.7, 30.9 Hz), 114.0, 106.4 (dq, *J* = 254.6, 35.3 Hz), 106.0, 45.9 (d, *J* = 22.4 Hz).

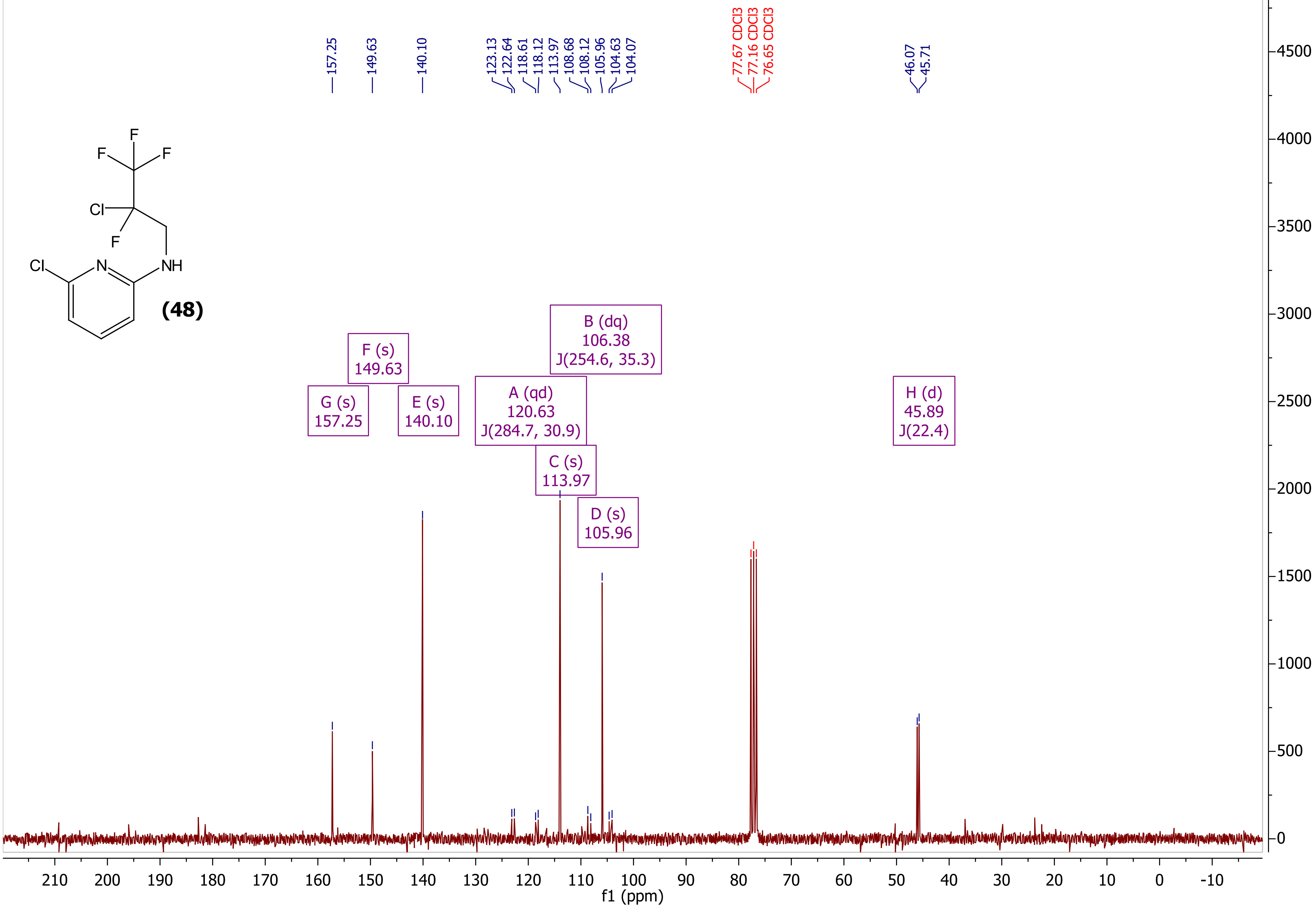

^{1}H NMR (250 MHz, Chloroform-*d*) δ 10.21 (bs, 1H), 7.95 (s, 1H), 7.59 – 7.42 (m, 1H), 6.87 – 6.37 (m, 2H), 4.59 (bs, 1H), 4.00 (t, *J* = 14.0 Hz, 1H), 3.85 (dd, *J* = 21.0, 15.2 Hz, 1H).

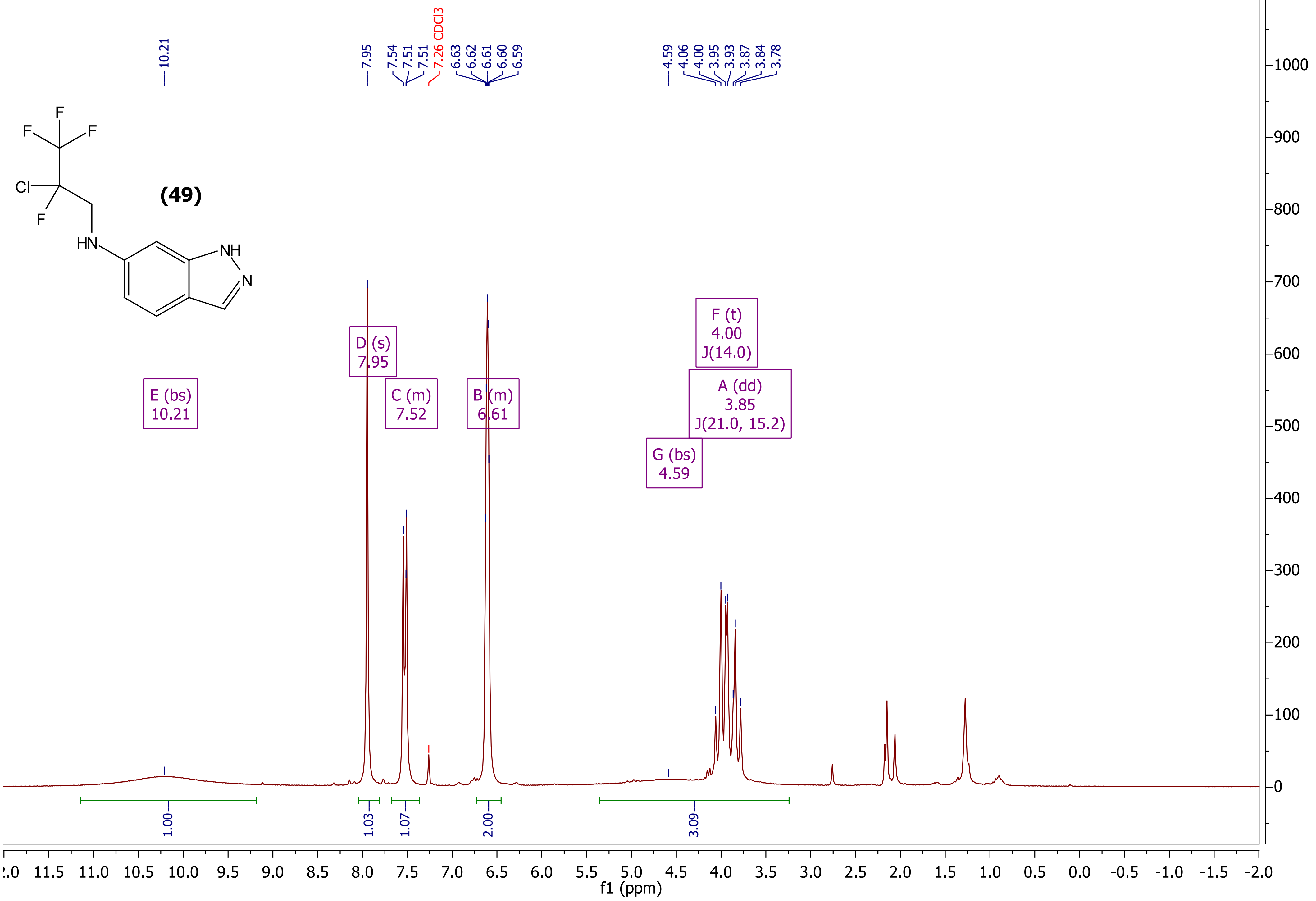

^{19}F NMR (235 MHz, Chloroform-*d*) δ -80.5 (d, *J* = 6.1 Hz), -129.9 (q, *J* = 6.2 Hz).

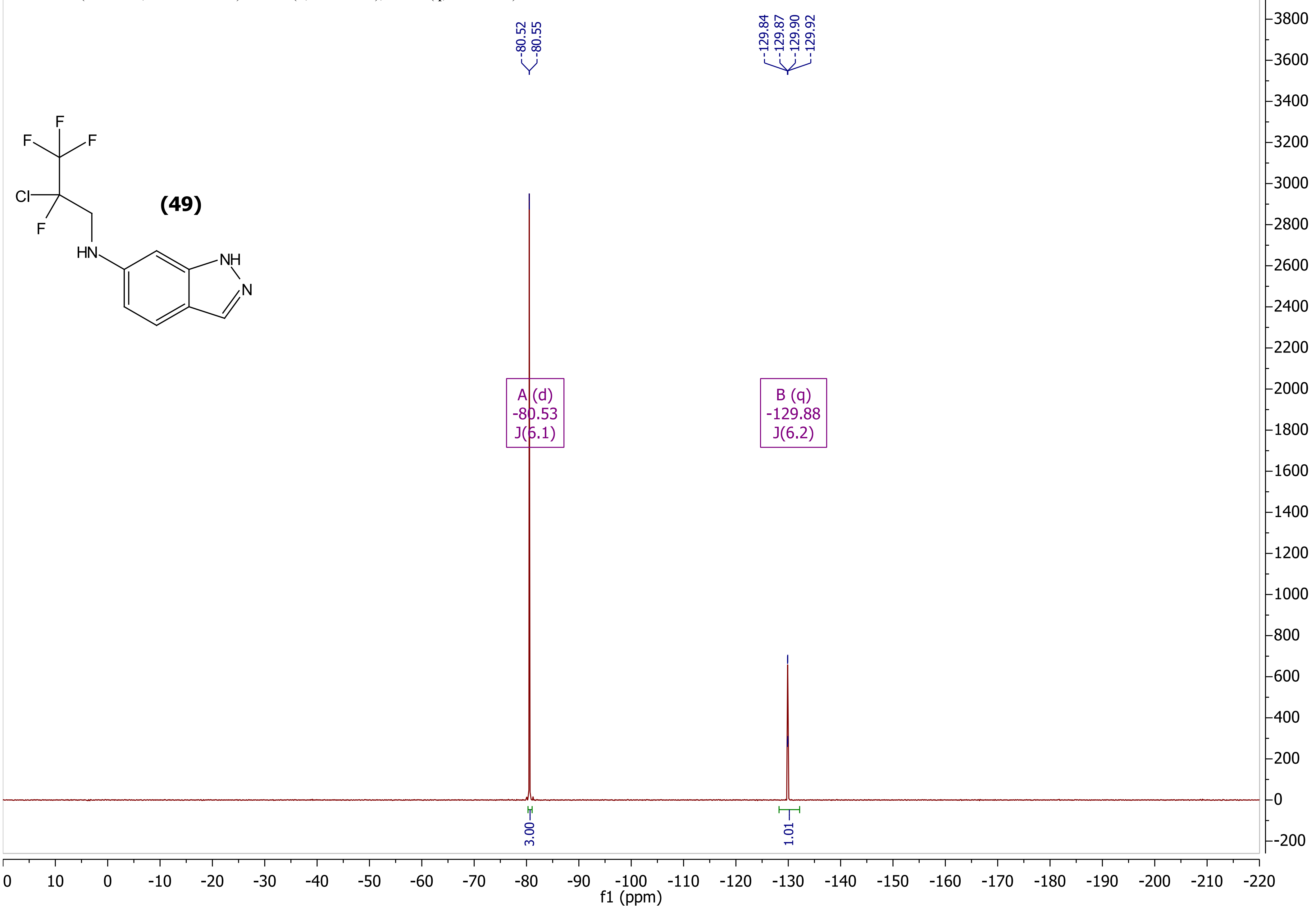

(49)

^{13}C NMR (63 MHz, Chloroform-*d*) δ 146.4, 141.9, 134.3, 121.9, 120.7 (qd, J = 284.9, 31.2 Hz), 117.3, 112.6, 106.9 (dq, J = 255.4, 34.8 Hz), 90.0 (d, J = 1.8 Hz), 49.0 (d, J = 22.2 Hz).

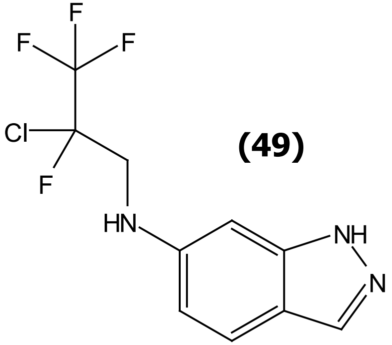

(49)

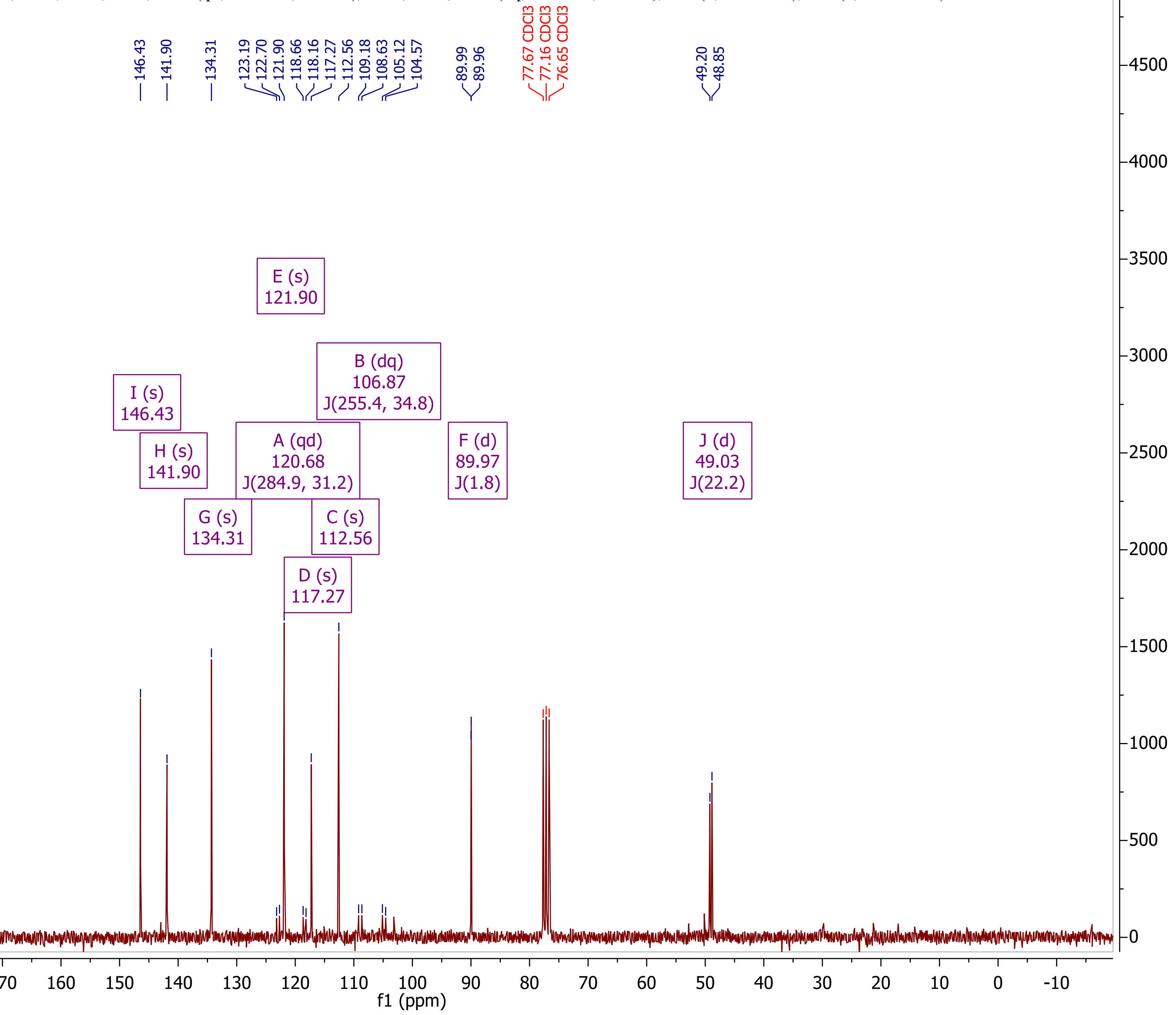

^{1}H NMR (400 MHz, Chloroform-*d*) δ 7.90 (s, 1H), 7.49 (d, *J* = 9.0 Hz, 1H), 6.76 (d, *J* = 2.2 Hz, 1H), 6.63 (dd, *J* = 9.0, 2.0 Hz, 1H), 5.10 (dd, *J* = 15.1, 12.7 Hz, 1H), 4.95 (dd, *J* = 22.5, 15.1 Hz, 1H), 4.21 (s, 1H), 4.04 (dd, *J* = 15.1, 12.2 Hz, 1H), 3.90 (dd, *J* = 21.5, 15.1 Hz, 1H).

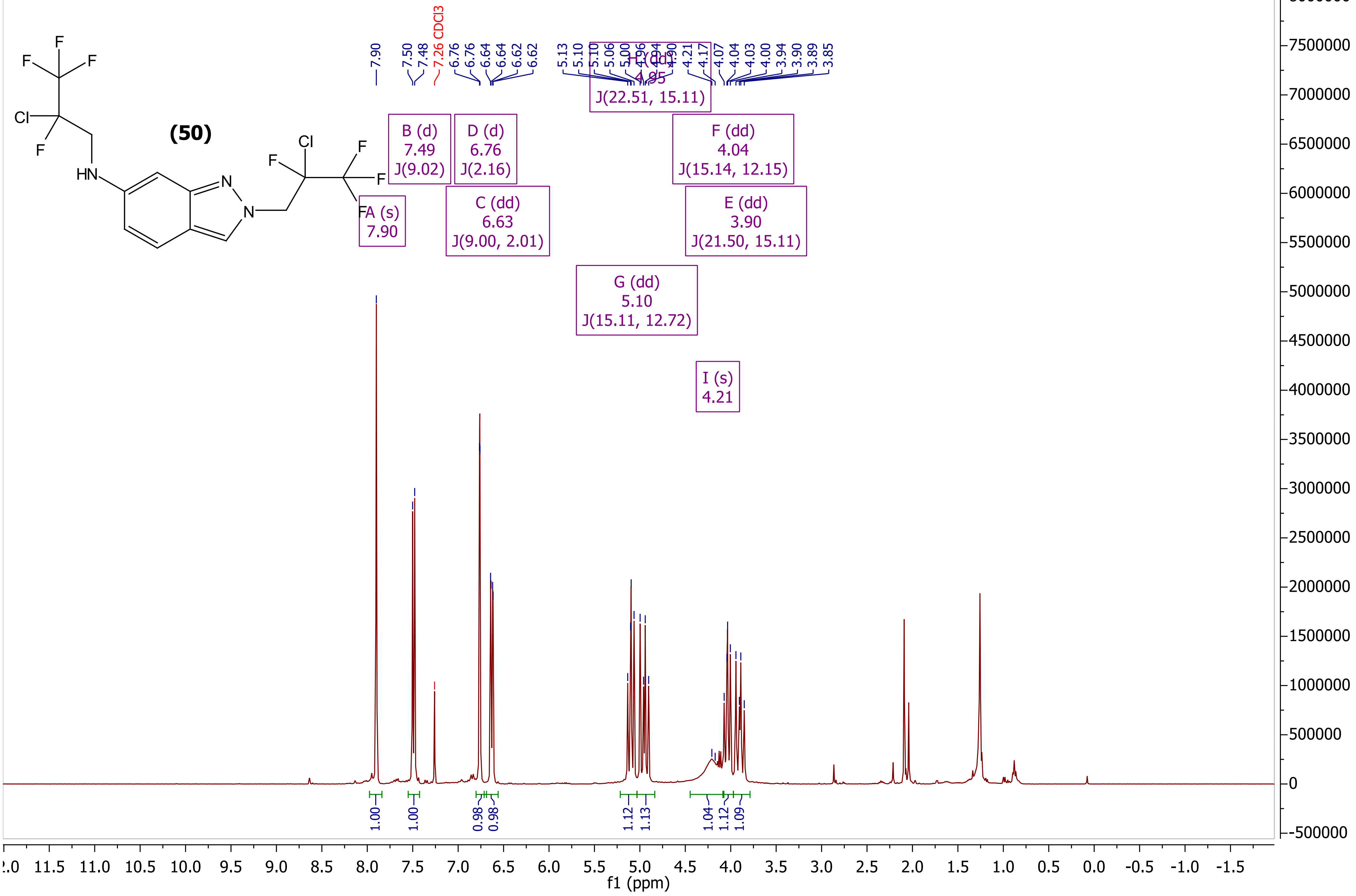

^{19}F NMR (376 MHz, Chloroform-*d*) δ -80.6 (d, *J* = 6.1 Hz), -80.8 (d, *J* = 6.2 Hz), -128.2 (q, *J* = 6.3 Hz), -129.8 – -130.1 (m).

(50)

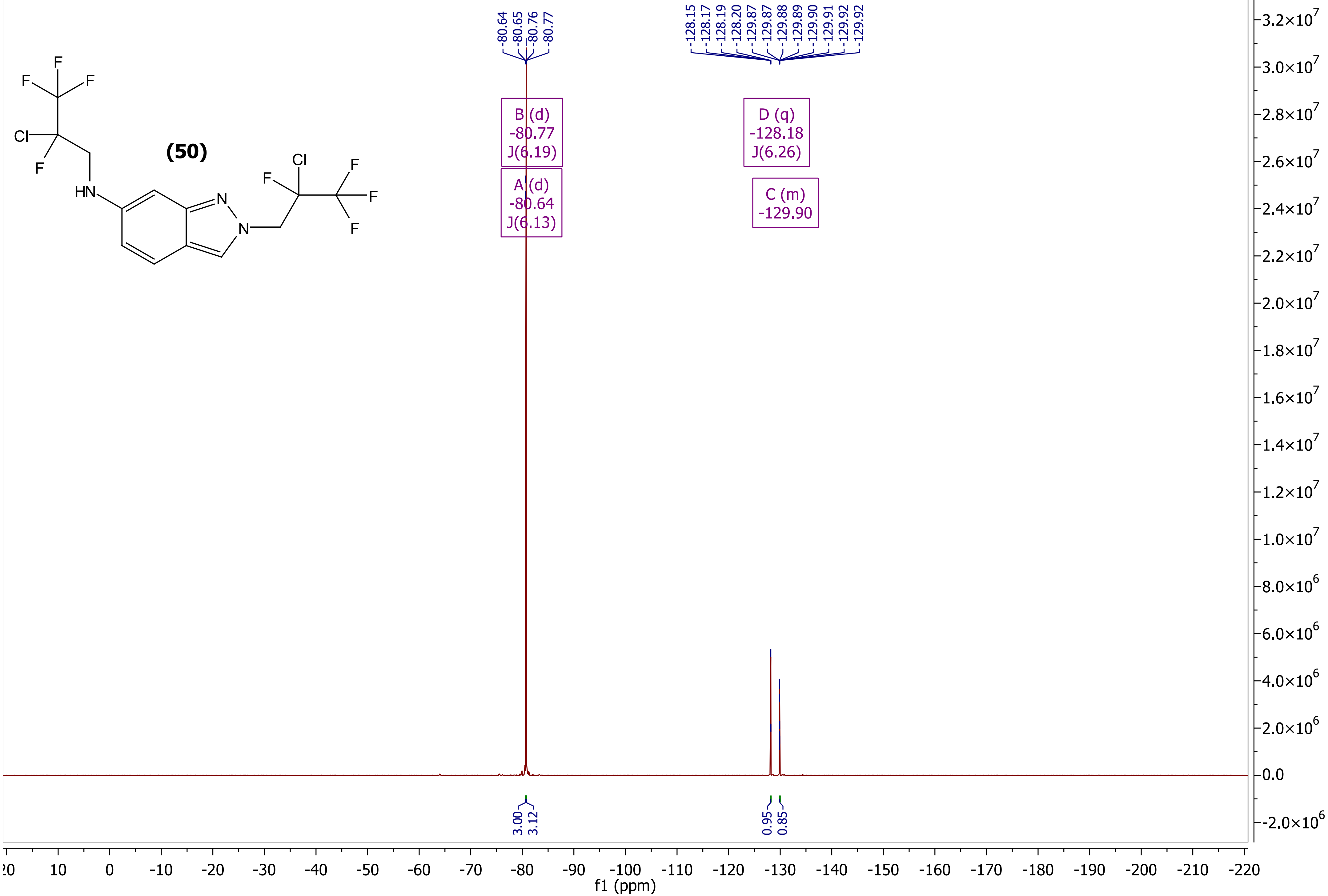

^{13}C NMR (101 MHz, Chloroform-*d*) δ 150.7, 145.6, 125.2 (d, *J* = 1.8 Hz), 121.4, 120.7 (qd, *J* = 284.8, 31.0 Hz), 120.3 (qd, *J* = 285.4, 31.0 Hz), 117.7, 117.3, 106.8 (dq, *J* = 255.3, 34.9 Hz), 104.4 (dq, *J* = 256.8, 36.3 Hz), 93.5 (d, *J* = 1.8 Hz), 56.6 (d, *J* = 22.1 Hz), 48.8 (d, *J* = 22.2 Hz).

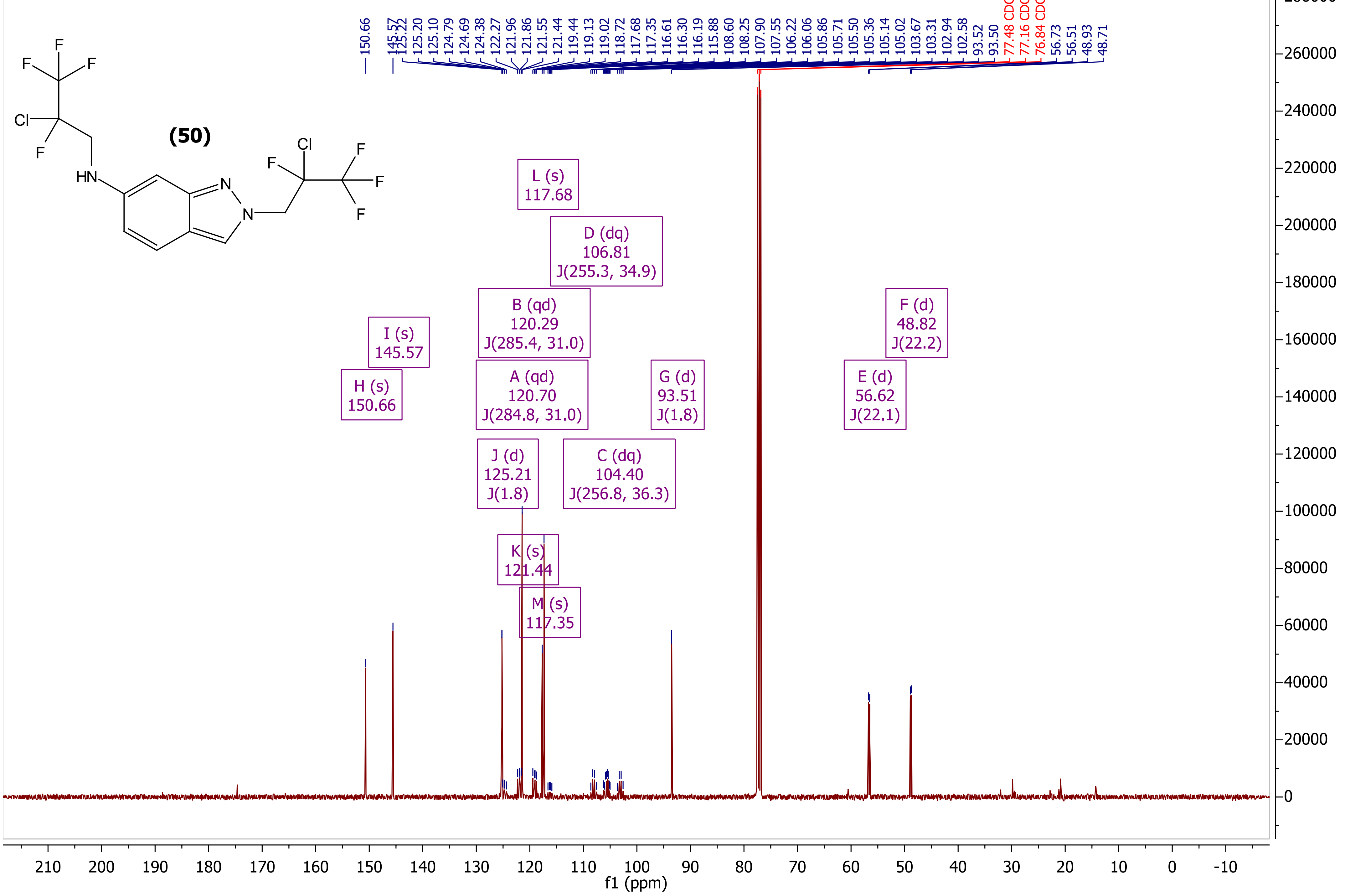

^{1}H NMR (500 MHz, DMSO-d_6) δ 8.13 – 8.07 (m, 2H), 7.62 – 7.57 (m, 3H), 6.19 – 6.05 (m, 2H).

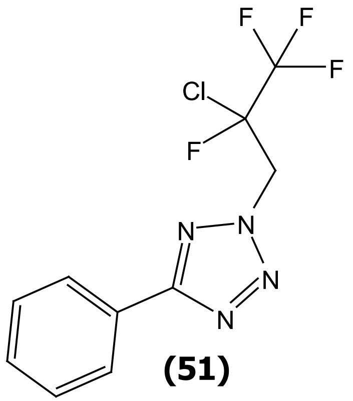

(51)

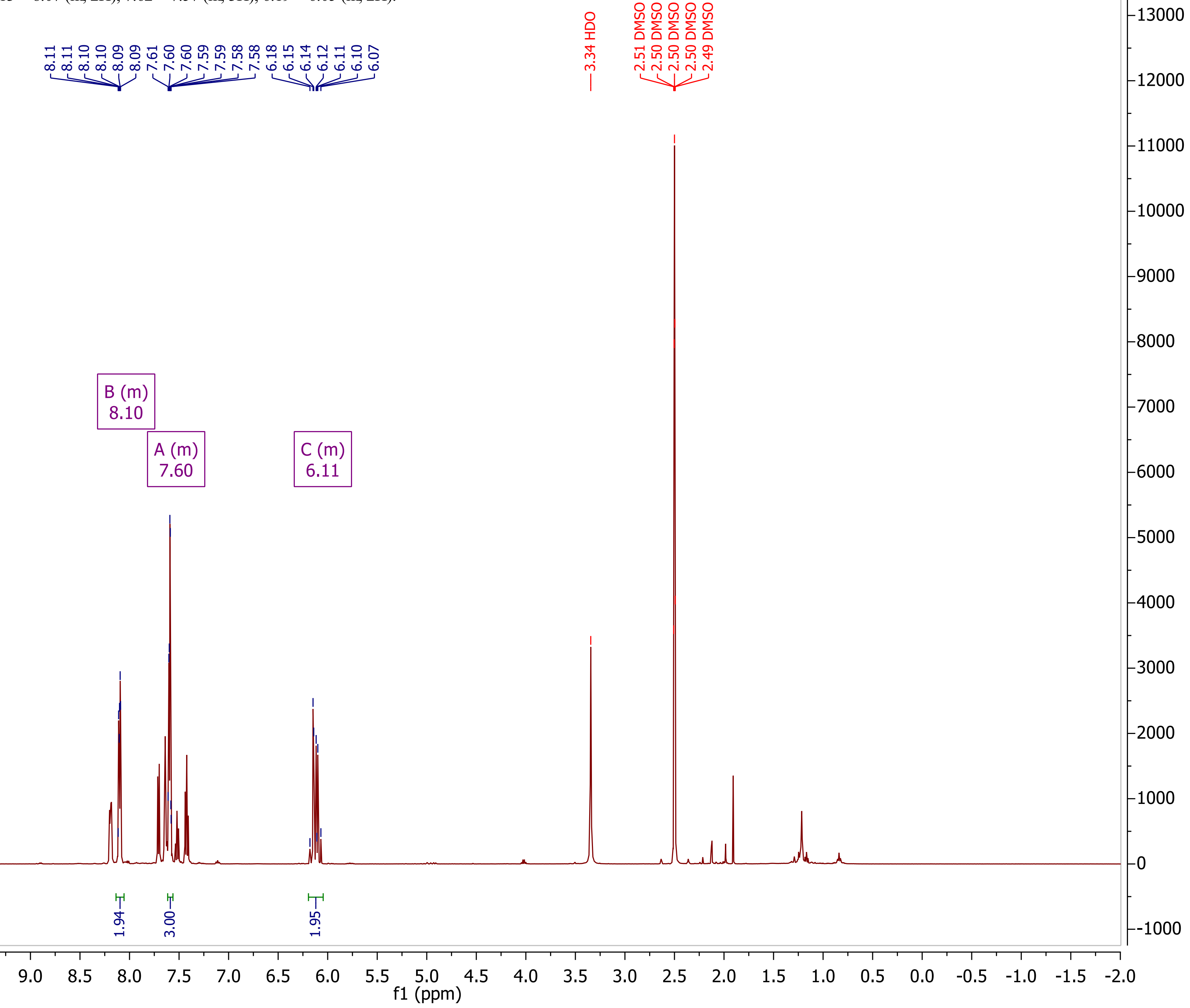

^{19}F NMR (376 MHz, DMSO-d_6) δ -79.9 (d, J = 6.5 Hz), -127.2 – -127.4 (m).

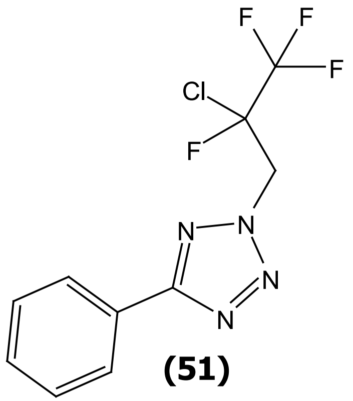

(51)

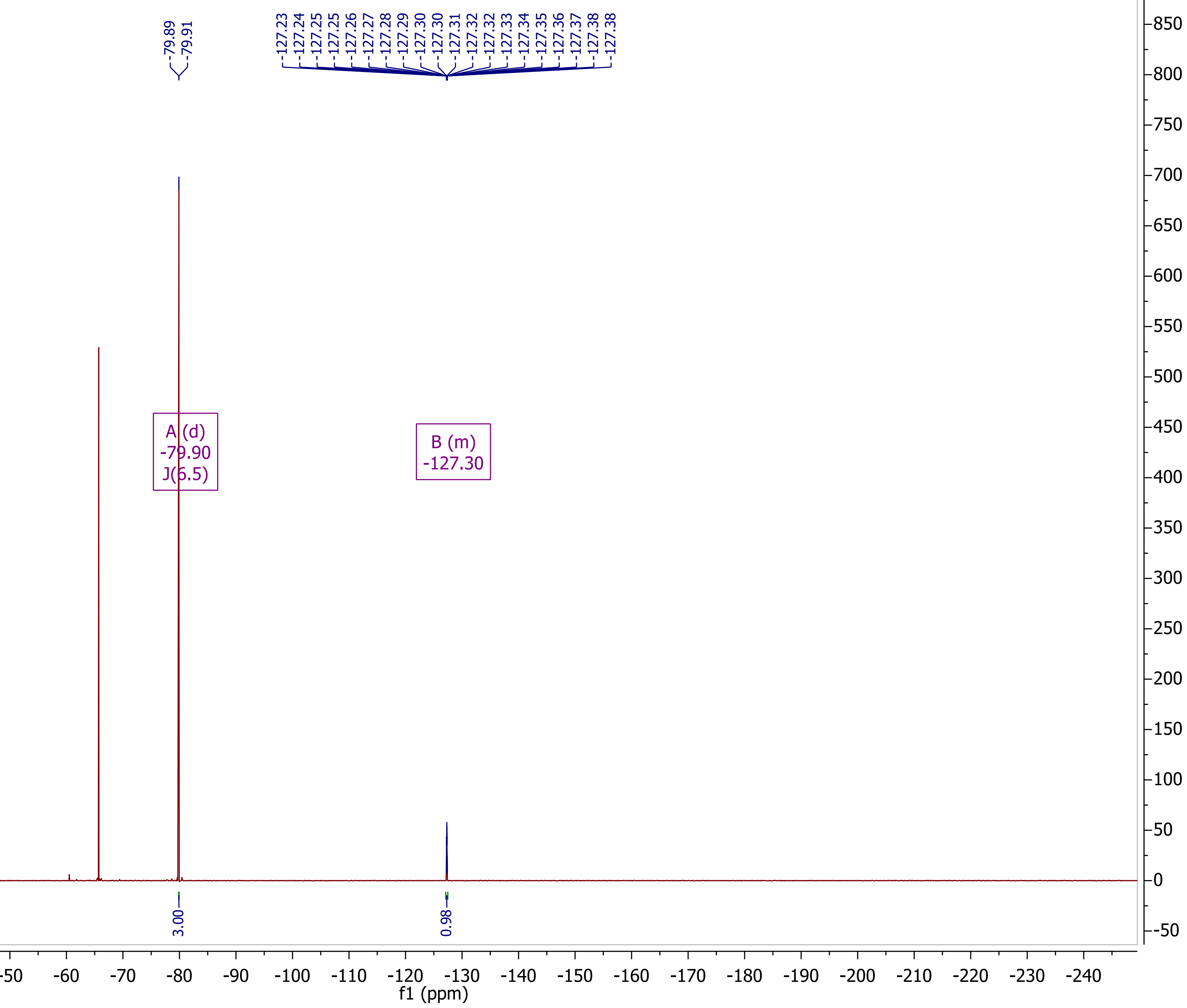

^{13}C NMR (126 MHz, DMSO-d_6) δ 164.8, 131.1, 129.5, 126.6, 126.1, 119.8 (qd, J = 285.3, 31.0 Hz), 103.4 (dq, J = 256.6, 36.8 Hz), 54.9 (d, J = 22.4 Hz).

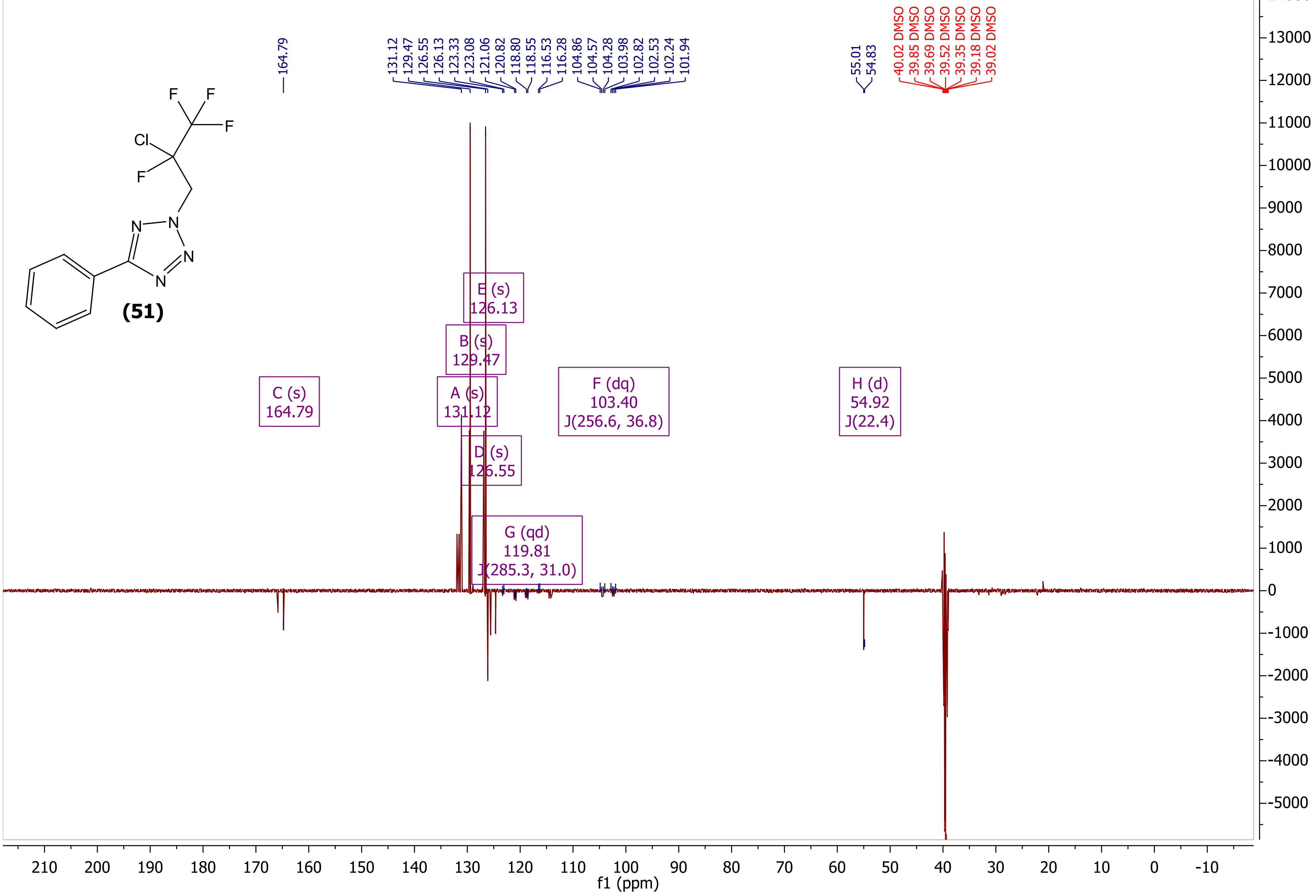

^{1}H NMR (500 MHz, DMSO-d_6) δ 9.83 (s, 1H), 8.21 – 8.17 (m, 2H), 7.74 – 7.68 (m, 2H), 7.67 – 7.61 (m, 3H), 7.52 (t, J = 7.5 Hz, 1H), 7.42 (t, J = 7.7 Hz, 2H).

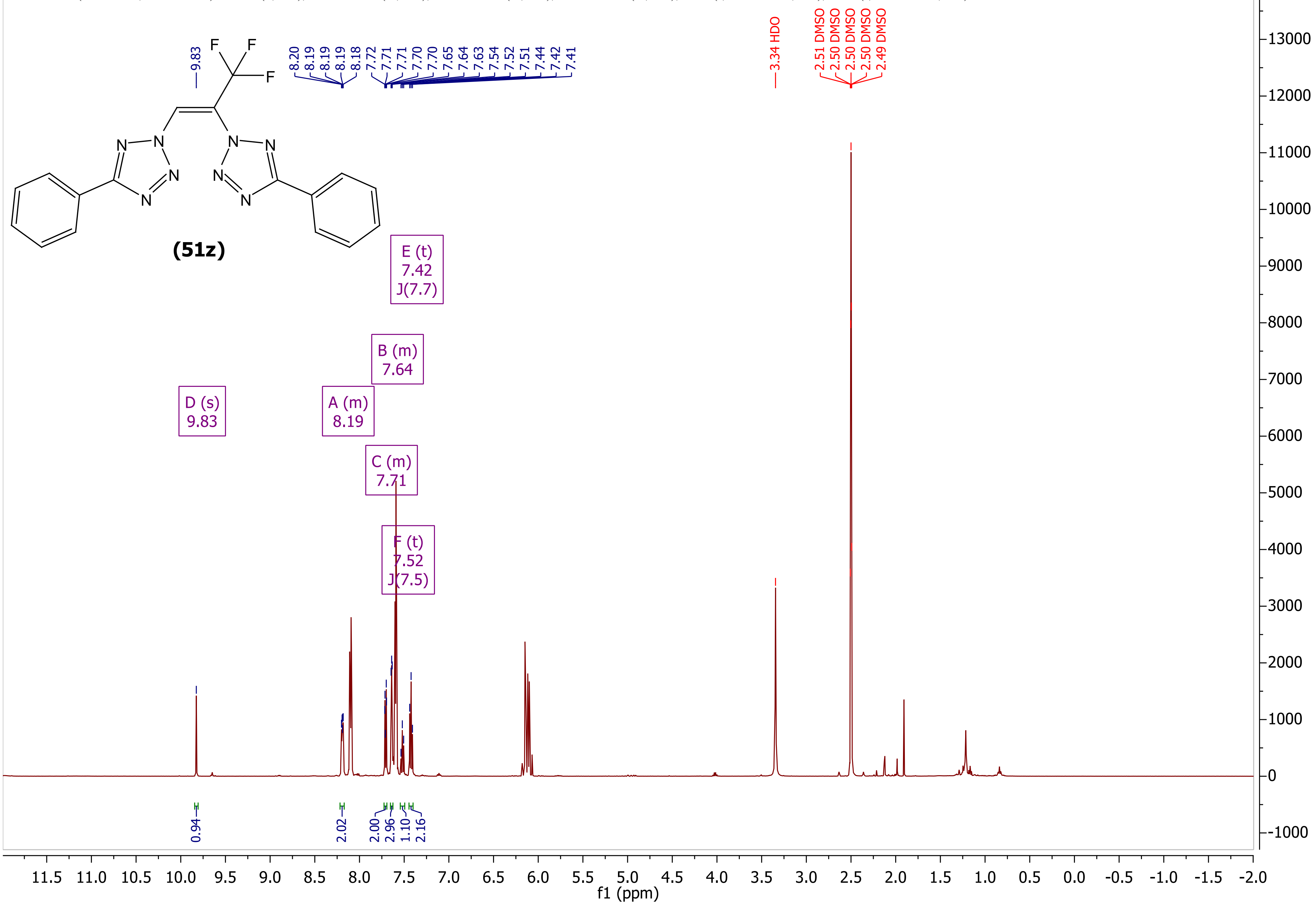

^{19}F NMR (376 MHz, DMSO-d_6) δ -65.7 (d, J = 1.1 Hz).

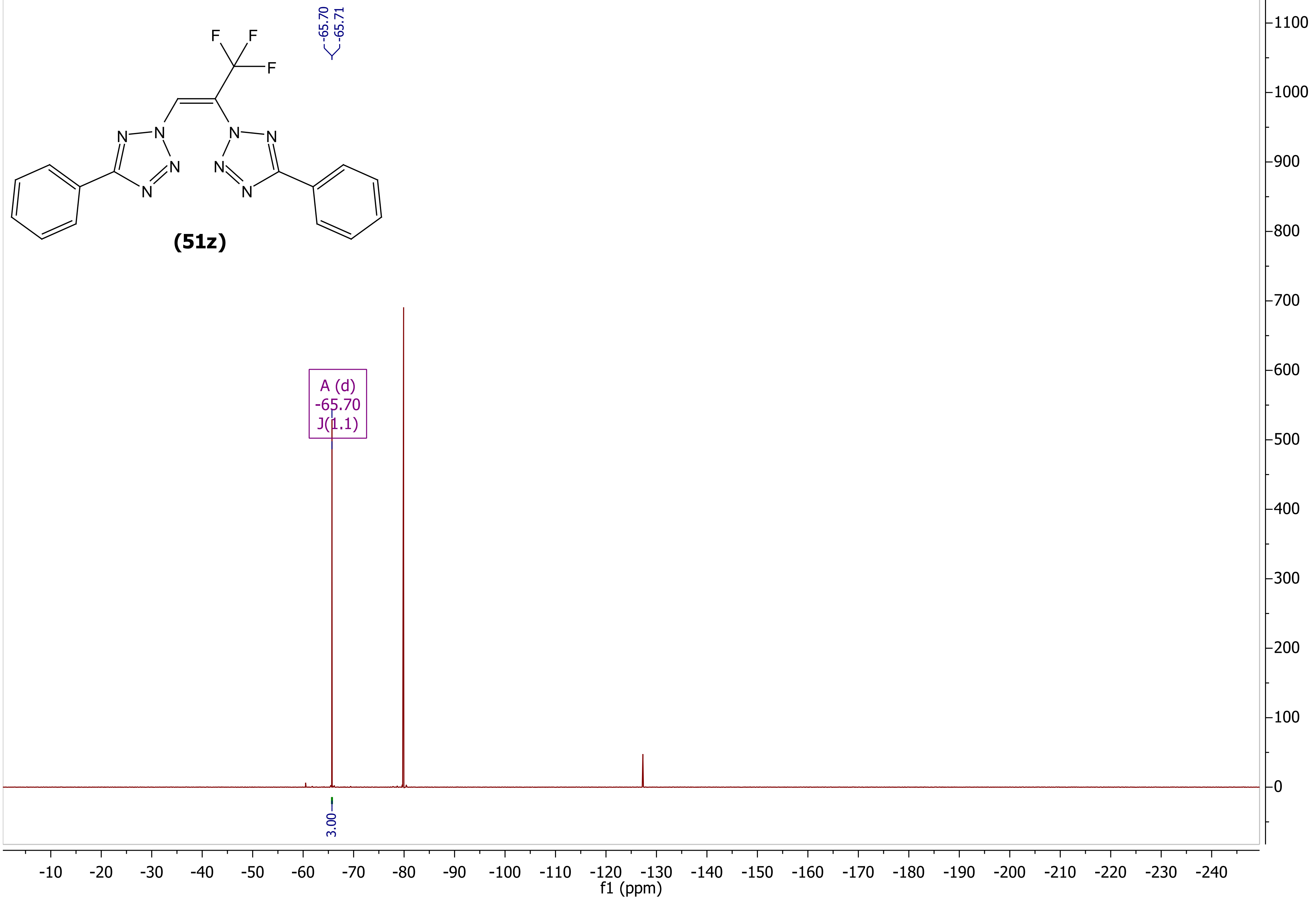

^{13}C NMR (126 MHz, DMSO-d_6) δ 165.8, 164.9, 132.0, 131.8 (q, J = 4.6 Hz), 131.6, 129.6, 129.4, 126.9, 126.6, 125.6, 124.7, 120.1 (q, J = 273.5 Hz), 114.4 (q, J = 39.1 Hz).

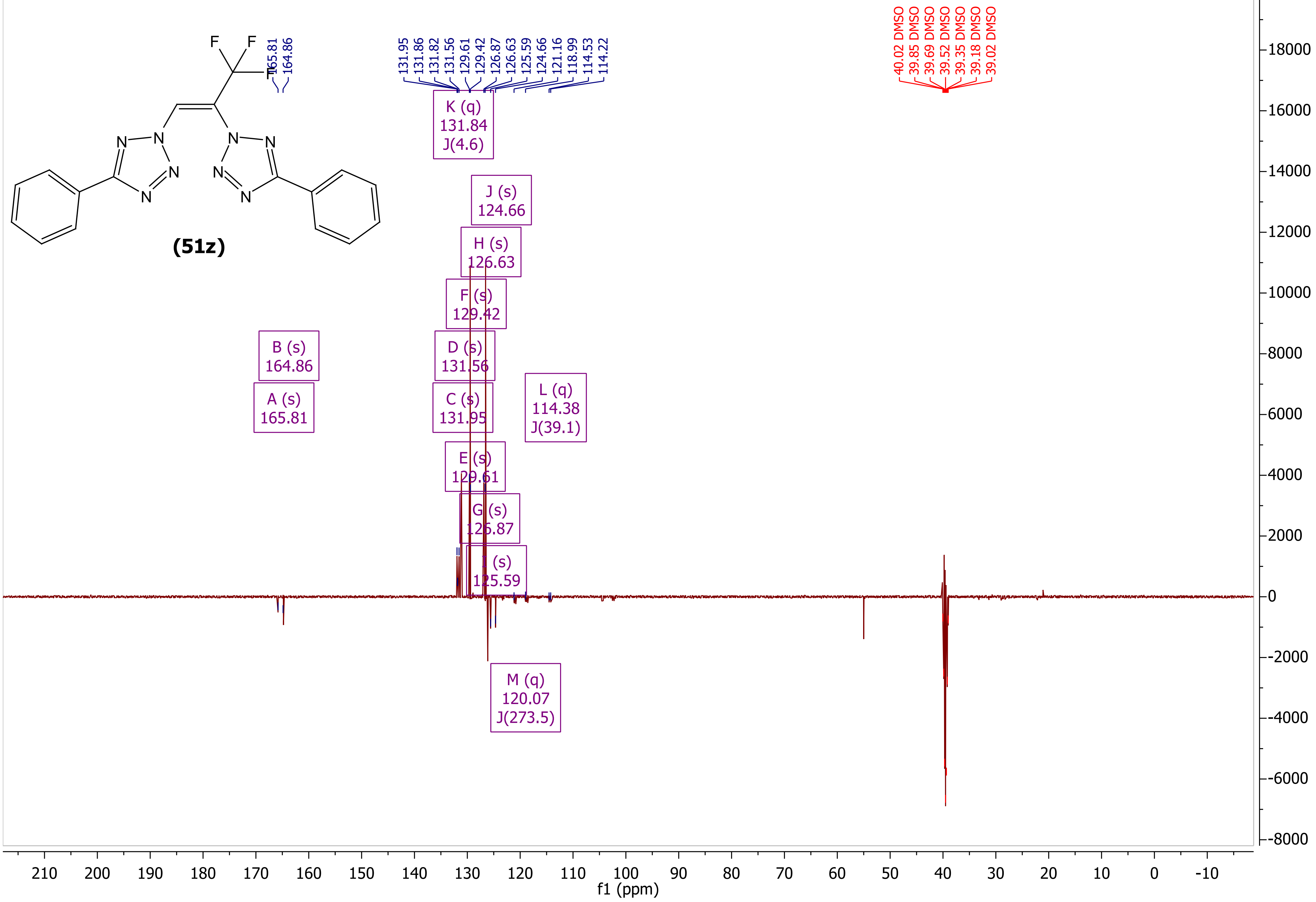

^{1}H NMR (250 MHz, Chloroform-*d*) δ 8.10 (d, *J* = 8.3 Hz, 1H), 7.67 – 7.49 (m, 2H), 7.50 – 7.33 (m, 1H), 5.49 – 5.18 (m, 2H).

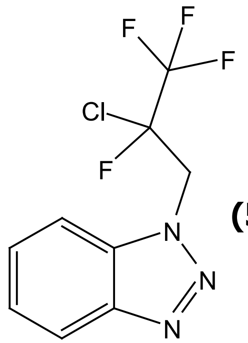

(52)

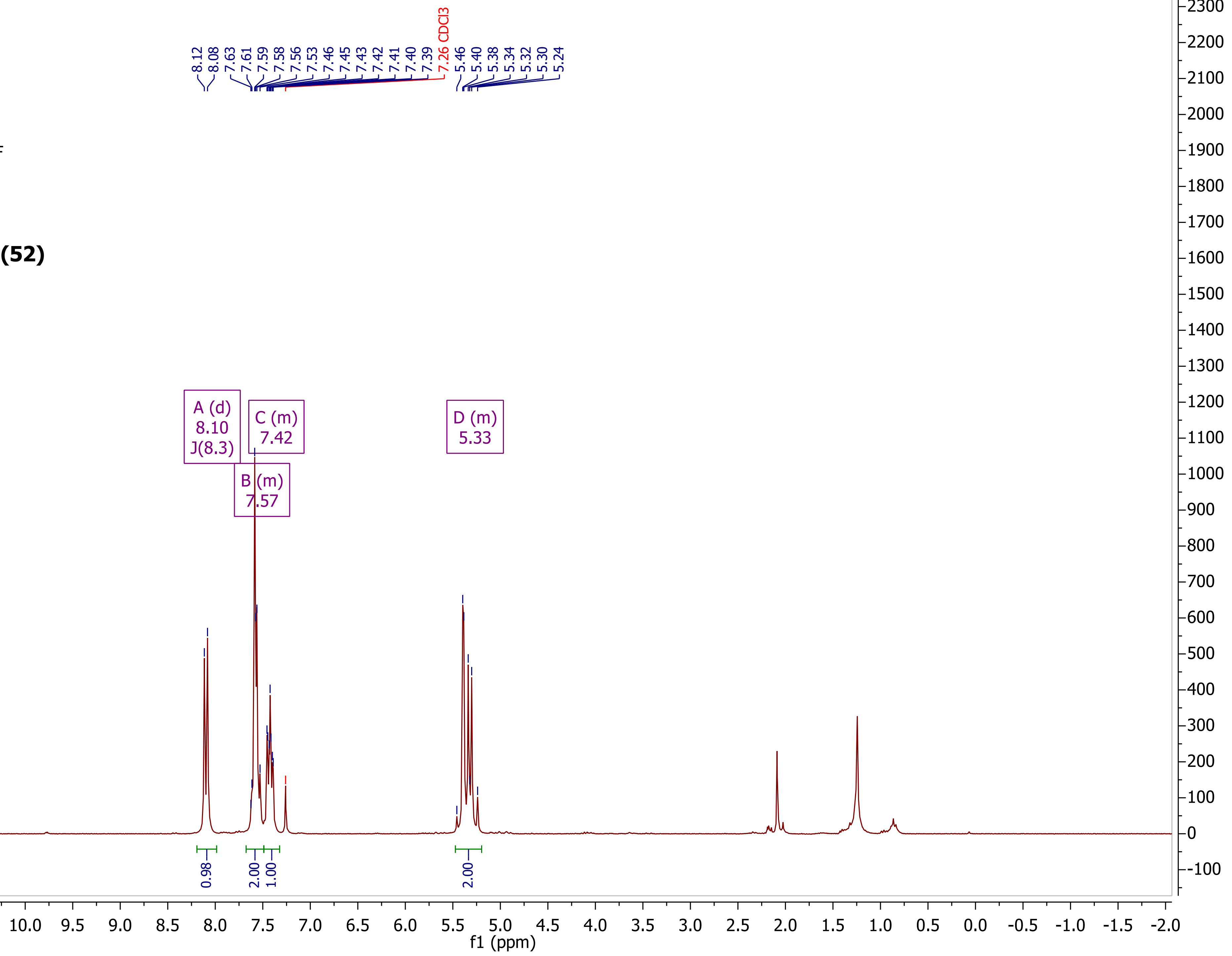

^{19}F NMR (235 MHz, Chloroform-*d*) δ -80.7 (d, J = 6.1 Hz), -126.9 (q, J = 6.0 Hz).

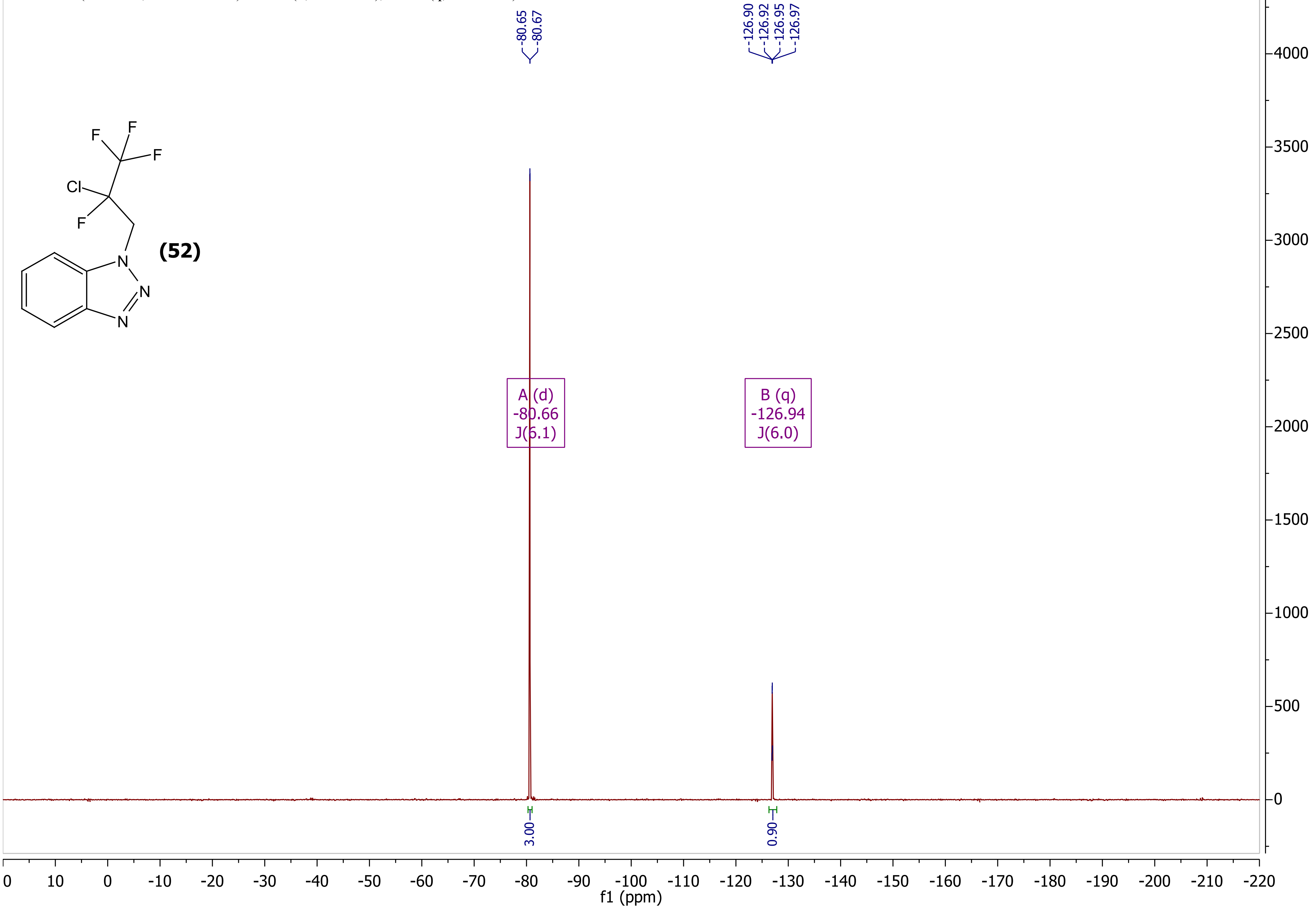

^{13}C NMR (63 MHz, Chloroform-d) δ 146.1, 133.7, 128.7, 124.7, 120.5, 120.2 (qd, J = 285.2, 30.6 Hz), 109.6 (d, J = 3.5 Hz), 104.8 (dq, J = 257.2, 36.5 Hz), 52.0 (d, J = 22.9 Hz).

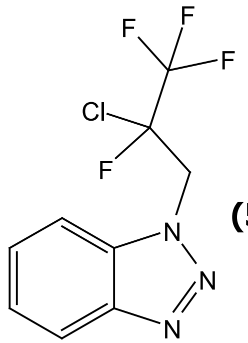

(52)

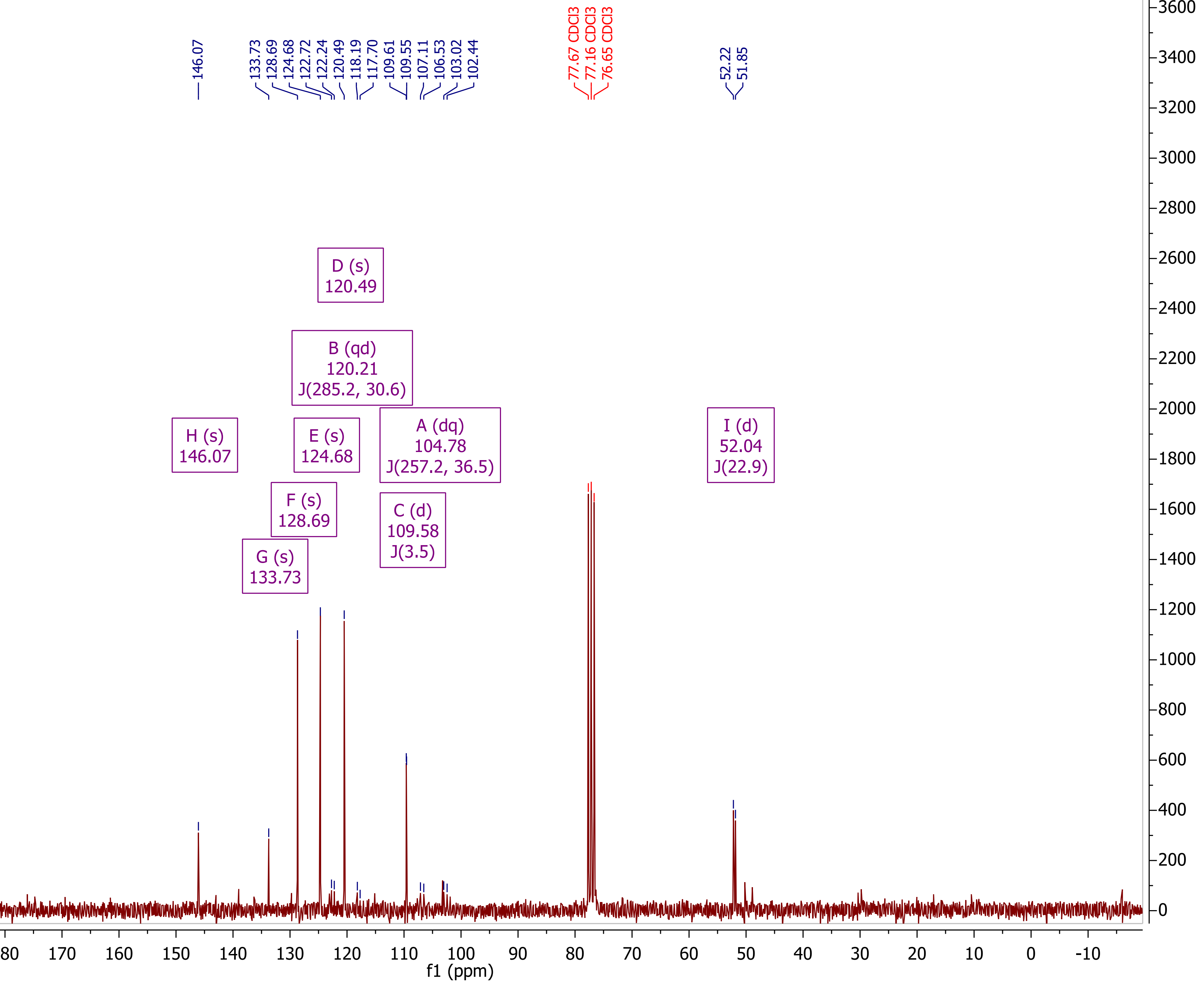

^{1}H NMR (500 MHz, DMSO-d_6) δ 8.65 (q, J = 1.2 Hz, 1H), 8.25 (dt, J = 8.4, 1.0 Hz, 1H), 7.78 (dt, J = 8.4, 1.1 Hz, 1H), 7.76 – 7.71 (m, 1H), 7.61 – 7.53 (m, 1H).

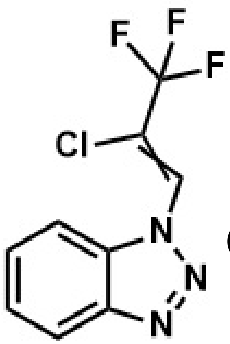

(52x)

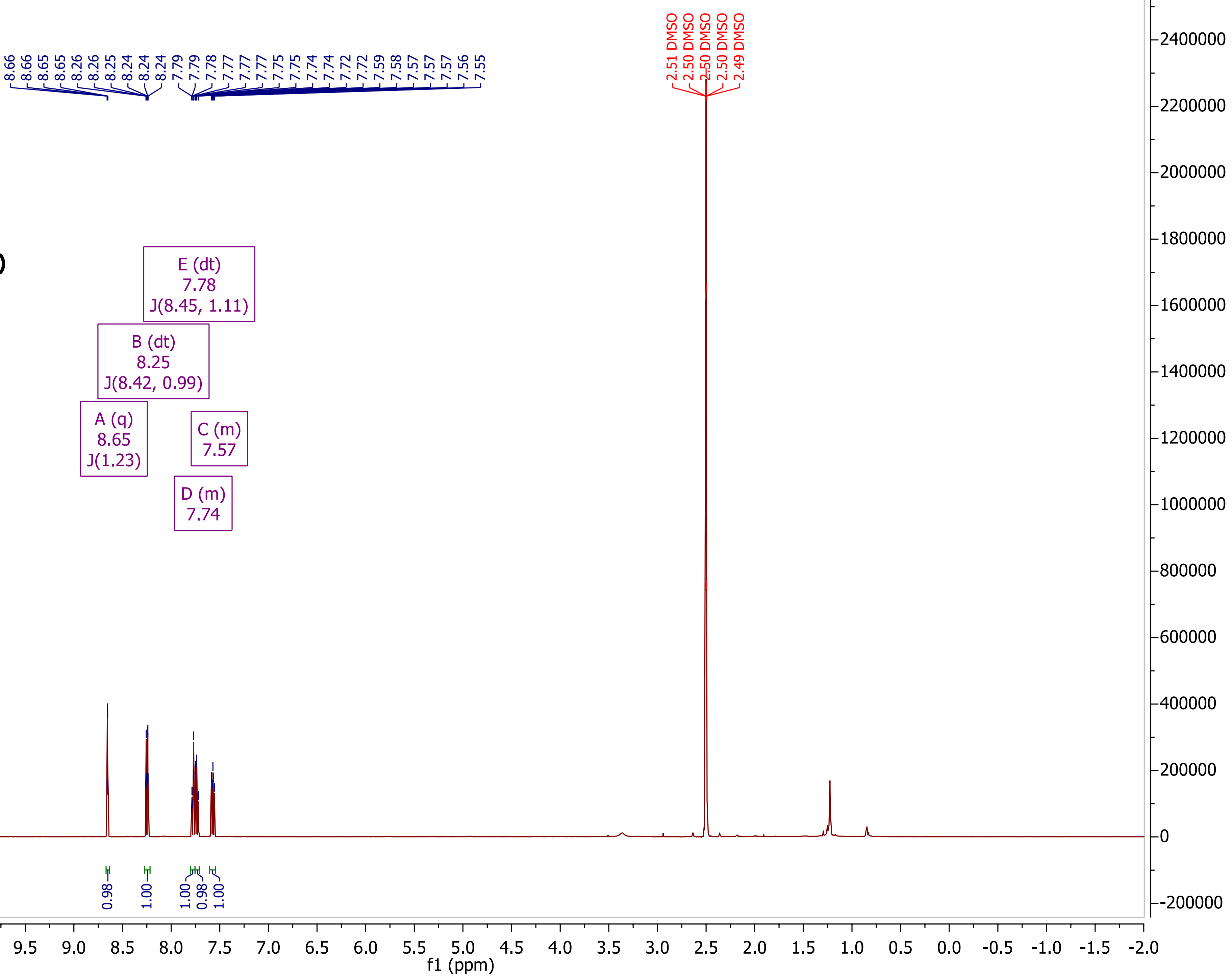

^{19}F NMR (376 MHz, DMSO-d_6) δ -66.2.

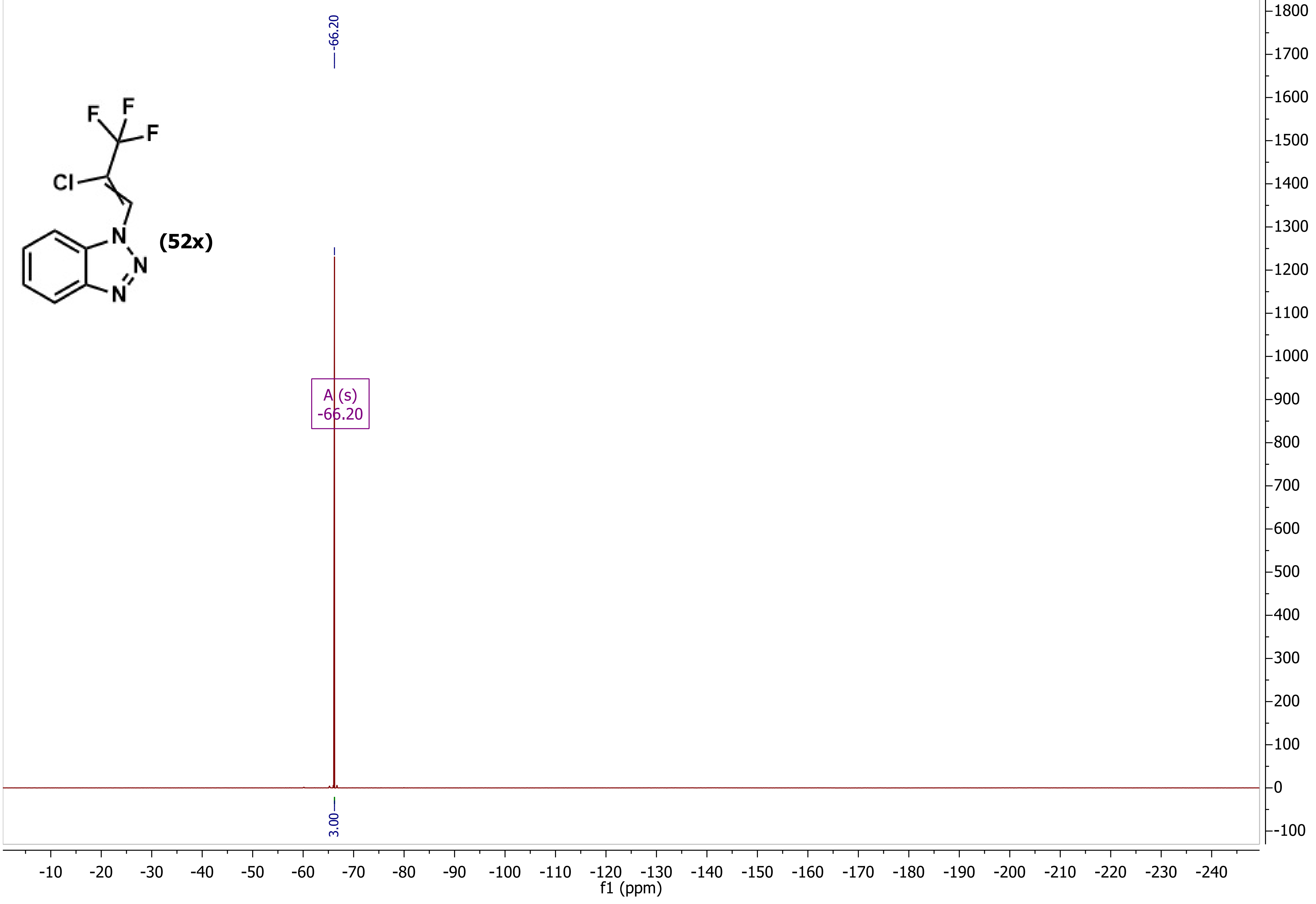

^{13}C NMR (126 MHz, DMSO-d_6) δ 144.5, 135.6 (q, J = 4.8 Hz), 132.9, 129.9, 125.4, 125.0 (q, J = 36.8 Hz), 120.2 (q, J = 274.5 Hz), 120.1, 110.1.

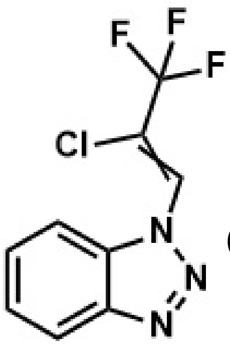

(52x)

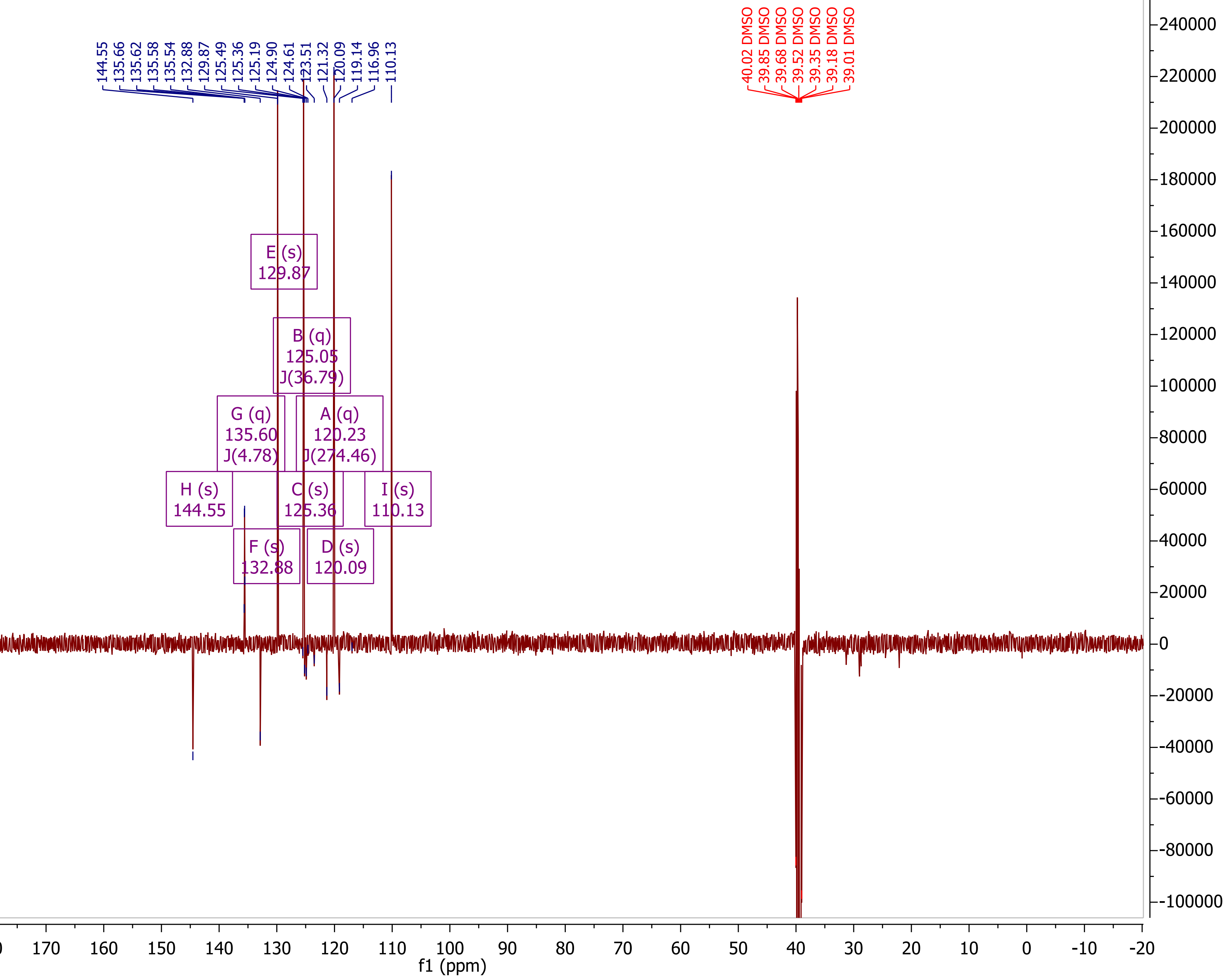

^{1}H NMR (500 MHz, DMSO-d_6) δ 9.34 (d, J = 1.2 Hz, 1H), 8.26 (dt, J = 8.3, 1.0 Hz, 1H), 7.68 – 7.61 (m, 3H), 7.59 – 7.56 (m, 1H), 7.54 – 7.49 (m, 1H), 7.45 – 7.41 (m, 2H).

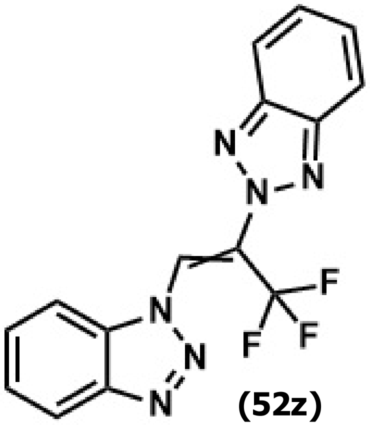

(52z)

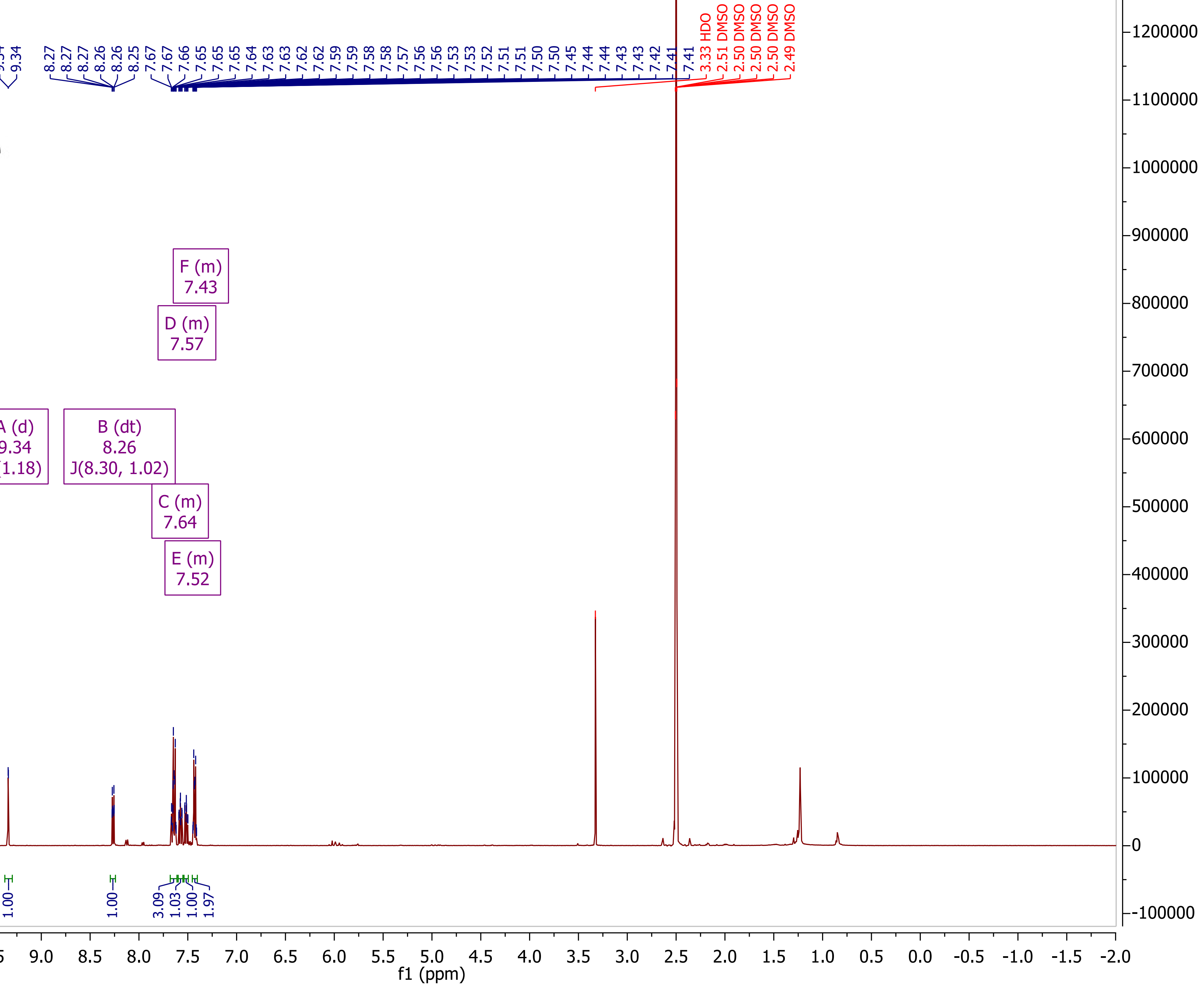

^{19}F NMR (376 MHz, DMSO-d_6) δ -66.0.

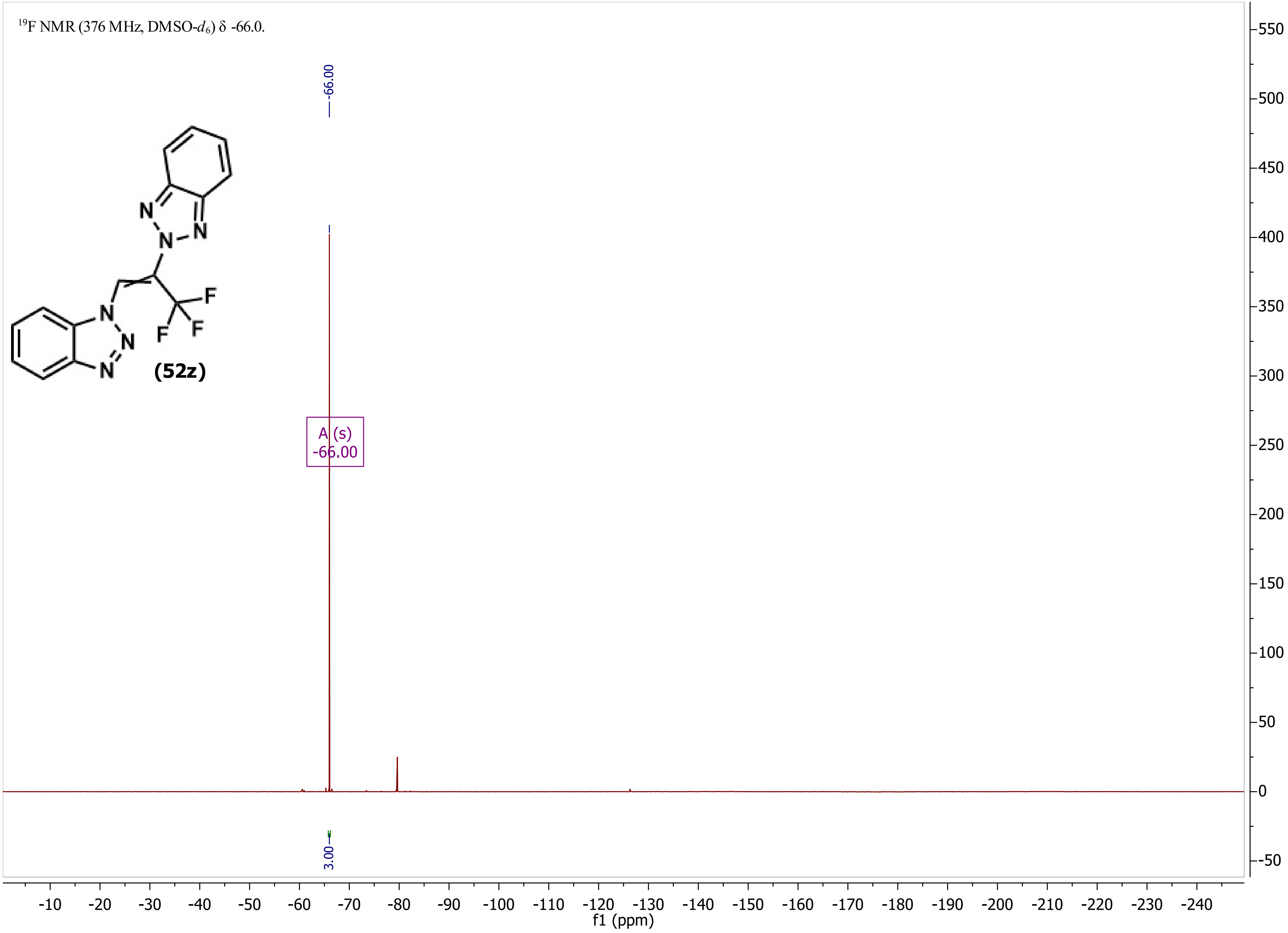

(52z)

^{13}C NMR (126 MHz, DMSO-d_6) δ 145.6, 145.5, 134.7 (q, J = 4.7 Hz), 134.5, 130.5, 129.9, 125.4, 121.7 (q, J = 274.5 Hz), 120.4, 118.8, 114.6 (q, J = 36.9 Hz), 110.6.

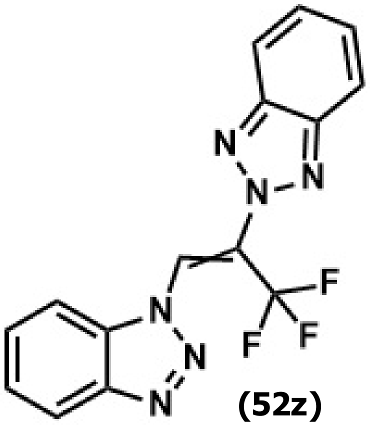

(52z)

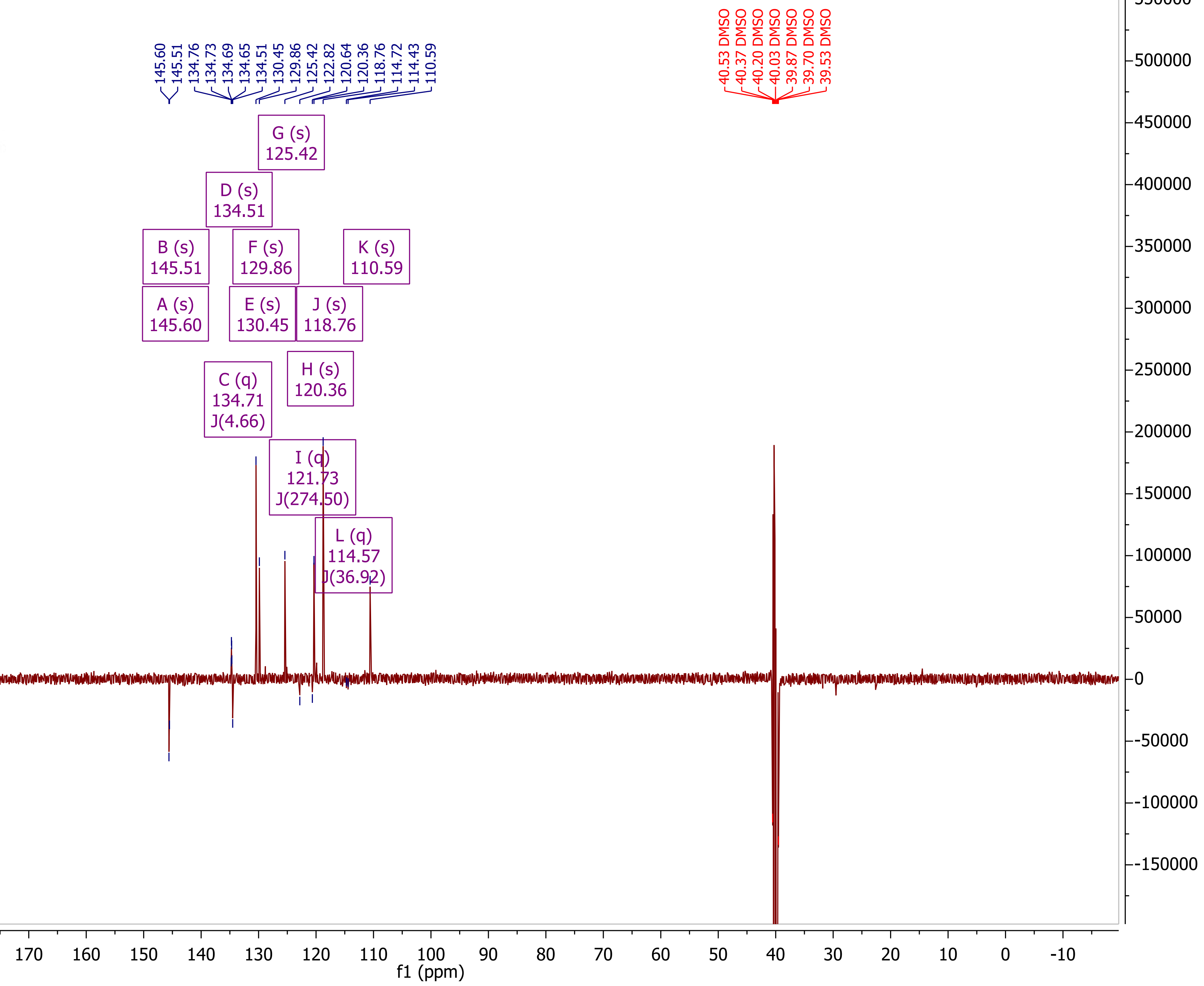

^{1}H NMR (250 MHz, Chloroform-*d*) δ 8.05 (s, 1H), 7.74 (d, *J* = 8.8 Hz, 1H), 7.67 (d, *J* = 8.5 Hz, 1H), 7.37 – 7.28 (m, 1H), 7.17 – 7.07 (m, 1H), 5.19 (dd, *J* = 15.0, 12.8 Hz, 1H), 5.03 (dd, *J* = 22.4, 15.0 Hz, 1H).

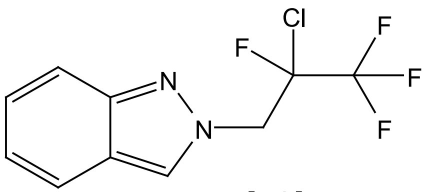

(53)

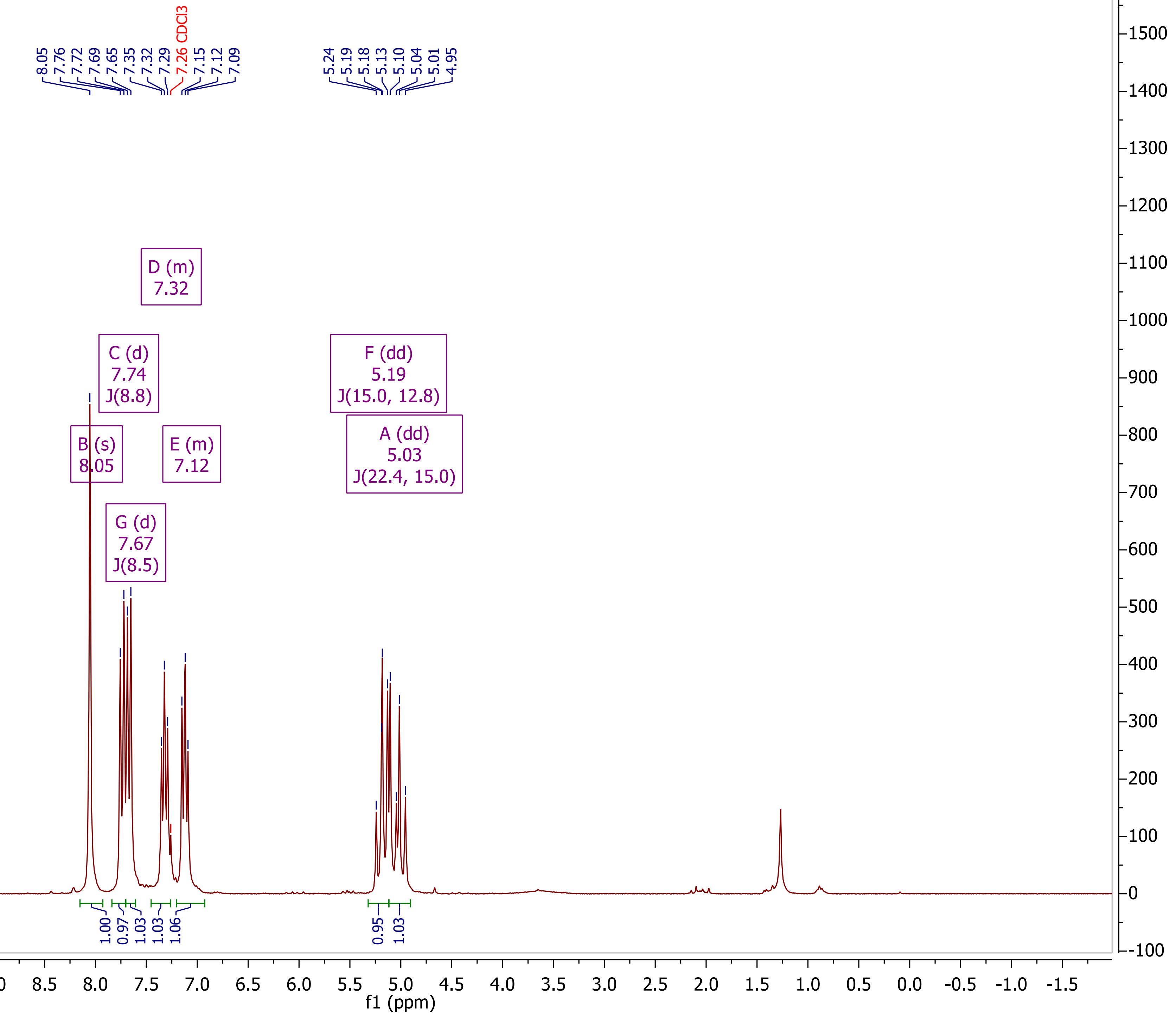

^{19}F NMR (235 MHz, Chloroform-*d*) δ -80.8 (d, *J* = 6.1 Hz), -128.1 (q, *J* = 6.1 Hz).

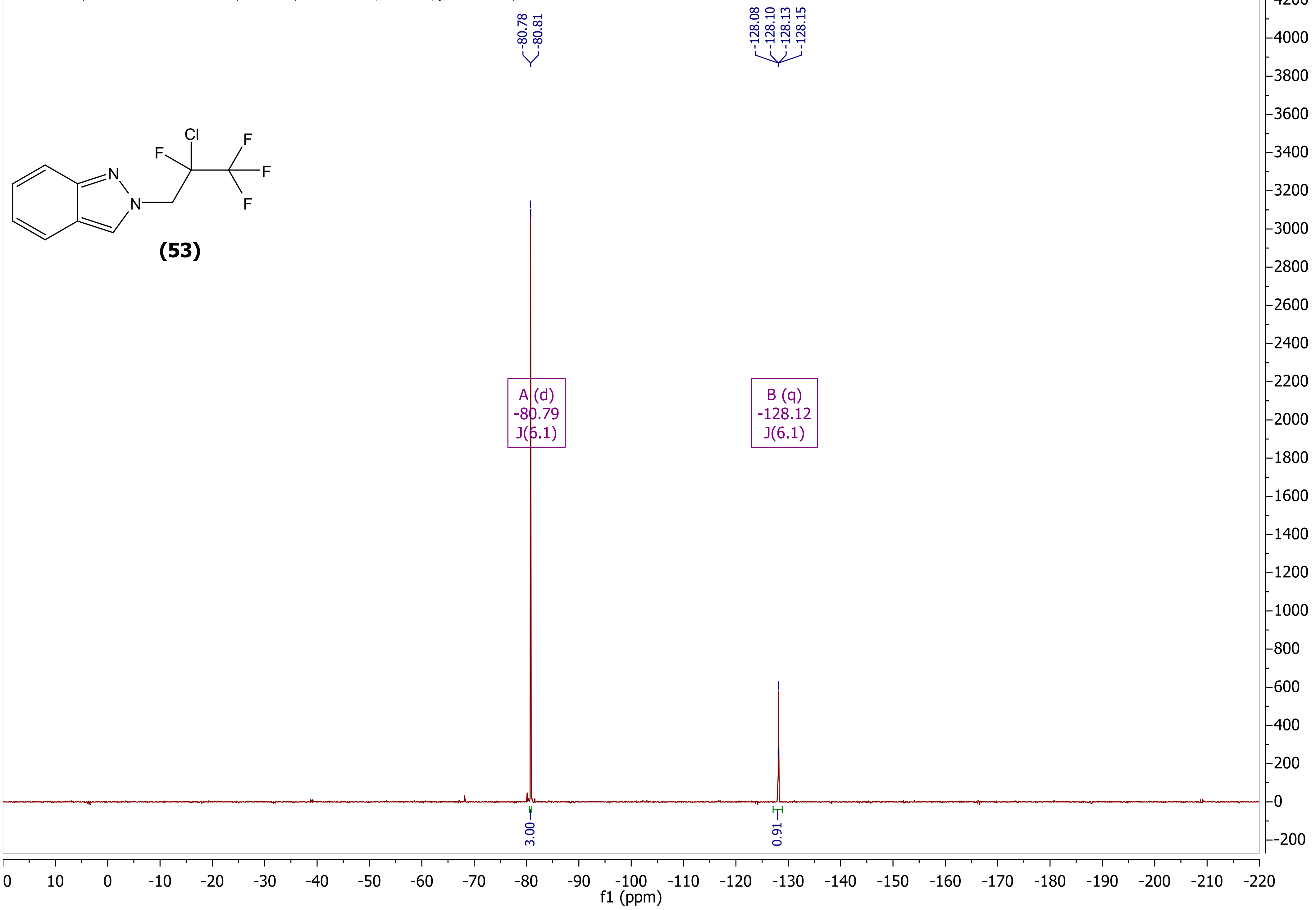

(53)

^{13}C NMR (63 MHz, Chloroform-*d*) δ 149.3, 127.1, 125.0 (d, *J* = 1.6 Hz), 122.8, 122.5, 120.4, 119.4 (qd, *J* = 285.4, 30.6 Hz), 117.9, 104.3 (dq, *J* = 257.2, 36.6 Hz), 57.0 (d, *J* = 21.9 Hz).

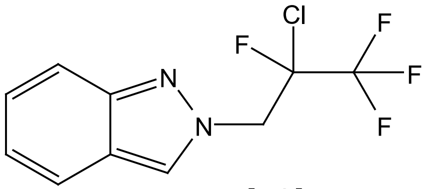

(53)

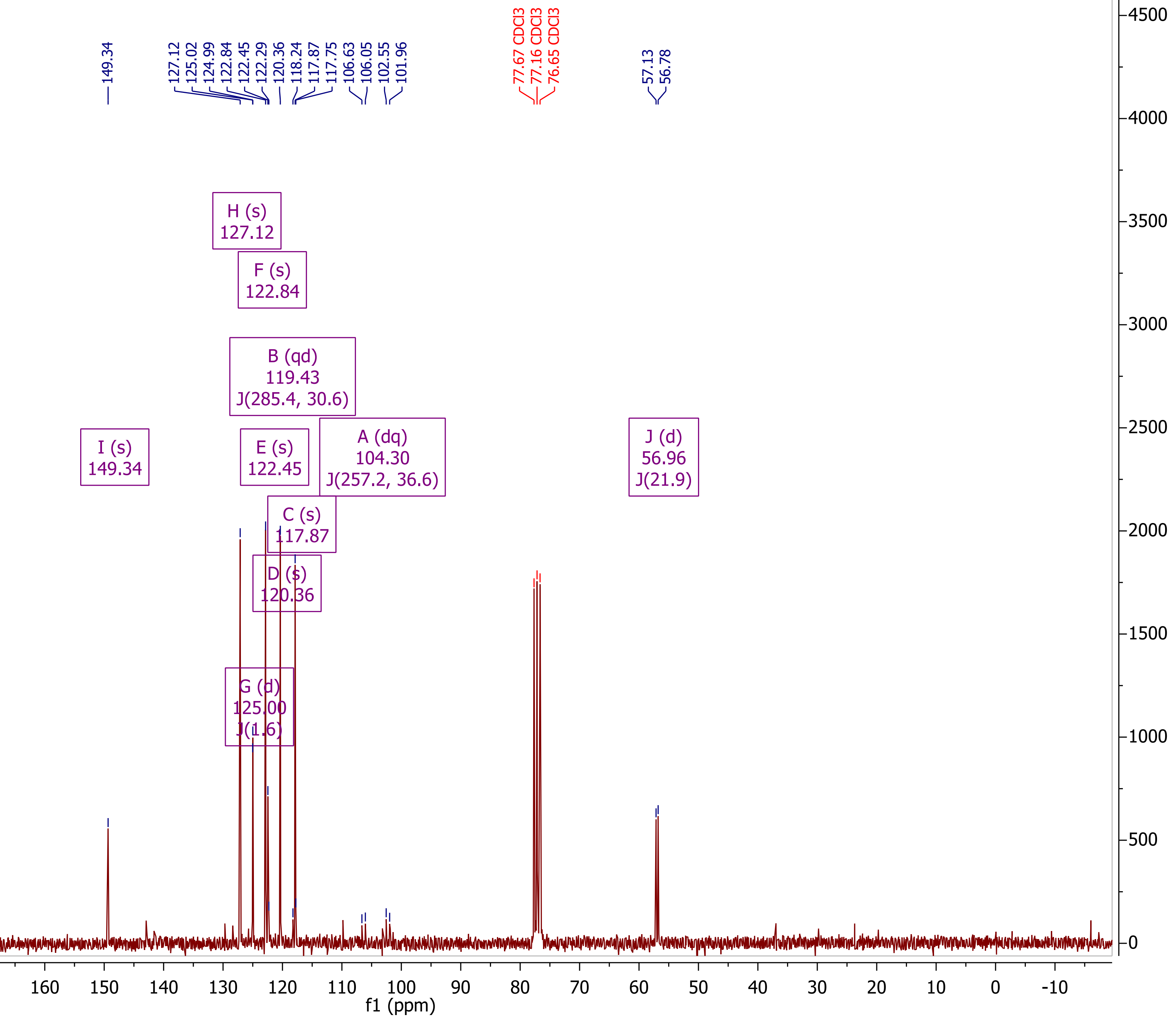

^{1}H NMR (500 MHz, DMSO-d_6) δ 7.90 – 7.87 (m, 2H), 7.59 – 7.29 (m), 7.01 (s, 1H), 5.24 (dd, J = 16.0, 14.2 Hz, 1H), 5.08 (dd, J = 22.5, 16.0 Hz, 1H).

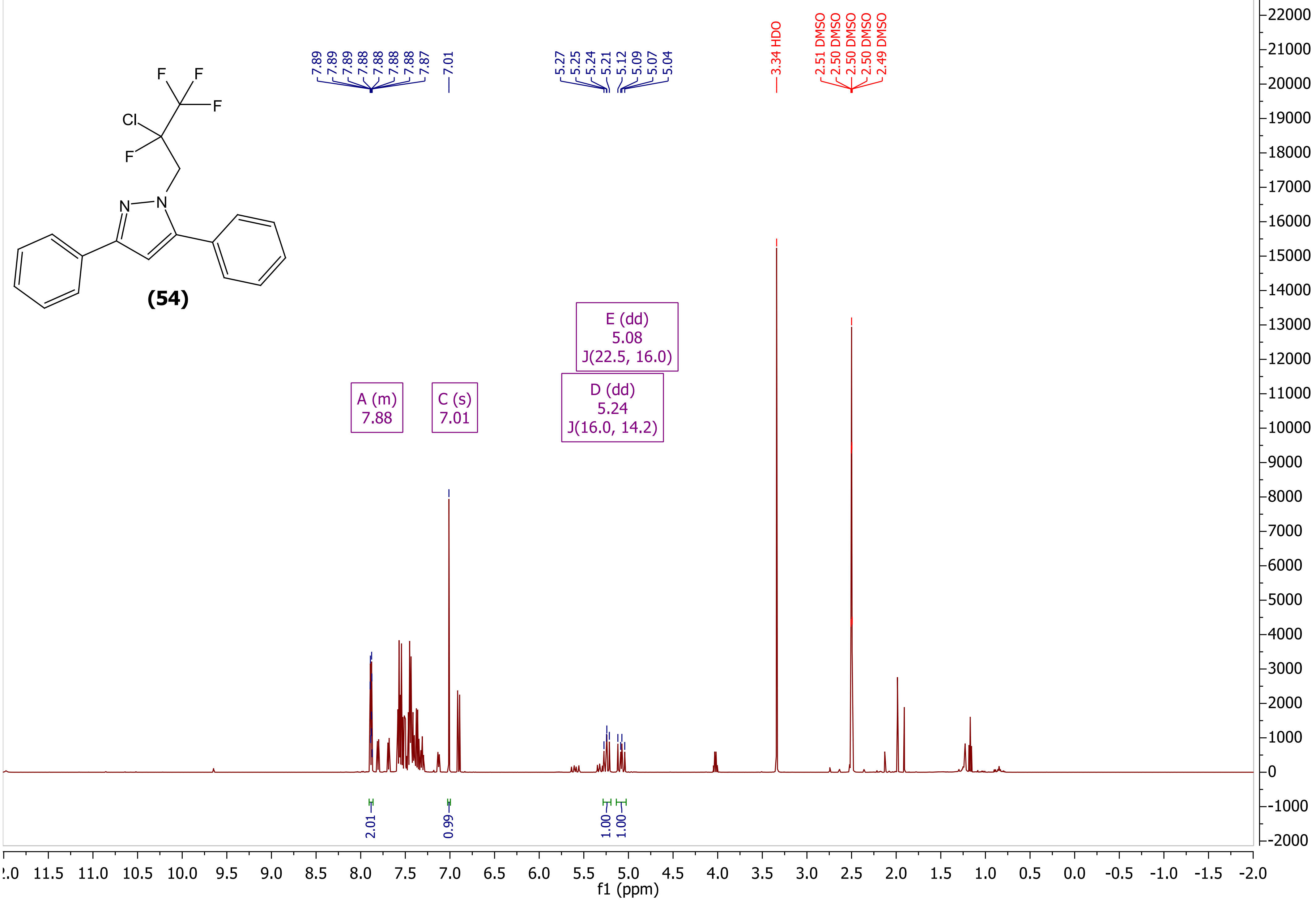

^{19}F NMR (376 MHz, DMSO-d_6) δ -80.4 (d, J = 6.4 Hz), -125.7 – -126.5 (m).

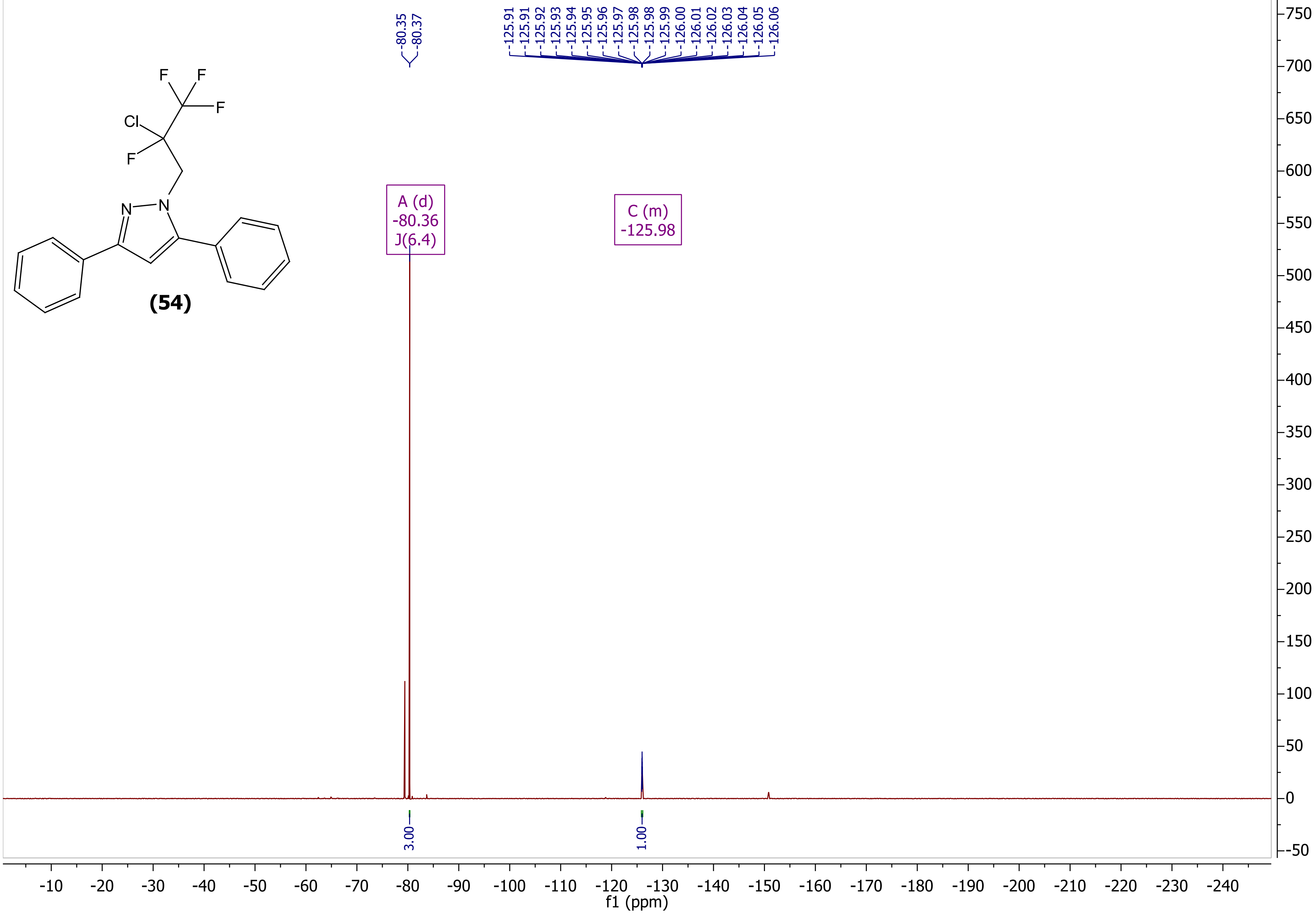

(54)

^{1}H NMR (500 MHz, DMSO-d_6) δ 7.82 – 7.79 (m, 2H), 7.70 – 7.67 (m, 2H), 7.59 – 7.29 (m), 7.15 – 7.10 (m, 2H), 6.91 (s, 1H), 6.89 (s, 1H), 5.60 (dd, J = 26.1, 15.5 Hz, 1H), 5.32 (dd, J = 15.5, 12.2 Hz, 1H).

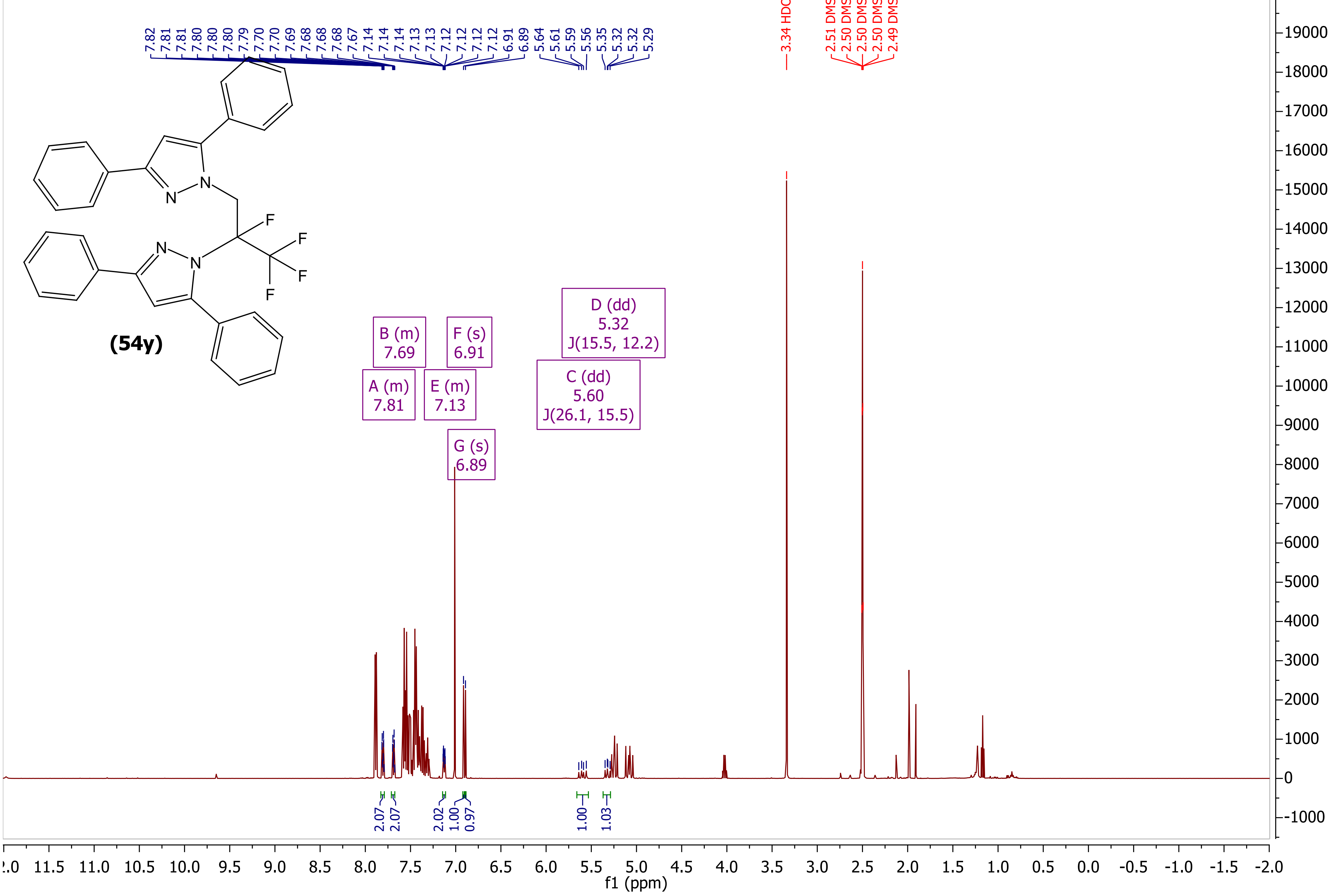

^{19}F NMR (376 MHz, DMSO-d_6) δ -79.4 (d, J = 5.3 Hz), -150.5 – -151.0 (m).

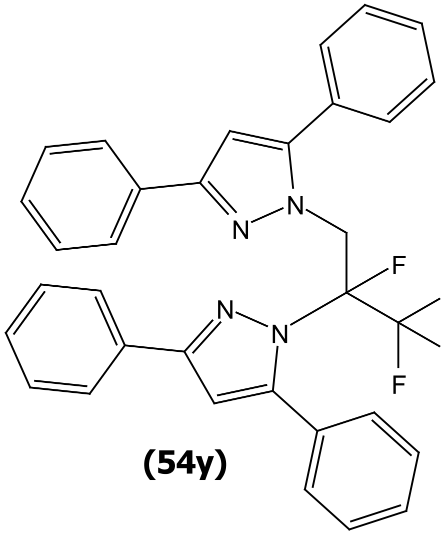

(54y)

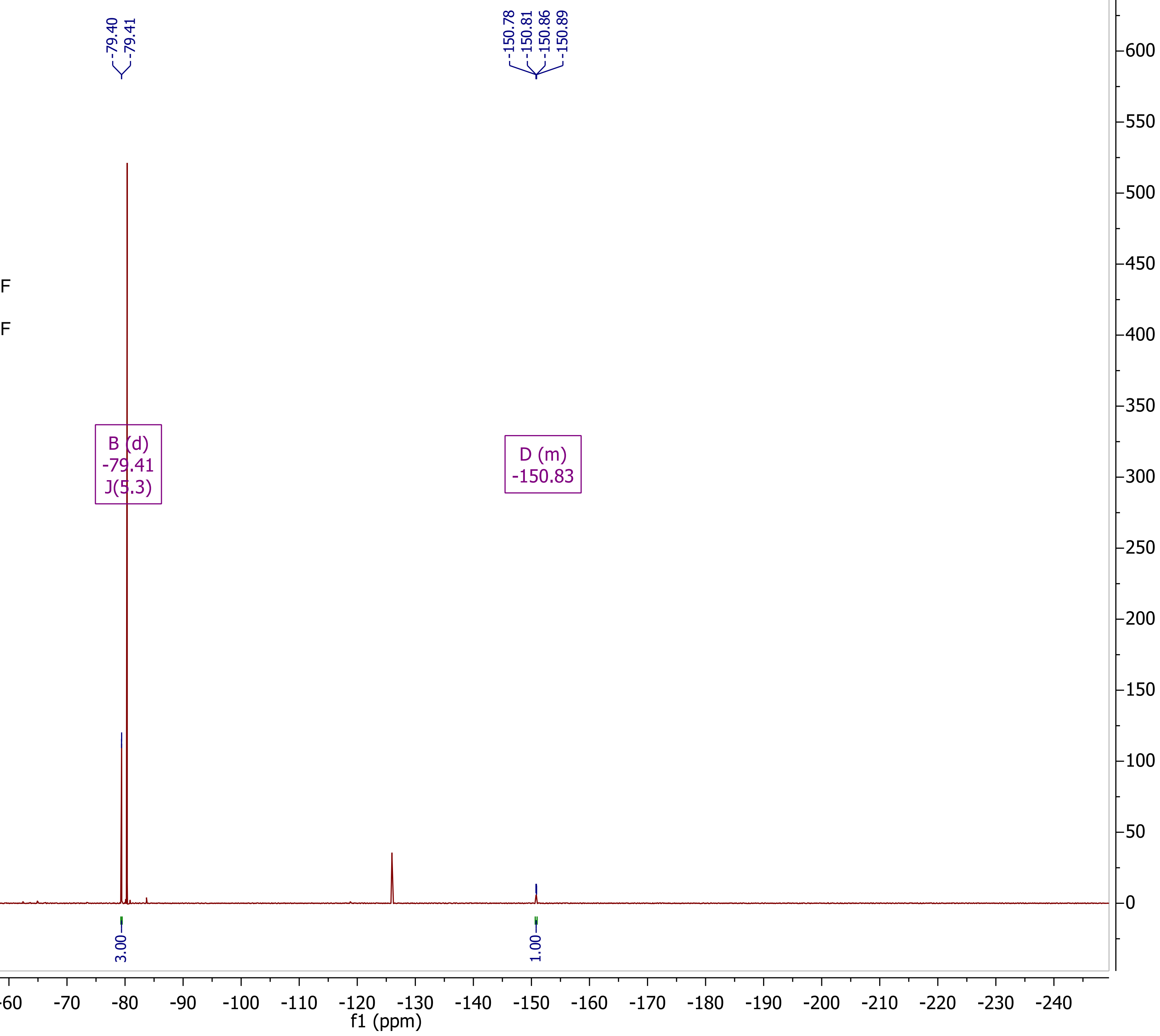

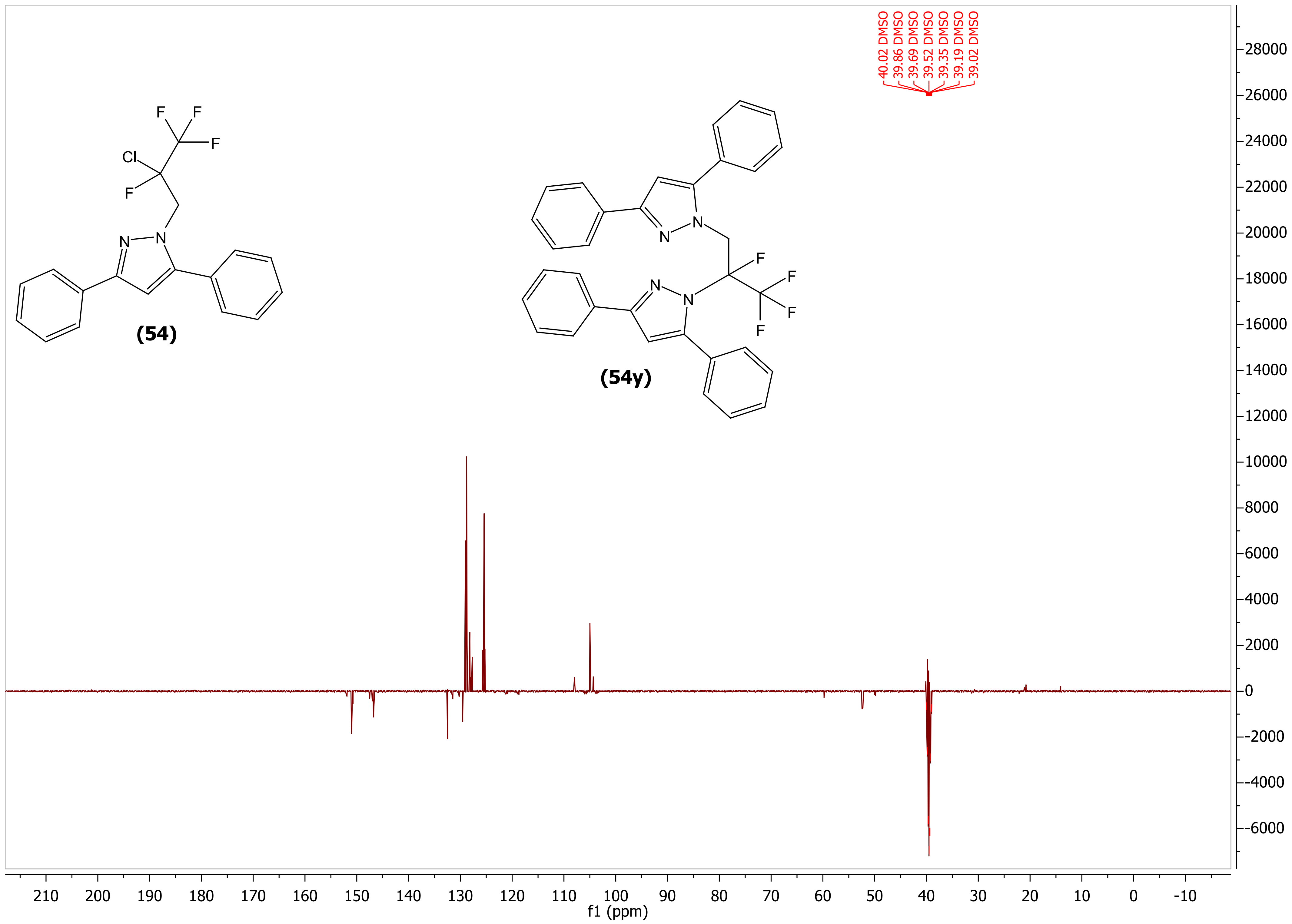

40.02 DMSO
39.86 DMSO
39.69 DMSO
39.52 DMSO
39.35 DMSO
39.19 DMSO
39.02 DMSO
(54)
(54y)
f1 (ppm)

^{1}H NMR (250 MHz, Chloroform-*d*) δ 7.84 (d, *J* = 7.1 Hz, 2H), 7.55 (s, 1H), 7.51 – 7.28 (m, 3H), 6.67 (d, *J* = 2.5 Hz, 1H), 5.07 – 4.63 (m, 2H).

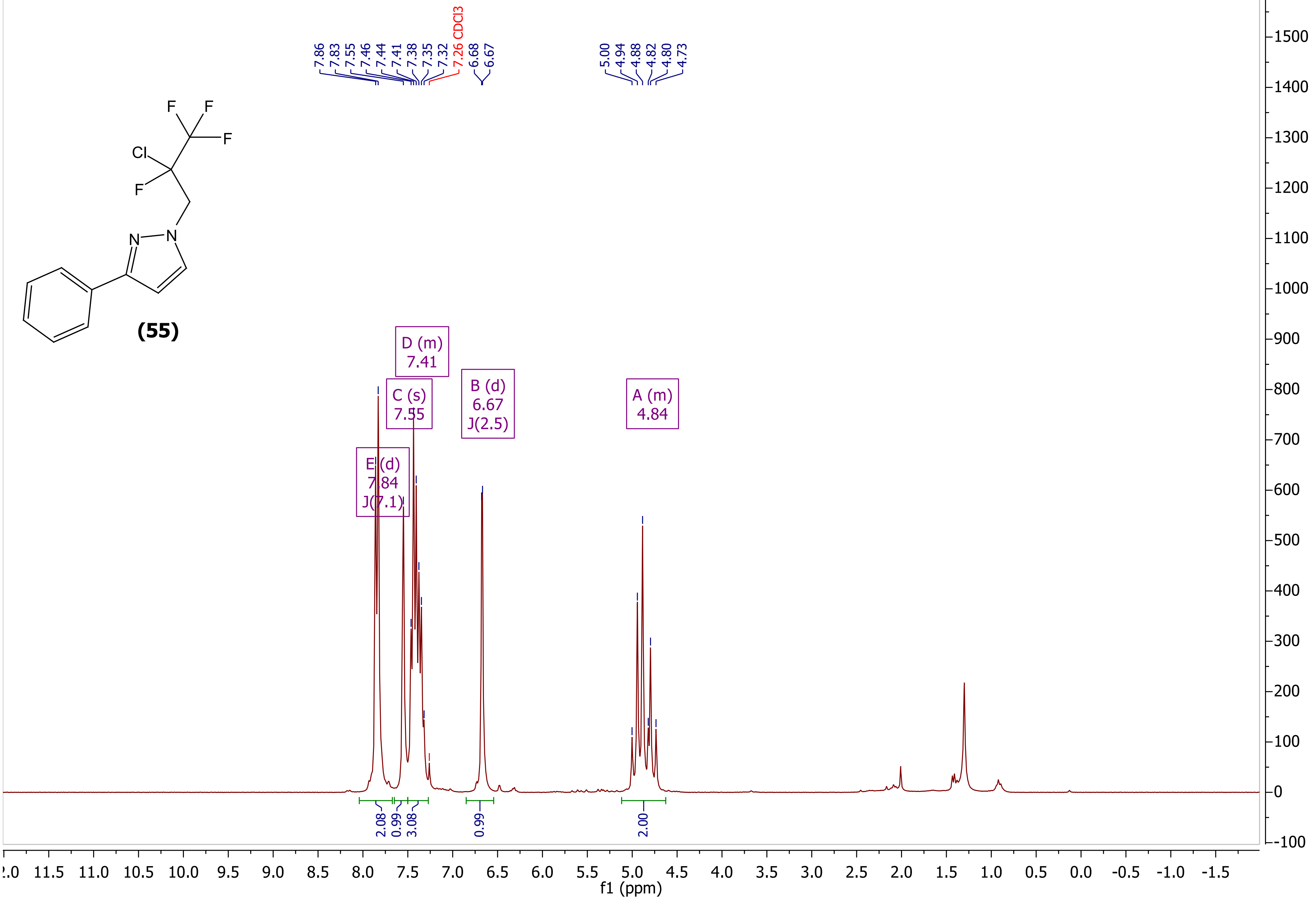

^{19}F NMR (235 MHz, Chloroform-d) δ -80.7 (d, J = 6.2 Hz), -128.8 (q, J = 6.3 Hz).

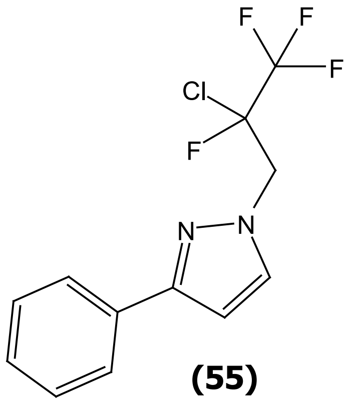

(55)

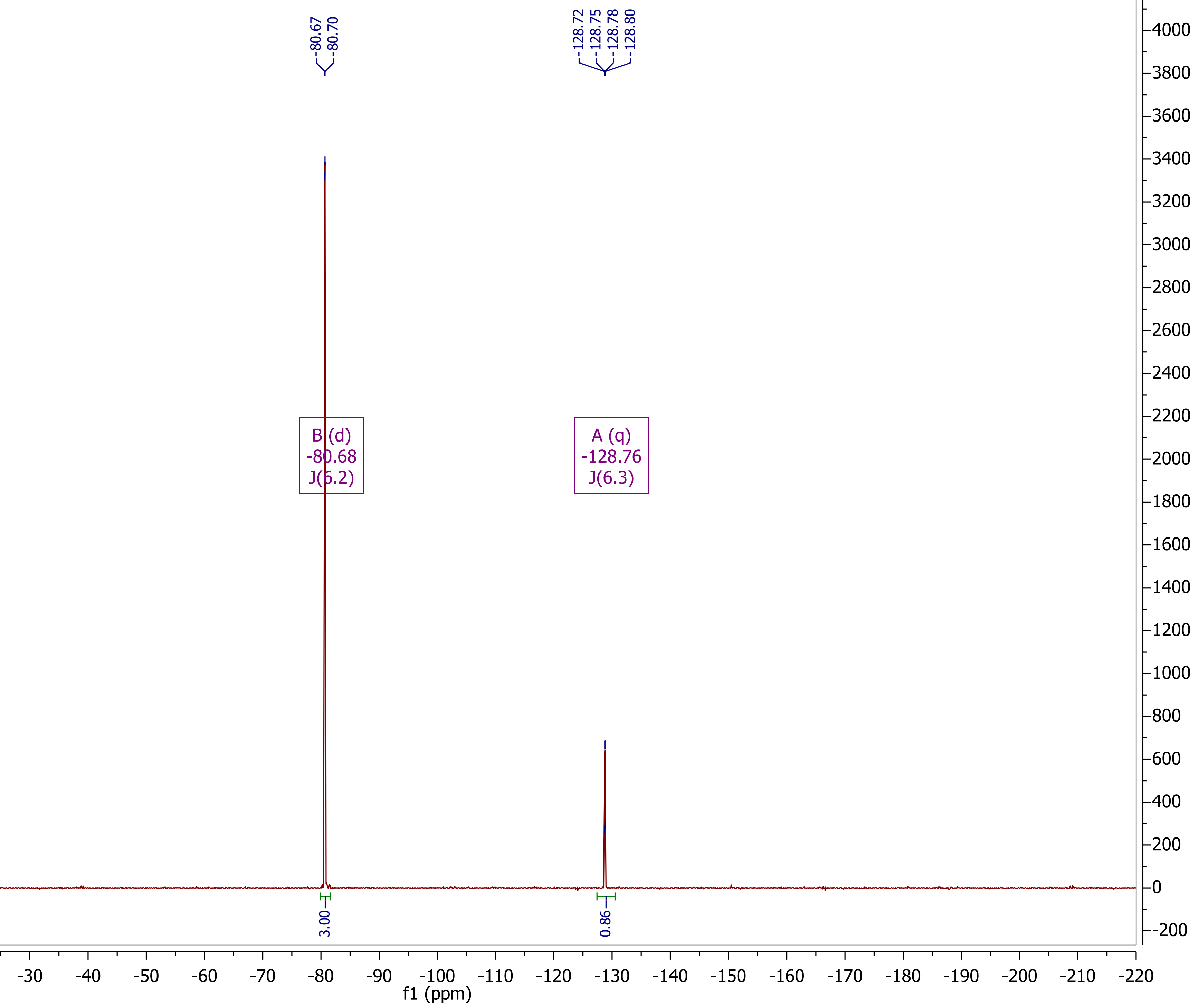

^{13}C NMR (63 MHz, Chloroform-*d*) δ 152.9, 132.9, 132.5 (d, *J* = 1.7 Hz), 128.8, 128.2, 126.0, 120.3 (qd, *J* = 285.0, 30.6 Hz), 104.8, 104.6 (dq, *J* = 256.4, 36.2 Hz), 55.8 (d, *J* = 22.3 Hz).

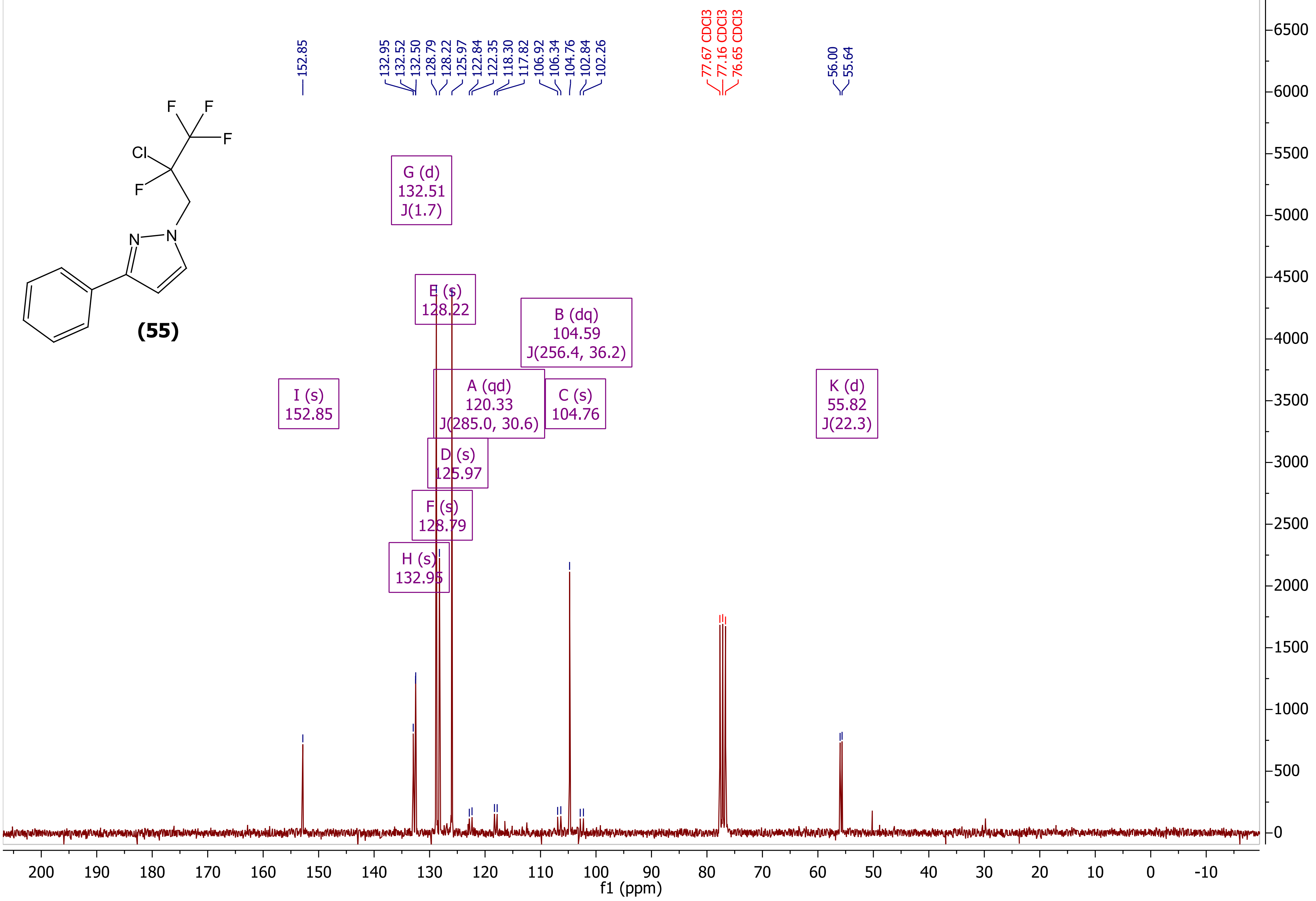

^{1}H NMR (500 MHz, DMSO-d_6) δ 7.87 (d, J = 2.3 Hz, 1H), 7.58 (dd, J = 1.9, 0.6 Hz, 1H), 6.37 – 6.36 (m, 1H), 5.31 – 5.13 (m, 2H).

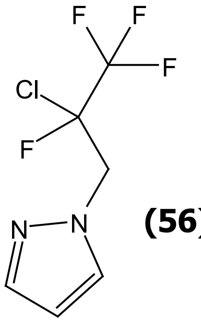

(56)

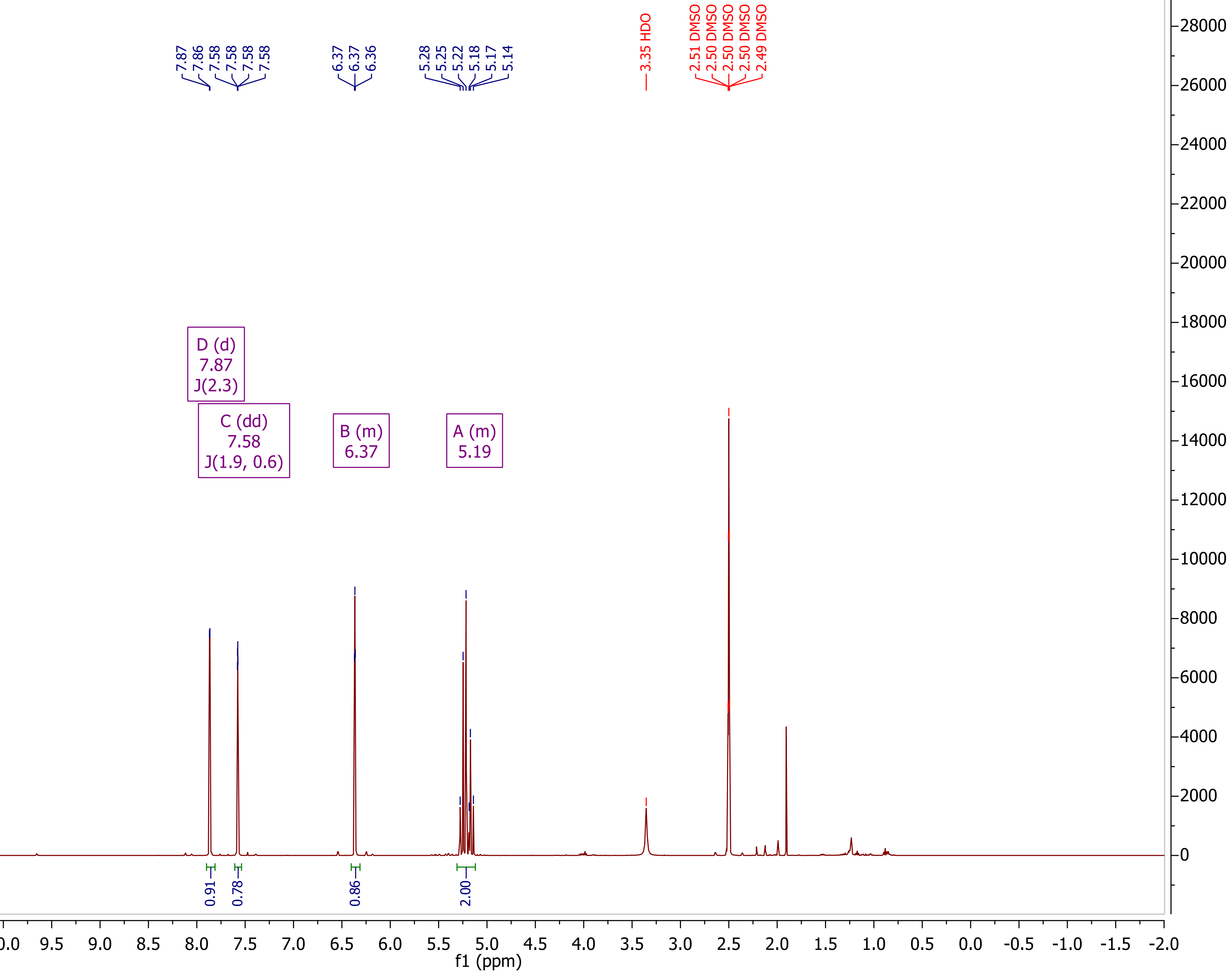

^{19}F NMR (376 MHz, DMSO-d_6) δ -79.9 (d, J = 6.6 Hz), -127.7 – -128.0 (m).

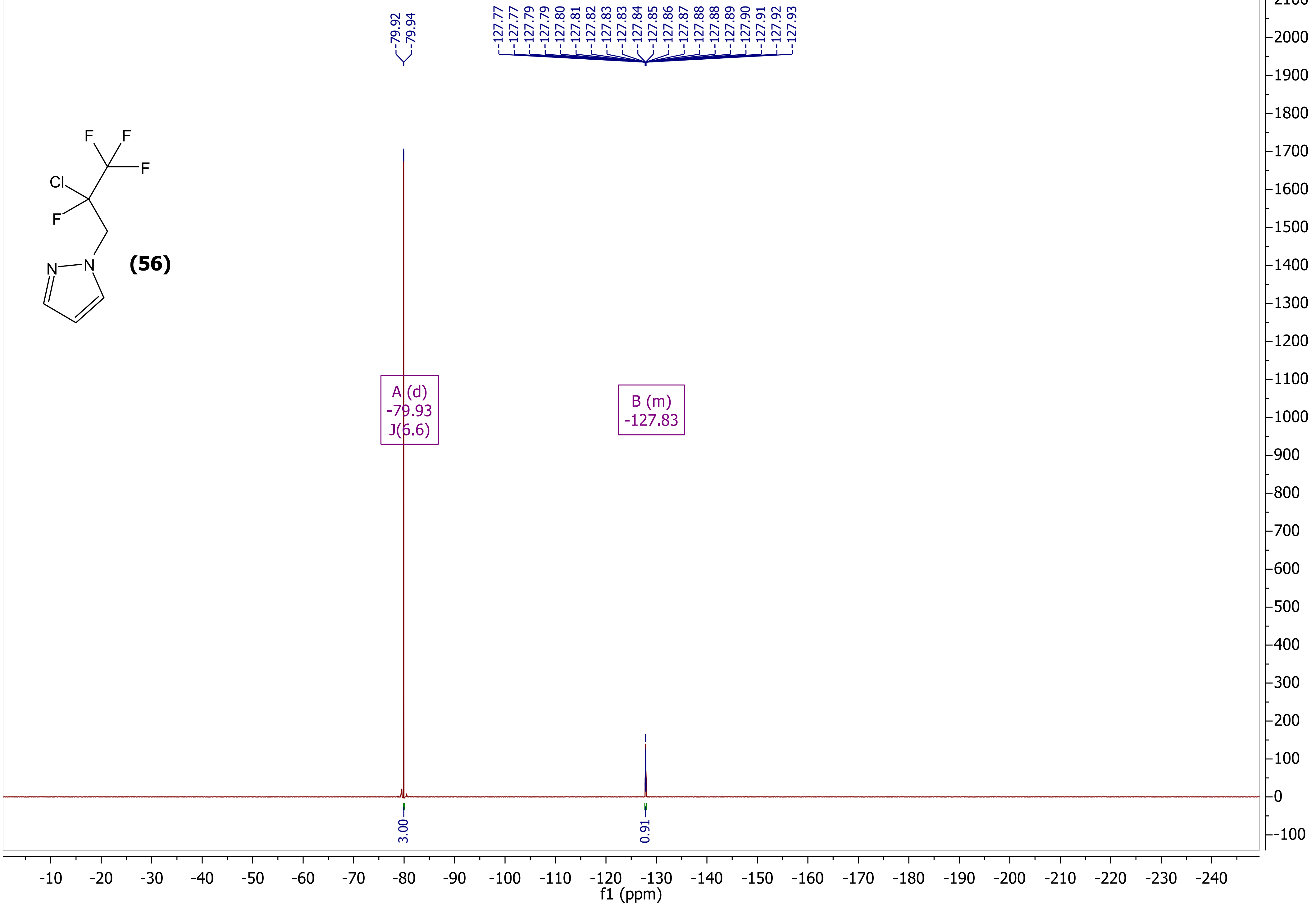

^{13}C NMR (126 MHz, DMSO-d_6) δ 140.7, 132.9 (d, J = 1.3 Hz), 120.5 (qd, J = 285.1, 31.3 Hz), 106.8, 105.2 (dq, J = 255.1, 35.8 Hz), 54.5 (d, J = 21.6 Hz).

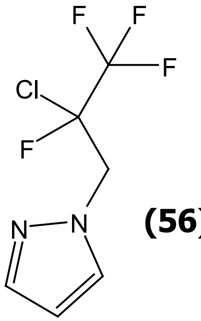

(56)

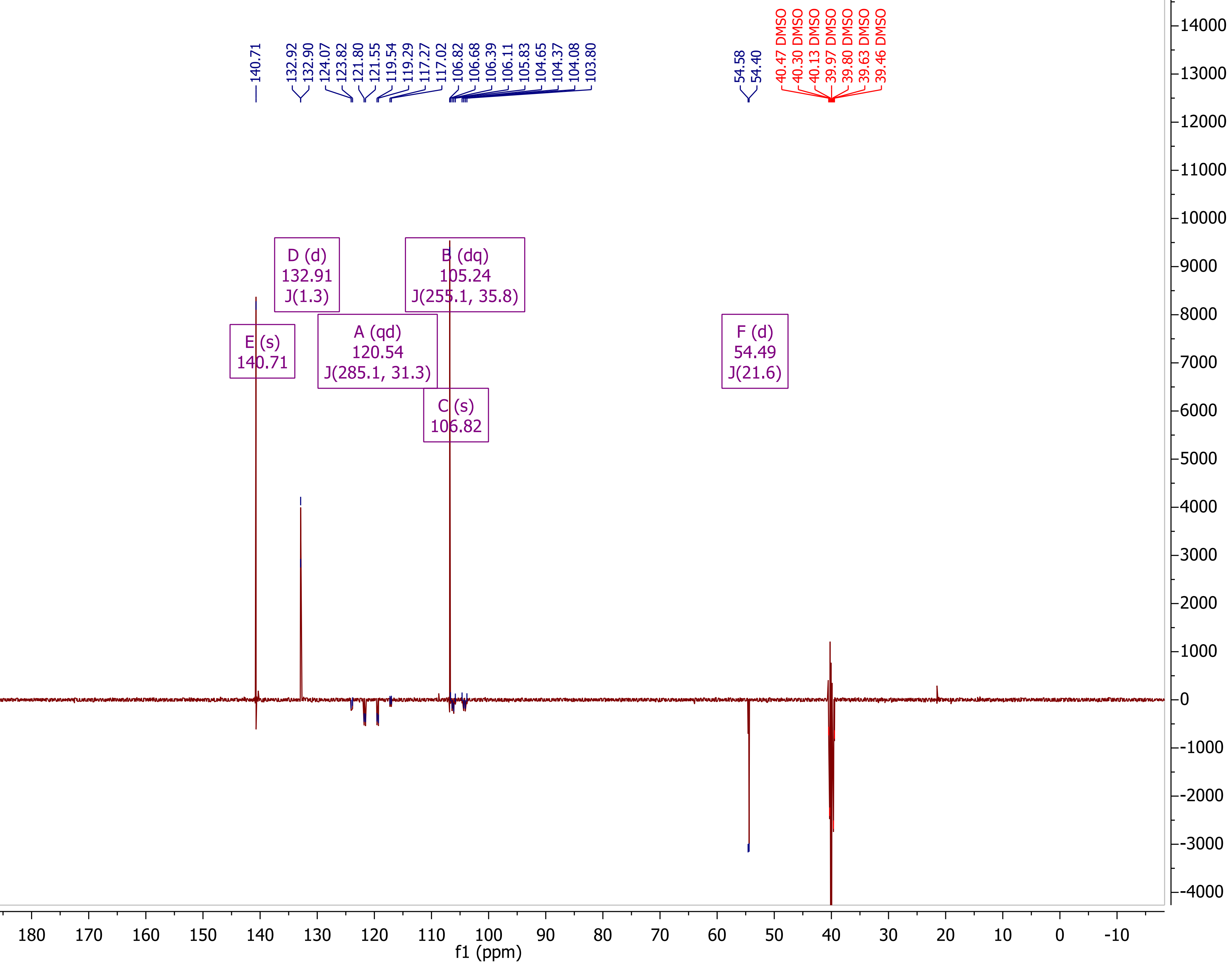

^{1}H NMR (500 MHz, DMSO-d_6) δ 11.21 (s, 1H), 7.56 (d, J = 8.0 Hz, 1H), 7.42 – 7.37 (m, 2H), 7.11 (td, J = 7.0, 1.1 Hz, 1H), 7.03 (td, J = 7.3, 1.0 Hz, 1H), 3.85 (dd, J = 15.5, 11.5 Hz, 1H), 3.69 (dd, J = 32.0, 15.5 Hz, 1H).

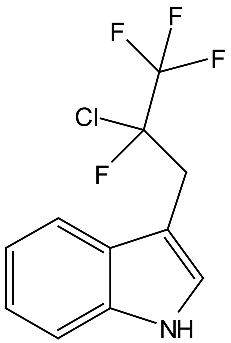

(57)

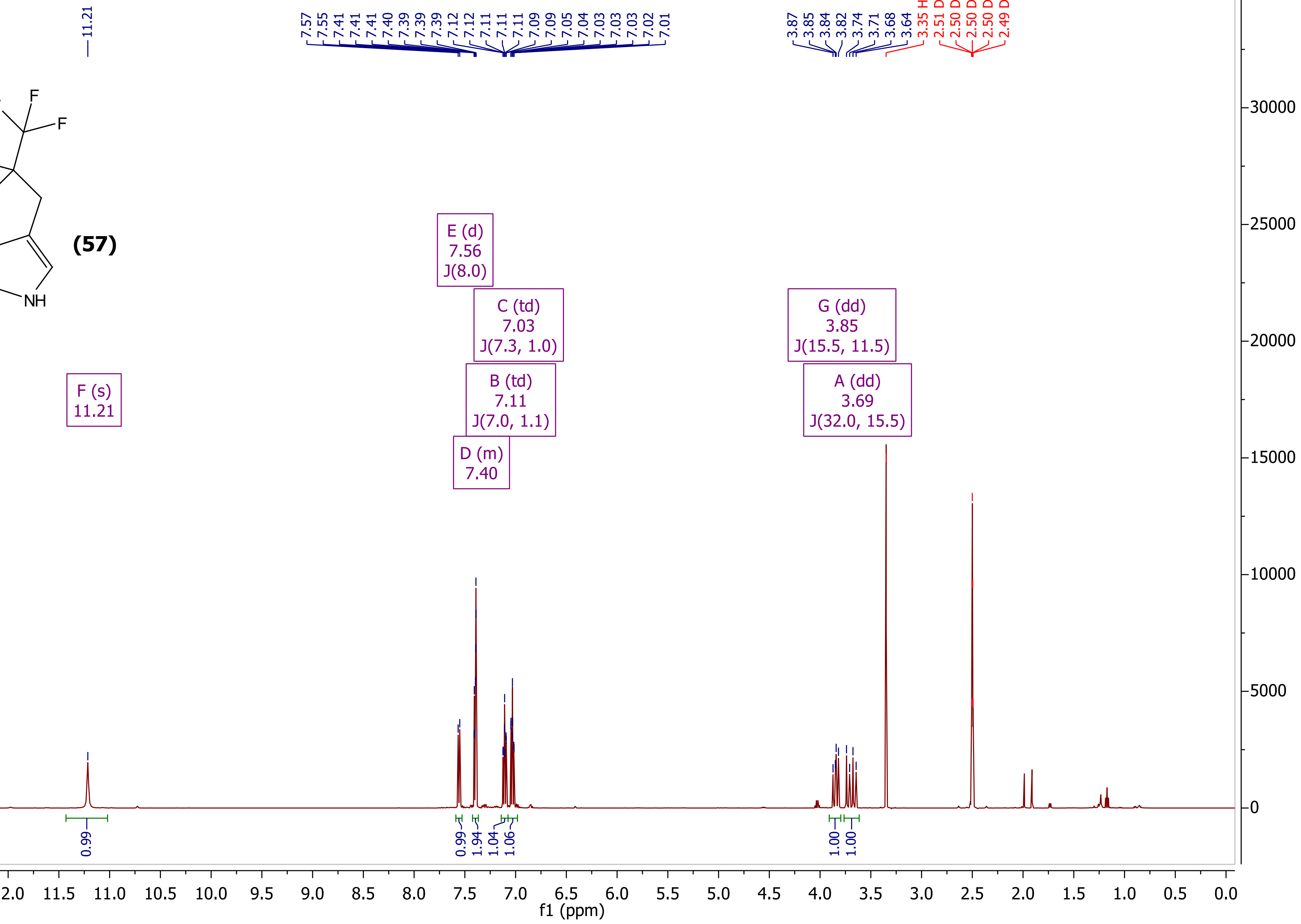

^{19}F NMR (376 MHz, DMSO-d_6) δ -80.5 (d, J = 6.4 Hz), -122.5 – -122.8 (m).

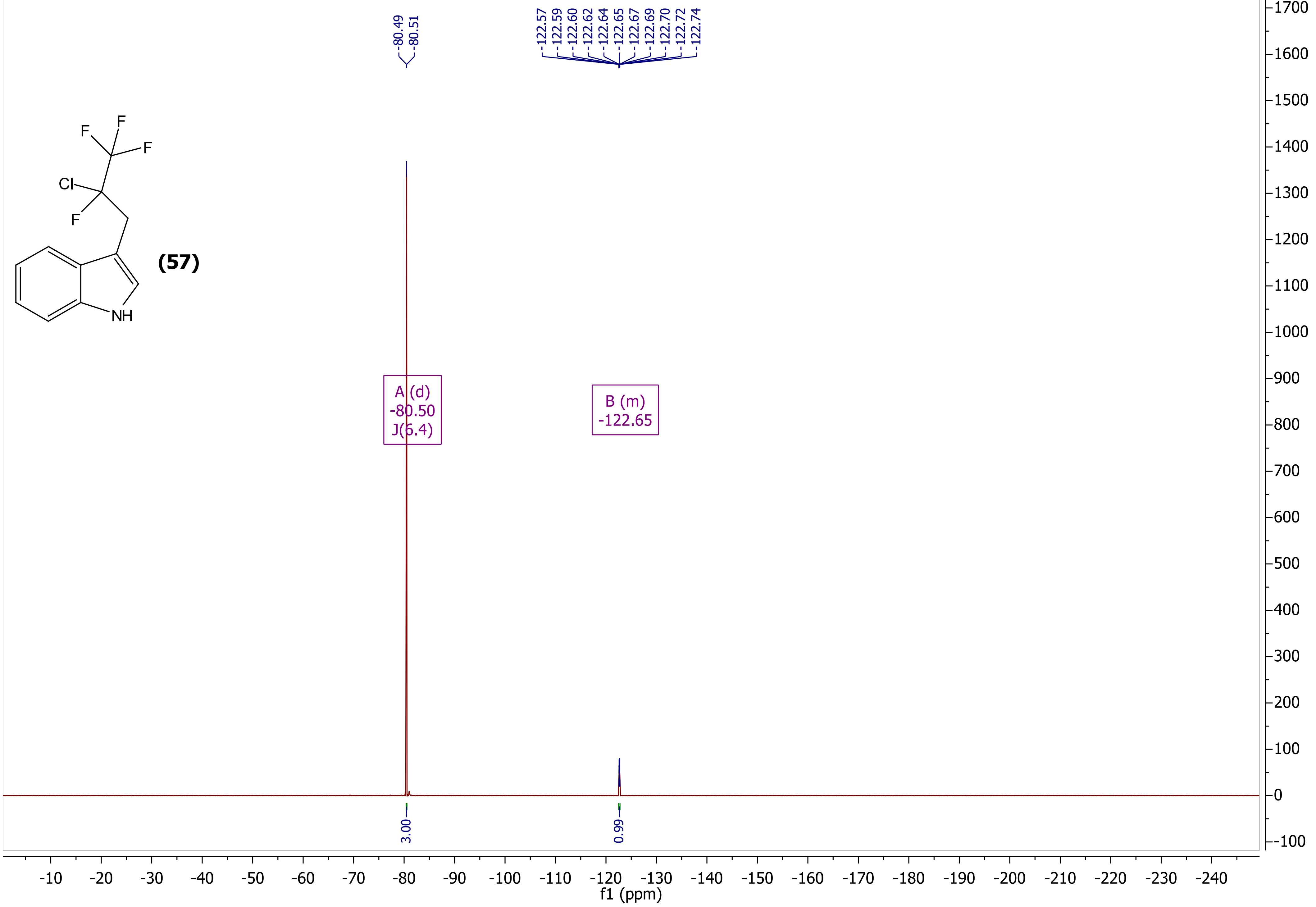

^{13}C NMR (126 MHz, DMSO-d_6) δ 136.0, 127.6, 126.6, 121.2, 121.0 (qd, J = 284.7, 32.1 Hz), 119.0, 118.6 (d, J = 2.2 Hz), 111.6, 107.9 (dq, J = 250.5, 34.4 Hz), 103.1 (d, J = 2.0 Hz), 31.9 (d, J = 21.7 Hz).

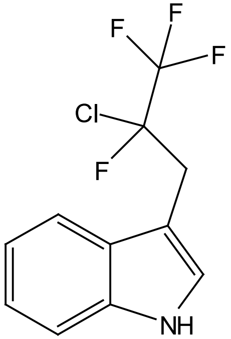

(57)

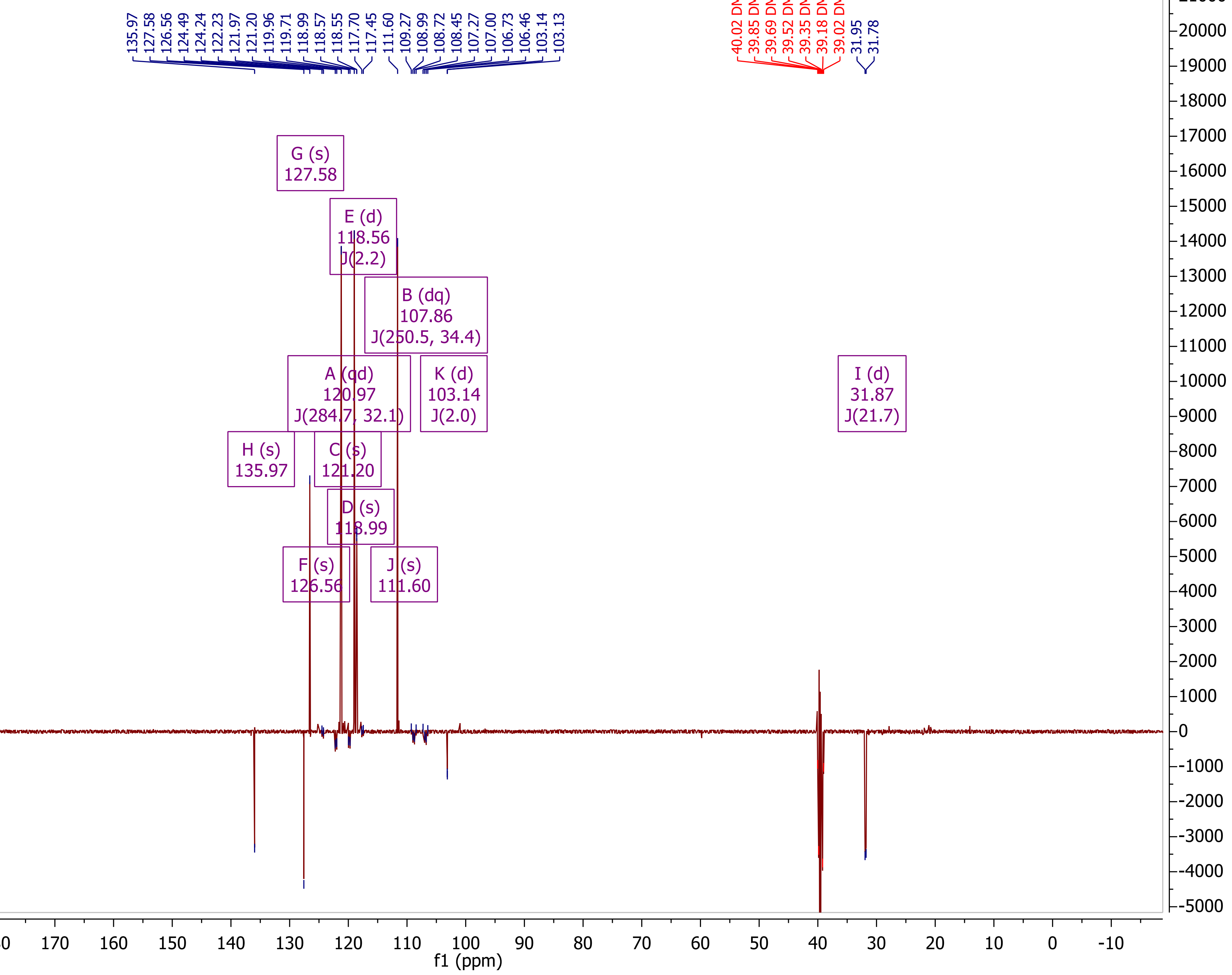

^{1}H NMR (250 MHz, Chloroform-*d*) δ 8.00 (bs, 1H), 7.65 (d, *J* = 1.9 Hz, 1H), 7.24 (dd, *J* = 8.5, 1.9 Hz, 1H), 7.14 (d, *J* = 8.4 Hz, 1H), 3.63 (dd, *J* = 15.5, 10.6 Hz, 1H), 3.42 (dd, *J* = 31.3, 15.4 Hz, 1H), 2.41 (s, 3H).

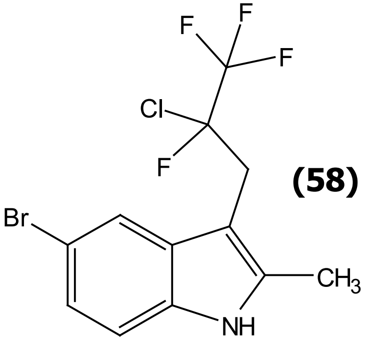

(58)

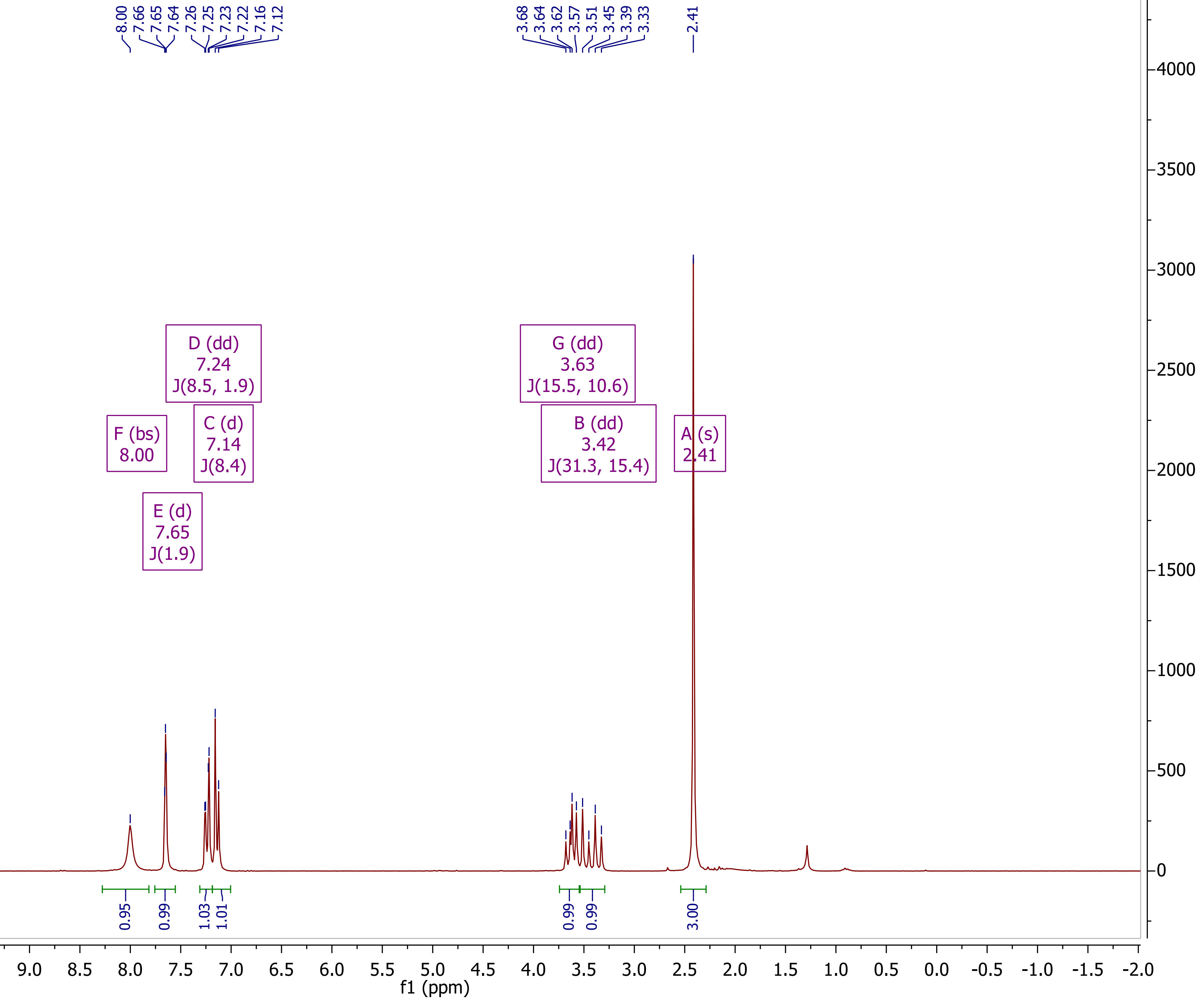

^{19}F NMR (235 MHz, Chloroform-*d*) δ -81.8 (d, *J* = 6.1 Hz), -123.4 (q, *J* = 6.1 Hz).

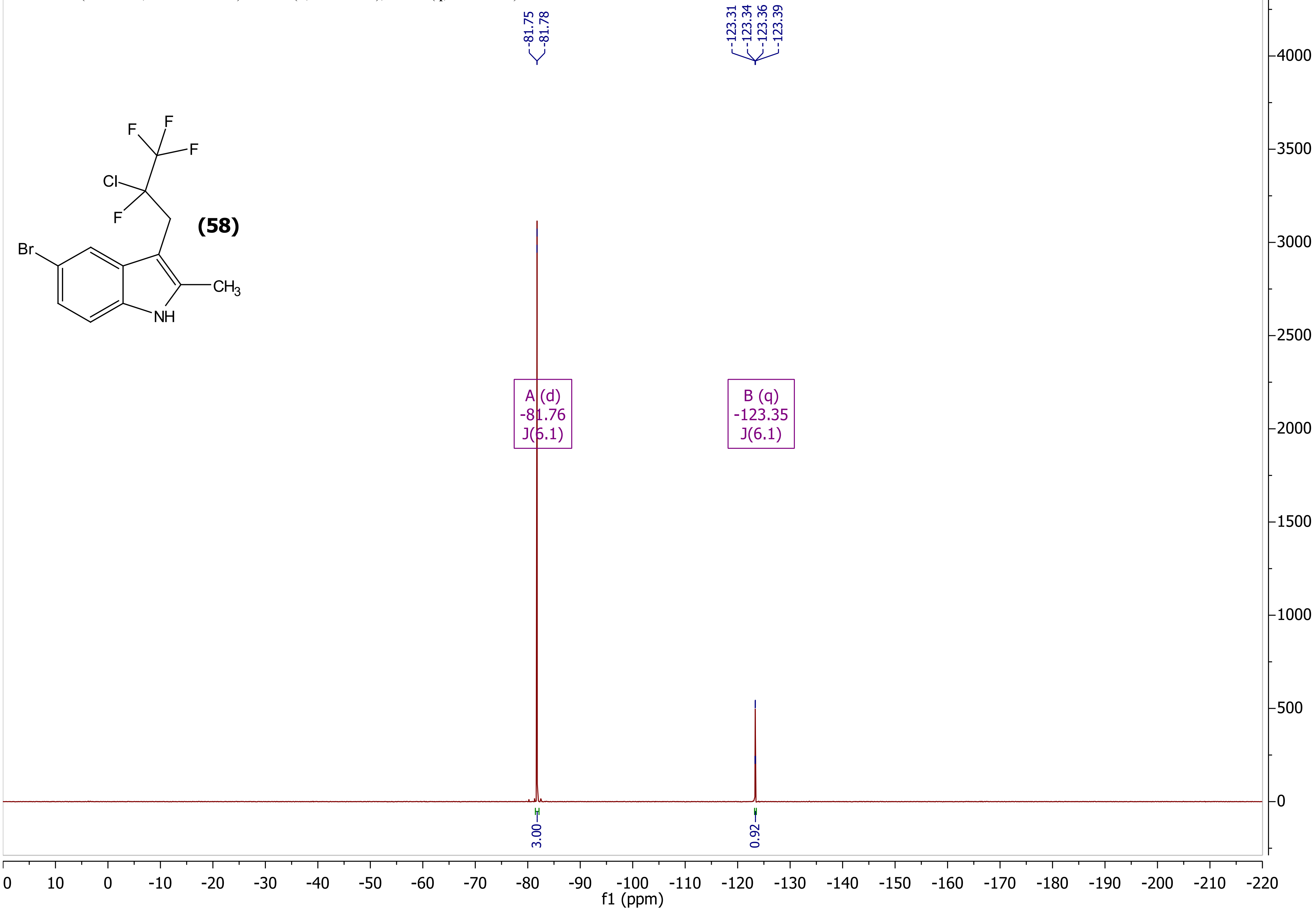

^{13}C NMR (63 MHz, Chloroform-*d*) δ 136.4, 133.8, 131.0, 124.5, 121.2 (qd, *J* = 284.6, 31.7 Hz), 121.1 (d, *J* = 2.9 Hz), 113.4, 111.9, 108.2 (dq, *J* = 252.9, 34.9 Hz), 101.5, 31.8 (d, *J* = 22.3 Hz), 12.2 (d, *J* = 1.8 Hz).

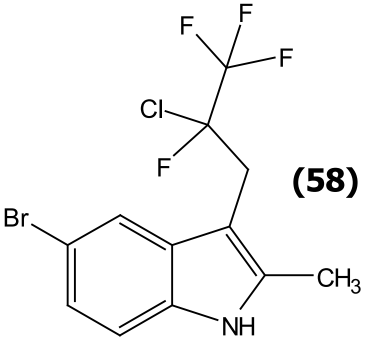

(58)

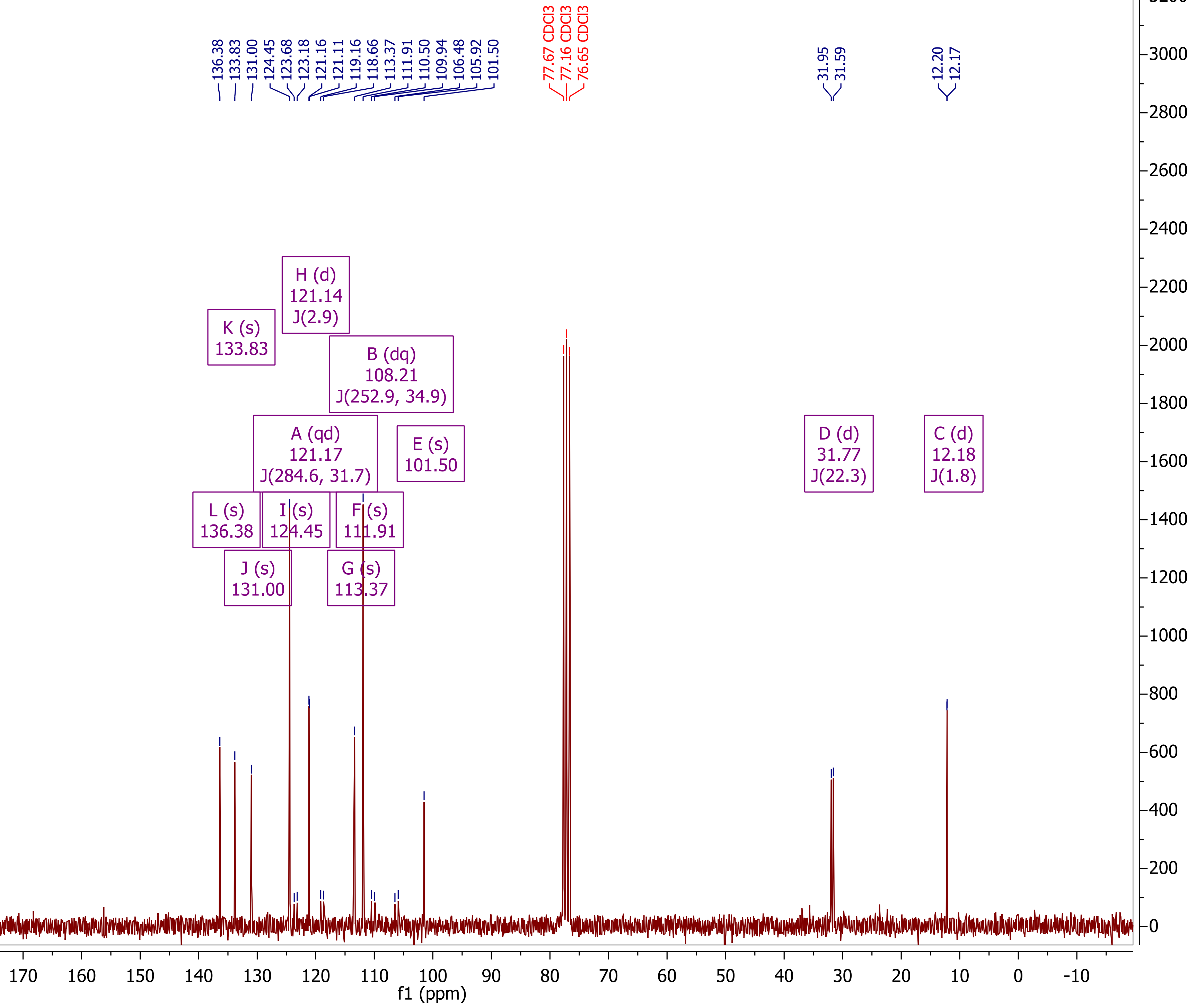

^{1}H NMR (500 MHz, DMSO-d_6) δ 11.55 (s, 1H), 7.36 (dd, J = 8.1, 0.9 Hz, 1H), 7.18 (dd, J = 7.6, 0.9 Hz, 1H), 6.95 (t, J = 7.8 Hz, 1H), 4.12 – 3.96 (m, 2H), 2.76 (q, J = 7.6 Hz, 2H), 1.27 (t, J = 7.6 Hz, 3H).

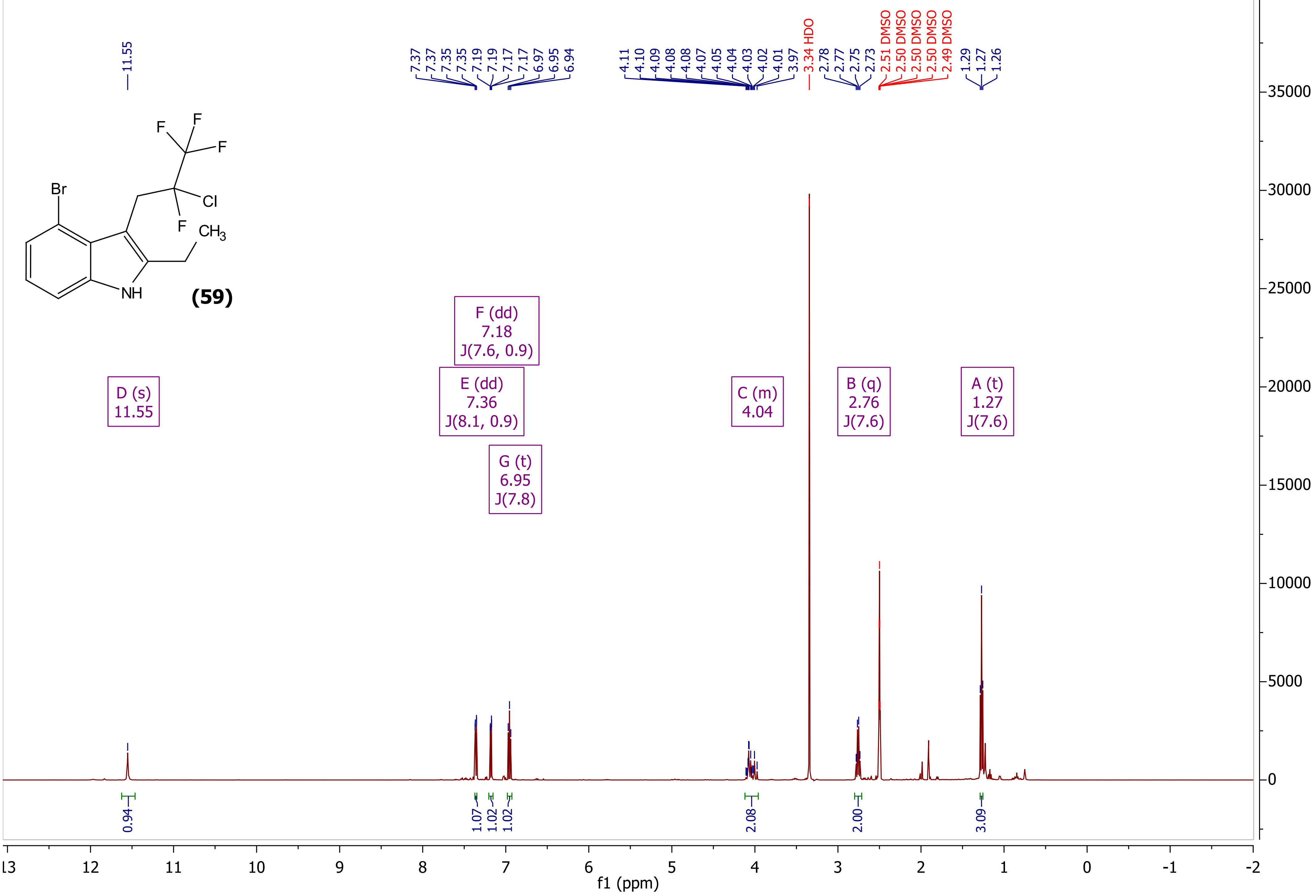

^{19}F NMR (376 MHz, DMSO-d_6) δ -80.9 (d, J = 6.7 Hz), -121.7 – -122.1 (m).

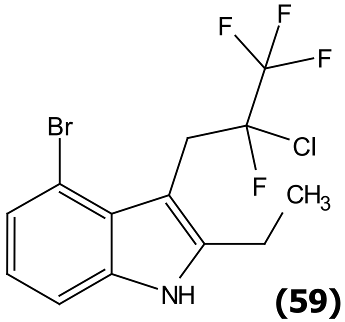

(59)

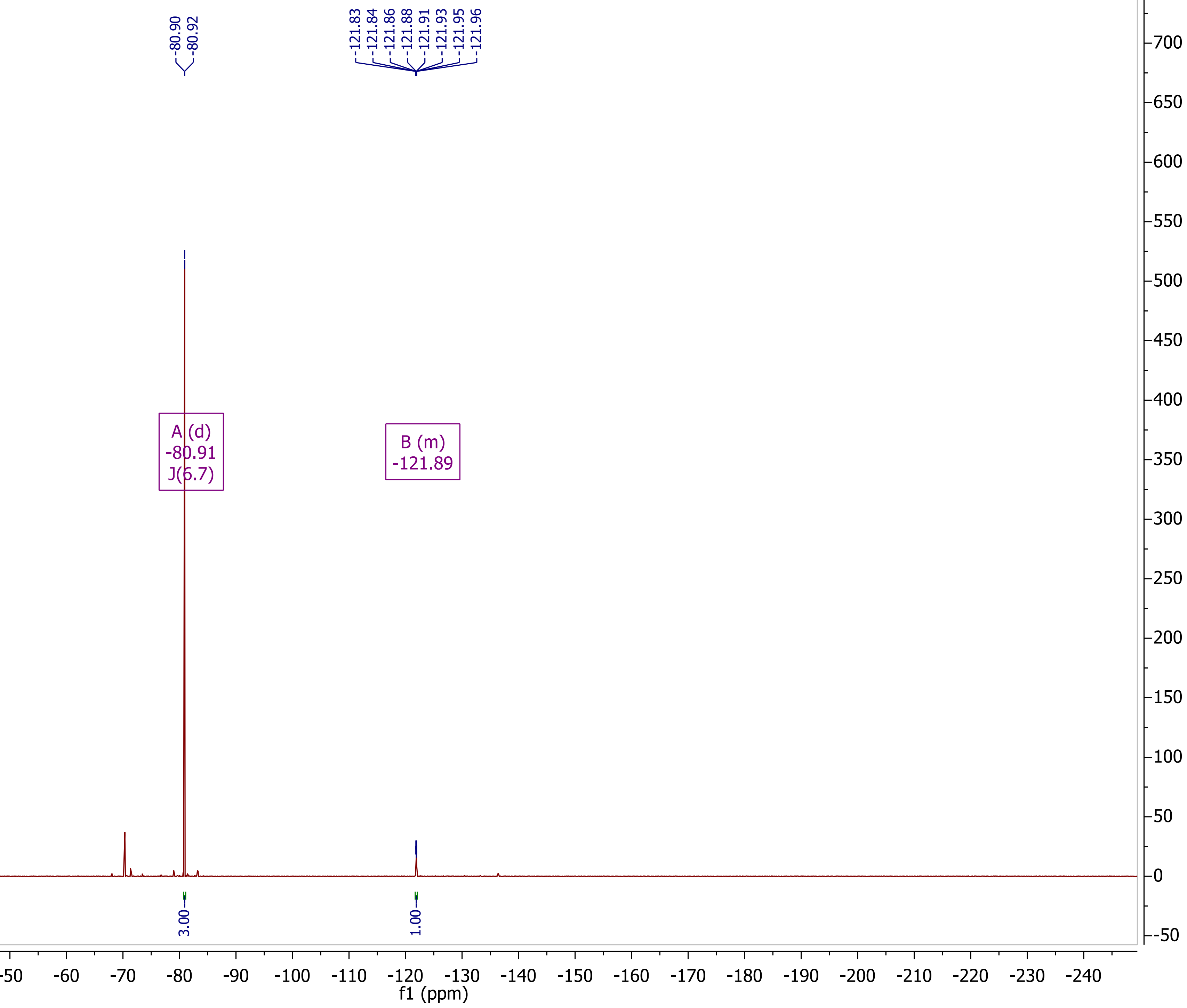

^{13}C NMR (126 MHz, DMSO-d_6) δ 143.4, 137.0, 125.6, 123.8, 121.6, 121.0 (qd, J = 284.9, 32.1 Hz), 112.0, 110.8, 108.1 (dq, J = 251.6, 34.1 Hz), 98.7, 29.5 (d, J = 20.4 Hz), 19.2 (d, J = 2.6 Hz), 13.5.

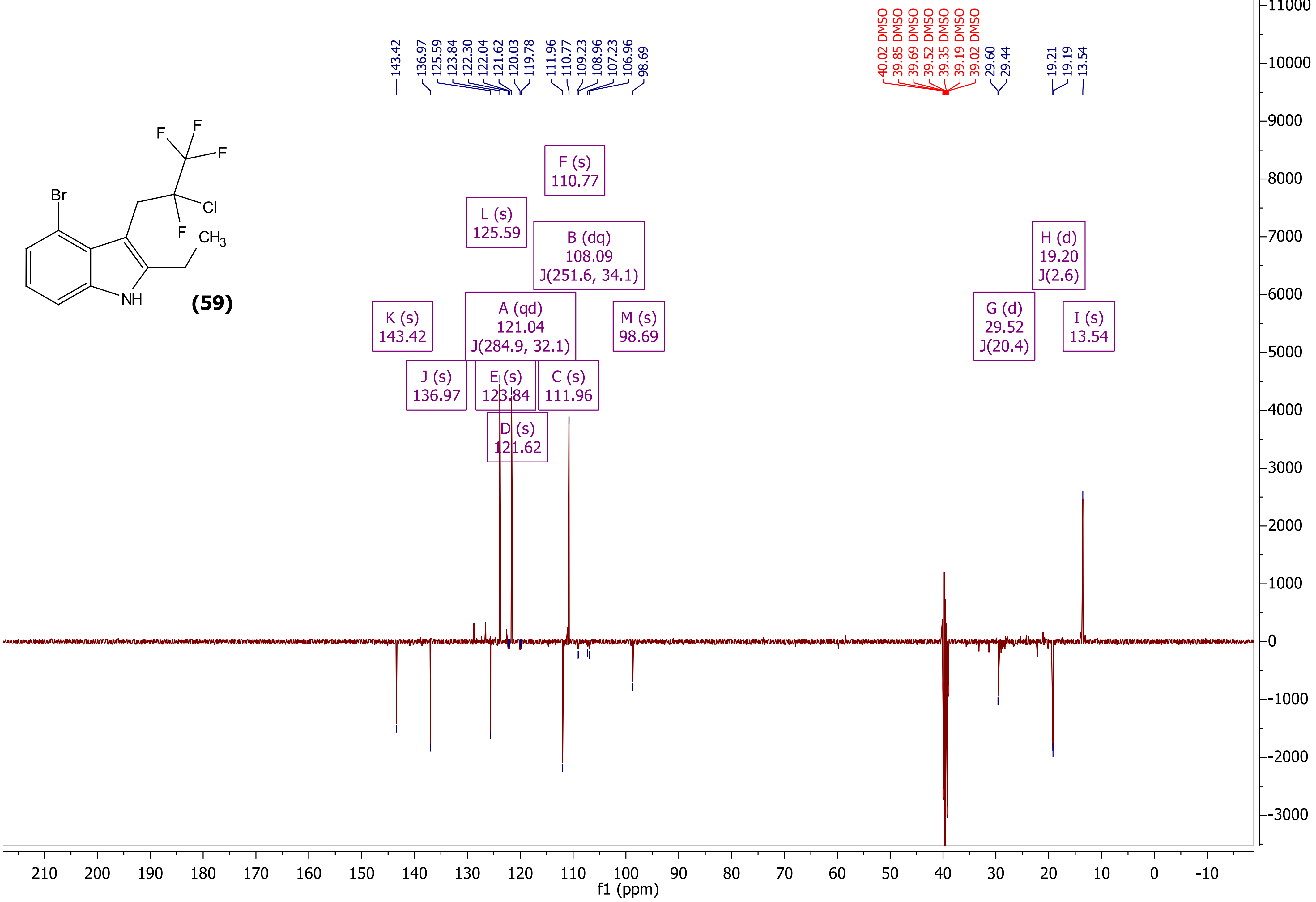

^{1}H NMR (500 MHz, DMSO-d_6) δ 7.57 (dt, J = 7.9, 1.1 Hz, 1H), 7.44 (dt, J = 8.2, 0.9 Hz, 1H), 7.39 (s, 1H), 7.18 (td, J = 7.1, 1.1 Hz, 1H), 7.07 (td, J = 7.1, 1.1 Hz, 1H), 3.84 (dd, J = 15.5, 11.4 Hz, 1H), 3.80 (s, 3H), 3.69 (dd, J = 32.0, 15.5 Hz, 1H).

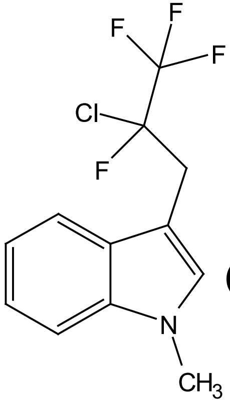

(60)

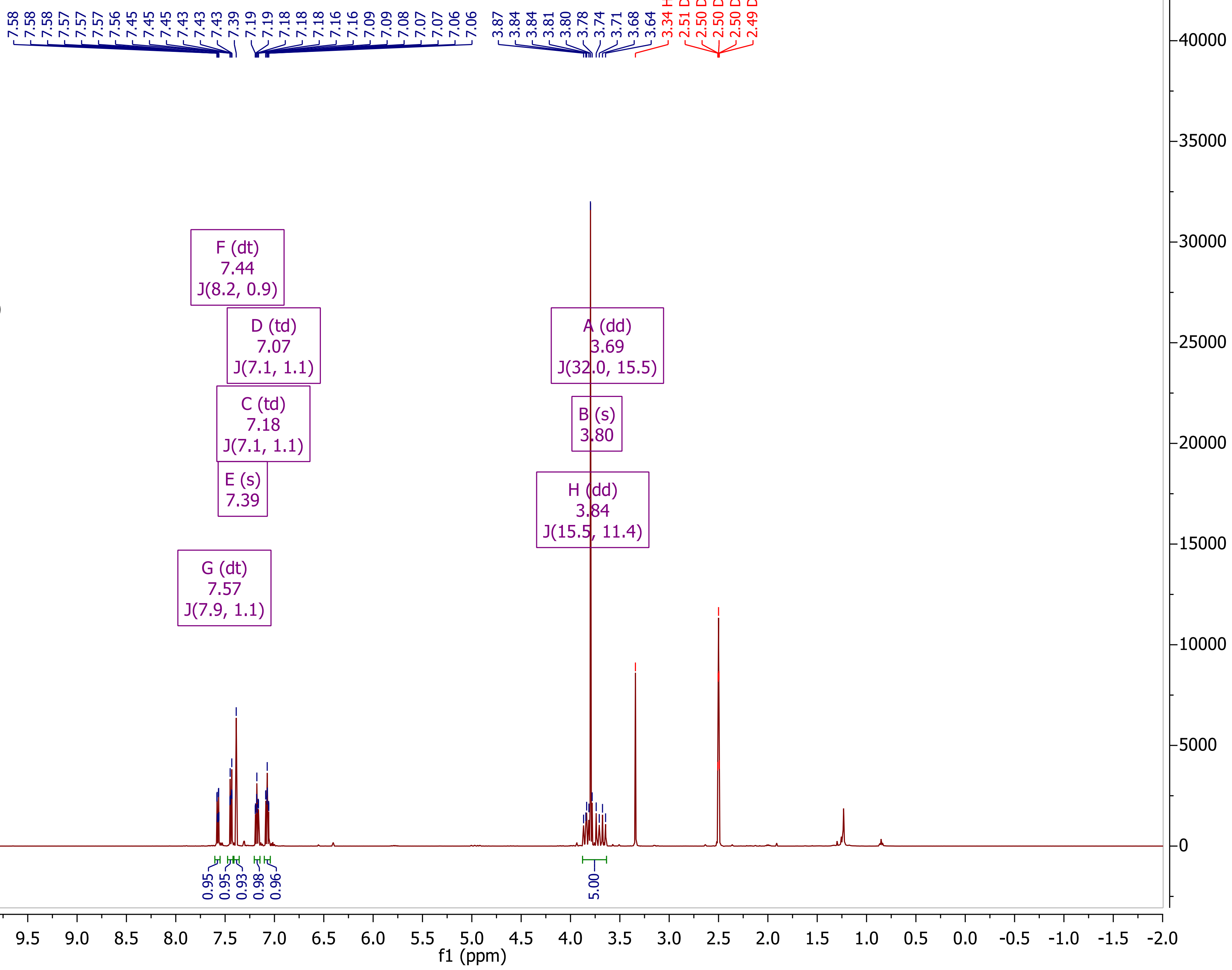

^{19}F NMR (376 MHz, DMSO-d_6) δ -80.5 (d, J = 6.5 Hz), -122.6 – -122.9 (m).

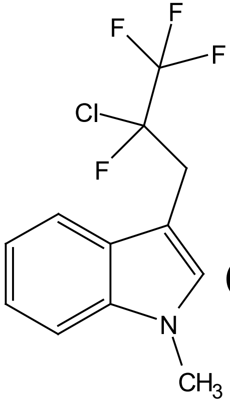

(60)

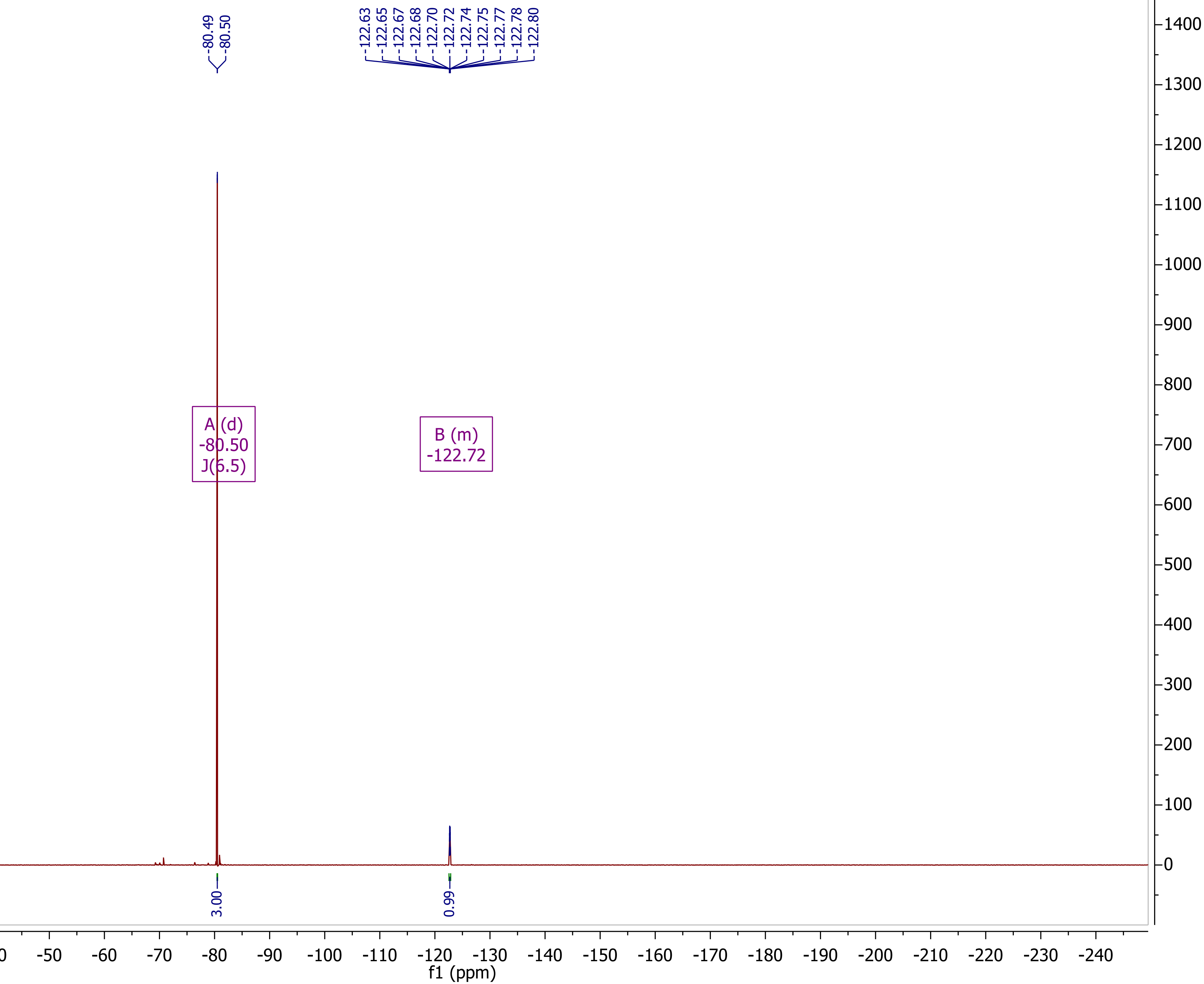

^{13}C NMR (126 MHz, DMSO-d_6) δ 136.4, 130.6, 127.9, 121.3, 120.9 (qd, J = 284.7, 32.2 Hz), 119.1, 118.8 (d, J = 2.2 Hz), 109.8, 107.7 (dq, J = 250.8, 34.7 Hz), 102.3 (d, J = 2.1 Hz), 32.5, 31.7 (d, J = 21.7 Hz).

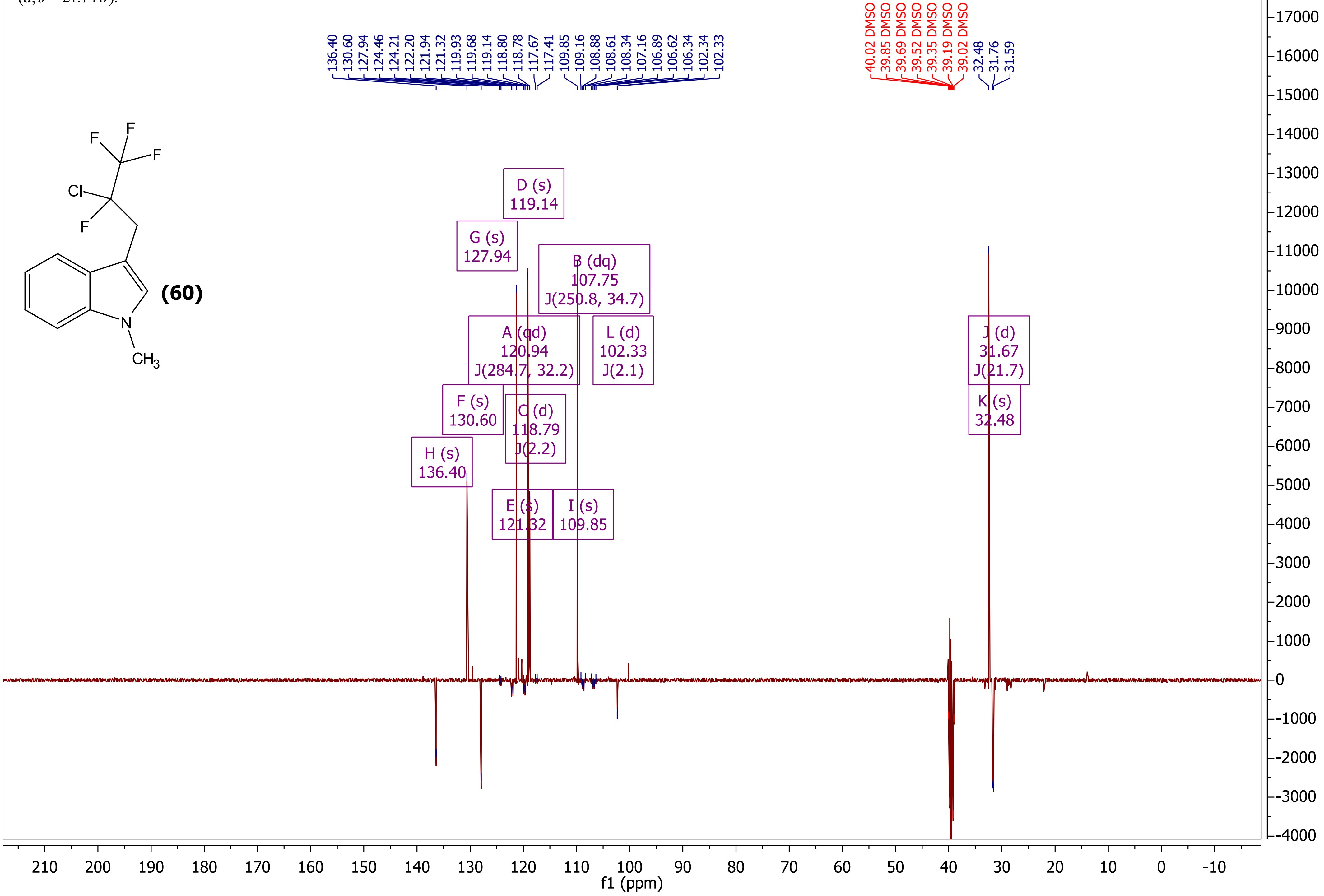

Supplement: Supplementary file 2 — Supporting Information [file ANIE-61-0-s001.pdf]
